# Accessing Medium-Sized Bridged Heterocycles via EnT-Catalyzed Intermolecular Dearomative (5 + 4) Cycloaddition of Furans and Oxazoles

**DOI:** 10.1021/jacs.6c00671

**Published:** 2026-06-16

**Authors:** Carla Hümpel, Debanjan Rana, Sophie Korgitzsch, Kiana Fischer, Constantin G. Daniliuc, Frank Glorius

**Affiliations:** Organisch-Chemisches Institut, 9185 Universität Münster, Corrensstraße 36, 48149 Münster, Germany

## Abstract

Medium-sized bridged heterocycles are highly attractive structural motifs in bioactive natural products and medicinal chemistry. However, their broader exploration remains limited due to the lack of concise and modular synthetic strategies, as their synthesis is challenged by unfavorable enthalpic and entropic constraints. Herein, we report an energy transfer (EnT)-catalyzed intermolecular (5 + 4) dearomative cycloaddition of furans with vinyl cyclopropanes, providing direct access to (*Z*)-10-oxabicyclo­[5.2.1]­deca-3,8-diene scaffolds in a single step. Visible light triplet sensitization unlocks a distinct diene-type 1,4-biradical activation mode in furans. This triplet-state reactivity enables intermolecular higher-order (5 + 4) cycloadditions that remain inaccessible under conventional thermal activation. Mechanistic investigations support an energy transfer pathway and provide insights into the origin of the observed regioselectivity. The resulting partially unsaturated cycloadducts offer multiple synthetic handles for downstream functionalization, enabling rapid and modular incorporation of heterobicyclo[5.2.1]­alkane motifs and highlighting their potential as versatile building blocks in synthetic chemistry.

## Graphical abstract

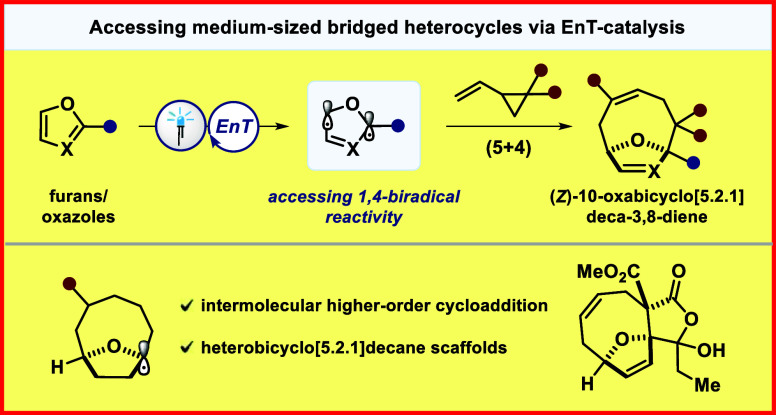

## Introduction

Medium-sized bridged heterocycles represent an important class of structural motifs that frequently occur in bioactive natural products and pharmaceutically relevant compounds.^1^ Compared to their smaller and larger counterparts, these frameworks strike a balance between three-dimensional rigidity and conformational flexibility, features that often translate into improved binding specificity to biological targets and enhanced pharmacokinetic performance.^2^ Among them, the heterobicyclo­[n.2.1]­alkane scaffold is a notable example, found in numerous natural products such as physalins, which exhibit diverse biological activities including cytotoxic and anti-inflammatory effects (Figure 1A,B).^3^ While the synthesis of smaller bridged bicyclic systems such as bicyclo[2.1.1]­hexanes and bicyclo[3.1.1]­heptanes has received tremendous attention as valuable (hetero)­arene (bio)­isosteres^4^ and witnessed remarkable progress,^5^ efficient synthetic approaches toward medium-sized bridged heterocycles remain scarce. The challenges associated with constructing medium-sized rings stem from both enthalpic and entropic constraints. Enthalpically, 8–12 membered rings suffer from pronounced transannular interactions that increase ring strain, additionally their formation through the cyclization of acyclic precursors is entropically disfavored (Figure 1C).^6^ To circumvent these barriers, most reported approaches rely on intramolecular cyclizations,^7,8^ employing methods such as ring-closing olefin metathesis,^9^ lactonization,^10^ or on approaches that involve ring expansion from smaller ring precursors.^7,11^ Although these transformations have demonstrated utility in diverse synthetic applications, including the synthesis of natural products,^7,12^ they typically require prefunctionalized substrates accessible only through lengthy multistep sequences. As a result, despite their promising biological importance, medium-sized rings remain underrepresented in pharmaceutical screening libraries,^13,14^ highlighting a clear gap in the development of concise, single-step, intermolecular strategies to assemble these frameworks from simple and readily available starting materials.

**Figure 1:**
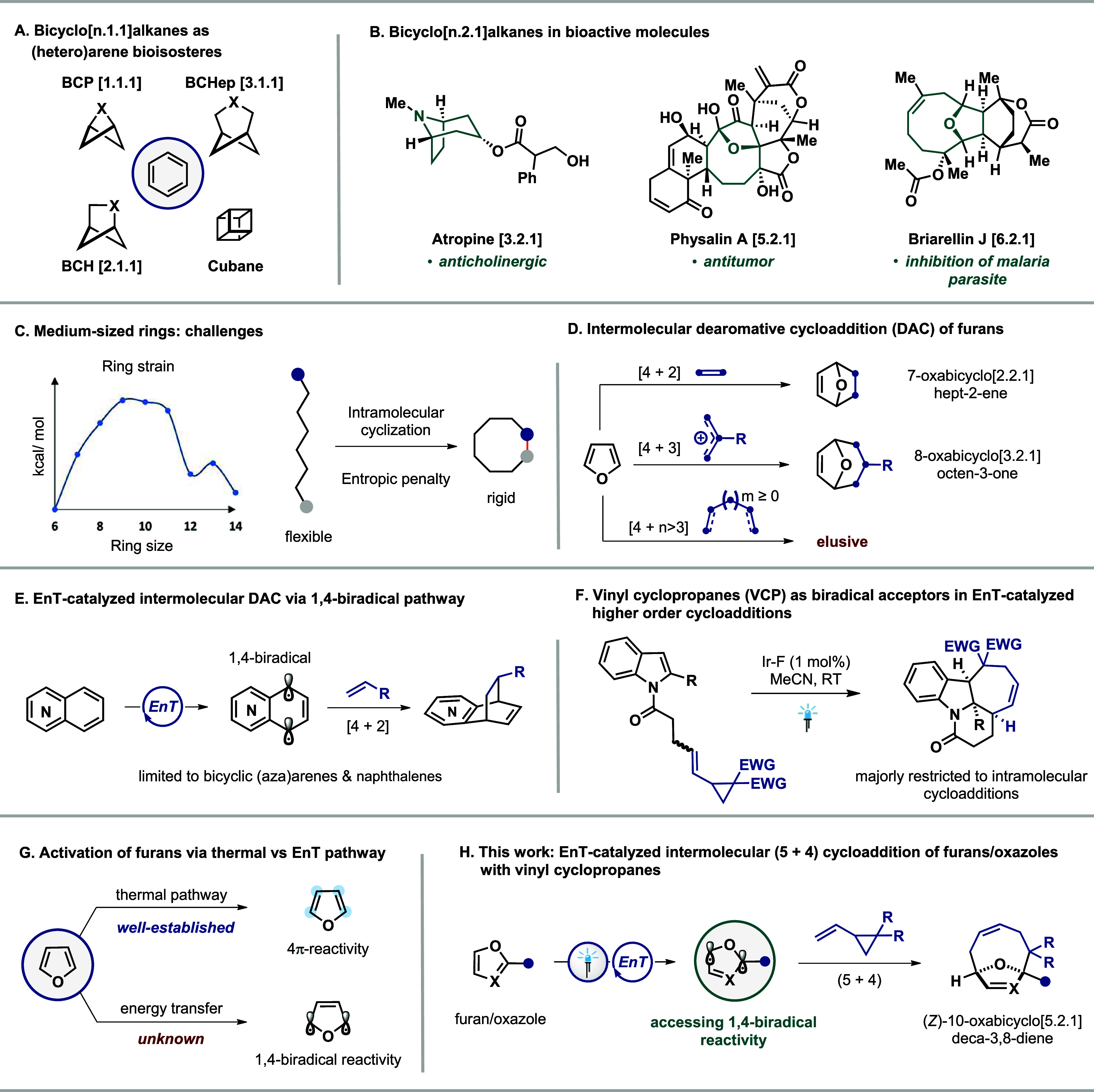
(A) Bicyclo­[n.1.1] alkanes as (hetero)­arene (bio)­isosteres. (B) Bicyclo­[n.2.1]­alkanes in natural products. (C) Synthetic challenges for constructing medium sized rings. (D) Different modes of intermolecular furan cycloaddition. (E) EnT-catalyzed intermolecular cycloaddition via 1,4-biradical pathway. (F) Vinyl cyclopropanes as biradical acceptors in EnT-catalyzed higher order cycloadditions. (G) Thermal vs EnT-catalyzed furan activation. (H) This work.

Dearomative cycloadditions offer an appealing strategy for the rapid assembly of molecular complexity in a single operation.^15^ Retrosynthetically, five-membered heteroarenes such as furans or azoles can serve as ideal 4π components in the construction of bicyclo­[n.2.1]­alkane architectures (Figure 1D). Classical examples include the Diels–Alder cycloaddition of furans with alkenes, which furnishes bicyclo[2.2.1]­heptene scaffolds.^16^ Moving beyond the classical [4 + 2] cycloaddition, intermolecular [4 + 3] cycloadditions of furans with allylic cation precursors have also been reported,^17^ affording 8-oxabicyclo[3.2.1]­octene frameworks. However, to the best of our knowledge, intermolecular higher-order cycloadditions ([4 + *n*]; *n* > 3) of furans, potentially providing access to larger oxabicyclo­[n.2.1]­alkanes, remain unprecedented, as their thermal 4π reactivity has been confined to [4 + 2]/[4 + 3] cycloaddition processes. Energy transfer photocatalysis has emerged as a fundamentally distinct approach for accessing reactivity manifolds that are inaccessible under thermal conditions through triplet-state activation.^18–20^ We therefore questioned whether triplet sensitization could unlock a diene-type 1,4-biradical manifold in furans. If achievable, productive interception of such triplet intermediates by suitable biradical acceptors could open up new avenues toward higher-order intermolecular cycloadditions (Figure 1G).

Although EnT-catalyzed intermolecular dearomative cycloadditions have seen remarkable progress over the past decade,^19,21,22^ accessing triplet-state reactivity in monocyclic heteroarenes such as furans has remained largely unexplored, primarily due to their high triplet energy barrier. Moreover, in contrast to the more common [2 + 2] activation mode,^23^ EnT-catalyzed intermolecular cycloadditions proceeding through a diene-type 1,4-biradical pathway have, to date, been confined to bicyclic arenes, such as quinoline and naphthalene derivatives undergoing intermolecular [4 + 2] cycloadditions with alkenes (Figure 1E).^24^ We recently demonstrated that productive intermolecular triplet-state reactivity in monocyclic heteroarenes can also be achieved by tuning their excited-state properties through a data-driven screening strategy, thereby establishing a general platform for harnessing monocyclic heteroarenes in EnT catalysis.^25^ In addition to accessing triplet-state reactivity in furans, the realization of higher-order cycloadditions also requires a suitable biradical acceptor. In this context, You and co-workers established vinyl cyclopropanes (VCPs) as five-carbon biradical acceptors^26^ in EnT-catalyzed higher-order cycloadditions (Figure 1F). They demonstrated intramolecular (5 + 2) and (5 + 4) dearomative cycloadditions of indole, pyrrole, and naphthalene derivatives with VCPs.^21,27^ While these studies elegantly demonstrate the feasibility of higher-order cycloadditions under EnT catalysis, they are predominantly limited to intramolecular processes and rely on prefunctionalized, heteroarene-tethered VCP substrates. In contrast, an intermolecular variant, although more challenging, would offer a modular approach to assemble medium-sized bridged heterocycles from readily available components. We therefore hypothesized that if furans could exhibit a 1,4-biradical character under EnT catalysis, interception of this intermediate by VCPs could enable intermolecular higher-order cycloadditions, providing direct access to larger bicyclo­[n.2.1]­alkane frameworks. Building on this conceptual foundation, herein we report the EnT-catalyzed intermolecular (5 + 4) dearomative cycloaddition of furans/oxazoles with vinyl cyclopropanes, enabling the direct and efficient synthesis of the (*Z*)-10-oxabicyclo­[5.2.1]­deca-3,8-diene scaffold (Figure 1H). The partially unsaturated nature of this framework provides multiple synthetic handles for the further generation of molecular complexity. Furthermore, we demonstrate the modular installation of oxabicyclo[5.2.1]­decane scaffolds as versatile building blocks in synthetic chemistry.

## Results and Discussion

### Reaction Development

We commenced our studies by examining the structure–excited-state property relationship across a series of substituted furans using our ML-based screening tool EnTdecker.^28^ Compared to unsubstituted furan, which possesses a relatively high triplet energy (*E*_T_ = 72 kcal/mol), we identified that C2-acyl substitution substantially lowers the triplet energy (**1a**, *E*_T_ = 59.5 kcal/mol) and should render the substrate amenable to energy transfer activation. Guided by this prediction, we subjected furan **1a** and Michael-acceptor-type VCP **2a** to irradiation with blue LEDs in the presence of [Ir­(dF­(CF_3_)­ppy)_2_(dtbbpy)]­PF_6_ (Ir-F, *E*_T_ = 61.8 kcal/mol) as the photocatalyst in CH_2_Cl_2_. Gratifyingly, the desired (5 + 4) cycloadduct was obtained in 29% yield (Figure 2, entry 1). Notably, the (5 + 4) cycloaddition furnished two regioisomers, both of which were readily separated by column chromatography. The major regioisomer resulted from the radical addition from the C5-position of furan to the VCP (**3a**), and the minor regioisomer arises from the radical addition from C2-position (**3a′**).

**Figure 2:**
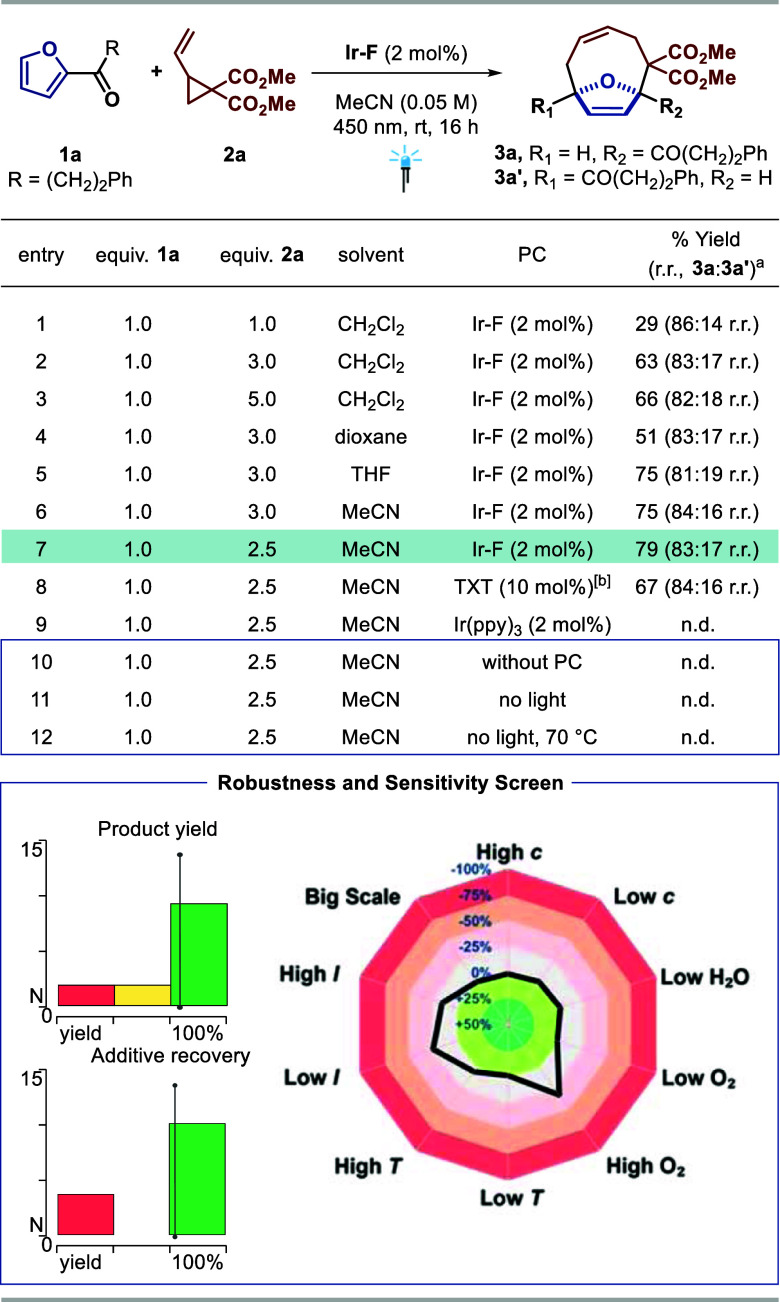
Optimization studies, sensitivity and robustness screening. ^[a]^reactions were carried out on a 0.1 mmol scale and yields were determined by ^1^H NMR analysis using dibromomethane as an internal standard, n.d. = not detected, ^[b]^λ_max_ = 405 nm. Sensitivity and Robustness Screen were conducted as reported by Glorius and co-workers.^30,31^

Encouraged by this initial result, we systematically evaluated reaction parameters to improve the efficiency.^29^ These optimization studies identified MeCN as the optimal solvent, and using an excess of **2a** furnished **3a** in 79% yield with an 83:17 regioisomeric ratio (Figure 2, entry 7). In addition to Ir-F, an organic photosensitizer thioxanthone was also competent and delivered the product in 67% (84:16 r.r.) yield. Control experiments confirmed the photochemical nature of the transformation. Reactions performed in the dark or in the absence of photocatalyst resulted in no product formation, establishing that both visible light and photocatalyst are required (Figure 2, entry 10, 11). Furthermore, to exclude the possibility of a background thermal pathway, the reaction conducted at 70 °C also failed to produce any cycloadduct (Figure 2, entry 12).

A reaction-sensitivity assessment revealed that the transformation is largely insensitive to variations in concentration or temperature (Figure 2).^30^ Importantly, the reaction could be scaled up to 1 mmol without any loss in yield, demonstrating the scalability of the developed protocol. In contrast, high oxygen levels or reduced light intensity significantly diminished the reaction efficiency. As a preliminary evaluation of functional-group tolerance, an additive-based robustness screen was conducted.^31^ The reaction displayed excellent compatibility, tolerating alcohols, ketones, aldehydes, and amides, whereas aniline and pyrrole additive completely suppressed reactivity, likely due to competing oxidative processes (Figure 2).

### Mechanistic Investigation

Having established the optimal reaction conditions, we next sought to obtain in-depth insights into the reaction mechanism. UV-vis absorption measurements indicated that neither **1a** nor **2a** show any absorption at the operational wavelength (λ_max_ = 450 nm), and Ir-F is the only absorbing species in this range (Figure 3A). Stern-Volmer quenching studies further demonstrated that furan **1a** quenches the triplet excited-state of the photocatalyst, whereas VCP **2a** shows no detectable quenching, implying a direct interaction between photocatalyst and **1a** (Figure 3B). To further probe into the nature of the photocatalyst-substrate interaction, we evaluated a panel of photocatalysts spanning a broad range of triplet energies and redox potentials. A clear trend was observed wherein the reaction outcome is strongly influenced by the triplet energy of the employed photocatalyst (Figure 3C). However, no correlation was observed with respect to the redox potentials. Considering the triplet energy of furan **1a** (59.5 kcal/mol), only photocatalysts with triplet energies >60 kcal/mol were competent in delivering the product.^19,20^

**Figure 3:**
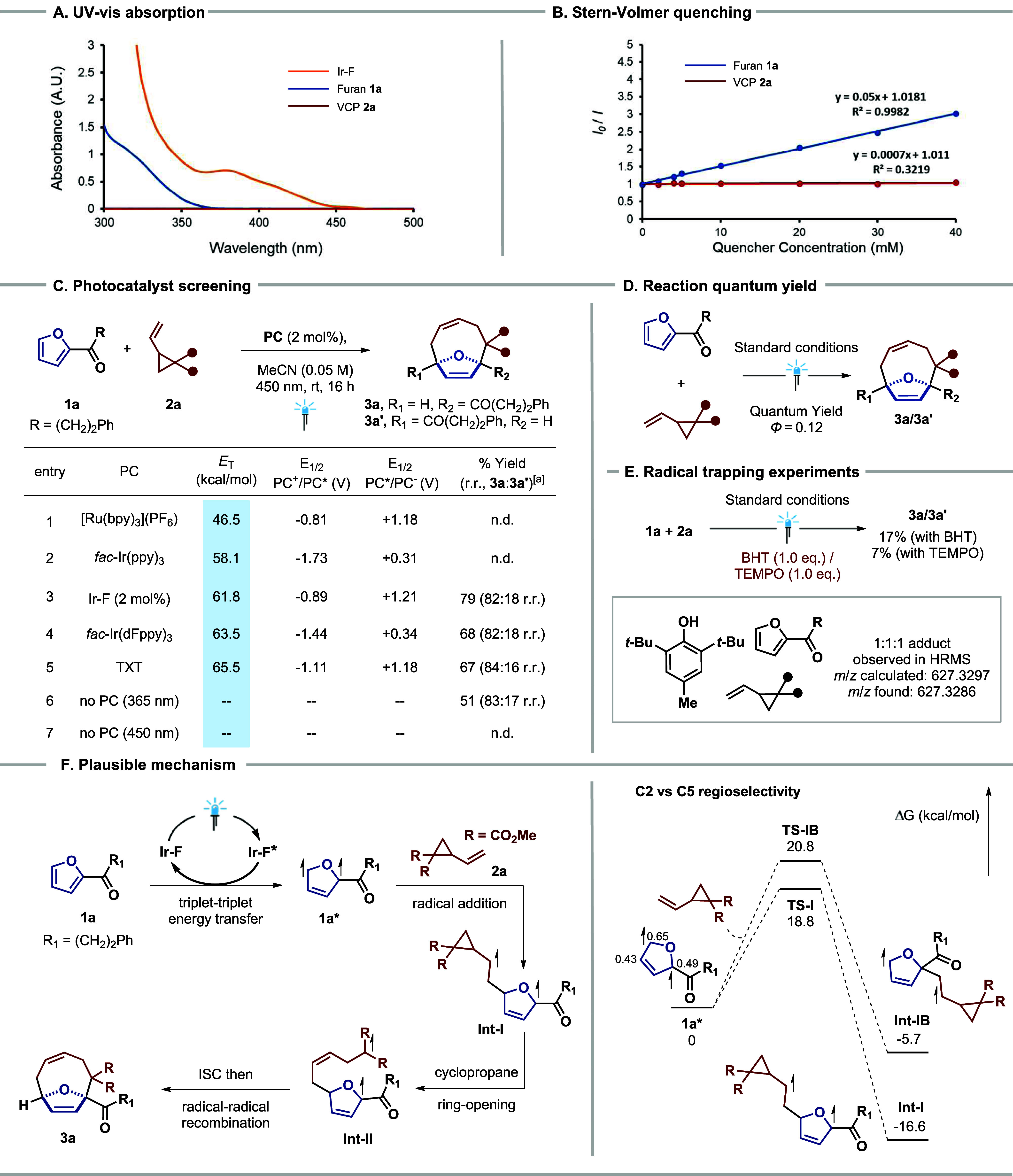
Mechanistic studies. (A) UV-vis absorption studies. (B) Stern-Volmer luminescence quenching studies. (C) Evaluation of different photocatalysts. (D) Quantum yield measurement. (E) Radical trapping experiments. (F) Proposed reaction mechanism. DFT calculations were carried out at the B3LYP­(D3)/def2-SVP//ωB97X-D3/def2-TZVPP level of theory. Labels in 1a* denote spin density from Mulliken population analysis (Supporting Information Section 4.1). PC, photocatalyst; TXT, thioxanthone; ISC, intersystem crossing.

Consistent with these findings, cyclic voltammetry studies revealed that neither of the reaction components **1a** or **2a** show any redox activity within the electrochemical window of the employed photocatalyst (Ir-F), thereby ruling out the possibility of a photoredox pathway (see Supporting Information Section 3.3.1). Notably, direct excitation at 365 nm also afforded the cycloadduct, albeit in reduced yield (Figure 3C, entry 6). However, under the standard reaction conditions (450 nm irradiation), direct excitation is not operative, as no product formation is observed in the absence of photocatalyst (Figure 3C, entry 7). This outcome aligns with the UV-vis spectrum of **1a**, which shows no absorption at 450 nm and only begins to absorb at ∼360 nm. These results indicate that, while direct excitation is possible at shorter wavelengths, it is not mechanistically relevant under the standard sensitized conditions. Quantum yield measurements revealed Φ = 0.1, effectively excluding a radical chain mechanism. To confirm the presence of radical species, the reaction was carried out in the presence of BHT and TEMPO as radical scavengers. In both cases the reactivity was suppressed and trapped radical intermediates could be detected in the presence of BHT by high-resolution mass spectrometry (Figure 3D,E).

Based on these mechanistic observations, we proposed that under visible light irradiation, the excited-state photocatalyst (Ir-F*) undergoes a triplet-triplet energy transfer with **1a** to generate its triplet excited state 1a* (Figure 3F). Experimentally, two regioisomeric (5 + 4) cycloadducts were observed (**3a** as the major isomer and **3a′** as the minor isomer), indicating that radical addition from 1a* to vinyl cyclopropane **2a** can occur from either the C2 or C5 position of 1a*. Spin density analysis of 1a* revealed that the C5 position has the highest spin density followed by the C2 position, suggesting C5 to be the preferential site for radical addition. In addition, the corresponding barriers were evaluated computationally, revealing that the radical addition from the C5 position to generate triplet diradical intermediate **Int-I** is both kinetically and thermodynamically more favorable compared to the C2-position to generate **Int-IB** (Figure 3F). These energetic differences account for the observed regioselectivity of the cycloaddition. From **Int-I**, the cyclopropane ring can undergo fragmentation to form **Int-II**. Subsequently, **Int-II** undergoes intersystem crossing followed by radical-radical recombination to afford the (5 + 4) cycloadduct **3a**.

### Reaction Scope

With a thorough mechanistic understanding and the optimized reaction conditions in hand, we next examined the generality of the developed dearomative cycloaddition protocol. We began by evaluating the substrate scope of furans **1** using VCP **2a** as the biradical acceptor (Figure 4). A broad range of functional groups on the C2-acyl substituent were well tolerated under the established conditions: alkyl groups (**3a**, **3b**), nitrile (**3c**), alcohol (**3d**) and protected amino acids (**3h**) all furnished the desired cycloadducts in good yields (52–77%, up to >95:5 r.r.). *N*-Acyl pyrazole and benzoyl-substituted derivatives afforded the corresponding products (**3e**, **3f**) in slightly reduced yields, yet with excellent regioselectivity. Interestingly, in the presence of a second furan moiety, chemoselective functionalization occurred exclusively at the 2-acyl substituted furan ring (**3g**). Evaluating alternative functional groups at the C2-position beyond the acyl group led us to discover that nitrile (**3i**) and ester (**3j**') substituted furans could be transformed to the desired products in good yields (51% and 48% respectively). Notably, these substrates were not amenable to the standard photocatalyst Ir-F due to their intrinsically higher triplet energies. Instead, the organic photosensitizer 3-OMe-TXT (*E*_T_ = 67.6 kcal/mol)^32^ was successfully employed (see Supporting Information, Section 2.5.3 exploring substitution-triplet energy-reactivity correlation). The protocol was further extended to additional substituents capable of lowering the triplet energy, including trifluoroacetate (**3k**), aryl (**3l**) and heteroaryl (**3m**) groups, thereby underscoring the broader applicability of the developed transformation. In contrast, furans bearing methoxy (**1ax**), Bpin (**1ay**) or alkyl (**1az**) substituents (*E*_T_ > 68 kcal/mol) were unreactive under the applied conditions, as their triplet energies preclude effective sensitization (see Supporting Information, Section 2.6 substrate limitations). Other disubstituted furans with methyl groups at the C5 (**3n**) or C3 (**3o**) positions participated smoothly, providing the corresponding cycloadducts in good yields. Remarkably, disubstituted furans bearing bromo (**3p**) or ester (**3q**) groups at the C4 position also proved compatible, delivering the desired products in 45% and 47% yield, respectively. While the preferential formation of **3a** over **3a′** was rationalized by computing the kinetic barriers, we sought to gain further insight into the observed regioselectivity across the furan substrate scope by correlating the difference in spin density at the C5 and C2 positions with the corresponding experimental regioisomeric excess (see Supporting Information, Section 4.2). A general trend was observed: substrates exhibiting a larger spin density difference show higher regioselectivity (e.g., **1c** and **1f**), whereas those with smaller differences display diminished selectivity (e.g., **1i** and **1j**). This correlation provides a qualitative rationale for the observed regioselectivities across the different substituted furans.

**Figure 4:**
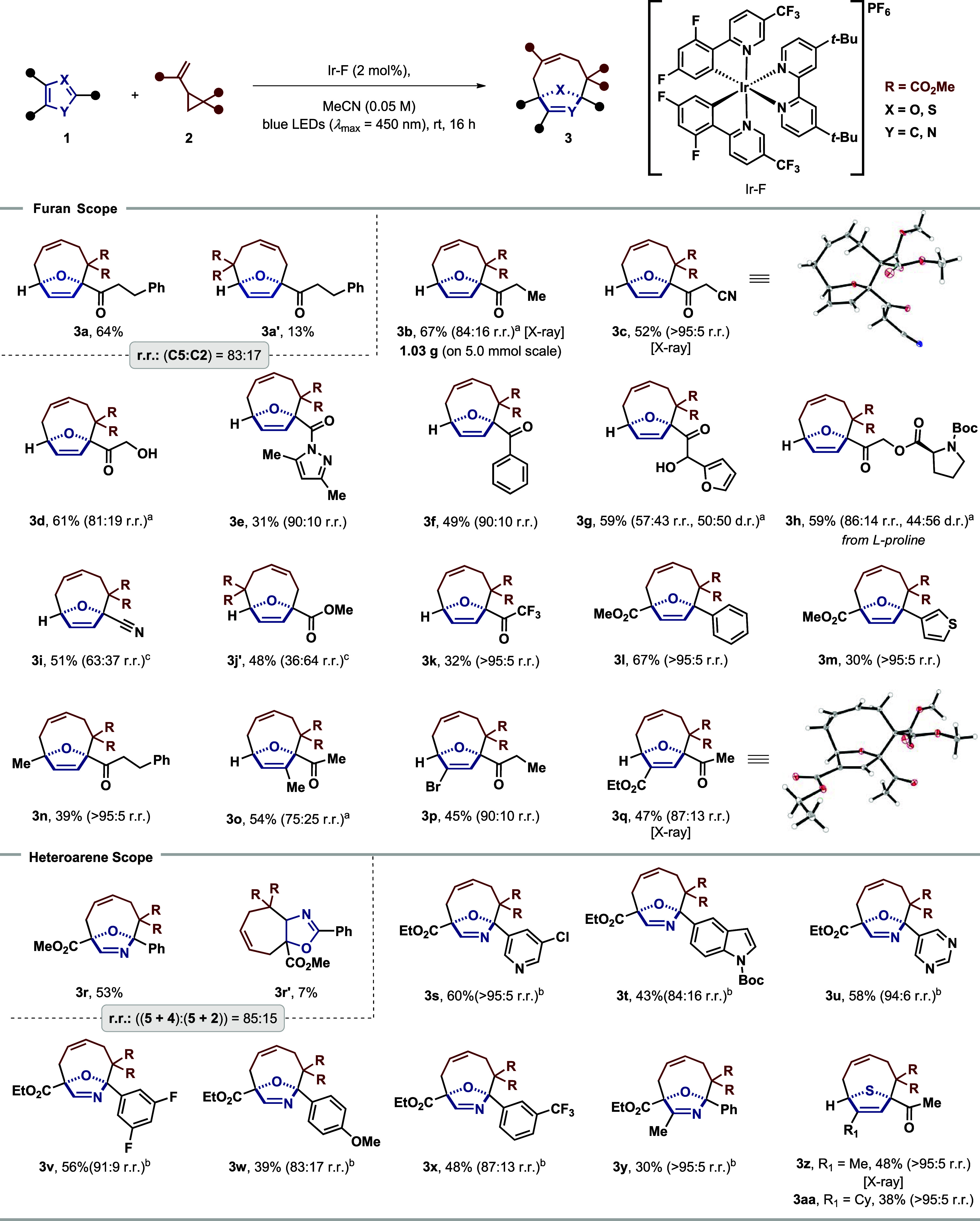
EnT-catalyzed (5 + 4) cycloaddition of furans/oxazoles/thiophenes with vinyl cyclopropanes. Standard reaction conditions: **1** (0.2 mmol), **2** (0.5 mmol), Ir-F (2 mol%), MeCN (2 mL), blue LEDs (λ_max_ = 450 nm), rt, 16 h, isolated yields are given. Crude yields given in brackets. n.d. = not detected. Regioisomeric ratios (r.r.) of the crude reaction mixtures, determined by ^1^H NMR analysis using dibromomethane as an internal standard, are given. Diastereomeric ratios (d.r.) were determined by ^1^H NMR or ^13^C NMR. ^[a]^Isolated combined yield of both regioisomers (**C5** and **C2**) is given, ^[b]^with **2** (0.4 mmol) and MeCN (4 mL), isolated yield of the (5 + 4) product is given, ^[c]^conducted with 3-OMe-TXT (10 mol%) instead of Ir-F.

Guided by our initial hypothesis, we next evaluated whether the triplet-state mediated 4π-reactivity could be extended beyond furans to other monocyclic heteroarenes. Pleasingly, oxazoles and thiophenes participated successfully in the transformation, affording the corresponding nitrogen and sulfur containing analogues of the oxabicyclo[5.2.1]­deca-3,8-diene scaffold. This highlights the conceptual versatility of the method, enabling formal “single-atom swaps” to access new analogues of medium-sized bridged heterocycles.^33^ Under the optimized conditions, oxazole **1r** underwent intermolecular (5 + 4) cycloaddition with VCP **2a**, affording the 10-oxa-8-azabicyclo[5.2.1]­deca-3,8-diene product **3r**. A minor (5 + 2) cycloadduct (**3r'**) was also detected, likely arising from a competing 2π-reactivity. Variation of the aryl substituent at the C2 position of the oxazole was well tolerated: substrates bearing pyridine (**3s**), indole (**3t**), and pyrimidine (**3u**) rings, as well as electron-poor (**3v**, **3x**) and electron-rich (**3w**) aromatics, all delivered the desired 10-oxa-8-azabicyclo[5.2.1]­deca-3,8-dienes in good yields. Notably, even a trisubstituted oxazole could be transformed to the corresponding product (**3y**) in moderate yield. Additionally, disubstituted thiophenes bearing a C2-acyl substituent (**3z** and **3aa**) also engaged effectively in the dearomative (5 + 4) cycloaddition, delivering the corresponding products in good yields.

Next, we turned our attention to exploring the scope of the VCP partner (Figure 5). Electron-deficient VCPs bearing ester or cyano substituents (**3ab**, **3ac**, **3ad**, and **3ae**) readily participated in the (5 + 4) cycloaddition, furnishing the corresponding bridged bicyclic scaffolds in good yields (59–79%). Remarkably, the reaction proved equally effective with unactivated VCPs, which delivered the desired cycloadducts (**3ag** **−3aj**) in excellent yields (63–71%). This outcome highlights the broad VCP scope of the developed protocol and, importantly, discards the need of electron-withdrawing groups that are typically required to promote cyclopropane ring opening or stabilize radical intermediates. Notably, the alkynyl functionality present in product **3aj** also offers a valuable synthetic handle for further diversification via click-type transformations.

**Figure 5:**
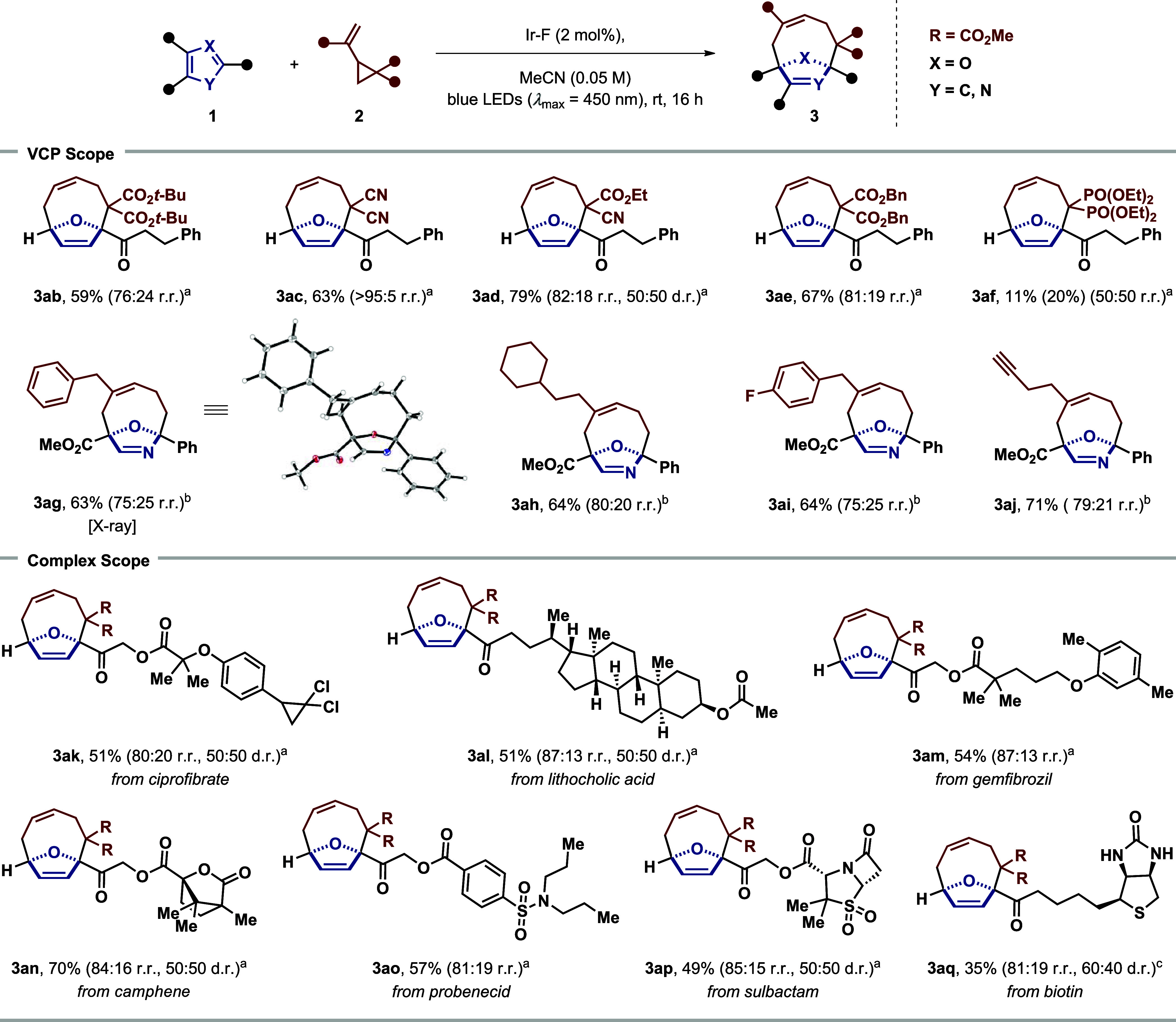
EnT-catalyzed (5 + 4) cycloaddition of furans/oxazoles with vinyl cyclopropanes. Standard reaction conditions: **1** (0.2 mmol), **2** (0.5 mmol), Ir-F (2 mol%), MeCN (2 mL), blue LEDs (λ_max_ = 450 nm), rt, 16 h, isolated yields are given. Crude yields given in brackets. Regioisomeric ratios (r.r.) of the crude reaction mixtures, determined by ^1^H NMR analysis using dibromomethane as an internal standard, are given. Diastereomeric ratios (d.r.) were determined by ^1^H NMR or ^13^C NMR. ^[a]^Isolated combined yield of both regioisomers is given, ^[b]^with **2** (0.4 mmol) and MeCN (4 mL), isolated yield of the (5 + 4) product is given, ^[c]^with TXT (10 mol%) instead of Ir-F.

Given the well-established biological significance of the oxabicyclo[5.2.1]­decane scaffold, we next examined the applicability of the developed transformation for late-stage functionalization (Figure 5). Furan derivatives prepared from ciprofibrate (**3ak**), gemfibrozil (**3am**), camphene (**3an**), probenecid (**3ao**) and sulbactam (**3ap**) all furnished the corresponding (5 + 4) cycloadducts in good yields (49–70%), demonstrating excellent functional-group tolerance and highlighting the potential of this protocol for late-stage functionalization. Furthermore, steroid- and (+)-biotin-derived furans also delivered the desired products (**3al** and **3aq**, 51% and 35% respectively). In the latter case, TXT (*E*_ox_ = 1.18 V) was employed owing to the highly oxidizable nature of the biotin core and the resulting product was isolated as a mixture of regioisomers due to its polar nature.

### Synthetic Applications

The bridged heterocyclic motifs accessed through the intermolecular (5 + 4) cycloaddition provide multiple synthetic handles for downstream functionalizations. We first targeted the complete saturation of the bicyclic framework to access the 10-oxabicyclo[5.2.1]­decane scaffold, a motif widely represented in bioactive molecules.^3^ Hydrogenation of cycloadduct **3b** under 1 bar of H_2_ furnished the fully saturated core (**4a**) in quantitative yield (Figure 6A). The corresponding oxazole-derived cycloadduct (**3r**) could also be partially reduced following a two-step protocol: selective olefin hydrogenation followed by ester reduction with NaBH_4_, afforded the imine-containing 10-oxa-8-azabicyclo[5.2.1]­dec-8-ene scaffold (**4k**) in 49% yield over two steps. The inherent olefinic sites in the cycloadduct also offer a platform for building molecular complexity (Figure 6B). Selective epoxidation of the alkene derived from the VCP fragment proceeded smoothly, providing an embedded epoxide (**4d**) in 86% yield. Surprisingly, treatment with BCl_3_ triggered a cascade-type rearrangement, generating a trifused ring system containing three contiguous quaternary stereocenters (**4c**), presumably through Lewis Acid activation of the ketone as well as hydrolysis of one of the ester groups, followed by lactone formation. Further when subjecting 4-aryl substituted thiophene **1ar** to our standard reaction conditions, a fused bicyclic product (**4e**) was obtained, forming via a formal [3,3] sigmatropic rearrangement (Figure 6C).

**Figure 6:**
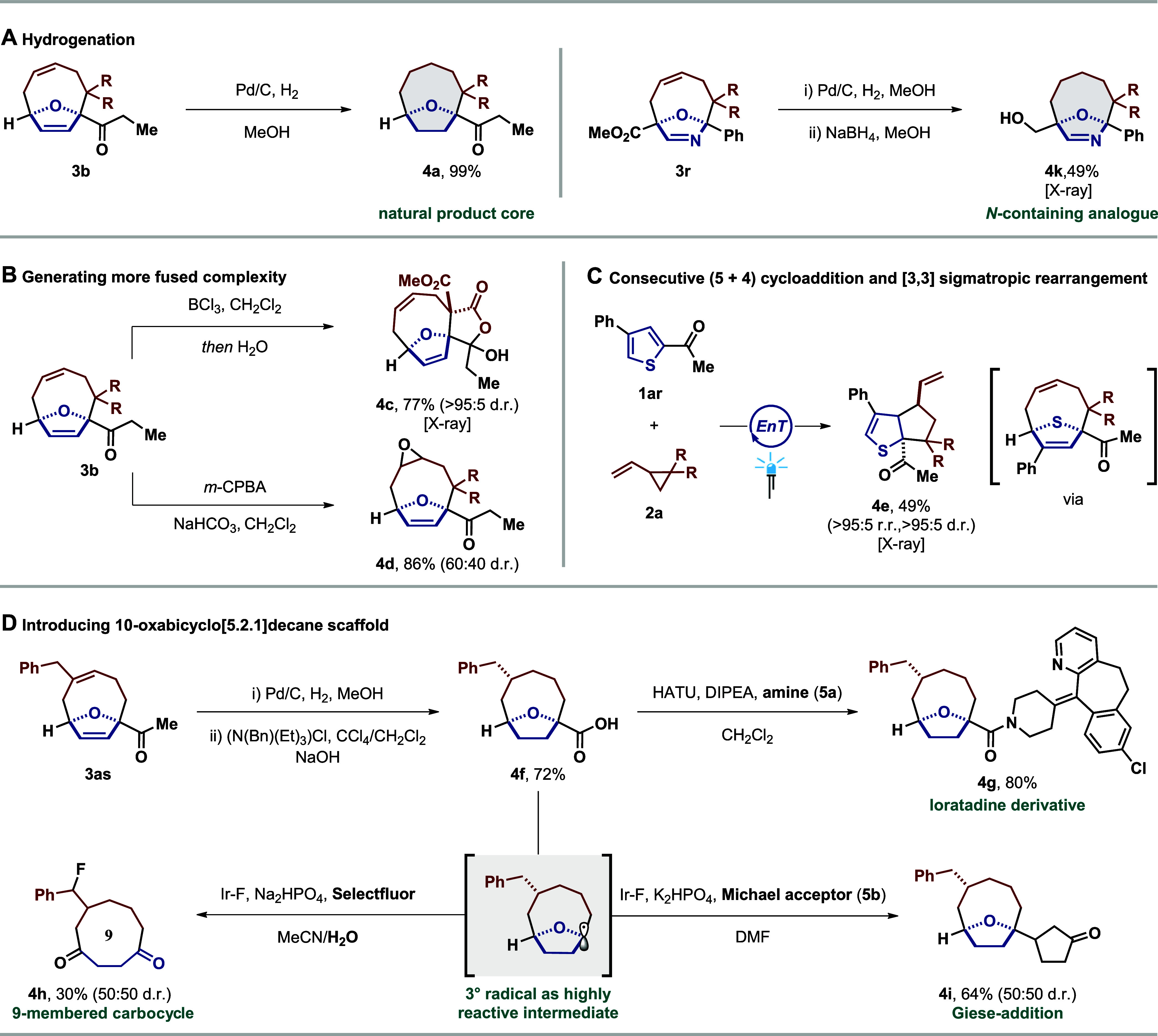
Synthetic applications. **3as** was obtained under standard reaction conditions on 3.0 mmol scale: **1** (3.0 mmol), **2** (6.0 mmol), Ir-F (1.3 mol%), MeCN (30 mL), blue LEDs (λ_max_ = 450 nm), rt, 16 h. Diastereomeric ratios (d.r.) were determined by ^1^H NMR or ^13^C NMR. All final postfunctionalization reactions were carried out on 0.1 mmol scale. For detailed experimental procedures see Supporting Information Section 2.4. **4e** was obtained under standard reaction conditions.

Given the limited availability of medium-sized bridged heterocycles, we next sought to establish a general strategy that enables these scaffolds to function as versatile building blocks (Figure 6D).^13,14^ The acetyl substituent present in most of the cycloadducts proved to be a highly versatile functional handle in this regard. Hydrogenation followed by haloform oxidation of the methyl ketone (**3as**) afforded the corresponding carboxylic acid (**4f**) in 72% yield. A simple amide coupling subsequently installed the bridged bicyclic scaffold onto a desloratadine derivative (**4g**), demonstrating the feasibility of late-stage functionalization. We then envisioned that by utilizing established photoredox mediated decarboxylative functionalization methods, this acid functionality could serve as an entry point for modular diversification through radical mediated processes.^34^ Under photoredox conditions, treatment with Selectfluor surprisingly led to an unexpected cleavage of the C–O bond, resulting in the formation of a 9-membered carbocycle (**4h**). More importantly, a decarboxylative Giese-type addition of the generated tertiary radical to cyclopentenone was achieved, delivering adduct (**4i**) through formation of a new C­(sp^3^)−C­(sp^3^) bond.^35^ Together, these transformations exemplify the potentially broad utility of this medium-sized bridged heterocycle as a versatile building block in synthetic chemistry.

## Conclusion

In summary, we have developed an energy transfer-catalyzed intermolecular (5 + 4) dearomative cycloaddition that enables direct access to medium-sized bridged heterocycles from simple substituted furans and VCPs. Beyond furans, oxazoles and thiophenes also participate in the transformation, providing diverse heterobicyclo[5.2.1]­alkene motifs in a single step. Importantly, the synthetic utility of the resulting scaffolds was demonstrated through downstream functionalizations, establishing these motifs as versatile building blocks for molecular assembly and offering a general approach for incorporating heterobicyclo[5.2.1]­alkane frameworks into synthetic and medicinal chemistry. While the thermal 4π-diene reactivity of five membered heteroarenes such as furans is well established in classical [4 + 2]/[4 + 3] cycloadditions, the EnT activation mode described herein unlocks a distinct diene-type 1,4-biradical reactivity manifold. This triplet-state pathway enables intermolecular higher-order cycloadditions that remain inaccessible under conventional thermal activation modes. More broadly, this strategy establishes a platform for designing intermolecular dearomative transformations by harnessing such excited-state reactivity. We also anticipate that this work will stimulate further development of modular synthetic approaches to medium-sized ring construction, and facilitate the broader incorporation of these underrepresented motifs into medicinal chemistry and drug discovery efforts.

## Supplementary Material



## Notes

Additional experimental details, materials, methods (Section 2.1), mechanistic investigation (Section 3), computational calculations (Section 4) and spectroscopic data for all compounds (Section 6) are provided in the SI (PDF).
