# Supplementary material for: Accessing Medium-Sized Bridged Heterocycles via EnT-Catalyzed Intermolecular Dearomative (5 + 4) Cycloaddition of Furans and Oxazoles
Source: J Am Chem Soc. 2026 Jun 16;148(25):25469–79. doi: 10.1021/jacs.6c00671 (PMC13339174; doi:10.1021/jacs.6c00671)
Supplement: Supplementary file 1 [file ja6c00671_si_001.pdf]

Supplementary Information

Accessing Medium-Sized Bridged Heterocycles via EnT-Catalyzed Intermolecular Dearomative (5+4) Cycloaddition of Furans and Oxazoles

Carla Hümpel[1,2], Debanjan Rana[1,2], Sophie Korgitzsch[1], Kiana Fischer[1], Constantin G. Daniliuc[1] and Frank Glorius[1,*]

[1]Organisch-Chemisches Institut, Universität Münster, 48149 Münster, Germany.

[2]These authors contributed equally.

*Correspondence to: glorius@uni-muenster.de

TABLE OF CONTENTS

1. GENERAL EXPERIMENTAL 3
1.1. Glassware, Solvents and Reagents 3
1.1.1. Photochemical set-up and light sources 3
1.2. Chromatography and Data Analysis 3
1.3. Naming of Compounds 4
2. EXPERIMENTAL SECTION 5
2.1. General Procedures 5
2.1.1. General Procedure A: Synthesis of 2-acyl furan derivatives 5
2.1.2. General Procedure B: Synthesis of 2-acyl aryl furan derivatives 5
2.1.3 General Procedure C: Synthesis of 2-acyl complex furan derivatives 6
2.1.4 General Procedure D: Synthesis of oxazoles 6
2.1.5 General Procedure E: Synthesis of unactivated VCPs 7
2.1.6 General Procedure F: Synthesis of (*Z*)-10-oxabicyclo[5.2.1]deca-3,8-dienes 8
2.2 Starting Material Synthesis 9
2.3 Substrate Scope 29
2.4. Product Diversification 65
2.5. Reaction Optimisation 73
2.5.1. Establishing the stoichiometry and the solvent 73
2.5.2. Establishing the photocatalyst 74
2.5.3. Exploring substitution-triplet energy-reactivity correlation 75
2.6. Substrate Limitations 77
2.7. Sensitivity Screen 79
2.8. Additive-based Robustness Screen 80
3. MECHANISTIC INVESTIGATIONS 83
3.1. UV/vis Absorption Spectroscopy 83
3.2. Stern-Volmer Analysis 83
3.3. Cyclic Voltammetry 84
3.3.1. Redox potential measurements 84
3.4. Quantum Yield Calculation 85
3.4.1. Determination of the photon flux 85
3.4.2. Determination of the reaction quantum yield 86
3.5. Trapping Experiments 88
4. COMPUTATIONAL CALCULATIONS 89
4.1. Computational Methods 89
4.2 DFT Calculation determining C5/C2 regioselectivity 89
4.2.1 Cartesian coordinates 92
5. X-RAY CRYSTALLOGRAPHY 110

5.1. X-Ray Diffraction Data 110

6. SPECTROSCOPIC DATA 129

7. REFERENCES 250

1. GENERAL EXPERIMENTAL

1.1. Glassware, Solvents and Reagents

All reactions were conducted under an inert atmosphere of argon using standard Schlenk techniques unless stated otherwise. All glassware and Teflon-coated magnetic stir bars were dried in an oven at 80 °C prior to use. All anhydrous solvents were commercially supplied and stored over 3 Å or 4 Å mol. sieves or dried using an activated alumina column drying system (MeCN, CH_2Cl_2, hexane, toluene, THF, Et_2O, DMF, MeOH). Reagents were purchased from commercial sources and used as received. Reaction temperatures are reported as the temperatures of the heating/cooling media, unless otherwise stated. Photocatalysts $Ir[(dF(CF_3)ppy)_2dtbbpy](PF_6)$[1], *fac*-$[Ir(ppy)_3]$[2], *fac*-$[Ir(dFppy)_3]$[2] and 4CzIPN[3], were prepared following literature procedures. Thioxanthone was purchased from BLD Pharm.

1.1.1. Photochemical set-up and light sources

Photochemical reactions were performed in a Hepatochem EvoluChemTM PhotoRedOx Box Duo device and irradiated with either two EvoluChem™ HCK1012-01-006 LEDs (30 W, λ_{max} = 365 nm), two EvoluChem™ HCK1012-010 LEDs (18 W, λ_{max} = 405 nm) or two EvoluChem™ HCK1012-01-008 (30 W, λ_{max} = 450 nm). To shield from irradiation, the setup was covered with a small cardboard box. The reaction temperature was determined to be between 30 °C and 33 °C using this setup.

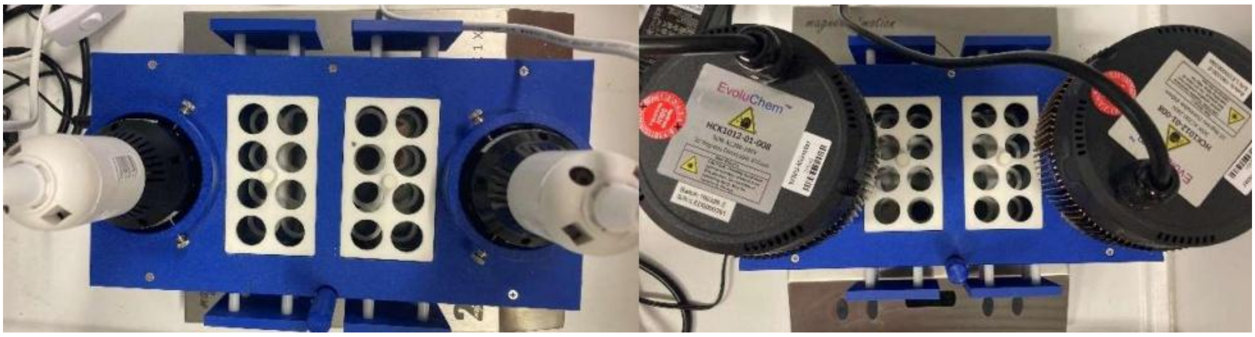

Figure S1: Hepatochem EvoluChemTM PhotoRedOx Box Duo device with two EvoluChem™ (405 nm [left] or 450 nm [right]).

1.2. Chromatography and Data Analysis

Thin layer chromatography (TLC) was performed to monitor reactions when practical using Merck silica gel 60 F_{254} aluminum plates and visualised under UV light, or by staining with aqueous basic potassium permanganate followed by heating. **Flash column chromatography** (FCC) was carried out using Acros Organics silica gel (35–70 mesh) or a Biotage Isolera™ flash purification system. **NMR spectra** were recorded on a Bruker Avance II 400, Agilent DD2 500 or DD2 600 spectrometers. All spectral data was acquired at 298 K. Deuterated solvents were purchased from Eurisotop ($CDCl_3$, deuteration > 99.8%). Chemical shifts (δ) are reported in parts per million (ppm) and referenced to $CDCl_3$ (^{1}H: 7.26 ppm; ^{13}C: 77.16 ppm). Coupling constants

(*J*) are given in Hertz (Hz) and refer to corresponding multiplicities (s = singlet, d = doublet, t = triplet, q = quartet, quin = quintet, hex = hextet, h = heptet, m = multiplet, app = apparent, br. = broad signal, dd = doublet of doublets, etc.). The ^{1}H NMR spectra are reported as follows: chemical shift (multiplicity, coupling constants, number of protons). NMR assignments were made according to spin systems, using two-dimensional NMR spectroscopy (COSY, HSQC, HMBC) to assist the characterisation. NMR yields were determined by ^{1}H NMR analysis using dibromomethane as an internal standard. The *d.r..* and *r.r..* values were determined by ^{1}H NMR analysis of the crude reaction mixture. When only a single regioisomer was detected, no *r.r..* is given. >95:5 *d.r.* indicates when only a single diastereomer could be detected. **High resolution mass spectra (HRMS)** were recorded using electrospray ionisation (ESI) on a Bruker Daltonics, MicroToF spectrometer and calibrated using formate ion clusters or using electron ionization (EI) on an Thermo Fisher Scientific Exactive GC Orbitrap.

1.3. Naming of Compounds

Compound names are those generated by ChemDraw Professional 23.1.2 software (PerkinElmer), following the IUPAC nomenclature.

2. EXPERIMENTAL SECTION

2.1. General Procedures

2.1.1. General Procedure A: Synthesis of 2-acyl furan derivatives

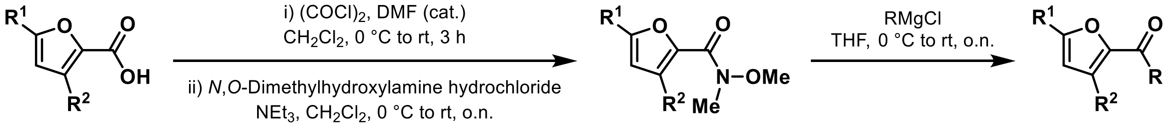

In the first step of the synthesis, the carboxylic acid (1.0 eq.) was dissolved in CH_2Cl_2 (0.2 M) in a Schlenk tube under argon. Then a few drops of DMF (cat.) were added and the solution was cooled to 0 °C. Oxalyl chloride (2.0 eq.) was added dropwise and the reaction mixture was stirred for 2 h at 0 °C. After warming up to room temperature, the reaction mixture was stirred for an additional hour. The solution was then concentrated and the crude acyl chloride was directly used in the second step.

In a Schlenk tube under argon *N*,*O*-dimethylhydroxylamine hydrochloride (1.0 eq.) was dissolved in CH_2Cl_2 (0.2 M) and the solution was cooled to 0 °C. Triethylamine (2.0 eq.) was then added dropwise and the solution was stirred for 15 minutes. The crude acyl chloride (1.0 eq.) was dissolved in CH_2Cl_2 (1.0 M) and slowly added to the solution at 0 °C, after which the reaction mixture was warmed up to room temperature and stirred overnight. Then the reaction was quenched with a sat. aq. $NaHCO_3$ solution and the phases were separated. The aqueous layer was extracted with CH_2Cl_2 (3 x 10 mL) and the combined organic layers dried over Na_2SO_4, filtered and concentrated. After purification *via* column chromatography the corresponding WEINREB amide compound was obtained.

2-Acetyl furans were prepared from the corresponding WEINREB amides *via* GRIGNARD addition. The WEINREB amide (1.0 eq.) was dissolved in THF (0.3 M) in a Schlenk tube under argon. The solution was cooled to 0 °C and alkylmagnesium chloride solution (2.0 eq.) was added dropwise. The reaction mixture was stirred overnight at rt. Then the reaction was quenched with sat. aq. NH_4Cl solution and the phases were separated. The aqueous layer was extracted with CH_2Cl_2 (3 x 10 mL) and the combined organic layers dried over Na_2SO_4, filtered and concentrated. After purification *via* column chromatography the title compound was obtained.

2.1.2. General Procedure B: Synthesis of 2-acyl aryl furan derivatives

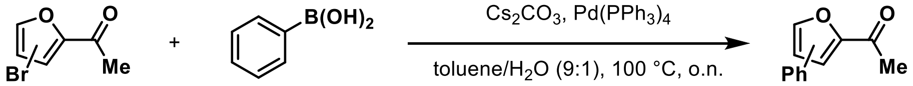

Following a modified literature procedure,[4] a dried Schlenk tube was charged with 2-acetylbromofuran (1.0 eq.), phenylboronic acid (2.0 eq.), caesium carbonate (2.0 eq.) and tetrakis(triphenylphosphine)palladium(0) (5 mol%) under argon. The toluene/H_2O (9:1, 0.4 M) solvent mixture was added and the solution was sparged with argon for 10 min. The reaction mixture was heated to 100 °C and stirred overnight. Then the reaction mixture was filtered through silica gel, concentrated and redissolved in EtOAc. The solution was washed with H_2O (3 x) and the phases were separated. The organic layer was dried over Na_2SO_4, filtered and concentrated. After purification *via* column chromatography on silica gel (*n*-pentane/EtOAc) the title compound was obtained.

2.1.3 General Procedure C: Synthesis of 2-acyl complex furan derivatives

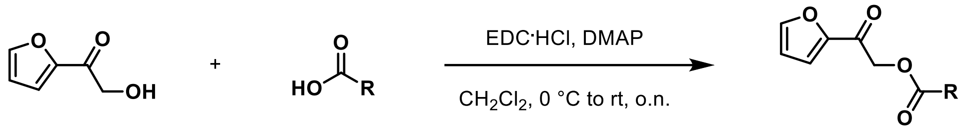

1-(Furan-2-yl)-2-hydroxyethan-1-one (1.0 eq.), the carboxylic acid (1.5 eq.) and 4-dimethylaminopyridine (20 mol%) were dissolved in CH_2Cl_2 (0.1 M) in a Schlenk tube under argon atmosphere. The solution was cooled down to 0 °C and 1-ethyl-3-(3-dimethylaminopropyl)carbodiimide hydrochloride (2.0 eq.) was added in one portion. The reaction mixture was stirred for 30 min at 0 °C and then at rt overnight. Sat. aq. NH_4Cl solution was added and the aqueous layer extracted with CH_2Cl_2 (3 x). The combined organic layers were dried over Na_2SO_4, filtered and concentrated and the ester was obtained after purification *via* column chromatography on silica gel (*n*-pentane/EtOAc).

2.1.4 General Procedure D: Synthesis of oxazoles

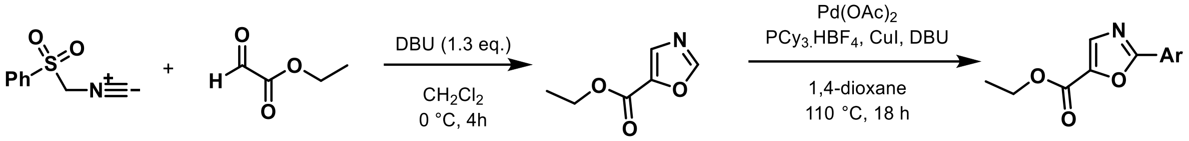

The oxazole derivatives were synthesized following reported literature procedures. In the first step,[5] tosylmethylisocyanide (7.81 g, 40 mmol, 1.0 eq.) was dissolved in CH_2Cl_2 (40 mL) and cooled to 0 °C under argon atmosphere. A separate solution of ethyl glyoxalate (13.1 mL of 50% in Toluene, 64 mmol, 1.6 eq.) and DBU (7.78 mL, 52 mmol, 1.3 eq.) was prepared in CH_2Cl_2 (40 mL) and this solution was added dropwise over 1 h to the flask containing tosylmethylisocyanide solution at 0 °C. After the addition the reaction was stirred further for 3 hours at 0 °C. After completion, the mixture was quenched with 2.0 M HCl (130 mL) and extracted with CH_2Cl_2 and the solvent was removed *in vacuo*. Purification by column chromatography afforded ethyl oxazole-5-carboxylate (3.95 g, 28 mmol, 70%).

In the second step,[6] an oven-dried Schlenk tube equipped with a stir bar was charged with $Pd(OAc)_2$ (5 mol%), $PCy_3{\cdot}HBF_4$ (7 mol%), and CuI (10 mol%). The Schlenk tube was then evacuated and backfilled with argon three times. After this, 1,4-dioxane (0.3 M), DBU (2.0 eq.), aryl bromide (1.0 eq.) and the oxazole-5-carboxylate (1.1 eq.) was added under argon flow. The Schlenk tube was then sealed and heated at 110 °C for 24 hr. After completion, the reaction was quenched with AcOH (5 eq.) and the mixture was filtered through celite with EtOAc. The filtrate was washed with water and the combined organic layers were then dried over anhydrous Na_2SO_4 and concentrated. After purification *via* column chromatography the corresponding oxazole derivatives were obtained.

2.1.5 General Procedure E: Synthesis of unactivated VCPs

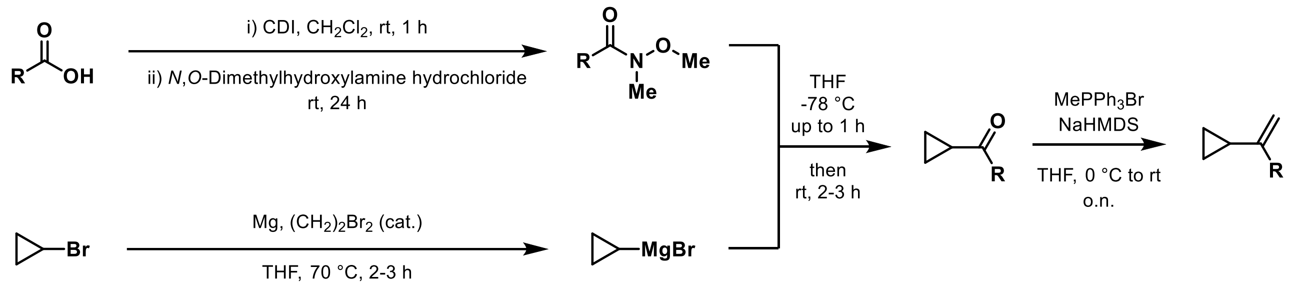

Step 1: Synthesis of WEINREB amides

In an oven-dried Schlenk tube under argon atmosphere, carboxylic acid (20 mmol, 1.0 eq.) and carbonyldiimidazole (4.22 g, 26 mmol, 1.3 eq.) were dissolved in dry CH_2Cl_2 (50 mL, 0.4 M) and stirred at room temperature for one hour. *N*,*O*-Dimethylhydroxylamine hydrochloride (1.70 g, 40 mmol, 2.0 eq.) was added and the reaction stirred for 24 hours. Upon completion, the reaction mixture was quenched with sat. aq. NH_4Cl solution (20 mL) and water (20 mL) and extracted twice with CH_2Cl_2 (2 x 30 mL). The combined organic fractions were washed with brine (30 mL), dried over $MgSO_4$, filtered and concentrated *in vacuo*. Purification by silica gel column chromatography afforded the desired products.

Step 2: Grignard reaction

In an oven-dried Schlenk tube under argon atmosphere, Mg chips (1.4 eq.) were dissolved in dry THF (5.5 M) and treated with a few drops of 1,2-dibromoethane. In a separate oven-dried Schlenk tube under argon, bromocyclopropane (1.4 eq.) was dissolved in dry THF (1.1 M) and the solution was slowly added to the Mg suspension, leading to heat and gas development. The reaction mixture was heated to 70 °C for 2-3 hours, upon which all Mg was consumed, and let cool to room temperature.

In an oven-dried Schlenk tube under argon atmosphere, the WEINREB amide (1.0 eq.) was dissolved in dry THF (1.0 M), cooled to -78 °C and slowly treated with the Grignard solution (1.4 eq.). The reaction mixture was stirred at -78 °C for 30-60 minutes and then at room temperature for 2-3 hours. Upon completion, the reaction mixture was quenched by addition of water and extracted with EtOAc or CH_2Cl_2 (3 x). The organic fraction was dried over $MgSO_4$, filtered and concentrated *in vacuo*. Column chromatography yielded the purified product.

Step 3: Wittig reaction

In an oven-dried Schlenk tube under argon atmosphere, methyltriphenylphosphonium bromide (1.2 eq.) was suspended in dry THF (0.7 M). The suspension was cooled to 0 °C before addition of NaHMDS (1.2 eq., 2.0 M solution in dry THF). The mixture was stirred at that temperature for 30-60 minutes. The respective ketone was dissolved in dry THF (0.6 M) and added to the reaction mixture, let warm to room temperature and stirred overnight. Upon completion, the reaction mixture was quenched by addition of water and sat. aq. NH_4Cl solution. The organic phase was separated, and the aqueous fraction was extracted with EtOAc or CH_2Cl_2 (3 x). The combined organic fraction was dried over $MgSO_4$, filtered and concentrated *in vacuo*. Purification by column chromatography (*n*-pentane) yielded the final VCP.

2.1.6 General Procedure F: Synthesis of (*Z*)-10-oxabicyclo[5.2.1]deca-3,8-dienes

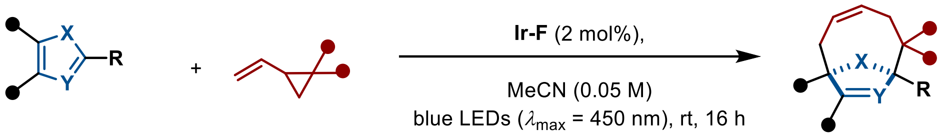

An oven-dried 10 mL Schlenk tube equipped with a Teflon-coated stir bar was charged with furan **1** (0.2 mmol, 1.0 eq., if solid), VCP **2** (0.5 mmol, 2.5 eq., if solid) and Ir-F (4.0 mg, 0.004 mmol, 2 mol%). The Schlenk tube was evacuated and backfilled with argon three times. Subsequently, dry MeCN (2 mL, 0.1 M), furan **1** (0.2 mmol, 1.0 eq., if liquid) and VCP **2** (0.5 mmol, 2.5 eq., if liquid) were added under positive argon pressure. The reaction was then sealed and irradiated with blue LEDs (30 W, λ_{max} = 450 nm) for 16 hours while stirring. Upon completion, the solvent was removed *in vacuo* and ^{1}H NMR was recorded for the crude reaction mixture using CH_2Br_2 (14 µL, 0.2 mmol, 1.0 eq.) as an internal standard. Subsequently, the crude reaction mixture was purified by silica gel column chromatography, to afford the desired products. Reaction with oxazole substrates was conducted with 2.0 eq. of VCP **2** and 4 mL MeCN (0.05 M).

Note: The structures for **3b**, **3b'**, **3c**, **3q**, **3z** and **3ag** were determined by X-ray crystallography. Based on this crystal structure data and high similarity to spectral data all regioisomers were assigned *via* **2D-NMR**. Diastereomeric ratios were determined either *via* integration of ^{1}H-peaks, or if not applicable (stereocenters are too far away and don't couple) *via* integration of diastereomeric ^{13}C-peaks. The structure of one diastereomer is shown. When diastereomers were separable, assignment was conducted *via* 1D-NOESY/1D-TOCSY NMR.

2.2 Starting Material Synthesis

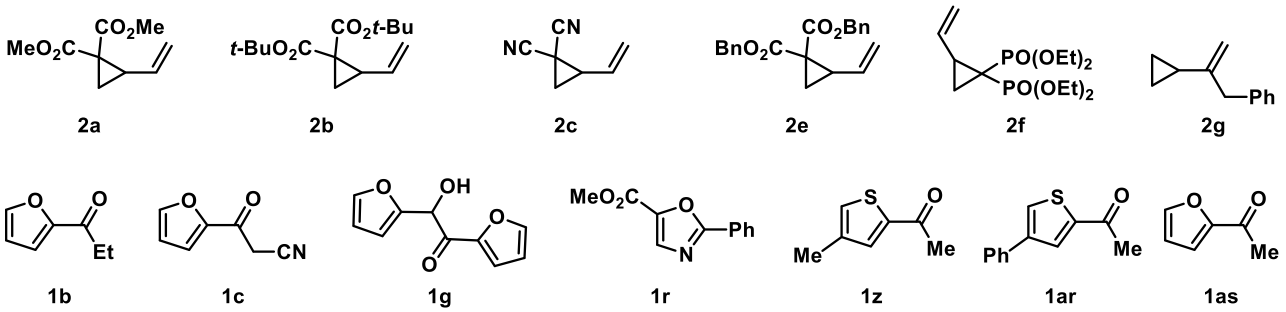

Figure S1: The following compounds **2a - 2f** and **1r**, **1z**, **1ar** were synthesized according to literature procedures.[7] Compound **2g** was previously made in our lab. Compounds **1b**, **1c**, **1g** and **1as** are commercial.

1-(Furan-2-yl)-3-phenylpropan-1-one (1a)

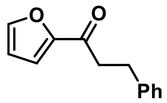

Synthesized following General Procedure A: *N*-Methoxy-*N*-methylfuran-2-carboxamide (775 mg, 1.0 mmol, 1.0 eq.), phenethylmagnesium chloride solution (1 M in THF, 10.0 mL, 10.0 mmol, 2.0 eq.) and THF (15 mL, 0.3 M) were used to obtain **1a** (911 mg, 4.6 mmol, 91%) as a colourless liquid after purification *via* column chromatography on silica gel (*n*-pentane/EtOAc 90:10). Spectral and physical data are in accordance with the literature.[8]

TLC: R_f **=** 0.3 (95:5 *n*-pentane/EtOAc).

NMR Spectroscopy (*see spectra*):

^{1}**H NMR** (400 MHz, $CDCl_3$): δ_H 7.57 (d, J = 1.7 Hz, 1H), 7.31 – 7.16 (m, 6H), 6.52 (dd, J = 3.9, 1.7 Hz, 1H), 3.25 – 3.12 (m, 2H), 3.05 (dd, J = 8.7, 6.6 Hz, 2H) ppm.

1-(Furan-2-yl)-2-hydroxyethan-1-one (1d)

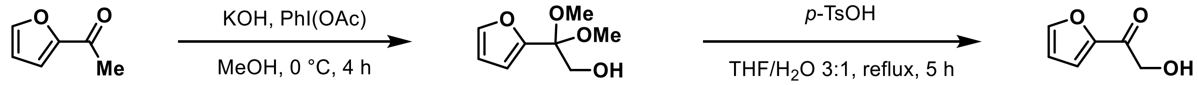

Following a literature procedure,[9] KOH (3.80 g, 67.5 mmol, 5.4 eq.) was dissolved in MeOH (90 mL), and the solution cooled to 0 °C. Next, a solution of 2-acetylfuran (1.65 g, 15.0 mmol, 1.0 eq.) in MeOH (30 mL) was added. Then, (diacetoxyiodo)benzene (7.25 g, 22.5 mmol, 1.5 eq.) was added portion-wise over the course of 15 min and the reaction mixture stirred at 0 °C for 4 h, after which it was quenched with H_2O. MeOH was removed under reduced pressure and the aqueous phase extracted with EtOAc (3 x 30 mL). The combined organic layers were washed with brine, dried (Na_2SO_4) and concentrated to yield the crude acetal, which was directly used in the next step without any further purification.

In the second step, the crude acetal was dissolved in THF/H_2O (30 mL, 3:1) and *p*-toluenesulfonic acid

monohydrate (5.71 g, 30.0 mmol, 2.0 eq.) was added. The reaction mixture was refluxed until full conversion was reached *via* TLC. The reaction was quenched with sat. aq. $NaHCO_3$ solution, and the aqueous layer extracted with EtOAc (3 x). The combined organic layers were then washed with brine, dried and concentrated. Purification *via* column chromatography on silica gel (*n*-pentane/EtOAc 60:40) yielded **1d** (951 mg, 7.5 mmol, 50%) as a slightly yellow solid. Spectral and physical data are in accordance with the literature.[10]

TLC: R_f **=** 0.4 (60:40 *n*-pentane/EtOAc).

NMR Spectroscopy (*see spectra*):

^{1}**H NMR** (400 MHz, $CDCl_3$): δ_H 7.63 (d, *J* = 1.7 Hz, 1H), 7.30 (d, *J* = 3.6 Hz, 1H), 6.63 – 6.57 (m, 1H), 4.74 (s, 2H) ppm.

(3,5-Dimethyl-1*H*-pyrazol-1-yl)(furan-2-yl)methanone (1e)

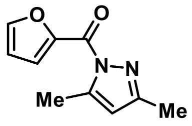

The title compound was prepared according to a literature procedure.[11] A Schlenk tube was charged with 2-fuoric hydrazide (378 mg, 3.0 mmol, 1.0 eq.), acetylacetone (465 µL, 4.5 mmol, 1.5 eq.) and carbon tetrabromide (995 mg, 3.0 mmol, 1.0 eq.) in MeCN (22.5 mL). The reaction mixture was stirred at 60 °C for 1 h, and after cooling down to room temperature quenched with H_2O. The aqueous layer was extracted with EtOAc (3 x), and the combined organic layers dried (Na_2SO_4), filtered and concentrated. After purification *via* column chromatography on silica gel (*n*-pentane/EtOAc 96:4) **1e** (315 mg, 1.7 mmol, 55%) was obtained as a white solid.

TLC: R_f **=** 0.4 (95:5 *n*-pentane/EtOAc).

NMR Spectroscopy (*see spectra*):

^{1}**H NMR** (400 MHz, $CDCl_3$): δ_H 7.93 (dd, *J* = 3.6, 0.8 Hz, 1H), 7.71 (dd, *J* = 1.7, 0.8 Hz, 1H), 6.59 (dd, *J* = 3.6, 1.7 Hz, 1H), 6.02 (s, 1H), 2.62 (s, 3H), 2.29 (s, 3H) ppm;

^{13}**C NMR** (101 MHz, $CDCl_3$): δ_C 156.9, 152.8, 147.6, 146.0, 145.6, 124.1, 112.4, 111.2, 14.6, 14.0 ppm.

Furan-2-yl(phenyl)methanone (1f)

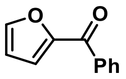

In a Schlenk tube *N*-methoxy-*N*-methyl-2-furancarboxamide (465 mg, 3.0 mmol, 1.0 eq.) was dissolved in dry THF (9.0 mL, 0.3 M) under argon. The solution was cooled to -78 °C and phenyl lithium solution (2 M in THF, 1.50 mL, 3.0 mmol, 1.0 eq.) was added dropwise. The reaction mixture was warmed up to room temperature and stirred overnight. Then the reaction was quenched with sat. aq. NH_4Cl solution and EtOAc. The phases were separated and the aqueous layer was extracted with EtOAc (3 × 10 mL). The combined organic layers

were washed with sat. aq. NaCl solution, dried over Na_2SO_4, filtered and concentrated. After purification *via* column chromatography on silica gel (*n*-pentane/EtOAc 93:7) **1f** was obtained as a lightly yellow liquid (400 mg, 2.3 mmol, 77%). Spectral and physical data are in accordance with the literature data.[12]

TLC: R_f **=** 0.3 (93:7 *n*-pentane/EtOAc).

NMR Spectroscopy (*see spectra*):

^{1}**H NMR** (400 MHz, $CDCl_3$): δ_H 8.01 – 7.94 (m, 2H), 7.71 (dd, *J* = 1.7, 0.8 Hz, 1H), 7.64 – 7.54 (m, 1H), 7.54 – 7.45 (m, 2H), 7.24 (dd, *J* = 3.6, 0.8 Hz, 1H), 6.60 (dd, *J* = 3.5, 1.7 Hz, 1H) ppm.

1-(*tert*-Butyl) 2-(2-(furan-2-yl)-2-oxoethyl) (*S*)-pyrrolidine-1,2-dicarboxylate (1h)

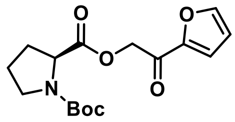

Synthesized following General Procedure C: 1-(Furan-2-yl)-2-hydroxyethan-1-one **1d** (88.2 mg, 0.7 mmol, 1.0 eq.), Boc-*L*-Proline (226 mg, 1.1 mmol, 1.5 eq.), 4-dimethylaminopyridine (17.1 mg, 0.14 mmol, 0.2 eq.), 1-ethyl-3-(3-dimethylaminopropyl)carbodiimide hydrochloride (268 mg, 1.4 mmol, 2.0 eq.) and CH_2Cl_2 (7 mL) were used to afford **1h** (220 mg, 0.7 mmol, 98%) as a slightly yellow liquid.

<u>Note:</u> Some ^{1}H NMR peaks are split into two signals due to rotamers.

TLC: R_f **=** 0.4 (75:25 *n*-pentane/EtOAc).

NMR Spectroscopy (*see spectra*):

^{1}**H NMR** (400 MHz, $CDCl_3$): δ_H 7.63 – 7.58 (m, 1H), 7.28 – 7.23 (m, 1H), 6.61 – 6.53 (m, 1H), 5.40 (d, *J* = 16.6 Hz, 0.5H), 5.28 – 5.14 (m, 1H), 5.05 (d, *J* = 16.6 Hz, 0.5H), 4.50 – 4.43 (m, 0.5H), 4.42 – 4.34 (m, 0.5H), 3.63 – 3.32 (m, 2H), 2.38 – 2.18 (m, 2H), 2.10 – 2.00 (m, 1H), 1.96 – 1.84 (m, 1H), 1.52 – 1.36 (m, 9H) ppm;

^{13}**C NMR** (101 MHz, $CDCl_3$): δ_C 181.8 and 181.4, 172.7 and 172.6, 154.7 and 154.0, 150.6 and 150.6, 147.0 and 146.9, 117.9 and 117.8, 112.7 and 112.6, 80.1 and 80.0, 65.6 and 65.4, 59.1 and 58.8, 46.8 and 46.5, 31.2 and 30.2, 28.6 and 28.4, 24.4 and 23.7 ppm.

HRMS (ESI^+): m/z calc'd for $C_{16}H_{21}NO_6Na$ $[M+Na]^+$: 346.1261, found 346.1254.

2,2,2-Trifluoro-1-(furan-2-yl)ethan-1-one (1k)

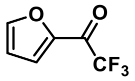

Following a literature procedure,[13] furan (290 µL, 4.0 mmol, 1.0 eq.) was dissolved in THF at 0 °C. *N*-butyllithium solution (1.4 M in hexanes, 3.2 mL, 4.4 mmol, 1.1 eq.) was added dropwise and the reaction mixture stirred for 30 min. The solution was then cooled further to -78 °C and ethyl trifluoroacetate (0.7 mL, 5.8 mmol, 1.45 eq.)

was added dropwise. The reaction was warmed to room temperature and stirred overnight. Afterwards, it was quenched with sat. aq. NH_4Cl solution and the aqueous layer extracted with Et_2O (3 x). The combined organic layers where dried over $MgSO_4$, filtered and concentrated. After purification *via* column chromatography on silica gel (*n*-pentane/EtOAc 97:3) **1k** was obtained as a volatile lightly yellow liquid (336 mg, product:CH_2Cl_2 1:0.97; corrected: 221 mg, 1.4 mmol, 35%). Spectral and physical data are in accordance with the literature data.[13]

TLC: R_f **=** 0.3 (97:3 *n*-pentane/EtOAc).

NMR Spectroscopy (*see spectra*):

^{1}**H NMR** (400 MHz, $CDCl_3$): δ_H 7.82 (d, J = 1.9 Hz, 1H), 7.56 – 7.50 (m, 1H), 6.69 (dd, J = 3.8, 1.7 Hz, 1H).

Methyl 5-phenylfuran-2-carboxylate (1l)

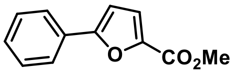

In an oven-dried 10 mL Schlenk tube under Ar, 5-phenylfuran-2-carboxylic acid (188 mg, 1.0 mmol, 1.0 eq.) and K_2CO_3 (155 mg, 1.12 mmol, 1.12 eq.) were dissolved in dry DMF (3 mL) and treated with methyl iodide (125 µL, 2.0 mmol, 2.0 eq.). The reaction mixture was stirred at room temperature overnight. Upon completion, the solvent was evaporated and the crude purified by column chromatography on silica gel (*n*-pentane/EtOAc 95:5) to yield **1l** as a white solid (165 mg, 0.82 mmol, 82%). Spectral data are in accordance with the literature.[14]

TLC: R_f **=** 0.3 (95:5 *n*-pentane/EtOAc).

NMR Spectroscopy (*see spectra*):

^{1}**H NMR** (400 MHz, $CDCl_3$): δ_H δ 7.83 – 7.76 (m, 2H), 7.48 – 7.39 (m, 2H), 7.39 – 7.32 (m, 1H), 7.29 – 7.23 (m, 1H), 6.75 (dt, J = 3.6, 0.6 Hz, 1H), 3.92 (s, 3H) ppm;

Methyl 5-(thiophen-2-yl)furan-2-carboxylate (1m)

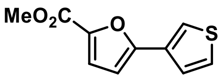

In a 50 mL Schlenk tube, methyl 5-bromofuran-2-carboxylate (410 mg, 2.0 mmol, 1.0 eq.) was dissolved in toluene (8 mL) and thiophen-3-ylboronic acid (307 mg, 2.4 mmol, 1.2 eq.), dissolved in MeOH (0.7 mL), was added. 2M $NaHCO_3$-solution (1.25 mL, 2.5 mmol, 1.2 eq.) and tetrakis(triphenylphosphine)palladium(0) (116 mg, 0.1 mmol, 5 mol%) were added and the reaction mixture sparged with nitrogen for 10 min. The reaction was then refluxed at 80 °C overnight. The reaction mixture was diluted with H_2O after cooling down to room temperature and extracted with EtOAc (3 x). The combined organic layers were washed with brine, dried over Na_2SO_4, filtered and concentrated. After purification *via* column chromatography on silica gel (*n*-pentane/EtOAc 97:3) **1m** was obtained as a white solid (250 mg, 1.2 mmol, 60%). Spectral and physical data are in accordance with the literature.[15]

TLC: R_f **=** 0.3 (97:3 *n*-pentane/EtOAc).

NMR Spectroscopy (*see spectra*):

^{1}H NMR (400 MHz, $CDCl_3$): δ_H 7.71 (dt, *J* = 2.9, 1.5 Hz, 1H), 7.43 – 7.33 (m, 2H), 7.25 – 7.19 (m, 1H), 6.56 (dd, *J* = 4.1, 2.3 Hz, 1H), 3.91 (s, 3H).

1-(5-Methylfuran-2-yl)-3-phenylpropan-1-one (1n)

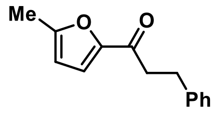

Synthesized following General Procedure A: *N*-Methoxy-*N*,5-dimethylfuran-2-carboxamide (169 mg, 1.0 mmol, 1.0 eq.), phenethylmagnesium chloride solution (1 M in THF, 0.70 mL, 2.0 mmol, 2.0 eq.) and THF (3.40 mL, 0.3 M) were used to obtain **1n** (201 mg, 0.94 mmol, 94%) as a colourless liquid after purification *via* column chromatography on silica gel (*n*-pentane/EtOAc 90:10). Spectral and physical data are in accordance with the literature.[16]

TLC: R_f **=** 0.3 (95:5 *n*-pentane/EtOAc).

NMR Spectroscopy (*see spectra*):

^{1}H NMR (400 MHz, $CDCl_3$): δ_H 7.32 – 7.17 (m, 6H), 7.07 (d, *J* = 3.4 Hz, 1H), 6.16 – 6.10 (m, 1H), 3.14 – 2.99 (m, 4H), 2.38 (m, 3H) ppm.

1-(3-Methylfuran-2-yl)ethan-1-one (1o)

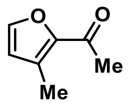

Synthesized following General Procedure A: *N*-Methoxy-*N*,3-dimethylfurane-2-carboxamide (169. mg, 1.0 mmol, 1.0 eq.), methylmagnesium chloride solution (3 M in THF, 0.7 mL, 2.0 mmol, 2.0 eq.) and THF (3.4 mL, 0.3 M) were used to obtain **1o** (111 mg, 0.89 mmol, 89%) as a slightly yellow liquid. Due to complete conversion of the educt, no purification *via* column chromatography was required. Spectral and physical data are in accordance with the literature data.[17]

TLC: R_f **=** 0.4 (95:5 *n*-pentane/EtOAc).

NMR Spectroscopy (*see spectra*):

^{1}H NMR (400 MHz, $CDCl_3$): δ_H 7.39 (d, *J* = 1.7 Hz, 1H), 6.38 (d, *J* = 1.6 Hz, 1H), 2.46 (s, 3H), 2.38 (s, 3H) ppm.

1-(4-Bromofuran-2-yl)propan-1-one (1p)

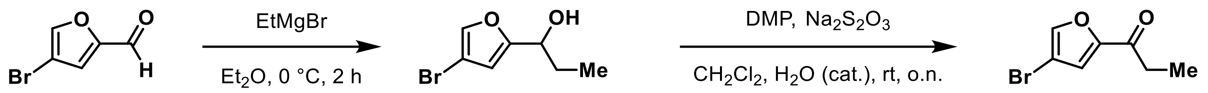

Following a patented procedure,[18] the title compound was synthesized in a two-step procedure. First, in a dried Schlenk flask 4-bromofuran-2-carbaldehyde (438 mg, 2.5 mmol, 1.0 eq.) was dissolved in diethyl ether (8.6 mL, 0.3 M) under argon. At 0 °C ethylmagnesium bromide solution (3 M in Et_2O, 1.67 mL, 5.0 mmol, 2.0 eq.) was added dropwise and the reaction mixture was stirred at 0 °C for 2 h. Afterwards, the reaction mixture was quenched with sat. aq. NH_4Cl solution, ice and CH_2Cl_2 and stirred for 10 min. The phases were separated and the aqueous layer was extracted with CH_2Cl_2 (3 x 15 mL). The combined organic layer was washed with brine, dried over Na_2SO_4, filtered and concentrated under reduced pressure. The residue was directly used in the next step without further purification.

In the second step, a Schlenk flask was charged with the crude 1-(4-bromofuran-2-yl)propan-1-ol (513 mg, 2.5 mmol, 1.0 eq.) and CH_2Cl_2 (8.3 mL, 0.3 M). Dess-Martin periodinane (2.12 g, 5.0 mmol, 2.0 eq.) was added portion-wise as well as one drop of H_2O under argon. The reaction mixture was stirred for 2 h at rt. Then an aq. $Na_2S_2O_3$ solution (1.88 g in 12 mL H_2O,10 Wt%) was added and the reaction mixture was stirred overnight at rt. The solution was filtered and the filtrate was extracted with CH_2Cl_2 (3 x 15 mL), washed with sat. aq. $NaHCO_3$ solution and dried over $MgSO_4$. Then the solution was filtered, concentrated and a diethyl ether/*n*-hexane solution (8 mL, 1:2) was added to the residue. After filtration the residue was concentrated and **1p** was obtained as a brown solid (221 mg, 1.1 mmol, 44%).

TLC: R_f **=** 0.5 (95:5 *n*-pentane/EtOAc).

NMR Spectroscopy (*see spectra*):

^{1}H NMR (400 MHz, $CDCl_3$): δ_H 7.55 (d, J = 0.9 Hz, 1H), 7.16 (d, J = 0.8 Hz, 1H), 2.82 (q, J = 7.4 Hz, 2H), 1.19 (t, J = 7.3 Hz, 3H) ppm.

HRMS (ESI^+): m/z calc'd for $C_7H_8BrO_2$ $[M+H]^+$: 202.9702 and 204.9682, found 202.9703 and 204.9682.

Ethyl 5-acetylfuran-3-carboxylate (1q)

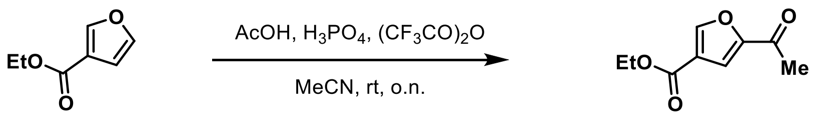

Following a modified literature procedure,[19] a dried Schlenk tube was charged with ethyl-furan-3-carboxylate (409 µL, 3.0 mmol, 1.0 eq.) in MeCN (4.5 mL) under argon. Glacial acetic acid (206 µL, 3.6 mmol, 1.2 eq.), $(CF_3CO)_2O$ (1.66 mL, 12 mmol, 4.0 eq.) and a few drops of H_3PO_4 were added to the solution. The reaction mixture was stirred overnight at room temperature and then quenched with sat. aq. $NaHCO_3$ solution. The phases were separated and the aqueous layer was extracted with EtOAc (3 x 20 mL). The combined organic layers were washed with brine, dried over $MgSO_4$ and concentrated under reduced pressure. The residue was purified *via* column chromatography on silica gel (*n*-pentane/EtOAc 94:6) and **1q** (420 mg, 2.3 mmol, 77%) was obtained as a white solid. Spectral and physical data are in accordance with the literature.[20]

TLC: R_f **=** 0.2 (94:6 *n*-pentane/EtOAc).

NMR Spectroscopy (*see spectra*):

^{1}H NMR (400 MHz, $CDCl_3$): δ_H 8.11 (d, *J* = 0.9 Hz, 1H), 7.46 (d, *J* = 0.9 Hz, 1H), 4.33 (q, *J* = 7.1 Hz, 2H), 2.50 (s, 3H), 1.36 (t, *J* = 7.1 Hz, 3H) ppm.

Ethyl 2-(5-chloropyridin-3-yl)oxazole-5-carboxylate (1s)

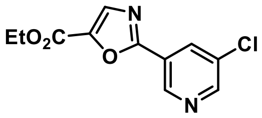

Synthesized following General Procedure D using: $Pd(OAc)_2$ (28 mg, 0.13 mmol, 5 mol%), $PCy_3{\cdot}HBF_4$ (65 mg, 0.18 mmol, 7 mol%), CuI (48 mg, 0.25 mmol, 10 mol%), DBU (0.75 mL, 5.0 mmol, 2.0 eq.), 3-bromo-5-chloropyridine (481 mg, 2.5 mmol, 1.0 eq.), ethyl oxazole-5-carboxylate (388 mg, 2.8 mmol, 1.1 eq.) and 1,4-dioxane (8.5 mL, 0.3 M). Purification *via* column chromatography on silica gel (*n*-pentane/EtOAc 100:0 – 80:20) afforded **1s** (347 mg, 1.37 mmol, 55%) as a white solid.

TLC: R_f **=** 0.60 (70:30 *n*-pentane/EtOAc).

NMR Spectroscopy (***see spectra***):

^{1}H NMR (400 MHz, $CDCl_3$): δ_H 9.23 (d, *J* = 1.9 Hz, 1H), 8.69 (d, *J* = 2.4 Hz, 1H), 8.45 – 8.32 (m, 1H), 7.86 (s, 1H), 4.43 (q, *J* = 7.1 Hz, 2H), 1.42 (t, *J* = 7.1 Hz, 3H) ppm;

^{13}C NMR (101 MHz, $CDCl_3$): δ_C 160.6, 157.6, 151.2, 146.1, 143.5, 135.4, 133.9, 132.7, 123.8, 62.0, 14.4 ppm.

HRMS (ESI^+): m/z calc'd for $C_{11}H_9ClN_2O_3H$ $[M+H]^+$: 253.0375, found 253.0373.

Ethyl 2-(1-(*tert*-butoxycarbonyl)-1*H*-indol-5-yl)oxazole-5-carboxylate (1t)

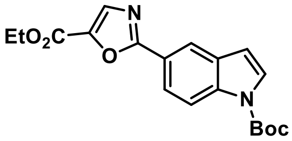

Synthesized following General Procedure D using: $Pd(OAc)_2$ (34 mg, 0.15 mmol, 5 mol%), $PCy_3{\cdot}HBF_4$ (78 mg, 0.21 mmol, 7 mol%), CuI (48 mg, 0.30 mmol, 10 mol%), DBU (0.75 mL, 6.0 mmol, 2.0 eq.), 5-bromo-1*H*-indole (588 mg, 3.0 mmol, 1.0 eq.), ethyl oxazole-5-carboxylate (466 mg, 3.3 mmol, 1.1 eq.) and 1,4-dioxane (8.5 mL, 0.3 M). Purification *via* column chromatography on silica gel (*n*-pentane/EtOAc 100:0 – 60:40) afforded ethyl 2-(1*H*-indol-5-yl)oxazole-5-carboxylate (446 mg, 1.74 mmol, 58%). The obtained product was Boc protected using Boc_2O (240 mg, 1.1 mmol, 1.1 eq.), DMAP (12.2 mg, 0.1 mmol, 10 mol%) and CH_2Cl_2 (20 mL) at r.t. Purification *via* column chromatography on silica gel (*n*-pentane/EtOAc 100:0 – 75:25) afforded **1t** (249 mg, 0.70 mmol, 70%) as a white solid.

TLC: R_f **=** 0.65 (80:20 *n*-pentane/EtOAc).

NMR Spectroscopy (***see spectra***):

^{1}H NMR (400 MHz, $CDCl_3$): δ_H 8.37 (d, J = 1.7 Hz, 1H), 8.25 (d, J = 8.7 Hz, 1H), 8.10 (dd, J = 8.7, 1.8 Hz, 1H), 7.84 (s, 1H), 7.65 (d, J = 3.7 Hz, 1H), 6.65 (d, J = 3.8 Hz, 1H), 4.42 (q, J = 7.1 Hz, 2H), 1.68 (s, 9H), 1.42 (t, J = 7.1 Hz, 3H) ppm;

^{13}C NMR (101 MHz, $CDCl_3$): δ_C 165.1, 158.1, 149.5, 142.2, 137.2, 135.6, 130.9, 127.5, 123.5, 121.2, 120.7, 115.7, 107.7, 84.5, 61.5, 28.3, 14.5 ppm.

HRMS (ESI$^+$): m/z calc'd for $C_{19}H_{20}N_2O_5Na$ [M+Na]$^+$: 379.1264, found 379.1261.

Ethyl 2-(pyrimidin-5-yl)oxazole-5-carboxylate (1u)

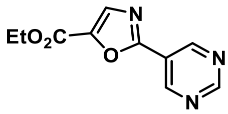

Synthesized following General Procedure D using: $Pd(OAc)_2$ (22.5 mg, 0.1 mmol, 5 mol%), $PCy_3{\cdot}HBF_4$ (52 mg, 0.14 mmol, 7 mol%), CuI (38 mg, 0.2 mmol, 10 mol%), DBU (0.60 mL, 4.0 mmol, 2.0 eq.), 1-bromo-3,5-difluorobenzene (318 mg, 2.0 mmol, 1.0 eq.), ethyl oxazole-5-carboxylate (283 mg, 2.2 mmol, 1.1 eq.) and 1,4-dioxane (7.0 mL, 0.3 M). Purification *via* column chromatography on silica gel (*n*-pentane/EtOAc 100:0 – 93:07) afforded **1u** (235 mg, 1.07 mmol, 57%) as a white solid.

TLC: R_f **=** 0.25 (80:20 *n*-pentane/EtOAc).

NMR Spectroscopy (***see spectra***):

^{1}H NMR (400 MHz, $CDCl_3$): δ_H 9.43 (s, 2H), 9.34 (s, 1H), 7.89 (s, 1H), 4.44 (q, J = 7.1 Hz, 2H), 1.42 (t, J = 7.1 Hz, 3H) ppm;

^{13}C NMR (101 MHz, $CDCl_3$): δ_C 160.4, 159.4, 157.5, 155.2, 143.7, 135.3, 121.5, 62.1, 14.4 ppm.

HRMS (ESI$^+$): m/z calc'd for $C_{10}H_9N_3O_3Na$ [M+Na]$^+$: 242.0536, found 242.0535.

Ethyl 2-(3,5-difluorophenyl)oxazole-5-carboxylate (1v)

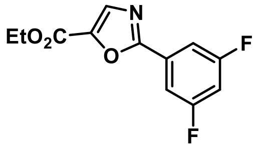

Synthesized following General Procedure D using: $Pd(OAc)_2$ (34 mg, 0.15 mmol, 5 mol%), $PCy_3{\cdot}HBF_4$ (78 mg, 0.21 mmol, 7 mol%), CuI (48 mg, 0.3 mmol, 10 mol%), DBU (0.75 mL, 6.0 mmol, 2.0 eq.), 1-bromo-3,5-difluorobenzene (579 mg, 3.0 mmol, 1.0 eq.), ethyl oxazole-5-carboxylate (466 mg, 3.3 mmol, 1.1 eq.) and 1,4-dioxane (8.5 mL, 0.3 M). Purification *via* column chromatography on silica gel (*n*-pentane/EtOAc 100:0 – 93:07) afforded **1v** (441 mg, 1.74 mmol, 35%) as a yellow solid.

TLC: R_f **=** 0.4 (95:05 *n*-pentane/EtOAc).

NMR Spectroscopy (***see spectra***):

^{1}H NMR (400 MHz, $CDCl_3$): δ_H 7.84 (s, 1H), 7.70 – 7.61 (m, 2H), 7.00 – 6.92 (m, 1H), 4.42 (q, *J* = 7.1 Hz, 2H), 1.41 (t, *J* = 7.2 Hz, 3H) ppm;

^{13}C{^{19}F} NMR (126 MHz, $CDCl_3$): δ_C 163.4, 162.0, 157.7, 143.1, 135.4, 129.3, 110.4, 107.1, 61.9, 14.4 ppm;

^{13}C NMR (126 MHz, $CDCl_3$): δ_C 163.4 (dd, *J* = 250.1, 12.5 Hz), 162.0 (t, *J* = 3.9 Hz), 157.7, 143.1, 135.4, 129.3 (t, *J* = 10.7 Hz), 110.4 (d, *J* = 28.2 Hz), 107.1 (t, *J* = 25.3 Hz), 61.9, 14.4 ppm;

^{19}F NMR (376 MHz, $CDCl_3$) δ_F -107.7 ppm.

HRMS (ESI^+): m/z calc'd for $C_{12}H_9F_2NO_3Na$ $[M+Na]^+$: 276.0443, found 276.0441.

Ethyl 2-(4-methoxyphenyl)oxazole-5-carboxylate (1w)

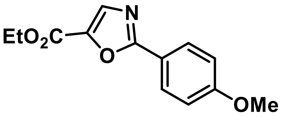

Synthesized following General Procedure **D** using: $Pd(OAc)_2$ (34 mg, 0.15 mmol, 5 mol%), $PCy_3{\cdot}HBF_4$ (78 mg, 0.21 mmol, 7 mol%), CuI (48 mg, 0.3 mmol, 10 mol%), DBU (0.75 mL, 6.0 mmol, 2.0 eq.), 1-bromo-4-methoxybenzene (561 mg, 3.0 mmol, 1.0 eq.), ethyl oxazole-5-carboxylate (466 mg, 3.3 mmol, 1.1 eq.) and 1,4-dioxane (8.5 mL, 0.3 M). Purification *via* column chromatography on silica gel (*n*-pentane/EtOAc 100:0 – 90:10) afforded **1w** (260 mg, 1.05 mmol, 35%) as a brown solid.

TLC: R_f **=** 0.75 (80:20 *n*-pentane/EtOAc).

NMR Spectroscopy (***see spectra***):

^{1}H NMR (400 MHz, $CDCl_3$): δ_H 8.16 – 8.01 (m, 2H), 7.79 (s, 1H), 7.06 – 6.91 (m, 2H), 4.40 (q, *J* = 7.1 Hz, 2H), 3.87 (s, 3H), 1.40 (t, *J* = 7.1 Hz, 3H) ppm;

^{13}C NMR (101 MHz, $CDCl_3$): δ_C 164.5, 162.5, 158.1, 142.0, 135.6, 129.2, 119.3, 114.5, 61.5, 55.6, 14.5 ppm.

HRMS (ESI^+): m/z calc'd for $C_{13}H_{13}NO_4Na$ $[M+Na]^+$: 270.0737, found 270.0734.

Ethyl 2-(3-(trifluoromethyl)phenyl)oxazole-5-carboxylate (1x)

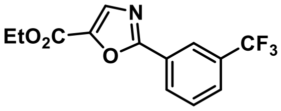

Synthesized following General Procedure D using: $Pd(OAc)_2$ (34 mg, 0.15 mmol, 5 mol%), $PCy_3{\cdot}HBF_4$ (78 mg, 0.21 mmol, 7 mol%), CuI (48 mg, 0.3 mmol, 10 mol%), DBU (0.75 mL, 6.0 mmol, 2.0 eq.), 1-bromo-3-

(trifluoromethyl)benzene (676 mg, 3.0 mmol, 1.0 eq.), ethyl oxazole-5-carboxylate (466 mg, 3.3 mmol, 1.1 eq.) and 1,4-dioxane (8.5 mL, 0.3 M). Purification *via* column chromatography on silica gel (*n*-pentane/EtOAc 100:0 – 90:10) afforded **1x** (411 mg, 1.44 mmol, 48%) as a brown solid.

TLC: R_f **=** 0.50 (90:10 *n*-pentane/EtOAc).

NMR Spectroscopy (***see spectra***):

^{1}**H NMR** (400 MHz, $CDCl_3$): δ_H 8.43 – 8.39 (m, 1H), 8.33 (dd, *J* = 7.8, 1.9 Hz, 1H), 7.86 (s, 1H), 7.80 – 7.73 (m, 1H), 7.63 (t, *J* = 7.9 Hz, 1H), 4.43 (q, *J* = 7.1 Hz, 2H), 1.42 (t, *J* = 7.2 Hz, 3H) ppm;

^{13}**C{**^{19}**F} NMR** (126 MHz, $CDCl_3$): δ_C 162.8, 157.8, 143.0, 135.4, 131.8, 130.4, 129.7, 128.2, 127.4, 124.3, 123.7, 61.8, 14.4 ppm;

^{13}**C NMR** (126 MHz, $CDCl_3$): δ_C 162.8, 157.8, 143.0, 135.4, 131.8 (q, *J* = 33.0 Hz), 130.4, 129.7, 128.2 (q, *J* = 3.7 Hz), 127.4, 124.3 (q, *J* = 4.2 Hz), 123.7 (q, *J* = 272.6 Hz), 61.8, 14.4 ppm;

^{19}**F NMR** (376 MHz, $CDCl_3$) δ_F -62.9 ppm.

HRMS (ESI^+): m/z calc'd for $C_{13}H_{10}F_3NO_3Na$ $[M+Na]^+$: 308.0505, found 308.0505.

Ethyl 4-methyl-2-phenyloxazole-5-carboxylate (1y)

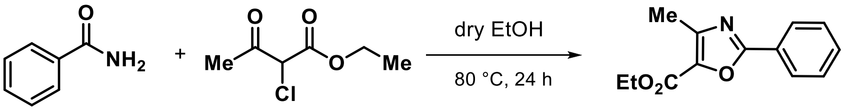

Following a literature procedure,[21] a Schlenk tube equipped with a stir bar was charged with benzamide (1.21 mg, 10.0 mmol, 1.0 eq.). After three rounds of evacuation and backfilling with argon, dry EtOH (15 mL) was added followed by ethyl 2-chloro-3-oxobutanoate (1.81 g, 11 mmol, 1.1 eq.). Then the Schlenk tube was sealed and heated at 80 °C for 24 hours. After completion the EtOH was evaporated and the residue was washed with water, extracted with ethyl acetate and the combined organic layers were dried with anhydrous Na_2SO_4 and filtered. Purification *via* column chromatography on silica gel (*n*-pentane/EtOAc 100:0 – 90:10) afforded **1y** (578 mg, 2.50 mmol, 25%) as a white solid.

TLC: R_f **=** 0.6 (90:10 *n*-pentane/EtOAc).

NMR Spectroscopy (***see spectra***):

^{1}**H NMR** (400 MHz, $CDCl_3$): δ_H 8.16 – 8.08 (m, 2H), 7.54 – 7.41 (m, 3H), 4.41 (q, *J* = 7.1 Hz, 2H), 2.54 (s, 3H), 1.41 (t, *J* = 7.1 Hz, 3H) ppm;

^{13}**C NMR** (101 MHz, $CDCl_3$): δ_C 162.4, 159.0, 147.2, 137.6, 131.6, 129.0, 127.3, 126.6, 61.2, 14.5, 13.7 ppm.

HRMS (ESI^+): m/z calc'd for $C_{13}H_{13}NO_3Na$ $[M+Na]^+$: 254.0788, found 254.0785.

1-(4-Cyclohexylthiophen-2-yl)ethan-1-one (1aa)

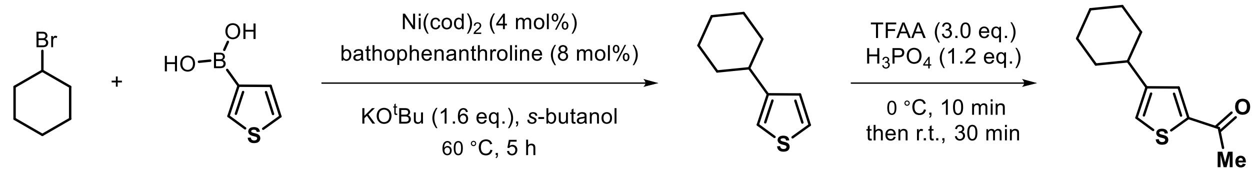

Following a literature procedure,[22] a Schlenk tube equipped with a stir bar was charged with $Ni(cod)_2$ (110 mg, 0.4 mmol, 4.0 mol%), bathophenanthroline (266 mg, 0.8 mM, 8.0 mol%), KO*t*Bu (1.80 g, 16.0 mmol, 1.6 eq.) and thiophen-3-ylboronic acid (1.54 g, 12 mmol, 1.2 eq.). After three rounds of evacuation and backfilling with argon, s-butanol was added (50 mL) was added and stirred for 10 min at r.t.. Then bromocyclohexane (1.63 g, 10.0 mmol, 1.0 eq.) was added under argon flow and the Schlenk tube was sealed. The reaction mixture was heatured at 60 °C for 5 hr. After completion, the reaction mixture was passed through a short silica plug and the residue was purified *via* column chromatography on silica gel (*n*-pentane/EtOAc 100:0) afforded 3-cyclohexylthiophene (721 mg, 4.3 mmol, 43%) as a colourless oil. The obtained 3-cyclohexylthiophene was acylated following a reported literature procedure in the next step.[23] To an oven-dried flask, acetic acid (258 mg, 4.3 mmol, 1.0 eq.) was added. The flask was then placed in ice bath and trifluoroacetic anhydride (TFAA, 3.61 mL, 17.2 mmol, 4.0 eq.) and ortho-phosphoric acid (301 µL, 5.2 mmol, 1.2 eq.) were added. To this mixture, 3-cyclohexylthiophene (715 mg, 4.3 mmol, 1.0 eq.) was added dropwise over 5 min and the reaction was stirred at 0 °C for another 10 min. After that the reaction was stirred at room temperature for 30 min. Upon completion, the mixture was diluted with ice cold dist. H_2O (25 mL) and quenched with slow portion-wise addition of Na_2CO_3 until the pH of the mixture rises to 8–9. The aqueous layer was extracted with CH_2Cl_2 (3 x 15 ml), and the combined organic layers were dried over magnesium sulfate, filtered and the solvent was removed under reduced pressure using a rotary evaporator. The residual mixture was purified by silica gel column chromatography (*n*-pentane/EtOAc 100:0 – 97:03) to afford the product **1aa** (355 mg, 1.70 mmol, 39%).

TLC: R_f **=** 0.25 (98:02 *n*-pentane/EtOAc).

NMR Spectroscopy (***see spectra***):

^{1}H NMR (400 MHz, $CDCl_3$): δ_H 7.57 (d, *J* = 1.5 Hz, 1H), 7.25 (d, *J* = 1.5 Hz, 1H), 2.65 – 2.54 (m, 1H), 2.53 (s, 3H), 2.03 – 1.90 (m, 2H), 1.88 – 1.67 (m, 3H), 1.44 – 1.30 (m, 4H), 1.31 – 1.16 (m, 1H) ppm;

^{13}C NMR (101 MHz, $CDCl_3$): δ_C 190.9, 150.4, 144.1, 132.3, 127.6, 39.7, 34.3, 27.0, 26.6, 26.1 ppm.

HRMS (ESI^+): m/z calc'd for $C_{12}H_{16}OSNa$ $[M+Na]^+$: 231.0814, found 231.0811.

2-(Furan-2-yl)-2-oxoethyl 2-(4-(2,2-dichlorocyclopropyl)phenoxy)-2-methylpropanoate (1ak)

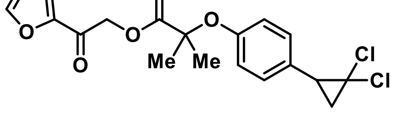

Synthesized following General Procedure C: 1-(Furan-2-yl)-2-hydroxyethan-1-one **1d** (63.0 mg, 0.5 mmol, 1.0 eq.), Ciprofibrate (224 mg, 0.75 mmol, 1.5 eq.), 4-dimethylaminopyridine (12.2 mg, 0.1 mmol, 0.2 eq.), 1-

ethyl-3-(3-dimethylaminopropyl)carbodiimide hydrochloride (192 mg, 1.0 mmol, 2.0 eq.) and CH_2Cl_2 (5 mL) were used to obtain **1ak** (181 mg, 0.46 mmol, 91%) as a slightly yellow oil.

TLC: R_f **=** 0.4 (83:17 *n*-pentane/EtOAc).

NMR Spectroscopy (*see spectra*):

> **^{1}H NMR** (400 MHz, $CDCl_3$): δ_H 7.63 – 7.58 (m, 1H), 7.28 (d, *J* = 3.8 Hz, 1H), 7.19 – 7.10 (m, 2H), 7.00 – 6.92 (m, 2H), 6.58 (dd, *J* = 3.7, 1.7 Hz, 1H), 5.27 (s, 2H), 2.84 (dd, *J* = 10.7, 8.5 Hz, 1H), 1.93 (dd, *J* = 7.4, 1.0 Hz, 1H), 1.81 – 1.76 (m, 1H), 1.70 (s, 6H) ppm;

> **^{13}C NMR** (101 MHz, $CDCl_3$): δ_C 181.3, 173.9, 154.8, 150.6, 147.0, 129.8, 128.5, 119.3, 118.0, 112.7, 79.3, 66.0, 61.0, 35.0, 26.0, 25.8, 25.7 ppm.

HRMS (ESI$^+$): m/z calc'd for $C_{19}H_{18}Cl_2O_5Na$ $[M+Na]^+$: 419.0424, found 419.0424.

(3*S*,5*R*,8*S*,9*S*,10*S*,13*R*,14*S*,17*S*)-17-((*R*)-5-(Furan-2-yl)-5-oxopentan-2-yl)-10,13-dimethylhexadecahydro-1*H*-cyclopenta[*a*]phenanthren-3-yl acetate (1al)

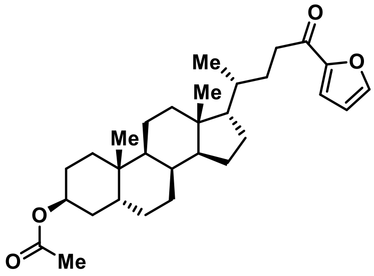

Following a literature procedure,[24] lithocholic acid (2.26 g, 6.0 mmol, 1.0 eq.) and DMAP (147 mg, 1.2 mmol, 0.2 eq.) were dissolved in dry CH_2Cl_2 (24 mL) in a Schlenk flask under argon. Acetic anhydride (2.04 mL, 21.6 mmol, 3.6 eq.) was added dropwise to the solution. The reaction mixture was stirred at room temperature for 1 h. Then the reaction mixture was washed with aq. HCl (1 N) solution (3 × 30 mL), aq. $NaHCO_3$ solution (5%, 3 x 30 mL), brine (3 x 20 mL) and distilled water (20 mL). The phases were separated and the organic layer was dried over $MgSO_4$ and concentrated. After purification *via* column chromatography on silica gel (*n*-pentane/EtOAc 80:20) 3*α*-acetoxy-5*β*-cholan-24-oic acid was obtained as a white solid (2.10 g, 5.02 mmol, 84%). In the second step, following a modified literature procedure,[25] a Schlenk flask was charged with palladium(II) acetate (50.5 mg, 0.23 mmol, 0.045 eq.), 3*α*-acetoxy-5*β*-cholan-24-oic acid (2.10 g, 5.0 mmol, 1.0 eq.), pivalic anhydride (2.30 mL, 11.3 mmol, 2.3 eq.; dissolved in 20 mL of dry THF), distilled H_2O (335 µL, 18.9 mmol, 3.8 eq.), 2-furanylboronic acid (1.00 g, 9.0 mmol, 1.8 eq.; dissolved in 10 mL of dry THF) and tris(*o*-anisyl)phosphine (185 mg, 0.53 mmol, 0.1 eq.) under argon atmosphere. The reaction mixture was then sonicated and stirred at 60 °C until TLC indicated complete conversion (19 h) of the starting material. The dark crude product was purified *via* column chromatography on silica gel (*n*-pentane/EtOAc 95:5) to obtain **1al** as a white solid (278 mg, 0.6 mmol, 12%). Spectral and physical data are in accordance with the literature.

TLC: R_f **=** 0.5 (95:5 *n*-pentane/EtOAc).

NMR Spectroscopy (*see spectra*):

^{1}H NMR (400 MHz, $CDCl_3$): δ_H 7.57 (dd, J = 1.8, 0.8 Hz, 1H), 7.17 (dd, J = 3.5, 0.8 Hz, 1H), 6.52 (dd, J = 3.6, 1.7 Hz, 1H), 4.78 – 4.65 (m, 1H), 2.90 – 2.68 (m, 2H), 2.03 (s, 3H), 2.00 – 1.00 (m, 29H), 0.97 (d, J = 6.3 Hz, 3H), 0.93 (s, 3H), 0.65 (s, 3H) ppm;

^{13}C NMR (101 MHz, $CDCl_3$): δ_C 190.5, 170.8, 153.0, 146.2, 116.8, 112.3, 74.6, 56.7, 56.2, 42.9, 42.1, 40.6, 40.3, 36.0, 35.7, 35.7, 35.2, 34.8, 32.4, 30.6, 28.4, 27.2, 26.8, 26.5, 24.4, 23.5, 21.6, 21.0, 18.7, 12.2 ppm.

2-(Furan-2-yl)-2-oxoethyl 5-(2,5-dimethylphenoxy)-2,2-dimethylpentanoate (1am)

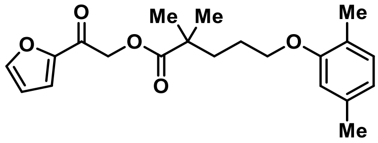

Synthesized following General Procedure C: 1-(Furan-2-yl)-2-hydroxyethan-1-one **1d** (88.2 mg, 0.7 mmol, 1.0 eq.), Gemfibrozil (263 mg, 1.1 mmol, 1.5 eq.), 4-dimethylaminopyridine (17.1 mg, 0.14 mmol, 0.2 eq.), 1-ethyl-3-(3-dimethylaminopropyl)carbodiimide hydrochloride (268 mg, 1.4 mmol, 2.0 eq.) and CH_2Cl_2 (7 mL) were used to obtain **1am** (104 mg, 0.3 mmol, 42%) as colourless oil.

TLC: R_f **=** 0.4 (92:8 *n*-pentane/EtOAc).

NMR Spectroscopy (*see spectra*):

^{1}H NMR (400 MHz, $CDCl_3$): δ_H 7.63 – 7.58 (m, 1H), 7.25 (d, J = 3.8 Hz, 1H), 6.99 (d, J = 7.4 Hz, 1H), 6.67 – 6.60 (m, 2H), 6.59 – 6.54 (m, 1H), 5.18 – 5.13 (m, 2H), 3.95 (t, J = 5.6 Hz, 2H), 2.30 (s, 3H), 2.18 (s, 3H), 1.91 – 1.76 (m, 4H), 1.31 (s, 6H) ppm;

^{13}C NMR (101 MHz, $CDCl_3$): δ_C 182.0, 177.4, 157.1, 150.9, 146.8, 136.6, 130.4, 123.7, 120.7, 117.6, 112.6, 112.1, 68.2, 65.3, 42.4, 37.3, 25.4, 25.2, 21.5, 15.9 ppm.

HRMS (ESI$^+$): m/z calc'd for $C_{21}H_{26}O_5Na$ $[M+Na]^+$: 381.1673, found 381.1707.

2-(Furan-2-yl)-2-oxoethyl (1*S*)-4,7,7-trimethyl-3-oxo-2-oxabicyclo[2.2.1]heptane-1-carboxylate (1an)

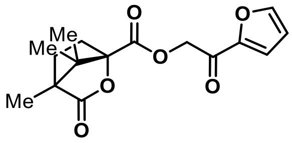

Synthesized following General Procedure C: 1-(Furan-2-yl)-2-hydroxyethan-1-one **1d** (88.2 mg, 0.7 mmol, 1.0 eq.), (1*S*)-Camphanic acid (198 mg, 1.1 mmol, 1.5 eq.), 4-dimethylaminopyridine (17.1 mg, 0.14 mmol, 0.2 eq.), 1-ethyl-3-(3-dimethylaminopropyl)carbodiimide hydrochloride (268 mg, 1.4 mmol, 2.0 eq.) and CH_2Cl_2 (7 mL) were used to obtain **1an** (212 mg, 0.7 mmol, 99%) as a white solid.

TLC: R_f **=** 0.3 (72:28 *n*-pentane/EtOAc).

NMR Spectroscopy (*see spectra*):

^{1}H NMR (400 MHz, $CDCl_3$): δ_H 7.62 (dd, J = 1.7, 0.8 Hz, 1H), 7.30 (dd, J = 3.6, 0.8 Hz, 1H), 6.60 (dd, J =

3.6, 1.7 Hz, 1H), 5.42 – 5.24 (m, 2H), 2.58 – 2.46 (m, 1H), 2.16 – 2.05 (m, 1H), 2.01 – 1.90 (m, 1H), 1.77 – 1.66 (m, 1H), 1.14 (d, *J* = 1.8 Hz, 6H), 1.13 (s, 3H) ppm;

^{13}C NMR (101 MHz, $CDCl_3$): δ_C 180.6, 178.2, 167.0, 150.5, 147.1, 118.1, 112.8, 91.2, 66.1, 55.1, 54.8, 30.9, 29.1, 16.7, 16.6, 9.9 ppm.

HRMS (ESI$^+$): m/z calc'd for $C_{16}H_{18}O_6Na$ [M+Na]$^+$: 329.0996, found 329.0995.

2-(Furan-2-yl)-2-oxoethyl 4-(*N*,*N*-dipropylsulfamoyl)benzoate (1ao)

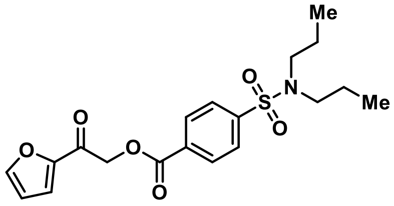

Synthesized following General Procedure C: 1-(Furan-2-yl)-2-hydroxyethan-1-one **1d** (88.2 mg, 0.7 mmol, 1.0 eq.), probenecid (300 mg, 1.1 mmol, 1.5 eq.), 4-dimethylaminopyridine (17.1 mg, 0.14 mmol, 0.2 eq.), 1-ethyl-3-(3-dimethylaminopropyl)carbodiimide hydrochloride (268 mg, 1.4 mmol, 2.0 eq.) and CH_2Cl_2 (7 mL) were used to obtain **1ao** (221 mg, 0.6 mmol, 80%) as white solid.

TLC: R_f **=** 0.3 (80:20 *n*-pentane/EtOAc).

NMR Spectroscopy (*see spectra*):

^{1}H NMR (400 MHz, $CDCl_3$): δ_H 8.27 – 8.22 (m, 2H), 7.94 – 7.87 (m, 2H), 7.67 – 7.62 (m, 1H), 7.35 – 7.31 (m, 1H), 6.65 – 6.58 (m, 1H), 5.46 (s, 2H), 3.15 – 3.06 (m, 4H), 1.62 – 1.49 (m, 4H), 0.88 (t, *J* = 7.4 Hz, 6H) ppm;

^{13}C NMR (101 MHz, $CDCl_3$): δ_C 181.3, 164.8, 150.7, 147.1, 144.8, 132.7, 130.8, 127.2, 118.1, 112.8, 66.3, 50.1, 22.1, 11.3 ppm.

HRMS (ESI$^+$): m/z calc'd for $C_{19}H_{23}NO_6SNa$ [M+Na]$^+$: 416.1138, found 416.1139.

2-(Furan-2-yl)-2-oxoethyl (2*S*,5*S*)-3,3-dimethyl-7-oxo-4-thia-1-azabicyclo[3.2.0]heptane-2-carboxylate 4,4-dioxide (1ap)

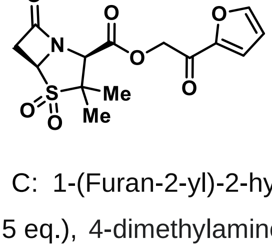

Synthesized following General Procedure C: 1-(Furan-2-yl)-2-hydroxyethan-1-one **1d** (88.2 mg, 0.7 mmol, 1.0 eq.), Sulbactam (250 mg, 1.1 mmol, 1.5 eq.), 4-dimethylaminopyridine (17.1 mg, 0.14 mmol, 0.2 eq.), 1-ethyl-3-(3-dimethylaminopropyl)carbodiimide hydrochloride (268 mg, 1.4 mmol, 2.0 eq.) and CH_2Cl_2 (7 mL) were used to obtain **1ap** (236 mg, 0.7 mmol, 99%) as a slightly yellow solid.

TLC: R_f **=** 0.4 (60:40 *n*-pentane/EtOAc).

NMR Spectroscopy (*see spectra*):

^{1}H NMR (400 MHz, $CDCl_3$): δ_H 7.67 – 7.62 (m, 1H), 7.30 (d, *J* = 3.6 Hz, 1H), 6.61 (dd, *J* = 3.7, 1.8 Hz, 1H), 5.60 – 5.51 (m, 1H), 5.16 – 5.07 (m, 1H), 4.69 – 4.65 (m, 1H), 4.50 (s, 1H), 3.54 – 3.39 (m, 2H), 1.70 (s, 3H), 1.67 (s, 3H) ppm;

^{13}C NMR (101 MHz, $CDCl_3$): δ_C 180.6, 171.2, 166.7, 150.3, 147.4, 118.4, 113.0, 66.4, 63.5, 63.3, 60.9, 38.1, 20.2, 17.9 ppm.

HRMS (ESI$^+$): m/z calc'd for $C_{14}H_{15}NO_7SNa$ [M+Na]$^+$: 364.0461, found 364.0460.

(3a*S*,4*S*,6a*R*)-4-(5-(Furan-2-yl)-5-oxopentyl)tetrahydro-1*H*-thieno[3,4-*d*]imidazol-2(3*H*)-one (1aq)

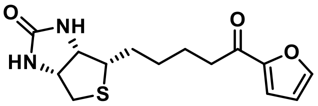

Following a modified literature procedure,[26] a Schlenk flask was charged with palladium acetate (112 mg, 0.5 mmol, 5 mol%), triphenylphosphine (262 mg, 1.0 mmol, 10 mol%), *D*-(+)-biotin (2.44 g, 10.0 mmol, 1.0 eq.) and 2-furanylboronic acid (1.34 g, 12.0 mmol, 1.2 eq.). After the reaction vessel was evacuated and backfilled with argon three times, THF (45 mL, 0.22 M), pivalic anhydride (3.0 mL, 15.0 mmol, 1.5 eq.) and distilled H_2O (431 µL) were added under argon. The reaction mixture was then heated to 50 °C and stirred for 90 hours. The solvent was removed under reduced pressure and the crude product was purified *via* column chromatography on silica gel (CH_2Cl_2/MeOH 96:4) to obtain **1aq** as a white solid (336 mg, 1.14 mmol, 11%).

TLC: R_f **=** 0.4 (96:4 CH_2Cl_2/MeOH).

NMR Spectroscopy (*see spectra*):

^{1}H NMR (400 MHz, $CDCl_3$): δ_H 7.58 (dd, *J* = 1.7, 0.8 Hz, 1H), 7.19 (dd, *J* = 3.6, 0.8 Hz, 1H), 6.53 (dd, *J* = 3.6, 1.7 Hz, 1H), 4.58 – 4.52 (m, 1H), 4.35 (dd, *J* = 7.9, 4.6 Hz, 1H), 3.23 – 3.14 (m, 1H), 2.93 (dd, *J* = 12.9, 5.0 Hz, 1H), 2.85 (td, *J* = 7.3, 1.5 Hz, 2H), 2.77 (d, *J* = 12.9 Hz, 1H), 1.90 – 1.63 (m, 4H), 1.59 – 1.41 (m, 2H) ppm;

^{13}C NMR (101 MHz, $CDCl_3$): δ_C 189.7, 163.8, 152.8, 146.5, 117.3, 112.4, 62.2, 60.5, 55.5, 40.7, 38.1, 28.6, 28.5, 24.2 ppm.

HRMS (ESI$^+$): m/z calc'd for $C_{14}H_{18}N_2O_3SNa$ [M+Na]$^+$: 317.0930, found 317.0929.

***N,N*-Dimethylfuran-2-carboxamide (1aw)**

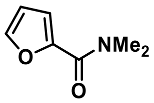

In an oven-dried 10 mL Schlenk tube under Ar, dimethylamine hydrochloride (89.7 mg, 1.1 mmol, 1.1 eq.) and

furan-2-carbonyl chloride (98.7 µL, 1.0 mmol, 1.0 eq.) were dissolved in dry CH_2Cl_2 (2 mL), cooled to 0 °C, and triethylamine (314 µL, 2.25 mmol, 2.25 eq.) was added dropwise. The reaction was let warm up to room temperature and stirred for 2 h. Upon completion, the reaction mixture was directly dry loaded onto silica and purified by column chromatography on silica gel (*n*-pentane/EtOAc 50:50 to 20:80) to obtain **1aw** as a colorless oil (98.6 mg, 0.71 mmol, 71%). Spectral data are in accordance with the literature.[27]

NMR Spectroscopy (*see spectra*):

^{1}**H NMR** (400 MHz, $CDCl_3$): δ_H 7.50 – 7.44 (m, 1H), 7.00 – 6.94 (m, 1H), 6.46 (dd, J = 3.4, 1.8 Hz, 1H), 3.17 (s, 6H) ppm;

1-(5-Bromofuran-2-yl)ethan-1-one (1ba)

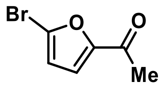

Synthesized following General Procedure A: 2-Bromo-*N*-methoxy-*N*-methylfuran-2-carboxamide (468 mg, 2.0 mmol, 1.0 eq.), methylmagnesium chloride (1.30 mL, 4.0 mmol, 2.0 eq.) and THF (6.70 mL, 0.3 M) were used to obtain **1ba** (334 mg, 1.8 mmol, 89%) as a white solid. Due to complete conversion of the educt, no purification *via* column chromatography was required. Spectral and physical data are in accordance with the literature data.[28]

TLC: R_f **=** 0.2 (90:10 *n*-pentane/EtOAc).

NMR Spectroscopy (*see spectra*):

^{1}**H NMR** (400 MHz, $CDCl_3$): δ_H 7.12 (d, J = 3.6 Hz, 1H), 6.49 (d, J = 3.6 Hz, 1H), 2.46 (s, 3H) ppm.

1-(5-Phenylfuran-2-yl)ethan-1-one (1bb)

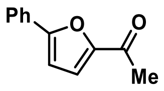

Synthesized following General Procedure B: 1-(5-Bromofuran-2-yl)ethan-1-one **1j** (309 mg, 1.64 mmol, 1.0 eq.), Phenylboronic acid (400 mg, 3.28 mmol, 2.0 eq.), Caesium carbonate (1.05 g, 3.28 mmol, 2.0 eq.), Tetrakis(triphenylphosphine)palladium(0) (94.7 mg, 0.082 mmol, 5mol%) and toluene/H_2O (9:1, 4.0 mL, 0.4 M) were used to obtain **1bb** (262 mg, 1.41 mmol, 86%) as a yellow liquid. Spectral and physical data are in accordance with the literature data.[29]

TLC: R_f **=** 0.4 (95:5 *n*-pentane/EtOAc).

NMR Spectroscopy (*see spectra*):

^{1}**H NMR** (400 MHz, $CDCl_3$): δ_H 7.82 – 7.75 (m, 2H), 7.46 – 7.39 (m, 2H), 7.39 – 7.32 (m, 1H), 7.25 (s, 1H), 6.77 (d, J = 3.7 Hz, 1H), 2.53 (s, 3H).

1-(Furan-3-yl)ethan-1-one (1bc)

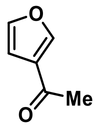

Synthesized following General Procedure A: *N*-methoxy-*N*-methylfuran-3-carboxamide (310 mg, 2.0 mmol, 1.0 eq.), methylmagnesium chloride (1.3 mL, 4.0 mmol, 2.0 eq.) and THF (6.7 mL, 0.3 M) were used to obtain **1bc** (198 mg, 1.8 mmol, 89%) as an off-white solid. Due to complete conversion of the educt, no purification *via* column chromatography was required. Spectral and physical data are in accordance with the literature data.[30]

TLC: R_f **=** 0.5 (95:5 *n*-pentane/EtOAc).

NMR Spectroscopy (*see spectra*):

^{1}H NMR (400 MHz, $CDCl_3$): δ_H 8.02 (dd, *J* = 1.5, 0.9 Hz, 1H), 7.46 – 7.41 (m, 1H), 6.76 (dd, *J* = 1.9, 0.8 Hz, 1H), 2.44 (s, 3H).

Ethyl 1-cyano-2-vinylcyclopropane-1-carboxylate (2d)

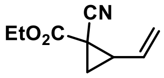

The title compound was prepared according to a modified literature procedure.[31] Ethyl 2-cyanoacetate (320 µL, 3.0 mmol, 1.0 eq.) and (*E*)-1,4-dibromobut-2-ene (641.7 mg, 3.0 mmol, 1.0 eq.) were dissolved in THF (25 mL) and K_2CO_3 (1.25 g, 7.5 mmol, 2.5 eq.) was added. The reaction mixture was refluxed overnight, filtered over celite, and the filter cake washed with Et_2O. The filtrate was washed with sat. aq. $NaHCO_3$-solution, H_2O and brine, dried over Na_2SO_4, filtered and concentrated. Purification by column chromatography on silica gel (*n*-pentane/EtOAc 100:0 – 95:5) yielded **2d** as a slightly yellow oil (357 mg, 2.2 mmol, 72%, 50:50 d.r.).

TLC: R_f **=** 0.3 (95:5 *n*-pentane/EtOAc).

NMR Spectroscopy (*see spectra*):

^{1}H NMR (400 MHz, $CDCl_3$): δ_H 5.74 – 5.57 (m, 1H), 5.49 – 5.40 (m, 1H), 5.38 (dd, *J* = 10.3, 1.0 Hz, 0.5H, d_1), 5.28 (d, *J* = 10.2 Hz, 0.5H, d_2), 4.31 – 4.22 (m, 2H), 2.66 – 2.59 (m, 1H), 2.02 – 1.85 (m, 1.5H), 1.65 (dd, *J* = 7.9, 5.0 Hz, 0.5H), 1.33 (q, *J* = 7.2 Hz, 3H) ppm;

^{13}C NMR (101 MHz, $CDCl_3$): δ_C 167.3, 165.3, 132.3, 130.7, 121.5, 121.0, 118.9, 116.9, 63.2, 63.0, 35.9, 33.9, 24.0, 22.7, 21.3, 20.5, 14.3, 14.2 ppm.

HRMS (ESI$^+$): m/z calc'd for $C_9H_{11}NO_2Na$ $[M+Na]^+$: 188.0682, found188.0681.

(3-Cyclopropylbut-3-en-1-yl)cyclohexane (2h)

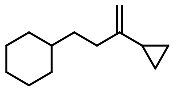

Synthesized following General Procedure E. Step 1 using: 3-Cyclohexylpropanoic acid (3.12 g, 20 mmol, 1.0 eq.), carbonyldiimidazole (4.22 g, 26 mmol, 1.3 eq.), *N,O*-dimethylhydroxylamine hydrochloride (1.70 g, 40 mmol, 2.0 eq.) and CH_2Cl_2 (50 mL, 0.4 M). 3-Cyclohexyl-*N*-methoxy-*N*-methylpropanamide was obtained after purification by column chromatography on silica gel (*n*-pentane/EtOAc 50:50) as a colourless oil (3.84 g, 19.3 mmol, 97%).

Step 2 using: 3-Cyclohexyl-*N*-methoxy-*N*-methylpropanamide (2.39 g, 12 mmol, 1.0 eq.) in THF (12 mL), Mg chips (481 mg, 19.8 mmol, 1.7 eq.) and bromocyclopropane (1.58 mL, 2.40 g, 19.8 mmol, 1.7 eq.) in THF (22 mL). Extraction with EtOAc and purification by column chromatography on silica gel (*n*-pentane/EtOAc 100:0 – 0:100) afforded 3-cyclohexyl-1-cyclopropylpropan-1-one as a yellow liquid (1.81 g, 10.1 mmol, 84%).

Step 3 using: 3-Cyclohexyl-1-cyclopropylpropan-1-one (1.08 g, 6.0 mmol, 1.0 eq.) in THF (10 mL), $MePPh_3Br$ (2.60 g, 7.2 mmol, 1.2 eq.) in THF (10 mL) and NaHMDS (2 M solution in THF, 3.6 mL, 1.32 g, 7.2 mmol, 1.2 eq.). Extraction with EtOAc, followed by purification by column chromatography on silica gel (*n*-pentane) afforded **2h** as a clear oil (1.00 g, 5.6 mmol, 93%, 75% over three steps).

TLC: R_f **=** 0.6 (*n*-pentane).

NMR Spectroscopy (*see spectra*):

> ^{1}**H NMR** (400 MHz, $CDCl_3$): δ_H 4.64 – 4.57 (m, 2H), 2.08 – 1.98 (m, 2H), 1.78 – 1.59 (m, 5H), 1.43 – 1.33 (m, 2H), 1.33 – 1.08 (m, 5H), 0.97 – 0.83 (m, 2H), 0.67 – 0.57 (m, 2H), 0.47 – 0.39 (m, 2H) ppm;
>
> ^{13}**C NMR** (101 MHz, $CDCl_3$): δ_C 151.9, 105.7, 37.7, 36.2, 33.6, 33.4, 26.9, 26.6, 16.3, 6.2 ppm.

HRMS (EI): m/z calc'd for $C_{13}H_{22}$ $[M]^+$: 178.1722, found 178.1719.

1-(2-Cyclopropylallyl)-4-fluorobenzene (2i)

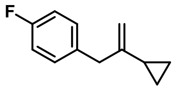

Synthesized following General Procedure E. Step 1 using: 2-(4-Fluorophenyl)acetic acid (3.08 g, 20 mmol, 1.0 eq.), carbonyldiimidazole (4.22 g, 26 mmol, 1.3 eq.), *N,O*-dimethylhydroxylamine hydrochloride (1.70 g, 40 mmol, 2.0 eq.) and CH_2Cl_2 (50 mL, 0.4 M). 2-(4-Fluorophenyl)-*N*-methoxy-*N*-methyl-acetamide was obtained after purification by column chromatography on silica gel (*n*-pentane/EtOAc 60:40 – 50:50) as an off-white oil (3.86 g, 19.6 mmol, 98%).

Step 2 using: 2-(4-Fluorophenyl)-*N*-methoxy-*N*-methyl-acetamide (2.37 g, 12 mmol, 1.0 eq.) in THF (12 mL), Mg chips (481 mg, 19.8 mmol, 1.7 eq.) and bromocyclopropane (1.58 mL, 2.40 g, 19.8 mmol, 1.7 eq.) in THF (22 mL). Extraction with EtOAc and purification by column chromatography on silica gel (*n*-pentane/EtOAc 100:0 – 0:100) afforded 1-cyclopropyl-2-(4-fluorophenyl)ethan-1-one as an orange solid (535 mg, 3.0 mmol, 25%).

Step 3 using: 1-Cyclopropyl-2-(4-fluorophenyl)ethan-1-one (499 mg, 2.8 mmol, 1.0 eq.) in THF (4.7 mL), $MePPh_3Br$ (1.20 g, 3.4 mmol, 1.2 eq.) in THF (4.7 mL) and NaHMDS (2 M solution in THF, 1.68 mL, 616 mg, 3.4 mmol, 1.2 eq.). Extraction with EtOAc, followed by purification by column chromatography on silica gel (*n*-pentane) afforded **2i** as a clear oil (201 mg, 1.14 mmol, 41%, 10% over three steps). Spectral and physical data are in accordance with the literature.[32]

TLC: R_f **=** 0.4 (*n*-pentane).

NMR Spectroscopy (*see spectra*):

^{1}H NMR (400 MHz, $CDCl_3$): δ_H 7.17 (dd, *J* = 8.7, 5.4 Hz, 2H), 6.97 (t, *J* = 8.8 Hz, 2H), 4.72 (s, 1H), 4.60 (d, *J* = 1.4 Hz, 1H), 3.35 (s, 2H), 1.25 (ddd, *J* = 13.6, 8.4, 5.5 Hz, 1H), 0.63 – 0.56 (m, 2H), 0.45 – 0.39 (m, 2H) ppm;

^{13}C{^{19}F} NMR (126 MHz, $CDCl_3$): δ_C 161.6, 150.4, 135.6, 130.5, 115.1, 108.6, 42.2, 16.0, 16.0, 6.4 ppm;

^{13}C NMR (126 MHz, $CDCl_3$): δ_C 161.6 (d, *J* = 243.4 Hz), 150.4, 135.6 (d, *J* = 3.3 Hz), 130.5 (d, *J* = 7.6 Hz), 115.1 (d, *J* = 21.2 Hz), 108.6, 42.2, 16.0 (d, *J* = 1.7 Hz), 6.4 ppm;

^{19}F NMR (376 MHz, $CDCl_3$): δ_F -117.6 ppm.

HRMS (EI): m/z calc'd for $C_{12}H_{13}F$ $[M]^+$: 176.0996, found 176.0994.

Hex-1-en-5-yn-2-ylcyclopropane (2j)

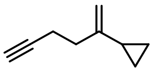

Synthesized following General Procedure E. Step 1 using: Pent-4-ynoic acid (1.96 g, 20 mmol, 1.0 eq.), carbonyldiimidazole (4.22 g, 26 mmol, 1.3 eq.), *N,O*-dimethylhydroxylamine hydrochloride (1.70 g, 40 mmol, 2.0 eq.) and CH_2Cl_2 (50 mL, 0.4 M). *N*-Methoxy-*N*-methylpent-4-ynamide was obtained after purification by column chromatography on silica gel (*n*-pentane/EtOAc 50:50 – 40:60) as an off-white oil (2.80 g, 19.8 mmol, 99%).

Step 2 using: *N*-Methoxy-*N*-methylpent-4-ynamide (1.41 g, 10 mmol, 1.0 eq.) in THF (10 mL), Mg chips (335 mg, 13.8 mmol, 1.4 eq.) and bromocyclopropane (1.10 mL, 1.67 g, 13.8 mmol, 1.4 eq.) in THF (15 mL). Extraction with CH_2Cl_2 and purification by column chromatography on silica gel (*n*-pentane/CH_2Cl_2 100:0 – 20:80) afforded 1-cyclopropylpent-4-yn-1-one as an off-white oil (525 mg, 4.3 mmol, 43%).

Step 3 using: 1-Cyclopropylpent-4-yn-1-one (489 mg, 4.0 mmol, 1.0 eq.) in THF (7 mL), $MePPh_3Br$ (1.71 g, 4.8 mmol, 1.2 eq.) in THF (7 mL) and NaHMDS (2 M solution in THF, 2.4 mL, 880 mg, 4.8 mmol, 1.2 eq.). Extraction with CH_2Cl_2, followed by purification by column chromatography on silica gel (*n*-pentane) afforded **2j** as a volatile, clear liquid (426 mg, product:*n*-pentane 1:0.96; corrected: 326 mg, 1.44 mmol, 36%, 15% over three steps).

Note: Due to volatility of the product, pentane is still present in the final compound.

TLC: R_f **=** 0.3 (*n*-pentane).

NMR Spectroscopy (*see spectra*):

^{1}H NMR (400 MHz, $CDCl_3$): δ_H 4.71 – 4.68 (m, 2H), 2.43 – 2.37 (m, 2H), 2.32 – 2.26 (m, 2H), 1.96 (t, J = 2.6 Hz, 1H), 1.36 – 1.19 (m, pentane and product, 1H), 0.67 – 0.61 (m, 2H), 0.48 – 0.42 (m, 2H) ppm;

^{13}C NMR (101 MHz, $CDCl_3$): δ_C 149.1, 107.4, 84.4, 68.5, 35.2, 17.7, 16.0, 6.03 ppm.

HRMS (EI): m/z calc'd for C_9H_{12} $[M]^+$: 120.0934, found 120.0934.

2.3 Substrate Scope

Dimethyl (*Z*)-1-(3-phenylpropanoyl)-10-oxabicyclo[5.2.1]deca-4,8-diene-2,2-dicarboxylate (3a) & dimethyl (*Z*)-7-(3-phenylpropanoyl)-10-oxabicyclo[5.2.1]deca-4,8-diene-2,2-dicarboxylate (3a')

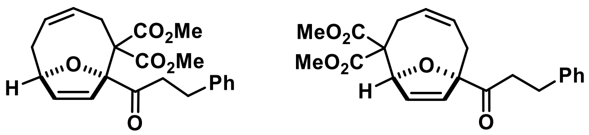

3a **3a'**

Synthesized following General Procedure F using: 1-(furan-2-yl)-3-phenylpropan-1-one **1a** (40.0 mg, 0.2 mmol, 1.0 eq.), dimethyl 2-vinylcyclopropane-1,1-dicarboxylate **2a** (92.0 mg, 0.5 mmol, 2.5 eq.), Ir-F (4.0 mg, 0.004 mmol, 2 mol%) and MeCN (2 mL). From crude ^{1}H NMR analysis, a crude yield of 77% and 83:17 regioisomeric ratio was determined. Purification *via* column chromatography on silica gel (*n*-pentane/EtOAc 100:0 – 92:8) afforded the title compound **3a** (49.2 mg, 0.13 mmol, 64%) as colourless oil, as well as the minor regioisomer **3a'** (10.0 mg, 0.03 mmol, 13%) as a colourless oil.

Characterization data for **3a**:

TLC: R_f **=** 0.3 (90:10 *n*-pentane/EtOAc).

NMR Spectroscopy (*see spectra*):

^{1}H NMR (599 MHz, $CDCl_3$): δ_H 7.24 (d, J = 7.5 Hz, 2H), 7.19 – 7.13 (m, 3H), 5.97 (d, J = 5.9 Hz, 1H), 5.86 (dd, J = 5.9, 1.6 Hz, 1H), 5.84 – 5.78 (m, 1H), 5.61 – 5.54 (m, 1H), 5.29 – 5.25 (m, 1H), 3.82 (s, 3H), 3.73 (s, 3H), 3.23 – 3.14 (m, 1H), 2.92 – 2.71 (m, 3H), 2.69 – 2.60 (m, 2H), 2.39 (dd, J = 13.7, 8.3 Hz, 1H), 2.26 – 2.19 (m, 1H) ppm;

^{13}C NMR (101 MHz, $CDCl_3$): δ_C 209.7, 170.9, 169.9, 141.7, 134.8, 129.7, 128.5, 128.4, 128.4, 127.5, 126.0, 99.9, 84.6, 67.0, 52.8, 52.7, 38.3, 33.1, 32.1, 29.5 ppm.

Characterization data for **3a'**:

TLC: R_f **=** 0.4 (90:10 *n*-pentane/EtOAc).

NMR Spectroscopy (*see spectra*):

^{1}H NMR (599 MHz, $CDCl_3$): δ_H 7.29 – 7.27 (m, 2H), 7.21 – 7.16 (m, 3H), 6.00 – 5.92 (m, 1H), 5.81 (dd, J = 6.0, 1.8 Hz, 1H), 5.69 (t, J = 2.1 Hz, 1H), 5.45 (dd, J = 6.0, 2.2 Hz, 1H), 5.44 – 5.38 (m, 1H), 3.77 (s, 3H), 3.76 (s, 3H), 3.14 – 3.05 (m, 1H), 3.02 – 2.93 (m, 1H), 2.93 – 2.81 (m, 2H), 2.76 – 2.65 (m, 2H), 2.60 – 2.49 (m, 2H) ppm;

^{13}C NMR (151 MHz, $CDCl_3$): δ_C 210.8, 171.4, 169.2, 141.4, 131.6, 130.0, 129.1, 129.0, 128.6, 128.6, 126.2, 101.1, 86.7, 63.1, 53.3, 52.7, 39.7, 35.2, 29.6, 29.0 ppm.

HRMS (ESI$^+$): m/z calc'd for $C_{22}H_{24}O_6Na$ $[M+Na]^+$: 407.1465, found 407.1465.

Dimethyl (*Z*)-1-propionyl-10-oxabicyclo[5.2.1]deca-4,8-diene-2,2-dicarboxylate (3b) & dimethyl (*Z*)-7-propionyl-10-oxabicyclo[5.2.1]deca-4,8-diene-2,2-dicarboxylate (3b')

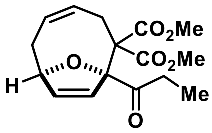

3b

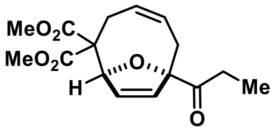

3b'

Synthesized following General Procedure F using: 1-(furan-2-yl)propan-1-one **1b** (24.8 mg, 0.2 mmol, 1.0 eq.), dimethyl 2-vinylcyclopropane-1,1-dicarboxylate **2a** (92.0 mg, 0.5 mmol, 2.5 eq.), Ir-F (4.0 mg, 0.004 mmol, 2 mol%) and MeCN (2 mL). From crude ^{1}H NMR analysis, a crude yield of 68% and 84:16 regioisomeric ratio was determined. Purification *via* column chromatography on silica gel (*n*-pentane/EtOAc 100:0 – 90:10) afforded the title compound **3b** (33.6 mg, 0.11 mmol, 55%) as white solid, as well as the minor regioisomer **3b'** (7.2 mg, 0.023 mmol, 12%) as a white solid.

<u>Scale-up:</u> For scaling up the reaction, a 150 mL Schlenk tube with two blue LEDs (30 W, λ_{max} = 450 nm), one on each side, and an external fan were employed. The reaction on 5.0 mmol scale afforded **3b** (830 mg, 2.7 mmol, 54%) as well as the minor regioisomer **3b'** (189 mg, 0.6 mmol, 12%). During the purification *via* column chromatography, remaining VCP **2a** (1.15 g, 6.2 mmol, 125%) could be recovered and reused in the (5+4) photo-cycloaddition.

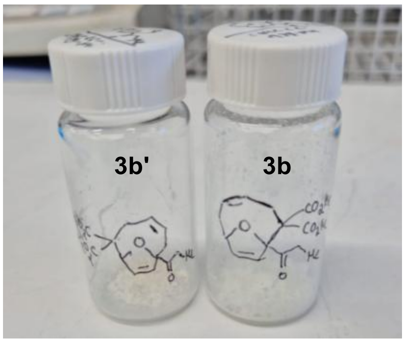

<u>Characterization data for **3b**:</u>

TLC: R_f **=** 0.3 (90:10 *n*-pentane/EtOAc).

NMR Spectroscopy (*see spectra*):

^{1}H NMR (599 MHz, $CDCl_3$): δ_H 6.01 (d, *J* = 5.9 Hz, 1H), 5.89 (d, *J* = 5.9 Hz, 1H), 5.85 – 5.77 (m, 1H), 5.61 – 5.56 (m, 1H), 5.33 – 5.25 (m, 1H), 3.80 (s, 3H), 3.74 (s, 3H), 2.93 – 2.78 (m, 1H), 2.70 – 2.54 (m, 2H), 2.51 – 2.31 (m, 2H), 2.26 – 2.19 (m, 1H), 1.00 (t, *J* = 7.2 Hz, 3H) ppm;

^{13}C NMR (101 MHz, $CDCl_3$): δ_C 211.5, 170.8, 170.0, 134.7, 129.8, 128.4, 127.7, 100.0, 84.4, 67.1, 52.8, 52.6, 33.1, 32.1, 29.8, 7.4 ppm.

<u>Characterization data for **3b'**:</u>

TLC: R_f **=** 0.4 (90:10 *n*-pentane/EtOAc).

NMR Spectroscopy (*see spectra*):

^{1}H NMR (599 MHz, $CDCl_3$): δ_H 6.03 – 5.91 (m, 1H), 5.81 (dd, *J* = 6.0, 1.8 Hz, 1H), 5.70 (t, *J* = 2.1 Hz, 1H), 5.50 (dd, *J* = 6.0, 2.3 Hz, 1H), 5.46 – 5.39 (m, 1H), 3.78 (s, 3H), 3.76 (s, 3H), 2.85 – 2.64 (m, 4H), 2.62 – 2.48 (m, 2H), 1.03 (t, *J* = 7.2 Hz, 3H) ppm;

^{13}C NMR (101 MHz, $CDCl_3$)*:* δ_C 212.5, 171.5, 169.2, 131.8, 130.1, 129.0, 128.7, 101.2, 86.6, 63.1, 53.3, 52.7, 35.4, 31.2, 29.0, 7.5 ppm.

HRMS (ESI$^+$): m/z calc'd for $C_{16}H_{20}O_6Na$ $[M+Na]^+$: 331.1152, found 331.1149.

Dimethyl (*Z*)-1-(2-cyanoacetyl)-10-oxabicyclo[5.2.1]deca-4,8-diene-2,2-dicarboxylate (3c)

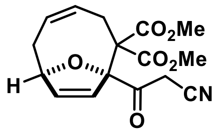

3c

Synthesized following General Procedure F using: 3-(furan-2-yl)-3-oxopropanenitrile (27.0 mg, 0.2 mmol, 1.0 eq.), dimethyl 2-vinylcyclopropane-1,1-dicarboxylate **2a** (92.0 mg, 0.5 mmol, 2.5 eq.), Ir-F (4.0 mg, 0.004 mmol, 2 mol%) and MeCN (2 mL). From crude ^{1}H NMR analysis, a crude yield of 53% and >95:5 regioisomeric ratio was determined. Purification *via* column chromatography on silica gel (*n*-pentane/EtOAc 100:0 – 70:30) afforded the title compound **3c** (33.3 mg, 0.10 mmol, 52%) as a single regioisomer as a white solid.

TLC: R_f **=** 0.2 (70:30 *n*-pentane/EtOAc).

NMR Spectroscopy (*see spectra*):

^{1}H NMR (400 MHz, $CDCl_3$): δ_H 6.14 (dd, *J* = 5.8, 1.7 Hz, 1H), 5.89 (dd, *J* = 5.9, 1.6 Hz, 1H), 5.86 – 5.77 (m, 1H), 5.68 – 5.57 (m, 1H), 5.36 – 5.27 (m, 1H), 3.90 (d, *J* = 19.3 Hz, 1H), 3.80 (s, 3H), 3.78 (s, 3H), 3.72 (d, *J* = 19.3 Hz, 1H), 2.72 – 2.63 (m, 1H), 2.61 – 2.54 (m, 1H), 2.46 – 2.31 (m, 1H), 2.30 – 2.18 (m, 1H) ppm;

^{13}C NMR (101 MHz, $CDCl_3$): δ_C 198.6, 170.7, 169.2, 136.4, 129.7, 128.4, 126.0, 114.3, 99.1, 84.5, 67.9, 53.1, 53.1, 32.6, 31.6, 28.3 ppm.

HRMS (ESI$^+$): m/z calc'd for $C_{16}H_{17}NO_6Na$ [M+Na]$^+$: 342.0948, found 342.0949.

Dimethyl (*Z*)-1-(2-hydroxyacetyl)-10-oxabicyclo[5.2.1]deca-4,8-diene-2,2-dicarboxylate (3d) & dimethyl (*Z*)-7-(2-hydroxyacetyl)-10-oxabicyclo[5.2.1]deca-4,8-diene-2,2-dicarboxylate (3d')

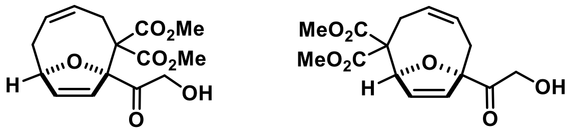

3d 3d'

Synthesized following General Procedure F using: 1-(furan-2-yl)-2-hydroxyethan-1-one **1d** (25.2 mg, 0.2 mmol, 1.0 eq.), dimethyl 2-vinylcyclopropane-1,1-dicarboxylate **2a** (92.0 mg, 0.5 mmol, 2.5 eq.), Ir-F (4.0 mg, 0.004 mmol, 2 mol%) and MeCN (2 mL). From crude ^{1}H NMR analysis, a crude yield of 67% and 81:19 regioisomeric ratio was determined. Purification *via* column chromatography on silica gel (*n*-pentane/EtOAc 100:0 – 60:40) afforded the title compound **3d** (29.5 mg, 0.095 mmol, 48%) as a colourless oil, as well as the minor regioisomer **3d'** (8.0 mg, 0.026 mmol, 13%) as a colourless oil.

Characterization data for **3d**:

TLC: R_f **=** 0.3 (60:40 *n*-pentane/EtOAc).

NMR Spectroscopy (*see spectra*):

^{1}H NMR (400 MHz, $CDCl_3$): δ_H 6.05 (dd, *J* = 6.0, 1.7 Hz, 1H), 5.92 (dd, *J* = 5.9, 1.6 Hz, 1H), 5.87 – 5.75 (m, 1H), 5.65 – 5.54 (m, 1H), 5.33 – 5.23 (m, 1H), 4.74 (d, *J* = 19.5 Hz, 1H), 4.28 (d, *J* = 19.5 Hz, 1H), 3.82 (s, 3H), 3.75 (s, 3H), 2.72 – 2.58 (m, 2H), 2.45 – 2.34 (m, 1H), 2.29 – 2.17 (m, 1H) ppm;

^{13}C NMR (151 MHz, $CDCl_3$): δ_C 209.7, 170.5, 169.5, 135.2, 129.5, 128.5, 126.9, 98.7, 84.9, 67.4, 65.0, 53.0, 52.9, 32.9, 31.8 ppm.

Characterization data for **3d'**:

TLC: R_f **=** 0.4 (60:40 *n*-pentane/EtOAc).

NMR Spectroscopy (*see spectra*):

^{1}H NMR (599 MHz, $CDCl_3$): δ_H 6.04 – 5.96 (m, 1H), 5.87 (dd, *J* = 6.0, 1.8 Hz, 1H), 5.69 (t, *J* = 2.1 Hz, 1H), 5.51 (dd, *J* = 6.0, 2.3 Hz, 1H), 5.47 – 5.39 (m, 1H), 4.68 (d, *J* = 20.6 Hz, 1H), 4.53 (d, *J* = 20.6 Hz, 1H), 3.79 (s, 3H), 3.77 (s, 3H), 2.80 – 2.73 (m, 1H), 2.71 – 2.64 (m, 1H), 2.64 – 2.56 (m, 2H) ppm;

^{13}C NMR (151 MHz, $CDCl_3$): δ_C 211.2, 171.2, 169.0, 130.8, 129.5, 129.3, 129.3, 100.2, 86.8, 65.9, 63.0, 53.3, 52.7, 35.6, 29.0 ppm.

HRMS (ESI^+): m/z calc'd for $C_{15}H_{18}O_7Na$ $[M+Na]^+$: 333.0945, found 333.0958.

Dimethyl (*Z*)-1-(3,5-dimethyl-1H-pyrazole-1-carbonyl)-10-oxabicyclo[5.2.1]deca-4,8-diene-2,2-dicarboxylate (3e)

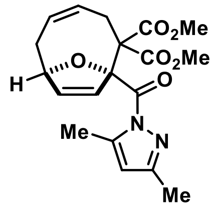

3e

Synthesized following modified General Procedure F using: (3,5-dimethyl-1H-pyrazol-1-yl)(furan-2-yl)methanone **1e** (38.0 mg, 0.2 mmol, 1.0 eq.), dimethyl 2-vinylcyclopropane-1,1-dicarboxylate **2a** (92.0 mg, 0.5 mmol, 2.5 eq.), Ir-F (4.0 mg, 0.004 mmol, 2 mol%) and MeCN (2 mL). The reaction was run for 48 h. From crude ^{1}H NMR analysis, a crude yield of 39% and 90:10 regioisomeric ratio was determined. Purification *via* column chromatography on silica gel (*n*-pentane/EtOAc 100:0 – 88:12) afforded the title compound **3e** (23.0 mg, 0.06 mmol, 31%) as a single as a white solid.

Note: The lower yield is attributed to inherently higher triplet energy of the pyrazole-substituted furan (>61 kcal/mol). The reaction proceeded slower and competitive decomposition of the starting material under the reaction conditions led to less product formation after full conversion.

TLC: R_f **=** 0.2 (88:12 *n*-pentane/EtOAc).

NMR Spectroscopy (*see spectra*):

^{1}H NMR (599 MHz, $CDCl_3$): δ_H 6.29 (dd, *J* = 6.0, 1.8 Hz, 1H), 5.89 (s, 1H), 5.85 – 5.81 (m, 1H), 5.81 – 5.73 (m, 1H), 5.57 – 5.54 (m, 1H), 5.52 – 5.46 (m, 1H), 3.83 (s, 3H), 3.69 (s, 3H), 2.98 – 2.92 (m, 1H), 2.80 –

2.73 (m, 1H), 2.61 – 2.56 (m, 1H), 2.48 (s, 3H), 2.39 – 2.30 (m, 1H), 2.17 (s, 3H) ppm;

^{13}C NMR (151 MHz, $CDCl_3$): δ_C 171.3, 170.8, 169.6, 151.4, 145.6, 133.9, 129.5, 128.4, 128.1, 110.3, 97.3, 87.5, 67.1, 52.8, 52.5, 33.2, 33.1, 14.5, 14.2 ppm.

HRMS (ESI$^+$): m/z calc'd for $C_{19}H_{22}N_2O_6Na$ [M+Na]$^+$: 397.1370, found 397.1377.

Dimethyl (*Z*)-1-benzoyl-10-oxabicyclo[5.2.1]deca-4,8-diene-2,2-dicarboxylate (3f)

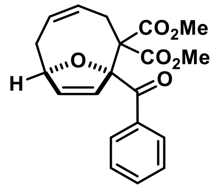

3f

Synthesized following General Procedure F using: furan-2-yl(phenyl)methanone **1f** (34.4 mg, 0.2 mmol, 1.0 eq.), dimethyl 2-vinylcyclopropane-1,1-dicarboxylate **2a** (92.0 mg, 0.5 mmol, 2.5 eq.), Ir-F (4.0 mg, 0.004 mmol, 2 mol%) and MeCN (2 mL). From crude ^{1}H NMR analysis, a crude yield of 50% and 90:10 regioisomeric ratio was determined. Purification *via* column chromatography on silica gel (*n*-pentane/EtOAc 100:0 – 91:9) afforded the title compound as a mixture of regioisomers **3f** (35.2 mg, 0.1 mmol, 49%, 90:10 r.r.) as a white solid.

TLC: R_f **=** 0.3 (90:10 *n*-pentane/EtOAc).

NMR Spectroscopy (*see spectra*):

^{1}H NMR (599 MHz, $CDCl_3$): δ_H 8.07 (dd, *J* = 8.2, 1.4 Hz, 0.18H, minor), 7.84 (dd, *J* = 8.2, 1.4 Hz, 2H, major), 7.57 – 7.53 (m, 0.08H, minor), 7.47 – 7.41 (m, 1H, major), 7.35 (t, *J* = 7.8 Hz, 2H), 6.13 (dd, *J* = 5.9, 1.6 Hz, 1H), 6.05 (d, *J* = 5.9 Hz, 1H), 5.88 – 5.80 (m, 1H), 5.63 – 5.56 (m, 1H), 5.32 – 5.27 (m, 1H), 3.85 (s, 3H, major), 3.82 (s, 0.3H, minor), 3.73 (s, 3H, major), 3.72 (s, 0.3H, minor), 2.74 – 2.66 (m, 2H), 2.61 – 2.56 (m, 0.08H, minor), 2.50 – 2.43 (m, 1H, major), 2.40 – 2.34 (m, 0.09H, minor), 2.34 – 2.25 (m, 1H, major) ppm;

^{13}C NMR (101 MHz, $CDCl_3$): δ_C 201.4, 170.8, 170.1, 137.3, 134.9, 131.8, 129.8, 129.3, 128.9, 128.4, 127.9, 101.3, 84.4, 68.0, 52.9, 52.7, 32.9, 32.2 ppm.

HRMS (ESI$^+$): m/z calc'd for $C_{20}H_{20}O_6Na$ [M+Na]$^+$: 379.1152, found 379.1143.

Dimethyl (*Z*)-1-(2-(furan-2-yl)-2-hydroxyacetyl)-10-oxabicyclo[5.2.1]deca-4,8-diene-2,2-dicarboxylate (3g) & dimethyl (*Z*)-7-(2-(furan-2-yl)-2-hydroxyacetyl)-10-oxabicyclo[5.2.1]deca-4,8-diene-2,2-dicarboxylate (3g')

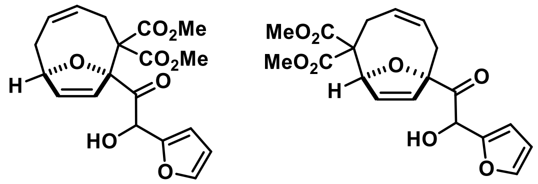

3g **3g'**

Synthesized following General Procedure F using: 2,2-furoin (38.4 mg, 0.2 mmol, 1.0 eq.), dimethyl 2-vinylcyclopropane-1,1-dicarboxylate **2a** (92.0 mg, 0.5 mmol, 2.5 eq.), Ir-F (4.0 mg, 0.004 mmol, 2 mol%) and MeCN (2 mL). From crude ^{1}H NMR analysis, a crude yield of 62% and 57:43 regioisomeric ratio was determined. Purification *via* column chromatography on silica gel (*n*-pentane/EtOAc 100:0 – 60:40) afforded the title compound **3g** (25.0 mg, 0.07 mmol, 33%, 50:50 d.r.) as a white solid, as well as the minor regioisomer **3g'** (19.2 mg, 0.05 mmol, 26%, 50:50 d.r.) as a colourless oil.

Characterization data for **3g**:

TLC: R_f **=** 0.3 (60:40 *n*-pentane/EtOAc).

NMR Spectroscopy (*see spectra*):

^{1}H NMR (599 MHz, $CDCl_3$): δ_H 7.38 (dd, *J* = 1.8, 0.9 Hz, 1H), 6.37 – 6.35 (m, 1H), 6.32 – 6.30 (m, 1H), 5.95 (dd, *J* = 6.0, 1.7 Hz, 1H), 5.88 (dd, *J* = 6.0, 1.6 Hz, 1H), 5.83 – 5.64 (m, 1H), 5.46 (d, *J* = 7.8 Hz, 1H), 5.45 – 5.41 (m, 1H), 5.35 – 5.32 (m, 1H), 3.82 (s, 3H), 3.62 (s, 3H), 2.79 – 2.74 (m, 1H), 2.71 – 2.65 (m, 1H), 2.49 – 2.44 (m, 1H), 2.29 – 2.24 (m, 1H) ppm;

^{13}C NMR (151 MHz, $CDCl_3$): δ_C 204.2, 171.2, 169.3, 151.5, 142.5, 135.1, 129.2, 128.7, 128.1, 110.8, 108.2, 98.9, 87.5, 69.0, 66.2, 52.8, 52.8, 33.2, 32.2 ppm.

Characterization data for **3g'**:

TLC: R_f **=** 0.4 (60:40 *n*-pentane/EtOAc).

NMR Spectroscopy (*see spectra*):

^{1}H NMR (599 MHz, $CDCl_3$): δ_H 7.26 – 7.25 (m, 1H), 6.29 (dd, *J* = 3.2, 1.8 Hz, 1H), 6.27 (dd, *J* = 3.3, 0.9 Hz, 1H), 5.92 (d, *J* = 6.2 Hz, 1H), 5.83 – 5.75 (m, 1H), 5.70 (dd, *J* = 5.9, 1.6 Hz, 1H), 5.65 (d, *J* = 6.0 Hz, 1H), 5.62 – 5.55 (m, 1H), 4.70 – 4.65 (m, 1H), 3.82 (s, 3H), 3.80 (s, 3H), 2.58 – 2.49 (m, 2H), 2.34 – 2.27 (m, 1H), 2.12 – 2.05 (m, 1H) ppm;

^{13}C NMR (151 MHz, $CDCl_3$): δ_C 206.8, 170.5, 169.6, 150.5, 142.4, 133.9, 129.6, 128.4, 125.6, 110.8, 109.4, 98.6, 83.6, 69.7, 68.6, 53.0, 53.0, 32.4, 31.4 ppm.

HRMS (ESI$^+$): m/z calc'd for $C_{19}H_{20}O_8Na$ $[M+Na]^+$: 399.1050, found 399.1047.

2-(2-((*Z*)-2,2-Bis(methoxycarbonyl)-10-oxabicyclo[5.2.1]deca-4,8-dien-1-yl)-2-oxoethyl) 1-(*tert*-butyl) (2*S*)-pyrrolidine-1,2-dicarboxylate (3h) & 2-(2-((*Z*)-6,6-bis(methoxycarbonyl)-10-oxabicyclo[5.2.1]deca-3,8-dien-1-yl)-2-oxoethyl) 1-(*tert*-butyl) (2*S*)-pyrrolidine-1,2-dicarboxylate (3h')

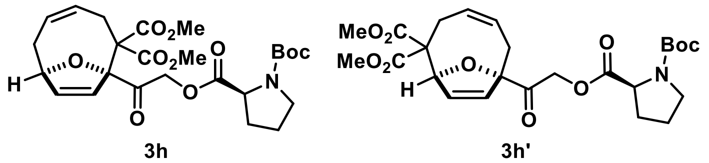

3h **3h'**

Synthesized following General Procedure F using: 1-(*tert*-butyl) 2-(2-(furan-2-yl)-2-oxoethyl) (*S*)-pyrrolidine-1,2-dicarboxylate **1h** (64.6 mg, 0.2 mmol, 1.0 eq.), dimethyl 2-vinylcyclopropane-1,1-dicarboxylate **2a** (92.0 mg, 0.5 mmol, 2.5 eq.), Ir-F (4.0 mg, 0.004 mmol, 2 mol%) and MeCN (2 mL). From crude ^{1}H NMR analysis, a crude

yield of 57% and 86:14 regioisomeric ratio was determined. Purification *via* column chromatography on silica gel (*n*-pentane/EtOAc 100:0 – 75:25) afforded the title compound **3h** (49.5 mg, 0.10 mmol, 49%, 44:56 d.r.) as a white solid, as well as the minor regioisomer **3h'** (10.2 mg, 0.02 mmol, 10%, 44:56 d.r.) as a colourless oil.

Characterization data for **3h**:

TLC: R_f **=** 0.3 (75:25 *n*-pentane/EtOAc).

NMR Spectroscopy (***see spectra***):

^{1}**H NMR** (400 MHz, $CDCl_3$): δ_H 6.11 – 5.98 (m, 1H), 5.93 (dd, *J* = 5.9, 1.7 Hz, 1H), 5.87 – 5.74 (m, 1H), 5.63 – 5.51 (m, 1H), 5.36 – 5.26 (m, 1H), 5.24 – 5.14 (m, 0.6H, d_1), 5.12 – 4.98 (m, 0.9H, d_2), 4.98 – 4.86 (m, 0.6H, d_1), 4.41 – 4.33 (m, 0.4H, d_2), 4.33 – 4.27 (m, , 0.6H, d_1), 3.79 – 3.76 (m, 3H), 3.76 – 3.73 (m, 3H), 3.60 – 3.28 (m, 2H), 2.73 – 2.57 (m, 2H), 2.45 – 2.33 (m, 1H), 2.31 – 2.10 (m, 3H), 2.04 – 1.90 (m, 1H), 1.88 – 1.77 (m, 1H), 1.43 (d, *J* = 3.0 Hz, 3H), 1.39 (d, *J* = 1.8 Hz, 6H) ppm;

^{13}**C NMR** (101 MHz, $CDCl_3$): δ_C 202.0 and 201.8, 172.8 and 172.6, 170.7 and 170.6, 169.4, 154.0 and 154.0, 135.1, 129.6 and 129.5, 128.6 and 128.4, 127.0 and 127.0, 99.0 and 98.9, 85.0, 80.0 and 79.8, 67.2 and 67.2, 66.0 and 65.6, 59.2 and 59.0 and 58.6, 52.9 and 52.9, 46.8 and 46.4, 33.0 and 32.9, 31.9 and 31.8, 31.1, 30.2, 28.6, 28.4, 24.4 and 24.4, 23.7 and 23.6 ppm.

Characterization data for **3h'**:

TLC: R_f **=** 0.4 (75:25 *n*-pentane/EtOAc).

NMR Spectroscopy (***see spectra***):

^{1}**H NMR** (400 MHz, $CDCl_3$): δ_H 5.99 (d, *J* = 9.4 Hz, 1H), 5.92 – 5.84 (m, 1H), 5.77 – 5.67 (m, 1H), 5.56 – 5.49 (m, 1H), 5.47 – 5.39 (m, 1H), 5.36 – 5.20 (m, 0.5H, d_1), 5.18 – 5.04 (m, 1.1H, d_2), 5.03 – 4.91 (m, 0.5H, d_1), 4.46 – 4.40 (m, 0.5H, d_1), 4.38 – 4.30 (m, 0.6H, d_2), 3.79 (d, *J* = 1.4 Hz, 3H), 3.77 (d, *J* = 1.4 Hz, 3H), 3.60 – 3.35 (m, 2H), 2.78 – 2.54 (m, 4H), 2.38 – 2.20 (m, 2H), 2.08 – 1.96 (m, 1H), 1.94 – 1.86 (m, 1H), 1.45 (d, *J* = 3.7 Hz, 3H), 1.42 (q, *J* = 3.1 Hz, 6H) ppm;

^{13}**C NMR** (101 MHz, $CDCl_3$): δ_C 202.0 and 201.8, 172.8 and 172.6, 170.6, 169.4, 154.0 and 154.0, 135.1, 129.6, 128.6 and 128.4, 127.0, 99.0, 84.9, 80.0 and 79.9, 67.2, 66.0 and 65.6, 59.2 and 59.0 and 58.6, 52.9 and 52.8, 46.8 and 46.4, 33.0, 31.8, 31.1, 30.2, 28.6, 28.4, 24.4, 23.7 and 23.6 ppm.

HRMS (ESI^+): m/z calc'd for $C_{25}H_{33}NO_{10}Na$ $[M+Na]^+$: 530.1997, found 530.1996.

Dimethyl (*Z*)-1-cyano-10-oxabicyclo[5.2.1]deca-4,8-diene-2,2-dicarboxylate (3i) & dimethyl (*Z*)-7-cyano-10-oxabicyclo[5.2.1]deca-4,8-diene-2,2-dicarboxylate (3i')

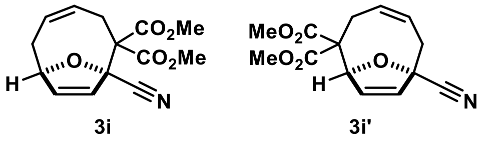

3i **3i'**

Synthesized following modified General Procedure F using: furan-2-carbonitrile **1i** (17.5 µL, 0.2 mmol, 1.0 eq.), dimethyl 2-vinylcyclopropane-1,1-dicarboxylate **2a** (92.0 mg, 0.5 mmol, 2.5 eq.), 3-OMe-thioxanthone (4.8 mg,

0.02 mmol, 10 mol%) and MeCN (2 mL). From crude ^{1}H NMR analysis, a crude yield of 52% and 63:37 regioisomeric ratio was determined. Purification *via* column chromatography on silica gel (*n*-pentane/EtOAc 100:0 – 89:11) afforded the title compound **3i** (17.2 mg, 0.062 mmol, 31%) as colourless oil, as well as the minor regioisomer **3i'** (11.1 mg, 0.041 mmol, 20%) as a colourless oil.

Characterization data for **3i**:

TLC: R_f **=** 0.3 (89:11 *n*-pentane/EtOAc).

NMR Spectroscopy (*see spectra*):

^{1}**H NMR** (400 MHz, $CDCl_3$): δ_H 5.89 – 5.87 (m, 1H), 5.86 – 5.79 (m, 1H), 5.78 (dd, *J* = 5.9, 1.6 Hz, 1H), 5.55 – 5.52 (m, 1H), 5.41 – 5.32 (m, 1H), 3.87 (s, 3H), 3.86 (s, 3H), 2.92 – 2.84 (m, 1H), 2.78 – 2.70 (m, 1H), 2.63 – 2.56 (m, 1H), 2.38 – 2.28 (m, 1H) ppm;

^{13}**C NMR** (101 MHz, $CDCl_3$): δ_C 170.2, 167.9, 134.7, 130.3, 127.4, 126.0, 118.3, 89.6, 84.8, 66.9, 53.7, 53.1, 33.3, 30.6 ppm.

Characterization data for **3i'**:

TLC: R_f **=** 0.4 (89:11 *n*-pentane/EtOAc).

NMR Spectroscopy (*see spectra*):

^{1}**H NMR** (400 MHz, $CDCl_3$): δ_H 6.08 – 5.98 (m, 2H), 5.68 (t, *J* = 1.6 Hz, 1H), 5.58 – 5.54 (m, 1H), 5.35 (ddd, *J* = 10.9, 9.0, 6.4 Hz, 1H), 3.78 (s, 3H), 3.77 (s, 3H), 3.04 – 2.96 (m, 1H), 2.69 – 2.63 (m, 3H) ppm;

^{13}**C NMR** (101 MHz, $CDCl_3$): δ_C 170.9, 168.7, 131.2, 130.4, 128.1, 127.5, 118.6, 86.8, 86.0, 62.4, 53.5, 52.8, 38.8, 29.1 ppm;

HRMS (ESI$^+$): m/z calc'd for $C_{14}H_{15}NO_5Na$ $[M+Na]^+$: 300.0842, found 300.0842.

Trimethyl (*Z*)-10-oxabicyclo[5.2.1]deca-4,8-diene-1,2,2-tricarboxylate (3j) & trimethyl (*Z*)-10-oxabicyclo[5.2.1]deca-3,8-diene-1,6,6-tricarboxylate (3j')

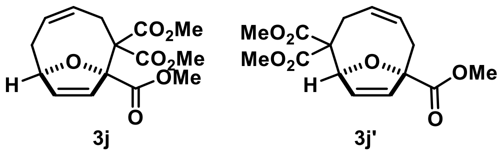

3j 3j'

Synthesized following modified General Procedure F using: methyl furan-2-carboxylate **1j** (21.4 µL, 0.2 mmol, 1.0 eq.), dimethyl 2-vinylcyclopropane-1,1-dicarboxylate **2a** (92.0 mg, 0.5 mmol, 2.5 eq.), 3-OMe-thioxanthone (4.8 mg, 0.02 mmol, 10 mol%) and MeCN (2 mL). From crude ^{1}H NMR analysis, a crude yield of 48% and 63:37 regioisomeric ratio was determined. Purification *via* column chromatography on silica gel (*n*-pentane/EtOAc 100:0 – 78:22) afforded the title compound **3j** (10.7 mg, 0.035 mmol, 17%) as colourless oil, as well **3j'** (20.5 mg, 0.066 mmol, 31%) as a colourless oil.

Note: In this case, the C2-addition regioisomer **3j'** was observed as the major product.

Characterization data for **3j**:

TLC: R_f **=** 0.3 (80:20 *n*-pentane/EtOAc).

NMR Spectroscopy (*see spectra*):

^{1}**H NMR** (400 MHz, $CDCl_3$): δ_H 6.07 (dd, *J* = 5.9, 1.6 Hz, 1H), 5.95 – 5.87 (m, 1H), 5.84 – 5.72 (m, 1H), 5.55 – 5.45 (m, 1H), 5.40 – 5.31 (m, 1H), 3.80 (s, 3H), 3.76 (s, 3H), 3.75 (s, 3H), 2.79 – 2.63 (m, 2H), 2.49 – 2.41 (m, 1H), 2.28 – 2.19 (m, 1H) ppm;

^{13}**C NMR** (101 MHz, $CDCl_3$): δ_C 171.9, 170.7, 169.6, 135.8, 134.0, 128.9, 127.4, 94.7, 89.1, 52.9, 52.9, 52.8, 33.1, 32.0 ppm.

Characterization data for **3j'**:

TLC: R_f **=** 0.4 (80:20 *n*-pentane/EtOAc).

NMR Spectroscopy (*see spectra*):

^{1}**H NMR** (400 MHz, $CDCl_3$): δ_H 6.05 – 5.93 (m, 1H), 5.84 (dd, *J* = 6.1, 1.8 Hz, 1H), 5.68 (t, *J* = 2.1 Hz, 1H), 5.63 (dd, *J* = 6.0, 2.3 Hz, 1H), 5.46 – 5.36 (m, 1H), 3.78 (s, 3H), 3.76 (s, 3H), 3.75 (s, 3H), 2.79 – 2.57 (m, 4H) ppm;

^{13}**C NMR** (101 MHz, $CDCl_3$): δ_C 172.4, 171.1, 169.2, 130.6, 129.4, 129.3, 128.9, 95.7, 86.4, 62.9, 53.3, 52.8, 52.7, 36.8, 29.0 ppm.

HRMS (ESI^+): m/z calc'd for $C_{15}H_{18}O_7Na$ $[M+Na]^+$: 333.0945, found 333.0942.

Dimethyl (*Z*)-1-(2,2,2-trifluoroacetyl)-10-oxabicyclo[5.2.1]deca-4,8-diene-2,2-dicarboxylate (3k)

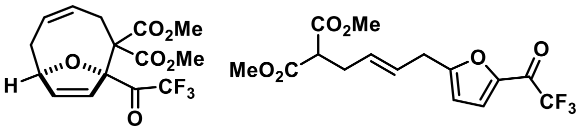

3k proposed strukture **3k'**

Synthesized following General Procedure F using: 2,2,2-trifluoro-1-(furan-2-yl)ethan-1-one **1k** (29.2 mg, 0.2 mmol, 1.0 eq.), dimethyl 2-vinylcyclopropane-1,1-dicarboxylate **2a** (92.0 mg, 0.5 mmol, 2.5 eq.), Ir-F (4.0 mg, 0.004 mmol, 2 mol%) and MeCN (2 mL). From crude ^{1}H NMR analysis, a crude yield of 32% and >95:5 regioisomeric ratio was determined. Purification *via* column chromatography on silica gel (*n*-pentane/EtOAc 100:0 –93:7) afforded the title compound **3k** (30.8 mg, **3k**:**3k'** 2:1; corrected: 20.5 mg,0.06 mmol, 30%) as a colourless solid.

Note: The product was obtained as an inseparable mixture and contains a the linear cycloadduct (ratio **3k**/**3k'** 2:1). Assignment of the peaks was conducted *via* 2D-NMR. **3k'** is formed from the same intermediate, however after radical addition and cyclopropane opening, quenching via HAT and rearomatization of the furan appears to be a competing pathway for the CF_3-containing furan.

Characterization data for **3k** and **3k'**:

TLC: R_f **=** 0.3 (80:20 *n*-pentane/EtOAc).

NMR Spectroscopy (*see spectra*):

^{1}H NMR (500 MHz, $CDCl_3$): δ_H 7.44 – 7.43 (m, 0.67H, **3k'**), 6.71 (d, *J* = 3.4 Hz, 0.67H, **3k'**), 6.39 – 6.37 (m, 0.67H, **3k'**), 6.07 (d, *J* = 5.9 Hz, 1H, **3k**), 5.90 (dd, *J* = 5.9, 1.6 Hz, 1H, **3k**), 5.88 – 5.80 (m, 1H, **3k**), 5.61 – 5.52 (m, 1H, **3k**), 5.38 – 5.35 (m, 1H, **3k**), 5.34 – 5.30 (m, 0.67H, **3k'**), 5.21 – 5.06 (m, 1.35H, **3k'**), 3.85 (s, 2H, **3k'**), 3.84 (s, 2H, **3k'**), 3.82 (s, 3H, **3k**), 3.78 (s, 3H, **3k**), 3.58 – 3.51 (m, 0.67H, **3k'**), 2.96 – 2.90 (m, 0.67H, **3k'**), 2.76 – 2.69 (m, 2H, **3k**), 2.50 – 2.48 (m, 0.67H, **3k'**), 2.47 – 2.40 (m, 1H, **3k**), 2.30 – 2.23 (m, 1H, **3k**) ppm;

^{13}C NMR{^{19}F} (126 MHz, $CDCl_3$): δ_C 188.6, 170.5 (**3k**), 169.0 (**3k**), 168.5 (**3k'**), 168.4 (**3k'**), 147.2 (**3k'**), 143.7 (**3k'**), 136.6 (**3k**), 132.4 (**3k'**), 128.9 (**3k**), 128.8 (**3k**), 125.7 (**3k**), 124.1 (**3k'**), 119.2 (**3k'**), 116.4 (**3k**), 111.4 (**3k'**), 110.5 (**3k'**), 97.7 (**3k'**), 87.1 (**3k**), 87.0 (**3k'**), 86.3 (**3k')** 67.6 (**3k**), 53.5 (**3k'**), 53.4 (**3k'**), 53.3 (**3k**), 53.1 (**3k**), 48.7 (**3k'**), 38.1 (**3k'**), 32.8 (**3k**), 31.6 (**3k**) ppm;

^{13}C NMR (126 MHz, $CDCl_3$): δ_C 188.6, 170.5 (**3k**), 169.0 (**3k**), 168.5 (**3k'**), 168.4 (**3k'**), 147.2 (**3k'**), 143.7 (**3k'**), 136.6 (**3k**), 132.4 (**3k'**), 128.9 (**3k**), 128.8 (**3k**), 125.7 (**3k**), 124.1 (q, *J* = 285.2 Hz) (**3k'**), 119.2 (**3k'**), 116.4 (q, *J* = 294.3 Hz) (**3k**), 111.4 (**3k'**), 110.5 (**3k'**), 97.7 (**3k'**), 87.1 (**3k**), 87.0 (**3k'**), 86.3 (**3k')** 67.6 (**3k**), 53.5 (**3k'**), 53.4 (**3k'**), 53.3 (**3k**), 53.1 (**3k**), 48.7 (**3k'**), 38.1 (**3k'**), 32.8 (**3k**), 31.6 (**3k**) ppm;

^{19}F NMR (376 MHz, $CDCl_3$): δ_F -72.8 (**3k**), -76.7 (**3k'**) ppm;

HRMS (ESI$^+$): m/z calc'd for $C_{15}H_{15}O_6F_3Na$ $[M+Na]^+$: 371.1723, found 371.0712.

Trimethyl (*Z*)-7-phenyl-10-oxabicyclo[5.2.1]deca-3,8-diene-1,6,6-tricarboxylate

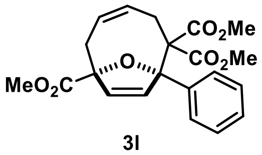

3l

Synthesized following modified General Procedure F using: methyl 5-phenylfuran-2-carboxylate **1l** (40.4 mg, 0.2 mmol, 1.0 eq.), dimethyl 2-vinylcyclopropane-1,1-dicarboxylate **2a** (92.0 mg, 0.5 mmol, 2.5 eq.), Ir-F (4.0 mg, 0.004 mmol, 2 mol%) and MeCN (2 mL). From crude ^{1}H NMR analysis, a crude yield of 65% and >95:5 regioisomeric ratio was determined. Purification *via* column chromatography on silica gel (*n*-pentane/EtOAc 100:0 – 70:30) afforded the title compound **3l** (48.0 mg, 0.124 mmol, 62%) as an off-white solid.

TLC: R_f **=** 0.5 (80:20 *n*-pentane/EtOAc).

NMR Spectroscopy (*see spectra*):

^{1}H NMR (400 MHz, $CDCl_3$): δ_H 7.86 – 7.76 (m, 2H), 7.31 – 7.23 (m, 2H), 7.22 – 7.13 (m, 1H), 6.81 (d, *J* = 5.9 Hz, 1H), 5.78 – 5.65 (m, 1H), 5.53 (ddd, *J* = 11.9, 9.4, 5.9 Hz, 1H), 5.48 (d, *J* = 5.9 Hz, 1H), 3.82 (s, 3H), 3.73 (s, 3H), 3.48 (ddd, *J* = 13.1, 10.1, 1.1 Hz, 1H), 3.07 (s, 3H), 3.01 – 2.91 (m, 1H), 2.76 (dd, *J* = 14.6, 8.9 Hz, 1H), 2.65 (dd, *J* = 13.5, 6.8 Hz, 1H) ppm;

^{13}C NMR (101 MHz, $CDCl_3$): δ_C 172.3, 170.5, 170.4, 142.9, 134.9, 130.1, 128.6, 127.7, 127.2, 126.4, 126.3, 95.6, 94.6, 69.7, 52.6, 52.6, 52.1, 36.5, 32.6 ppm.

HRMS (ESI$^+$): m/z calc'd for $C_{21}H_{22}O_7Na$ $[M+Na]^+$: 409.1258, found 409.1249.

Trimethyl (*Z*)-7-(thiophen-3-yl)-10-oxabicyclo[5.2.1]deca-3,8-diene-1,6,6-tricarboxylate

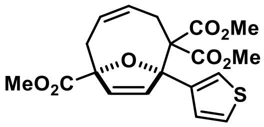

3m

Synthesized following General Procedure F using: methyl 5-(thiophen-3-yl)furan-2-carboxylate **1m** (41.6 mg, 0.2 mmol, 1.0 eq.), dimethyl 2-vinylcyclopropane-1,1-dicarboxylate **2a** (92.0 mg, 0.5 mmol, 2.5 eq.), Ir-F (4.0 mg, 0.004 mmol, 2 mol%) and MeCN (2 mL). The reaction was run for 96 h. From crude ^{1}H NMR analysis, a crude yield of 32% and >95:5 regioisomeric ratio was determined. Purification *via* column chromatography on silica gel (*n*-pentane/EtOAc 100:0 –91:9) afforded the title compound **3m** (23.8 mg,0.06 mmol, 30%) as a colourless oil.

Note: After 96 h, 25% of starting material was remaining.

Characterization data for **3m**:

TLC: R_f **=** 0.4 (91:9 *n*-pentane/EtOAc).

NMR Spectroscopy (*see spectra*):

^{1}**H NMR** (400 MHz, $CDCl_3$): δ_H 7.58 – 7.52 (m, 1H), 7.44 – 7.37 (m, 1H), 7.18 – 7.11 (m, 1H), 6.62 – 6.55 (m, 1H), 5.80 – 5.67 (m, 1H), 5.57 – 5.47 (m, 1H), 5.47 – 5.43 (m, 1H), 3.84 (s, 3H), 3.76 (s, 3H), 3.45 – 3.34 (m, 1H), 3.24 (s, 3H), 2.98 – 2.88 (m, 1H), 2.83 – 2.71 (m, 1H), 2.65 (dd, *J* = 13.2, 7.0 Hz, 1H) ppm;

^{13}**C NMR** (126 MHz, $CDCl_3$): δ_C 172.3, 170.8, 170.3, 144.5, 134.6, 130.1, 128.7, 127.1, 126.5, 124.3, 122.1, 95.8, 94.0, 69.2, 52.6, 52.6, 52.4, 36.5, 32.1 ppm.

HRMS (ESI$^+$): m/z calc'd for $C_{19}H_{20}O_7SNa$ $[M+Na]^+$: 415.0822, found 415.0825.

Dimethyl (*Z*)-7-methyl-1-(3-phenylpropanoyl)-10-oxabicyclo[5.2.1]deca-4,8-diene-2,2-dicarboxylate (3n)

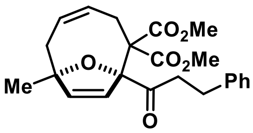

3n

Synthesized following General Procedure F using: 1-(5-methylfuran-2-yl)-3-phenylpropan-1-one **1n** (42.8 mg, 0.2 mmol, 1.0 eq.), dimethyl 2-vinylcyclopropane-1,1-dicarboxylate **2a** (92.0 mg, 0.5 mmol, 2.5 eq.), Ir-F (4.0 mg, 0.004 mmol, 2 mol%) and MeCN (2 mL). From crude ^{1}H NMR analysis, a crude yield of 40% and >95:5 regioisomeric ratio was determined. Purification *via* column chromatography on silica gel (*n*-pentane/EtOAc 100:0 – 90:10) afforded the title compound **3n** (31.2 mg, 0.08 mmol, 39%) as a single regioisomer as a colourless oil.

Note: No starting material remained, the lower yield is attributed to steric hinderance at the C5-position and thus slower product formation, while competing decomposition of the starting material under the reaction conditions takes place.

TLC: R_f **=** 0.3 (90:10 *n*-pentane/EtOAc).

NMR Spectroscopy (*see spectra*):

^{1}H NMR (599 MHz, $CDCl_3$): δ_H 7.28 – 7.23 (m, 2H), 7.22 – 7.13 (m, 3H), 5.88 (d, *J* = 5.8 Hz, 1H), 5.84 – 5.76 (m, 2H), 5.66 – 5.59 (m, 1H), 3.81 (s, 3H), 3.72 (s, 3H), 3.33 – 3.23 (m, 1H), 2.92 – 2.72 (m, 3H), 2.68 – 2.60 (m, 1H), 2.51 – 2.40 (m, 2H), 2.37 – 2.30 (m, 1H), 1.54 (s, 3H) ppm;

^{13}C NMR (151 MHz, $CDCl_3$): δ_C 210.2, 171.0, 170.0, 141.8, 138.6, 129.6, 128.5, 128.4, 126.8, 126.2, 125.9, 100.0, 91.9, 67.0, 52.8, 52.7, 39.9, 38.2, 32.0, 29.5, 28.1 ppm.

HRMS (ESI$^+$): m/z calc'd for $C_{23}H_{26}O_6Na$ $[M+Na]^+$: 421.1622, found 421.1622.

Dimethyl (*Z*)-1-acetyl-9-methyl-10-oxabicyclo[5.2.1]deca-4,8-diene-2,2-dicarboxylate (3o) & dimethyl (*Z*)-7-acetyl-8-methyl-10-oxabicyclo[5.2.1]deca-4,8-diene-2,2-dicarboxylate (3o')

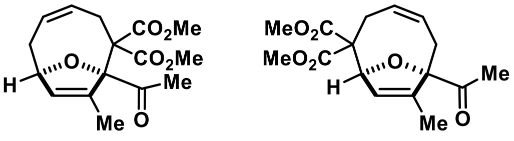

3o **3o'**

Synthesized following General Procedure F using: 1-(3-methylfuran-2-yl)ethan-1-one **1o** (24.8 mg, 0.2 mmol, 1.0 eq.), dimethyl 2-vinylcyclopropane-1,1-dicarboxylate **2a** (92.0 mg, 0.5 mmol, 2.5 eq.), Ir-F (4.0 mg, 0.004 mmol, 2 mol%) and MeCN (2 mL). From crude ^{1}H NMR analysis, a crude yield of 60% and 75:25 regioisomeric ratio was determined. Purification *via* column chromatography on silica gel (*n*-pentane/EtOAc 100:0 – 89:11) afforded the title compound were employed to obtain the title compound **3o** (23.9 mg, 0.08 mmol, 39%) as a colourless oil, as well as the minor regioisomer **3o'** (9.2 mg, 0.03 mmol, 15%) as a colourless oil.

Characterization data for **3o**:

TLC: R_f **=** 0.3 (89:11 *n*-pentane/EtOAc).

NMR Spectroscopy (*see spectra*):

^{1}H NMR (400 MHz, $CDCl_3$): δ_H 5.95 – 5.83 (m, 1H), 5.80 – 5.74 (m, 1H), 5.62 – 5.51 (m, 1H), 5.21 – 5.12 (m, 1H), 3.76 (s, 3H), 3.74 (s, 3H), 2.70 – 2.57 (m, 2H), 2.50 – 2.40 (m, 1H), 2.29 – 2.16 (m, 4H), 1.67 (t, *J* = 1.6 Hz, 3H) ppm;

^{13}C NMR (151 MHz, $CDCl_3$): δ_C 207.9, 171.2, 170.1, 134.7, 132.9, 129.7, 128.3, 99.7, 82.4, 67.3, 52.7, 52.6, 33.0, 32.7, 25.3, 13.0 ppm.

Characterization data for **3o'**:

TLC: R_f **=** 0.4 (89:11 *n*-pentane/EtOAc).

NMR Spectroscopy (*see spectra*):

^{1}H NMR (400 MHz, $CDCl_3$): δ_H 6.05 – 5.93 (m, 1H), 5.67 (t, *J* = 1.9 Hz, 1H), 5.57 – 5.49 (m, 1H), 5.46 – 5.43 (m, 1H), 3.77 (s, 3H), 3.77 (s, 3H), 2.87 – 2.76 (m, 1H), 2.73 – 2.54 (m, 2H), 2.46 – 2.37 (m, 1H), 2.26

(s, 3H), 1.42 (t, J = 1.8 Hz, 3H) ppm;

^{13}C NMR (151 MHz, $CDCl_3$): δ_C 209.4, 171.6, 169.4, 139.3, 129.9, 128.4, 124.0, 102.0, 85.5, 65.2, 53.2, 52.6, 32.8, 29.3, 25.8, 11.6 ppm.

HRMS (ESI$^+$): m/z calc'd for $C_{16}H_{20}O_6Na$ [M+Na]$^+$: 331.1152, found 331.1161.

Dimethyl (*Z*)-8-bromo-1-propionyl-10-oxabicyclo[5.2.1]deca-4,8-diene-2,2-dicarboxylate (3p)

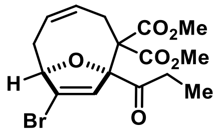

3p

Synthesized following General Procedure F using: 1-(4-bromofuran-2-yl)propan-1-one **1p** (40.6 mg, 0.2 mmol, 1.0 eq.), dimethyl 2-vinylcyclopropane-1,1-dicarboxylate **2a** (92.0 mg, 0.5 mmol, 2.5 eq.), Ir-F (4.0 mg, 0.004 mmol, 2 mol%) and MeCN (2 mL). From crude ^{1}H NMR analysis, a crude yield of 62% and 90:10 regioisomeric ratio was determined. Purification *via* column chromatography on silica gel (*n*-pentane/EtOAc 100:0 – 94:6) afforded the title compound **3p** (34.6 mg, 0.09 mmol, 45%) as a single regioisomer as a colourless oil.

TLC: R_f **=** 0.2 (94:6 *n*-pentane/EtOAc).

NMR Spectroscopy (*see spectra*):

^{1}H NMR (400 MHz, $CDCl_3$)*:* δ_H 6.04 (d, J = 1.4 Hz, 1H), 5.91 – 5.78 (m, 1H), 5.70 – 5.59 (m, 1H), 5.19 – 5.12 (m, 1H), 3.80 (s, 3H), 3.72 (s, 3H), 2.92 – 2.77 (m, 1H), 2.74 – 2.61 (m, 2H), 2.56 – 2.40 (m, 3H), 1.02 (t, J = 7.2 Hz, 3H) ppm;

^{13}C NMR (101 MHz, $CDCl_3$): δ_C 209.6, 170.6, 169.5, 129.9, 127.8, 127.7, 123.6, 99.3, 87.4, 68.1, 52.9, 52.8, 32.4, 32.0, 29.9, 7.5 ppm.

HRMS (ESI$^+$): m/z calc'd for $C_{16}H_{19}BrO_6Na$ [M+Na]$^+$: 409.0257 and 411.0239, found 409.0268 and 411.0246.

8-Ethyl 2,2-dimethyl (*Z*)-1-acetyl-10-oxabicyclo[5.2.1]deca-4,8-diene-2,2,8-tricarboxylate (3q)

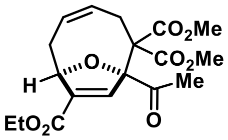

3q

Synthesized following modified General Procedure F using: ethyl 5-acetylfuran-3-carboxylate **1q** (24.8 mg, 0.2 mmol, 1.0 eq.), dimethyl 2-vinylcyclopropane-1,1-dicarboxylate **2a** (92.0 mg, 0.5 mmol, 2.5 eq.), Ir-F (4.0 mg, 0.004 mmol, 2 mol%) and MeCN (2 mL). The reaction was run for 48 h. From crude ^{1}H NMR analysis, a crude yield of 47% and 87:13 regioisomeric ratio was determined. Purification *via* column chromatography on silica gel (*n*-pentane/EtOAc 100:0 – 88:12) afforded the title compound as a mixture of regioisomers **3q** (34.2 mg,

0.09 mmol, 47%, 93:7 r.r.) as a white solid.

TLC: R_f **=** 0.3 (88:12 *n*-pentane/EtOAc).

NMR Spectroscopy (*see spectra*):

> **^{1}H NMR** (400 MHz, $CDCl_3$): δ_H 6.72 (d, J = 1.4 Hz, 1H, major), 6.52 (d, J = 1.0 Hz, 0.07H, minor), 6.06 (d, J = 1.8 Hz, 0.07H, minor), 5.97 (d, J = 9.6 Hz, 0.08H, minor), 5.87 – 5.75 (m, 1H, major), 5.62 – 5.49 (m, 1H), 5.51 – 5.46 (m, 1H, major), 4.32 – 4.08 (m, 2H), 3.81 (s, 3H), 3.74 (s, 3H, major), 3.68 (s, 0.3H, minor), 2.82 – 2.68 (m, 2H), 2.57 – 2.42 (m, 2H), 2.25 (s, 0.3H, minor), 2.23 (s, 3H, major), 1.31 (t, J = 7.2 Hz, 3H) ppm;

> **^{13}C NMR** (101 MHz, $CDCl_3$): δ_C 207.3, 170.8, 169.4, 161.7, 138.9, 137.6, 129.4, 129.2, 99.9, 84.8, 67.7, 61.3, 53.0, 52.9, 33.5, 32.6, 25.2, 14.2 ppm.

HRMS (ESI^+): m/z calc'd for $C_{18}H_{22}O_8Na$ $[M+Na]^+$: 389.1207, found 389.1207.

Trimethyl (*Z*)-7-phenyl-10-oxa-8-azabicyclo[5.2.1]deca-3,8-diene-1,6,6-tricarboxylate (3r) & Trimethyl 2-phenyl-5,8-dihydro-4H-cyclohepta[d]oxazole-4,4,8a(3aH)-tricarboxylate (3r')

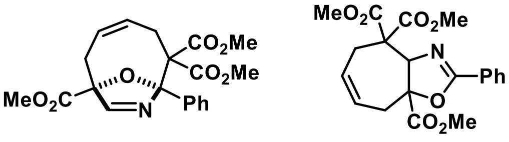

3r 3r'

Synthesized following General Procedure F using: methyl 2-phenyloxazole-5-carboxylate **1r** (40.6 mg, 0.2 mmol, 1.0 eq.), dimethyl 2-vinylcyclopropane-1,1-dicarboxylate **2a** (73.7 mg, 0.4 mmol, 2.0 eq.), Ir-F (4.0 mg, 0.004 mmol, 2 mol%) and MeCN (4 mL, 0.05 M). From crude ^{1}H NMR analysis, a crude yield of 55% was determined for the major (5+4) regioisomer **3r** and 10% for the minor (5+2) regioisomer **3r'**. Purification *via* column chromatography on silica gel (*n*-pentane/EtOAc 100:0 – 88:12) afforded **3r** (41.1 mg, 0.1 mmol, 53%) and **3r'** (5.4 mg, 0.01 mmol, 7%) as a yellowish oil.

Characterization data for **3r**:

TLC: R_f **=** 0.3 (80:20 *n*-pentane/EtOAc).

NMR Spectroscopy (***see spectra***):

> **^{1}H NMR** (400 MHz, $CDCl_3$): δ_H 7.92 – 7.81 (m, 2H), 7.32 – 7.27 (m, 2H), 7.25 – 7.19 (m, 2H), 5.97 – 5.81 (m, 1H), 5.71 – 5.59 (m, 1H), 3.81 (s, 3H), 3.68 (s, 3H), 3.40 – 3.22 (m, 4H), 2.98 (ddd, J = 15.3, 6.2, 2.2 Hz, 1H), 2.86 (dd, J = 15.3, 8.6 Hz, 1H), 2.64 (dd, J = 13.3, 7.4 Hz, 1H) ppm;

> **^{13}C NMR** (101 MHz, $CDCl_3$): δ_C 169.6, 169.2, 168.4, 160.2, 141.0, 131.2, 128.3, 128.1, 128.0, 127.1, 114.4, 97.6, 68.1, 52.9, 52.5, 52.5, 35.2, 32.5 ppm.

Characterization data for **3r'**:

TLC: R_f **=** 0.3 (80:20 *n*-pentane/EtOAc).

NMR Spectroscopy (***see spectra***):

^{1}H NMR (400 MHz, $CDCl_3$): δ_H 8.02 – 7.96 (m, 2H), 7.54 – 7.48 (m, 1H), 7.45 – 7.38 (m, 2H), 5.58 – 5.50 (m, 2H), 5.43 – 5.35 (m, 1H), 3.83 (s, 3H), 3.82 (s, 3H), 3.77 (s, 3H), 3.11 (d, *J* = 18.3 Hz, 1H), 2.90 (dd, *J* = 18.3, 6.9 Hz, 1H), 2.81 (dd, *J* = 18.4, 7.3 Hz, 1H), 2.61 (dd, *J* = 18.3, 3.0 Hz, 1H) ppm;

^{13}C NMR (101 MHz, $CDCl_3$): δ_C 172.2, 170.3, 169.0, 164.9, 132.3, 129.0, 128.5, 127.2, 126.7, 123.3, 89.4, 73.7, 60.5, 53.5, 53.4, 53.1, 31.8, 29.3 ppm.

HRMS (ESI$^+$): m/z calc'd for $C_{20}H_{21}NO_7Na$ [M+Na]$^+$: 410.1210, found 410.1211.

1-Ethyl 6,6-dimethyl (*Z*)-7-(5-chloropyridin-3-yl)-10-oxa-8-azabicyclo[5.2.1]deca-3,8-diene-1,6,6-tricarboxylate (3s)

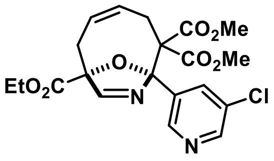

3s

Synthesized following General Procedure F using: ethyl 2-(5-chloropyridin-3-yl)oxazole-5-carboxylate **1s** (48.5 mg, 0.2 mmol, 1.0 eq.), dimethyl 2-vinylcyclopropane-1,1-dicarboxylate **2a** (73.7 mg, 0.4 mmol, 2.0 eq.), Ir-F (4.0 mg, 0.004 mmol, 2 mol%) and MeCN (4 mL, 0.05 M). From crude ^{1}H NMR analysis, a crude yield of 64% was determined for the major (5+4) regioisomer and 3% for the minor (5+2) regioisomer. Purification *via* column chromatography on silica gel (*n*-pentane/EtOAc 100:0 – 80:20) afforded the major regioisomer **3s** (52.4 mg, 0.12 mmol, 60%) as a yellowish oil.

TLC: R_f **=** 0.3 (70:30 *n*-pentane/EtOAc).

NMR Spectroscopy (***see spectra***):

^{1}H NMR (400 MHz, $CDCl_3$): δ_H 8.85 (s, 1H), 8.43 (d, *J* = 2.4 Hz, 1H), 8.24 – 8.15 (m, 1H), 7.24 (s, 1H), 6.01 – 5.90 (m, 1H), 5.71 – 5.62 (m, 1H), 4.18 – 4.02 (m, 2H), 3.83 (s, 3H), 3.48 (s, 3H), 3.16 (dd, *J* = 13.2, 9.2 Hz, 1H), 3.00 – 2.87 (m, 2H), 2.61 (dd, *J* = 13.5, 7.7 Hz, 1H), 1.14 (t, *J* = 7.1 Hz, 3H) ppm;

^{13}C NMR (101 MHz, $CDCl_3$): δ_C 169.4, 168.2, 168.0, 161.7, 147.9, 147.6, 138.0, 136.3, 131.0, 130.5, 128.3, 112.7, 98.3, 67.4, 62.5, 52.9, 52.7, 34.6, 32.6, 14.0 ppm.

HRMS (ESI$^+$): m/z calc'd for $C_{20}H_{21}ClN_2O_7Na$ [M+Na]$^+$: 459.0930, found 459.0922.

1-Ethyl 6,6-dimethyl (*Z*)-7-(1-(tert-butoxycarbonyl)-1*H*-indol-5-yl)-10-oxa-8-azabicyclo[5.2.1]deca-3,8-diene-1,6,6-tricarboxylate (3t)

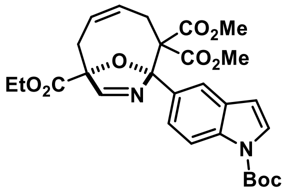

3t

Synthesized following General Procedure F using: ethyl 2-(1-(tert-butoxycarbonyl)-1*H*-indol-5-yl)oxazole-5-carboxylate **1t** (71.3 mg, 0.2 mmol, 1.0 eq.), dimethyl 2-vinylcyclopropane-1,1-dicarboxylate **2a** (73.7 mg, 0.4 mmol, 2.0 eq.), Ir-F (4.0 mg, 0.004 mmol, 2 mol%) and MeCN (4 mL, 0.05 M). From crude ^{1}H NMR analysis, a crude yield of 53% was determined for the major (5+4) regioisomer and 10% for the minor (5+2) regioisomer. Purification *via* column chromatography on silica gel (*n*-pentane/EtOAc 100:0 – 85:15) afforded the major regioisomer **3t** (46.5 mg, 0.086 mmol, 43%) as a yellowish oil.

TLC: R_f **=** 0.3 (80:20 *n*-pentane/EtOAc).

NMR Spectroscopy (***see spectra***):

^{1}H NMR (400 MHz, $CDCl_3$): δ_H 8.08 (s, 1H), 8.00 (d, *J* = 9.0 Hz, 1H), 7.87 (d, *J* = 8.9 Hz, 1H), 7.54 (d, *J* = 3.7 Hz, 1H), 7.23 (s, 1H), 6.54 (d, *J* = 3.7 Hz, 1H), 5.97 – 5.85 (m, 1H), 5.76 – 5.58 (m, 1H), 4.15 – 4.02 (m, 2H), 3.83 (s, 3H), 3.42 – 3.23 (m, 4H), 2.99 (ddd, *J* = 15.3, 6.2, 2.1 Hz, 1H), 2.89 (dd, *J* = 15.3, 8.5 Hz, 1H), 2.66 (dd, *J* = 13.3, 7.5 Hz, 1H), 1.65 (s, 9H), 1.13 (t, *J* = 7.1 Hz, 3H) ppm;

^{13}C NMR (101 MHz, $CDCl_3$): δ_C 169.7, 168.9, 168.6, 160.1, 149.9, 135.7, 134.8, 131.1, 129.5, 128.4, 126.0, 124.5, 121.0, 114.7, 113.5, 108.0, 97.6, 83.7, 68.2, 62.1, 52.6, 52.5, 35.1, 32.6, 28.3, 14.0 ppm.

HRMS (ESI$^+$): m/z calc'd for $C_{28}H_{32}N_2O_9Na$ [M+Na]$^+$: 563.2000, found 563.1999.

1-Ethyl 6,6-dimethyl (*Z*)-7-(pyrimidin-5-yl)-10-oxa-8-azabicyclo[5.2.1]deca-3,8-diene-1,6,6-tricarboxylate (3u)

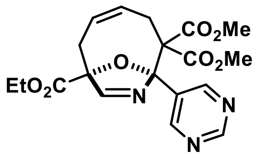

3u

Synthesized following General Procedure F using: ethyl 2-(pyrimidin-5-yl)oxazole-5-carboxylate **1u** (43.8 mg, 0.2 mmol, 1.0 eq.), dimethyl 2-vinylcyclopropane-1,1-dicarboxylate **2a** (73.7 mg, 0.4 mmol, 2.0 eq.), Ir-F (4.0 mg, 0.004 mmol, 2 mol%) and MeCN (4 mL, 0.05 M). From crude ^{1}H NMR analysis, a crude yield of 66% was determined for the major (5+4) regioisomer and 4% for the minor (5+2) regioisomer. Purification *via* column chromatography on silica gel (*n*-pentane/EtOAc 100:0 – 90:10) afforded the major regioisomer **3u** (45.1 mg, 0.1 mmol, 48%) as a yellowish oil.

TLC: R_f **=** 0.3 (70:30 *n*-pentane/EtOAc).

NMR Spectroscopy (***see spectra***):

^{1}H NMR (400 MHz, $CDCl_3$): δ_H 9.07 (s, 2H), 9.05 (s, 1H), 7.24 (s, 1H), 6.05 – 5.94 (m, 1H), 5.72 – 5.61 (m, 1H), 4.08 (m, 2H), 3.83 (s, 3H), 3.50 (s, 3H), 3.11 (dd, *J* = 13.3, 9.0 Hz, 1H), 3.01 – 2.87 (m, 2H), 2.61 (dd, *J* = 13.3, 7.7 Hz, 1H), 1.11 (t, *J* = 7.1 Hz, 3H) ppm;

^{13}C NMR (101 MHz, $CDCl_3$): δ_C 169.4, 168.1, 167.9, 162.0, 157.9, 156.8, 134.7, 131.0, 128.3, 112.1, 98.3, 67.0, 62.5, 52.9, 52.7, 34.4, 32.5, 14.0 ppm.

HRMS (ESI^+): m/z calc'd for $C_{19}H_{21}N_3O_7Na$ $[M+Na]^+$: 426.1271, found 426.1272.

1-Ethyl 6,6-dimethyl (*Z*)-7-(3,5-difluorophenyl)-10-oxa-8-azabicyclo[5.2.1]deca-3,8-diene-1,6,6-tricarboxylate (3v)

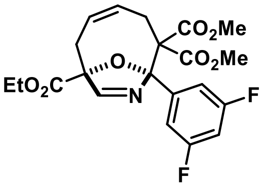

3v

Synthesized following General Procedure F using: ethyl 2-(3,5-difluorophenyl)oxazole-5-carboxylate **1v** (50.6 mg, 0.2 mmol, 1.0 eq.), dimethyl 2-vinylcyclopropane-1,1-dicarboxylate **2a** (73.7 mg, 0.4 mmol, 2.0 eq.), Ir-F (4.0 mg, 0.004 mmol, 2 mol%) and MeCN (4 mL, 0.05 M). From crude ^{1}H NMR analysis, a crude yield of 57% was determined for the major (5+4) regioisomer and 6% for the minor (5+2) regioisomer. Purification *via* column chromatography on silica gel (*n*-pentane/EtOAc 100:0 – 90:10) afforded the major regioisomer **3v** (49.0 mg, 0.11 mmol, 56%) as a colourless oil.

TLC: R_f **=** 0.2 (90:10 *n*-pentane/EtOAc).

NMR Spectroscopy (***see spectra***):

^{1}H NMR (400 MHz, $CDCl_3$): δ_H 7.53 – 7.39 (m, 2H), 7.22 (s, 1H), 6.74 – 6.60 (m, 1H), 5.97 – 5.83 (m, 1H), 5.73 – 5.59 (m, 1H), 4.19 – 4.07 (m, 2H), 3.83 (s, 3H), 3.43 (s, 3H), 3.25 (dd, *J* = 13.4, 9.3 Hz, 1H), 3.01 – 2.84 (m, 2H), 2.63 (dd, *J* = 13.4, 7.5 Hz, 1H), 1.18 (t, *J* = 7.1 Hz, 3H) ppm;

^{13}C{^{19}F} NMR (151 MHz, $CDCl_3$): δ_C 169.4, 168.4, 168.1, 161.8, 161.1, 144.7, 131.0, 128.4, 113.5, 111.6, 103.4, 67.8, 62.4, 52.8, 52.6, 34.7, 32.6, 13.9 ppm;

^{13}C NMR (151 MHz, $CDCl_3$): δ_C 169.4, 168.4, 168.1, 161.8 (dd, *J* = 246.3, 12.4 Hz), 161.1, 144.7 (t, *J* = 7.9 Hz), 131.0, 128.4, 113.5, 111.6 (d, *J* = 27.8 Hz), 103.4 (t, *J* = 25.3 Hz), 67.8, 62.4, 52.8, 52.6, 34.7, 32.6, 13.9 ppm;
Note: Ester substituted quaternary carbon that is adjacent to the oxazole oxygen is not visible in ^{13}C with reasonable scans.

^{19}F NMR (563 MHz, $CDCl_3$) δ_F -111.0 ppm.

HRMS (ESI^+): m/z calc'd for $C_{21}H_{21}F_2NO_7Na$ $[M+Na]^+$: 460.1178, found 460.1181.

1-Ethyl 6,6-dimethyl (*Z*)-7-(4-methoxyphenyl)-10-oxa-8-azabicyclo[5.2.1]deca-3,8-diene-1,6,6-tricarboxylate (3w) & 8a-ethyl 4,4-dimethyl 2-(4-methoxyphenyl)-5,8-dihydro-4H-cyclohepta[d]oxazole-4,4,8a(3aH)-tricarboxylate (3w')

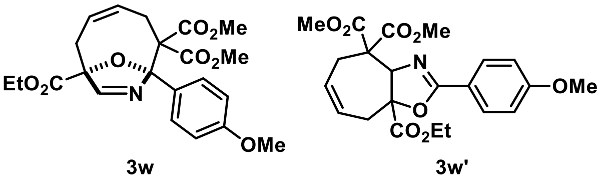

Synthesized following General Procedure F using: ethyl 2-(4-methoxyphenyl)oxazole-5-carboxylate **1w** (49.5 mg, 0.2 mmol, 1.0 eq.), dimethyl 2-vinylcyclopropane-1,1-dicarboxylate **2a** (73.7 mg, 0.4 mmol, 2.0 eq.), Ir-F (4.0 mg, 0.004 mmol, 2 mol%) and MeCN (4 mL, 0.05 M). From crude ^{1}H NMR analysis, a crude yield of 34% was determined for the major (5+4) regioisomer and 7% for the minor (5+2) regioisomer. Purification *via* column chromatography on silica gel (*n*-pentane/ EtOAc 100:0 – 80:20) afforded a mixture of **3w** and **3w'** (33.7 mg, 0.08 mmol, 39%, 83:17 r.r..) as a colourless oil. Characterization data for the combined regioisomers are reported below.

TLC: R_f **=** 0.3 (80:20 *n*-pentane/EtOAc).

NMR Spectroscopy (***see spectra***):

> ^{1}**H NMR** (400 MHz, $CDCl_3$, mixture of regioisomers): δ_H 7.93 – 7.86 (m, 0.34H, min), 7.83 – 7.73 (m, 1.66H, maj), 7.19 (s, 0.78H, maj), 6.90 – 6.87 (m, 0.34H, min), 6.83 – 6.73 (m, 1.66H, maj), 5.95 – 5.80 (m, 0.83H, maj), 5.71 – 5.58 (m, 0.83H, maj), 5.55 – 5.46 (m, 0.34H, min), 5.40 – 5.32 (m, 0.17H, min), 4.24 (q, *J* = 7.1 Hz, 0.34H, min), 4.10 (q, *J* = 7.1 Hz, 1.66H, maj), 3.82 (s, 0.51H, min), 3.81 – 3.79 (m, 3H), 3.76 (s, 2.49H, maj), 3.75 (s, 0.51H, min), 3.36 (s, 2.40H, maj), 3.33 – 3.23 (m, 0.83H, maj), 3.14 – 3.05 (m, 0.17H, min), 3.01 – 2.72 (m, 2H), 2.68 – 2.51 (m, 1H), 1.27 (t, *J* = 7.1 Hz, 0.51H, min), 1.16 (t, *J* = 7.1 Hz, 2.49H, maj) ppm;

> ^{13}**C NMR** (101 MHz, $CDCl_3$, mixture of regioisomers): δ_C 171.9, 170.3, 169.8, 169.1, 168.8, 168.5, 164.6, 162.7, 160.0, 159.2, 133.5, 131.1, 130.6, 129.4, 128.3, 127.1, 123.5, 119.5, 114.3, 113.8, 112.3, 97.7, 89.0, 73.7, 68.1, 62.2, 62.1, 60.7, 55.48, 55.2, 53.3, 52.9, 52.5, 52.4, 35.0, 32.5, 31.8 29.1, 14.2, 14.0 ppm.

HRMS (ESI$^+$): m/z calc'd for $C_{22}H_{25}NO_8Na$ [M+Na]$^+$: 454.1472, found 454.1468.

1-Ethyl 6,6-dimethyl (*Z*)-7-(3-(trifluoromethyl)phenyl)-10-oxa-8-azabicyclo[5.2.1]deca-3,8-diene-1,6,6-tricarboxylate (3x)

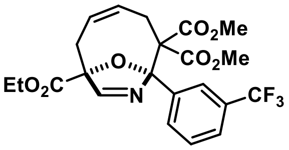

3x

Synthesized following General Procedure F using: ethyl 2-(3-(trifluoromethyl)phenyl)oxazole-5-carboxylate **1x** (57.4 mg, 0.2 mmol, 1.0 eq.), dimethyl 2-vinylcyclopropane-1,1-dicarboxylate **2a** (73.7 mg, 0.4 mmol, 2.0 eq.), Ir-F (4.0 mg, 0.004 mmol, 2 mol%) and MeCN (4 mL, 0.05 M). From crude ^{1}H NMR analysis, a crude yield of

48% was determined for the major (5+4) regioisomer and 7% for the minor (5+2) regioisomer. Purification *via* column chromatography on silica gel (*n*-pentane/EtOAc 100:0 – 90:10) afforded the major regioisomer **3x** (45.1 mg, 0.1 mmol, 48%) as a yellowish oil.

TLC: R_f **=** 0.3 (80:20 *n*-pentane/EtOAc).

NMR Spectroscopy (***see spectra***):

^{1}H NMR (400 MHz, $CDCl_3$): δ_H 8.15 (d, *J* = 8.0 Hz, 1H), 8.08 (s, 1H), 7.51 (d, *J* = 7.8 Hz, 1H), 7.40 (t, *J* = 7.9 Hz, 1H), 7.25 (s, 1H), 6.00 – 5.88 (m, 1H), 5.73 – 5.62 (m, 1H), 4.18 – 4.02 (m, 2H), 3.82 (s, 3H), 3.38 (s, 3H), 3.31 – 3.21 (m, 1H), 3.04 – 2.87 (m, 2H), 2.64 (dd, *J* = 13.3, 7.6 Hz, 1H), 1.12 (t, *J* = 7.1 Hz, 3H) ppm;

^{13}C{^{19}F} NMR (126 MHz, $CDCl_3$): δ_C 169.5, 168.6, 168.3, 161.1, 142.0, 132.2, 131.1, 129.3, 128.4, 127.5, 125.3, 124.9, 124.4, 113.9, 67.9, 62.4, 52.6, 52.5, 34.8, 32.5, 13.9 ppm;

^{13}C NMR (126 MHz, $CDCl_3$): δ_C 169.5, 168.6, 168.3, 161.1, 142.0, 132.2, 131.1, 129.3 (q, *J* = 31.8 Hz), 128.4, 127.5, 125.3 (q, *J* = 3.8 Hz), 124.9 (q, *J* = 4.1 Hz), 124.4 (q, *J* = 272.4 Hz), 113.9, 67.9, 62.4, 52.6, 52.5, 34.8, 32.5, 13.9 ppm;
Note: Ester substituted quaternary carbon that is adjacent to the oxazole oxygen is not visible in ^{13}C with reasonable scans.

^{19}F NMR (376 MHz, $CDCl_3$) δ_F -62.4 ppm.

HRMS (ESI$^+$): m/z calc'd for $C_{22}H_{22}F_3NO_7Na$ $[M+Na]^+$: 492.1241, found 492.1238.

1-Ethyl 6,6-dimethyl (*Z*)-9-methyl-7-phenyl-10-oxa-8-azabicyclo[5.2.1]deca-3,8-diene-1,6,6-tricarboxylate (3y)

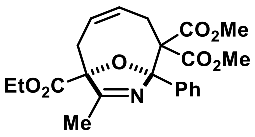

3y

Synthesized following General Procedure F using: ethyl 4-methyl-2-phenyloxazole-5-carboxylate **1y** (46.3 mg, 0.2 mmol, 1.0 eq.), dimethyl 2-vinylcyclopropane-1,1-dicarboxylate **2a** (73.7 mg, 0.4 mmol, 2.0 eq.), Ir-F (4.0 mg, 0.004 mmol, 2 mol%) and MeCN (4 mL, 0.05 M). From crude ^{1}H NMR analysis, a crude yield of 30% was determined for the major (5+4) regioisomer. Purification *via* column chromatography on silica gel (*n*-pentane/EtOAc 100:0 – 85:15) afforded **3y** (24.9 mg, 0.06 mmol, 30%) as a yellowish oil.

TLC: R_f **=** 0.3 (80:20 *n*-pentane/EtOAc).

NMR Spectroscopy (***see spectra***):

^{1}H NMR (400 MHz, $CDCl_3$): δ_H 7.91 – 7.80 (m, 2H), 7.26 – 7.17 (m, 3H), 5.98 – 5.87 (m, 1H), 5.77 – 5.66 (m, 1H), 4.16 – 3.93 (m, 2H), 3.81 (s, 3H), 3.38 – 3.24 (m, 4H), 2.90 (d, *J* = 7.2 Hz, 2H), 2.61 (dd, *J* = 13.3, 7.5 Hz, 1H), 1.89 (s, 3H), 1.07 (t, *J* = 6.9 Hz, 3H) ppm;

^{13}C NMR (101 MHz, CDCl$_3$): δ_C 169.8, 169.2, 168.6, 167.4, 141.4, 131.8, 128.3, 127.8, 127.5, 126.8, 112.4, 98.1, 70.7, 62.0, 52.4, 52.3, 34.2, 32.7, 15.1, 13.9 ppm.

HRMS (ESI$^+$): m/z calc'd for $C_{22}H_{25}NO_7Na$ [M+Na]$^+$: 438.1523, found 438.1520.

Dimethyl (*Z*)-1-acetyl-8-methyl-10-thiabicyclo[5.2.1]deca-4,8-diene-2,2-dicarboxylate (3z)

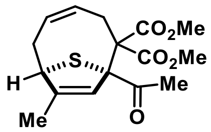

3z

Synthesized following General Procedure F using: 1-(4-methylthiophen-2-yl)ethan-1-one **1z** (28.0 mg, 0.2 mmol, 1.0 eq.), dimethyl 2-vinylcyclopropane-1,1-dicarboxylate **2a** (92.0 mg, 0.5 mmol, 2.5 eq.), Ir-F (4.0 mg, 0.004 mmol, 2 mol%) and MeCN (2 mL). From crude ^{1}H NMR analysis, a crude yield of 47% and >95:5 regioisomeric ratio was determined. Purification *via* column chromatography on silica gel (*n*-pentane/EtOAc 100:0 – 90:10) afforded the title compound **3z** (30.9 mg, 0.095 mmol, 48%) as a single regioisomer as a white solid.

TLC: R_f **=** 0.3 (90:10 *n*-pentane/EtOAc).

NMR Spectroscopy (***see spectra***):

^{1}H NMR (400 MHz, CDCl$_3$): δ_H 5.92 – 5.80 (m, 1H), 5.57 – 5.49 (m, 1H), 5.49 – 5.45 (m, 1H), 4.14 (t, J = 4.0 Hz, 1H), 3.79 (s, 3H), 3.74 (s, 3H), 3.47 – 3.32 (m, 1H), 2.72 – 2.61 (m, 3H), 2.28 (s, 3H), 1.62 (s, 3H) ppm;

^{13}C NMR (101 MHz, CDCl$_3$): δ_C 203.7, 172.2, 168.5, 147.2, 130.8, 128.8, 127.0, 65.8, 64.6, 57.2, 52.8, 52.7, 35.7, 35.1, 25.8, 15.9 ppm.

HRMS (ESI$^+$): m/z calc'd for $C_{16}H_{20}O_5SNa$ [M+Na]$^+$: 347.0924, found 347.0923.

Dimethyl (*Z*)-1-acetyl-8-cyclohexyl-10-thiabicyclo[5.2.1]deca-4,8-diene-2,2-dicarboxylate (3aa)

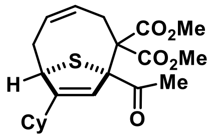

3aa

Synthesized following General Procedure F using: 1-(4-cyclohexylthiophen-2-yl)ethan-1-one **1aa** (41.6 mg, 0.2 mmol, 1.0 eq.), dimethyl 2-vinylcyclopropane-1,1-dicarboxylate **2a** (92.0 mg, 0.5 mmol, 2.5 eq.), Ir-F (4.0 mg, 0.004 mmol, 2 mol%) and MeCN (2 mL). From crude ^{1}H NMR analysis, a crude yield of 37% and >95:5 regioisomeric ratio was determined. Purification *via* column chromatography on silica gel (*n*-pentane/EtOAc 100:0 – 97:3) afforded the title compound **3aa** (30.1 mg, 0.077 mmol, 38%) as a single regioisomer as a white solid.

TLC: R_f **=** 0.4 (92:8 *n*-pentane/EtOAc).

NMR Spectroscopy (***see spectra***):

^{1}H NMR (400 MHz, $CDCl_3$): δ_H 5.92 – 5.75 (m, 1H), 5.47 – 5.36 (m, 2H), 4.36 (t, *J* = 3.8 Hz, 1H), 3.79 (s, 3H), 3.74 (s, 3H), 3.48 – 3.31 (m, 1H), 2.72 – 2.57 (m, 3H), 2.25 (s, 3H), 1.91 (d, *J* = 13.1 Hz, 1H), 1.82 – 1.64 (m, 5H), 1.35 – 1.13 (m, 4H), 1.12 – 0.95 (m, 1H) ppm;

^{13}C NMR (101 MHz, $CDCl_3$)*:* δ_C 203.7, 172.3, 170.2, 156.7, 130.3, 129.9, 123.8, 65.2, 54.7, 52.8, 52.6, 38.5, 36.0, 35.6, 32.6, 31.6, 26.6, 26.3, 26.3, 25.7 ppm.

HRMS (ESI$^+$): m/z calc'd for $C_{21}H_{28}O_5SNa$ $[M+Na]^+$: 415.1550, found 415.1548.

Di-*tert*-butyl (*Z*)-1-(3-phenylpropanoyl)-10-oxabicyclo[5.2.1]deca-4,8-diene-2,2-dicarboxylate (3ab) & di-*tert*-butyl (*Z*)-7-(3-phenylpropanoyl)-10-oxabicyclo[5.2.1]deca-4,8-diene-2,2-dicarboxylate (3ab')

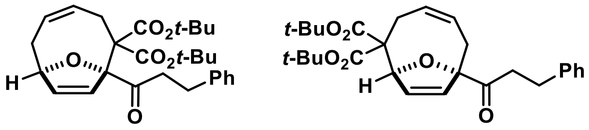

3ab **3ab'**

Synthesized following General Procedure F using: 1-(furan-2-yl)-3-phenylpropan-1-one **1a** (40.0 mg, 0.2 mmol, 1.0 eq.), di-*tert*-butyl 2-vinylcyclopropane-1,1-dicarboxylate **2b** (134 mg, 0.5 mmol, 2.5 eq.), Ir-F (4.0 mg, 0.004 mmol, 2 mol%) and MeCN (2 mL). From crude ^{1}H NMR analysis, a crude yield of 59% and 76:24 regioisomeric ratio was determined. Purification *via* column chromatography on silica gel (*n*-pentane/EtOAc 100:0 – 97:3) afforded the title compound **3ab** (41.9 mg, 0.09 mmol, 45%) as a colourless oil, as well as the minor regioisomer **3ab'** (13.2 mg, 0.03 mmol, 14%) as a colourless oil.

Characterization data for **3ab**:

TLC: R_f **=** 0.2 (97:3 *n*-pentane/EtOAc).

NMR Spectroscopy (***see spectra***):

^{1}H NMR (400 MHz, $CDCl_3$): δ_H 7.26 – 7.10 (m, 5H), 5.91 – 5.82 (m, 3H), 5.58 – 5.47 (m, 1H), 5.27 (t, *J* = 5.5 Hz, 1H), 3.21 – 3.08 (m, 1H), 3.02 – 2.54 (m, 5H), 2.39 – 2.29 (m, 1H), 2.27 – 2.16 (m, 1H), 1.54 (s, 9H), 1.47 (s, 9H) ppm;

^{13}C NMR (101 MHz, $CDCl_3$): δ_C 209.1, 169.6, 168.4, 142.0, 133.7, 130.3, 128.6, 128.5, 128.4, 127.9, 125.8, 99.7, 82.1, 82.0, 68.5, 38.4, 33.2, 33.0, 29.8, 28.2 ppm.

Characterization data for **3ab'**:

TLC: R_f **=** 0.3 (97:3 *n*-pentane/EtOAc).

NMR Spectroscopy (***see spectra***):

^{1}H NMR (400 MHz, $CDCl_3$): δ_H 7.30 – 7.21 (m, 2H), 7.21 – 7.12 (m, 3H), 6.06 – 5.94 (m, 1H), 5.84 (dd, *J* = 6.0, 1.8 Hz, 1H), 5.60 (t, *J* = 2.0 Hz, 1H), 5.47 – 5.36 (m, 2H), 3.22 – 3.09 (m, 1H), 3.01 – 2.90 (m, 1H), 2.89 – 2.79 (m, 2H), 2.78 – 2.69 (m, 1H), 2.62 – 2.43 (m, 3H), 1.47 (d, *J* = 1.9 Hz, 18H) ppm;

^{13}C NMR (151 MHz, $CDCl_3$): δ_C 211.0, 170.4, 168.0, 141.5, 131.5, 129.5, 129.2, 128.6, 128.5, 126.1, 101.0, 86.8, 82.2, 82.1, 64.4, 39.6, 34.8, 29.6, 29.1, 28.1, 28.0, 28.0 ppm.

HRMS (ESI^+): m/z calc'd for $C_{28}H_{36}O_6Na$ $[M+Na]^+$: 491.2404, found 491.2406.

(*Z*)-1-(3-Phenylpropanoyl)-10-oxabicyclo[5.2.1]deca-4,8-diene-2,2-dicarbonitrile (3ac)

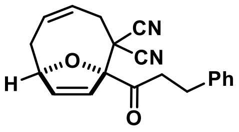

3ac

Synthesized following General Procedure F using: 1-(furan-2-yl)-3-phenylpropan-1-one **1a** (40.0 mg, 0.2 mmol, 1.0 eq.), 2-vinylcyclopropane-1,1-dicarbonitrile **2c** (59.0 mg, 0.5 mmol, 2.5 eq.), Ir-F (4.0 mg, 0.004 mmol, 2 mol%) and MeCN (2 mL). From crude ^{1}H NMR analysis, a crude yield of 75% and >95:5 regioisomeric ratio was determined. Purification *via* column chromatography on silica gel (*n*-pentane/EtOAc 100:0 – 90:10) afforded the title compound **3ac** (40.1 mg, 0.13 mmol, 63%) as a single regioisomer as a colourless oil.

TLC: R_f **=** 0.4 (90:10 *n*-pentane/EtOAc).

NMR Spectroscopy (***see spectra***):

^{1}H NMR (400 MHz, $CDCl_3$): δ_H 7.32 – 7.24 (m, 2H), 7.24 – 7.14 (m, 3H), 5.91 – 5.79 (m, 3H), 5.67 – 5.57 (m, 1H), 5.55 (t, *J* = 3.3 Hz, 1H), , 3.10 – 3.00 (m, 1H), 2.99 – 2.80 (m, 4H), 2.79 – 2.66 (m, 2H), 2.51 – 2.39 (m, 1H) ppm;

^{13}C NMR (101 MHz, $CDCl_3$): δ_C 207.4, 140.7, 135.3, 133.5, 128.7, 128.5, 126.4, 126.2, 124.3, 115.2, 113.4, 97.9, 89.6, 40.3, 38.9, 34.1, 33.3, 29.0 ppm.

HRMS (ESI^+): m/z calc'd for $C_{20}H_{18}N_2O_2Na$ $[M+Na]^+$: 341.1261, found 341.1265.

Ethyl (*Z*)-2-cyano-1-(3-phenylpropanoyl)-10-oxabicyclo[5.2.1]deca-4,8-diene-2-carboxylate (3ad) & ethyl (*Z*)-2-cyano-7-(3-phenylpropanoyl)-10-oxabicyclo[5.2.1]deca-4,8-diene-2-carboxylate (3ad')

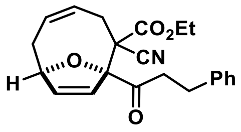

3ad

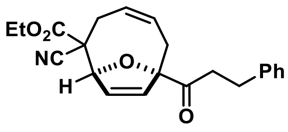

3ad'

Synthesized following General Procedure F using: 1-(furan-2-yl)-3-phenylpropan-1-one **1a** (40.0 mg, 0.2 mmol, 1.0 eq.), ethyl 1-cyano-2-vinylcyclopropane-1-carboxylate **2d** (82.5 mg, 0.5 mmol, 2.5 eq.), Ir-F (4.0 mg, 0.004 mmol, 2 mol%) and MeCN (2 mL). From crude ^{1}H NMR analysis, a crude yield of 82% and 82:18 regioisomeric ratio was determined. Purification *via* column chromatography on silica gel (*n*-pentane/EtOAc 100:0 – 90:10) afforded the title compound **3ad** (45.1 mg, 0.12 mmol, 62%, 53:47 d.r..) as a colourless oil, as well as the minor regioisomer **3ad'** (12.5 mg, 0.03 mmol, 17%, 70:30 d.r..) as a colourless oil. Diastereomers were assigned *via* 1D-NOESY NMR.

Characterization data for **3ad d$_1$**:

TLC: R_f **=** 0.2 (90:10 *n*-pentane/EtOAc).

NMR Spectroscopy (***see spectra***):

^{1}**H NMR** (599 MHz, $CDCl_3$): δ_H 7.28 – 7.23 (m, 2H), 7.20 – 7.14 (m, 3H), 5.92 – 5.84 (m, 1H), 5.81 (dd, *J* = 5.9, 1.5 Hz, 1H), 5.76 (d, *J* = 6.0 Hz, 1H), 5.59 – 5.53 (m, 1H), 5.52 – 5.50 (m, 1H), 4.26 – 4.13 (m, 2H), 3.04 – 2.93 (m, 2H), 2.92 – 2.72 (m, 4H), 2.47 – 2.35 (m, 2H), 1.29 (t, *J* = 7.2 Hz, 3H) ppm;

^{13}**C NMR** (151 MHz, $CDCl_3$): δ_C 210.0, 169.4, 141.2, 133.7, 131.8, 128.5, 128.5, 128.5, 126.2, 125.8, 117.0, 97.8, 89.6, 63.2, 54.6, 38.1, 33.5, 33.5, 29.0, 14.1 ppm.

Characterization data for **3ad d$_2$**:

TLC: R_f **=** 0.3 (90:10 *n*-pentane/EtOAc).

NMR Spectroscopy (***see spectra***):

^{1}**H NMR** (400 MHz, $CDCl_3$): δ_H 7.32 – 7.22 (m, 2H), 7.22 – 7.11 (m, 3H), 5.99 – 5.94 (m, 2H), 5.82 – 5.76 (m, 1H), 5.69 – 5.62 (m, 1H), 5.36 (t, *J* = 4.8 Hz, 1H), 4.41 – 4.29 (m, 2H), 3.06 – 2.61 (m, 6H), 2.45 – 2.40 (m, 1H), 2.34 – 2.26 (m, 1H), 1.40 (t, *J* = 7.1 Hz, 3H) ppm;

^{13}**C NMR** (151 MHz, $CDCl_3$): δ_C 209.1, 166.2, 141.0, 134.6, 130.5, 128.6, 128.5, 127.3, 126.2, 126.2, 118.4, 99.6, 86.3, 63.0, 38.9, 33.2, 33.0, 29.1, 14.1 ppm.

Characterization data for **3ad'**:

TLC: R_f **=** 0.5 (90:10 *n*-pentane/EtOAc).

NMR Spectroscopy (***see spectra***):

^{1}**H NMR** (599 MHz, $CDCl_3$): δ_H 7.31 – 7.26 (m, 2H), 7.22 – 7.14 (m, 3H), 6.15 (dd, *J* = 6.0, 1.9 Hz, 0.7H, d$_1$), 5.96 – 5.92 (m, 0.2H, d$_2$), 5.91 – 5.85 (m, 0.7H, d$_1$), 5.77 (dd, *J* = 6.0, 1.8 Hz, 0.2H, d$_2$), 5.65 (d, *J* = 6.0 Hz, 0.2H, d$_2$), 5.61 – 5.55 (m, 0.7H, d$_1$), 5.54 (dd, *J* = 6.0, 2.1 Hz, 0.7H, d$_1$), 5.51 (t, *J* = 1.9 Hz, 0.2H, d$_2$), 5.43 (t, *J* = 2.1 Hz, 0.7H, d$_1$), 4.37 – 4.25 (m, 2H), 3.12 – 3.02 (m, 1H), 3.01 – 2.82 (m, 6H), 2.51 – 2.42 (m, 2H), 1.38 – 1.33 (m, 3H) ppm;

^{13}**C NMR** (151 MHz, $CDCl_3$): δ_C 209.8 and 209.5, 168.2 and 165.7, 141.2 and 141.1, 132.2, 131.5, 129.2, 128.6 and 128.6, 128.5 and 128.5, 126.4, 126.3 and 126.3, 117.0, 102.5 and 100.8, 88.2 and 85.9, 63.5 and 63.2, 50.6, 39.8 and 39.7, 35.4 and 35.0, 30.1, 29.5 and 29.5, 14.1 and 14.1 ppm.

HRMS (ESI^+): m/z calc'd for $C_{22}H_{23}NO_4Na$ $[M+Na]^+$: 388.1519, found 388.1520.

Dibenzyl (*Z*)-1-(3-phenylpropanoyl)-10-oxabicyclo[5.2.1]deca-4,8-diene-2,2-dicarboxylate (3ae) & dibenzyl (*Z*)-7-(3-phenylpropanoyl)-10-oxabicyclo[5.2.1]deca-4,8-diene-2,2-dicarboxylate (3ae')

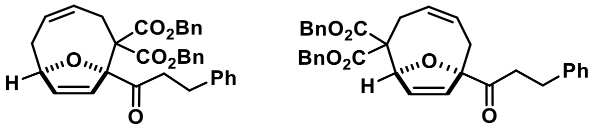

3ae **3ae'**

Synthesized following General Procedure F using: 1-(furan-2-yl)-3-phenylpropan-1-one **1a** (40.0 mg, 0.2 mmol, 1.0 eq.), dibenzyl 2-vinylcyclopropane-1,1-dicarboxylate **2e** (168.1 mg, 0.5 mmol, 2.5 eq.), Ir-F (4.0 mg, 0.004 mmol, 2 mol%) and MeCN (2 mL). From crude ^{1}H NMR analysis, a crude yield of 67% and 81:19 regioisomeric ratio was determined. Purification *via* column chromatography on silica gel (*n*-pentane/EtOAc 97:3 – 93:7) afforded the title compound **3ae** (57.7 mg, 0.11 mmol, 54%) as a colourless oil, as well as the minor regioisomer **3ae'** (14.0 mg, 0.03 mmol, 13%) as a colourless oil.

<u>Characterization data for **3ae**:</u>

TLC: R_f **=** 0.3 (97:3 *n*-pentane/EtOAc).

NMR Spectroscopy (***see spectra***):

^{1}**H NMR** (400 MHz, $CDCl_3$): δ_H 7.31 – 7.04 (m, 15H), 5.90 (dd, *J* = 6.0, 1.7 Hz, 1H), 5.83 (dd, *J* = 6.0, 1.6 Hz, 1H), 5.79 – 5.67 (m, 1H), 5.55 – 5.45 (m, 1H), 5.26 (d, *J* = 12.4 Hz, 1H), 5.23 – 5.18 (m, 1H), 5.11 – 5.00 (m, 3H), 3.16 – 3.04 (m, 1H), 2.81 – 2.54 (m, 5H), 2.38 – 2.26 (m, 1H), 2.22 – 2.07 (m, 1H) ppm;

^{13}**C NMR** (101 MHz, $CDCl_3$): δ_C 209.6, 170.2, 169.2, 141.8, 135.7, 135.3, 134.9, 129.7, 128.8, 128.6, 128.6, 128.6, 128.5, 128.5, 128.4, 128.3, 128.1, 127.6, 125.9, 100.0, 84.6, 67.7, 67.5, 67.3, 38.4, 33.1, 32.2, 29.5 ppm.

<u>Characterization data for **3ae'**:</u>

TLC: R_f **=** 0.4 (97:3 *n*-pentane/EtOAc).

NMR Spectroscopy (***see spectra***):

^{1}**H NMR** (400 MHz, $CDCl_3$): δ_H 7.36 – 7.14 (m, 15H), 6.02 – 5.90 (m, 1H), 5.70 (t, *J* = 2.0 Hz, 1H), 5.66 (dd, *J* = 6.0, 1.8 Hz, 1H), 5.46 – 5.35 (m, 2H), 5.21 – 5.02 (m, 4H), 3.15 – 3.04 (m, 1H), 2.99 – 2.80 (m, 3H), 2.76 – 2.66 (m, 2H), 2.64 – 2.56 (m, 1H), 2.55 – 2.42 (m, 1H) ppm;

^{13}**C NMR** (101 MHz, $CDCl_3$): δ_C 210.8, 170.6, 168.3, 141.4, 135.4, 135.1, 131.6, 130.0, 129.0, 128.8, 128.7, 128.7, 128.7, 128.6, 128.6, 128.5, 128.5, 128.1, 126.2, 101.1, 86.6, 67.6, 67.4, 63.3, 39.7, 35.1, 29.6, 29.0 ppm.

HRMS (ESI^+): m/z calc'd for $C_{34}H_{32}O_6Na$ $[M+Na]^+$: 559.2091, found 559.2087.

Tetraethyl (1-(3-phenylpropanoyl)-10-oxabicyclo[5.2.1]deca-4,8-diene-2,2-diyl)(*Z*)-bis(phosphonate) (3af) & tetraethyl (7-(3-phenylpropanoyl)-10-oxabicyclo[5.2.1]deca-4,8-diene-2,2-diyl)(*Z*)-bis(phosphonate) (3af')

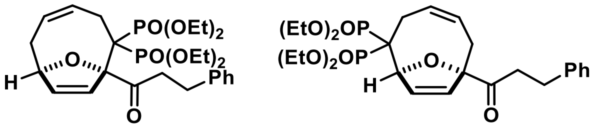

3af **3af'**

Synthesized following General Procedure F using: 1-(furan-2-yl)-3-phenylpropan-1-one **1a** (40.0 mg, 0.2 mmol, 1.0 eq.), tetraethyl (2-vinylcyclopropane-1,1-diyl)bis(phosphonate) **2f** (170 mg, 0.5 mmol, 2.5 eq.), Ir-F (4.0 mg, 0.004 mmol, 2 mol%) and MeCN (2 mL). From crude ^{1}H NMR analysis, a crude yield of 20% was determined. Purification *via* column chromatography on silica gel (CH_2Cl_2/MeOH 100:0 – 97:3 afforded the title compound (11.9 mg, 0.02 mmol, 11%, 50:50 r.r.) as a colourless oil as a mixture of regioisomers **3af** and **3af'**.

Note: The lower yield is attributed to a slower reaction and competitive decomposition of the starting material under the reaction conditions.

TLC: R_f **=** 0.3 (97:3 CH_2Cl_2/MeOH).

NMR Spectroscopy (***see spectra***):

^{1}H NMR (400 MHz, $CDCl_3$): δ_H 7.28 – 7.21 (m, 2H), 7.19 – 7.14 (m, 3H), 6.16 – 6.11 (m, 0.5H, r_1), 6.11 – 6.05 (m, 0.5H, r_2), 6.02 (d, J = 5.8 Hz, 0.5H, r_1), 5.90 (d, J = 5.8 Hz, 0.5H, r_2), 5.87 – 5.79 (m, 1.0H), 5.70 – 5.42 (m, 1.5H), 5.41 – 5.29 (m, 0.5H, r_1), 4.21 – 4.09 (m, 8H), 3.17 – 2.99 (m, 2H), 2.97 – 2.48 (m, 4H), 2.45 – 2.20 (m, 2H), 1.39 – 1.27 (m, 12H) ppm;

^{13}C NMR (151 MHz, $CDCl_3$): δ_C 211.6, 141.3, 132.5, 131.5 and 131.5 and 131.5, 131.0, 128.6 and 128.5, 128.5 and 128.5, 126.9, 126.2 and 126.2, 104.8 and 103.9, 98.1 and 97.7, 63.0 and 62.7, 38.6 and 37.1, 38.0 and 36.2, 29.8 and 28.5, 29.5 and 29.4, 16.5 and 16.5 ppm.

HRMS (ESI$^+$): m/z calc'd for $C_{26}H_{38}O_8P_2Na$ [M+Na]$^+$: 563.1934, found 563.1935.

Methyl (*Z*)-3-benzyl-7-phenyl-10-oxa-8-azabicyclo[5.2.1]deca-3,8-diene-1-carboxylate (3ag) & methyl 7-benzyl-2-phenyl-3a,4,5,8-tetrahydro-8a*H*-cyclohepta[*d*]oxazole-8a-carboxylate (3ag')

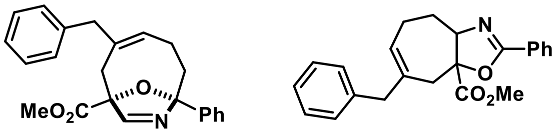

3ag **3ag'**

Synthesized following General Procedure F using: methyl 2-phenyloxazole-5-carboxylate **1r** (40.6 mg, 0.2 mmol, 1.0 eq.), (2-cyclopropylallyl)benzene **2g** (63.2 mg, 0.4 mmol, 2.0 eq.), Ir-F (4.0 mg, 0.004 mmol, 2 mol%) and MeCN (2 mL). From crude ^{1}H NMR analysis, a crude yield of 61% and 75:25 regioisomeric ratio was determined. Purification *via* column chromatography on silica gel (*n*-pentane/EtOAc 100:0 – 88:12) afforded the title compound **3ag** (45.4 mg, 0.13 mmol, 63%) as a colourless oil, as well as the (5+2) regioisomer **3ag'** (15.2 mg, 0.04 mmol, 21%) as a colourless oil.

<u>Characterization data for **3ag**:</u>

TLC: R_f **=** 0.6 (88:12 *n*-pentane/EtOAc).

NMR Spectroscopy (***see spectra***):

^{1}**H NMR** (400 MHz, $CDCl_3$): δ_H 7.59 – 7.54 (m, 2H), 7.35 – 7.29 (m, 4H), 7.28 – 7.16 (m, 4H), 6.70 (s, 1H), 5.73 (t, *J* = 8.1 Hz, 1H), 3.74 (s, 3H), 3.36 – 3.22 (m, 2H), 2.91 (d, *J* = 15.0 Hz, 1H), 2.65 (dd, *J* = 15.0, 1.3 Hz, 1H), 2.59 – 2.43 (m, 2H), 2.15 – 2.11 (m, 1H), 2.03 – 1.89 (m, 1H) ppm;

^{13}**C NMR** (101 MHz, $CDCl_3$): δ_C 170.1, 157.0, 144.6, 138.9, 136.4, 129.5, 129.4, 128.6, 128.4, 127.6, 126.6, 124.8, 114.0, 95.8, 52.9, 46.7, 39.5, 39.2, 24.1 ppm.

<u>Characterization data for **3ag'**:</u>

TLC: R_f **=** 0.3 (88:12 *n*-pentane/EtOAc).

NMR Spectroscopy (***see spectra***):

^{1}**H NMR** (400 MHz, $CDCl_3$): δ_H 7.94 – 7.87 (m, 2H), 7.52 – 7.43 (m, 1H), 7.43 – 7.35 (m, 2H), 7.33 – 7.25 (m, 2H), 7.25 – 7.12 (m, 3H), 5.40 (t, *J* = 4.0 Hz, 1H), 4.76 (dd, *J* = 10.0, 2.9 Hz, 1H), 3.70 (s, 3H), 3.29 (s, 2H), 2.73 (q, *J* = 15.5 Hz, 2H), 2.54 – 2.39 (m, 1H), 2.35 – 2.24 (m, 2H), 2.14 – 2.01 (m, 1H) ppm;

^{13}**C NMR** (101 MHz, $CDCl_3$): δ_C 172.1, 161.3, 139.4, 131.8, 131.6, 129.3, 128.5, 128.4, 127.4, 126.4, 89.8, 71.6, 52.9, 47.6, 34.1, 28.0, 27.3 ppm.

HRMS (ESI$^+$): m/z calc'd for $C_{23}H_{23}NO_3Na$ [M+Na]$^+$: 384.1570, found 384.1569.

Methyl (*Z*)-3-(2-cyclohexylethyl)-7-phenyl-10-oxa-8-azabicyclo[5.2.1]deca-3,8-diene-1-carboxylate (3ah) & methyl 7-(2-cyclohexylethyl)-2-phenyl-3a,4,5,8-tetrahydro-8a*H*-cyclohepta[*d*]oxazole-8a-carboxylate (3ah')

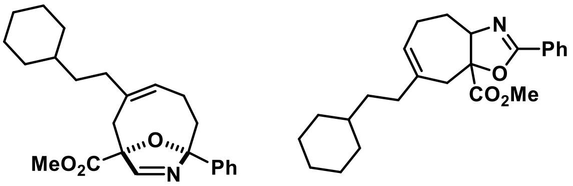

3ah **3ah'**

Synthesised following General Procedure F using: methyl 2-phenyloxazole-5-carboxylate **1r** (40.6 mg, 0.2 mmol, 1.0 eq.), (3-cyclopropylbut-3-en-1-yl)cyclohexane **2h** (71.3 mg, 0.4 mmol, 2.0 eq.), Ir-F (4.4 mg, 0.004 mmol, 2 mol%) and MeCN (2 mL). From crude ^{1}H NMR analysis, a crude yield of 64% and 80:20 regioisomeric ratio was determined. Purification *via* column chromatography on silica gel (*n*-pentane/EtOAc 100:0 – 80:20) afforded the title compound **3ah** (49.1 mg, 0.13 mmol, 64%) as colourless oil, as well as the (5+2) isomer **3ah'** (12.5 mg, 0.033 mmol, 16%) as a colourless oil.

<u>Characterization data for **3ah**:</u>

TLC: R_f **=** 0.3 (*n*-pentane/EtOAc 90:10).

NMR Spectroscopy (*see spectra*):

^{1}H NMR (599 MHz, $CDCl_3$): δ_H 7.59 – 7.56 (m, 2H), 7.35 – 7.32 (m, 2H), 7.27 – 7.24 (m, 1H), 7.19 (s, 1H), 5.62 – 5.58 (m, 1H), 3.81 (s, 3H), 2.94 (d, *J* = 14.9 Hz, 1H), 2.63 (d, *J* = 14.7 Hz, 1H), 2.50 – 2.41 (m, 2H), 2.12 – 2.06 (m, 1H), 2.03 – 1.97 (m, 1H), 1.95 – 1.85 (m, 2H), 1.74 – 1.67 (m, 4H), 1.67 – 1.61 (m, 1H), 1.30 – 1.11 (m, 6H), 0.94 – 0.83 (m, 2H) ppm;

^{13}C NMR (151 MHz, $CDCl_3$): δ_C 170.5, 157.1, 144.8, 137.7, 128.5, 127.7, 127.1, 124.9, 114.2, 96.0, 53.0, 40.3, 39.2, 37.6, 37.6, 35.8, 33.7, 33.4, 26.9, 26.6, 26.6, 24.2 ppm.

Characterization data for **3ah'**:

TLC: R_f **=** 0.2 (*n*-pentane/EtOAc 90:10).

NMR Spectroscopy (*see spectra*):

^{1}H NMR (599 MHz, $CDCl_3$): δ_H 8.00 – 7.95 (m, 2H), 7.51 – 7.47 (m, 1H), 7.44 – 7.39 (m, 2H), 5.30 – 5.27 (m, 1H), 4.78 (dd, *J* = 9.9, 3.1 Hz, 1H), 3.79 (s, 3H), 2.81 (d, *J* = 15.3 Hz, 1H), 2.78 (d, *J* = 16.3 Hz, 1H), 2.42 – 2.35 (m, 1H), 2.34 – 2.27 (m, 1H), 2.27 – 2.20 (m, 1H), 2.12 – 2.04 (m, 1H), 1.96 – 1.92 (m, 2H), 1.73 – 1.65 (m, 4H), 1.65 – 1.59 (m, 1H), 1.29 – 1.08 (m, 6H), 0.94 – 0.81 (m, 2H) ppm;

^{13}C NMR (151 MHz, $CDCl_3$): δ_C 172.0, 161.6, 132.8, 131.9, 128.7, 128.5, 127.1, 125.0, 90.1, 70.9, 52.9, 38.9, 37.5, 35.8, 34.4, 33.6, 33.4, 27.9, 27.3, 26.8, 26.5, 26.5 ppm.

HRMS (ESI$^+$): m/z calc'd for $C_{24}H_{31}NO_3Na$ $[M+Na]^+$: 404.2196, found 404.2197.

Methyl (*Z*)-3-(4-fluorobenzyl)-7-phenyl-10-oxa-8-azabicyclo[5.2.1]deca-3,8-diene-1-carboxylate (3ai) & methyl 7-(4-fluorobenzyl)-2-phenyl-3a,4,5,8-tetrahydro-8a*H*-cyclohepta[*d*]oxazole-8a-carboxylate (3ai')

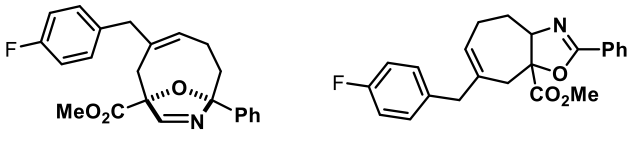

3ai **3ai'**

Synthesised following General Procedure F using: methyl 2-phenyloxazole-5-carboxylate **1r** (40.6 mg, 0.2 mmol, 1.0 eq.), 1-(2-cyclopropylallyl)-4-fluorobenzene **2i** (70.5 mg, 0.4 mmol, 2.0 eq.), Ir-F (4.0 mg, 0.004 mmol, 2 mol%) and MeCN (2 mL). From crude ^{1}H NMR analysis, a crude yield of 67% and 75:25 regioisomeric ratio was determined. Purification *via* column chromatography on silica gel (*n*-pentane/EtOAc 100:0 – 80:20) afforded the title compound **3ai** (48.8 mg, 0.13 mmol, 64%) as colourless oil, as well as the (5+2) isomer **3ai'** (14.7 mg, 0.039 mmol, 19%) as a colourless oil.

Characterization data for **3ai**:

TLC: R_f **=** 0.4 (*n*-pentane/EtOAc 80:20).

NMR Spectroscopy (*see spectra*):

^{1}H NMR (599 MHz, $CDCl_3$): δ_H 7.58 – 7.55 (m, 2H), 7.36 – 7.32 (m, 2H), 7.28 – 7.24 (m, 1H), 7.17 – 7.13 (m, 2H), 7.03 – 6.98 (m, 2H), 6.82 (s, 1H), 5.74 – 5.70 (m, 1H), 3.74 (s, 3H), 3.30 (d, *J* = 14.8 Hz, 1H), 3.24 (d, *J* = 14.7 Hz, 1H), 2.89 (d, *J* = 15.0 Hz, 1H), 2.62 (d, *J* = 15.0 Hz, 1H), 2.52 – 2.45 (m, 2H), 2.15 – 2.10

(m, 1H), 1.96 – 1.89 (m, 1H) ppm;

^{13}C{^{19}F} NMR (151 MHz, $CDCl_3$): δ_C 170.1, 161.8, 157.0, 144.5, 136.1, 134.5, 130.8, 129.7, 128.4, 127.7, 124.8, 115.3, 114.2, 95.5, 52.9, 45.7, 39.4, 39.2, 24.1 ppm;

^{13}C NMR (126 MHz, $CDCl_3$): δ_C 170.1, 161.8 (d, J = 244.6 Hz), 157.0, 144.5, 136.2, 134.5 (d, J = 3.3 Hz), 130.8 (d, J = 7.9 Hz), 129.7, 128.4, 127.7, 124.8, 115.4 (d, J = 21.2 Hz), 114.2, 95.5, 52.9, 45.8, 39.4, 39.3, 24.1 ppm;

^{19}F NMR (563 MHz, $CDCl_3$): δ_F -116.7 ppm.

Characterization data for **3ai'**:

TLC: R_f **=** 0.2 (*n*-pentane/EtOAc 80:20).

NMR Spectroscopy (*see spectra*):

^{1}H NMR (500 MHz, $CDCl_3$): δ_H 7.92 – 7.88 (m, 2H), 7.51 – 7.45 (m, 1H), 7.43 – 7.37 (m, 2H), 7.13 – 7.07 (m, 2H), 6.99 – 6.92 (m, 2H), 5.40 – 5.36 (m, 1H), 4.75 (dd, J = 9.8, 3.1 Hz, 1H), 3.72 (s, 3H), 3.25 (s, 2H), 2.76 (d, J = 15.5 Hz, 1H), 2.66 (d, J = 15.6 Hz, 1H), 2.48 – 2.40 (m, 1H), 2.33 – 2.25 (m, 1H), 2.12 – 2.02 (m, 1H), 1.26 (s, 1H) ppm;

^{13}C{^{19}F} NMR (126 MHz, $CDCl_3$): δ_C 172.1, 161.7, 161.4, 135.0, 131.7, 131.6, 130.6, 128.5, 128.5, 127.6, 127.4, 115.2, 89.7, 71.6, 52.9, 46.7, 34.0, 28.0, 27.3 ppm;

^{13}C NMR (126 MHz, $CDCl_3$): δ_C 172.1, 161.6 (d, J = 243.1 Hz), 161.4, 135.0, 131.7, 131.6, 130.6 (d, J = 7.9 Hz), 128.5, 128.5, 127.6, 127.4, 115.2 (d, J = 21.2 Hz), 89.7, 71.6, 52.9, 46.7, 34.0, 28.0, 27.3 ppm;

^{19}F NMR (470 MHz, $CDCl_3$): δ_F -117.2 ppm.

HRMS (ESI$^+$): m/z calc'd for $C_{23}H_{22}NO_3FH$ [M+H]$^+$: 380.1657, found 380.1658.

Methyl (*Z*)-3-(but-3-yn-1-yl)-7-phenyl-10-oxa-8-azabicyclo[5.2.1]deca-3,8-diene-1-carboxylate (3aj) & methyl 7-(but-3-yn-1-yl)-2-phenyl-3a,4,5,8-tetrahydro-8a*H*-cyclohepta[*d*]oxazole-8a-carboxylate (3aj')

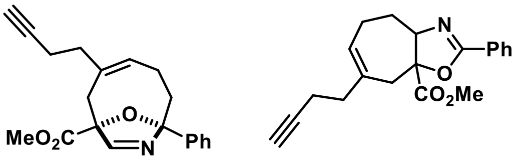

3aj 3aj'

Synthesized following General Procedure F using: methyl 2-phenyloxazole-5-carboxylate **1r** (40.6 mg, 0.2 mmol, 1.0 eq.), hex-1-en-5-yn-2-ylcyclopropane **2j** (96.0 mg, 0.5 mmol, 2.5 eq., 1:1 pentane/VCP), Ir-F (4.0 mg, 0.004 mmol, 2 mol%) and MeCN (2 mL). From crude ^{1}H NMR analysis, a crude yield of 70% and 79:21 regioisomeric ratio was determined. Purification *via* column chromatography on silica gel (*n*-pentane/EtOAc 100:0 – 88:12) afforded the title compound **3aj** (46.2 mg, 0.14 mmol, 71%) as a colourless oil, as well as the (5+2) regioisomer **3aj'** (12.0 mg, 0.04 mmol, 19%) as a colourless oil.

Characterization data for **3aj**:

TLC: R_f **=** 0.4 (90:10 *n*-pentane/EtOAc).

NMR Spectroscopy (***see spectra***):

^{1}H NMR (400 MHz, $CDCl_3$): δ_H 7.65 – 7.58 (m, 2H), 7.42 – 7.36 (m, 2H), 7.34 – 7.27 (m, 2H), 5.74 (t, *J* = 8.2 Hz, 1H), 3.86 (s, 3H), 3.01 (d, *J* = 14.9 Hz, 1H), 2.72 (d, *J* = 14.9 Hz, 1H), 2.59 – 2.45 (m, 2H), 2.41 – 2.08 (m, 5H), 2.03 (t, *J* = 2.5 Hz, 1H), 2.00 – 1.88 (m, 1H) ppm;

^{13}C NMR (101 MHz, $CDCl_3$): δ_C 170.2, 156.9, 144.4, 135.2, 128.9, 128.5, 128.4, 127.7, 124.8, 114.3, 95.6, 83.8, 69.1, 53.0, 39.9, 39.0, 38.8, 24.0, 17.6 ppm.

Characterization data for **3aj'**:

TLC: R_f **=** 0.3 (90:20 *n*-pentane/EtOAc).

NMR Spectroscopy (***see spectra***):

^{1}H NMR (400 MHz, $CDCl_3$): δ_H 8.03 – 7.93 (m, 2H), 7.53 – 7.46 (m, 1H), 7.46 – 7.38 (m, 2H), 5.38 (t, *J* = 4.1 Hz, 1H), 4.80 (dd, *J* = 9.5, 2.8 Hz, 1H), 3.80 (s, 3H), 2.82 (q, *J* = 15.7 Hz, 2H), 2.49 – 2.05 (m, 8H), 1.91 (t, *J* = 2.3 Hz, 1H) ppm;

^{13}C NMR (101 MHz, $CDCl_3$): δ_C 171.8, 131.9, 130.5, 128.6, 128.6, 127.0, 124.9, 89.9, 84.0, 71.0, 68.8, 53.0, 40.1, 34.1, 27.7, 27.2, 17.5 ppm.

HRMS (ESI$^+$): m/z calc'd for $C_{20}H_{21}NO_3Na$ [M+Na]$^+$: 346.1414, found 346.1413.

Dimethyl (*Z*)-1-(2-((2-(4-(2,2-dichlorocyclopropyl)phenoxy)-2-methylpropanoyl)oxy)acetyl)-10-oxabicyclo[5.2.1]deca-4,8-diene-2,2-dicarboxylate (3ak) & dimethyl (*Z*)-1-(2-((2-(4-(2,2-dichlorocyclopropyl)phenoxy)-2-methylpropanoyl)oxy)acetyl)-10-oxabicyclo[5.2.1]deca-4,8-diene-2,2-dicarboxylate (3ak')

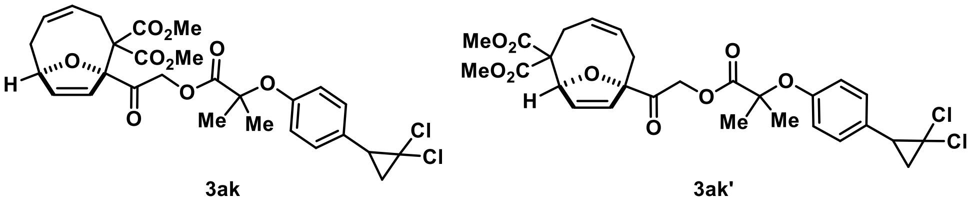

3ak 3ak'

Synthesized following General Procedure F using: 2-(furan-2-yl)-2-oxoethyl 2-(4-(2,2-dichlorocyclopropyl)phenoxy)-2-methylpropanoate **1ak** (79.2 mg, 0.2 mmol, 1.0 eq.), dimethyl 2-vinylcyclopropane-1,1-dicarboxylate **2a** (92.0 mg, 0.5 mmol, 2.5 eq.), Ir-F (4.0 mg, 0.004 mmol, 2 mol%) and MeCN (2 mL). From crude ^{1}H NMR analysis, a crude yield of 53% and 87:13 regioisomeric ratio was determined. Purification *via* column chromatography on silica gel (*n*-pentane/EtOAc 100:0 – 83:17) afforded the title compound **3ak** (51.3 mg, 0.088 mmol, 44%, 50:50 d.r.) as a colourless oil, as well as the minor regioisomer **3ak'** (8.5 mg, 0.02 mmol, 7%, 50:50 d.r.) as a colourless oil.

Characterization data for **3ak**:

TLC: R_f **=** 0.3 (85:15 *n*-pentane/EtOAc).

NMR Spectroscopy (*see spectra*):

^{1}H NMR (400 MHz, $CDCl_3$): δ_H 7.09 (d, J = 8.3 Hz, 2H), 6.88 (d, J = 8.8 Hz, 2H), 6.06 (dd, J = 5.9, 1.7 Hz, 1H), 5.97 (dd, J = 6.0, 1.6 Hz, 1H), 5.88 – 5.76 (m, 1H), 5.64 – 5.53 (m, 1H), 5.35 – 5.31 (m, 1H), 5.23 – 5.00 (m, 2H), 3.81 (s, 3H), 3.75 (d, J = 1.8 Hz, 3H), 2.87 – 2.77 (m, 1H), 2.75 – 2.61 (m, 2H), 2.45 – 2.35 (m, 1H), 2.31 – 2.20 (m, 1H), 1.98 – 1.86 (m, 1H), 1.81 – 1.72 (m, 1H), 1.64 (d, J = 3.6 Hz, 6H) ppm;

^{13}C NMR (101 MHz, $CDCl_3$): δ_C 201.7, 173.7, 170.6, 169.3, 154.9, 135.1, 129.7, 129.5, 128.5, 128.2, 127.2, 119.3 and 119.3, 99.0, 85.0, 79.3, 67.1, 66.2, 61.0, 52.9, 52.9, 35.0, 33.0, 31.8, 26.0, 25.8 and 25.8, 25.5 and 25.5 ppm.

Characterization data for **3ak'**:

TLC: R_f **=** 0.4 (85:15 *n*-pentane/EtOAc).

NMR Spectroscopy (*see spectra*):

^{1}H NMR (400 MHz, $CDCl_3$): δ_H 7.16 – 7.12 (m, 2H), 6.93 – 6.89 (m, 2H), 6.03 – 5.95 (m, 1H), 5.89 (dd, J = 6.0, 1.8 Hz, 1H), 5.72 (t, J = 2.1 Hz, 1H), 5.55 (dd, J = 6.0, 2.3 Hz, 1H), 5.46 – 5.39 (m, 1H), 5.25 – 5.09 (m, 2H), 3.79 (s, 3H), 3.77 (s, 3H), 2.86 – 2.80 (m, 1H), 2.79 – 2.72 (m, 1H), 2.71 – 2.64 (m, 1H), 2.63 – 2.57 (m, 2H), 1.96 – 1.90 (m, 1H), 1.81 – 1.75 (m, 1H), 1.66 (s, 6H) ppm;

^{13}C NMR (151 MHz, $CDCl_3$): δ_C 203.4, 173.9, 171.2, 169.0, 154.9, 131.0, 129.8, 129.4, 129.4, 128.4, 127.5, 119.2, 100.6, 86.9, 79.2, 67.1, 63.0, 61.0 and 61.0, 53.3, 52.8, 35.1, 35.0, 29.0, 26.0, 25.7 and 25.7, 25.6 and 25.6 ppm.

HRMS (ESI$^+$): m/z calc'd for $C_{28}H_{30}Cl_2O_9Na$ $[M+Na]^+$: 603.1159, found 603.1161.

Dimethyl (*Z*)-1-((*R*)-4-((3*S*,5*R*,8*S*,9*S*,10*S*,13*R*,14*S*,17*S*)-3-acetoxy-10,13-dimethylhexadecahydro-1*H*-cyclopenta[*a*]phenanthren-17-yl)pentanoyl)-10-oxabicyclo[5.2.1]deca-4,8-diene-2,2-dicarboxylate (3al) & dimethyl (*Z*)-7-((*R*)-4-((3*S*,5*R*,8*S*,9*S*,10*S*,13*R*,14*S*,17*S*)-3-acetoxy-10,13-dimethylhexadecahydro-1*H*-cyclopenta[*a*]phenanthren-17-yl)pentanoyl)-10-oxabicyclo[5.2.1]deca-4,8-diene-2,2-dicarboxylate (3al')

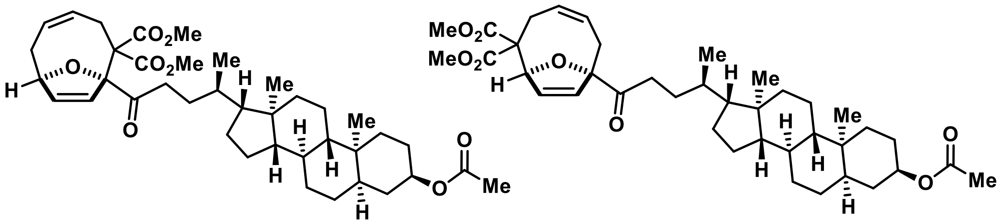

3al 3al'

Synthesized following General Procedure F using: (3*S*,5*R*,8*S*,9*S*,10*S*,13*R*,14*S*,17*S*)-17-((*R*)-5-(furan-2-yl)-5-oxopentan-2-yl)-10,13-dimethylhexadecahydro-1*H*-cyclopenta[*a*]phenanthren-3-yl acetate **1al** (93.7 mg, 0.2 mmol, 1.0 eq.), dimethyl 2-vinylcyclopropane-1,1-dicarboxylate **2a** (92.0 mg, 0.5 mmol, 2.5 eq.), Ir-F (4.0 mg, 0.004 mmol, 2 mol%) and MeCN (4 mL). From crude ^{1}H NMR analysis, a crude yield of 52% and 80:20 regioisomeric ratio was determined. Purification *via* column chromatography on silica gel (*n*-pentane/EtOAc 100:0 – 93:7) afforded the title compound **3al** (53.6 mg, 0.082 mmol, 41%, 50:50 d.r.) as a colourless oil, as well as the minor regioisomer **3al'** (13.2 mg, 0.02 mmol, 10%, 50:50 d.r.) as a colourless oil.

Characterization data for **3al**:

TLC: R_f **=** 0.3 (97:3 *n*-pentane/EtOAc).

NMR Spectroscopy (*see spectra*):

^{1}H NMR (400 MHz, $CDCl_3$): δ_H 6.01 (d, *J* = 6.0 Hz, 1H), 5.93 – 5.87 (m, 1H), 5.86 – 5.74 (m, 1H), 5.64 – 5.53 (m, 1H), 5.32 (s, 1H), 4.77 – 4.64 (m, 1H), 3.79 (s, 3H), 3.74 (s, 3H), 2.86 – 2.48 (m, 4H), 2.42 – 2.30 (m, 1H), 2.29 – 2.18 (m, 1H), 2.02 (s, 3H), 1.97 – 1.88 (m, 1H), 1.87 – 1.74 (m, 5H), 1.71 – 1.61 (m, 2H), 1.57 – 1.49 (m, 2H), 1.47 – 1.30 (m, 8H), 1.28 – 1.19 (m, 4H), 1.12 – 0.99 (m, 2H), 0.90 (s, 3H), 0.88 (d, *J* = 1.8 Hz, 1.5H, d_1), 0.87 (d, *J* = 1.7 Hz, 1.5H, d_2), 0.62 (s, 3H) ppm;

^{13}C NMR (101 MHz, $CDCl_3$): δ_C 211.4, 170.8, 170.0, 134.7, 129.8, 128.4, 127.9 and 127.8, 100.1 and 100.0, 90.3, 84.6, 74.5, 67.0, 56.6 and 56.6, 56.2 and 56.1, 52.8 and 52.6, 42.8, 42.0, 40.5, 40.3, 35.9, 35.5 and 35.4, 35.2, 34.7, 33.6, 33.4, 33.1, 32.4, 32.2, 29.6 and 29.4, 28.3 and 28.2, 27.1, 26.8, 26.4, 24.3, 23.5, 21.6, 21.0, 18.7 and 18.6, 12.2 and 12.2 ppm.

Characterization data for **3al'**:

TLC: R_f **=** 0.4 (97:3 *n*-pentane/EtOAc).

NMR Spectroscopy (*see spectra*):

^{1}H NMR (400 MHz, $CDCl_3$): δ_H 6.03 – 5.92 (m, 1H), 5.85 – 5.78 (m, 1H), 5.72 (s, 1H), 5.52 – 5.47 (m, 1H), 5.47 – 5.38 (m, 1H), 4.77 – 4.66 (m, 1H), 3.79 (s, 3H), 3.77 (s, 3H), 2.77 – 2.48 (m, 6H), 2.03 (s, 3H), 1.92 – 1.76 (m, 5H), 1.76 – 1.62 (m, 6H), 1.51 – 1.35 (m, 9H), 1.22 – 0.98 (m, 6H), 0.92 (s, 3H), 0.91 (d, *J* = 3.1 Hz, 1.5H, d_1), 0.89 (d, *J* = 3.1 Hz, 1.5H. d_2), 0.64 (d, *J* = 2.1 Hz, 3H) ppm;

^{13}C NMR (151 MHz, $CDCl_3$): δ_C 212.5 and 212.4, 171.4, 170.8, 169.2, 131.9 and 131.8, 130.2 and 130.1, 129.0 and 129.0, 128.7 and 128.7, 101.3 and 101.2, 86.7 and 86.7, 74.6, 63.2 and 63.1, 56.7, 56.2 and 56.2, 53.3, 52.7, 42.9 and 42.9, 42.1, 40.6 and 40.6, 40.3 and 40.3, 36.0, 35.6 and 35.5, 35.4 and 35.4, 35.2, 35.0 and 34.8, 34.7, 32.4, 29.7 and 29.6, 29.0, 28.4 and 28.3, 27.2, 26.8, 26.5, 24.4 and 24.4, 23.5, 21.6, 21.0, 18.7 and 18.6, 12.2 ppm.

HRMS (ESI^+): m/z calc'd for $C_{39}H_{56}O_8Na$ $[M+Na]^+$: 675.3867, found 675.3868.

Dimethyl (*Z*)-1-(2-((5-(2,5-dimethylphenoxy)-2,2-dimethylpentanoyl)oxy)acetyl)-10-oxabicyclo[5.2.1]deca-4,8-diene-2,2-dicarboxylate (3am) & dimethyl (*Z*)-7-(2-((5-(2,5-dimethylphenoxy)-2,2-dimethylpentanoyl)oxy)acetyl)-10-oxabicyclo[5.2.1]deca-4,8-diene-2,2-dicarboxylate (3am')

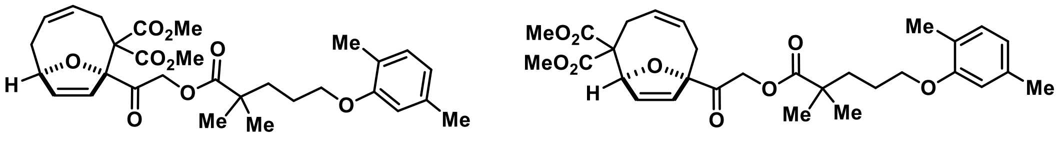

3am **3am'**

Synthesized following General Procedure F using: 2-(furan-2-yl)-2-oxoethyl 5-(2,5-dimethylphenoxy)-2,2-dimethylpentanoate **1am** (71.6 mg, 0.2 mmol, 1.0 eq.), dimethyl 2-vinylcyclopropane-1,1-dicarboxylate **2a** (92.0 mg, 0.5 mmol, 2.5 eq.), Ir-F (4.0 mg, 0.004 mmol, 2 mol%) and MeCN (2 mL). From crude ^{1}H NMR analysis, a crude yield of 54% and 87:13 regioisomeric ratio was determined. Purification *via* column

chromatography on silica gel (*n*-pentane/EtOAc 100:0 – 85:15) afforded the major regioisomer **3am** (49.0 mg, 0.09 mmol, 45%) as a white solid, as well as the minor regioisomer **3am'** (10.2 mg, 0.02 mmol, 9%) as a colourless oil.

Characterization data for **3am**:

TLC: R_f **=** 0.3 (85:15 *n*-pentane/EtOAc).

NMR Spectroscopy (***see spectra***):

^{1}**H NMR** (400 MHz, $CDCl_3$): δ_H 6.99 (d, *J* = 7.4 Hz, 1H), 6.64 (d, *J* = 7.5 Hz, 1H), 6.60 (s, 1H), 6.07 (dd, *J* = 6.0, 1.6 Hz, 1H), 5.98 (dd, *J* = 5.9, 1.6 Hz, 1H), 5.88 – 5.76 (m, 1H), 5.64 – 5.54 (m, 1H), 5.36 – 5.28 (m, 1H), 5.07 – 4.96 (m, 2H), 3.97 – 3.88 (m, 2H), 3.77 (s, 3H), 3.77 (s, 3H), 2.73 – 2.61 (m, 2H), 2.43 – 2.36 (m, 1H), 2.30 (s, 3H), 2.28 – 2.21 (m, 1H), 2.16 (s, 3H), 1.85 – 1.70 (m, 4H), 1.25 (d, *J* = 5.2 Hz, 6H) ppm;

^{13}**C NMR** (151 MHz, $CDCl_3$): δ_C 202.2, 177.4, 170.7, 169.4, 157.2, 136.5, 135.0, 130.4, 129.6, 128.5, 127.3, 123.7, 120.7, 112.1, 99.2, 84.9, 68.2, 67.1, 65.5, 56.1, 52.9, 42.2, 37.1, 33.0, 31.9, 25.3, 25.3, 25.1, 21.5, 15.9 ppm.

Characterization data for **3am'**:

TLC: R_f **=** 0.4 (85:15 *n*-pentane/EtOAc).

NMR Spectroscopy (***see spectra***):

^{1}**H NMR** (400 MHz, $CDCl_3$): δ_H 7.00 (d, *J* = 7.4 Hz, 2H, **3aj'** and **A**), 6.65 (d, *J* = 7.6 Hz, 2H, **3aj'** and **A**), 6.61 (d, *J* = 5.5 Hz, 2H, **3aj'** and **A**), 5.99 (q, *J* = 8.7 Hz, 1H), 5.88 (dd, *J* = 6.0, 1.8 Hz, 1H), 5.72 (t, *J* = 2.0 Hz, 1H), 5.55 (dd, *J* = 6.0, 2.2 Hz, 1H), 5.48 – 5.35 (m, 1H), 5.13 – 4.98 (m, 2H), 3.98 – 3.90 (m, 4H, **3aj'** and **A**), 3.79 (s, 3H), 3.77 (s, 3H), 2.80 – 2.64 (m, 2H), 2.63 – 2.54 (m, 2H), 2.30 (s, 7H, **3aj'** and **A**), 2.17 (s, 6H, **3aj'** and **A**), 1.88 – 1.68 (m, 8H, **3aj'** and **A**), 1.26 (d, *J* = 8.4 Hz, 13H, **3aj'** and **A**) ppm;

^{13}**C NMR** (151 MHz, $CDCl_3$): δ_C 203.9 (**3aj'**), 182.9 (**A**), 177.4 (**3aj'**), 171.2 (**3aj'**), 169.0 (**3aj'**), 157.2 and 157.1 (**3aj'** and **A**), 136.6 and 136.6 (**3aj'** and **A**), 131.2 (**3aj'**), 130.4 and 130.4 (**3aj'** and **A**), 129.5 (**3aj'**), 129.3 (**3aj'**), 129.2 (**3aj'**), 123.8 and 123.8 (**3aj'** and **A**), 120.9 and 120.8 (**3aj'** and **A**), 112.2 and 112.1 (**3aj'** and **A**), 86.9 (**3aj'**), 68.2 and 68.0 (**3aj'** and **A**), 66.3 (**3aj'**), 63.0 (**3aj'**), 53.3 (**3aj'**), 52.7 (**3aj'**), 42.3 and 42.0 (**3aj'** and **A**), 37.3 and 37.1 (**3aj'** and **A**), 35.0 (**3aj'**), 29.0 (**3aj'**), 25.4 and 25.3 (**3aj'** and **A**), 25.2 and 25.2 (**3aj'** and **A**), 21.5 and 21.5 (**3aj'** and **A**), 15.9 and 15.9 (**3aj'** and **A**) ppm.

Note: The minor regioisomer contains gemfibrozil (**A**) as a 1:1 impuritiy, which could not be separated from the product, presumably an artefact from the synthesis of **1am**. All the integrals resulting from gemfibrzozil thus integrate double. ^{13}C-peaks were assigned *via* 2D-^{1}H NMR.

HRMS (ESI$^+$): m/z calc'd for $C_{30}H_{38}O_9Na$ $[M+Na]^+$: 565.2408, found 565.2403.

Dimethyl (*Z*)-1-(2-(((1*S*)-4,7,7-trimethyl-3-oxo-2-oxabicyclo[2.2.1]heptane-1-carbonyl)oxy)acetyl)-10-oxabicyclo[5.2.1]deca-4,8-diene-2,2-dicarboxylate & dimethyl (3an) (*Z*)-7-(2-(((1*S*)-4,7,7-trimethyl-3-oxo-2-oxabicyclo[2.2.1]heptane-1-carbonyl)oxy)acetyl)-10-oxabicyclo[5.2.1]deca-4,8-diene-2,2-dicarboxylate (3an')

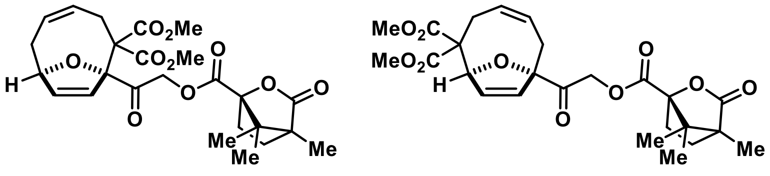

Synthesized following General Procedure F using: 2-(furan-2-yl)-2-oxoethyl (1*S*)-4,7,7-trimethyl-3-oxo-2-oxabicyclo[2.2.1]heptane-1-carboxylate **1an** (61.2 mg, 0.2 mmol, 1.0 eq.), dimethyl 2-vinylcyclopropane-1,1-dicarboxylate **2a** (92.0 mg, 0.5 mmol, 2.5 eq.), Ir-F (4.0 mg, 0.004 mmol, 2 mol%) and MeCN (2 mL). From crude ^{1}H NMR analysis, a crude yield of 70% and 84:16 regioisomeric ratio was determined. Purification *via* column chromatography on silica gel (*n*-pentane/EtOAc 100:0 – 70:30) afforded the title compound were employed to obtain the major regioisomer **3an** (56.0 mg, 0.11 mmol, 57%, 50:50 d.r.) as a colourless oil, as well as the minor regioisomer **3an'** (13.2 mg, 0.03 mmol, 13%, 50:50 d.r.) as a colourless oil.

<u>Characterization data for **3an**:</u>

TLC: R_f **=** 0.3 (70:30 *n*-pentane/EtOAc).

NMR Spectroscopy (*see spectra*):

^{1}H NMR (400 MHz, $CDCl_3$): δ_H 6.08 – 6.03 (m, 1H), 5.97 – 5.91 (m, 1H), 5.83 – 5.75 (m, 1H), 5.60 – 5.52 (m, 1H), 5.34 – 5.29 (m, 1H), 5.26 (d, *J* = 17.1 Hz, 0.5H, d_1), 5.21 – 5.08 (m, 1H, d_2), 5.04 (d, *J* = 17.1 Hz, 0.5H, d_1), 3.76 (d, *J* = 2.4 Hz, 3H), 3.73 (d, *J* = 3.5 Hz, 3H), 2.71 – 2.60 (m, 2H), 2.52 – 2.34 (m, 2H), 2.27 – 2.18 (m, 1H), 2.09 – 1.99 (m, 1H), 1.93 – 1.85 (m, 1H), 1.70 – 1.61 (m, 1H), 1.08 (s, 3H), 1.08 – 0.99 (m, 6H) ppm;

^{13}C NMR (101 MHz, $CDCl_3$): δ_C 200.9 and 200.9, 178.2 and 178.2, 170.6 and 170.5, 169.3 and 169.3, 167.0 and 166.9, 135.2 and 135.1, 129.4, 128.5, 127.2 and 127.1, 99.0, 91.3 and 91.2, 85.1, 67.2 and 67.1, 66.5 and 66.4, 55.0 and 55.0, 54.5 and 54.5, 52.9 and 52.9, 52.8, 33.0 and 32.9, 31.8 and 31.8, 30.9 and 30.8, 29.1 and 29.0, 16.8 and 16.7, 16.6, 9.9 and 9.8 ppm.

<u>Characterization data for **3an'**:</u>

TLC: R_f **=** 0.4 (70:30 *n*-pentane/EtOAc).

NMR Spectroscopy (*see spectra*):

^{1}H NMR (400 MHz, $CDCl_3$): δ_H 6.05 – 5.94 (m, 1H), 5.91 (dd, *J* = 6.0, 1.8 Hz, 1H), 5.76 – 5.70 (m, 1H), 5.58 – 5.51 (m, 1H), 5.48 – 5.37 (m, 1H), 5.32 (d, *J* = 17.8 Hz, 0.5H, d_1), 5.29 – 5.17 (m, 1H, d_2), 5.14 (d, *J* = 17.8 Hz, 0.5H, d_1), 3.79 (s, 3H), 3.77 (s, 3H), 2.81 – 2.56 (m, 4H), 2.54 – 2.42 (m, 1H), 2.14 – 2.03 (m, 1H), 1.99 – 1.88 (m, 1H), 1.76 – 1.65 (m, 1H), 1.14 – 1.11 (m, 6H), 1.11 – 1.08 (m, 3H) ppm;

^{13}C NMR (101 MHz, $CDCl_3$): δ_C 202.8 and 202.8, 178.2, 171.2, 168.9, 167.1 and 167.0, 130.9 and 130.9, 129.6 and 129.6, 129.5, 129.3, 100.5, 91.2, 87.0 and 87.0, 68.1, 67.3 and 67.2, 63.0 and 62.9, 55.1, 54.7

and 54.7, 53.3, 52.8, 35.2, 35.0, 30.9, 29.1 and 29.1, 25.8, 16.7 and 16.7, 9.9 ppm.

HRMS (ESI$^+$): m/z calc'd for $C_{25}H_{30}O_{10}Na$ [M+Na]$^+$: 513.1731, found 513.1738.

Dimethyl (*Z*)-1-(2-((4-(*N*,*N*-dipropylsulfamoyl)benzoyl)oxy)acetyl)-10-oxabicyclo[5.2.1]deca-4,8-diene-2,2-dicarboxylate (3ao) & dimethyl (*Z*)-7-(2-((4-(*N*,*N*-dipropylsulfamoyl)benzoyl)oxy)acetyl)-10-oxabicyclo[5.2.1]deca-4,8-diene-2,2-dicarboxylate (3ao')

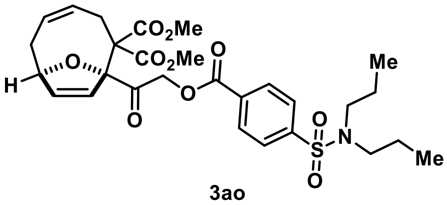

3ao

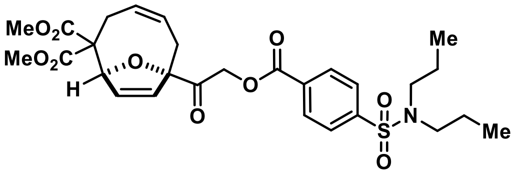

3ao'

Synthesized following General Procedure F using: 2-(furan-2-yl)-2-oxoethyl 4-(*N*,*N*-dipropylsulfamoyl)benzoate **1ao** (78.6 mg, 0.2 mmol, 1.0 eq.), dimethyl 2-vinylcyclopropane-1,1-dicarboxylate **2a** (92.0 mg, 0.5 mmol, 2.5 eq.), Ir-F (4.0 mg, 0.004 mmol, 2 mol%) and MeCN (2 mL). From crude ^{1}H NMR analysis, a crude yield of 57% and 81:19 regioisomeric ratio was determined. Purification *via* column chromatography on silica gel (*n*-pentane/EtOAc 100:0 – 80:20) afforded the major regioisomer **3ao** (53.6 mg, 0.09 mmol, 46%) as a white solid, as well as the minor regioisomer **3ao'** (12.6 mg, 0.02 mmol, 11%) as a colourless oil.

Characterization data for **3ao**:

TLC: R_f **=** 0.3 (80:20 *n*-pentane/EtOAc).

NMR Spectroscopy (***see spectra***):

^{1}H NMR (400 MHz, $CDCl_3$): δ_H 8.19 (d, *J* = 8.5 Hz, 2H), 7.85 (d, *J* = 8.5 Hz, 2H), 6.15 (dd, *J* = 5.9, 1.7 Hz, 1H), 5.99 (dd, *J* = 5.9, 1.6 Hz, 1H), 5.90 – 5.78 (m, 1H), 5.67 – 5.57 (m, 1H), 5.42 – 5.24 (m, 3H), 3.80 (s, 3H), 3.77 (s, 3H), 3.11 – 3.05 (m, 4H), 2.77 – 2.58 (m, 2H), 2.45 – 2.34 (m, 1H), 2.33 – 2.21 (m, 1H), 1.59 – 1.46 (m, 4H), 0.86 (t, *J* = 7.4 Hz, 6H) ppm;

^{13}C NMR (101 MHz, $CDCl_3$): δ_C 202.2, 170.7, 169.3, 164.8, 144.5, 135.5, 133.1, 130.7, 129.7, 128.4, 127.0, 126.9, 99.2, 84.8, 67.4, 66.7, 53.0, 50.1, 50.0, 32.9, 31.8, 22.0, 11.3 ppm.

Characterization data for **3ao'**:

TLC: R_f **=** 0.4 (80:20 *n*-pentane/EtOAc).

NMR Spectroscopy (***see spectra***):

^{1}H NMR (400 MHz, $CDCl_3$): δ_H 8.23 – 8.16 (d, *J* = 8.4 Hz, 2H), 7.89 (d, *J* = 8.4 Hz, 2H), 6.07 – 5.96 (m, 1H), 5.94 (dd, *J* = 6.1, 1.8 Hz, 1H), 5.78 (t, *J* = 2.1 Hz, 1H), 5.58 (dd, *J* = 6.0, 2.2 Hz, 1H), 5.49 – 5.26 (m, 3H), 3.80 (s, 3H), 3.78 (s, 3H), 3.14 – 3.05 (m, 4H), 2.82 – 2.58 (m, 4H), 1.62 – 1.48 (m, 1.62 – 1.48, 4H), 0.87 (t, *J* = 7.4 Hz, 6H) ppm;

^{13}C NMR (101 MHz, $CDCl_3$): δ_C 203.5, 171.2, 169.0, 164.8, 144.7, 132.5, 131.0, 130.7, 129.6, 129.5, 129.4, 127.2, 100.6, 87.0, 67.4, 63.0, 53.4, 52.8, 50.1, 35.1, 29.0, 22.1, 11.3 ppm.

HRMS (ESI$^+$): m/z calc'd for $C_{28}H_{35}NO_{10}SNa$ [M+Na]$^+$: 600.1874, found 600.1875.

Dimethyl (*Z*)-1-(2-(((2*S*,5*S*)-3,3-dimethyl-4,4-dioxido-7-oxo-4-thia-1-azabicyclo[3.2.0]heptane-2-carbonyl)oxy)acetyl)-10-oxabicyclo[5.2.1]deca-4,8-diene-2,2-dicarboxylate (3ap) & dimethyl (Z)-7-(2-(((2*S*,5*S*)-3,3-dimethyl-4,4-dioxido-7-oxo-4-thia-1-azabicyclo[3.2.0]heptane-2-carbonyl)oxy)acetyl)-10-oxabicyclo[5.2.1]deca-4,8-diene-2,2-dicarboxylate (3ap')

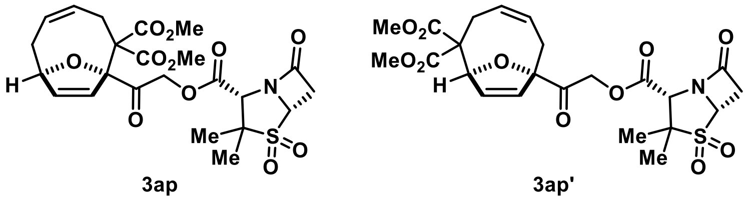

3ap **3ap'**

Synthesized following General Procedure F using: 2-(furan-2-yl)-2-oxoethyl (2*S*,5*S*)-3,3-dimethyl-7-oxo-4-thia-1-azabicyclo[3.2.0]heptane-2-carboxylate 4,4-dioxide **1ap** (68.2 mg, 0.2 mmol, 1.0 eq.), dimethyl 2-vinylcyclopropane-1,1-dicarboxylate **2a** (92.0 mg, 0.5 mmol, 2.5 eq.), Ir-F (4.0 mg, 0.004 mmol, 2 mol%) and MeCN (2 mL). From crude ^{1}H NMR analysis, a crude yield of 52% and 85:15 regioisomeric ratio was determined. Purification *via* column chromatography on silica gel (*n*-pentane/EtOAc 100:0 – 60:40) afforded the major regioisomer **3ap** (42.3 mg, 0.081 mmol, 40%, 50:50 d.r.) as a white solid, as well as the minor regioisomer **3ap'** (9.8 mg, 0.019 mmol, 9%, 50:50 d.r.) as a colourless oil.

Characterization data for **3ap**:

TLC: R_f **=** 0.3 (60:40 *n*-pentane/EtOAc).

NMR Spectroscopy (*see spectra*):

^{1}**H NMR** (400 MHz, $CDCl_3$): δ_H 6.13 – 6.04 (m, 1H), 5.99 – 5.88 (m, 1H), 5.86 – 5.75 (m, 1H), 5.65 – 5.52 (m, 1H), 5.45 (dd, *J* = 17.3, 1.4 Hz, 0.5H, d_1), 5.36 – 5.28 (m, 1H), 5.24 – 5.08 (m, 1H, d_2), 4.89 (dd, *J* = 17.3, 1.4 Hz, 0.5H, d_1), 4.65 – 4.58 (m, 1H), 4.42 (dd, *J* = 7.5, 1.4 Hz, 1H), 3.78 (d, *J* = 3.6 Hz, 3H), 3.76 (d, *J* = 8.3 Hz, 3H), 3.51 – 3.36 (m, 2H), 2.75 – 2.56 (m, 2H), 2.44 – 2.33 (m, 1H), 2.29 – 2.20 (m, 1H), 1.64 (d, *J* = 10.6 Hz, 3H), 1.54 (d, *J* = 12.1 Hz, 3H) ppm;

^{13}**C NMR** (101 MHz, $CDCl_3$): δ_C 201.7 and 201.3, 171.1 and 170.9, 170.7 and 170.6, 169.2, 166.7 and 166.6, 135.4, 129.5 and 129.4, 128.5, 126.9 and 126.7, 99.0 and 98.9, 85.2 and 84.8, 67.4 and 67.2, 67.1 and 67.0, 63.4, 63.3 and 63.3, 61.0 and 60.9, 53.0 and 53.0, 53.0 and 52.9, 38.2 and 38.0, 32.9 and 32.9, 31.8 and 31.7, 20.3 and 20.1, 18.3 and 18.0 ppm.

Characterization data for **3ap'**:

TLC: R_f **=** 0.4 (60:40 *n*-pentane/EtOAc).

NMR Spectroscopy (*see spectra*):

^{1}**H NMR** (400 MHz, $CDCl_3$): δ_H 6.05 – 5.96 (m, 1H), 5.95 – 5.90 (m, 1H), 5.77 – 5.70 (m, 1H), 5.53 (dd, *J* = 5.9, 2.8 Hz, 1H), 5.49 – 5.38 (m, 1.5H, d_1), 5.30 (d, *J* = 18.3 Hz, 0.5H, d_2), 5.12 (d, *J* = 17.8 Hz, 0.5H, d_2), 4.99 (d, *J* = 17.9 Hz, 0.5H, d_1), 4.67 – 4.61 (m, 1H), 4.46 (d, *J* = 1.1 Hz, 1H), 3.79 (s, 3H), 3.77 (s, 3H), 3.48 – 3.43 (m, 2H), 2.77 – 2.48 (m, 6H), 1.71 – 1.65 (m, 3H), 1.62 (s, 3H) ppm;

^{13}C NMR (151 MHz, $CDCl_3$): δ_C 203.2 and 203.0, 171.1, 171.0, 168.9, 166.7 and 166.7, 130.6, 129.9, 129.6 and 129.6, 129.2 and 129.1, 87.1 and 87.0, 67.7 and 67.7, 63.4 and 63.3, 63.3 and 63.3, 62.9 and 62.9, 61.0 and 61.0, 53.4, 52.8, 38.1 and 38.1, 35.2, 29.8, 29.0, 20.2 and 20.2, 18.1 and 18.0 ppm.

HRMS (ESI$^+$): m/z calc'd for $C_{23}H_{27}NO_{11}SNa$ [M+Na]$^+$: 548.1197, found 548.1198.

Dimethyl (*Z*)-1-(5-((3a*S*,4*S*,6a*R*)-2-oxohexahydro-1*H*-thieno[3,4-*d*]imidazol-4-yl)pentanoyl)-10-oxabicyclo[5.2.1]deca-4,8-diene-2,2-dicarboxylate (3aq) & dimethyl (*Z*)-7-(5-((3a*S*,4*S*,6a*R*)-2-oxohexahydro-1*H*-thieno[3,4-*d*]imidazol-4-yl)pentanoyl)-10-oxabicyclo[5.2.1]deca-4,8-diene-2,2-dicarboxylate (3aq')

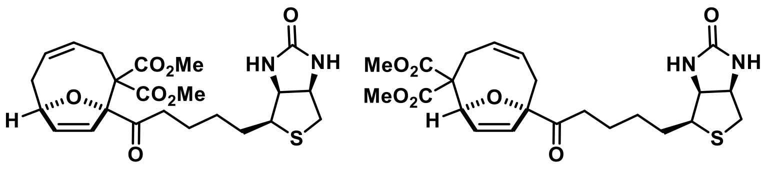

3aq 3aq'

Synthesized following modified General Procedure F using: (3a*S*,4*S*,6a*R*)-4-(5-(furan-2-yl)-5-oxopentyl)tetrahydro-1*H*-thieno[3,4-*d*]imidazol-2(3*H*)-one **1aq** (58.8 mg, 0.2 mmol, 1.0 eq.), dimethyl 2-vinylcyclopropane-1,1-dicarboxylate **2a** (92.0 mg, 0.5 mmol, 2.5 eq.), thioxanthone (4.2 mg, 0.02 mmol, 10 mol%) and CH_2Cl_2 (4 mL) (reaction irradiated at 405 nm). From crude ^{1}H NMR analysis, a crude yield of 42% and 81:19 regioisomeric ratio was determined. Purification *via* column chromatography on silica gel (CH_2Cl_2/MeOH 100:0 – 94:4) afforded the title compound as a mixture of regioisomers **3aq** & **3aq'** (33.7 mg, 0.07 mmol, 35%, 60:40 d.r..) as a colorless oil.

TLC: R_f **=** 0.4 (96:4 CH_2Cl_2/MeOH).

NMR Spectroscopy (***see spectra***):

^{1}H NMR (400 MHz, $CDCl_3$): δ_H 6.14 – 6.07 (m, 0.3H), 6.05 – 5.94 (m, 0.7H), 5.92 – 5.84 (m, 0.3H), 5.83 – 5.73 (m, 0.7H), 5.71 – 5.68 (m, 0.3H), 5.65 – 5.52 (m, 0.7H), 5.51 – 5.38 (m, 1H), 5.33 (d, *J* = 13.6 Hz, 0.7H), 5.22 – 4.93 (m, 2H), 4.53 – 4.44 (m, 1H), 4.35 – 4.25 (m, 1H), 3.86 – 3.61 (m, 6H), 3.19 – 3.08 (m, 1H), 3.04 – 2.81 (m, 1H), 2.78 – 2.65 (m, 2H), 2.49 – 2.04 (m, 5H), 1.71 – 1.47 (m, 5H), 1.45 – 1.30 (m, 2H) ppm;

^{13}C NMR (151 MHz, $CDCl_3$): δ_C 211.8 and 211.2, 210.5 and 208.4, 173.2 and 173.2, 171.6 and 171.6, 170.6 and 170.5, 170.5, 169.9, 163. and 163.3, 138.6 and 138.6, 135.0, 134.1 and 134.0, 131.3, 130.3 and 129.9, 129.8 and 129.6, 128.8 and 128.6, 127.6, 116.8, 99.9 and 99.8, 87.0 and 86.9, 67.4 and 67.3, 64.6, 62.1 and 62.0, 61.7 and 61.7, 60.2 and 60.1, 55.6 and 55.6, 55.5, 53.0 and 52.9, 52.8 and 52.8, 52.7 and 52.7, 52.5, 49.1 and 49.1, 48.4 and 48.4, 44.7 and 44.7, 40.8, 40.7 and 40.7, 39.6 and 39.5, 37.5 and 37.4, 36.6, 36.4, 36.1 and 36.0, 36.0, 35.6, 35.3, 33.1, 32.2, 29.8, 29.0, 28.6 and 28.5, 28.5 and 28.5, 28.1 and 28.1, 28.0 and 27.9, 23.4 and 23.3, 23.2 and 23.0, 22.9 and 22.7 ppm.

HRMS (ESI$^+$): m/z calc'd for $C_{23}H_{30}N_2O_7SNa$ [M+Na]$^+$: 501.1666, found 501.1668.

2.4. Product Diversification

Dimethyl 1-propionyl-10-oxabicyclo[5.2.1]decane-2,2-dicarboxylate (4a)

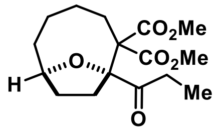

4a

Dimethyl (*Z*)-1-propionyl-10-oxabicyclo[5.2.1]deca-4,8-diene-2,2-dicarboxylate **3b** (30.8 mg, 0.1 mmol, 1.0 eq.) and Pd/C (10% Pd, 10.0 mg, 0.01 mmol, 10 mol%) were dissolved in MeOH (0.1 M) under argon. A hydrogen-filled balloon was attached and the reaction vessel purged with H_2 for 10 min. The reaction mixture was stirred overnight at rt under H_2-atmosphere. Then, the reaction mixture was diluted with CH_2Cl_2, filtered and concentrated to yield the pure title compound **4a** (31.1 mg, 0.1 mmol, quant.) as a colourless oil.

TLC: R_f **=** 0.3 (90:10 *n*-pentane/EtOAc).

NMR Spectroscopy (***see spectra***):

^{1}**H NMR** (400 MHz, $CDCl_3$): δ_H 4.74 – 4.67 (m, 1H), 3.74 (s, 3H), 3.73 (s, 3H), 3.16 – 2.94 (m, 2H), 2.54 – 2.42 (m, 1H), 2.37 – 2.23 (m, 1H), 2.16 – 1.74 (m, 7H), 1.54 – 1.33 (m, 3H), 1.05 (t, *J* = 7.2 Hz, 3H) ppm;

^{13}**C NMR** (101 MHz, $CDCl_3$): δ_C 216.2, 171.6, 170.9, 93.5, 81.0, 65.8, 52.7, 52.6, 35.2, 34.5, 33.7, 30.8, 28.5, 25.0, 24.5, 7.6 ppm

HRMS (ESI^+): m/z calc'd for $C_{16}H_{24}O_6Na$ $[M+Na]^+$: 335.1465, found 335.1462.

Dimethyl 1-(hydroxymethyl)-7-phenyl-10-oxa-8-azabicyclo[5.2.1]dec-8-ene-6,6-dicarboxylate (4k) & Dimethyl 2-(4-(5-(hydroxymethyl)-2-phenyl-4,5-dihydrooxazol-5-yl)butyl)malonate (4k')

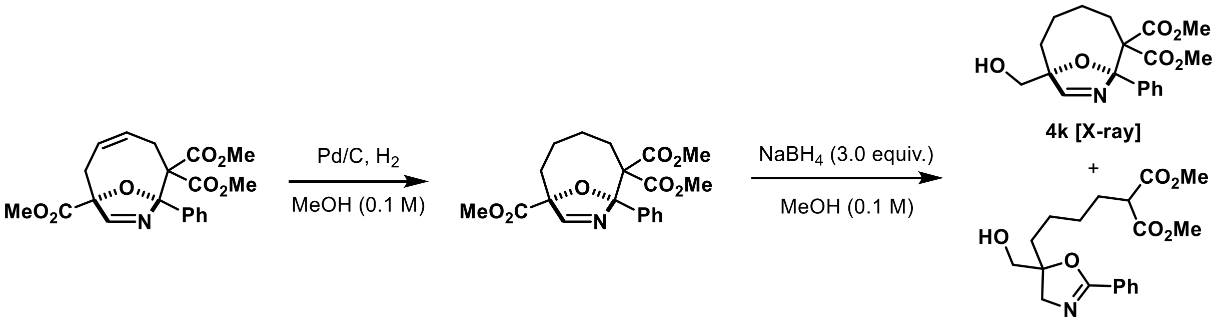

Trimethyl-(*Z*)-7-phenyl-10-oxa-8-azabicyclo[5.2.1]deca-3,8-diene-1,6,6-tricarboxylate **3r** (38.7 mg, 0.1 mmol, 1.0 eq.) and Pd/C (10% Pd, 10.0 mg, 0.01 mmol, 10 mol%) were dissolved in MeOH (0.1 M) under argon. A hydrogen-filled balloon was attached and the reaction vessel purged with H_2 for 10 min. The reaction mixture was stirred overnight at rt under H_2-atmosphere. Then, the reaction mixture was diluted with CH_2Cl_2, filtered, concentrated and purified by column chromatography to yield trimethyl 7-phenyl-10-oxa-8-azabicyclo[5.2.1]dec-8-ene-1,6,6-tricarboxylatethe in quantitative yield. In the next step the obtained hydrogenated product, was dissolved in MeOH (0.1 M) and cooled to 0 °C. Then $NaBH_4$ (3.0 eq.) was added portion-wise. After the addition the ice bath was removed and stirred overnight at room temperature. After completion, the reaction was

quenched with water and extracted with EtOAc. The solvent was removed under reduced pressure and the mixture was purified by column chromatography to yield pure **4k** (17.7 mg, 0.05 mmol, 49%). A minor byproduct (**4k**') was isolated in 21% yield. Based on the NMR characterization data and HRMS, the byproduct was assigned to the following structure **4k'**.

Characterization data for **4k**:

TLC: R_f **=** 0.5 (50:50 *n*-pentane/EtOAc).

NMR Spectroscopy (***see spectra***):

^{1}**H NMR** (400 MHz, $CDCl_3$): δ_H 7.72 – 7.65 (m, 2H), 7.60 (s, 1H), 7.34 – 7.28 (m, 2H), 7.26 – 7.22 (m, 1H), 3.69 – 3.62 (m, 4H), 3.56 – 3.50 (m, 1H), 3.49 (s, 3H), 2.49 – 2.38 (m, 1H), 2.16 – 2.07 (m, 1H), 1.98 – 1.80 (m, 4H), 1.47 – 1.28 (m, 3H);

^{13}**C NMR** (101 MHz, $CDCl_3$): δ_C 169.8, 169.0, 165.1, 142.4, 128.1, 127.6, 127.2, 112.6, 97.5, 67.6, 66.7, 52.2, 52.0, 33.4, 33.2, 26.8, 23.9 ppm.

HRMS (ESI$^+$): m/z calc'd for $C_{19}H_{23}NO_6Na$ $[M+Na]^+$: 384.1418, found 384.1417.

Characterization data for **4k'**:

TLC: R_f **=** 0.20 (50:50 *n*-pentane/EtOAc).

NMR Spectroscopy (***see spectra***):

^{1}**H NMR** (599 MHz, $CDCl_3$): δ_H 7.94 – 7.90 (m, 2H), 7.49 – 7.46 (m, 1H), 7.43 – 7.39 (m, 2H), 3.93 (d, J = 14.8 Hz, 1H), 3.78 – 3.73 (m, 2H), 3.72 (s, 3H), 3.71 (s, 3H), 3.62 (d, J = 12.0 Hz, 1H), 3.33 (t, J = 7.5 Hz, 1H), 1.93 – 1.87 (m, 2H), 1.85 – 1.78 (m, 1H), 1.70 – 1.64 (m, 1H), 1.47 – 1.32 (m, 4H);

^{13}**C NMR** (151 MHz, $CDCl_3$): δ_C 169.9, 163.4, 131.5, 128.5, 128.3, 127.9, 88.3, 66.7, 60.6, 52.6, 52.6, 51.7, 35.2, 28.8, 27.7, 22.7 ppm.

HRMS (ESI$^+$): m/z calc'd for $C_{19}H_{25}NO_6Na$ $[M+Na]^+$: 386.15741, found 386.15740.

Methyl (*Z*)-3-ethyl-3-hydroxy-1-oxo-7,10-dihydro-1*H*,3*H*-3a,6-epoxycyclonona[*c*]furan-10a(6*H*)-carboxylate (4c)

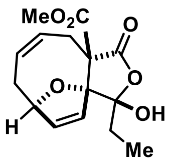

4c

Prepared following a modified literature procedure.[33] BCl_3 (1.0 M in heptane, 0.7 mL, 0.7 mmol, 7.0 eq.) was dissolved in CH_2Cl_2 (7 mL) and cooled to 0 °C. To this solution was added dimethyl (*Z*)-1-propionyl-10-oxabicyclo[5.2.1]deca-4,8-diene-2,2-dicarboxylate **3b** (30.8 mg, 0.1 mmol, 1.0 eq.) dissolved in CH_2Cl_2 (7 mL) and the reaction mixture was stirred for 20 min at 0 °C. The reaction was then quenched with H_2O (14 mL) and stirred for 2 h. The aqueous layer was extracted with CH_2Cl_2 (3 x 10 mL) and the combined organic layers dried

over Na_2SO_4, filtered and concentrated. Purification *via* column chromatography on silica gel (*n*-pentane/EtOAc 100:0 – 88:12) yielded the title compound **4c** (22.6 mg, 0.08 mmol, 77%) as a slightly yellow solid.

TLC: R_f **=** 0.2 (88:12 *n*-pentane/EtOAc).

NMR Spectroscopy (***see spectra***):

^{1}**H NMR** (400 MHz, $CDCl_3$): δ_H 6.44 (s, 1H), 6.05 (d, *J* = 6.1 Hz, 1H), 5.86 – 5.71 (m, 2H), 5.50 – 5.36 (m, 1H), 5.32 (s, 1H), 3.88 (s, 3H), 2.86 – 2.78 (m, 1H), 2.75 – 2.65 (m, 2H), 2.30 – 2.23 (m, 1H), 2.03 – 1.89 (m, 1H), 1.81 – 1.68 (m, 1H), 1.02 (t, *J* = 7.5 Hz, 3H) ppm;

^{13}**C NMR** (101 MHz, $CDCl_3$): δ_C 171.5, 171.4, 133.1, 129.7, 127.7, 126.8, 110.7, 97.3, 88.0, 65.5, 54.3, 33.1, 28.4, 26.9, 7.1 ppm.

HRMS (ESI$^+$): m/z calc'd for $C_{15}H_{18}O_6Na$ [M+Na]$^+$: 317.1001, found 317.0994.

Dimethyl-8-propionyl-4,11-dioxatricyclo[6.2.1.03,5]undec-9-ene-7,7-dicarboxylate (4d)

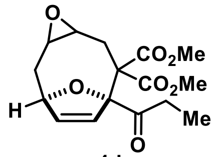

4d

Synthesized following a modified literature procedure.[34] Dimethyl (*Z*)-1-propionyl-10-oxabicyclo[5.2.1]deca-4,8-diene-2,2-dicarboxylate **3b** (30.8 mg, 0.1 mmol, 1.0 eq.) was dissolved in CH_2Cl_2 (0.2 M). $NaHCO_3$ (16.8 mg, 0.2 mmol, 2.0 eq.) and *m*-CPBA (75% purity, 34.5 mg, 0.15 mmol, 1.5 eq.) were added and the reaction mixture stirred at rt overnight. The reaction mixture was filtered, washed with CH_2Cl_2 and the filtrate concentrated. After purification *via* column chromatography on silica gel (*n*-pentane/EtOAc 80:20 – 75:25), the title compound **4d** (27.8 mg, 0.09 mmol, 86%, 60:40 d.r..) was obtained as a white solid.

TLC: R_f **=** 0.4 (75:25 *n*-pentane/EtOAc).

NMR Spectroscopy (***see spectra***):

^{1}**H NMR** (599 MHz, $CDCl_3$): δ_H 6.15 (dd, *J* = 5.9, 1.7, 0.6H, d_1), 6.07 (dd, *J* = 6.1, 1.6 Hz, 0.4H, d_2), 5.88 (dd, *J* = 6.1, 1.6 Hz, 0.4H, d_2), 5.72 (dd, *J* = 5.9, 1.6 Hz, 0.6H, d_1), 5.39 – 5.35 (m, 0.4H, d_2), 5.29 – 5.22 (, 0.6H, d_1), 3.83 (d, *J* = 0.5 Hz, 1.2H), 3.80 (dd, *J* = 5.0, 0.5 Hz, 3.6H, d_1), 3.71 (d, *J* = 0.5 Hz, 1.2H), 3.20 – 3.16 (m, 0.4H, d_2), 3.16 – 3.11 (m, 0.6H, d_1)., 3.07 – 3.02 (m, 0.6H, d_1), 3.00 – 2.91 (m, 0.6H, d_1), 2.88 – 2.76 (m, 0.8H, d_2), 2.74 – 2.68 (m, 0.4H, d_2), 2.68 – 2.59 (m, 1.2H), 2.51 – 2.33 (m, 1.6H), 1.88 – 1.78 (m, 0.8H, d_2), 1.63 – 1.55 (m, 0.6H, d_1), 1.45 – 1.36 (m, 0.6H, d_1), 1.04 – 0.99 (m, 3H) ppm;

^{13}**C NMR** (151 MHz, $CDCl_3$): δ_C 211.7 and 210.7, 171.0 and 169.9, 169.8 and 169.6, 136.0 and 131.6, 130.2 and 126.3, 100.5 and 99.1, 85.2 and 82.1, 64.3 and 63.3, 55.3 and 55.2, 53.3 and 53.2, 53.1 and 53.0, 52.8 and 51.8, 34.7 and 34.0, 33.6 and 32.3, 29.9 and 29.2, 7.5 and 7.3 ppm.

HRMS (ESI$^+$): m/z calc'd for $C_{16}H_{20}O_7Na$ [M+Na]$^+$: 347.1101, found 347.1100.

Dimethyl 6a-acetyl-3-phenyl-4-vinyl-3a,4,5,6a-tetrahydro-6*H*-cyclopenta[*b*]thiophene-6,6-dicarboxylate (4e)

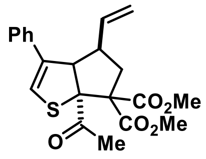

4e

Synthesized following General Procedure F using: 1-(4-phenylthiophen-2-yl)ethan-1-one **1ar** (40.4 mg, 0.2 mmol, 1.0 eq.), dimethyl 2-vinylcyclopropane-1,1-dicarboxylate **2a** (92.0 mg, 0.5 mmol, 2.5 eq.), Ir-F (4.0 mg, 0.004 mmol, 2 mol%) and MeCN (2 mL). Purification *via* column chromatography on silica gel (*n*-pentane/EtOAc 100:0 – 90:10) afforded the title compound **4e** (38.1 mg, 0.1 mmol, 49%) as a white solid.

TLC: R_f **=** 0.3 (92:8 *n*-pentane/EtOAc).

NMR Spectroscopy (***see spectra***):

^{1}**H NMR** (400 MHz, $CDCl_3$): δ_H 7.33 – 7.24 (m, 4H), 7.22 – 7.16 (m, 1H), 6.47 (s, 1H), 5.56 (ddd, *J* = 17.0, 10.0, 8.9 Hz, 1H), 4.89 (d, *J* = 17.0 Hz, 1H), 4.69 (dd, *J* = 10.1, 1.6 Hz, 1H), 4.48 (d, *J* = 10.6 Hz, 1H), 3.78 (s, 3H), 3.78 (s, 3H), 3.46 – 3.37 (m, 1H), 2.69 – 2.61 (m, 1H), 2.56 – 2.49 (m, 1H), 2.43 (s, 3H) ppm;

^{13}**C NMR** (101 MHz, $CDCl_3$): δ_C 200.9, 171.1, 169.9, 137.9, 137.8, 135.0, 128.4, 127.1, 125.9, 119.8, 116.0, 81.1, 65.9, 57.9, 53.1, 52.8, 45.4, 42.3, 25.1 ppm.

HRMS (ESI^+): m/z calc'd for $C_{21}H_{22}O_5SNa$ $[M+Na]^+$: 409.1080, found 409.1080.

(*Z*)-1-(5-Benzyl-10-oxabicyclo[5.2.1]deca-4,8-dien-1-yl)ethan-1-one (3as) & (*Z*)-1-(3-Benzyl-10-oxabicyclo[5.2.1]deca-3,8-dien-1-yl)ethan-1-one (3as')

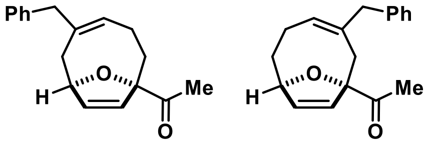

3as **3as'**

Synthesized following a modified General Procedure F: 1-(Furan-2-yl)ethan-1-one **1as** (330 mg, 3.0 mmol, 1.0 eq.), (2-cyclopropylallyl)benzene **2g** (948 mg, 6.0 mmol, 2.0 eq.), Ir-F (40.0 mg, 0.04 mmol, 1.3 mol%) and MeCN (30 mL) were employed to obtain the major regioisomer **3as** (223 mg, 0.85 mmol, 28%) after purification *via* column chromatography on silica gel (*n*-pentane/EtOAc 100:0 – 98:2) as a colourless oil, as well as the minor regioisomer **3as'** (70.7 mg, 0.26 mmol, 9%) as a colourless oil.

Note: Unreacted (2-cyclopropylallyl)benzene (456 mg, 2.9 mmol, 97%) could be recovered during the purification step.

Characterization data for **3as**:

TLC: R_f **=** 0.2 (98:2 *n*-pentane/EtOAc).

NMR Spectroscopy (***see spectra***):

^{1}H NMR (400 MHz, $CDCl_3$): δ_H 7.32 – 7.25 (m, 2H), 7.23 – 7.15 (m, 1H), 7.15 – 7.09 (m, 2H), 5.64 (dd, *J* = 5.8, 1.4 Hz, 1H), 5.55 (t, *J* = 8.3 Hz, 1H), 5.34 – 5.25 (m, 2H), 3.17 (d, *J* = 14.7 Hz, 1H), 3.01 (d, *J* = 14.4 Hz, 1H), 2.72 – 2.64 (m, 1H), 2.39 – 2.27 (m, 1H) 2.13 (s, 3H), 2.12 – 2.06 (m, 1H), 1.97 – 1.79 (m, 3H) ppm;

^{13}C NMR (101 MHz, $CDCl_3$): δ_C 211.6, 139.5, 137.0, 130.6, 129.5, 128.6, 128.4, 127.6, 126.3, 95.8, 86.3, 46.9, 38.2, 36.2, 34.7, 24.6, 23.6 ppm.

Characterization data for **3as'**:

TLC: R_f **=** 0.2 (98:2 *n*-pentane/EtOAc).

NMR Spectroscopy (***see spectra***):

^{1}H NMR (400 MHz, $CDCl_3$): δ_H 7.31 – 7.23 (m, 2H), 7.20 – 7.10 (m, 3H), 5.81 (dd, *J* = 5.9, 1.9 Hz, 1H), 5.61 – 5.52 (m, 1H), 5.27 (dd, *J* = 5.9, 2.1 Hz, 1H), 5.17 – 5.10 (m, 1H), 3.18 (d, *J* = 14.7 Hz, 1H), 3.01 (d, *J* = 14.4 Hz, 1H), 2.57 – 2.45 (m, 2H), 2.41 – 2.27 (m, 1H), 2.17 (s, 3H), 2.09 – 1.97 (m, 1H), 1.94 – 1.77 (m, 1H), 1.65 – 1.56 (m, 1H) ppm;

^{13}C NMR (101 MHz, $CDCl_3$): δ_C 210.8, 139.4, 137.4, 132.8, 129.6, 128.8, 128.4, 126.9, 126.2, 99.3, 84.2, 46.8, 39.4, 32.8, 25.9, 23.0 ppm.

HRMS (ESI$^+$): m/z calc'd for $C_{18}H_{20}O_2Na$ $[M+Na]^+$: 291.1355, found 291.1355.

Cis-1-(5-Benzyl-10-oxabicyclo[5.2.1]decan-1-yl)ethan-1-one (4j) & trans-1-(5-Benzyl-10-oxabicyclo[5.2.1]decan-1-yl)ethan-1-one (4j')

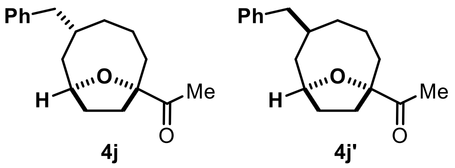

(*Z*)-1-(5-benzyl-10-oxabicyclo[5.2.1]deca-4,8-dien-1-yl)ethan-1-one **3as** (216 mg, 0.8 mmol, 1.0 eq.) and Pd/C (10% Pd, 80.0 mg, 0.01 mmol, 10 mol%) were dissolved in MeOH (0.1 M) under argon. A hydrogen-filled balloon was attached and the reaction vessel purged with H_2 for 10 min. The reaction mixture was stirred overnight at rt under H_2-atmosphere. Then, the reaction mixture was diluted with CH_2Cl_2, filtered and concentrated. Purification *via* column chromatography on silica gel (*n*-pentane/EtOAc 100:0 – 98:2) afforded the major diastereomer **4j** (156 mg, 0.57 mmol, 72%) as a colourless oil, as well as the minor diastereomer **4j'** (60.5 mg, 0.22 mmol, 28%) as a colourless oil. The structure of 4j was assigned by 1D-NOESY NMR.

Characterization data for **4j**:

TLC: R_f **=** 0.2 (98:2 *n*-pentane/EtOAc).

NMR Spectroscopy (***see spectra***):

^{1}H NMR (400 MHz, $CDCl_3$): δ_H 7.31 – 7.25 (m, 2H), 7.22 – 7.12 (m, 3H), 4.63 – 4.56 (m, 1H), 2.62 – 2.46

(m, 2H), 2.21 (s, 3H), 2.15 – 1.60 (m, 10H), 1.56 – 1.44m, 3H) ppm;

^{13}C NMR (101 MHz, $CDCl_3$): δ_C 214.2, 141.3, 129.4, 128.4, 126.0, 91.5, 80.0, 45.5, 40.9, 38.0, 36.1, 33.8, 32.4, 29.5, 25.2, 24.3 ppm.

Characterization data for **4j'**:

TLC: R_f **=** 0.2 (98:2 *n*-pentane/EtOAc).

NMR Spectroscopy (***see spectra***):

^{1}H NMR (400 MHz, $CDCl_3$): δ_H 7.32 – 7.26 (m, 2H), 7.22 – 7.14 (m, 3H), 4.26 – 4.14 (m, 1H), 2.60 (dd, *J* = 13.6, 6.7 Hz, 1H), 2.47 – 2.38 (m, 2H), 2.19 (s, 3H), 2.15 – 2.06 (m, 1H), 2.02 – 1.81 (m, 4H), 1.74 – 1.57 (m, 3H), 1.53 – 1.41 (m, 2H), 1.37 – 1.25 (m, 1H), 1.11 – 0.99 (m, 1H) ppm;

^{13}C NMR (101 MHz, $CDCl_3$): δ_C 212.7, 141.4, 129.4, 128.3, 125.9, 89.1, 78.0, 45.0, 41.3, 38.3, 37.7, 36.7, 34.9, 31.9, 25.9, 22.3 ppm.

HRMS (ESI^+): m/z calc'd for $C_{18}H_{24}O_2Na$ $[M+Na]^+$: 295.1669, found 295.1669.

***Cis*-5-Benzyl-10-oxabicyclo[5.2.1]decane-1-carboxylic acid (4f)**

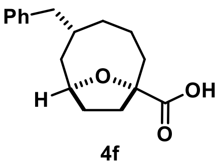

4f

The title compound was synthesized according to a literature procedure.[35] **4j** (0.6 mmol, 156 mg, 1.0 eq.) was dissolved in CCl_4 (1.4 mL) and CH_2Cl_2 (2.0 mL). Benzyltriethylammonium chloride (36.0 mg, 0.16 mmol, 0.25 eq.) was added, and a 50% solution of NaOH (0.9 mL) was added dropwise. The reaction mixture was stirred overnight at rt. Water was added and the solution acidified with 2N HCl, until pH = 2 was reached. The aqueous layer was then extracted with EtOAc (3 x), the combined organic phases dried over $MgSO_4$, filtered and concentrated to yield **4f** (154 mg, 0.6 mmol, 99%) as a brown oil.

TLC: R_f **=** 0.3 (30:70 *n*-pentane/EtOAc).

NMR Spectroscopy (***see spectra***):

^{1}H NMR (400 MHz, $CDCl_3$): δ_H 7.27 (d, *J* = 10.1 Hz, 2H), 7.23 – 7.10 (m, 3H), 4.72 – 4.62 (m, 1H), 2.64 – 2.44 (m, 2H), 2.39 – 2.29 (m, 1H), 2.28 – 2.16 (m, 1H), 2.13 – 2.02 (m, 2H), 1.98 – 1.76 (m, 5H), 1.73 – 1.63 (m, 1H), 1.61 – 1.40 (m, 3H) ppm.

^{13}C NMR (101 MHz, $CDCl_3$): δ_C 176.9, 141.0, 129.3, 128.5, 126.1, 87.1, 80.7, 45.4, 40.4, 37.8, 36.6, 33.5, 33.1, 29.6, 24.0 ppm.

HRMS (ESI^+): m/z calc'd for $C_{17}H_{22}O_3Na$ $[M+Na]^+$: 297.1461, found 297.1461.

***Cis*-(5-Benzyl-10-oxabicyclo[5.2.1]decan-1-yl)(4-(8-chloro-5,6-dihydro-11*H*-benzo[5,6]cyclohepta[1,2-*b*]pyridin-11-ylidene)piperidin-1-yl)methanone (4g)**

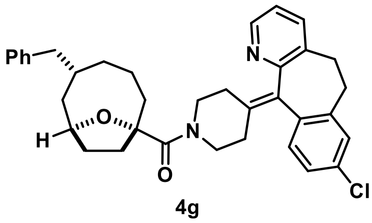

4g

To a solution of **4f** (27.4 mg, 0.1 mmol, 1.0 eq.) and desloratadine (37.3 mg, 0.12 mmol, 1.2 eq.) in CH_2Cl_2 (0.5 M), were added HATU (45.6 mg, 0.12 mmol, 1.2 eq.) and DIPEA (42 µL, 0.24 mmol, 2.4 eq.). The reaction mixture was stirred for 24 h at rt, and after completion of the reaction concentrated. Purification *via* column chromatography on silica gel (CH_2Cl_2/MeOH 100:0 – 98:2) afforded **4g** (45.3 mg, 0.08 mmol, 80%) as a slightly yellow oil.

TLC: R_f **=** 0.3 (97:3 CH_2Cl_2/MeOH).

NMR Spectroscopy (***see spectra***):

^{1}H NMR (599 MHz, $CDCl_3$): δ_H 8.42 (s, 1H), 7.46 – 7.42 (m, 1H), 7.29 – 7.23 (m, 2H), 7.21 – 7.07 (m, 7H), 4.58 – 4.41 (m, 1H), 4.36 – 3.95 (m, 2H), 3.62 (d, J = 34.8 Hz, 1H), 3.47 – 3.32 (m, 2H), 3.21 – 2.93 (m, 1H), 2.89 – 2.77 (m, 2H), 2.70 – 2.62 (m, 1H), 2.60 – 2.43 (m, 3H), 2.42 – 2.29 (m, 3H), 2.18 – 2.11 (m, 1H), 2.00 – 1.71 (m, 7H), 1.66 – 1.36 (m, 4H), 1.27 – 1.24 (m, 1H) ppm;

^{13}C NMR (151 MHz, $CDCl_3$): δ_C 174.1, 146.9, 141.3, 139.7, 138.1, 137.6, 134.0, 133.5, 133.0, 130.8, 129.3, 129.1, 128.3, 126.3, 125.9, 122.4, 89.7, 89.6, 80.1, 47.3, 45.5, 44.8, 40.7, 38.8, 38.8, 38.0, 34.7, 33.6, 31.9, 31.7, 29.8, 28.3, 24.4 ppm.

HRMS (ESI^+): m/z calc'd for $C_{36}H_{39}N_2O_2ClNa$ $[M+Na]^+$: 589.2592, found 589.2592.

6-(Fluoro(phenyl)methyl)cyclononane-1,4-dione (4h)

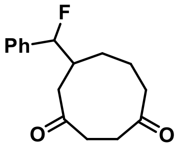

4h

Synthesized using a literature procedure.[36] **4f** (27.4 mg, 0.1 mmol, 1.0 eq.), Selectfluor (106.3 mg, 0.3 mmol, 3.0 eq.), Na_2HPO_4 (28.4 mg, 0.2 mmol, 2.0 eq.) and Ir-F (1.0 mg, 0.001 mmol, 1 mol%) were dissolved in $MeCN/H_2O$ (1:1 v/v, 1 mL) and the solution sparged with argon for 10 min. The reaction was irradiated with blue LEDs (30 W, λ_{max} = 450 nm) for 24 hours while stirring. Water was added and the aqueous layer extracted with Et_2O (3 x). The combined organic layers were dried over Na_2SO_4, filtered and concentrated. From crude ^{1}H NMR analysis, a crude yield of 31% (50:50 d.r.) was determined. Purification *via* column chromatography on silica gel (*n*-pentane/EtOAc 100:0 – 90:10) afforded **4h** (8.1 mg, 0.03 mmol, 31%, 50:50 d.r.) as colourless oil. One of the diastereomer was separated and the structure was confirmed by extensive 2D-NMR analysis (see NMRs).

TLC: R_f **=** 0.3 (90:10 *n*-pentane/EtOAc).

NMR Spectroscopy (***see spectra***):

^{1}**H NMR** (400 MHz, $CDCl_3$): δ_H 7.44 – 7.34 (m, 3H), 7.32 – 7.27 (m, 2H), 5.30 – 5.09 (m, 1H), 2.91 – 2.72 (m, 2H), 2.71 – 2.64 (m, 1H), 2.59 – 2.48 (m, 2H), 2.48 – 2.26 (m, 4H), 1.92 – 1.79 (m, 1H), 1.73 – 1.45 (m, 3H) ppm;

^{13}**C NMR** (101 MHz, $CDCl_3$): δ_C 215.1 and 214.8, 213.5 and 212.7, 138.8 and 138.0, 128.9 and 128.8, 128.8, 126.2 (d, *J* = 7.6 Hz) (d_1) and 126.2 (d, *J* = 7.2 Hz) (d_2), 97.7 (d, *J* = 177.5 Hz) (d_1) and 97.3 (d, *J* = 177.1 Hz) (d_2), 47.0 (d, *J* = 3.9 Hz) (d_2) and 46.2 (d, *J* = 3.9 Hz) (d_1), 45.0 and 44.8, 39.7 and 39.4, 39.4 and 39.2, 39.3 and 39.1, 31.8 (d, *J* = 3.5 Hz) (d_1) and 29.7 (d, *J* = 3.5 Hz) (d_2), 21.4 and 21.1 ppm;

^{19}**F{^{1}H} NMR** (376 MHz, $CDCl_3$): δ_F -180.9, -181.0.

HRMS (ESI$^+$): m/z calc'd for $C_{16}H_{19}FO_2Na$ $[M+Na]^+$: 285.1261, found 285.1261.

Cis-3-(5-Benzyl-10-oxabicyclo[5.2.1]decan-1-yl)cyclopentan-1-one (4i)

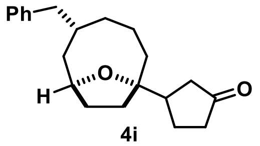

4i

Synthesized using a literature procedure.[37] A Schlenk tube was equipped with **Ir-F** (1.0 mg, 0.001 mmol, 1 mol%), **4f** (27.4 mg, 0.1 mmol, 1.0 eq.), K_2HPO_4 (20.9 mg, 0.12 mmol, 1.2 eq.) and cyclopent-2-en-1-one (10 µL, 0.12 mmol, 1.2 eq.) and DMF (0.5 mL). The reaction mixture was sparged with argon for 15 min and then irradiated with blue LEDs (30 W, λ_{max} = 450 nm) for 24 hours while stirring. The crude reaction was diluted with sat. aq. $NaHCO_3$-solution and the aqueous layer extracted with Et_2O (3 x). The combined organic layers were washed with H_2O and brine and dried over Na_2SO_4, filtered and concentrated. Purification *via* column chromatography on silica gel (*n*-pentane/EtOAc 100:0 – 95:5) afforded **4i** (20.1 mg, 0.06 mmol, 64%, 50:50 d.r.) as colourless oil.

TLC: R_f **=** 0.3 (95:5 *n*-pentane/EtOAc).

NMR Spectroscopy (***see spectra***):

^{1}**H NMR** (400 MHz, $CDCl_3$): δ_H 7.32 – 7.25 (m, 2H), 7.21 – 7.12 (m, 3H), 4.48 – 4.39 (m, 1H), 2.61 – 2.43 (m, 2H), 2.38 – 1.94 (m, 8H), 1.90 – 1.41 (m, 12H) ppm;

^{13}**C NMR** (101 MHz, $CDCl_3$): δ_C 219.7 and 219.6, 141.5 and 141.5, 129.3, 128.3, 125.9 and 125.9, 86.2 and 86.1, 79.5 and 79.2, 48.4 and 48.3, 45.6, 41.7 and 41.6, 40.9 and 40.8, 39.4 and 39.0, 39.0 and 38.9, 37.9 and 37.9, 34.2 and 34.1, 32.4 and 31.4, 30.2 and 29.8, 24.9, 24.4 and 24.3 ppm.

HRMS (ESI$^+$): m/z calc'd for $C_{21}H_{28}O_2Na$ $[M+Na]^+$: 335.1982, found 335.1981.

2.5. Reaction Optimisation

Reactions were performed using **1a** (0.05 mmol) according to a modified **General Procedure F**. Modifications to standard conditions and key observations from each study are stated.

2.5.1. Establishing the stoichiometry and the solvent

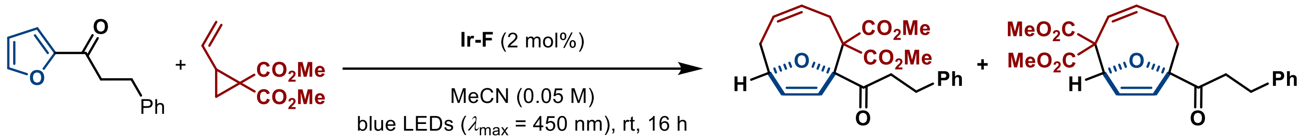

Table S1: Establishing the stoichiometry

Entry	1a (XX eq.) 2a (XX eq.)	% Yield (3a)[a]	% Yield (3a')[a]
1	1a (1.0 eq.) 2a (1.0 eq.)	25	4
2	1a (1.5 eq.) 2a (1.0 eq.)	28	6
3	1a (1.0 eq.) 2a (2.0 eq.)	41	9
4	1a (1.0 eq.) 2a (3.0 eq.)	**52**	**11**
5	1a (1.0 eq.) 2a (5.0 eq.)	54	12

[a] Yields were determined by ^{1}H NMR analysis using dibromomethane as an internal standard.

Key observations: Upon screening different stoichiometries, excess of the VCP **2a** increased the overall yield, while using the furan **1a** in excess did not show any significant improvement of the reaction outcome. This presumably can be explained by the instability of the furan under irradiation, which leads to slow furan decomposition as a competing side reactivity. Thus, higher equivalents of VCP leads to a preference of the productive cycloaddition pathway over decomposition. Further increase of the VCP equivalents to 5.0 did not increase the yield any further.

Table S2: Establishing the solvent

Entry	solvent (0.05 M)	% Yield (3a)[a]	% Yield (3a')[a]
1	MeCN	**63**	**12**
2	dioxane	42	9
3	THF	61	14
4	TFT	60	12
5	DMC	54	13
6	chlorobenzene	52	9

[a] Yields were determined by ^{1}H NMR analysis using dibromomethane as an internal standard.

Key observations: A number of solvents performed well under our reaction conditions, and the choice of

solvent did not influence the regioselectivity significantly (between 4:1 and 5:1). Switching from CH_2Cl_2 to MeCN could increase the yield to 75%.

2.5.2. Establishing the photocatalyst

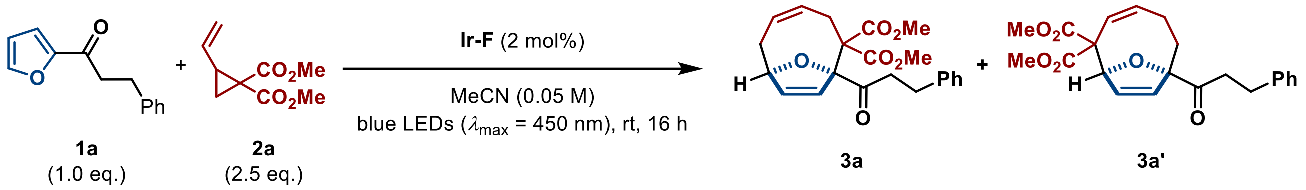

Table S3: Establishing the photocatalyst

Entry	PC (mol%)	E_T (kcal/mol)[38]	% Yield (3a)[a]	% Yield (3a')[a]
1	365 nm	-	42	9
2	TXT (10 mol%), 405 nm	65.5	56	11
3	$Ir(dFppy)_3$ (2 mol%), 450 nm	63.5	56	12
4	[Ir-F] (2 mol%), 450 nm	61.8	**65**	**14**
5	$Ir(ppy)_3$ (2 mol%), 450 nm	58.1	n.d.	n.d.
6	$Ru(bpy)_3Cl_2$	46.5	n.d.	n.d.
7	without PC	-	n.d.	n.d.
8	No light	-	n.d.	n.d.

[a] Yields were determined by ^{1}H NMR analysis using dibromomethane as an internal standard.

Key observations: The triplet energy of furan **1a** was calculated to be 59.4 kcal/mol, thus the triplet energy of $Ir(ppy)_3$ as well as $Ru(bpy)_3Cl_2$ are too low to excite **1a**. All other photocatalysts were able to generate **3a** in moderate to good yields.

2.5.3. Exploring substitution-triplet energy-reactivity correlation

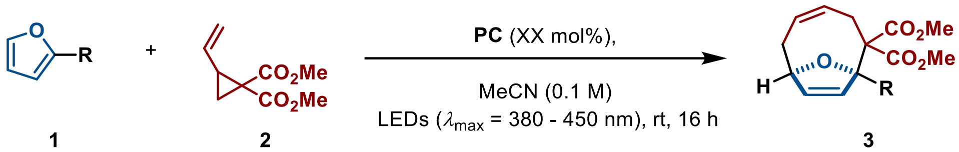

1 (1.0 eq.) **2** (2.5 eq.) **3**

Table S4: C2-substitution.

Entry	R	E_T (kcal/mol)[39]	PC (E_T)	wavelenght (nm)	% Yield (3)[a]
1	−2-CO$_2$Me , 5-Phenyl (**1l**)	54.4	Ir-F (**61.8**)	450	67 (>95:5 r.r.)
2	−C(O)CF$_3$ (**1k**)	56.4	Ir-F (**61.8**)	450	32 (>95:5 r.r.)
3	− (4-pyridinyl) (**1au**)	58.5	Ir-F (**61.8**)	450	n.d.
4	−C(O)H (**1at**)	58.4	Ir-F (**61.8**)	450	40 (>95:5 r.r.)
5	−2-Br, 5-CO$_2$Me (**1av**)	59.0	Ir-F (**61.8**)	450	traces
6	−C(O)Ph (**1f**)	59.7	Ir-F (**61.8**)	450	49 (90:10 r.r.)
7	−C(O)Et (**1b**)	60.6	Ir-F (**61.8**)	450	67 (84:16 r.r.)
8	−C(O)pyrazole (**1e**)	61.2	Ir-F (**61.8**)	450	31 (90:10 r.r.)
9	−CN (**1i**)	63.9	3-OMe-TXT (**67.6**)	380	52 (63:37 r.r.)
10	−CO$_2$Me (**1j**) *	67.2	3-OMe-TXT (**67.6**)	380	48 (36:64 r.r.)
11	−C(O)NMe$_2$ (**1aw**)	67.9	3-OMe-TXT (**67.6**)	380	15 (>95:5 r.r.)
12	−OMe (**1ax**)	68.6	3-OMe-TXT (**67.6**)	380	n.d.
13	−Bpin (**1ay**)	70.2	3-OMe-TXT (**67.6**)	380	n.d.
14	−Me (**1az**)	72.8	3-OMe-TXT (**67.6**)	380	n.d.

2 mol% of Ir-F was used. 10 mol% of 3-OMe-TXT was used. For furan substrates predicted triplet energies from EnTdecker model are given. [*] Reaction conducted with Ir-F at 450 nm yielded no product. [a] Yields were determined by ^{1}H NMR analysis using dibromomethane as an internal standard.

Key observations: For excitation with standard photocatalyst **Ir-F** acyl-, benzoyl-, trifluoroacyl-, aldehyde-, phenyl- and -bromo substitution (entry 1-8) effectively lower the triplet energy enough, probably due to extension of the π-system and for the special case of bromo-substitution because of the heavy atom effect. However, 2-aryl substitution alone does not activate the furan sufficiently for reaction with the electron-deficient VCP and no conversion is observed (entry 3). Adding an additional ester substituent in the 5-position enabled us to employ the 2-aryl substituted furans successfully (entry 1). For the bromo-substituted furan, decomposition was observed during the course of the reaction, presumably due to the labile nature of the C−Br bond.

Nitrile, ester and amide substituents lower the triplet energy less effectively, and are not accessible using our standard reaction conditions (entry 10). To our delight, employing 3-OMe-TXT derivative with a higher triplet

energy (E_T = 67.6 kcal/mol)[40] enabled activation of nitrile and ester substituted furans (entry 10 and 11). Alkyl, boronic ester and methoxy substituted furans do not react because of their inherent high triplet energy (entry 12-14), which cannot be reached by commonly used energy transfer photocatalysts.

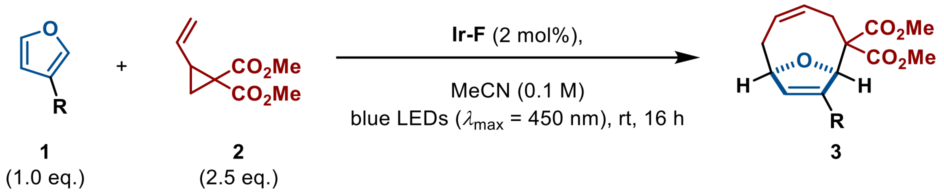

Table S5: C3-substitution.

Entry	R	E_T (kcal/mol)[39]	% Yield (3)[a]
1	–CO_2Me	71.6	n.d.
2	–C(O)Me	68.8	n.d.
3	–Ph	66.6	n.d.

. [a] Yields were determined by ^{1}H NMR analysis using dibromomethane as an internal standard. For furan substrates predicted triplet energies from EnTdecker model are given.

Key observations: C2-substution compared to C3-subsitution has a high impact on the triplet energy. The corresponding 3-acyl, -aryl or -ester furans do not react under the energy transfer conditions due to their inherent inaccessibility *via* energy transfer.

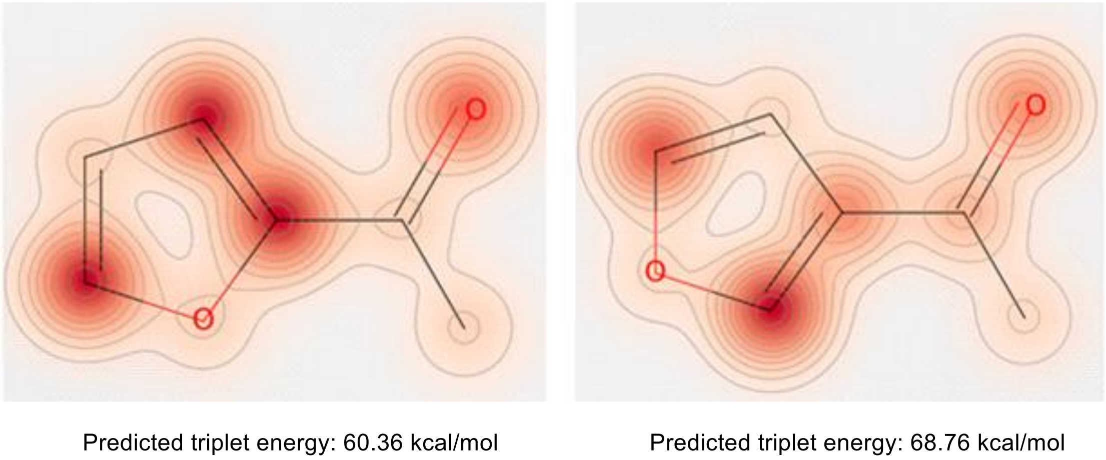

Figure S1: Comparison of EnTdecker[39] predicted triplet energies and spin densities for **C2**(left) / **C3**(right) acyl furan.

2.6. Substrate Limitations

Additional Substrate scope examples

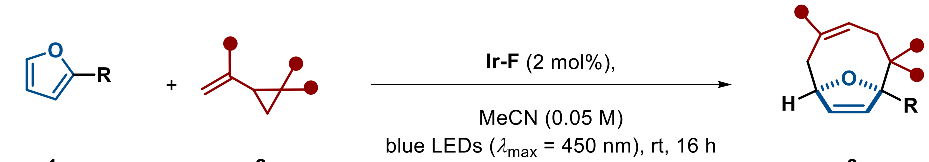

1 (1.0 eq.) **2** (2.5 eq.) **3**

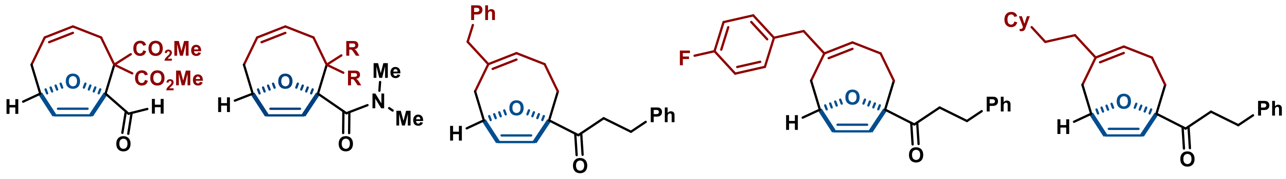

P1, 40% (>95:5 r.r.) **P2**, 15% (>95:5) **P3**, 62% (81:19 r.r.) **P4**, 40% (80:20 r.r.) **P5**, 60% (82:18 r.r.)

Figure S2.1: Additional scope entried. Reaction conditions: **1** (0.2 mmol), VCP (0.5 mmol), Ir-F (2 mol%), MeCN (0.1 M), blue LEDs (λ_{max} = 450 nm), rt, 16 h. Yields given were determined by ^{1}H NMR analysis of the crude reaction using dibromomethane as an internal standard. For **P2** the reaction was conducted using 3-OMe-TXT (10 mol%) with purple LEDs (λ_{max} = 380 nm).

Heteroarene Substrate Limitations

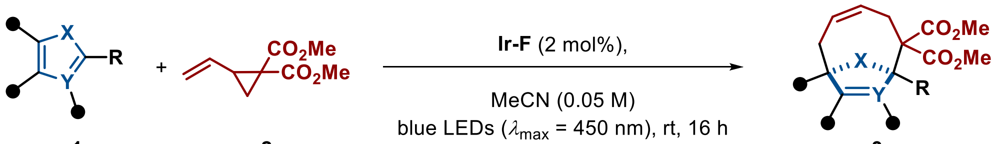

1 (1.0 eq.) **2** (2.5 eq.) **3**

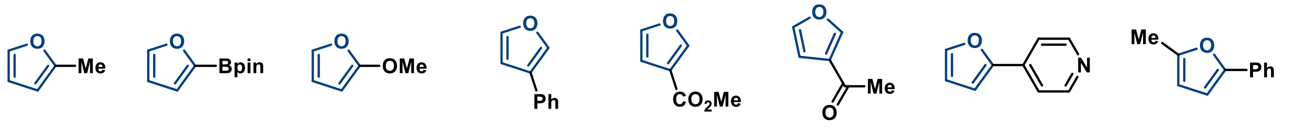

n.d. n.d. n.d. n.d. n.d. n.d. n.d. n.d.

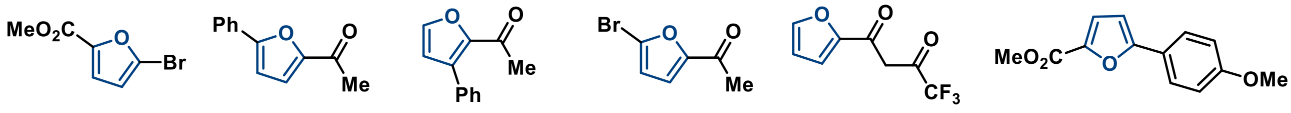

n.d. n.d. 11% 17% n.d. n.d.

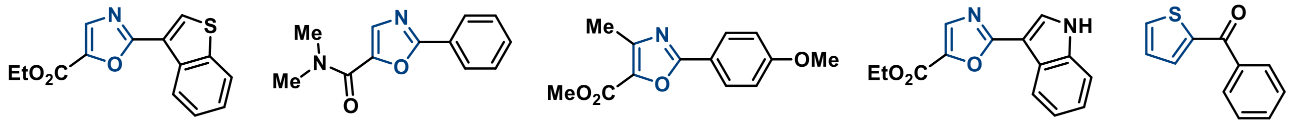

n.d n.d. 10% 10% 13%

Figure S2.2: Unsuccessful heteroarenes. Reaction conditions: **A** (0.1 mmol), VCP (0.25 mmol), Ir-F (2 mol%), MeCN (0.05 M), blue LEDs (λ_{max} = 450 nm), rt, 16 h. Yields given were determined by ^{1}H NMR analysis of the crude reaction using dibromomethane as an internal standard. Reactions with the first three substrates were using 3-OMe-TXT (10 mol%) with purple LEDs (λ_{max} = 380 nm).

Key observations: 2-Methyl, -Bpin or -methoxy substituted furans remained unreacted under the reaction conditions due to their inherent inaccessibility via energy transfer activation (E_T > 68 kcal/mol). 3-Aryl, -ester and -acyl substituted furans also processed high triplet energies (see Table S5), and thus did not participate in our reaction.

The 2-aryl substituted furans either remained unreacted or decomposed in case of additional acyl substitution on the 5-position. Amide- and benzothiophene-substituted oxazoles showed no conversion. The tertiary- and unprotected indole-substituted oxazoles afforded the products in low yields, in the first case presumably due to steric hinderance and in the letter because of side reactivity of the free indole NH. Benzoylthiophene participated in the reaction, but was low-yielding, due to decomposition of the (5+4) cycloadduct by remaining starting material.

The aldehyde cycloadduct **P1** could be clearly identified in the crude ^{1}H NMR, however the compound was unstable on silica (you could observe loss of the aldehyde proton and the ester groups after the column).

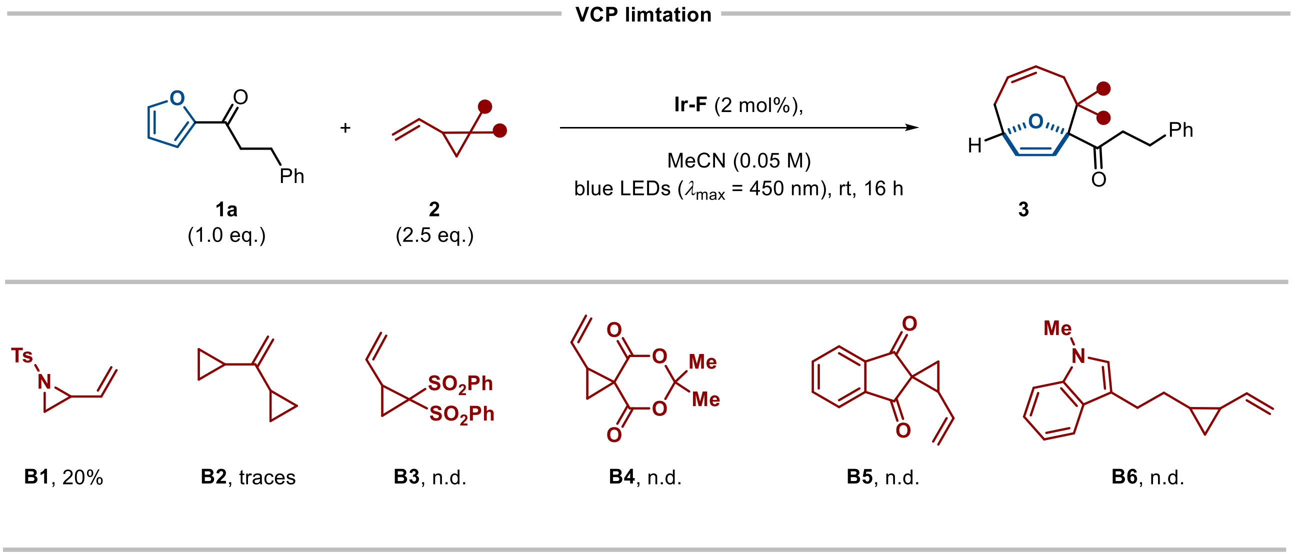

Figure S2.3: Unsuccessful vinyl cyclopropanes. Reaction conditions: **1a** (0.1 mmol), VCP (0.25 mmol), Ir-F (2 mol%), MeCN (0.05 M), blue LEDs (λ_{max} = 450 nm), rt, 16 h. Yields given were determined by ^{1}H NMR analysis of the crude reaction using dibromomethane as an internal standard.

With VCPs **B3** and **B4** no reaction was observed and the starting materials remained. VCPs **B4** and **B5** were deemed to be to sterically hindered for the reaction to occur.

2.7. Sensitivity Screen

The sensitivity assessment was conducted as reported by Glorius and coworkers[41] using conditions modified from **General Procedure E**. During the sensitivity screen a reaction with unmodified conditions was performed and the combined yield obtained (79%) of both regioisomers was taken as the benchmark.

Table S6: Sensitivity assessment of the (5+4) cycloaddition reaction.

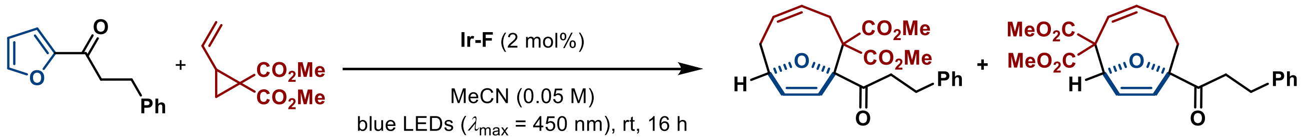

1a (1.0 eq.) **2a** (2.5 eq.) **3a** **3a'**

Entry	Modification	Deviation from standard conditions	Yield (3a & 3a') [a]	Deviation from benchmark
1	high H_2O	H_2O (20 µL)	75%	-4%
2	high O_2	air	44%	-35%
3	low O_2	Freeze Pump Thaw	79%	0%
4	low concentration	2.2 mL MeCN	78%	-1%
5	high concentration	1.8 mL MeCN	79%	0%
6	high intensity	2 cm distance to LED	62%	-17%
7	low intensity	32 cm distance to LED	53%	-26%
8	big scale	standard scale x10	78%	-1%
9	low temperature	cooled photoreactor (-30 °C)	79%	0%
10	high temperature	fan off	72%	-7%

[a] Yields were determined by ^{1}H NMR analysis using dibromomethane as an internal standard.

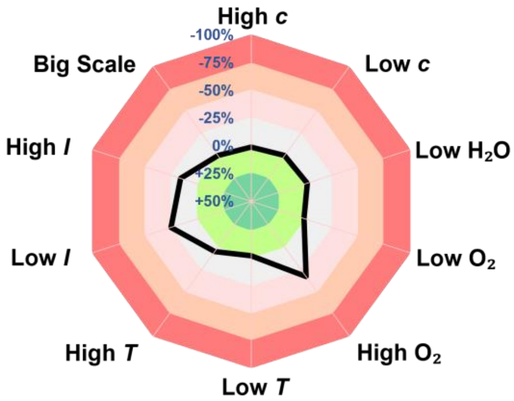

Figure S3: Radar diagram representation of sensitivity screen.

2.8. Additive-based Robustness Screen

The robustness screen was conducted as reported by Glorius and coworkers,[42] using conditions modified from **General Procedure F**.

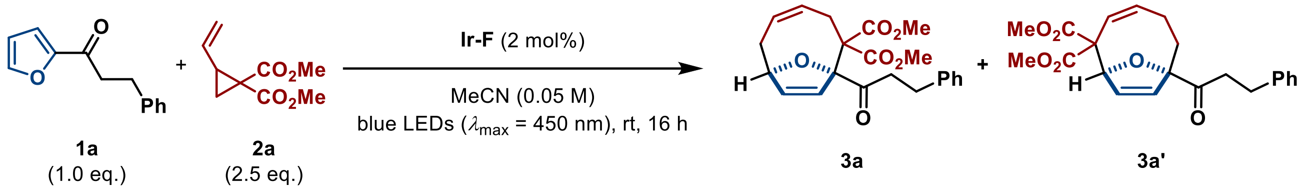

Stock solution preperation:

To an oven-dried Schlenk tube equipped with a Teflon-coated magnetic stir bar was added Ir-F (32.0 mg, 0.032 mmol, 2.00 mol%) and 1-(furan-2-yl)-3-phenylpropan-1-one **1a** (321.0 mg, 1.6 mmol, 1.0 eq.). The Schlenk tube was evacuated and backfilled with argon three times before dimethyl 2-vinylcyclopropane-1,1-dicarboxylate **2a** (736.0 mg, 4.0 mmol, 2.5 eq.) and MeCN (16 mL, 0.1 M) were added.

Reaction preperation:

To an oven-dried Schlenk tube equipped with a Teflon-coated magnetic stir bar was added the freshly prepared stock solution (1.0 mL) and the respective additive (0.1 mmol, 1.0 eq). A control reaction without additive was also prepared simultaneously. The reaction mixtures were stirred under irradiation with blue LEDs (18 W, λ_{max} = 450 nm) for 16 h. After this time, a mesitylene solution (0.1 M in EtOAc, 1.0 mL. 1.0 eq.) was added as an internal standard for analyzing the product yield and the remaining additive by GC-FID. The results are shown in **Table S6**.

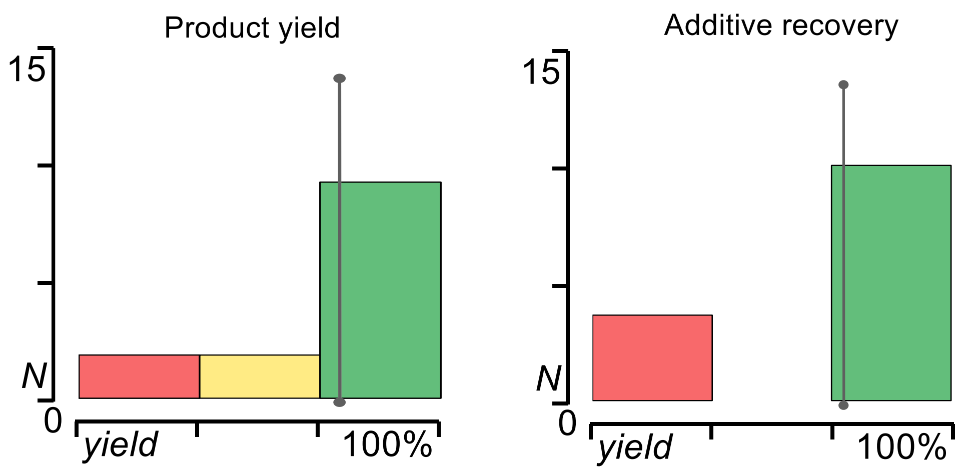

Figure S4: Bar graph representation of robustness screen. Left: Impact of additive normed on product yield. Right: Recovery of additive. Grey Line: average.

Table S7: Summary of additive-based robustness screen results.

Entry	Additive	Product yield (%)[a]	Additive recovery (%)[a]	Entry	Additive	Product yield (%)[a]	Additive recovery (%)[a]
1	none	69	69	9	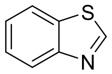	43	100
2	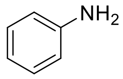	0	96	10	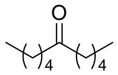	58	0
3	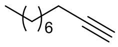	44	96	11	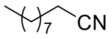	53	100
4	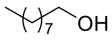	57	94	12	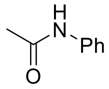	55	100
5	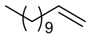	57	0	13	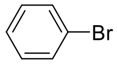	60	100
6	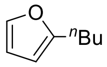	44	0	14	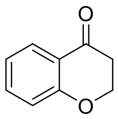	73	92
7	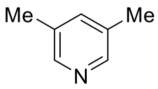	54	100	15	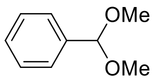	60	93
8	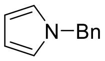	4	0	16	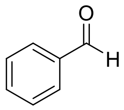	70	85

[a] Yields were determined by GC-FID analysis using mesitylene as an internal standard.

Only aniline and pyrrole shut down the reactivity potentially due to competitive quenching, which was confirmed by performing Stern-Volmer quenching studies with the photocatalyst **Ir-F** (figure S5).

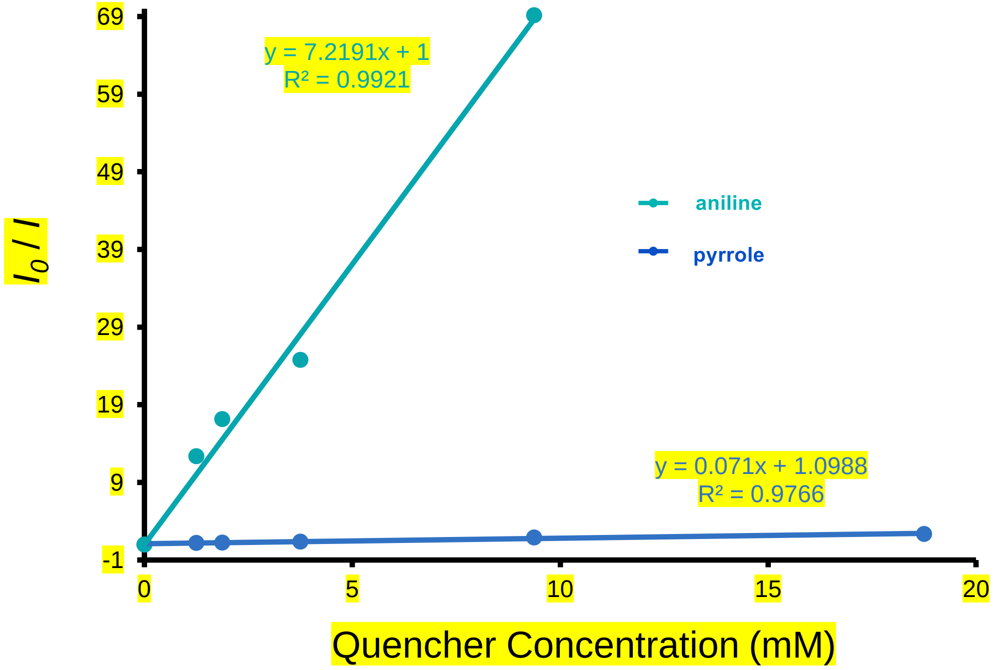

Figure S5: Luminescence quenching of the excited state of photocatalyst by additive **2** and **8** in MeCN.

3. MECHANISTIC INVESTIGATIONS

3.1. UV/vis Absorption Spectroscopy

UV/vis absorption spectra were recorded on a Jasco V-730 spectrophotometer, equipped with a temperature control unit at 25 °C. The samples were measured in Starna® fluorescence quartz cuvettes (type: 29-F, chamber volume = 1.400 mL, H × W × D = 48 mm × 12.5 mm × 12.5 mm, path length = 10 mm).

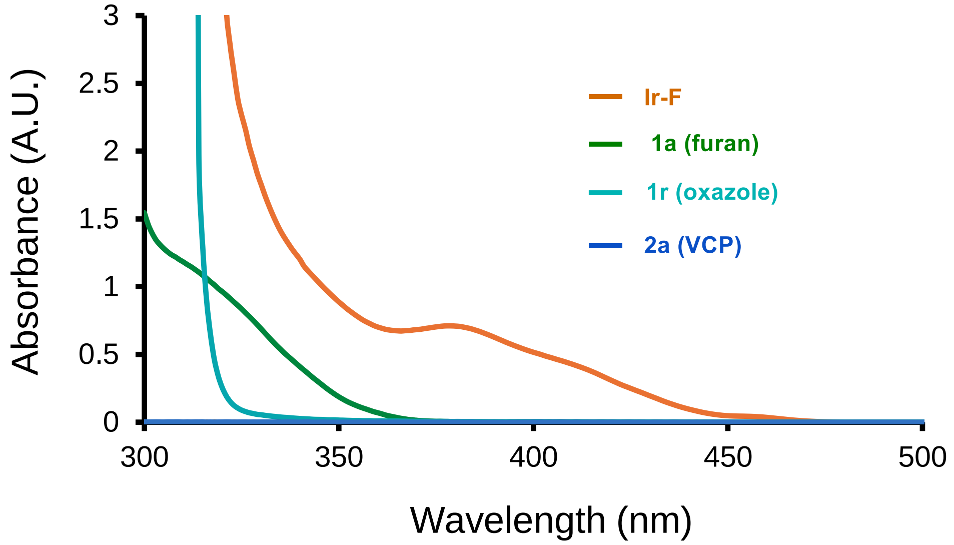

Figure S6: UV/vis absorption spectra of furan (**1a**), oxazole (**1r**), Ir-F and the VCPs **2a** in MeCN.

UV/vis spectroscopy of the individual reaction components revealed that the photocatalyst Ir-F is the only light absorbing species near λ = 450 nm, eliminating the possibility that direct excitation at 450 nm of either furan and oxazole (**1a** and **1r**) or VCP **2a** are responsible for reactivity.

3.2. Stern-Volmer Analysis

Quenching studies were carried out on a BMG LABTECH VANTAStar microplate spectrofluorometer using a 96-microtiter plate in a nitrogen-filled glovebox. The following parameters were set: slot width = 8 nm, excitation wavelength λ_{ex} = 400 nm, measured luminescence wavelength λ = 473 nm, temperature = 25 °C. All samples were prepared with degassed MeCN in the glovebox to minimize oxygen exposure. The quenching studies were performed using a solution of photocatalyst (6.25 µM). The varying concentrations of the potential quencher were achieved by dilution of the respective stock solutions (50 mM for **1a** and **2a**, 10 mM for **1r**) in a seven-step concentration series (one well-plate column per quencher).

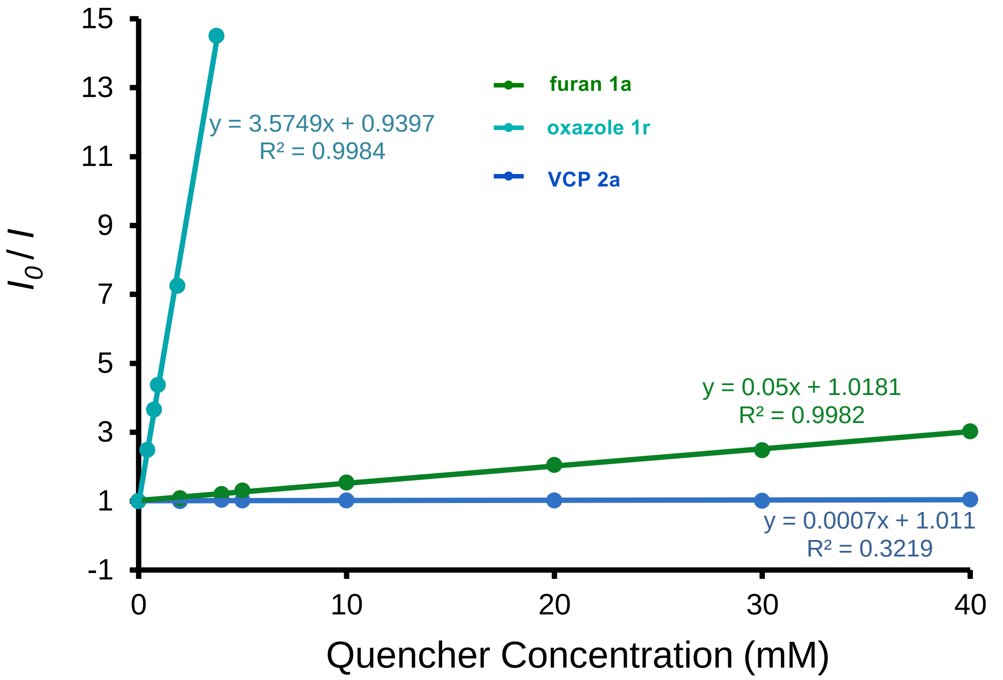

Figure S7: Luminescence quenching of the excited state of photocatalyst using **1a**, **1r** and **2a** in MeCN.

Stern–Volmer quenching studies clearly demonstrated that furan **1a** and oxazole **1r** are effective quenchers of the photocatalyst excited state, whereas VCP **2a** gave no indication that it can interact with this excited state species.

3.3. Cyclic Voltammetry

3.3.1. Redox potential measurements

Cyclic voltammograms (CVs) were collected at room temperature using a Metrohm Dropsens µStat-i 400s potentiostat. A 2 mm glassy carbon disc electrode, an Ag/AgCl (2 M LiCl in ethanol) electrode, and a platinum sheet electrode were used as the working, reference, and counter electrodes, respectively and were all supplied by Metrohm. The electrolyte solution contained 0.1 M tetrabutylammonium hexafluorophosphate ($TBAPF_6$) and 10 mM of the given substrate in MeCN. Before each measurement, the solution was purged with N_2 gas to avoid the interference of atmospheric oxygen. The scan rate was set at 0.1 V/s (E_{step} = 0.002 V) and 10 scans were taken for each compound in the potential window of −2.1 V to +2.1 V.

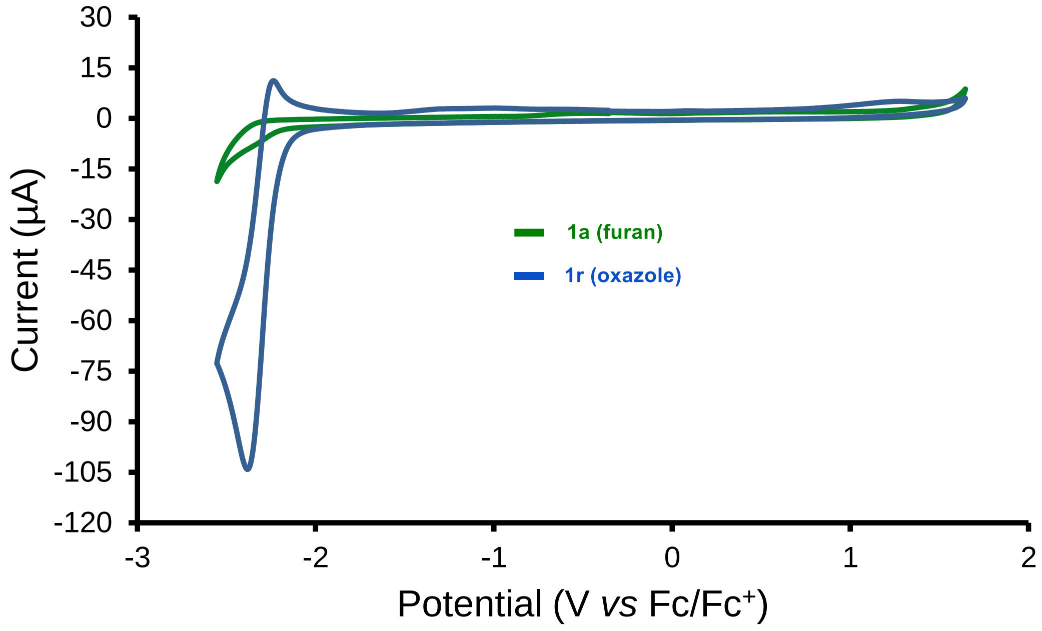

Figure S8: Cyclic voltammetry of **1a**, **1r** in 0.1 M $TBAPF_6$, using a 2 mm glassy carbon disk working electrode, Pt sheet counter electrode and an Ag/AgCl (2 M LiCl in ethanol) reference electrode. Set at 0.1 V/s scan rate. Potentials are referenced against Fc^+/Fc.

The half peak potential of Fc^+ /Fc in 0.1 M solution of $TBAPF_6$ in acetonitrile amounts to 0.454 V vs saturated calomel electrode (SCE), thus the half peak potentials for the substrates were converted to SCE by adding this value to $E_{1/2}$ (vs Fc+ /Fc).

The CV measurements show no oxidation for either **1a** and **1r** in the redox window -2.1 V to +2.1 V (*vs* SCE). Further, the CV shows no reduction of the furan **1a**, however **1r** is reduced at -2.30 V *vs* Fc^+/Fc (-1.85 V *vs* SCE).

Considering the redox potentials of **Ir-F** ($E_{1/2}$ PC^*/PC^- = 1.21 V *vs* SCE and $E_{1/2}$ PC^*/PC^+ = -0.89 V), neither oxidation or reduction of **1a** and **1r** are feasible.

3.4. Quantum Yield Calculation

3.4.1. Determination of the photon flux

First, the photon flux of a blue LED (3 W, λ_{max} = 420 nm) was determined by standard ferrioxalate actinometry according to a modified literature procedure by Yoon and coworkers[43]. Two solutions were prepared and stored in the dark. All following steps were also carried out in a darkened lab to prevent undesired irradiation.

Solution 1: Potassium ferrioxalate hydrate (737 mg, 1.50 mmol) was dissolved in aq. H_2SO_4 (0.05 M, 10 mL) to afford a 0.15 M ferrioxalate solution (attention: light sensitive!).

Solution 2: 1,10-Phenanthroline monohydrate (20 mg, 0.10 mmol) and NaOAc (4.5 g) were dissolved

in aq. H_2SO_4 (0.50 M, 20 mL).

To determine the photon flux, the reduction of $[Fe(C_2O_4)_3]^{3-}$ to $[Fe(C_2O_4)_2]^{2-}$ over time is measured[44,45]. Accordingly, **Solution 1** (1 mL) was irradiated for 60 s at λ_{max} = 420 nm (distance: 5 cm) in a 10 mL Schlenk tube. Subsequently, **Solution 2** (175 µL) was added and the mixture was stirred for 1 h to ensure that all Fe(II)-ions were coordinated by phenanthroline. The absorbance of the solution was then measured at λ = 510 nm. In addition, the absorbance of a non-irradiated control sample was measured. The same procedure was repeated two times. The average absorbance of the three irradiated samples and the three control samples were used to calculate the generated amount of Fe(II) ($n_{Fe(II)}$) according to the Lambert–Beer law (Equation 1), where V is the total volume ($1.175 \cdot 10^{-3}$ L), ΔA (510 nm) is the difference in absorbance between the irradiated and non-irradiated control samples (at λ = 510 nm), l is the path length of the cuvette (1.0 cm), and ε is the molar attenuation coefficient of the ferrioxalate actinometer at λ = 510 nm (11100 L mol^{-1} cm^{-1})[44].

$$n_{Fe(II)} = \frac{V \bullet \Delta A(510\ \mathrm{nm})}{l \bullet \varepsilon} \tag{1}$$

The photonflux (ϕ_q) can be calculated using Equation 2, where ϕ_F is the quantum yield of the ferrioxalate actinometer (1.13 at λ = 392 nm) and t is the irradiation time (60 s)[44,46].

$$\phi_q = \frac{n_{Fe(II)}}{\phi_F \bullet t \bullet f} \tag{2}$$

The fraction of light absorbed at λ = 420 nm by the actinometer (f) is calculated by using Equation 3, where A(420 nm) is the absorbance of **Solution 1** at λ = 420 nm.

$$f = 1 - 10^{-A(420\ nm)} \tag{3}$$

In this case, the absorbance A(420 nm) of **Solution 1** was 2.705.

Table S8: Determination of the photon flux.

	Run 1	Run 2	Run 3	Average
A(510 nm)	2.716	2.695	2.526	2.645
$A_{control}$(510 nm)	0.228	0.221	0.202	0.217
ΔA(510 nm) =	2.428			
ϕ_q =	$3.80 \cdot 10^{-9}$ mol·s^{-1}			
f =	0.998			

3.4.2. Determination of the reaction quantum yield

To benchmark the quantum yield of our (5+4) cycloaddition reaction, the quantum yield of the standard reaction with furan **1a** and VCP **2a** was determined.

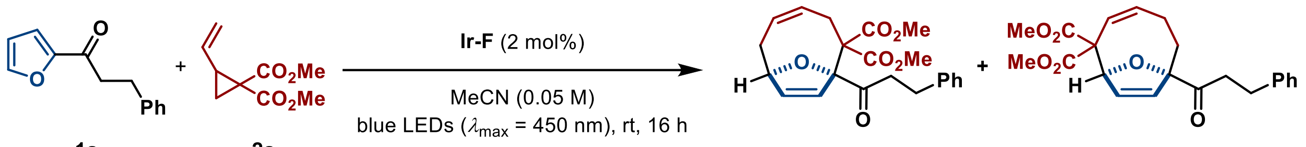

To an oven-dried 10 mL Schlenk tube equipped with a Teflon-coated magnetic stir bar was added furan **1a** (20.0 mg, 0.1 mmol, 1.0 eq) and Ir-F (2.0 mg, 0.002 mmol, 2 mol%). The Schlenk tube was evacuated and backfilled with argon three times. Subsequently, dry MeCN (2 mL, 0.05 M) and VCP **2a** (46.0 mg, 0.25 mmol, 2.5 eq.) were added under positive argon pressure. The reaction mixture was then stirred under irradiation in the calibrated set-up (3 W, λ = 420 nm) for the specified time. After this time, the solvent was removed under reduced pressure and the yield was determined by quantitative NMR spectroscopy using dibromomethane as an internal standard. The quantum yield (ϕ) of the reaction can be calculated using Equation 4, where ϕ_q is the photon flux and t is the irradiation time. The fraction of light absorbed (f_R) by the reaction was determined by measuring the absorbance of a non-irradiated control reaction (Equation 3).

$$\phi = \frac{n_{product}}{\phi_q \bullet t \bullet f_R} \quad (4)$$

Table S9: Determination of the reaction quantum yield.

	Experiment 1	Experiment 2	Experiment 3
Time (s)	18000	18000	18000
$n_{product}$ (mol)	$8.0 \cdot 10^{-6}$	$8.0 \cdot 10^{-6}$	$8.0 \cdot 10^{-6}$
ϕ =	0.113	0.113	0.113
ϕ (Average) =	0.113		

3.5. Trapping Experiments

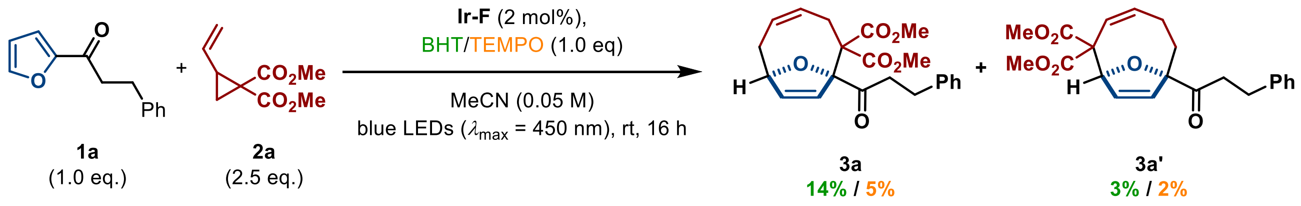

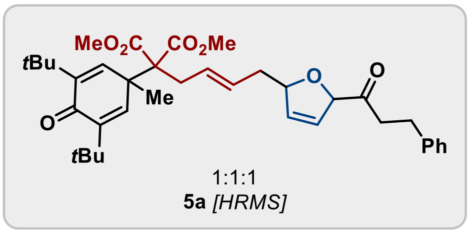

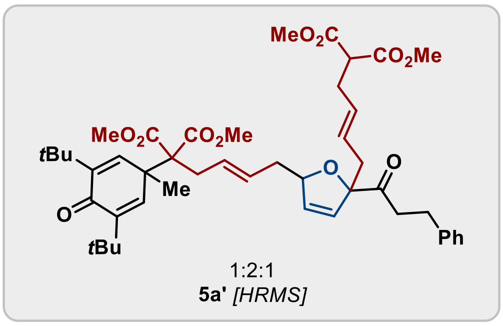

Figure S9: Effect of the trapping agents TEMPO and BHT on the (5+4) cycloaddition reaction.

When the radical trapping agents 2,2,6,6-tetramethylpiperidin-1-yloxyl (TEMPO) or butylated hydroxytoluene (BHT) were added to the standard reaction, product formation was completely suppressed, when adding 5.0 eq. of TEMPO. Addition of the radical trap BHT lead to the observation of two different trapping adducts by high resolution mass spectrometry (HRMS), the 1:1:1 adduct **5a** as well as the 1:2:1 adduct **5a'**, containing two VCP molecules. This suggests that a carbon-centered radical on the furan core is present in the reaction mechanism.

5a: HRMS (ESI$^+$): m/z calc'd for $C_{37}H_{48}O_7Na$ $[M+Na]^+$: 627.3297 found: 627.3286.

5a': HRMS (ESI$^+$): m/z calc'd for $C_{46}H_{60}O_{11}Na$ $[M+Na]^+$: 811.4033 found: 811.4023.

4. COMPUTATIONAL CALCULATIONS

4.1. Computational Methods

All computations were done using ORCA 6.0.1 software package.[47] Geometry optimization of all relevant stationary points was performed using the B3LYP functional[48] on a recontracted Ahlrichs double-ζ-basis (def2-SVP)[49] applying the CPCM continuum solvation model for acetonitrile[50,51] and a Grimme D3 atom-pairwise dispersion correction.[52] All optimized geometries were confirmed to be local minima on the respective potential energy surface by the absence of imaginary frequencies, as obtained from the vibrational frequency calculations at the same level of theory. In most cases, a conformational search was carried out using the GOAT implementation at the GFN2-xTB level.[53]

Relaxed surface scan and Nudged elastic band calculations were performed to identify transition state geometries.[54] Transition states were characterized by one negative eigenvalue of the Hessian and the analysis of the corresponding eigenvector vibration. Intrinsic reaction coordinate (IRC) calculations were carried out to confirm the transition states are connected to the correct minima.

The electronic energy of the previously optimized geometries was then refined through an additional single point calculation on the ωB97X-D3 functional[55] and def2-TZVPP basis set,[49] with the CPCM continuum solvation model for acetonitrile.[50,51] The reported Gibbs free energies at 298 K were obtained as the sum of the electronic energies and the corresponding free energy corrections (ZPVE, thermal corrections, enthalpy correction, entropic corrections), as obtained from the frequency calculation.

4.2 DFT Calculation determining C5/C2 regioselectivity

Table S10: Summary of energies.

Name	Electronic energy / E_h	ZPVE / E_h	Thermal correction / E_h	Enthalpy correction / E_h	Entropy correction / E_h
**1a**	-653.11115	0.21791524	0.01249155	0.00094421	-0.05177592
**1a***	-653.01096	0.21372964	0.01299924	0.00094421	-0.05348447
**2a**	-651.14385	0.1993396	0.01424541	0.00094421	-0.05529699
**TS-I**	-1304.1478	0.41387186	0.02843178	0.00094421	-0.08682166
**TS-IB**	-1304.1467	0.41461999	0.02788287	0.00094421	-0.08505067
**Int-I**	-1304.2117	0.41860237	0.02729294	0.00094421	-0.08303086
**Int-IB**	-1304.1927	0.41700263	0.02757352	0.00094421	-0.08336964
**1c**	-474.968686	0.10652778	0.00856539	0.00094421	-0.04323878
**1c***	-474.871326	0.10228475	0.00921546	0.00094421	-0.04580253
**1d**	-457.339727	-457.955415	0.11231571	0.00797548	0.00094421
**1d***	-457.239274	-457.854664	0.10818909	0.00855962	0.00094421

**1f**	-573.714803	-574.468041	0.1611672	0.00994246	0.00094421
**1f***	-573.619638	-574.370043	0.15720041	0.01040103	0.00094421
**1i**	-321.872964	-322.297929	0.06893095	0.00528275	0.00094421
**1i***	-321.763782	-322.187346	0.06377788	0.00620044	0.00094421
**1j**	-457.35351	-457.966067	0.11281394	0.00802533	0.00094421
**1j***	-457.243373	-457.855862	0.10784683	0.00889398	0.00094421
**1k**	-679.603689	-680.502164	0.08478239	0.00892451	0.00094421
**1k***	-679.510876	-680.410182	0.08063034	0.00947869	0.00094421
**1at**	-342.937426	-343.393288	0.07955473	0.00551362	0.00094421
**1at***	-342.839506	-343.295664	0.07540221	0.00602993	0.00094421

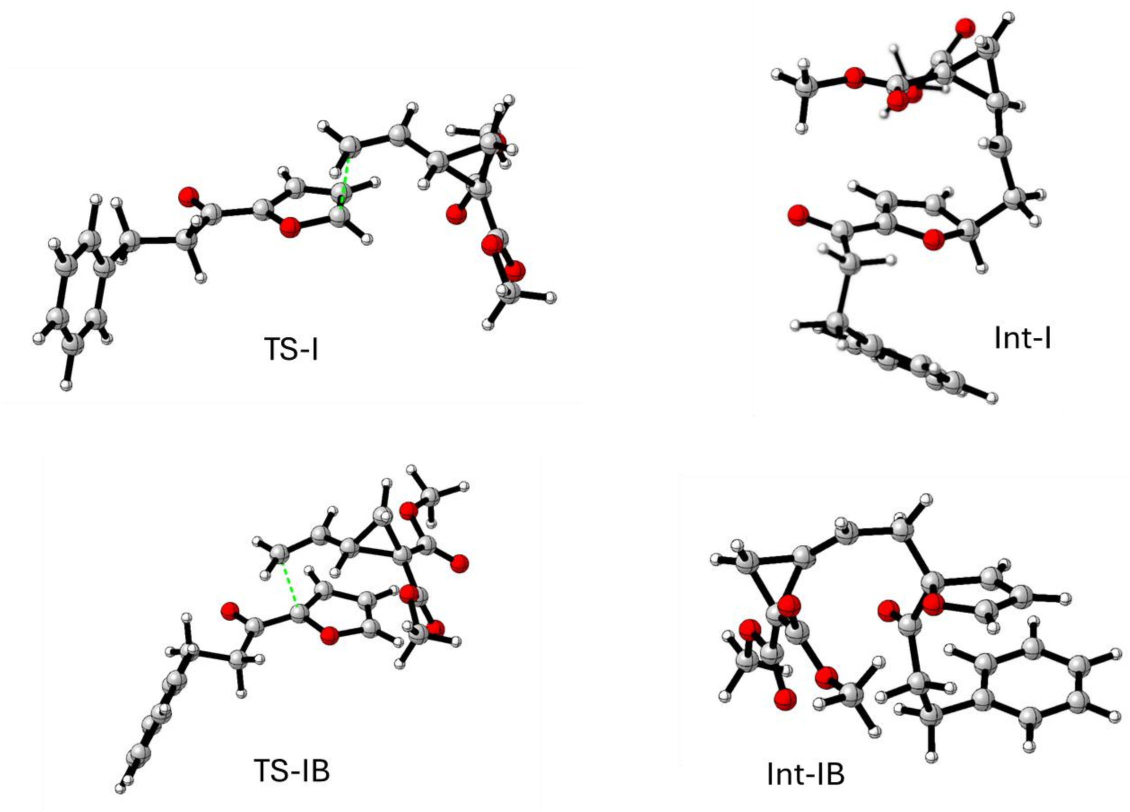

Figure S10: Optimized geometries for transition states and Int-I/Int-IB.

Table S11: Summary of spin density of different furans and their corresponding r.r. obtained from the (5+4) cycloaddition. r.r. ratio denotes (3:3') that is the ratio between the (5+4) cycloadducts formed from the radical addition from C5 vs C2 position.

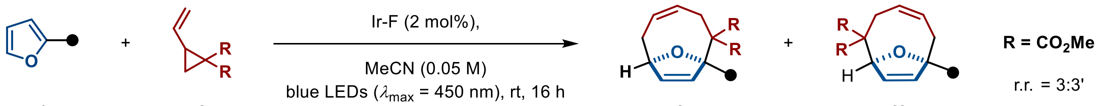

1 2 3 3'

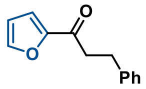

1a, E_T : 59.5

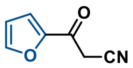

1c, E_T : 57.2

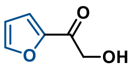

1d, E_T : 59.7

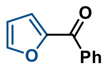

1f, E_T : 58.1

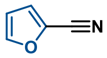

1i, E_T : 65.1

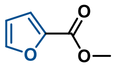

1j, E_T : 65.0

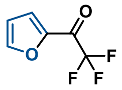

1k, E_T : 54.1

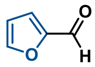

1at, E_T : 57.8

Name	Spin density at C5	Spin density at C2	Difference in spin density (C5 - C2)	Regioisomeric ratio (3:3')	Regioisomeric excess (%3 - %3')
1a	0.653816	0.497117	0.156699	83:17	66
1c	0.675916	0.487101	0.188815	95:5	90
1d	0.693167	0.522521	0.170646	81:19	62
1f	0.648908	0.457013	0.191895	90:10	80
1i	0.734509	0.612901	0.121608	63:37	26
1j	0.737697	0.62542	0.112277	36:64	-28
1k	0.64744	0.429196	0.218244	95:5	90
1at	0.669378	0.497141	0.172237	95:5	90

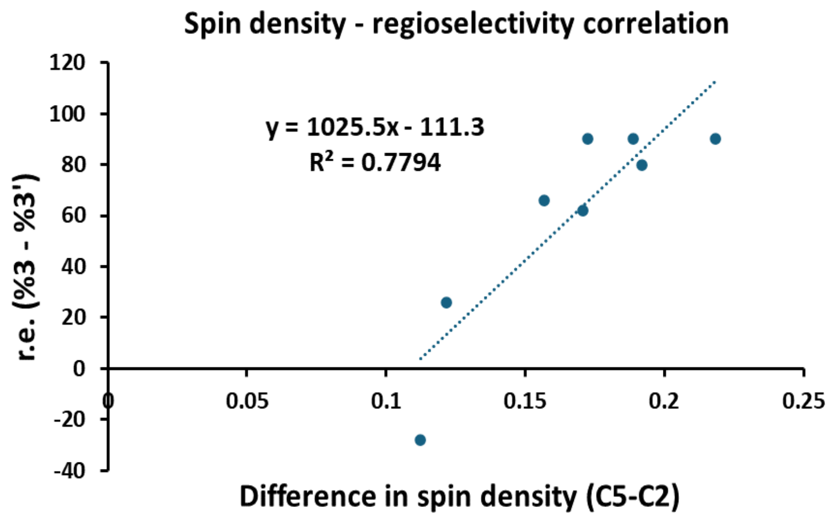

Figure S11: Spin density – regioselectivity correlation.

To rationalize the regioselectivity observed across different C2-substituted furan substrates, the spin density of the following furans (**1a**, **1c**, **1d**, **1f**, **1i**, **1j**, **1k**, **1at**) were calculated based on Mulliken populaton analysis. In addition, the regioisomeric excess (%3 − %3′) was determined, defined as the difference in the percentage of (5+4) cycloadducts formed via addition at the C5 position (3) versus the C2 position (3′).

As shown in **Fig. S11**, a clear relationship is observed between the difference in spin density at the C5 and C2 positions and the corresponding difference in the proportion of regioisomers formed. Substrates exhibiting high regioselectivity display a larger difference in spin density (e.g., **1c**, **1f**, **1k**, and **1at**), whereas those with lower regioselectivity show a smaller difference (e.g., **1i** and **1j**).

While spin density provides a useful descriptor for predicting regioselectivity, additional factors also contribute, including the polarity of the triplet excited 1,4-biradical, which can be influenced by substituents. Furthermore, steric effects are not captured by this descriptor. Overall, this analysis provides a preliminary rationale for the observed regioselectivity across different furans in the (5+4) cycloaddition.

4.2.1 Cartesian coordinates

1a

27

O	2.05402051	-1.8089767	1.726907896
C	1.57801526	-0.7282813	1.397410701
C	0.58054425	-0.0001692	2.277326228
C	-0.8583237	-0.5332532	2.048197944
C	-1.376365	-0.2558369	0.656748437

C	-1.164967	-1.1721819	-0.385089542
C	-1.5588345	-0.8746471	-1.693019369
C	-2.1803966	0.34575452	-1.978385829
C	-2.4084343	1.26176077	-0.945721597
C	-2.0069404	0.96156459	0.359029422
C	1.90842849	-0.1478504	0.09598444
C	2.64482866	-0.6483801	-0.955289317
C	2.55514342	0.31096677	-2.002553749
C	1.77234235	1.32844085	-1.514392701
O	1.3829015	1.06511754	-0.252782653
H	0.8688929	-0.1921018	3.32178129
H	0.60760066	1.08283713	2.091264051
H	-1.5151634	-0.0612735	2.795226115
H	-0.8612357	-1.6172437	2.244677631
H	-0.6762753	-2.1263927	-0.169247907
H	-1.380169	-1.5989533	-2.492183752
H	-2.4893537	0.58018666	-3.000416598
H	-2.899273	2.21540684	-1.158031042
H	-2.1784389	1.68616342	1.160636572
H	3.16920836	-1.6013419	-0.96195688
H	3.00582749	0.26072701	-2.991768215
H	1.42639644	2.26799738	-1.939911575

1a*

27

O	1.93110207	-1.1695136	-2.304100178
C	1.6033268	-0.188749	-1.567293036
C	0.94370937	1.04855593	-2.137241992
C	-0.5833436	0.82069311	-2.305119211
C	-1.2228649	0.43703751	-0.992607967
C	-1.2633377	-0.9090514	-0.588719755
C	-1.7109775	-1.2637899	0.68765568

C	-2.1264022	-0.2744642	1.582963498
C	-2.1088113	1.06876629	1.185485863
C	-1.6613201	1.41782497	-0.091130622
C	1.72276904	-0.3376838	-0.168492367
C	2.11939825	-1.531179	0.609066695
C	1.98055224	-1.18573	1.932995914
C	1.54001346	0.15004187	1.986201942
O	1.40627773	0.66963583	0.70278586
H	1.11240836	1.91744467	-1.483045686
H	1.39181505	1.26145065	-3.120496287
H	-0.7362756	0.02075317	-3.047065782
H	-1.0409567	1.73924988	-2.705601596
H	-0.9253848	-1.6850589	-1.280268442
H	-1.7258762	-2.3154212	0.985414623
H	-2.4688118	-0.5464627	2.584747761
H	-2.4410273	1.84738965	1.877441701
H	-1.6305844	2.47027828	-0.388049794
H	2.43423244	-2.4727358	0.168837106
H	2.1667663	-1.8106457	2.805550282
H	1.29359291	0.82140252	2.804514787

2a

25

C	1.42974644	-2.1850493	2.497991836
C	1.13116573	-1.9441657	1.216263846
C	-0.2288063	-1.6139514	0.73384944
C	-0.6711738	-1.9662443	-0.65292001
C	-0.4176127	-0.5105918	-0.327954313
C	0.8333165	0.12455627	-0.869953134
O	1.17014955	1.20266568	-0.159357427
C	2.35132021	1.90470377	-0.568126657
O	1.49364782	-0.3114033	-1.787302718

C	-1.6351244	0.34828383	-0.168423044
O	-1.5650525	1.4555061	-0.910469698
C	-2.6926943	2.34117115	-0.864183526
O	-2.595572	0.04905612	0.508225733
H	2.44850007	-2.4363359	2.805769677
H	0.66395838	-2.1435099	3.279917629
H	1.91820644	-1.9985571	0.454693264
H	-1.0106108	-1.5937014	1.498366191
H	0.05798967	-2.4292261	-1.322517153
H	-1.7120403	-2.262379	-0.799457565
H	3.23249325	1.25074306	-0.491511207
H	2.25111267	2.25485898	-1.606023326
H	2.44877496	2.75732224	0.113945385
H	-3.5957124	1.83192424	-1.231577252
H	-2.8664749	2.68775092	0.164937866
H	-2.4394971	3.18660303	-1.51422382

TS-I

52

O	-0.3329954	-3.2941428	3.147844809
C	-0.6888992	-2.3405714	2.4058074
C	-2.1389092	-2.0911323	2.027786683
C	-3.0973692	-3.1232083	2.629809823
C	-4.5332082	-2.858134	2.247395767
C	-5.0739853	-3.3943138	1.067565792
C	-6.390867	-3.1159227	0.68857999
C	-7.1919915	-2.2914918	1.486208209
C	-6.6654726	-1.7504118	2.664151116
C	-5.3478528	-2.0320697	3.038392567
C	0.29957021	-1.4674703	1.877664743
C	1.74581528	-1.4669328	2.075492395
C	2.23655509	-0.4194225	1.355679148

C	1.1377137	0.21460052	0.680860988
O	-0.0490351	-0.4348646	1.044897672
H	-2.4259347	-1.0719185	2.345909479
H	-2.2301379	-2.0810263	0.926173136
H	-2.989666	-3.1136205	3.725747061
H	-2.7962034	-4.1282147	2.29460892
H	-4.4529356	-4.0413462	0.440723749
H	-6.7943203	-3.5468354	-0.231886495
H	-8.2229218	-2.0754364	1.193650242
H	-7.2846209	-1.1079014	3.296295092
H	-4.9423344	-1.6074998	3.961732783
H	2.27963156	-2.1858232	2.69122856
H	3.26830663	-0.0834785	1.267182544
H	1.03828607	1.24770682	0.354963036
H	0.00163336	5.28803812	-3.776975136
C	0.74028623	5.32421786	-2.967900702
H	0.24156806	5.48532135	-2.000982152
O	1.40483556	4.05440375	-2.982731503
H	1.14575972	1.64965701	-3.020902545
H	1.46041991	6.13696754	-3.142612956
C	2.1737469	1.35011589	-2.803579177
H	3.34805859	-0.3901308	-2.143799584
C	2.33326993	0.0183112	-2.195692241
C	2.37075455	3.86654733	-2.075257708
H	2.95659237	2.62762486	-4.445308536
C	3.25420416	1.98155588	-3.617731841
O	2.68853202	4.70103121	-1.256452479
C	3.02725758	2.52947712	-2.225223573
C	1.30798746	-0.6543024	-1.61439375
H	1.45633914	-1.6539174	-1.201505206
H	4.17648899	1.41538003	-3.760917225
H	0.27685836	-0.3046142	-1.714908593

C	3.93839229	2.15587072	-1.088832881
O	3.6284496	2.2527027	0.078704727
O	5.09971408	1.64659252	-1.504117214
H	5.51498766	0.29690005	0.034028276
C	5.98350025	1.14206679	-0.491996821
H	6.22842937	1.93145167	0.232611989
H	6.88571587	0.80961372	-1.017988377

TS-IB

52

O	3.41315368	-1.7548166	1.59343873
C	3.07182076	-0.6114767	1.225281695
C	1.61383518	-0.215782	1.073970026
C	0.72573989	-1.4005011	0.665259961
C	-0.7307497	-1.0185812	0.575242147
C	-1.2618006	-0.4868371	-0.611120833
C	-2.6004683	-0.0900156	-0.685012198
C	-3.4333428	-0.2174005	0.432174424
C	-2.9166305	-0.7450128	1.620496552
C	-1.5771508	-1.1410081	1.688784357
C	4.08263625	0.3497983	0.870234802
C	5.52127857	0.29593404	1.178019566
C	5.96421245	1.61000471	1.18528209
C	4.88342853	2.42886728	0.870988193
O	3.74238311	1.67521229	0.658449937
H	1.26663801	0.18418725	2.045543031
H	1.51244041	0.61192008	0.355726355
H	0.86561997	-2.2099542	1.396905585
H	1.07277551	-1.7848913	-0.308208782
H	-0.615236	-0.3865537	-1.488214835
H	-2.996122	0.31763055	-1.619356651
H	-4.4808966	0.09006674	0.375929715

H	-3.5606431	-0.8520407	2.497705567
H	-1.1787895	-1.5556061	2.619699632
H	6.07386739	-0.6176745	1.377731226
H	6.97418592	1.96539863	1.381870468
H	4.79073199	3.49633683	0.693324277
H	2.05273271	5.21882714	-3.927801186
C	2.66667614	5.28323608	-3.022014265
H	2.05483166	5.09353948	-2.12799911
O	3.67721175	4.27530614	-3.157735437
H	3.56675059	1.78740586	-2.506256417
H	3.12659766	6.27880385	-2.94087492
C	4.64365044	1.59699381	-2.508051915
H	6.07831114	0.06730037	-1.839845444
C	5.02162948	0.33250315	-1.875140342
C	4.52950397	4.13251967	-2.13552532
H	4.802088	2.65466911	-4.45489538
C	5.38778406	2.20180101	-3.654638518
O	4.50255777	4.84373618	-1.15483682
C	5.4452598	2.95598535	-2.341459161
C	4.08379767	-0.4981242	-1.314265216
H	4.36315627	-1.4931061	-0.964582773
H	6.31918338	1.71690413	-3.952046788
H	3.01882638	-0.3026002	-1.462582638
C	6.77359999	3.03308687	-1.645244793
O	7.19772601	4.00481227	-1.061890849
O	7.49004878	1.91146406	-1.804693724
H	8.77891513	2.05549263	-0.16543942
C	8.8123851	1.90158071	-1.253364892
H	9.4243614	2.69238115	-1.710840177
H	9.22717701	0.91378691	-1.484929531

Int-I

52

O	3.72308953	-1.9954548	2.166853228
C	2.58659339	-1.9702668	1.659447898
C	1.69996808	-3.2004293	1.657622253
C	0.93191947	-3.3352595	2.998130392
C	0.03761483	-2.1494582	3.27280829
C	-1.2609185	-2.0926155	2.744821506
C	-2.0511654	-0.9511629	2.903697588
C	-1.5516213	0.15810902	3.595952315
C	-0.2625156	0.11005104	4.136100061
C	0.52229136	-1.0362307	3.976340493
C	2.05318391	-0.7450316	1.129140782
C	2.61091412	0.56692574	1.173610075
C	1.71281327	1.41863952	0.614255074
C	0.50845914	0.63903077	0.181916175
O	0.84064462	-0.7261833	0.535031568
H	0.98269432	-3.1588749	0.825257321
H	2.34808026	-4.0802885	1.524665427
H	1.66890825	-3.4457445	3.809757637
H	0.33645621	-4.2612354	2.964030074
H	-1.6514056	-2.9512923	2.190533185
H	-3.0600446	-0.9248654	2.483021686
H	-2.1665858	1.0536336	3.717504935
H	0.13536156	0.96933483	4.682491712
H	1.53285717	-1.0645847	4.39305803
H	3.58863164	0.80637544	1.584590367
H	1.82013526	2.49245666	0.469539951
H	-0.3898695	0.9129612	0.768854324
H	4.7981287	4.25831265	0.764638771
C	4.79395111	4.23328736	-0.331378465
H	5.80271952	4.43316912	-0.721885892
O	4.36577913	2.91586883	-0.694123996

H	1.88552595	2.31290014	-2.704444613
H	4.09889975	4.98826979	-0.727941497
C	2.24723084	1.27879791	-2.70397811
H	1.51768208	-0.7549165	-2.322422712
C	1.29364581	0.30582398	-2.205481496
C	4.29959998	2.64288848	-2.000290498
H	3.57060536	1.74471829	-4.428503205
C	3.3276585	0.98093402	-3.687194839
O	4.53158291	3.46573168	-2.862977759
C	3.79950514	1.25878038	-2.280534522
C	0.1625313	0.71196559	-1.318931925
H	-0.7132684	0.06178553	-1.481150549
H	3.42543118	-0.0566487	-4.014438334
H	-0.1508916	1.74983613	-1.524412489
C	4.31950802	0.08058423	-1.521514814
O	3.88120242	-1.0472825	-1.630488372
O	5.37415021	0.38509775	-0.762510644
H	5.30891283	-1.0870837	0.723359953
C	5.9928934	-0.6918814	-0.041899924
H	6.88381633	-0.2596557	0.429195419
H	6.27972935	-1.499403	-0.731191837

Int-IB

52

O	2.19055688	-0.6014297	-0.932412414
C	1.95386968	-0.359121	0.237166569
C	1.10149443	0.8123157	0.662932497
C	-0.0506927	1.09226733	-0.315509851
C	-1.0643039	-0.0286784	-0.314057829
C	-1.0343963	-1.0379421	-1.288717288
C	-1.9233445	-2.1166485	-1.232174393
C	-2.8583211	-2.2038153	-0.195776787

C	-2.9014706	-1.2009476	0.779619623
C	-2.0119211	-0.1249656	0.71817412
C	2.448338	-1.3345155	1.333527001
C	1.28055124	-2.2489267	1.617224414
C	0.87042622	-2.0134629	2.924500714
C	1.70106577	-1.0460301	3.471960001
O	2.63844182	-0.6183032	2.579513378
H	0.71950232	0.66190152	1.681305964
H	1.78635817	1.67587749	0.70516165
H	0.36064831	1.236337	-1.325279289
H	-0.5320137	2.03650971	-0.016535042
H	-0.2955405	-0.9806777	-2.09135409
H	-1.8839468	-2.8932353	-2.000906574
H	-3.552636	-3.0469074	-0.149500753
H	-3.6317848	-1.2572463	1.59142072
H	-2.04749	0.65308673	1.486598365
H	0.86016536	-2.9319657	0.88221738
H	0.04948217	-2.5016092	3.449312936
H	1.72825223	-0.5853735	4.457637268
H	2.84778419	1.76261026	-3.229858538
C	3.88421213	2.12407272	-3.30942553
H	3.88315356	3.22318132	-3.339081124
O	4.65908143	1.64589457	-2.205284555
H	4.69003737	-0.5099455	-1.096710276
H	4.3589002	1.71806359	-4.210512368
C	5.1994994	-0.2621964	-0.164054754
H	5.54799897	-1.0626406	1.845440873
C	4.95834732	-1.1708588	0.933021229
C	4.27668457	2.04872463	-0.979988504
H	6.74930883	0.85153482	-1.275559158
C	6.357545	0.65829184	-0.276346354
O	3.37869602	2.84727093	-0.81052167

C	5.06877557	1.36254068	0.080534618
C	3.75620969	-2.0610669	0.950672611
H	3.89566416	-2.8749574	1.677470363
H	7.09172661	0.63424781	0.531541056
H	3.58069409	-2.5127081	-0.038972234
C	4.92251846	1.73832455	1.523486094
O	5.76963994	1.49022992	2.359238752
O	3.75382613	2.30063812	1.8266012
H	2.49750001	2.98520138	3.262294206
C	3.5086487	2.564363	3.212809072
H	4.24288052	3.28231244	3.606209252
H	3.5623766	1.6307172	3.78950746

1c

15

O	-1.26298	-1.74983	1.294294
C	-0.9713	-0.96382	0.411584
C	-1.96739	-0.64834	-0.72343
C	-2.37783	0.751854	-0.75099
N	-2.69998	1.865129	-0.75544
C	0.324529	-0.30256	0.354581
C	1.414596	-0.34341	1.198475
C	2.396381	0.514185	0.626181
C	1.838032	1.016418	-0.52508
O	0.592358	0.531033	-0.6987
H	-2.85126	-1.28562	-0.5755
H	-1.50917	-0.89926	-1.69509
H	1.485024	-0.92289	2.116763
H	3.389796	0.735351	1.0116
H	2.199202	1.701752	-1.28923

1c*

15

O	-1.24867	-1.57406	1.381622
C	-0.96338	-0.85977	0.378531
C	-1.96504	-0.6355	-0.76078
C	-2.57817	0.69226	-0.70741
N	-3.04434	1.751974	-0.63921
C	0.302584	-0.24876	0.291824
C	1.445085	-0.31694	1.226871
C	2.43921	0.449545	0.663634
C	1.950792	0.972582	-0.54965
O	0.638503	0.541395	-0.76733
H	-2.7555	-1.39598	-0.67248
H	-1.48188	-0.75017	-1.74554
H	1.446846	-0.87965	2.156147
H	3.436444	0.637968	1.059718
H	2.377499	1.615096	-1.31594

1d

15

O	1.66974	-1.15471	1.016535
C	1.103183	-0.44259	0.193599
C	1.89942	0.111438	-0.97942
O	3.227882	-0.31643	-0.91037
C	-0.30778	-0.11	0.29933
C	-1.26879	-0.45219	1.226095
C	-2.47622	0.173023	0.800331
C	-2.16565	0.852246	-0.35281
O	-0.86345	0.688649	-0.66427
H	1.405604	-0.21661	-1.91599
H	1.818329	1.21674	-0.95947

H	3.271763	-0.87072	-0.10689
H	-1.11195	-1.07882	2.101706
H	-3.451	0.129709	1.282076
H	-2.75107	1.470248	-1.03046

1d*

15

O	1.63256	-1.09362	1.056965
C	1.087505	-0.38784	0.157638
C	1.933656	0.108578	-1.00813
O	3.244107	-0.36841	-0.86673
C	-0.28141	-0.06898	0.23533
C	-1.29109	-0.43417	1.256942
C	-2.46803	0.140345	0.844427
C	-2.22077	0.827415	-0.3641
O	-0.87744	0.691932	-0.73195
H	1.479178	-0.22934	-1.96187
H	1.905444	1.217927	-1.03199
H	3.224656	-0.87785	-0.03166
H	-1.0868	-1.03871	2.13633
H	-3.43758	0.094183	1.33919
H	-2.84402	1.418533	-1.03041

1f

21

O	-0.48048	-1.35566	-2.02354
C	-0.44273	-0.70128	-0.98349
C	0.857019	-0.16489	-0.46875
C	1.892804	0.015226	-1.40471
C	3.144101	0.475705	-0.99852

C	3.384385	0.743695	0.355188
C	2.367596	0.549629	1.295728
C	1.107241	0.103979	0.889169
C	-1.70301	-0.44428	-0.27376
C	-2.95978	-0.95777	-0.51648
C	-3.83638	-0.35706	0.432648
C	-3.05692	0.4873	1.183878
O	-1.77359	0.442869	0.767403
H	1.695453	-0.20952	-2.45453
H	3.936825	0.624312	-1.73609
H	4.366389	1.100441	0.676764
H	2.556354	0.744569	2.354372
H	0.32692	-0.04999	1.633243
H	-3.20606	-1.68358	-1.28815
H	-4.9058	-0.5229	0.547024
H	-3.27034	1.159235	2.012636

1f*

21

O	-0.61389	-0.96961	-2.11853
C	-0.47527	-0.46543	-0.94645
C	0.858264	-0.05479	-0.44795
C	1.951937	-0.22474	-1.32491
C	3.243524	0.132246	-0.94191
C	3.479276	0.669115	0.330412
C	2.40521	0.843192	1.210126
C	1.108425	0.487587	0.831793
C	-1.68627	-0.34873	-0.21789
C	-3.02783	-0.74564	-0.68228
C	-3.89689	-0.45151	0.346222
C	-3.14272	0.098027	1.39556

O	-1.79804	0.157483	1.042159
H	1.759948	-0.64345	-2.31381
H	4.073939	-0.00885	-1.63923
H	4.491715	0.948731	0.633079
H	2.576771	1.261005	2.205906
H	0.291793	0.6327	1.535954
H	-3.23106	-1.18035	-1.65594
H	-4.97483	-0.60541	0.371033
H	-3.394	0.468422	2.386651

1i

10

N	3.396746	-0.17766	-0.00422
C	2.234196	-0.1413	-0.01123
C	0.819854	-0.07639	-0.01293
C	-0.05711	0.933693	0.314407
C	-1.36351	0.396967	0.111987
C	-1.18479	-0.89208	-0.32056
O	0.130322	-1.19176	-0.40043
H	0.211918	1.93119	0.655758
H	-2.31384	0.90334	0.267661
H	-1.87378	-1.68599	-0.60043

1i*

10

N	3.418366	-0.14163	0.008969
C	2.234881	-0.15317	-0.01519
C	0.861466	-0.12937	-0.03073
C	-0.10076	0.960858	0.322422

C	-1.34365	0.430127	0.124063
C	-1.19904	-0.90858	-0.32597
O	0.15563	-1.25553	-0.42171
H	0.1852	1.953015	0.661991
H	-2.30132	0.926948	0.276804
H	-1.91077	-1.68265	-0.60064

1j

15

O	1.183665	-1.50097	-0.56015
C	0.810089	-0.48866	2.83E-05
C	-0.60413	-0.14192	0.171192
C	-1.73504	-0.81634	-0.22469
C	-2.84246	-0.02314	0.203429
C	-2.30221	1.072687	0.827306
O	-0.9521	1.011453	0.813188
O	1.63222	0.427145	0.526095
C	3.036436	0.169478	0.404121
H	-1.75679	-1.76448	-0.75775
H	-3.90122	-0.23526	0.068363
H	-2.74012	1.943782	1.310255
H	3.302167	-0.77256	0.906636
H	3.54229	1.013923	0.886846
H	3.327204	0.104826	-0.65505

1j*

15

O	1.149008	-1.4786	-0.54704
C	0.806519	-0.43868	0.027903
C	-0.56301	-0.08096	0.202959

C	-1.77823	-0.82463	-0.23145
C	-2.84238	-0.07223	0.177122
C	-2.34229	1.085623	0.833504
O	-0.93778	1.075432	0.8435
O	1.676848	0.461544	0.546476
C	3.060549	0.151049	0.394909
H	-1.76268	-1.7717	-0.76385
H	-3.9008	-0.2917	0.040621
H	-2.82094	1.938071	1.308662
H	3.308424	-0.80116	0.890199
H	3.61295	0.973696	0.865801
H	3.333789	0.074223	-0.6695

1k

14

O	-1.58282	1.880542	-0.65067
C	-1.21662	0.727822	-0.53048
C	-2.24019	-0.42232	-0.75173
F	-1.83233	-1.25127	-1.72478
F	-2.39425	-1.14428	0.369143
F	-3.42835	0.071096	-1.09589
C	0.134888	0.340562	-0.18184
C	1.253152	1.10609	0.085962
C	2.304071	0.191725	0.366154
C	1.755184	-1.06469	0.250034
O	0.452651	-0.98718	-0.07795
H	1.293839	2.193428	0.077869
H	3.335974	0.424405	0.620943
H	2.164793	-2.06592	0.368306

1k*

14

O	-1.49628	1.894134	-0.59218
C	-1.20716	0.660271	-0.51468
C	-2.26981	-0.40601	-0.75879
F	-1.89101	-1.29312	-1.7037
F	-2.53403	-1.12605	0.354585
F	-3.41704	0.149602	-1.15906
C	0.101385	0.258818	-0.18384
C	1.271352	1.106466	0.093285
C	2.31057	0.239799	0.358376
C	1.813382	-1.07015	0.25448
O	0.458045	-1.04437	-0.07559
H	1.261953	2.192705	0.077069
H	3.340602	0.49376	0.605472
H	2.258009	-2.05583	0.369638

1at

11

C	2.051076	-0.22635	-0.13226
C	0.608783	-0.08606	-0.04422
C	-0.21097	0.986557	0.240627
C	-1.54612	0.497317	0.177535
C	-1.44601	-0.83672	-0.14177
O	-0.15559	-1.19826	-0.27732
O	2.836663	0.688399	0.050338
H	2.393537	-1.25929	-0.38567
H	0.122873	1.997545	0.466396
H	-2.46617	1.054063	0.345052
H	-2.18807	-1.6172	-0.29872

1at*

11			
C	2.037285	-0.2514	-0.13768
C	0.637423	-0.1751	-0.06623
C	-0.22401	0.989097	0.241353
C	-1.51869	0.527096	0.183688
C	-1.47546	-0.84262	-0.1407
O	-0.1533	-1.26511	-0.292
O	2.750247	0.775247	0.077369
H	2.476335	-1.23888	-0.38718
H	0.146039	1.986784	0.461142
H	-2.43384	1.093634	0.352756
H	-2.24204	-1.59877	-0.29254

5. X-RAY CRYSTALLOGRAPHY

5.1. X-Ray Diffraction Data

X-ray crystal structure analysis of 3b (glo10915): A colourless, prism-shaped crystal was mounted on a MiTeGen micromount. The crystals were crystallised from ethyl acetate and *n*-pentane. Data for **3b** were collected from a single crystal in 3.17 hours at 100(2) K on a Bruker D8 VENTURE KAPPA diffractometer with a microfocus sealed tube using a multilayer mirror as monochromator and a Bruker PHOTON III CPAD detector. The diffractometer was equipped with an Oxford Cryostream 1000 low temperature device and used Mo K_α radiation (λ = 0.71073 Å). All data were integrated with SAINT V8.41 yielding 32351 reflections of which 3544 where independent and 84.3% were greater than $2\sigma(F^2)$.[56] A Multi-Scan absorption correction using SADABS 2016/2 was applied.[57] The structure was solved by Intrinsic Phasing methods with SHELXT 2018/2 and refined by full-matrix least-squares methods against F^2 using SHELXL-2019/2.[58,59] All non-hydrogen atoms were refined with anisotropic displacement parameters. All hydrogen atoms were refined isotropic on calculated positions using a riding model with their U_{iso} values constrained to 1.5 times the U_{eq} of their pivot atoms for terminal sp^3 carbon atoms and 1.2 times for all other carbon atoms. Crystallographic data for the structures reported in this paper have been deposited with the Cambridge Crystallographic Data Centre.[60] CCDC 2516935 contain the supplementary crystallographic data for this paper. These data can be obtained free of charge from The Cambridge Crystallographic Data Centre *via* www.ccdc.cam.ac.uk/structures. This report and the CIF file were generated using FinalCif.[61]

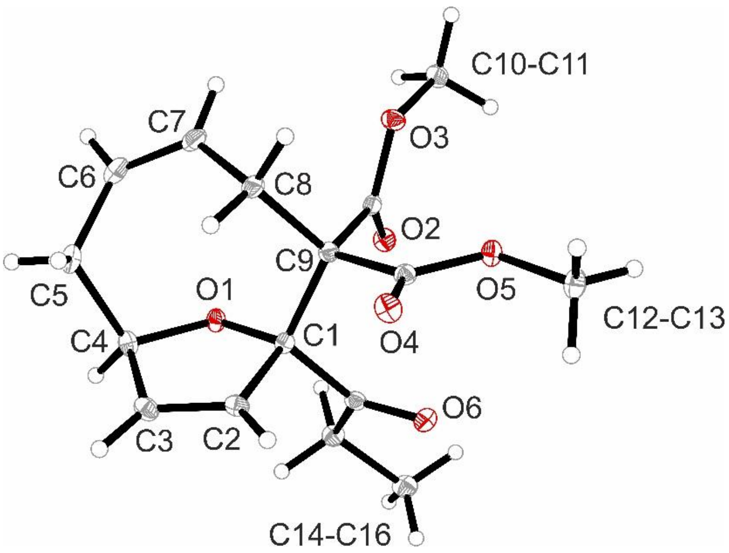

Figure S12: Crystal structure of compound **3b**. Thermal ellipsoids are shown at 30% probability.

Table S12. Crystal data and structure refinement for **3b**

CCDC number	2516935
Empirical formula	$C_{16}H_{20}O_6$
Formula weight	308.32
Temperature [K]	100(2)
Crystal system	monoclinic
Space group (number)	$P2_1/c$ (14)
a [Å]	14.8324(5)
b [Å]	7.7714(2)
c [Å]	14.6972(4)
α [°]	90
β [°]	113.9400(10)
γ [°]	90
Volume [Å^3]	1548.38(8)
Z	4
ρ_{calc} [gcm^{-3}]	1.323
μ [mm^{-1}]	0.101
F(000)	656

Crystal size [mm^3]	0.045×0.102×0.129
Crystal colour	colourless
Crystal shape	prism
Radiation	Mo K_α (λ=0.71073 Å)
2θ range [°]	5.57 to 54.98 (0.77 Å)
Index ranges	−19 ≤ h ≤ 19<br>−10 ≤ k ≤ 10<br>−19 ≤ l ≤ 19
Reflections collected	32351
Independent reflections	3544<br>Rint = 0.0454<br>Rsigma = 0.0231
Completeness to θ = 25.242°	99.9
Data / Restraints / Parameters	3544 / 0 / 202
Absorption correction Tmin/Tmax (method)	0.987 / 0.995 (Multi-Scan)
Goodness-of-fit on F^2	1.029
Final R indexes<br>[I≥2σ(I)]	R_1 = 0.0332<br>wR_2 = 0.0848
Final R indexes<br>[all data]	R_1 = 0.0424<br>wR_2 = 0.0921
Largest peak/hole [eÅ^{-3}]	0.33/−0.21

X-ray crystal structure analysis of 3b' (glo10919): A colourless, prism-shaped crystal was mounted on a MiTeGen micromount. The crystals were crystallised from ethyl acetate and *n*-pentane. Data for **3b'** were collected from a single crystal in 7.39 hours at 100(2) K on a Bruker D8 VENTURE KAPPA diffractometer with a microfocus sealed tube using a multilayer mirror as monochromator and a Bruker PHOTON III CPAD detector. The diffractometer used Mo K_α radiation (λ = 0.71073 Å). All data were integrated with SAINT V8.41 yielding 36209 reflections of which 3233 where independent and 60.7% were greater than 2σ(F^2).[56] A Multi-Scan absorption correction using SADABS 2016/2 was applied.[57] The structure was solved by Intrinsic Phasing methods with SHELXT 2018/2 and refined by full-matrix least-squares methods against F^2 using SHELXL-2019/2.[58,59] All non-hydrogen atoms were refined with anisotropic displacement parameters. All hydrogen atoms were refined isotropic on calculated positions using a riding model with their U_{iso} values constrained to 1.5 times the U_{eq} of their pivot atoms for terminal sp^3 carbon atoms and 1.2 times for all other carbon atoms. Crystallographic data for the structures reported in this paper have been deposited with the Cambridge Crystallographic Data Centre.[60] CCDC 2516936 contain the supplementary crystallographic data for this paper. These data can be obtained free of charge from The Cambridge Crystallographic Data Centre *via* www.ccdc.cam.ac.uk/structures. This report and the CIF file were generated using FinalCif.[61]

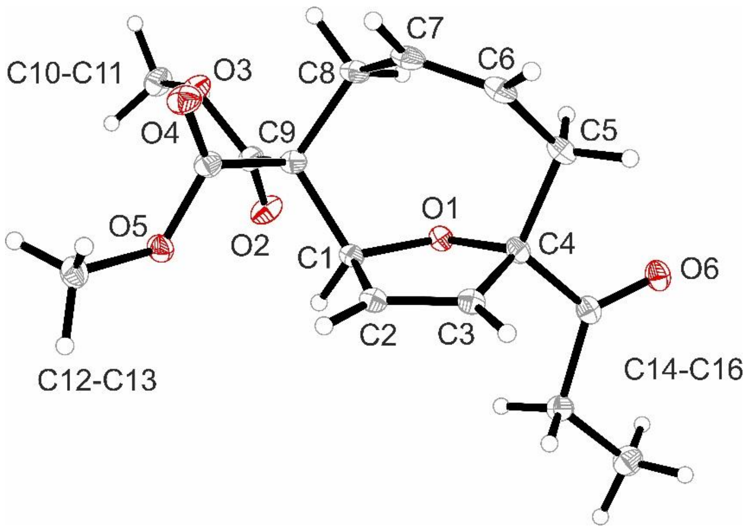

Figure S13: Crystal structure of compound **3b'**. Thermal ellipsoids are shown at 30% probability.

Table S13. Crystal data and structure refinement for **3b'**

CCDC number	2516936
Empirical formula	$C_{16}H_{20}O_6$
Formula weight	308.32
Temperature [K]	100(2)
Crystal system	monoclinic
Space group (number)	$P2_1/n$ (14)
a [Å]	6.8160(6)
b [Å]	23.606(2)
c [Å]	9.5508(10)
α [°]	90
β [°]	100.480(3)
γ [°]	90
Volume [Å^3]	1511.1(3)
Z	4
ρ_{calc} [gcm^{-3}]	1.355
μ [mm^{-1}]	0.104
F(000)	656
Crystal size [mm^3]	0.053×0.056×0.109
Crystal colour	colourless
Crystal shape	prism

Radiation	Mo K_α (λ=0.71073 Å)
2θ range [°]	4.67 to 53.63 (0.79 Å)
Index ranges	−8 ≤ h ≤ 8<br>−29 ≤ k ≤ 29<br>−12 ≤ l ≤ 12
Reflections collected	36209
Independent reflections	3233<br>Rint = 0.148<br>Rsigma = 0.0766
Completeness to θ = 25.242°	99.9
Data / Restraints / Parameters	3233 / 0 / 203
Absorption correction Tmin/Tmax (method)	0.989 / 0.995 (Multi-Scan)
Goodness-of-fit on F^2	1.015
Final R indexes<br>[I≥2σ(I)]	R_1 = 0.0530<br>wR_2 = 0.1264
Final R indexes<br>[all data]	R_1 = 0.1007<br>wR_2 = 0.1553
Largest peak/hole [eÅ^{-3}]	0.24/−0.24
Extinction coefficient	0.028(4)

X-ray crystal structure analysis of 3c (glo10867): A colourless, prism-shaped crystal was mounted on a MiTeGen micromount. The crystals were crystallised from *n*-pentane and ethyl acetate. Data for **3c** were collected from a single crystal in 5.03 hours at 100(2) K on a Bruker D8 VENTURE KAPPA diffractometer with a microfocus sealed tube using a multilayer mirror as monochromator and a Bruker PHOTON III CPAD detector. The diffractometer was equipped with an Oxford Cryostream 1000 low temperature device and used Mo K_α radiation (λ = 0.71073 Å). All data were integrated with SAINT V8.41 yielding 43660 reflections of which 3480 where independent and 85.9% were greater than 2σ(F^2).[56] A Multi-Scan absorption correction using SADABS 2016/2 was applied.[57] The structure was solved by Intrinsic Phasing methods with SHELXT 2018/2 and refined by full-matrix least-squares methods against F^2 using SHELXL-2019/2.[58,59] All non-hydrogen atoms were refined with anisotropic displacement parameters. All hydrogen atoms were refined isotropic on calculated positions using a riding model with their U_{iso} values constrained to 1.5 times the U_{eq} of their pivot atoms for terminal sp^3 carbon atoms and 1.2 times for all other carbon atoms. Crystallographic data for the structures reported in this paper have been deposited with the Cambridge Crystallographic Data Centre.[60] CCDC 2516937 contain the supplementary crystallographic data for this paper. These data can be obtained free of charge from The Cambridge Crystallographic Data Centre *via* www.ccdc.cam.ac.uk/structures. This report and the CIF file were generated using FinalCif.[61]

*Special refinement details for **3c**:* The entire molecule is disordered over two positions. Several restraints (SIMU, SAME, SADI, ISOR and DELU) were used in order to improve refinement stability.

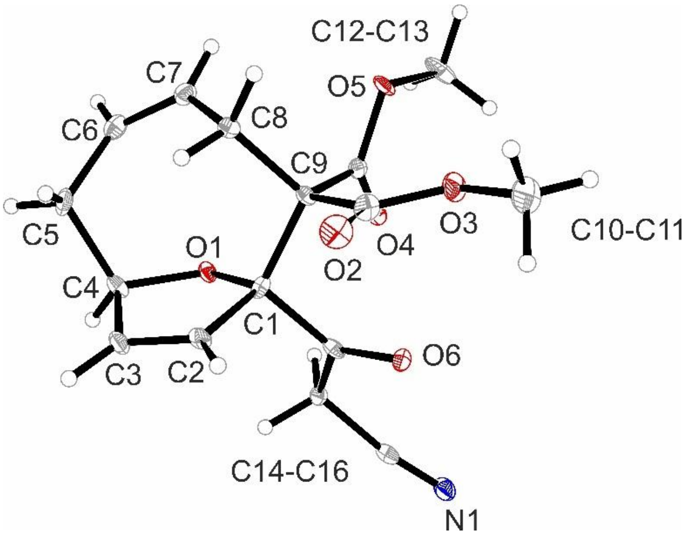

Figure S14: Crystal structure of compound **3c**. Thermal ellipsoids are shown at 30% probability.

Table S14. Crystal data and structure refinement for **3c**

CCDC number	2516937
Empirical formula	$C_{16}H_{17}NO_6$
Formula weight	319.3
Temperature [K]	100(2)
Crystal system	monoclinic
Space group (number)	$P2_1/n$ (14)
a [Å]	8.2022(3)
b [Å]	15.8345(5)
c [Å]	11.8302(4)
α [°]	90
β [°]	99.1070(10)
γ [°]	90
Volume [Å^3]	1517.11(9)
Z	4
ρ_{calc} [gcm^{-3}]	1.398
μ [mm^{-1}]	0.108
F(000)	672
Crystal size [mm^3]	0.055×0.067×0.153
Crystal colour	colourless
Crystal shape	prism

Radiation	Mo K_{α} (λ=0.71073 Å)
2θ range [°]	4.33 to 55.00 (0.77 Å)
Index ranges	−10 ≤ h ≤ 10<br>−20 ≤ k ≤ 20<br>−15 ≤ l ≤ 14
Reflections collected	43660
Independent reflections	3480<br>Rint = 0.0538<br>Rsigma = 0.0238
Completeness to θ = 25.242°	99.9
Data / Restraints / Parameters	3480 / 453 / 420
Absorption correction Tmin/Tmax (method)	0.984 / 0.994 (Multi-Scan)
Goodness-of-fit on F^2	1.080
Final R indexes<br>[I≥2σ(I)]	R_1 = 0.0320<br>wR_2 = 0.0789
Final R indexes<br>[all data]	R_1 = 0.0390<br>wR_2 = 0.0827
Largest peak/hole [eÅ^{-3}]	0.17/−0.14

X-ray crystal structure analysis of 3q (glo10857): A colourless, prism-shaped crystal was mounted on a MiTeGen micromount. The crystals were crystallised from *n*-pentane and ethyl acetate. Data for **3q** were collected from a single crystal in 2.67 hours at 100(2) K on a Bruker D8 VENTURE KAPPA diffractometer with a microfocus sealed tube using a multilayer mirror as monochromator and a Bruker PHOTON III CPAD detector. The diffractometer was equipped with an Oxford Cryostream 1000 low temperature device and used Mo K_{α} radiation (λ = 0.71073 Å). All data were integrated with SAINT V8.41 yielding 49260 reflections of which 4036 where independent and 80.6% were greater than 2σ(F^2).[56] A Multi-Scan absorption correction using SADABS 2016/2 was applied.[57] The structure was solved by Intrinsic Phasing methods with SHELXT 2018/2 and refined by full-matrix least-squares methods against F^2 using SHELXL-2019/2.[58,59] All non-hydrogen atoms were refined with anisotropic displacement parameters. All hydrogen atoms were refined isotropic on calculated positions using a riding model with their U_{iso} values constrained to 1.5 times the U_{eq} of their pivot atoms for terminal sp^3 carbon atoms and 1.2 times for all other carbon atoms. Crystallographic data for the structures reported in this paper have been deposited with the Cambridge Crystallographic Data Centre.[60] CCDC 2516938 contain the supplementary crystallographic data for this paper. These data can be obtained free of charge from The Cambridge Crystallographic Data Centre *via* www.ccdc.cam.ac.uk/structures. This report and the CIF file were generated using FinalCif.[61]

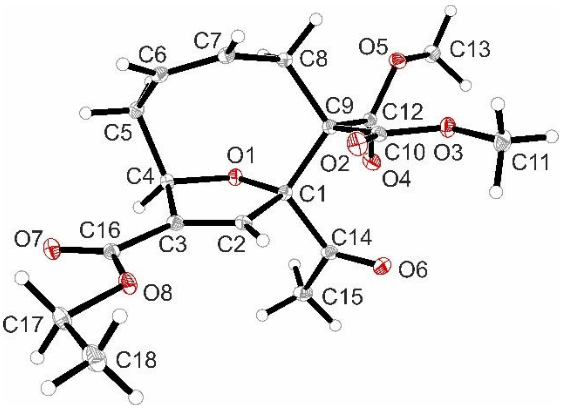

Figure S15: Crystal structure of compound **3q**. Thermal ellipsoids are shown at 30% probability.

Table S15. Crystal data and structure refinement for **3q**

CCDC number	2516938
Empirical formula	$C_{18}H_{22}O_8$
Formula weight	366.35
Temperature [K]	100(2)
Crystal system	orthorhombic
Space group (number)	*Pbcn* (60)
a [Å]	27.2432(8)
b [Å]	7.9233(2)
c [Å]	16.2660(4)
α [°]	90
β [°]	90
γ [°]	90
Volume [Å^3]	3511.11(16)
Z	8
ρ_{calc} [gcm^{-3}]	1.386
μ [mm^{-1}]	0.11
F(000)	1552
Crystal size [mm^3]	0.059×0.109×0.117
Crystal colour	colourless
Crystal shape	prism
Radiation	Mo K_{α} (λ=0.71073 Å)
2θ range [°]	5.01 to 54.99 (0.77 Å)

Index ranges	−35 ≤ h ≤ 33<br>−10 ≤ k ≤ 10<br>−21 ≤ l ≤ 19
Reflections collected	49260
Independent reflections	4036<br>Rint = 0.0601<br>Rsigma = 0.0282
Completeness to θ = 25.242°	99.9
Data / Restraints / Parameters	4036 / 0 / 239
Absorption correction Tmin/Tmax (method)	0.987 / 0.994 (Multi-Scan)
Goodness-of-fit on F^2	1.042
Final R indexes<br>[I≥2σ(I)]	R_1 = 0.0343<br>wR_2 = 0.0819
Final R indexes<br>[all data]	R_1 = 0.0466<br>wR_2 = 0.0905
Largest peak/hole [eÅ^{-3}]	0.32/−0.20

X-ray crystal structure analysis of 3z (glo10916): A colourless, prism-shaped crystal was mounted on a nylon loop. The crystals were crystallised from ethyl acetate and *n*-pentane. Data for **3z** were collected from a single crystal in 3.21 hours at 100(2) K on a Bruker D8 VENTURE KAPPA diffractometer with a microfocus sealed tube using a multilayer mirror as monochromator and a Bruker PHOTON III CPAD detector. The diffractometer used Mo K_α radiation (λ = 0.71073 Å). All data were integrated with SAINT V8.41 yielding 30940 reflections of which 3308 where independent and 92.4% were greater than 2σ(F^2).[56] A Multi-Scan absorption correction using SADABS 2016/2 was applied.[57] The structure was solved by Intrinsic Phasing methods with SHELXT 2018/2 and refined by full-matrix least-squares methods against F^2 using SHELXL-2019/2.[58,59] All non-hydrogen atoms were refined with anisotropic displacement parameters. All hydrogen atoms were refined isotropic on calculated positions using a riding model with their U_{iso} values constrained to 1.5 times the U_{eq} of their pivot atoms for terminal sp^3 carbon atoms and 1.2 times for all other carbon atoms. Crystallographic data for the structures reported in this paper have been deposited with the Cambridge Crystallographic Data Centre.[60] CCDC 2516939 contain the supplementary crystallographic data for this paper. These data can be obtained free of charge from The Cambridge Crystallographic Data Centre *via* www.ccdc.cam.ac.uk/structures. This report and the CIF file were generated using FinalCif.[61]

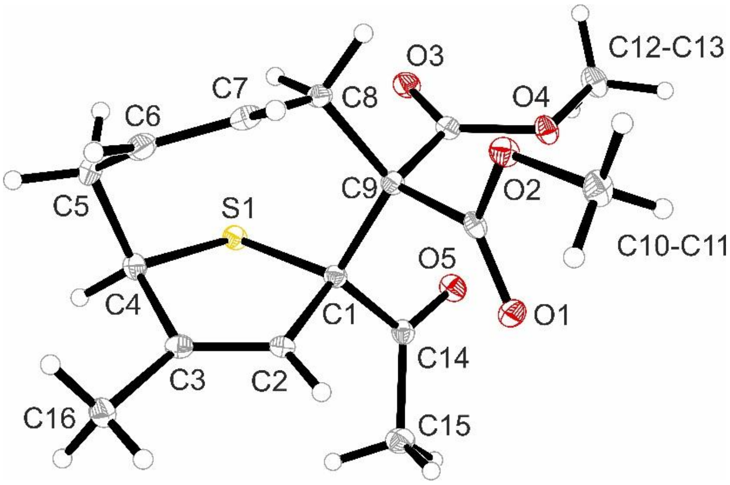

Figure S16: Crystal structure of compound **3z**. Thermal ellipsoids are shown at 30% probability.

Table S16. Crystal data and structure refinement for **3z**

CCDC number	2516939
Empirical formula	$C_{16}H_{20}O_5S$
Formula weight	324.38
Temperature [K]	100(2)
Crystal system	orthorhombic
Space group (number)	$Pna2_1$ (33)
a [Å]	15.7851(9)
b [Å]	8.5465(6)
c [Å]	11.5838(6)
α [°]	90
β [°]	90
γ [°]	90
Volume [$Å^3$]	1562.74(16)
Z	4
ρ_{calc} [gcm^{-3}]	1.379
μ [mm^{-1}]	0.228
F(000)	688
Crystal size [mm^3]	0.06×0.127×0.133
Crystal colour	colourless
Crystal shape	prism
Radiation	Mo K_α (λ=0.71073 Å)

2θ range [°]	5.16 to 53.46 (0.79 Å)
Index ranges	$-19 \le h \le 19$<br>$-10 \le k \le 10$<br>$-14 \le l \le 14$
Reflections collected	30940
Independent reflections	3308<br>Rint = 0.0789<br>Rsigma = 0.0402
Completeness to θ = 25.242°	99.9
Data / Restraints / Parameters	3308 / 1 / 203
Absorption correction Tmin/Tmax (method)	0.970 / 0.986 (Multi-Scan)
Goodness-of-fit on F^2	1.052
Final R indexes [I≥2σ(I)]	R_1 = 0.0297<br>wR_2 = 0.0682
Final R indexes [all data]	R_1 = 0.0340<br>wR_2 = 0.0711
Largest peak/hole [eÅ^{-3}]	0.18/−0.19

X-ray crystal structure analysis of 3ag (glo10965): A colourless, prism-shaped crystal was mounted on a MiTeGen micromount. The crystals were crystallised from ethyl acetate and *n*-pentane. Data for **3ag** were collected from a single crystal in 16.48 hours at 100(2) K on a Bruker D8 VENTURE KAPPA diffractometer with a microfocus sealed tube using a multilayer mirror as monochromator and a Bruker PHOTON III CPAD detector. The diffractometer was equipped with an Oxford Cryostream 1000 low temperature device and used Mo K_α radiation (λ = 0.71073 Å). All data were integrated with SAINT V8.41 yielding 62070 reflections of which 4250 where independent and 83.7% were greater than 2σ(F^2).[56] A Multi-Scan absorption correction using SADABS 2016/2 was applied.[57] The structure was solved by Intrinsic Phasing methods with SHELXT 2018/2 and refined by full-matrix least-squares methods against F^2 using SHELXL-2019/2.[58,59] All non-hydrogen atoms were refined with anisotropic displacement parameters. All hydrogen atoms were refined isotropic on calculated positions using a riding model with their U_{iso} values constrained to 1.5 times the U_{eq} of their pivot atoms for terminal sp^3 carbon atoms and 1.2 times for all other carbon atoms. Crystallographic data for the structures reported in this paper have been deposited with the Cambridge Crystallographic Data Centre.[60] CCDC 2516934 contain the supplementary crystallographic data for this paper. These data can be obtained free of charge from The Cambridge Crystallographic Data Centre *via* www.ccdc.cam.ac.uk/structures. This report and the CIF file were generated using FinalCif.[61]

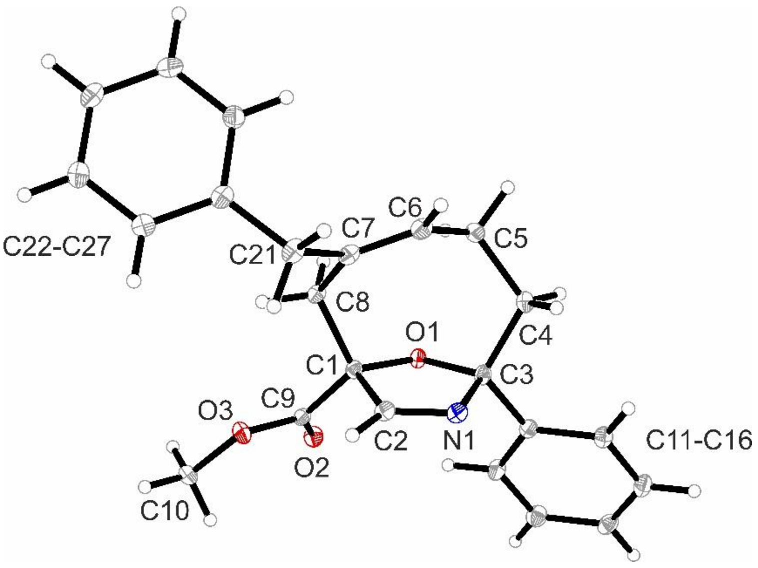

Figure S17: Crystal structure of compound **3ag**. Thermal ellipsoids are shown at 30% probability.

Table S17. Crystal data and structure refinement for **3ag**

CCDC number	2516934
Empirical formula	$C_{23}H_{23}NO_3$
Formula weight	361.42
Temperature [K]	100(2)
Crystal system	monoclinic
Space group (number)	$P2_1/c$ (14)
a [Å]	17.8896(9)
b [Å]	8.6260(5)
c [Å]	12.8106(6)
α [°]	90
β [°]	110.211(2)
γ [°]	90
Volume [Å^3]	1855.15(17)
Z	4
ρ_{calc} [gcm^{-3}]	1.294
μ [mm^{-1}]	0.085
F(000)	768
Crystal size [mm^3]	0.039×0.068×0.096
Crystal colour	colourless

Crystal shape	prism
Radiation	Mo K_α (λ=0.71073 Å)
2θ range [°]	4.85 to 55.01 (0.77 Å)
Index ranges	−23 ≤ h ≤ 23<br>−11 ≤ k ≤ 10<br>−16 ≤ l ≤ 16
Reflections collected	62070
Independent reflections	4250<br>Rint = 0.0477<br>Rsigma = 0.0187
Completeness to θ = 25.242°	99.9
Data / Restraints / Parameters	4250 / 0 / 245
Absorption correction Tmin/Tmax (method)	0.992 / 0.997 (Multi-Scan)
Goodness-of-fit on F^2	1.027
Final R indexes<br>[I≥2σ(I)]	R_1 = 0.0349<br>wR_2 = 0.0850
Final R indexes<br>[all data]	R_1 = 0.0442<br>wR_2 = 0.0914
Largest peak/hole [eÅ^{-3}]	0.32/−0.19

X-ray crystal structure analysis of 4c (glo10929): A colourless, prism-shaped crystal was mounted on a MiTeGen micromount. The crystals were crystallised from ethyl acetate and *n*-pentane. Data for **4c** were collected from a single crystal in 2.53 hours at 100(2) K on a Bruker D8 VENTURE KAPPA diffractometer with a microfocus sealed tube using a multilayer mirror as monochromator and a Bruker PHOTON III CPAD detector. The diffractometer was equipped with an Oxford Cryostream 1000 low temperature device and used Mo K_α radiation (λ = 0.71073 Å). All data were integrated with SAINT V8.41 yielding 39470 reflections of which 2915 where independent and 91.9% were greater than 2σ(F^2).[56] A Multi-Scan absorption correction using SADABS 2016/2 was applied.[57] The structure was solved by Intrinsic Phasing methods with SHELXT 2018/2 and refined by full-matrix least-squares methods against F^2 using SHELXL-2019/2.[58,59] All non-hydrogen atoms were refined with anisotropic displacement parameters. All hydrogen atoms were refined with isotropic displacement parameters. Some of their coordinates were refined freely and some on calculated positions using a riding model with their U_{iso} values constrained to 1.5 times the U_{eq} of their pivot atoms for terminal sp^3 carbon atoms and 1.2 times for all other carbon atoms. Crystallographic data for the structures reported in this paper have been deposited with the Cambridge Crystallographic Data Centre.[60] CCDC 2516940 contain the supplementary crystallographic data for this paper. These data can be obtained free of charge from The Cambridge Crystallographic Data Centre *via* www.ccdc.cam.ac.uk/structures. This report and the CIF file were generated using FinalCif.[61]

*Special refinement details for **4c**:* The hydrogen at O6 atom was refined freely.

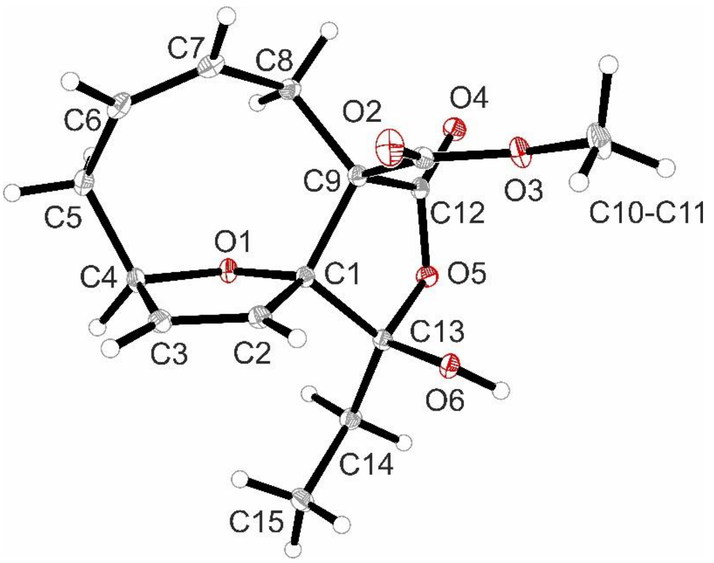

Figure S18: Crystal structure of compound **4c**. Thermal ellipsoids are shown at 30% probability.

Table S18. Crystal data and structure refinement for **4c**

CCDC number	2516938
Empirical formula	$C_{15}H_{18}O_6$
Formula weight	294.29
Temperature [K]	100(2)
Crystal system	monoclinic
Space group (number)	$P2_1/c$ (14)
a [Å]	8.2889(2)
b [Å]	9.5530(2)
c [Å]	17.4600(5)
α [°]	90
β [°]	94.6410(10)
γ [°]	90
Volume [$Å^3$]	1378.02(6)
Z	4
ρ_{calc} [gcm^{-3}]	1.419
μ [mm^{-1}]	0.11
F(000)	624
Crystal size [mm^3]	0.106×0.123×0.156
Crystal colour	colourless

Crystal shape	prism
Radiation	Mo K_α (λ=0.71073 Å)
2θ range [°]	4.68 to 53.47 (0.79 Å)
Index ranges	−10 ≤ h ≤ 10<br>−12 ≤ k ≤ 12<br>−22 ≤ l ≤ 22
Reflections collected	39470
Independent reflections	2915<br>Rint = 0.0378<br>Rsigma = 0.0173
Completeness to θ = 25.242°	99.8
Data / Restraints / Parameters	2915 / 0 / 196
Absorption correction Tmin/Tmax (method)	0.983 / 0.988 (Multi-Scan)
Goodness-of-fit on F^2	1.023
Final R indexes<br>[I≥2σ(I)]	R_1 = 0.0351<br>wR_2 = 0.0948
Final R indexes<br>[all data]	R_1 = 0.0379<br>wR_2 = 0.0980
Largest peak/hole [eÅ^{-3}]	0.40/−0.28

X-ray crystal structure analysis of 4e (glo10973): A colourless, prism-shaped crystal was mounted on a loop. The crystals were crystallised from ethyl acetate and *n*-pentane. Data for **4e** were collected from a single crystal in 4.61 hours at 100(2) K on a Bruker D8 VENTURE KAPPA diffractometer with a microfocus sealed tube using a multilayer mirror as monochromator and a Bruker PHOTON III CPAD detector. The diffractometer was equipped with an Oxford Cryostream 1000 low temperature device and used Mo K_α radiation (λ = 0.71073 Å). All data were integrated with SAINT V8.41 yielding 49843 reflections of which 4382 where independent and 85.2% were greater than 2σ(F^2).[56] A Multi-Scan absorption correction using SADABS 2016/2 was applied.[57] The structure was solved by Intrinsic Phasing methods with SHELXT 2018/2 and refined by full-matrix least-squares methods against F^2 using SHELXL-2019/2.[58,59] All non-hydrogen atoms were refined with anisotropic displacement parameters. All hydrogen atoms were refined isotropic on calculated positions using a riding model with their U_{iso} values constrained to 1.5 times the U_{eq} of their pivot atoms for terminal sp^3 carbon atoms and 1.2 times for all other carbon atoms. Crystallographic data for the structures reported in this paper have been deposited with the Cambridge Crystallographic Data Centre.[60] CCDC 2516941 contain the supplementary crystallographic data for this paper. These data can be obtained free of charge from The Cambridge Crystallographic Data Centre *via* www.ccdc.cam.ac.uk/structures. This report and the CIF file were generated using FinalCif.[61]

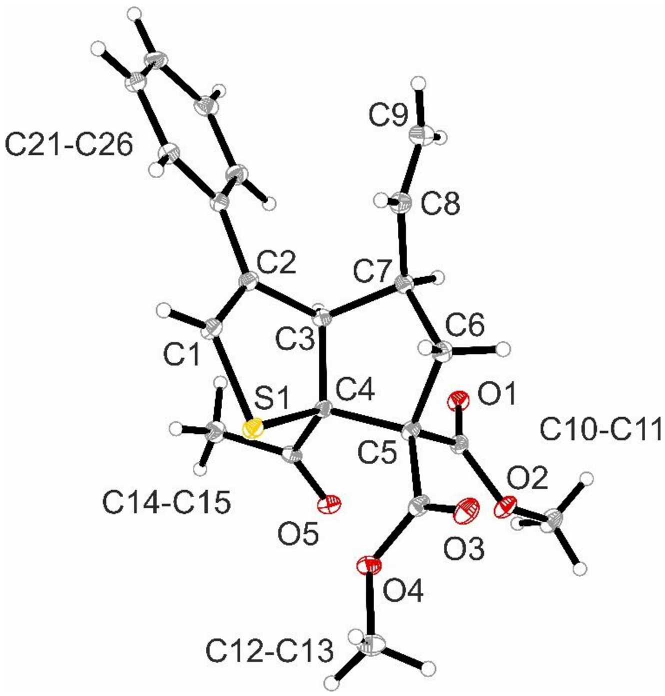

Figure S19: Crystal structure of compound **4e**. Thermal ellipsoids are shown at 30% probability.

Table S19. Crystal data and structure refinement for **4e**

CCDC number	2516941
Empirical formula	$C_{21}H_{22}O_5S$
Formula weight	386.44
Temperature [K]	100(2)
Crystal system	monoclinic
Space group (number)	$P2_1/n$ (14)
a [Å]	13.6840(3)
b [Å]	7.4325(2)
c [Å]	18.9482(6)
α [°]	90
β [°]	98.5440(10)
γ [°]	90
Volume [Å^3]	1905.76(9)
Z	4
ρ_{calc} [gcm^{-3}]	1.347
μ [mm^{-1}]	0.199
F(000)	816

Crystal size [mm^3]	0.043×0.078×0.095
Crystal colour	colourless
Crystal shape	prism
Radiation	Mo K_α (λ=0.71073 Å)
2θ range [°]	3.97 to 55.03 (0.77 Å)
Index ranges	−17 ≤ h ≤ 17<br>−9 ≤ k ≤ 9<br>−24 ≤ l ≤ 24
Reflections collected	49843
Independent reflections	4382<br>Rint = 0.0583<br>Rsigma = 0.0259
Completeness to θ = 25.242°	99.9
Data / Restraints / Parameters	4382 / 0 / 247
Absorption correction Tmin/Tmax (method)	0.981 / 0.991 (Multi-Scan)
Goodness-of-fit on F^2	1.044
Final R indexes<br>[I≥2σ(I)]	R_1 = 0.0328<br>wR_2 = 0.0800
Final R indexes<br>[all data]	R_1 = 0.0414<br>wR_2 = 0.0856
Largest peak/hole [eÅ^{-3}]	0.35/−0.25

X-ray crystal structure analysis of 4k (glo10974): A colourless, needle-shaped crystal was mounted on a loop. The crystals were crystallised from CH_2Cl_2 and *n*-pentane. Data for **4k** were collected from a single crystal in 6.93 hours at 100(2) K on a Bruker D8 VENTURE KAPPA diffractometer with a microfocus sealed tube using a multilayer mirror as monochromator and a Bruker PHOTON III CPAD detector. The diffractometer was equipped with an Oxford Cryostream 1000 low temperature device and used Mo K_α radiation (λ = 0.71073 Å). All data were integrated with SAINT V8.41 yielding 47926 reflections of which 3974 where independent and 86.6% were greater than 2σ(F^2).[56] A Multi-Scan absorption correction using SADABS 2016/2 was applied.[57] The structure was solved by Intrinsic Phasing methods with SHELXT 2018/2 and refined by full-matrix least-squares methods against F^2 using SHELXL-2019/2.[58,59] All non-hydrogen atoms were refined with anisotropic displacement parameters. All hydrogen atoms were refined with isotropic displacement parameters. Some of their coordinates were refined freely and some on calculated positions using a riding model with their U_{iso} values constrained to 1.5 times the U_{eq} of their pivot atoms for terminal sp^3 carbon atoms and 1.2 times for all other carbon atoms. Crystallographic data for the structures reported in this paper have been deposited with the Cambridge Crystallographic Data Centre.[60] CCDC 2516942 contain the supplementary crystallographic data for this paper. These data can be obtained free of charge from The Cambridge Crystallographic Data Centre *via* www.ccdc.cam.ac.uk/structures. This report and the CIF file were generated using FinalCif.[61]

__Special refinement details for 4k:__ The hydrogen at O2 atom was refined freely.

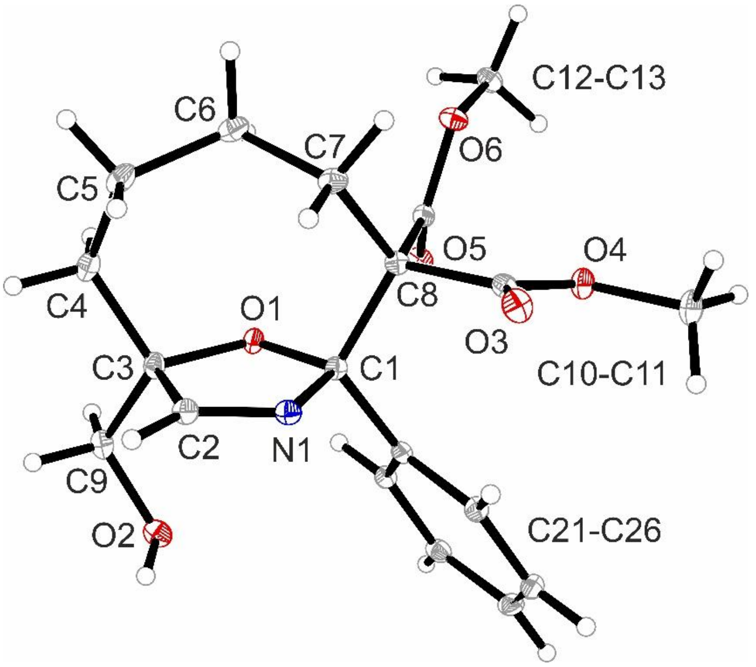

Figure S20: Crystal structure of compound **4k**. Thermal ellipsoids are shown at 30% probability.

Table S20. Crystal data and structure refinement for **4k**

CCDC number	2516942
Empirical formula	$C_{19}H_{23}NO_6$
Formula weight	361.38
Temperature [K]	100(2)
Crystal system	monoclinic
Space group (number)	$P2_1/c$ (14)
a [Å]	13.0243(5)
b [Å]	11.0366(4)
c [Å]	13.5155(5)
α [°]	90
β [°]	116.7490(10)
γ [°]	90
Volume [Å^3]	1734.87(11)
Z	4
ρ_{calc} [gcm^{-3}]	1.384
μ [mm^{-1}]	0.103

$F(000)$	768
Crystal size [mm^3]	0.035×0.079×0.109
Crystal colour	colourless
Crystal shape	needle
Radiation	Mo K_{α} (λ=0.71073 Å)
2θ range [°]	5.00 to 54.99 (0.77 Å)
Index ranges	−16 ≤ h ≤ 16<br>−14 ≤ k ≤ 14<br>−17 ≤ l ≤ 17
Reflections collected	47926
Independent reflections	3974<br>Rint = 0.05<br>Rsigma = 0.0214
Completeness to θ = 25.242°	99.8
Data / Restraints / Parameters	3974 / 0 / 241
Absorption correction Tmin/Tmax (method)	0.989 / 0.996 (Multi-Scan)
Goodness-of-fit on F^2	1.031
Final R indexes<br>[I≥2σ(I)]	R_1 = 0.0344<br>wR_2 = 0.0873
Final R indexes<br>[all data]	R_1 = 0.0410<br>wR_2 = 0.0927
Largest peak/hole [eÅ^{-3}]	0.35/−0.27

6. SPECTROSCOPIC DATA

^{1}H NMR (400 MHz, $CDCl_3$) of **1a**

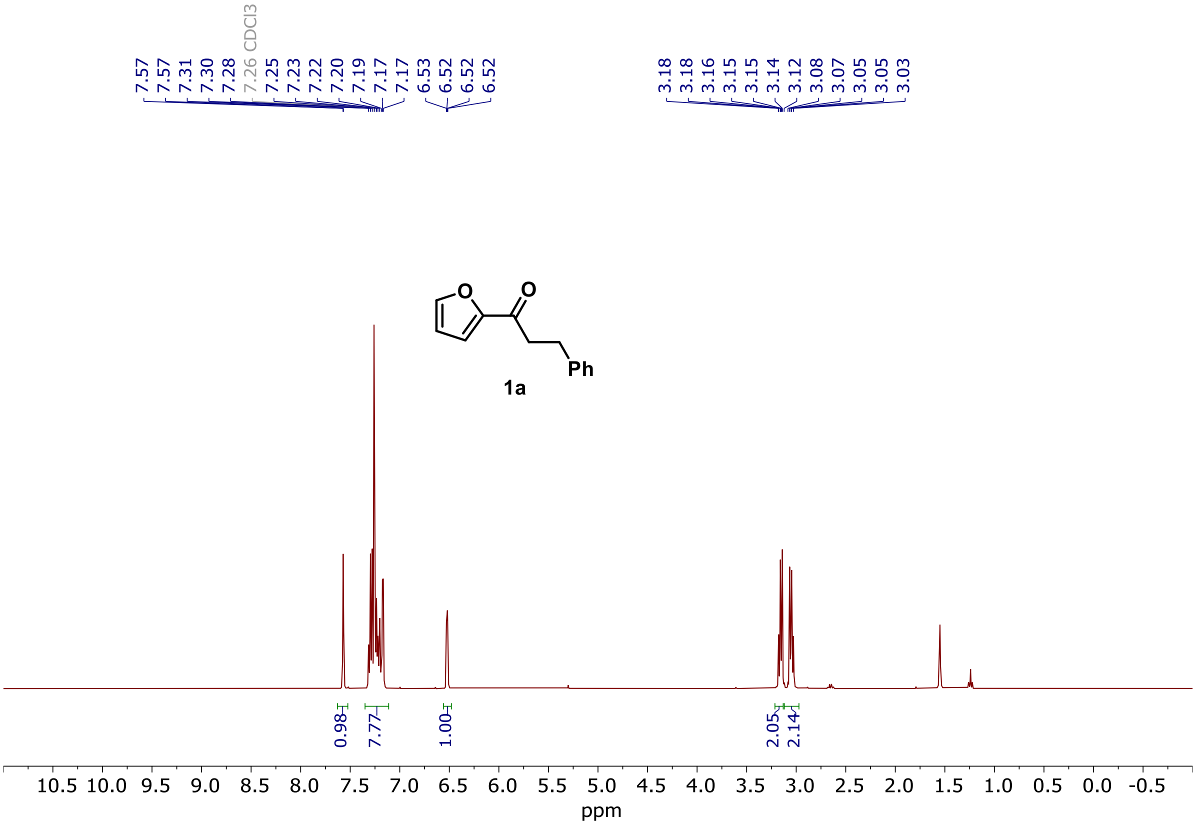

^{1}H NMR (400 MHz, $CDCl_3$) of **1d**

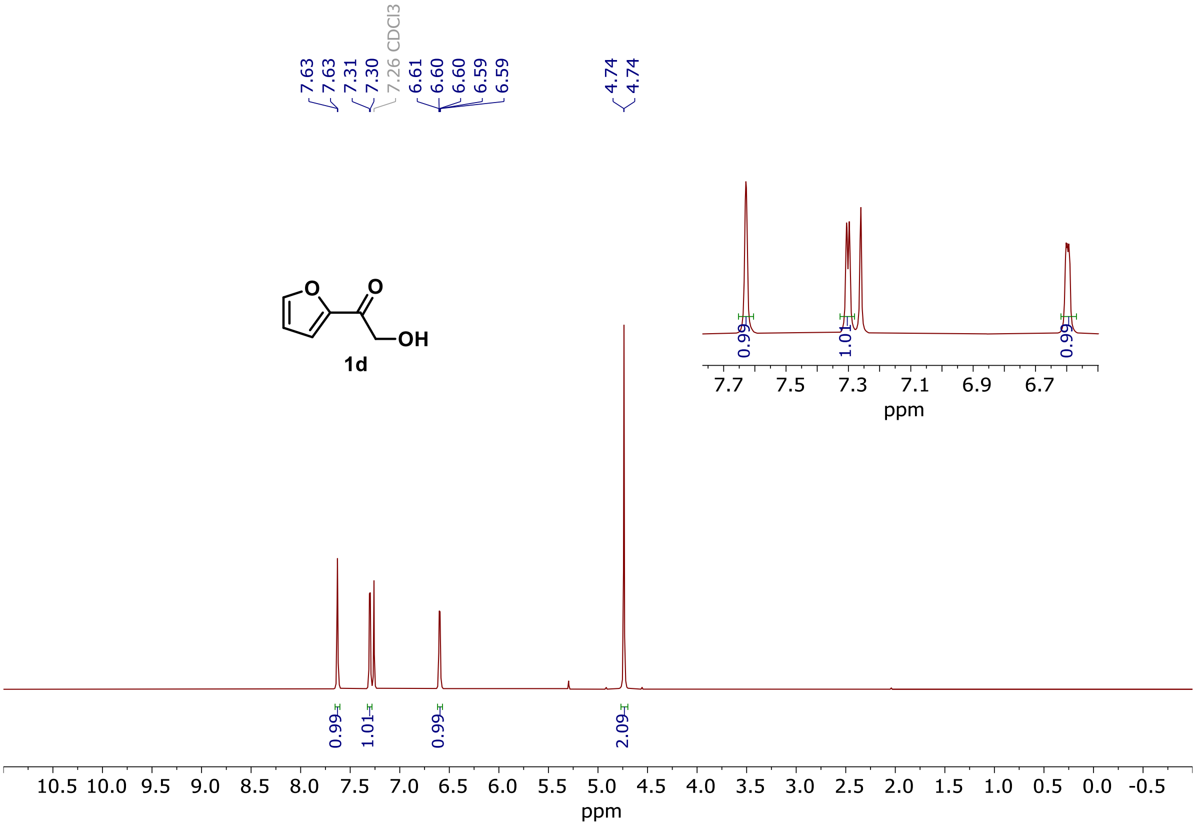

^{1}H NMR (400 MHz, $CDCl_3$) of **1e**

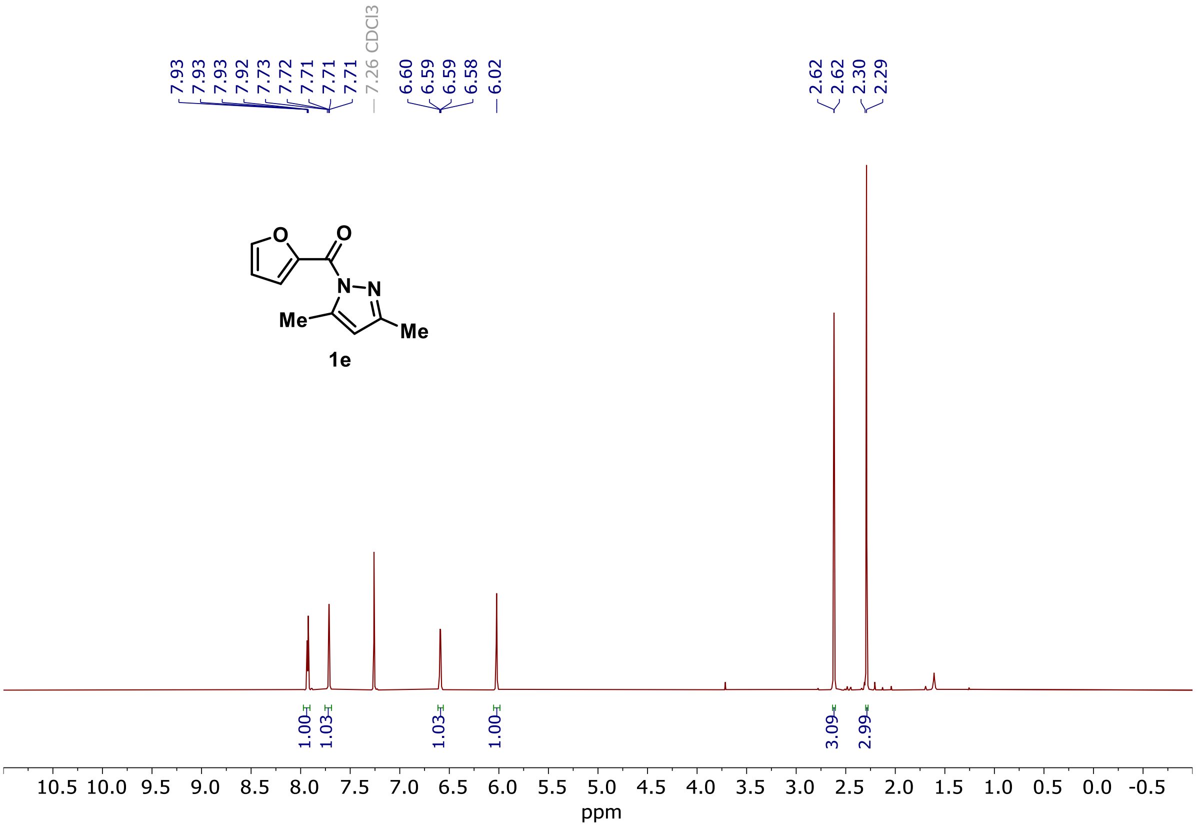

^{13}C NMR (101 MHz, $CDCl_3$) of **1e**

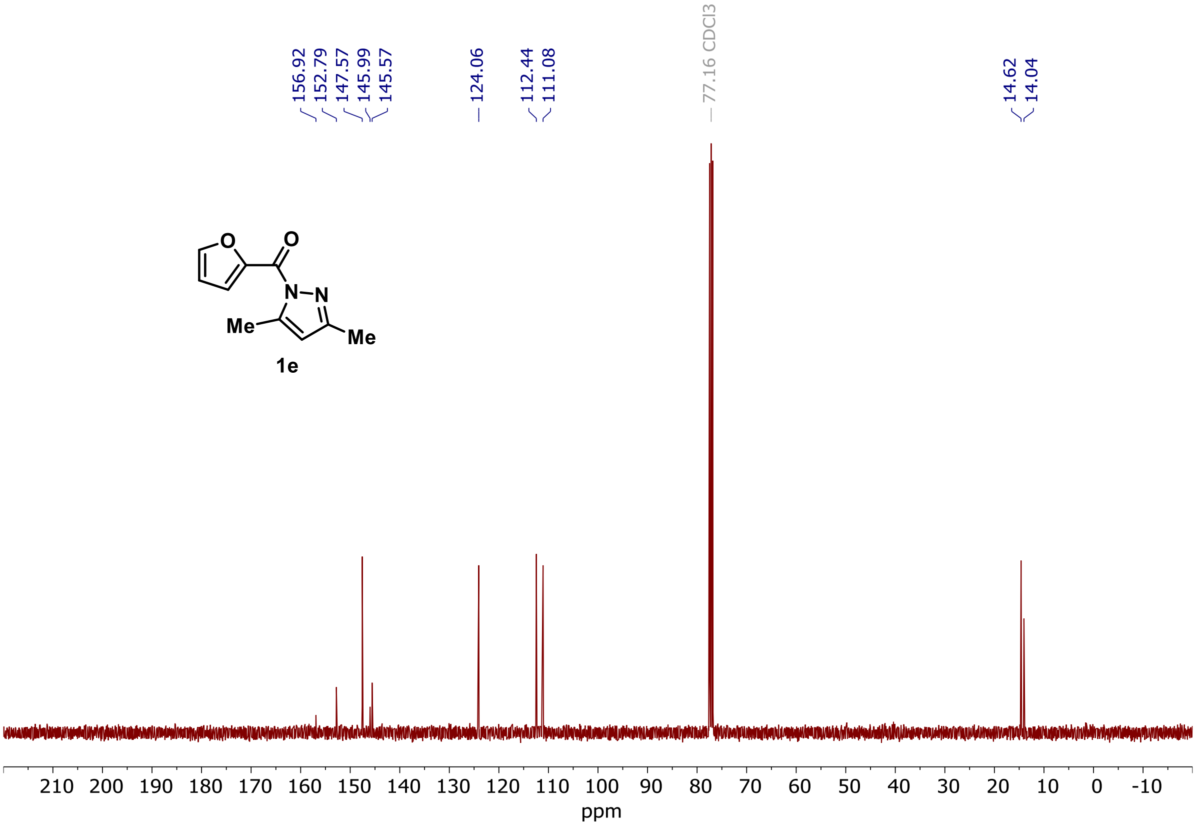

^{1}H NMR (400 MHz, $CDCl_3$) of **1f**

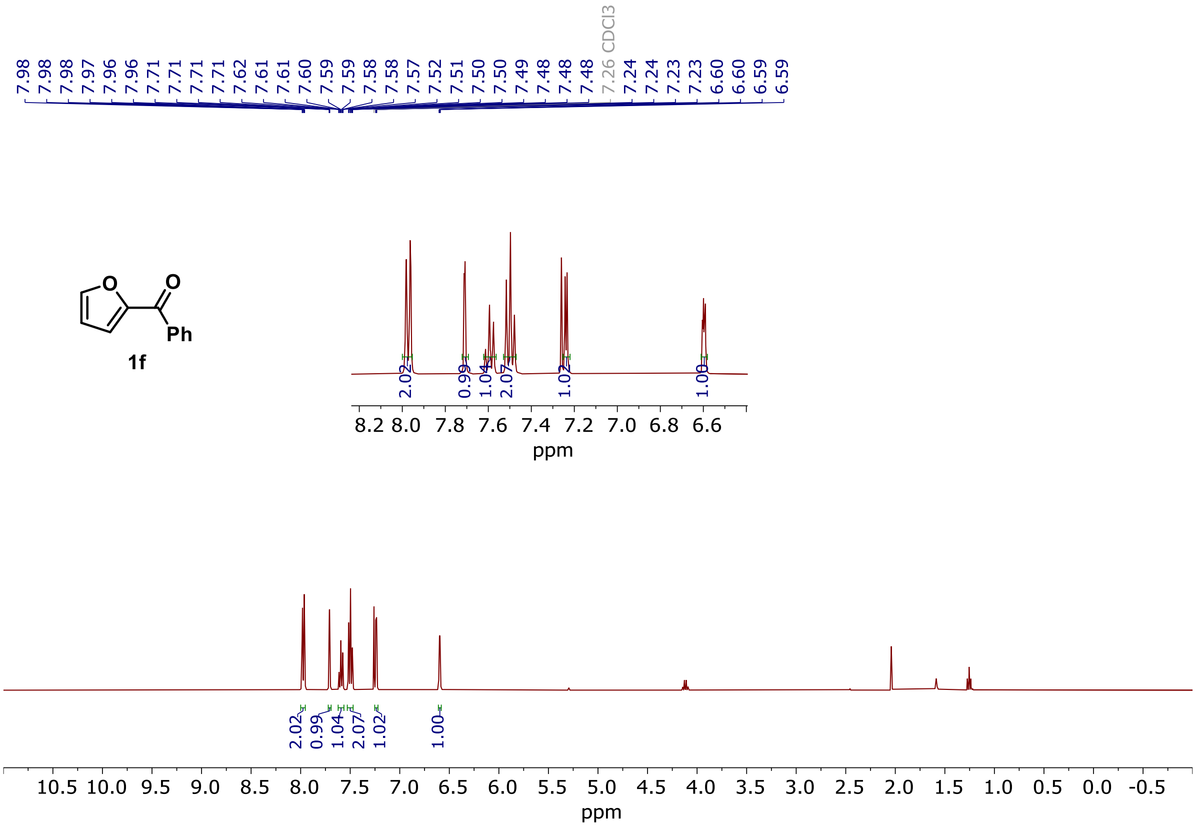

^{1}H NMR (400 MHz, $CDCl_3$) of **1h**

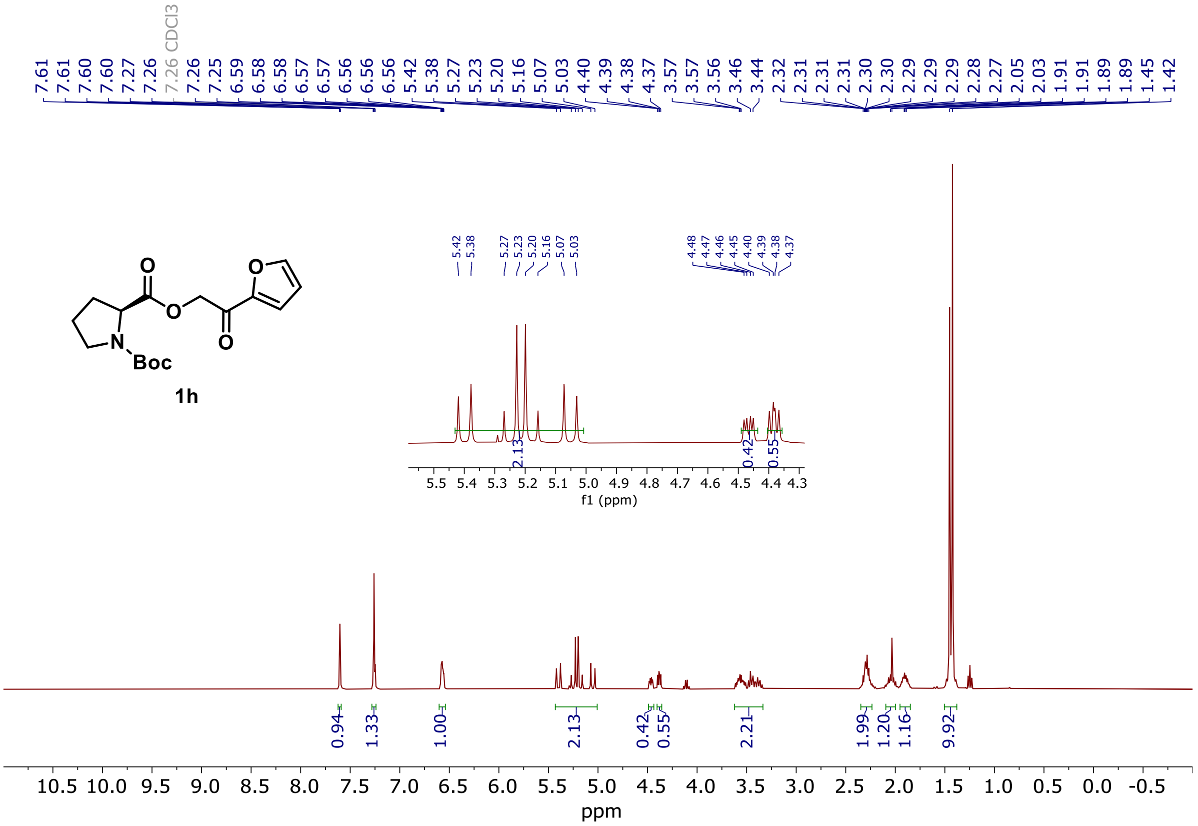

^{13}C NMR (101 MHz, $CDCl_3$) of **1h**

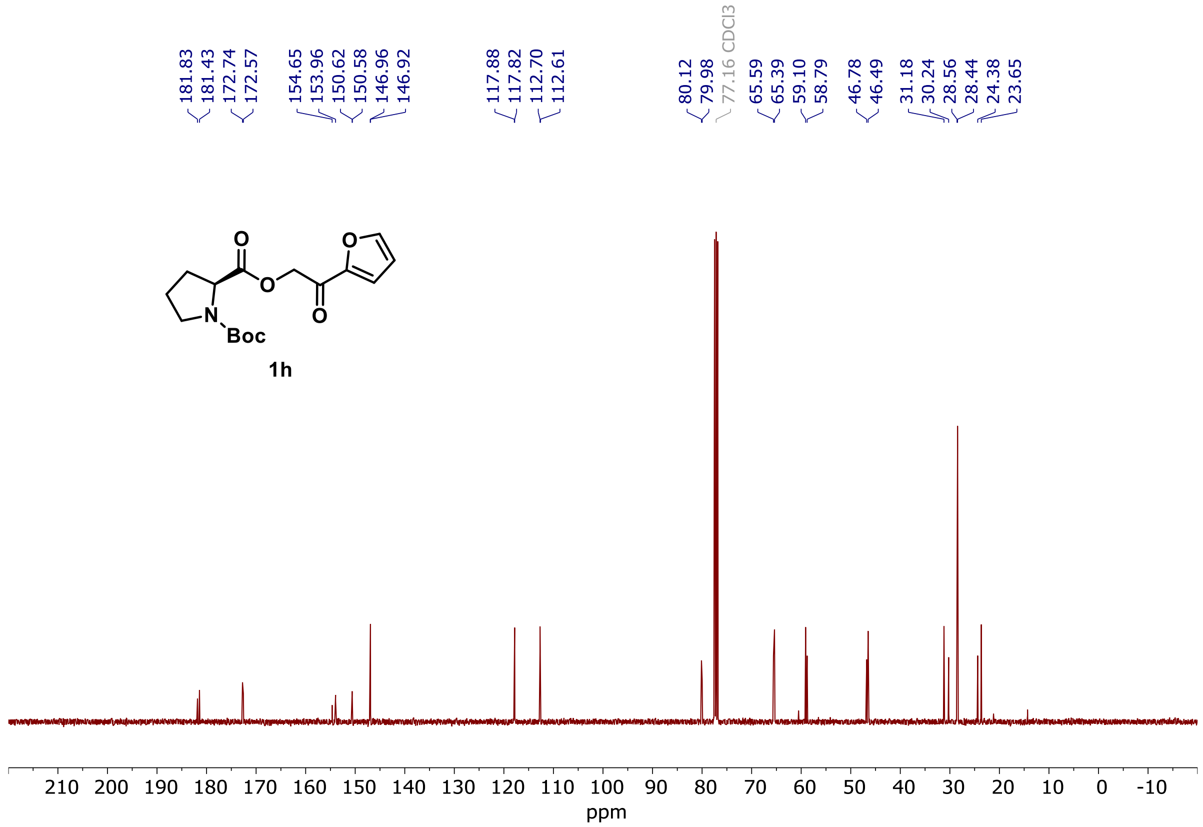

^{1}H NMR (400 MHz, $CDCl_3$) of **1k**

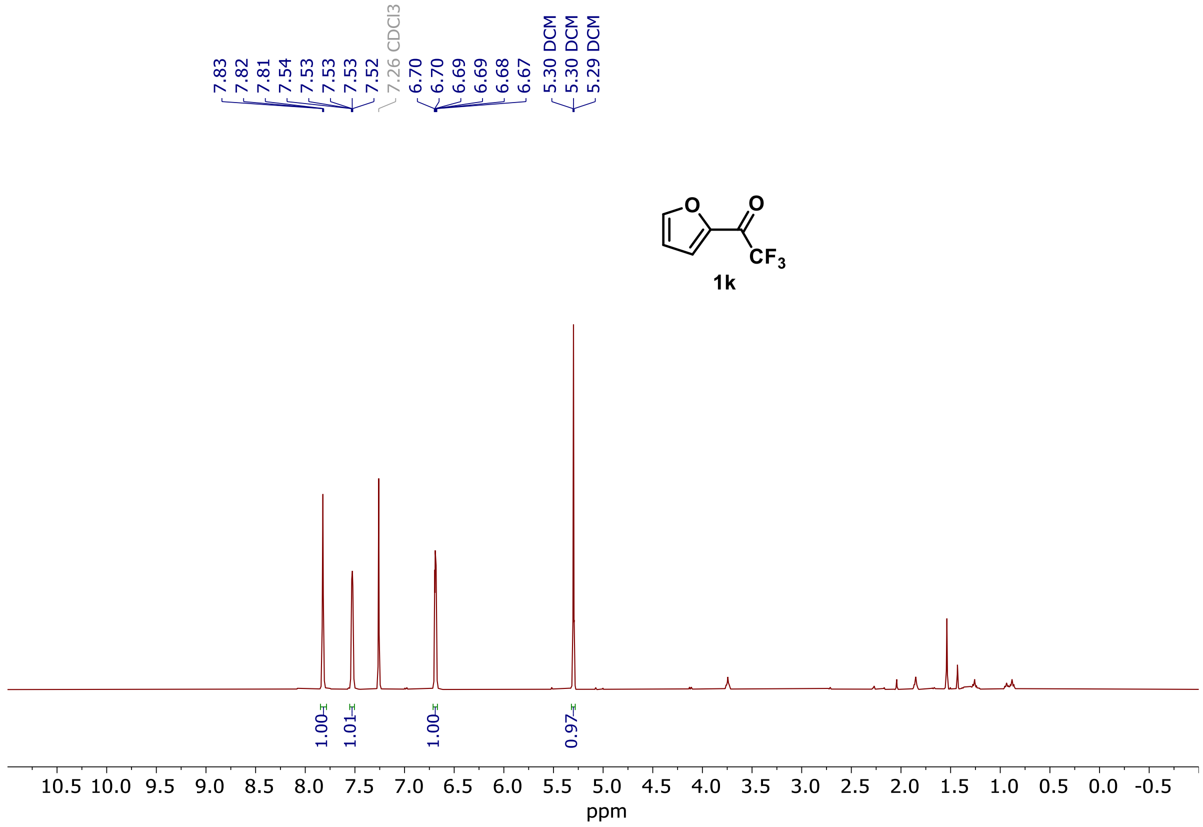

^1H NMR (400 MHz, $CDCl_3$) of **1l**

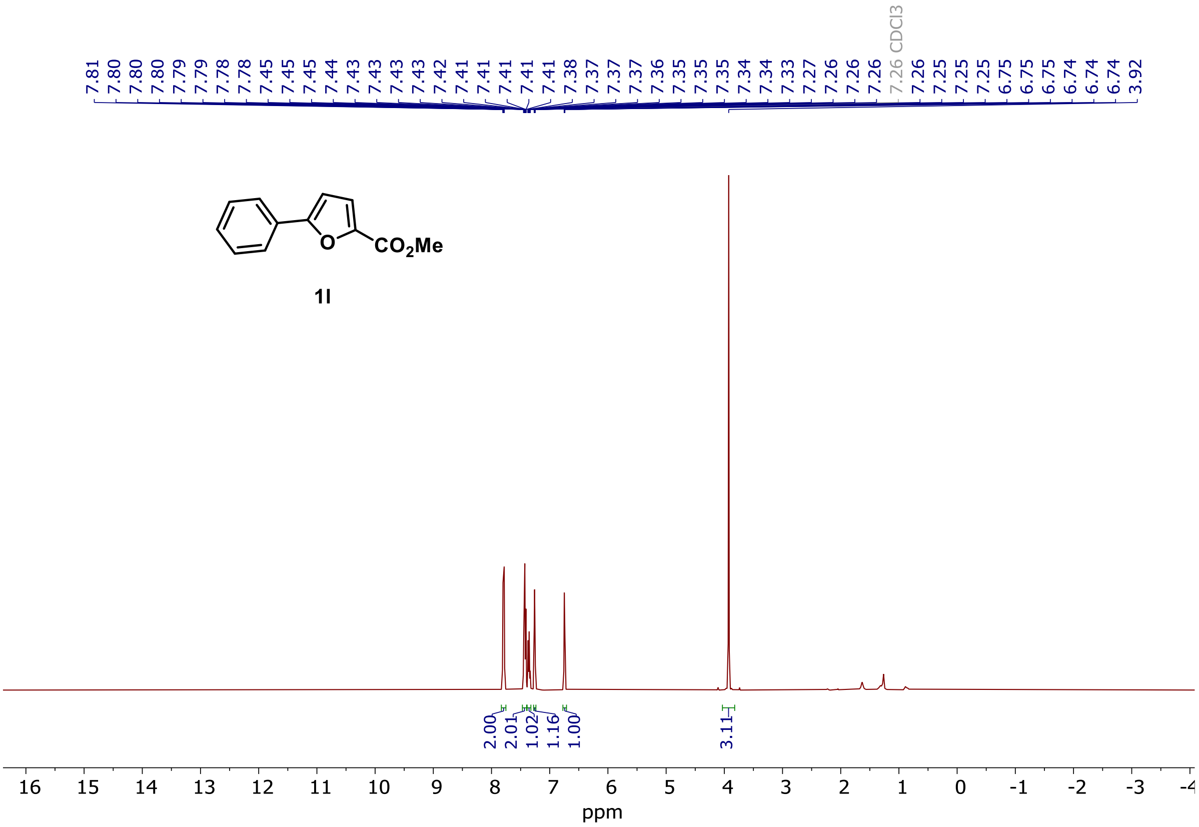

^1H NMR (400 MHz, $CDCl_3$) of **1m**

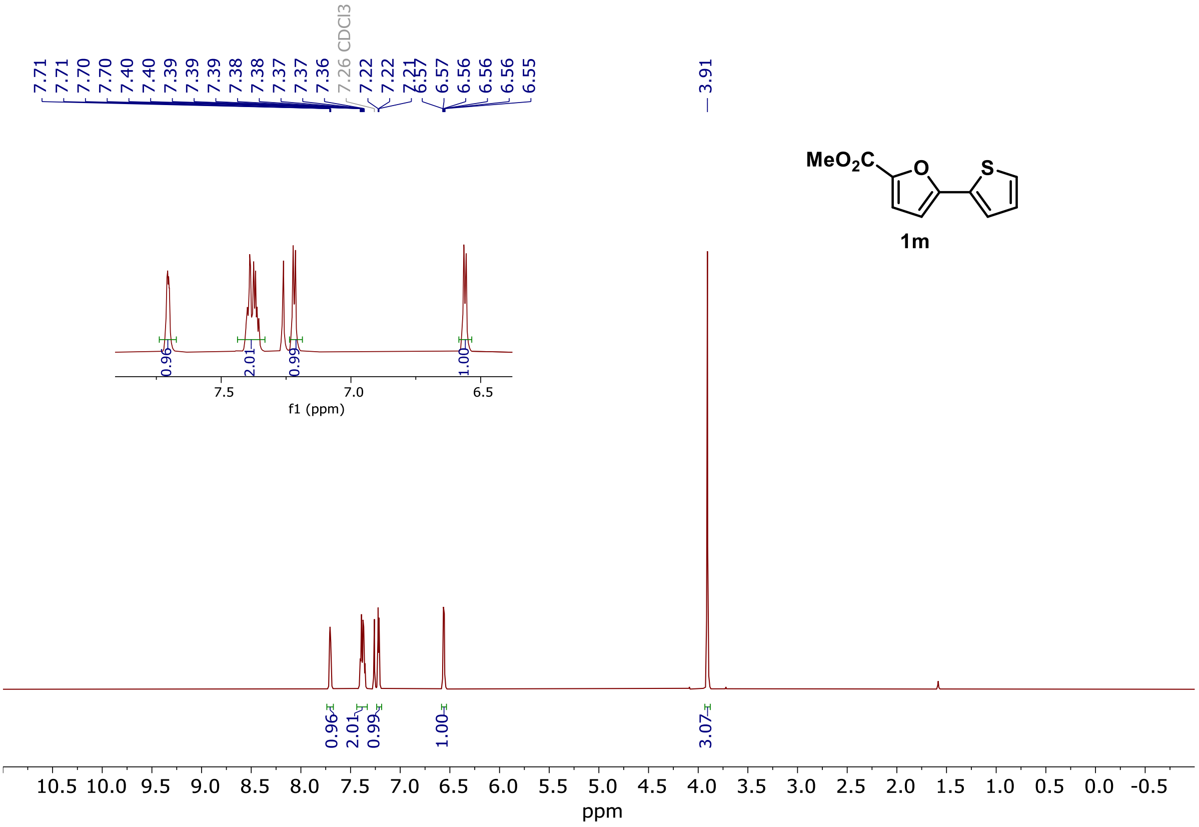

^{1}H NMR (400 MHz, $CDCl_3$) of **1n**

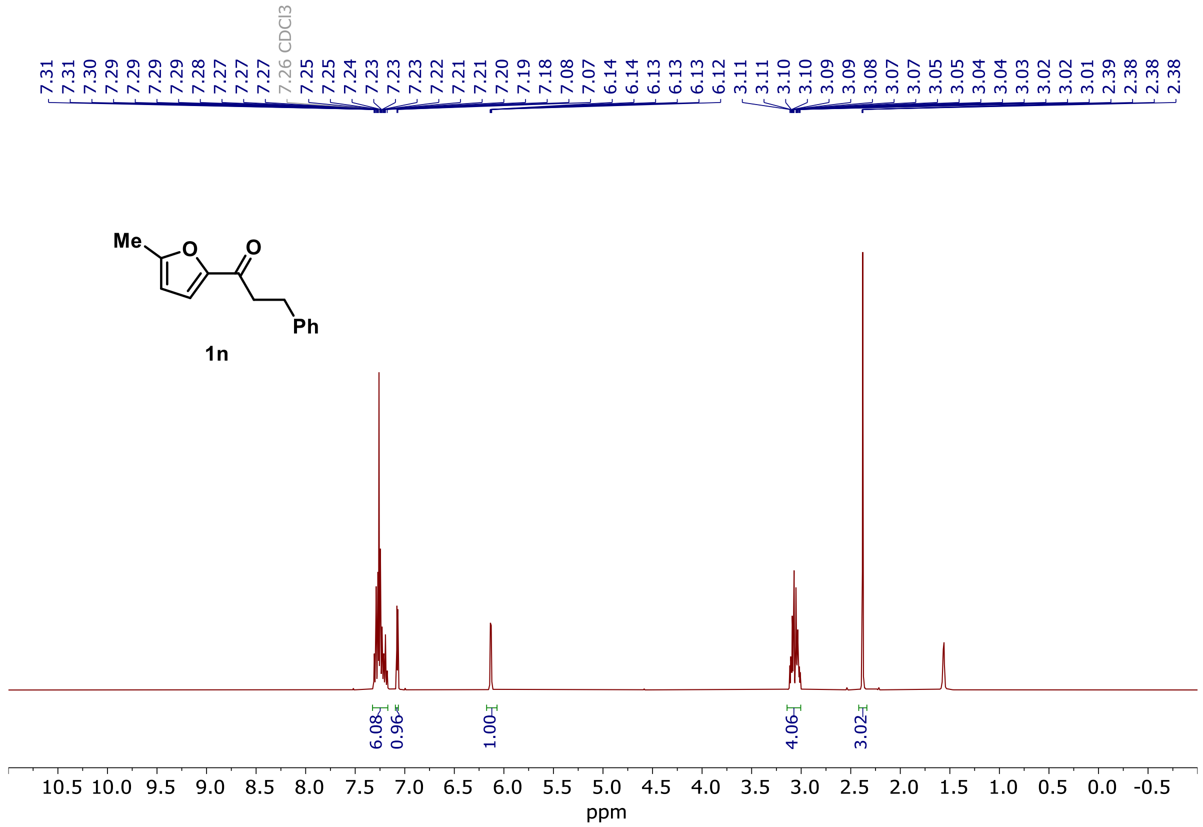

^{1}H NMR (400 MHz, $CDCl_3$) of **1o**

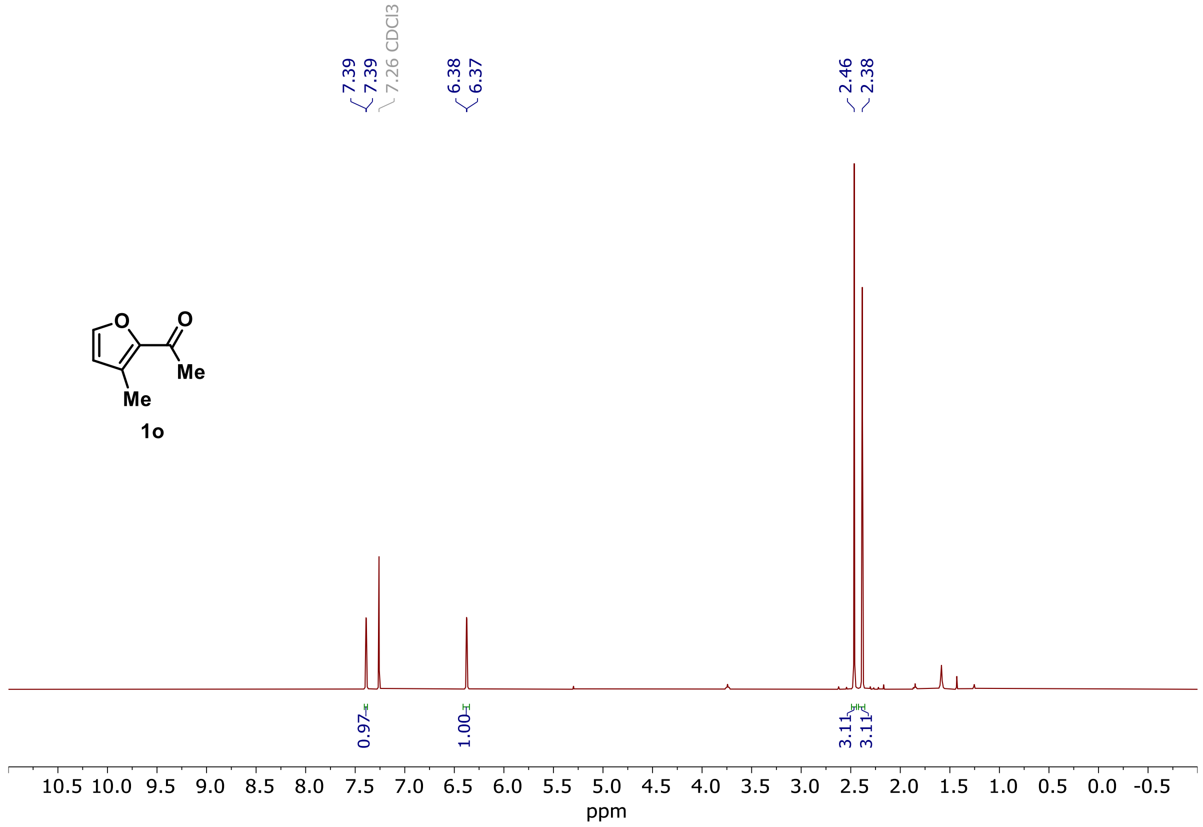

1H NMR (400 MHz, $CDCl_3$) of **1p**

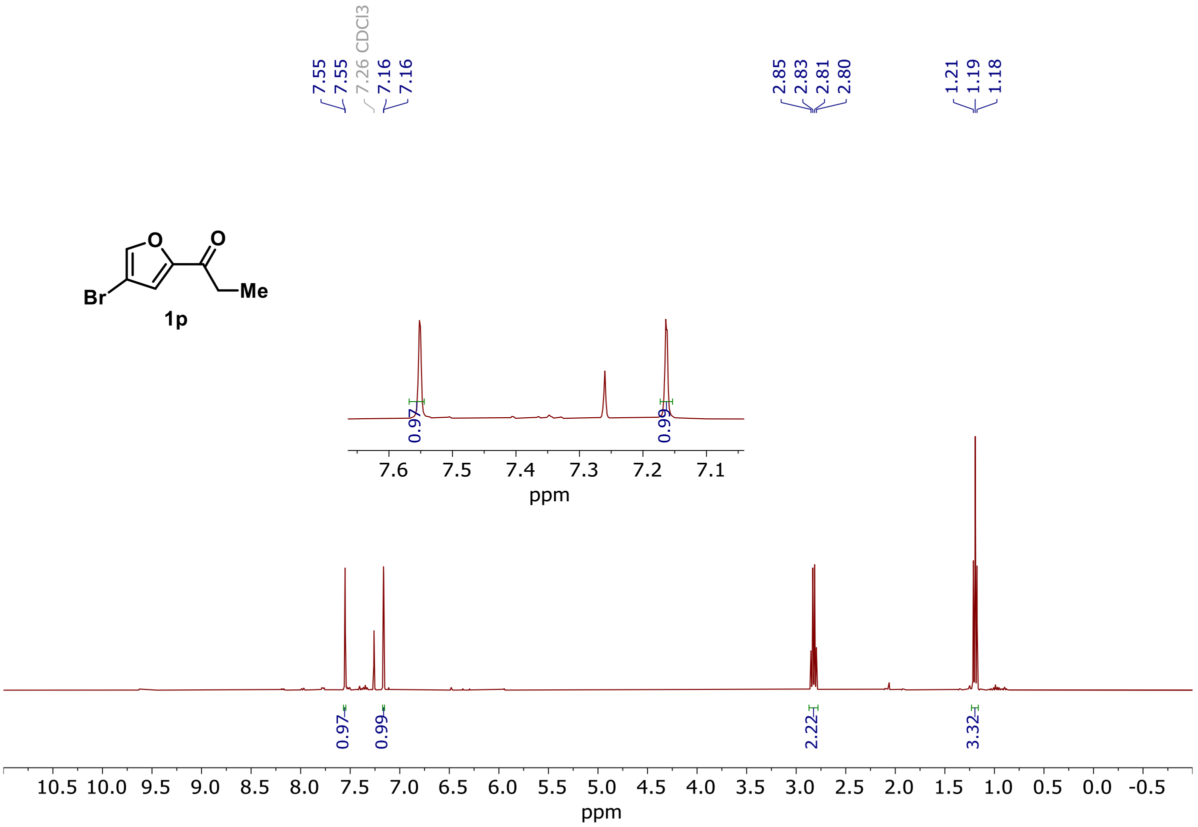

^{1}H NMR (400 MHz, $CDCl_3$) of **1q**

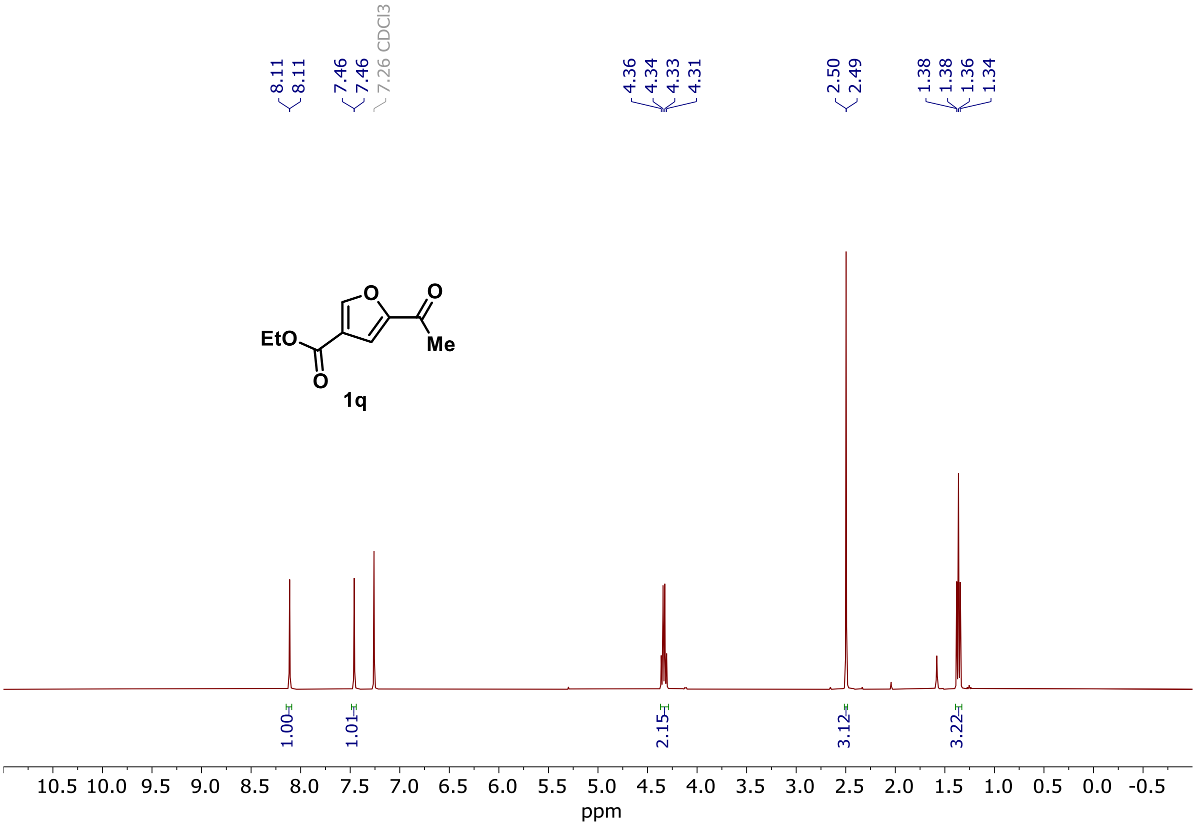

^{1}H NMR (400 MHz, $CDCl_3$) of **1s**

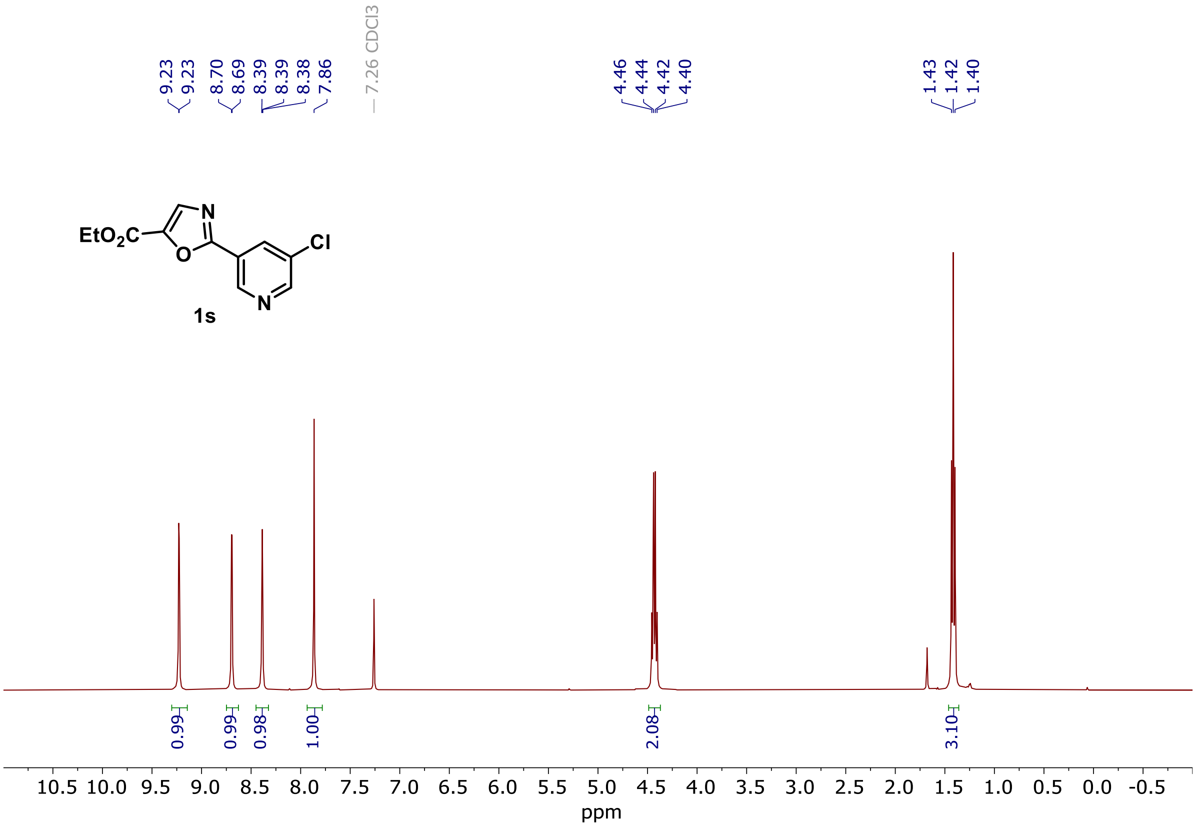

^{13}C NMR (101 MHz, $CDCl_3$) of **1s**

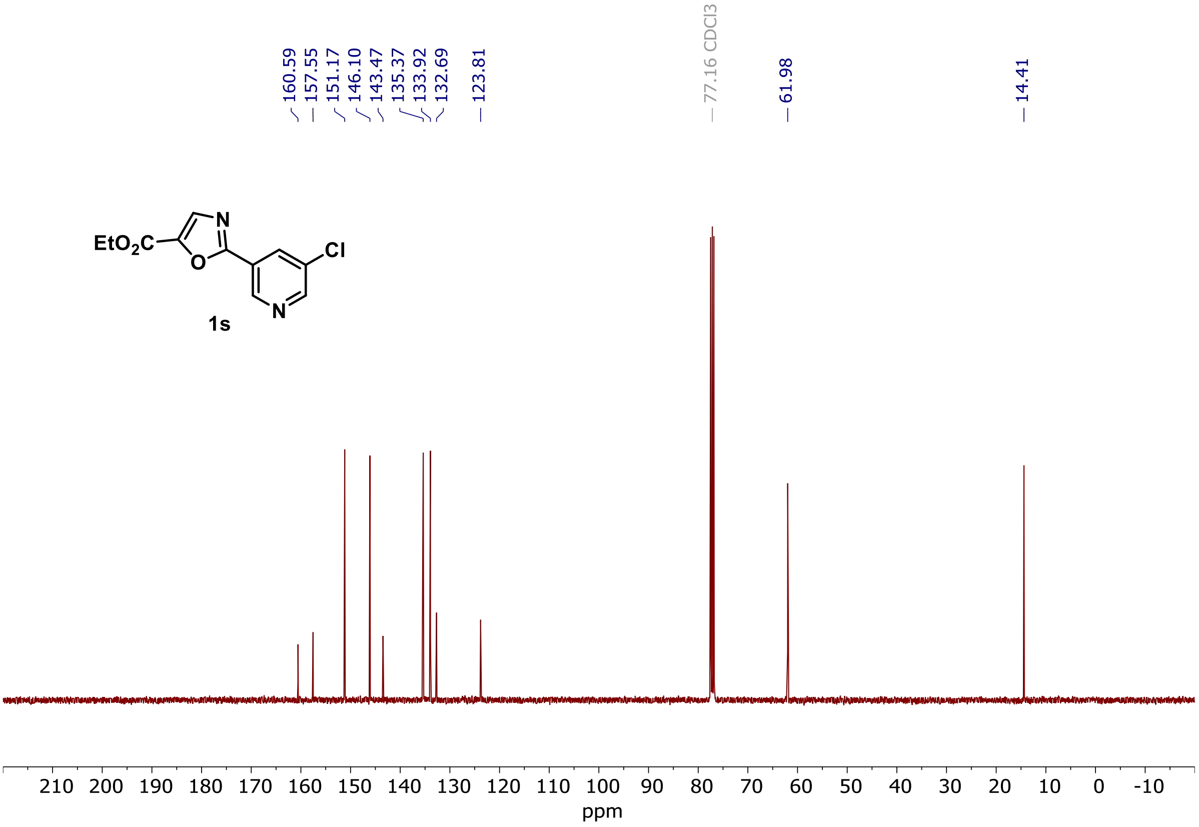

^{1}H NMR (400 MHz, $CDCl_3$) of **1t**

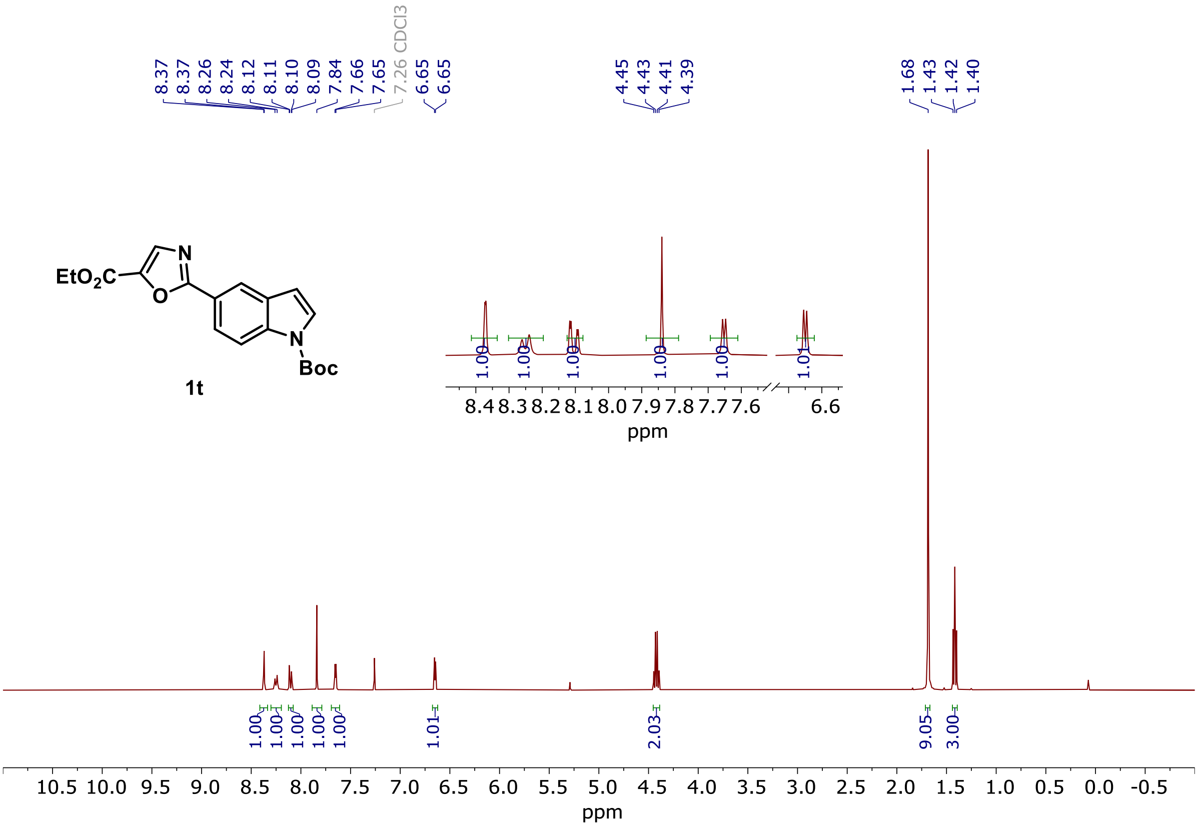

^{13}C NMR (101 MHz, $CDCl_3$) of **1t**

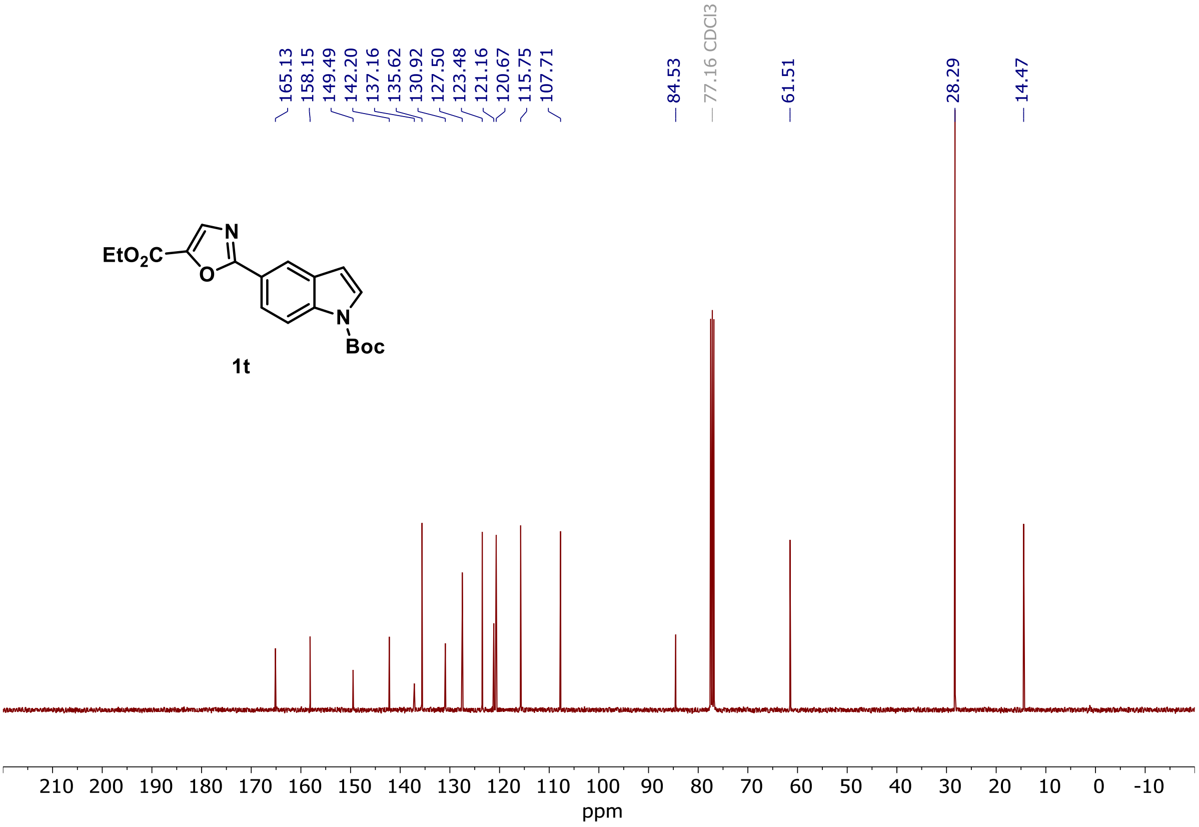

^{1}H NMR (400 MHz, $CDCl_3$) of **1u**

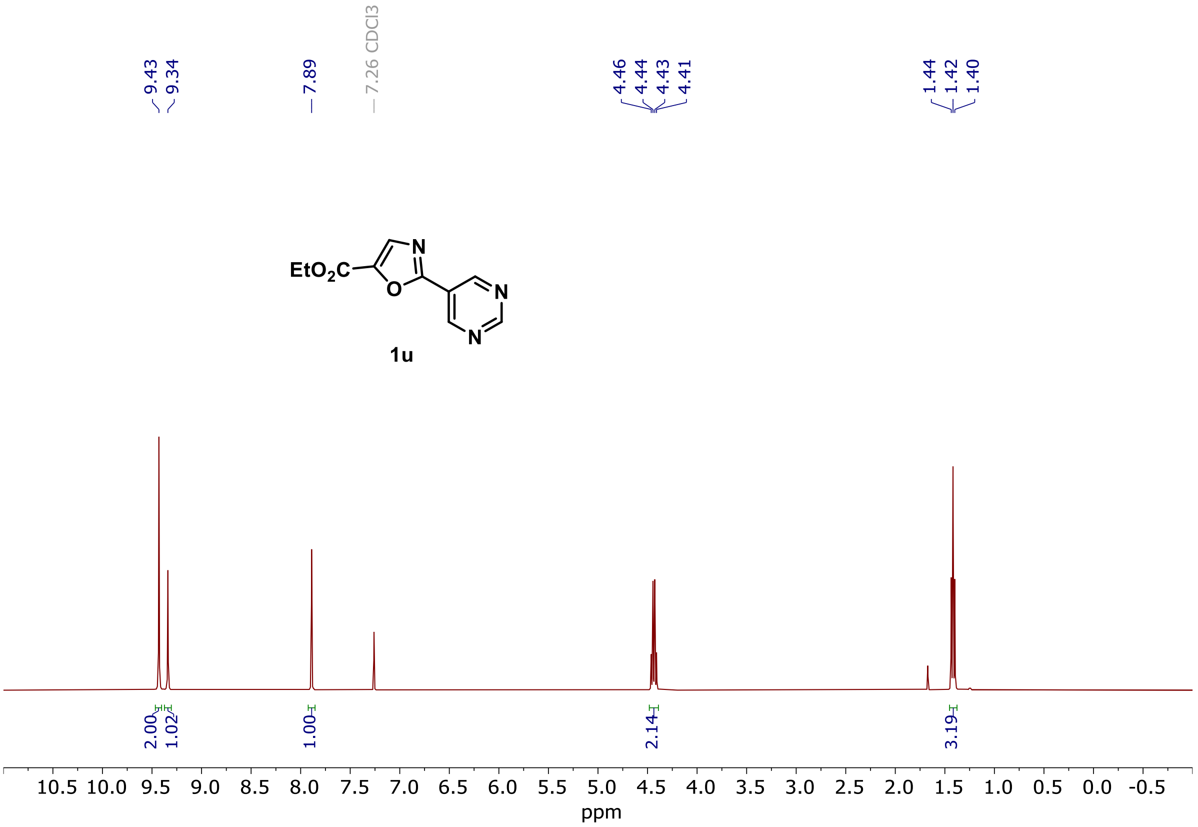

^{13}C NMR (101 MHz, $CDCl_3$) of **1u**

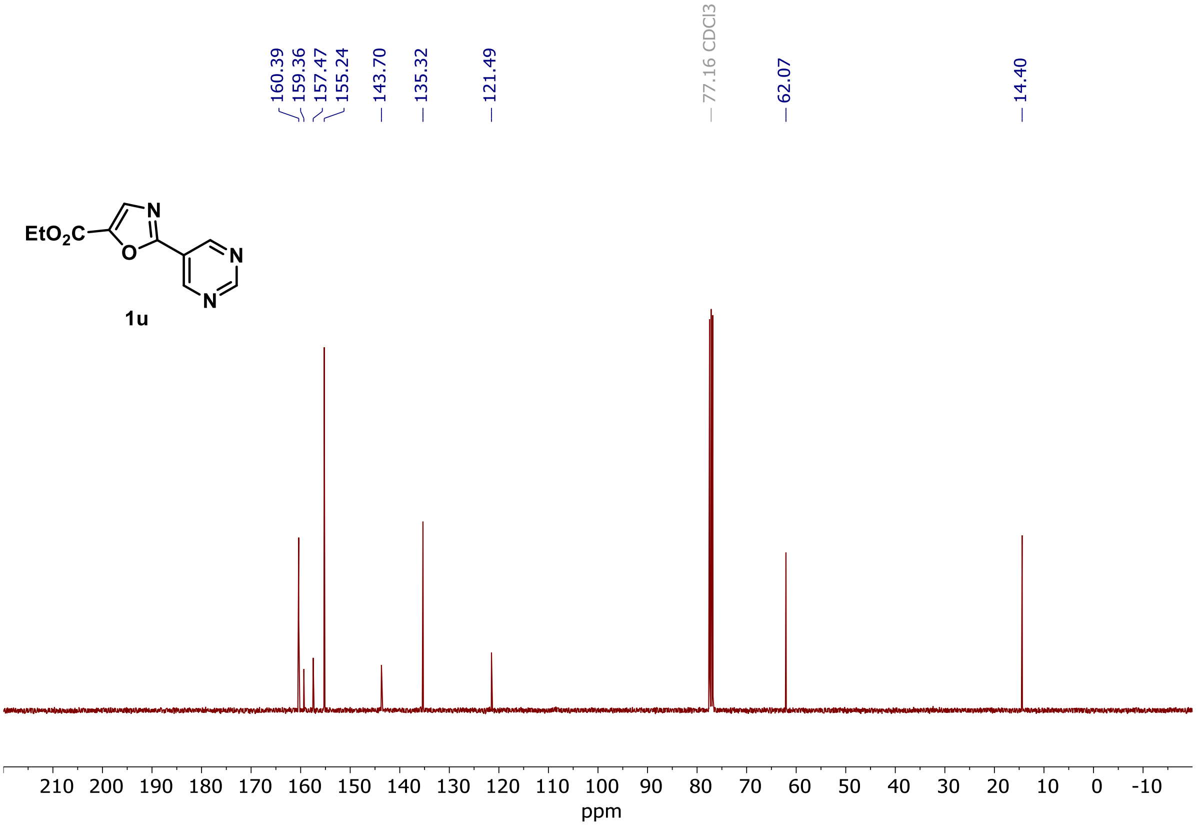

^{1}H NMR (400 MHz, $CDCl_3$) of **1v**

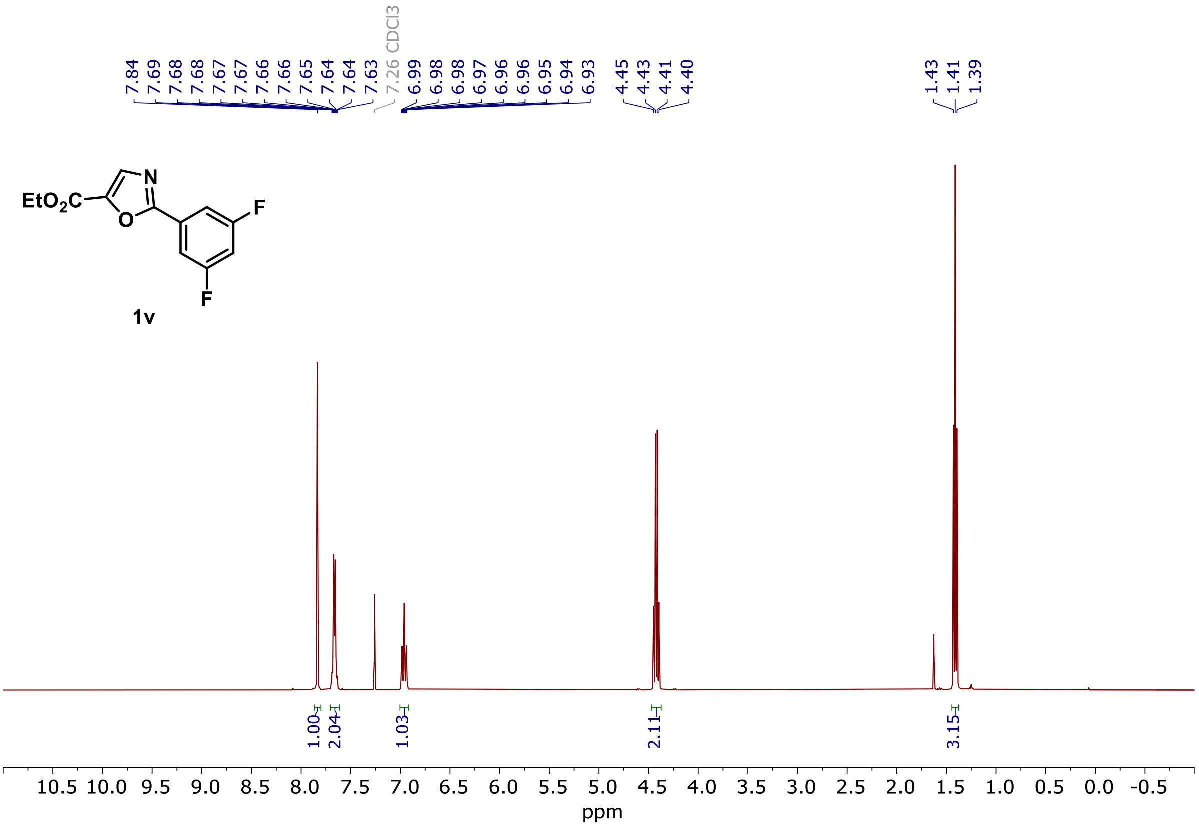

$^{13}C\{^{19}F\}$ NMR (126 MHz, $CDCl_3$) of **1v**

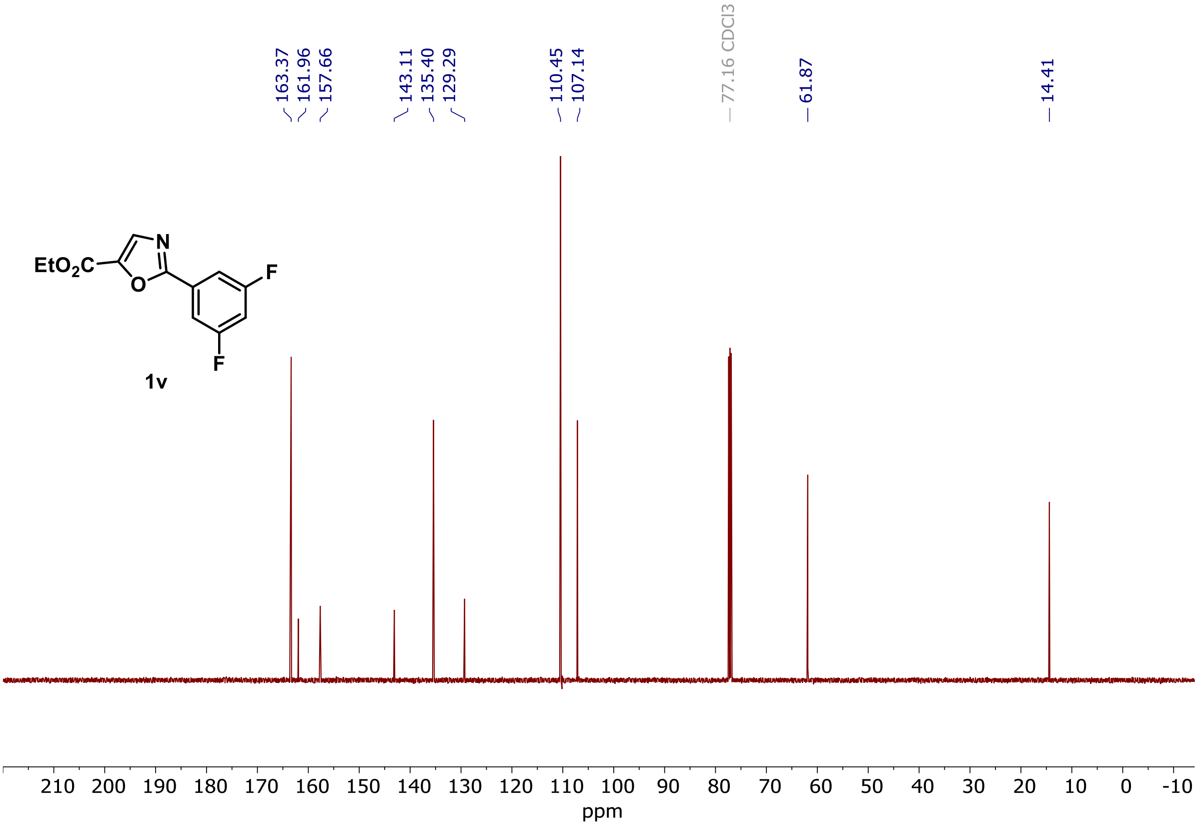

^{13}C NMR (126 MHz, $CDCl_3$) of **1v**

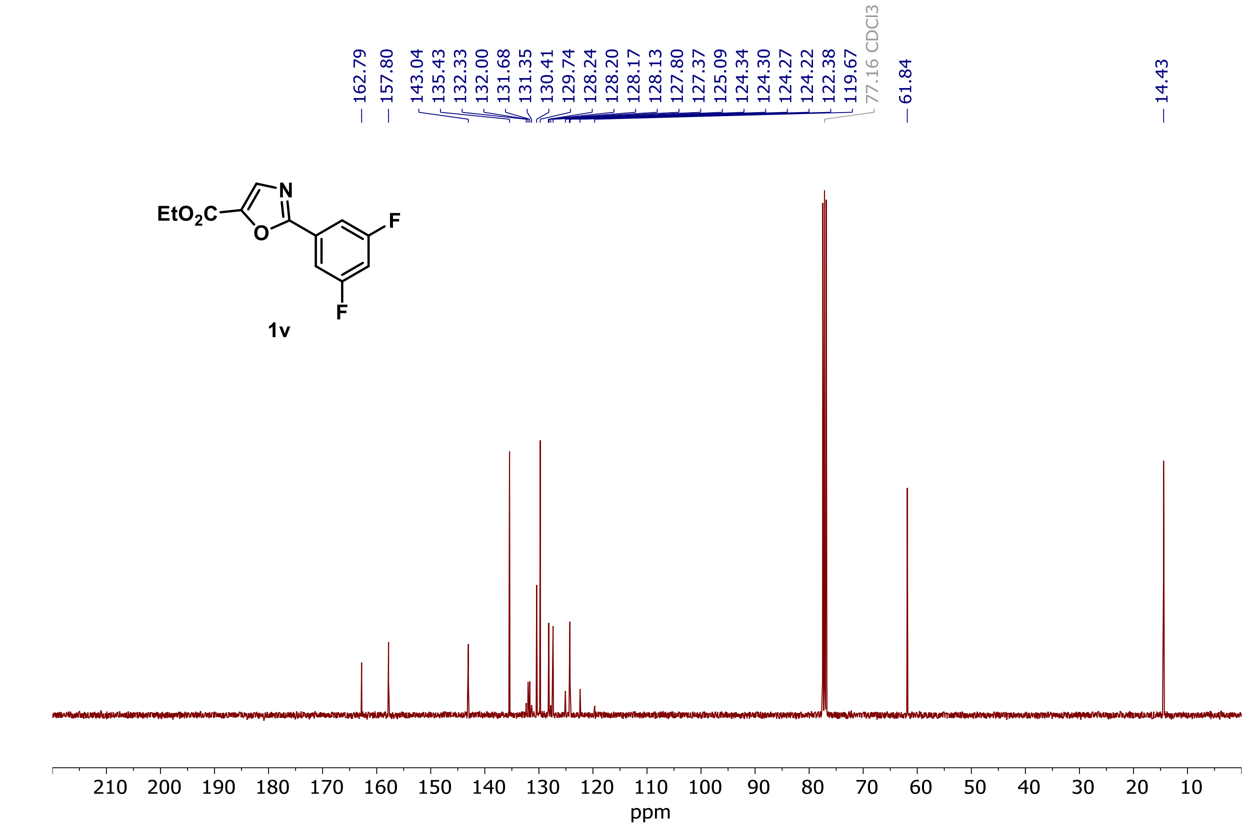

^{19}F NMR (376 MHz, $CDCl_3$) of **1v**

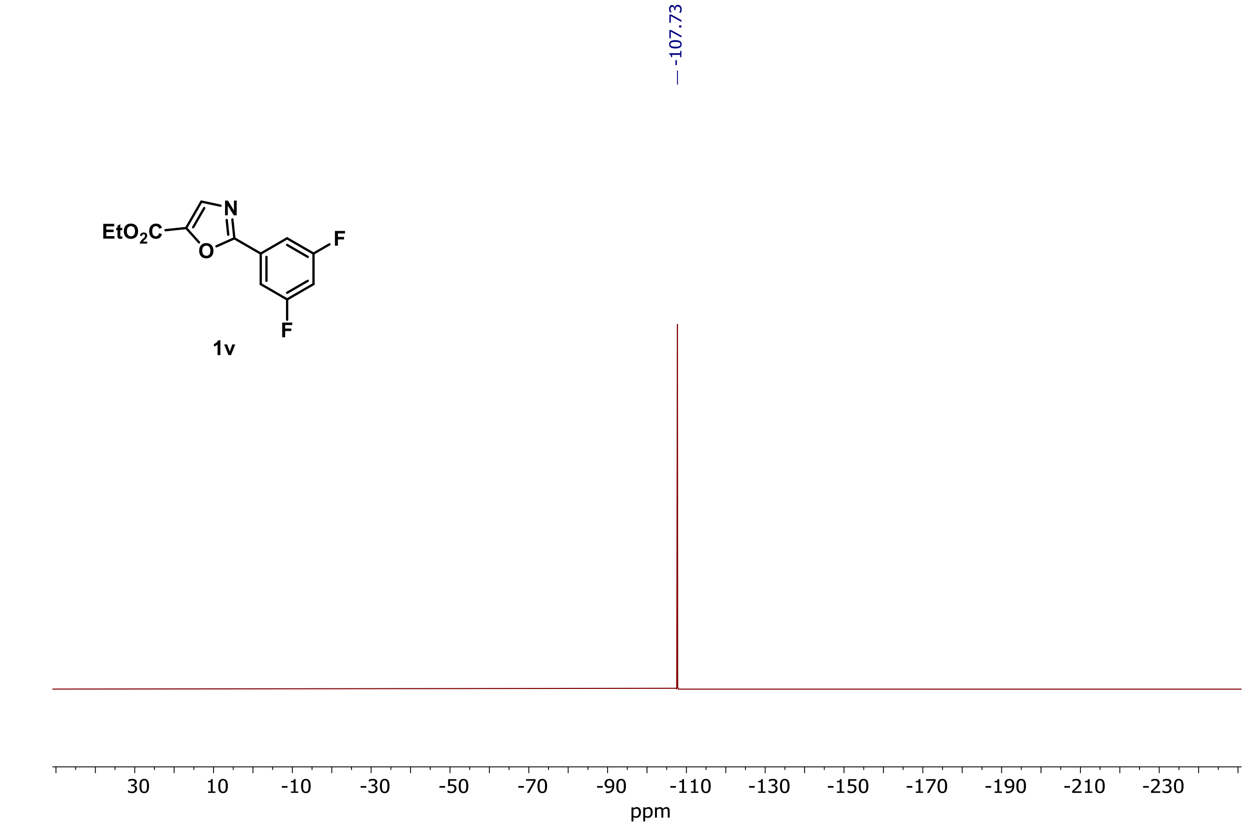

^{1}H NMR (400 MHz, $CDCl_3$) of **1w**

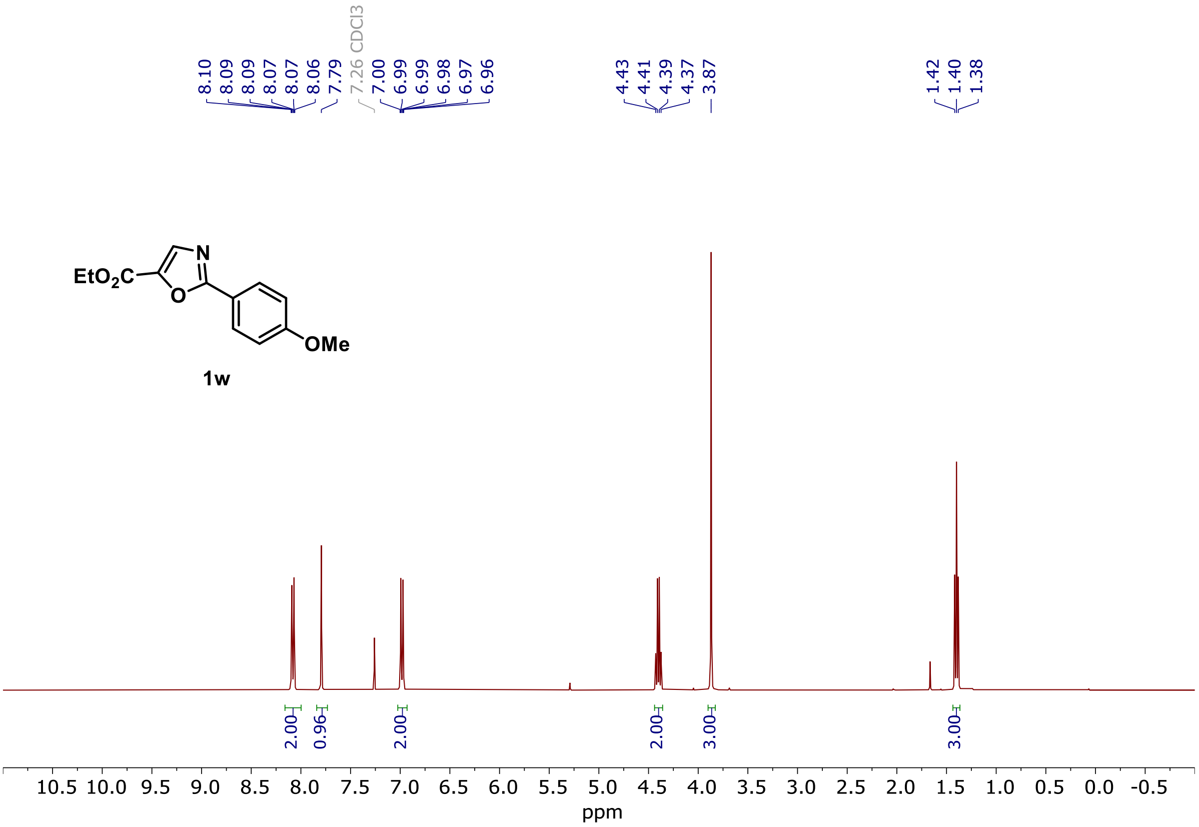

^{13}C NMR (101 MHz, $CDCl_3$) of **1w**

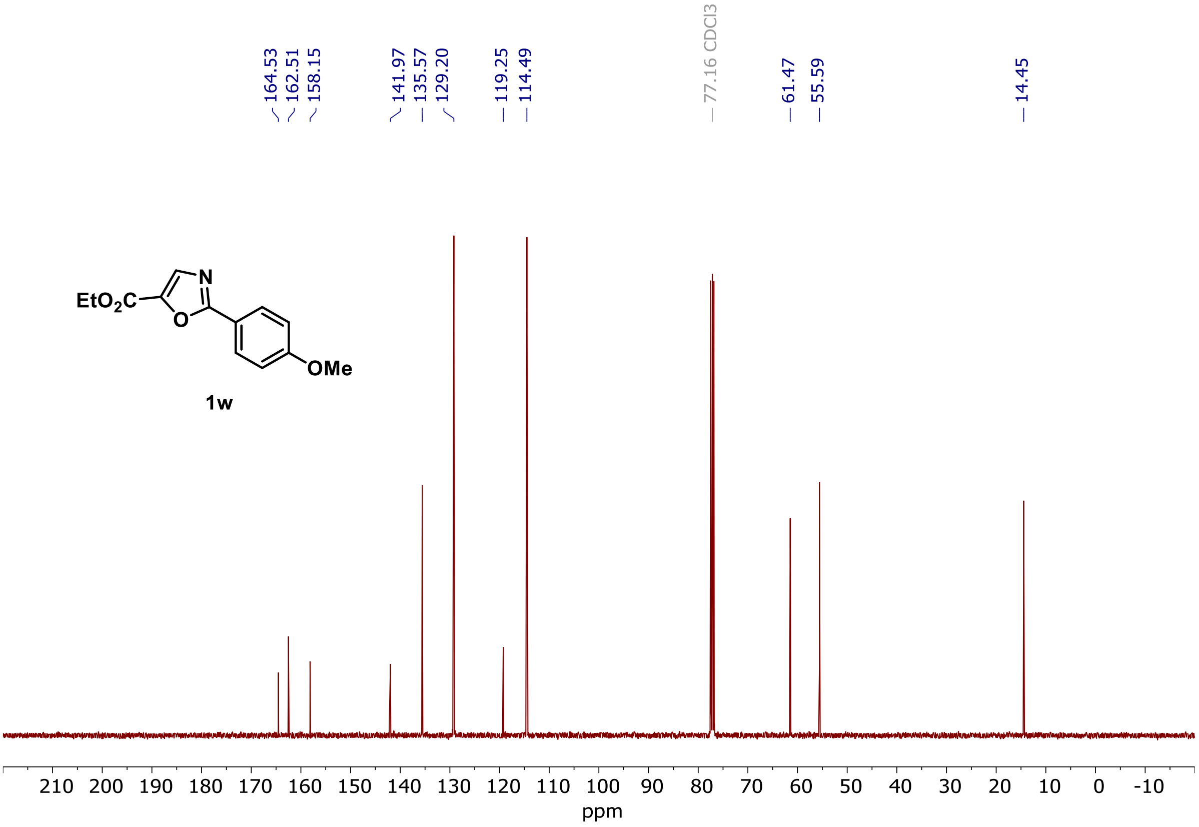

^{1}H NMR (400 MHz, $CDCl_3$) of **1x**

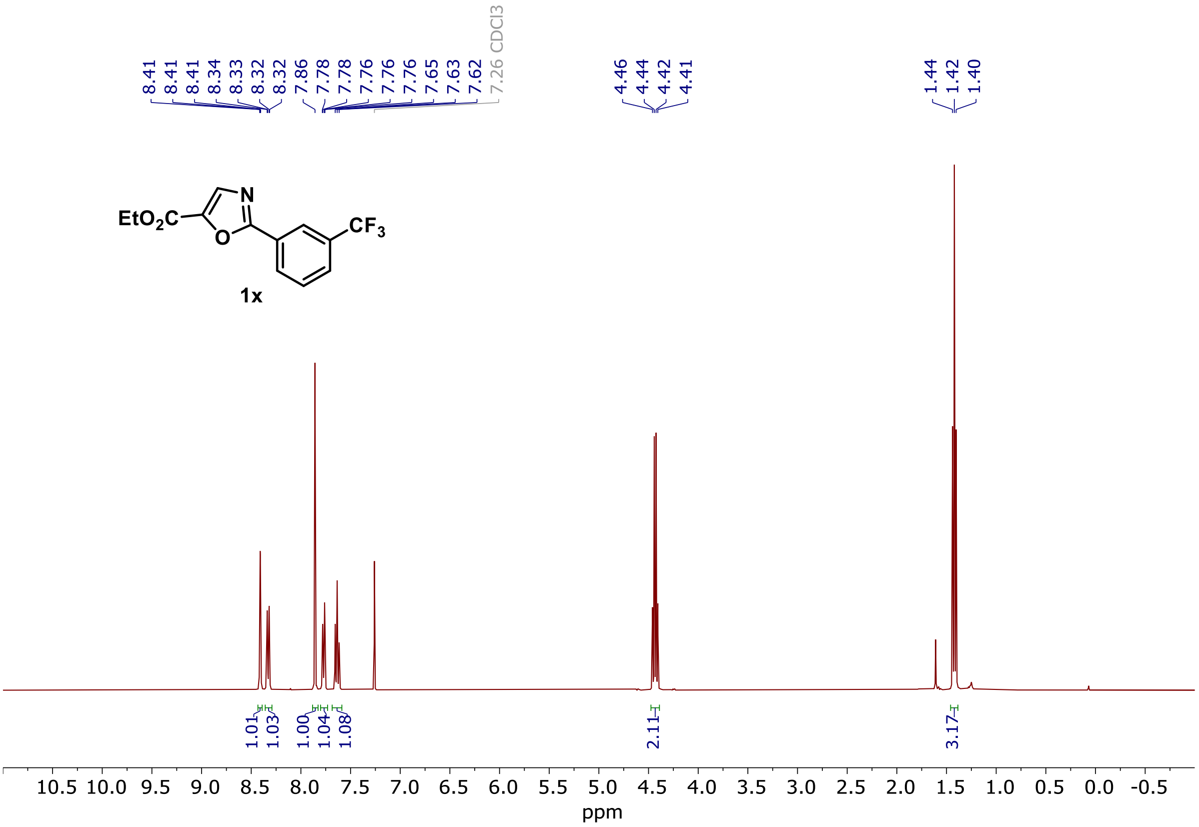

^{13}C{^{19}F} NMR (126 MHz, $CDCl_3$) of **1x**

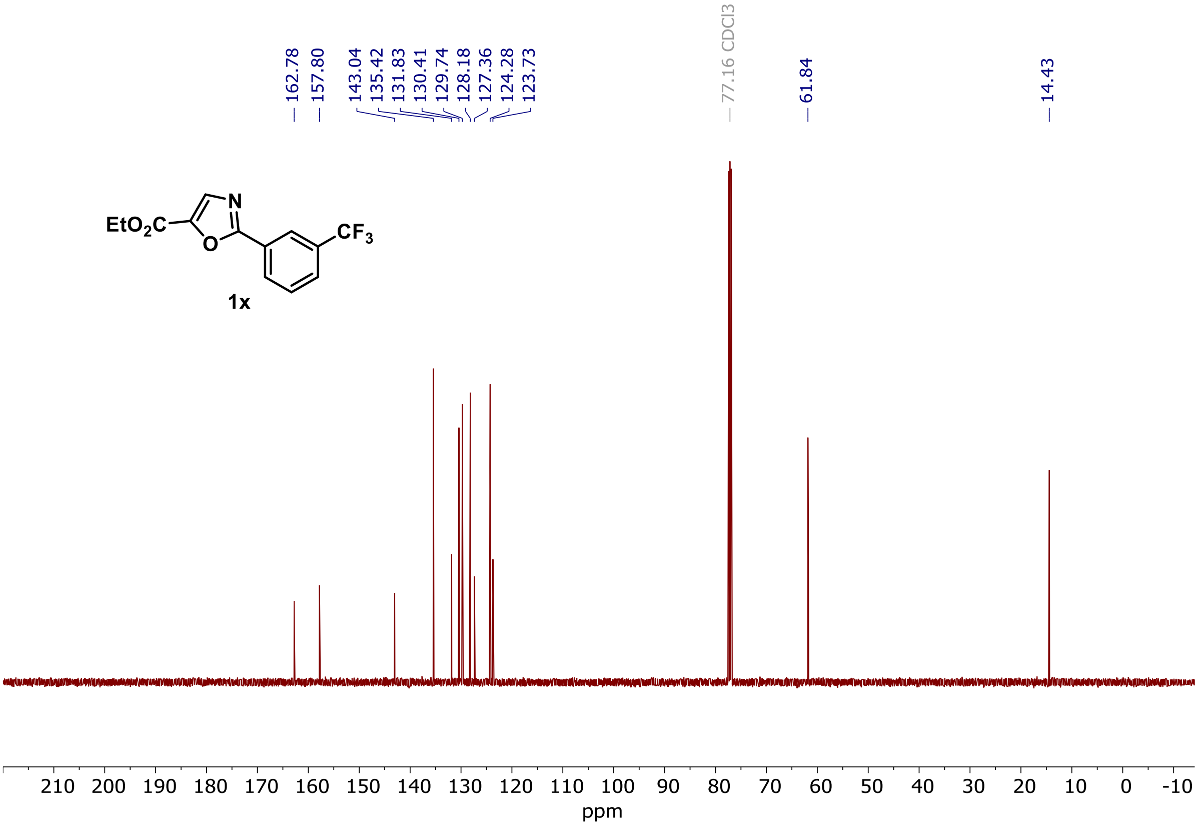

^{13}C NMR (126 MHz, $CDCl_3$) of **1x**

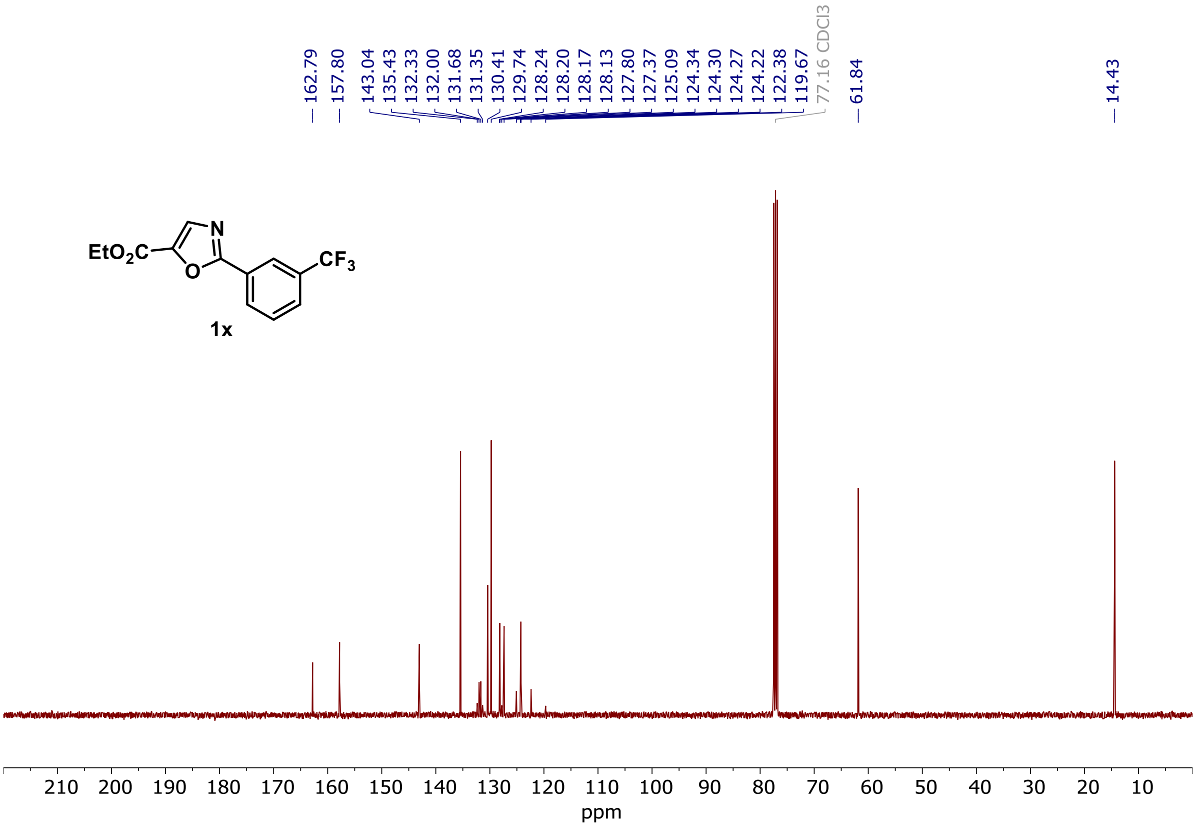

^{19}F NMR (376 MHz, $CDCl_3$) of **1x**

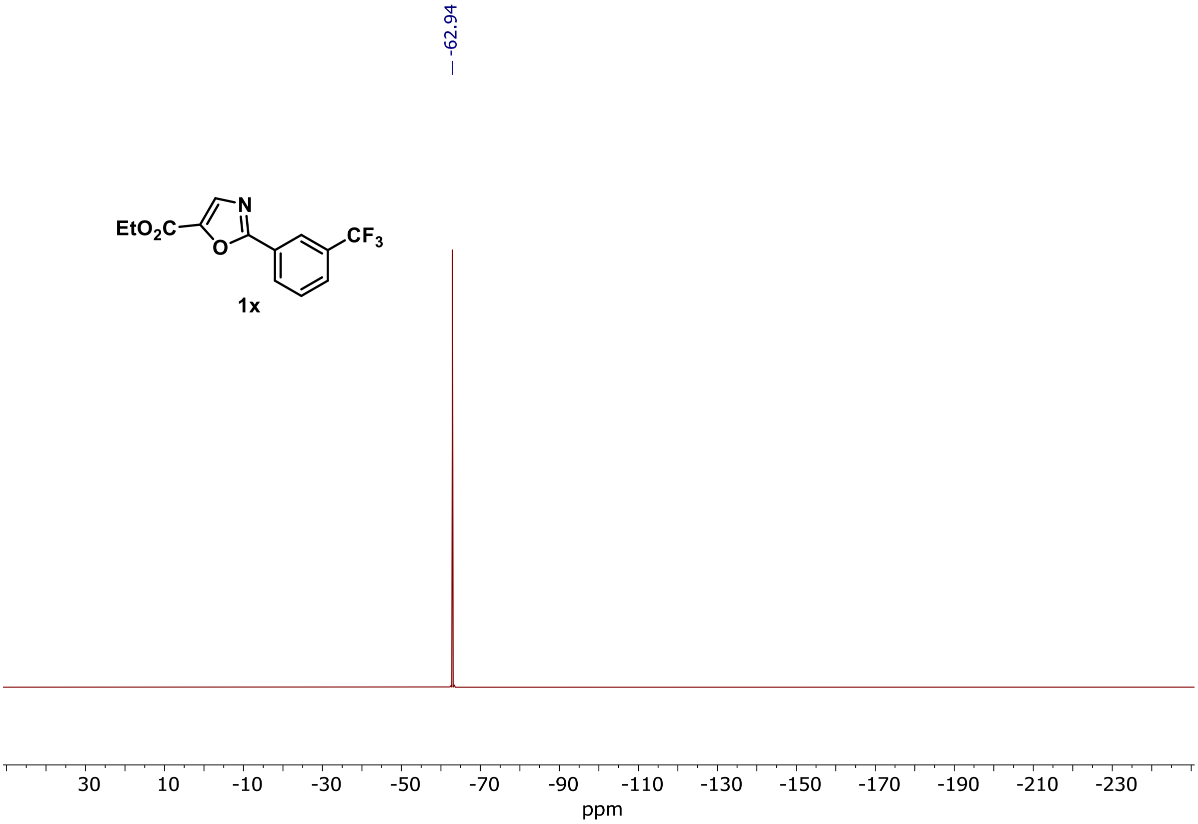

^{1}H NMR (400 MHz, $CDCl_3$) of **1y**

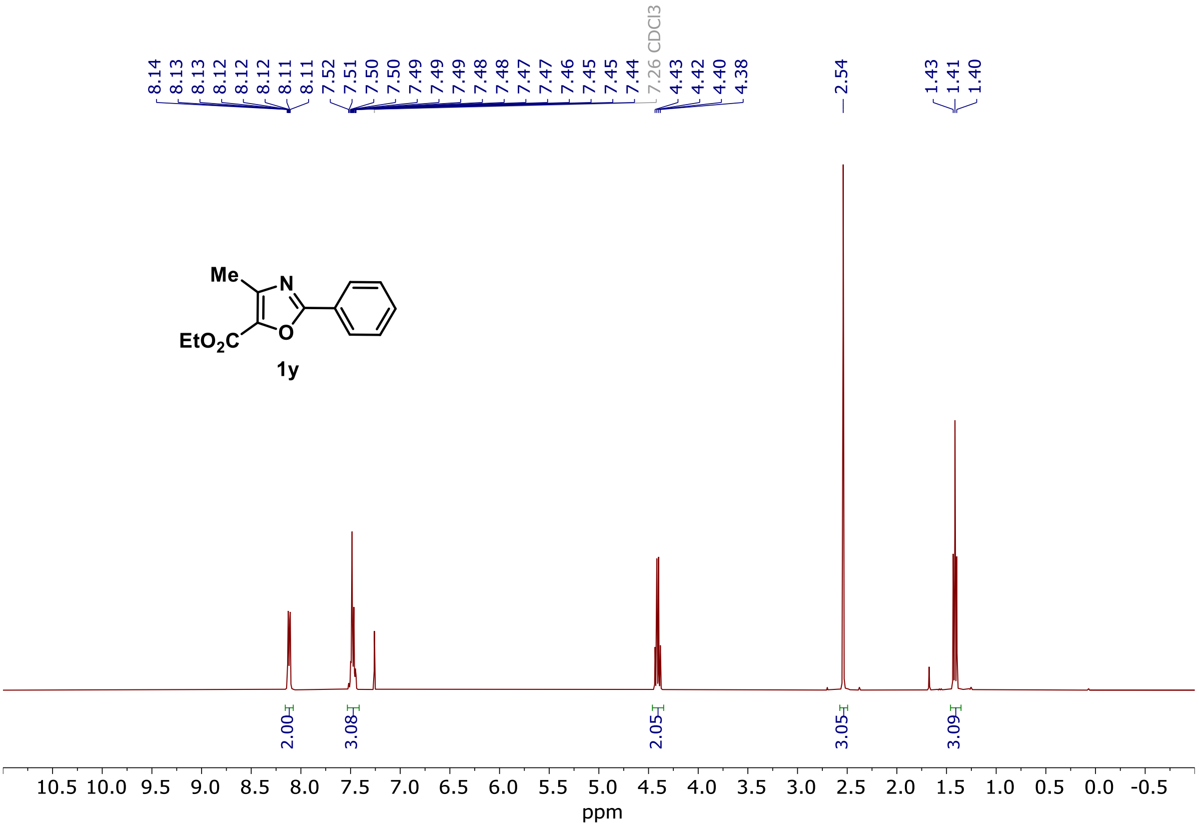

^{13}C NMR (101 MHz, $CDCl_3$) of **1y**

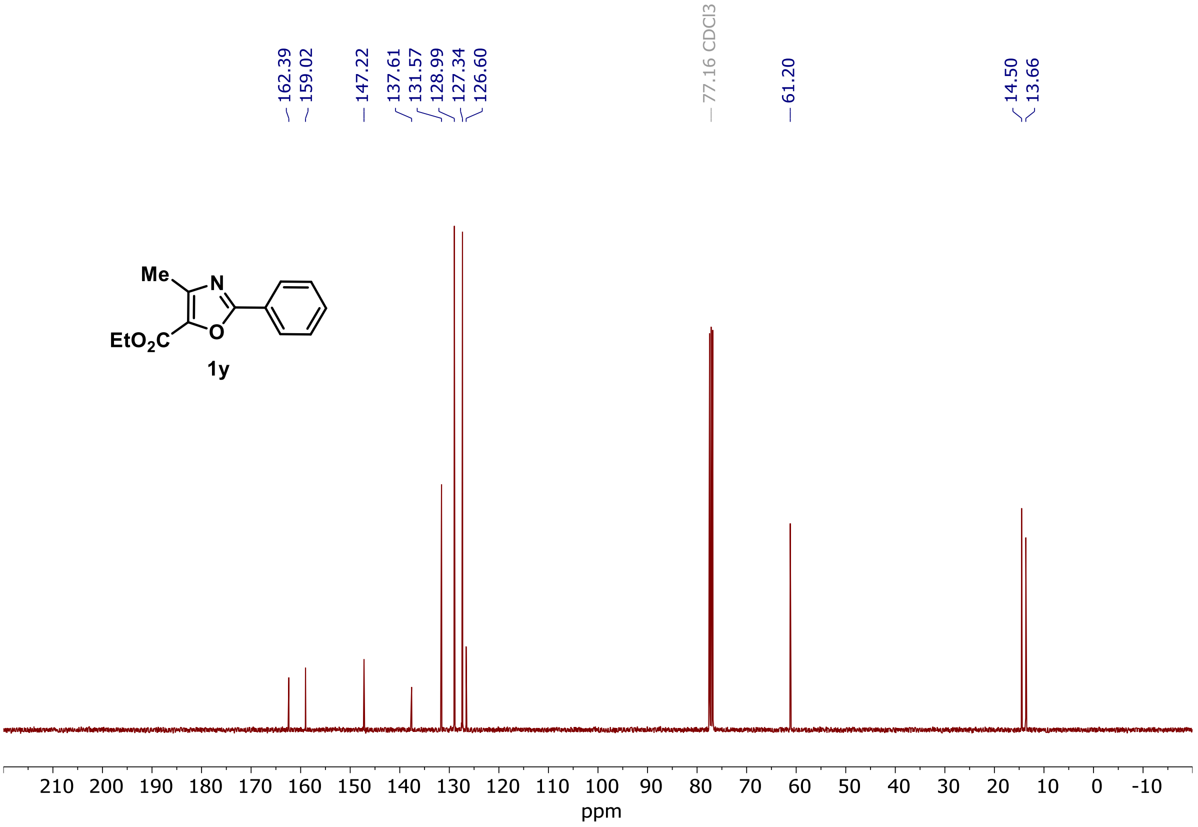

^{1}H NMR (400 MHz, $CDCl_3$) of **1aa**

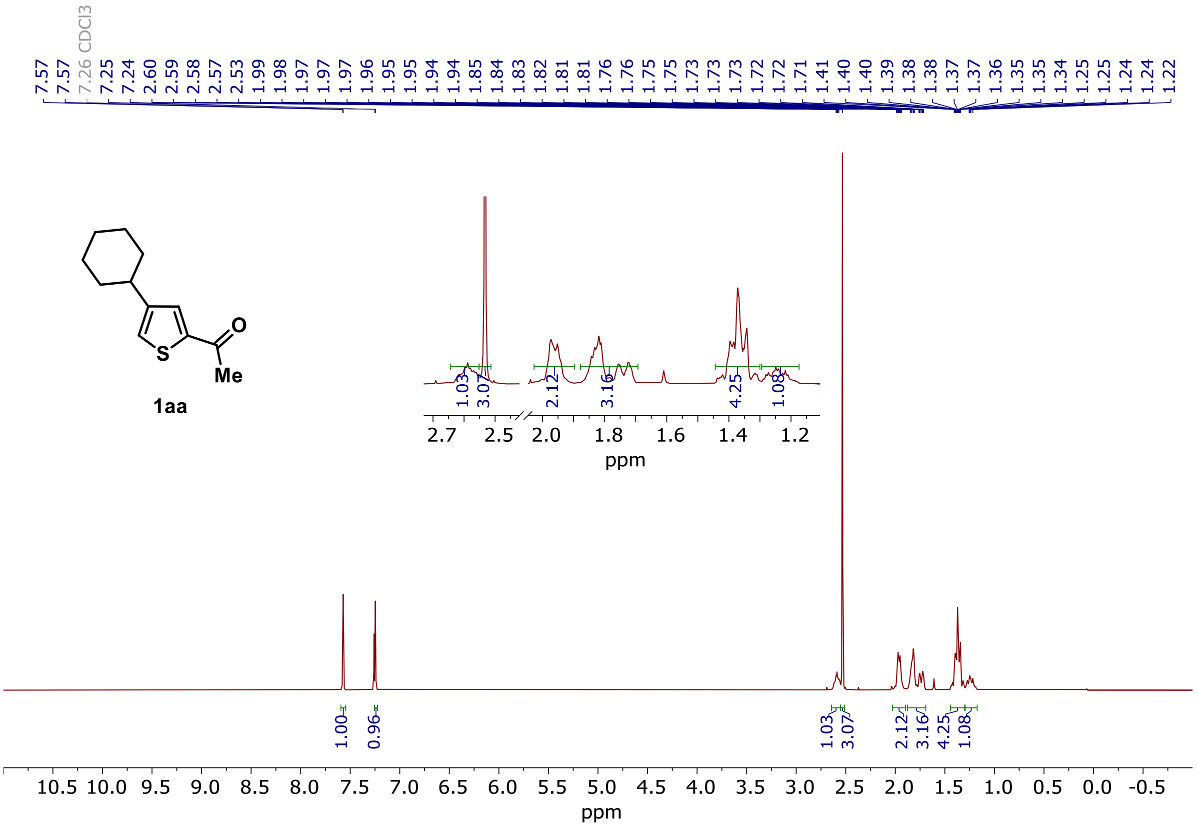

^{13}C NMR (101 MHz, $CDCl_3$) of **1aa**

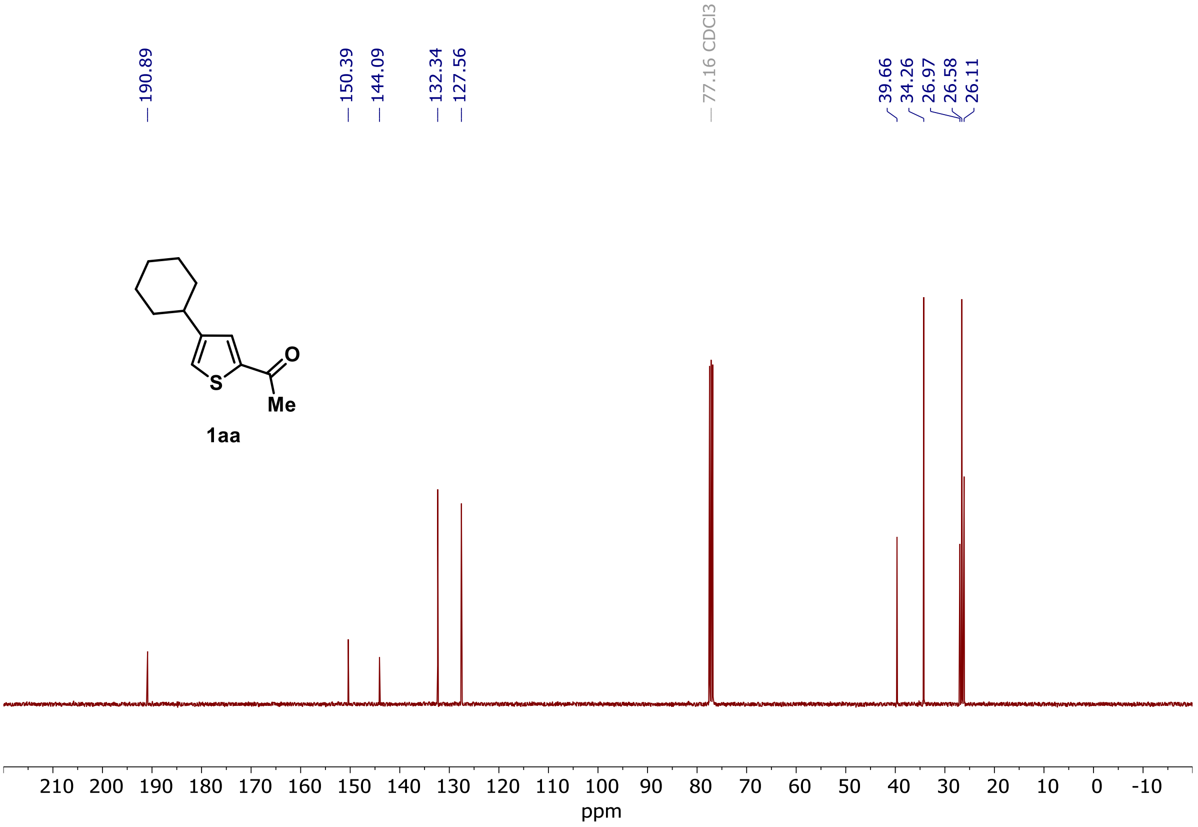

^{1}H NMR (400 MHz, $CDCl_3$) of **1ak**

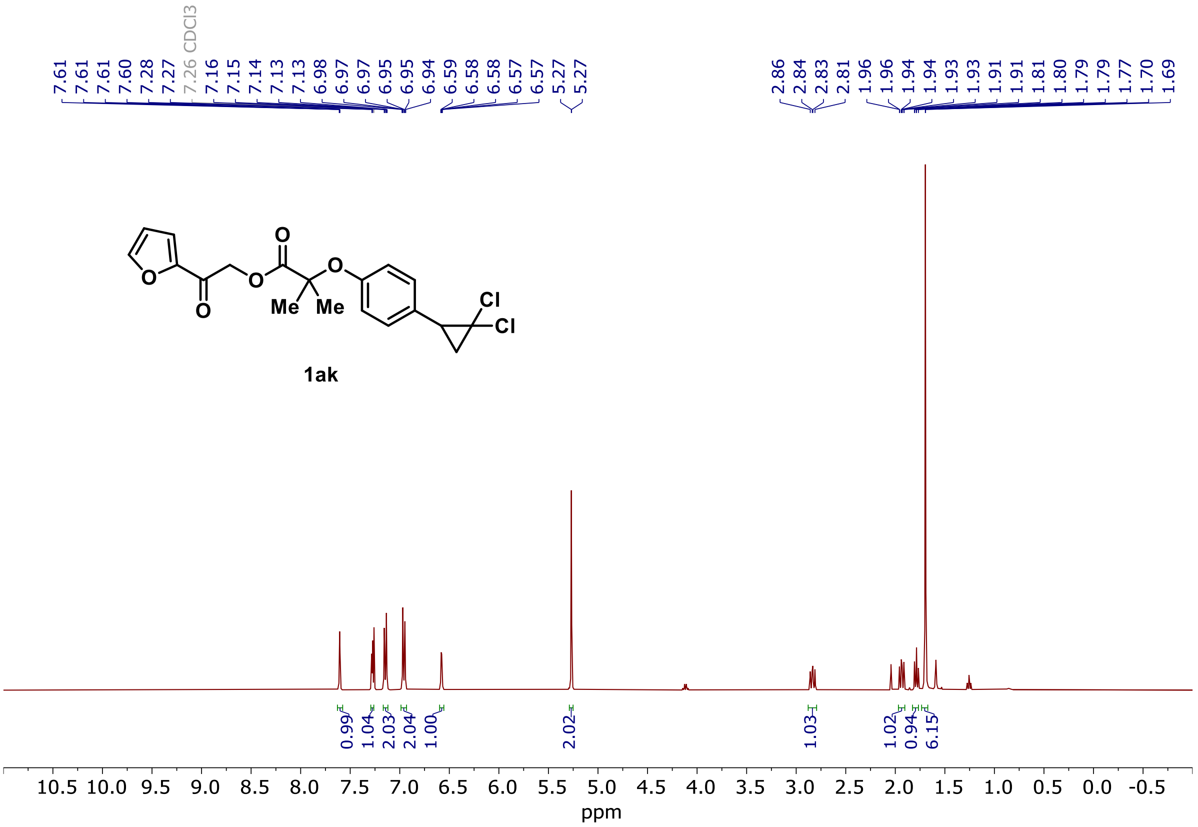

^{13}C NMR (101 MHz, $CDCl_3$) of **1ak**

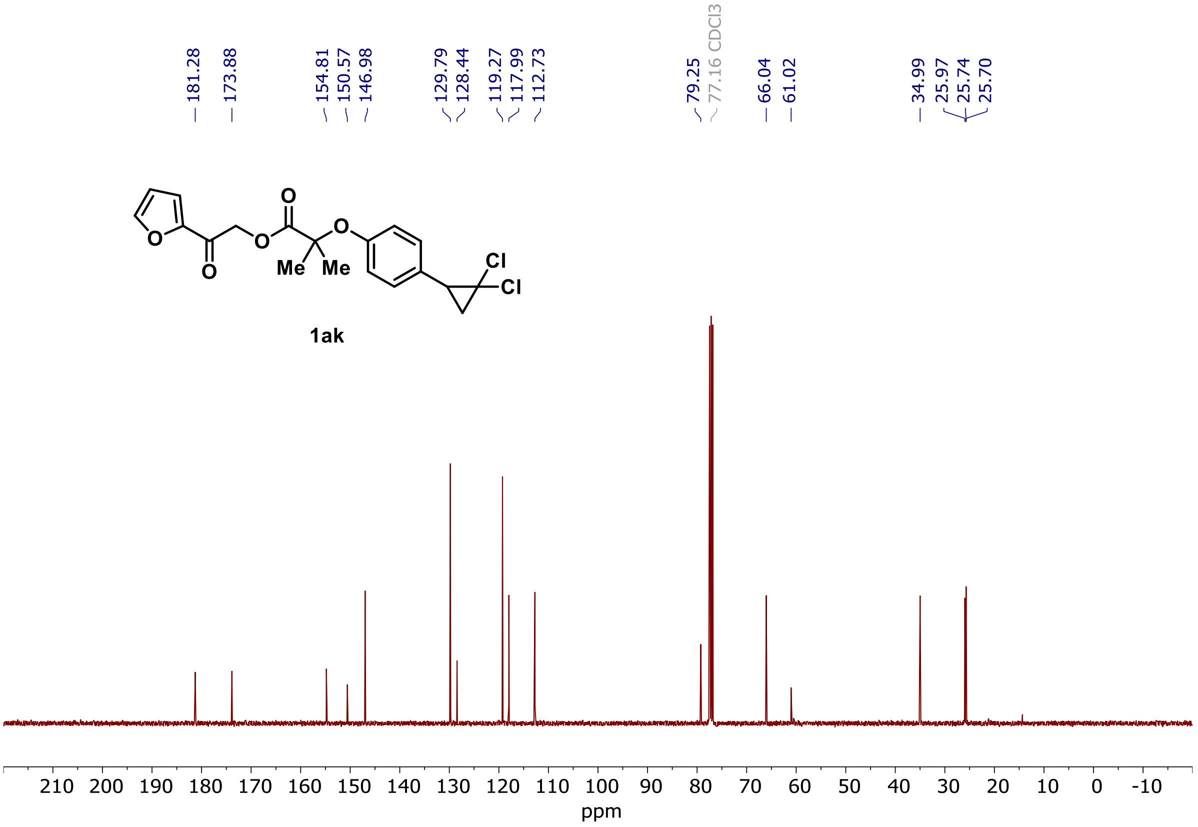

1H NMR (400 MHz, CDCl3) of **1al**

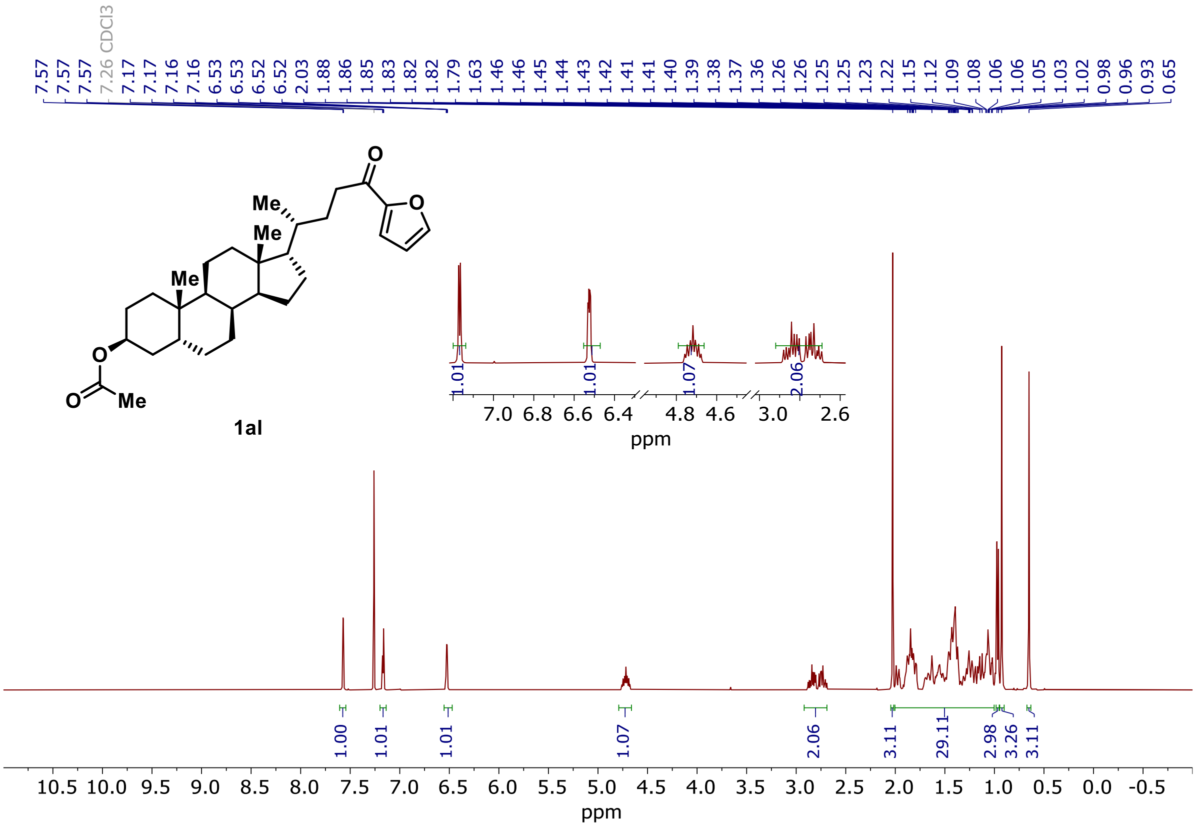

^{13}C NMR (101 MHz, CDCl3) of **1al**

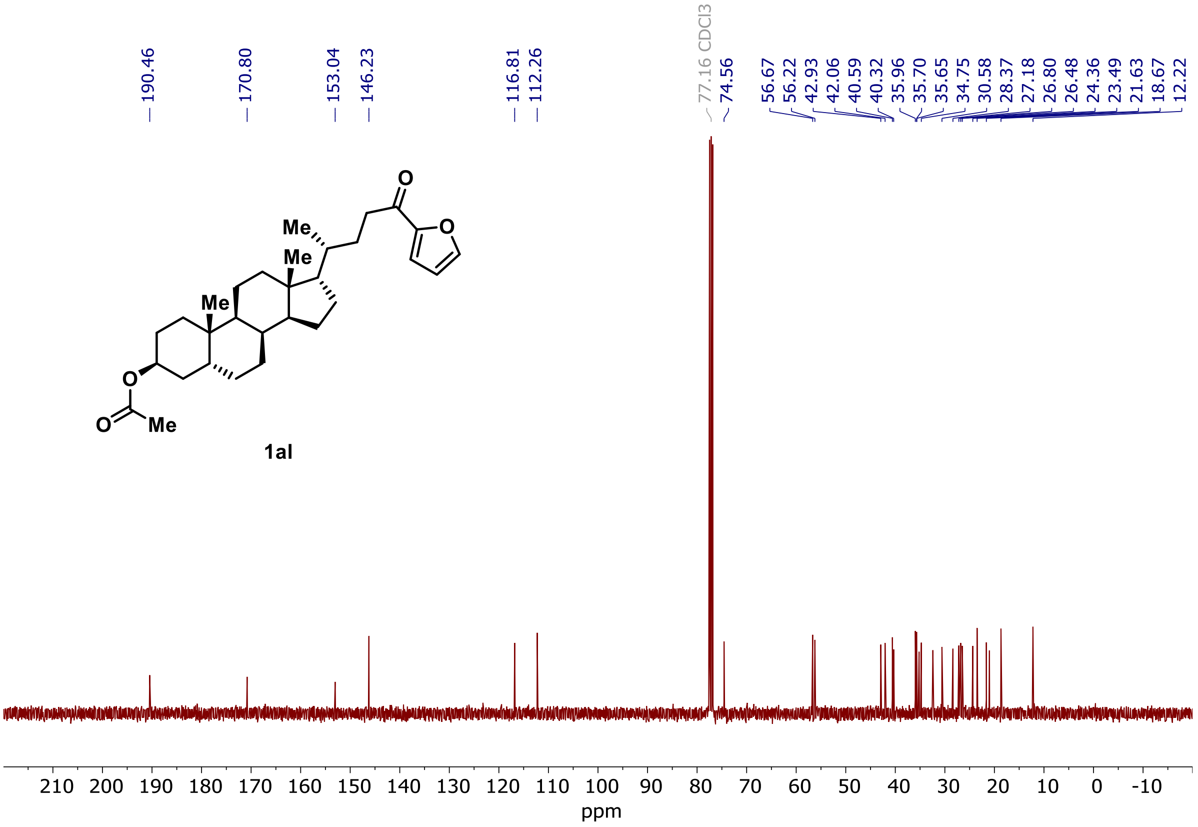

^{1}H NMR (400 MHz, $CDCl_3$) of **1am**

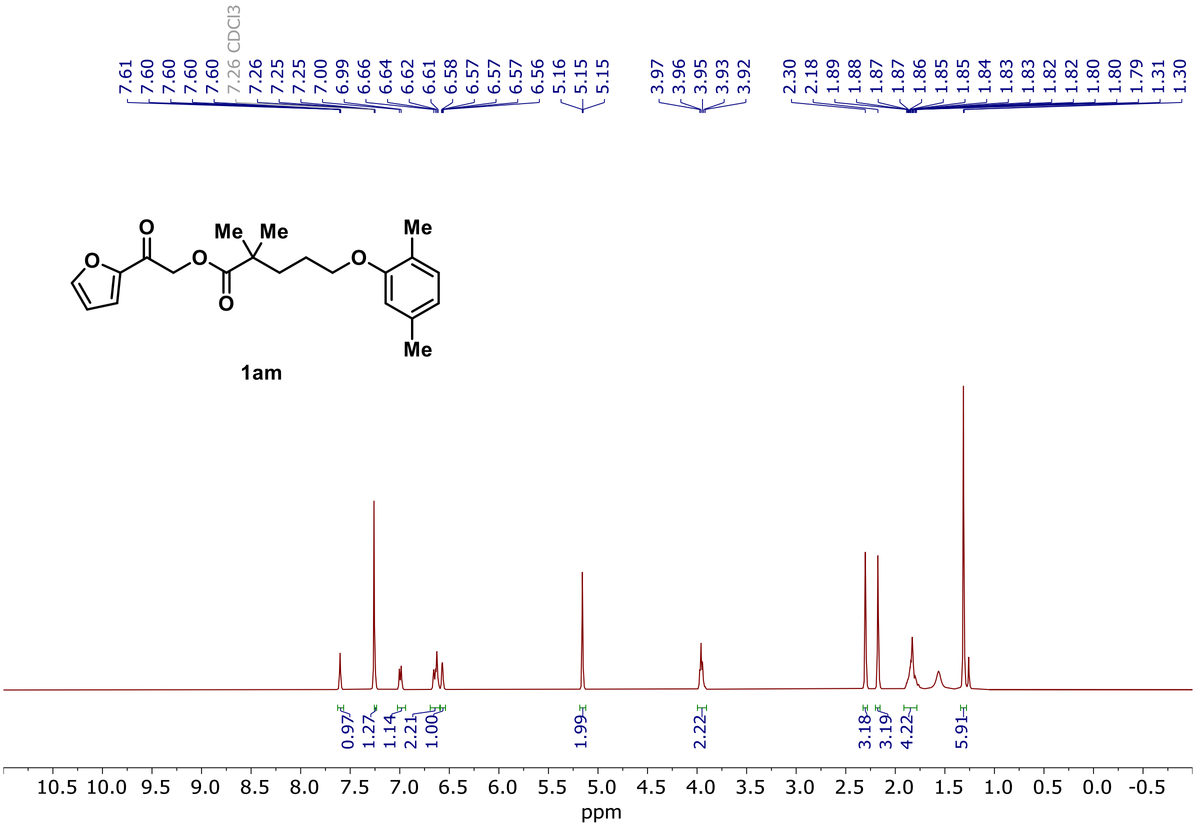

^{13}C NMR (101 MHz, $CDCl_3$) of **1am**

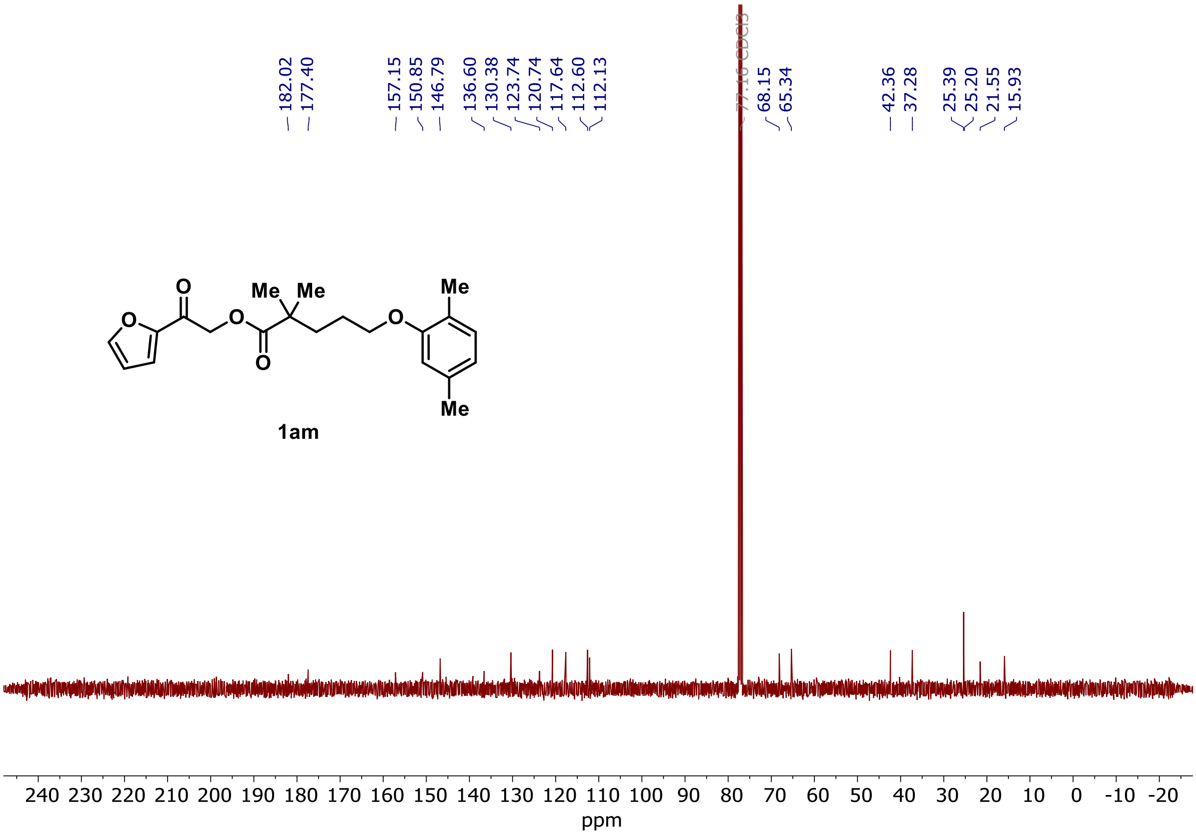

^{1}H NMR (400 MHz, $CDCl_3$) of **1an**

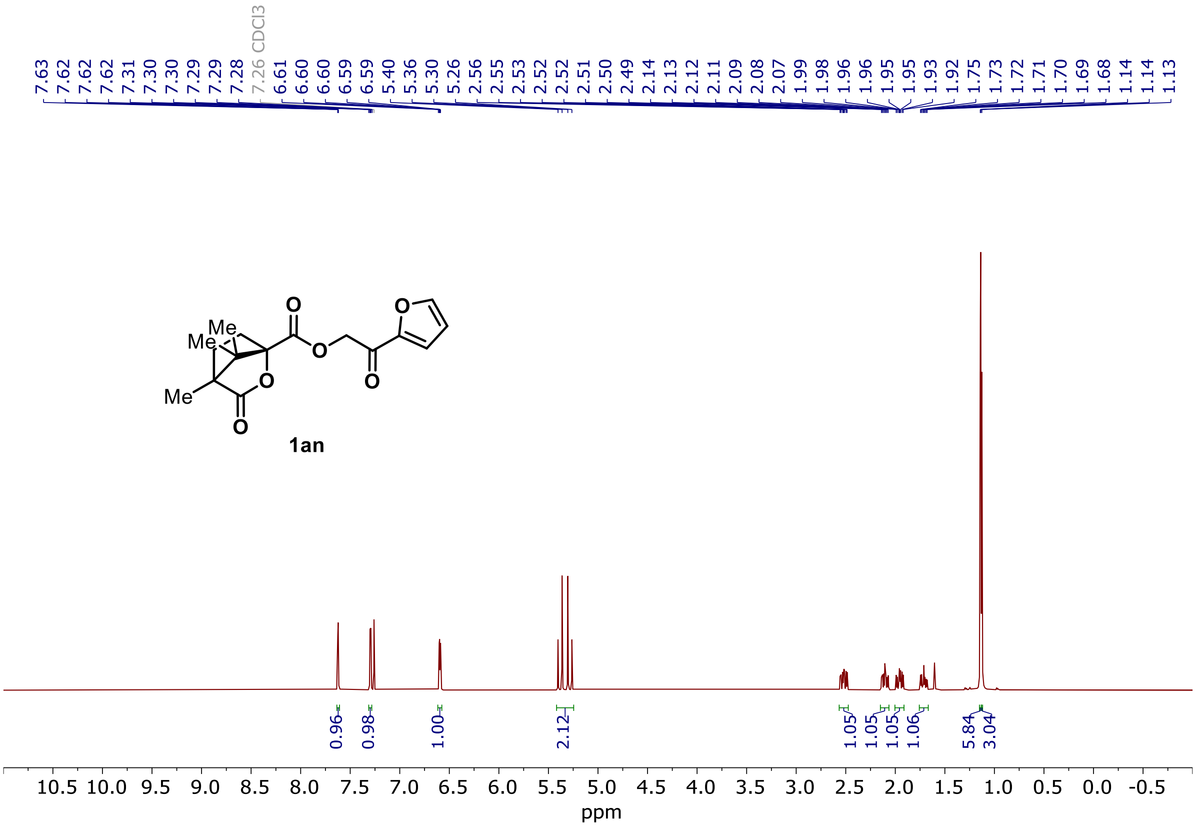

^{13}C NMR (101 MHz, $CDCl_3$) of **1an**

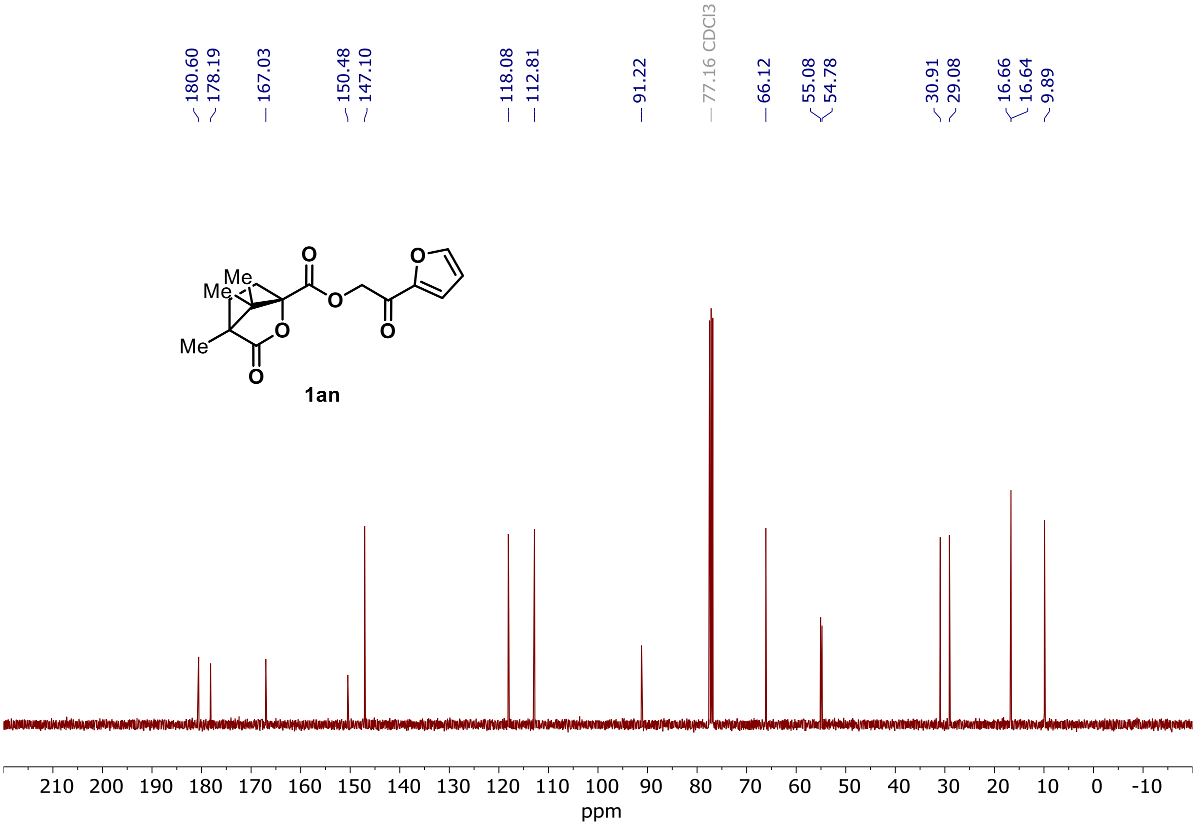

^{1}H NMR (400 MHz, $CDCl_3$) of **1ao**

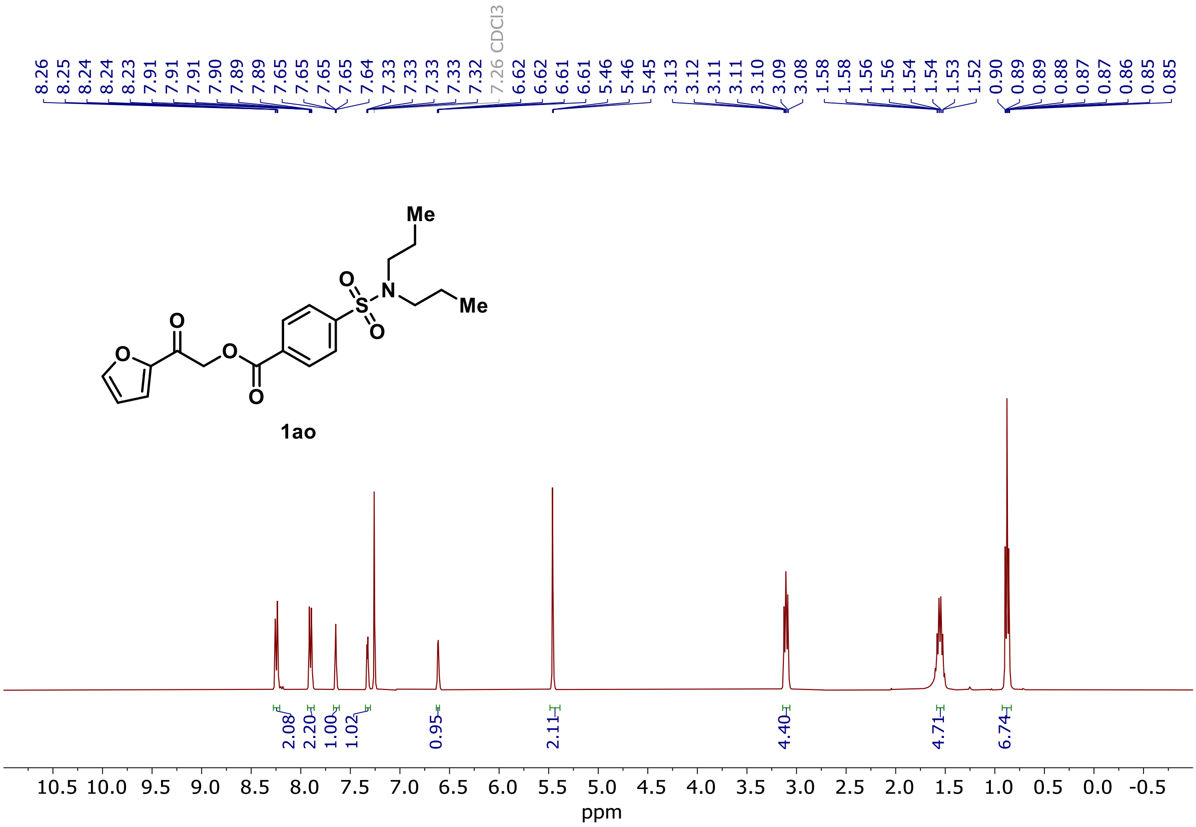

^{13}C NMR (101 MHz, $CDCl_3$) of **1ao**

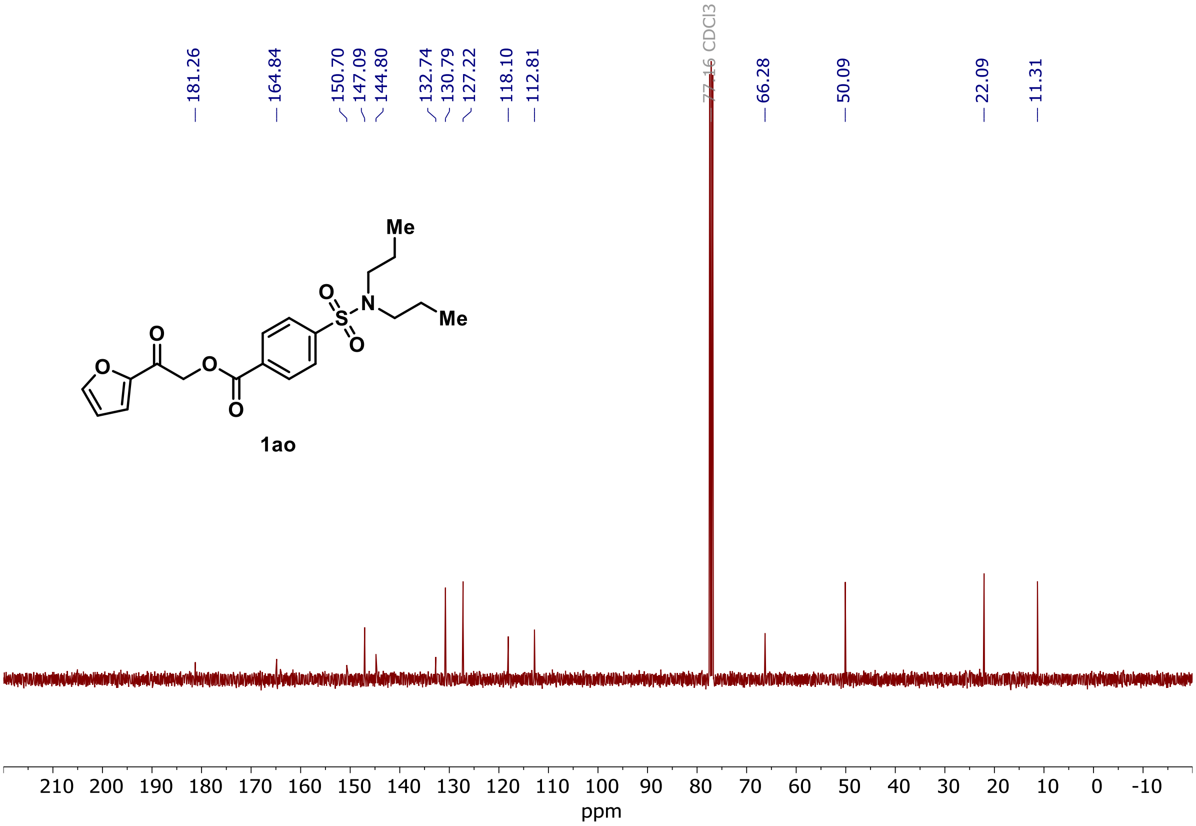

^{1}H NMR (400 MHz, $CDCl_3$) of **1ap**

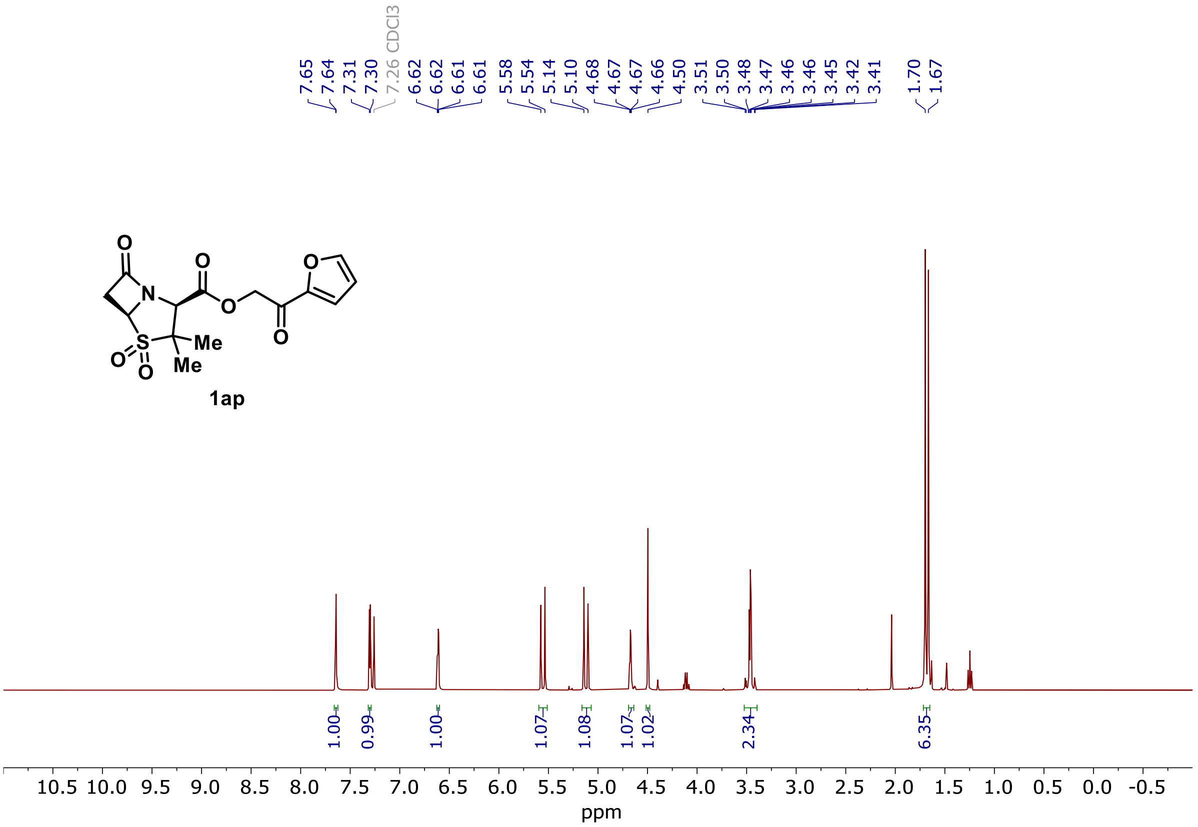

^{13}C NMR (101 MHz, $CDCl_3$) of **1ap**

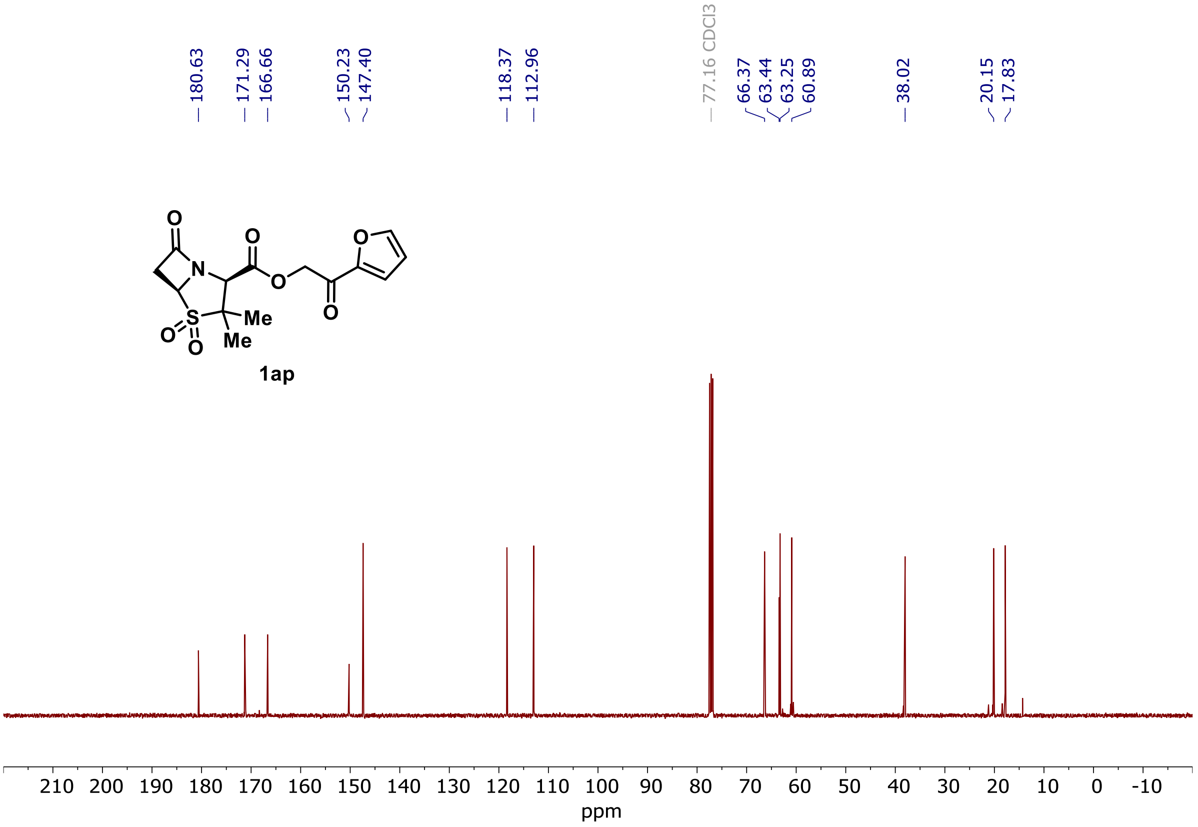

^{1}H NMR (400 MHz, $CDCl_3$) of **1aq**

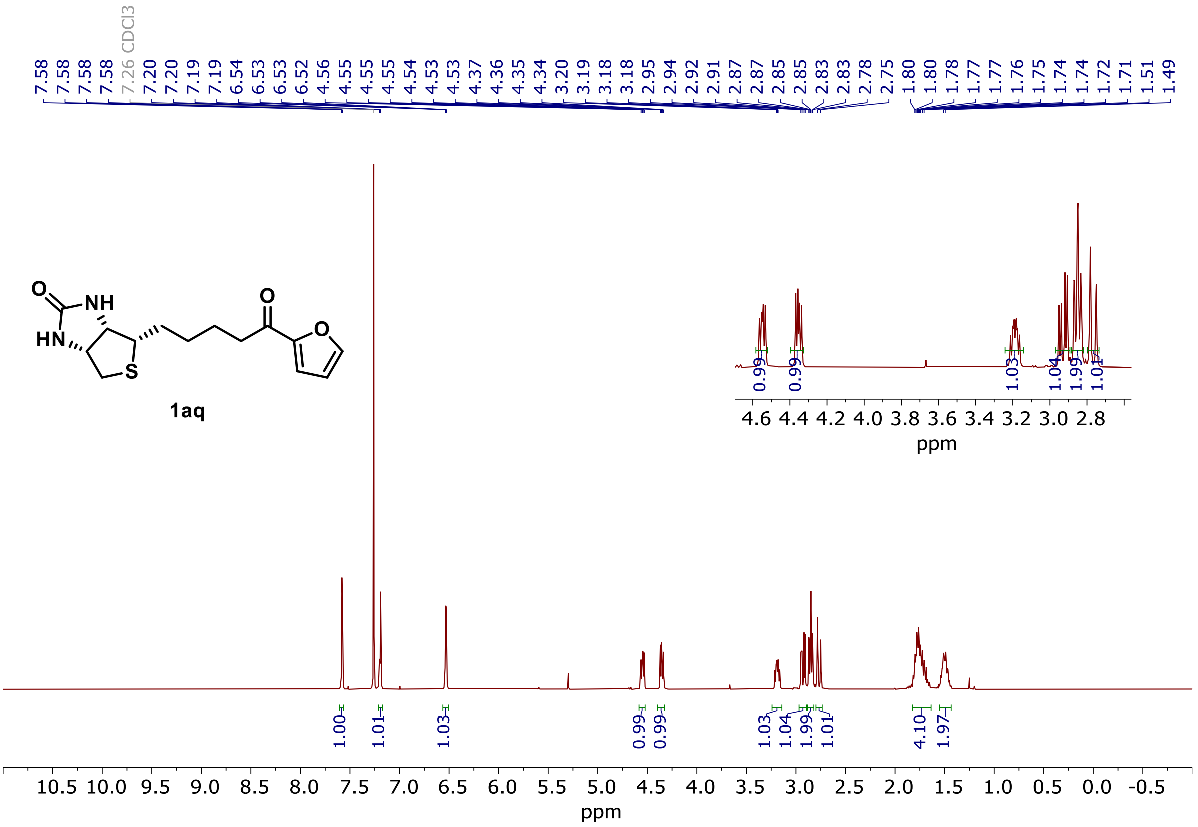

^{13}C NMR (101 MHz, $CDCl_3$) of **1aq**

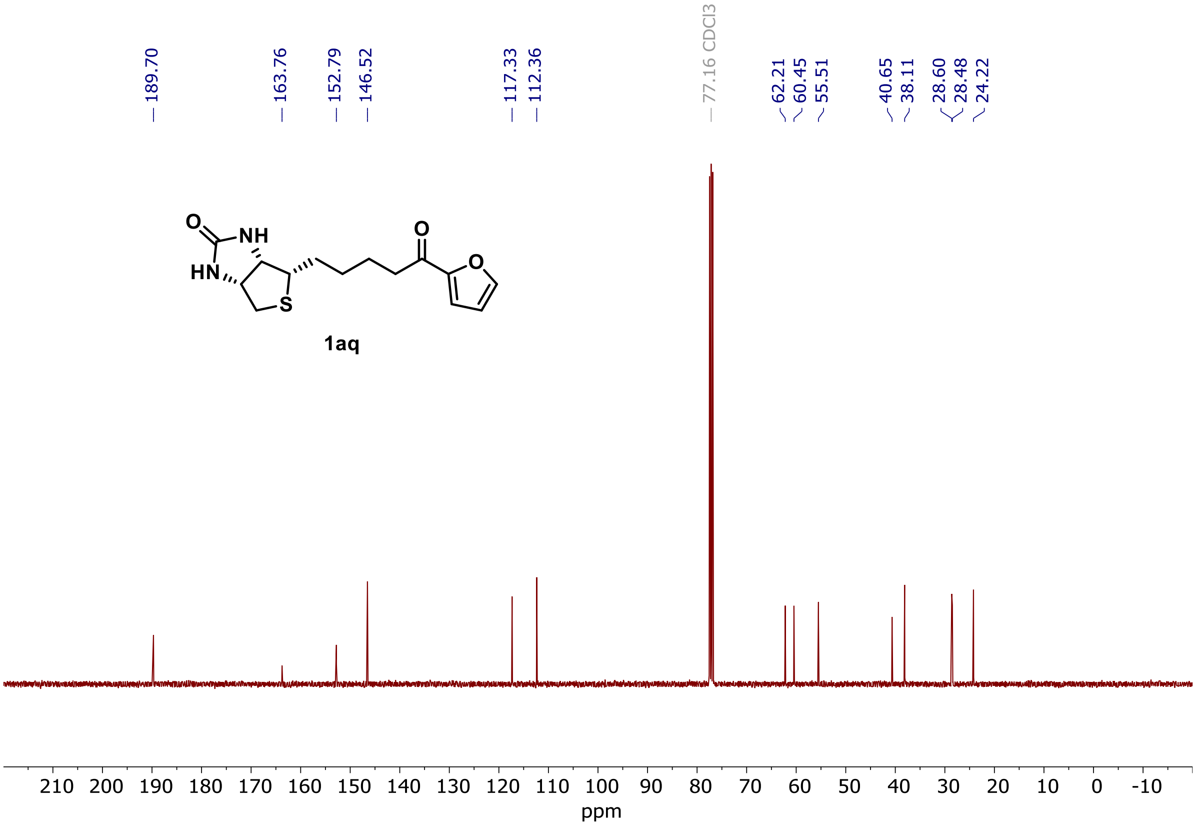

^{1}H NMR (400 MHz, $CDCl_3$) of **1aw**

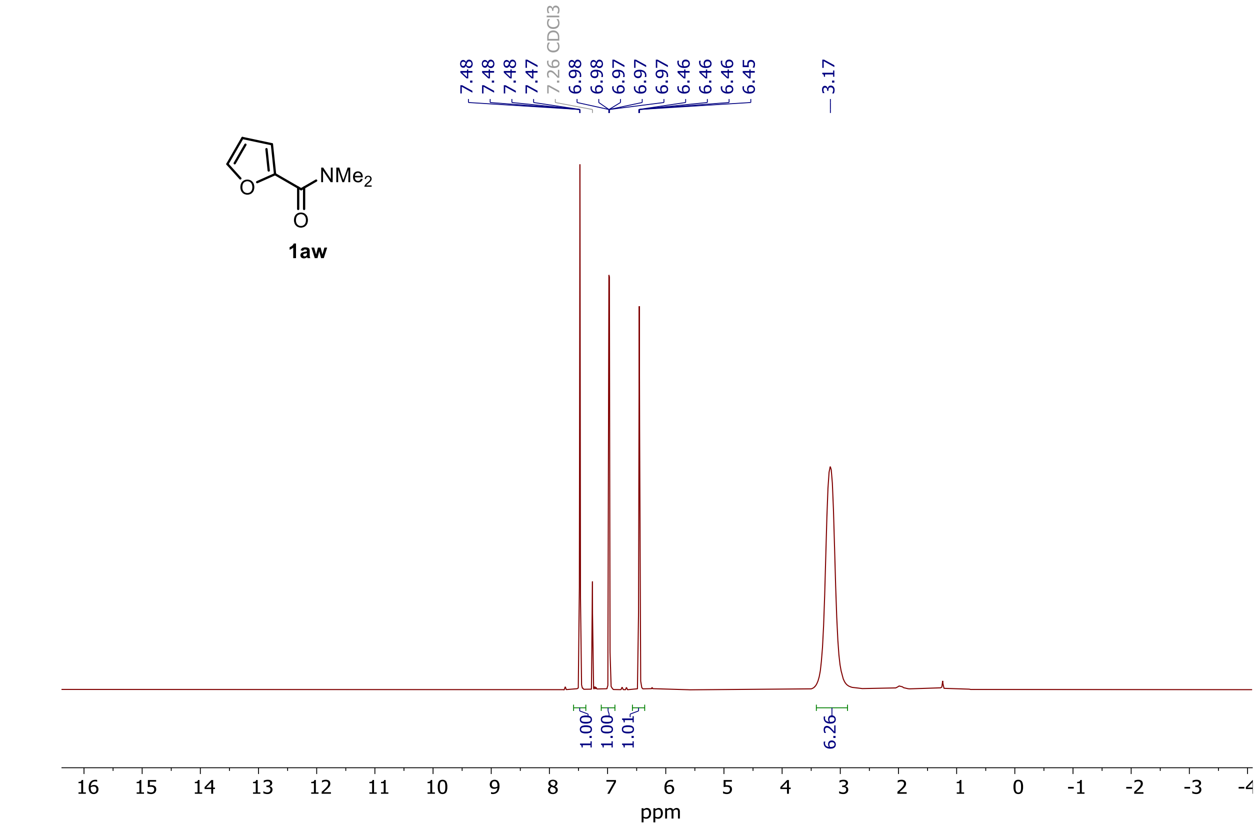

^{1}H NMR (400 MHz, $CDCl_3$) of **1ba**

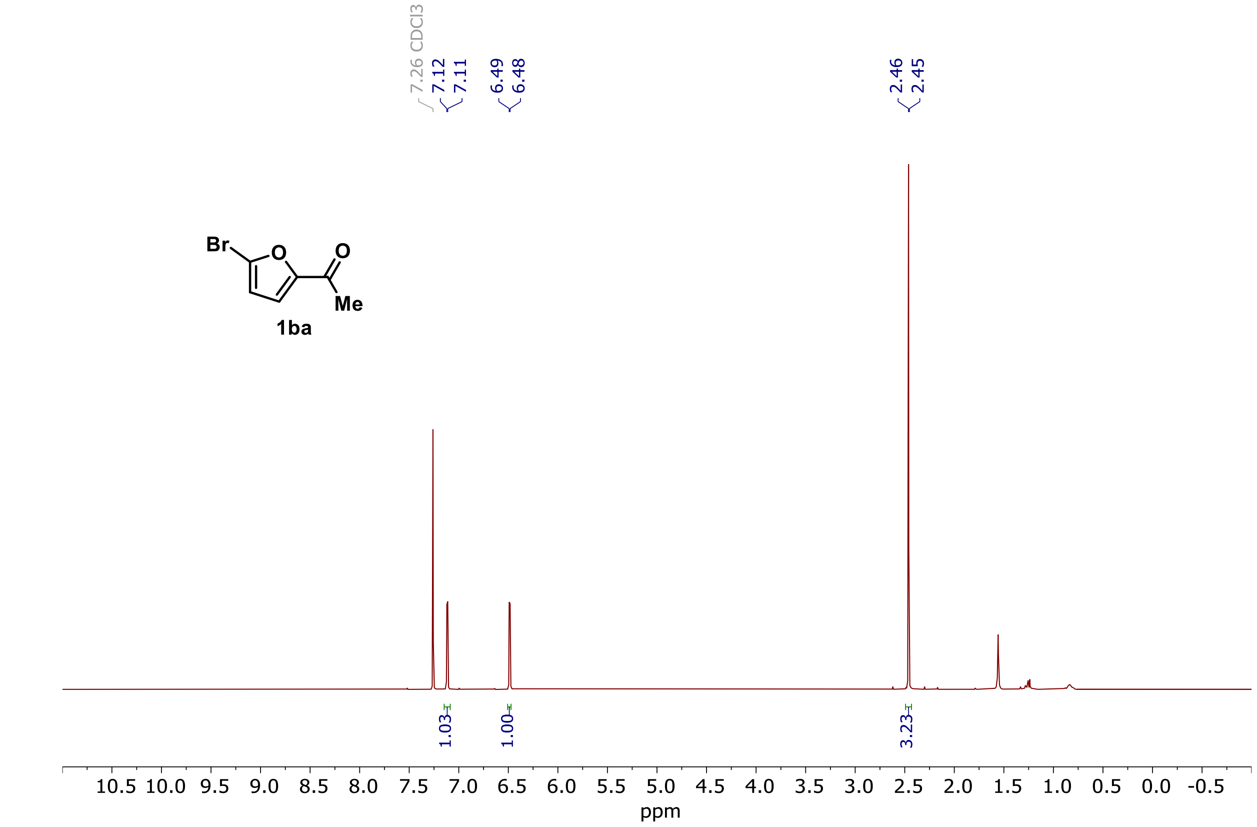

1H NMR (400 MHz, CDCl3) of **1bb**

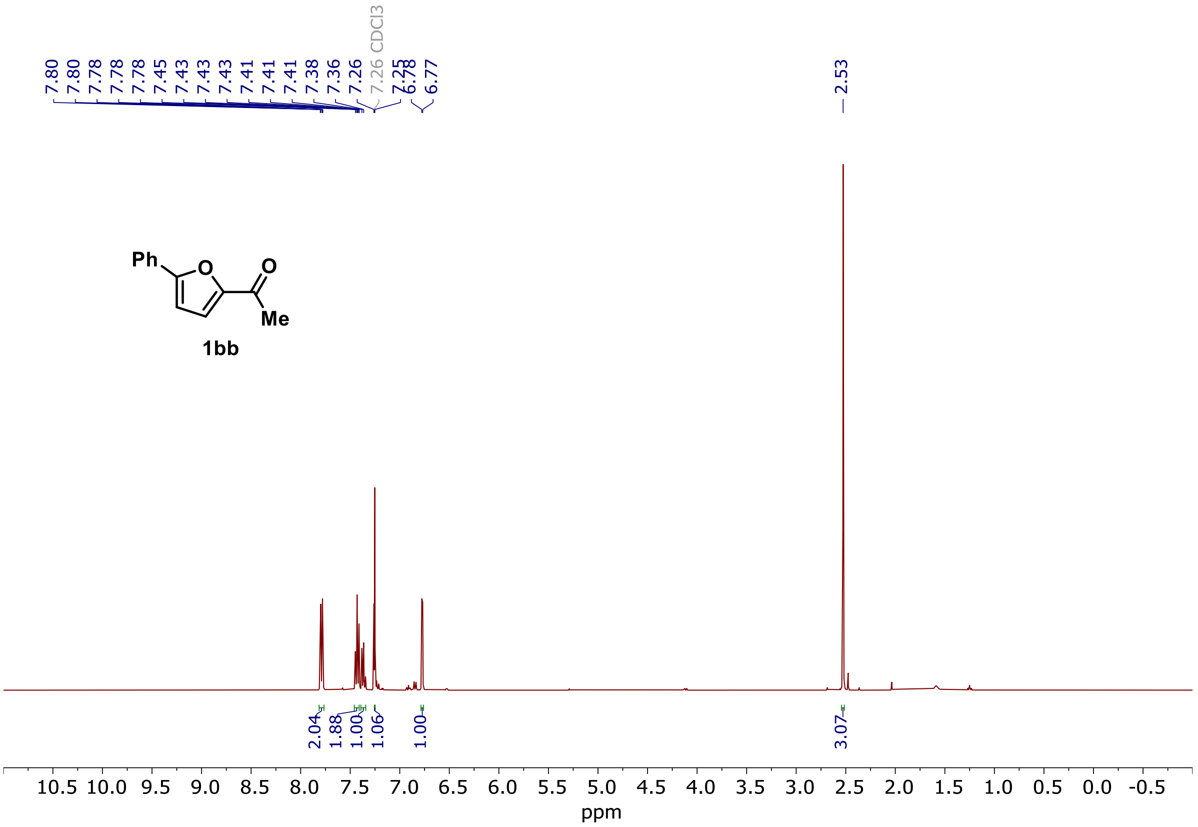

^{1}H NMR (400 MHz, $CDCl_3$) of **1bc**

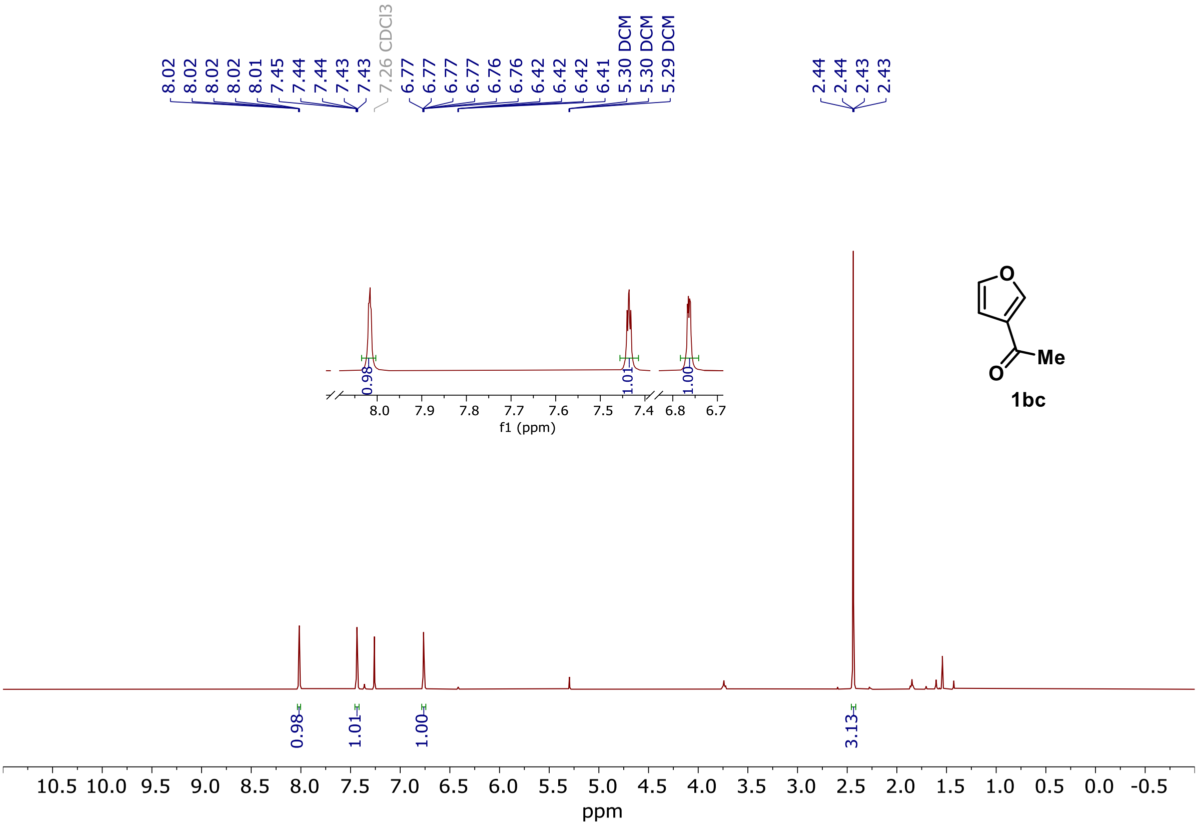

^{1}H NMR (400 MHz, $CDCl_3$) of **2d**

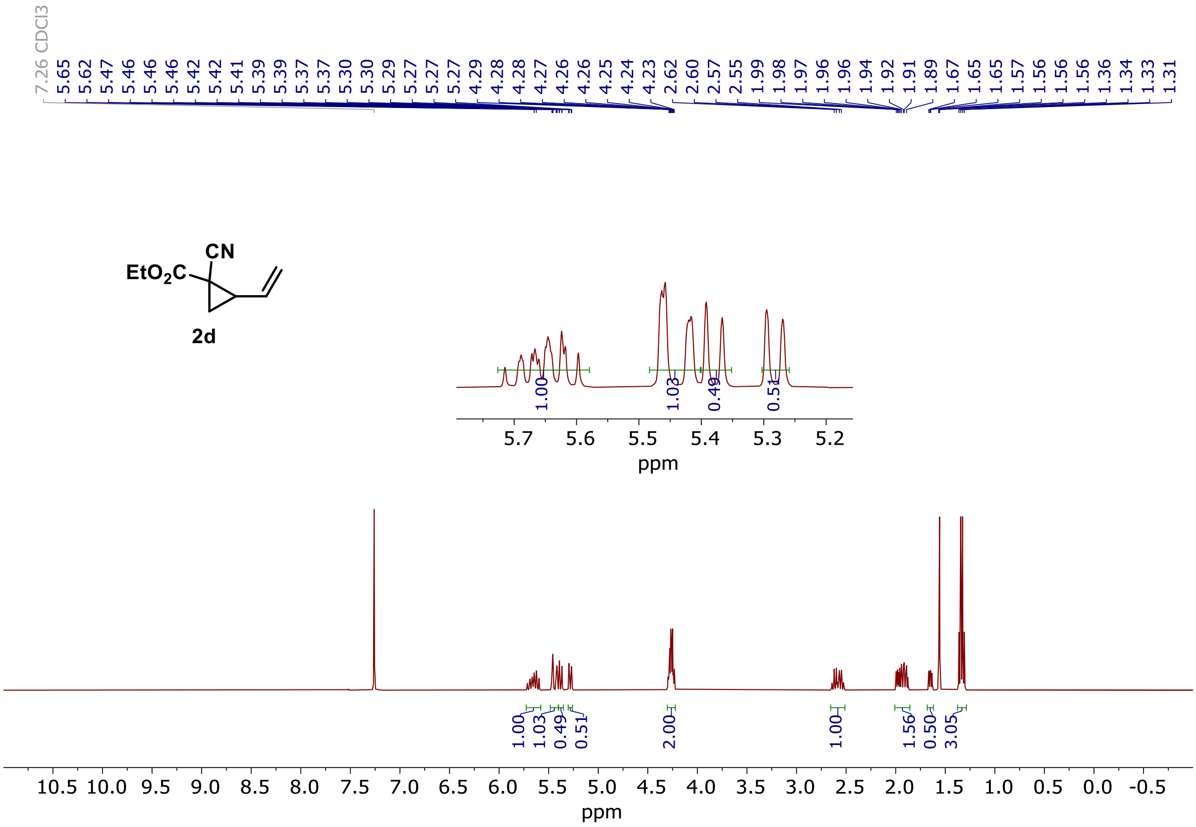

^{13}C NMR (101 MHz, $CDCl_3$) of **2d**

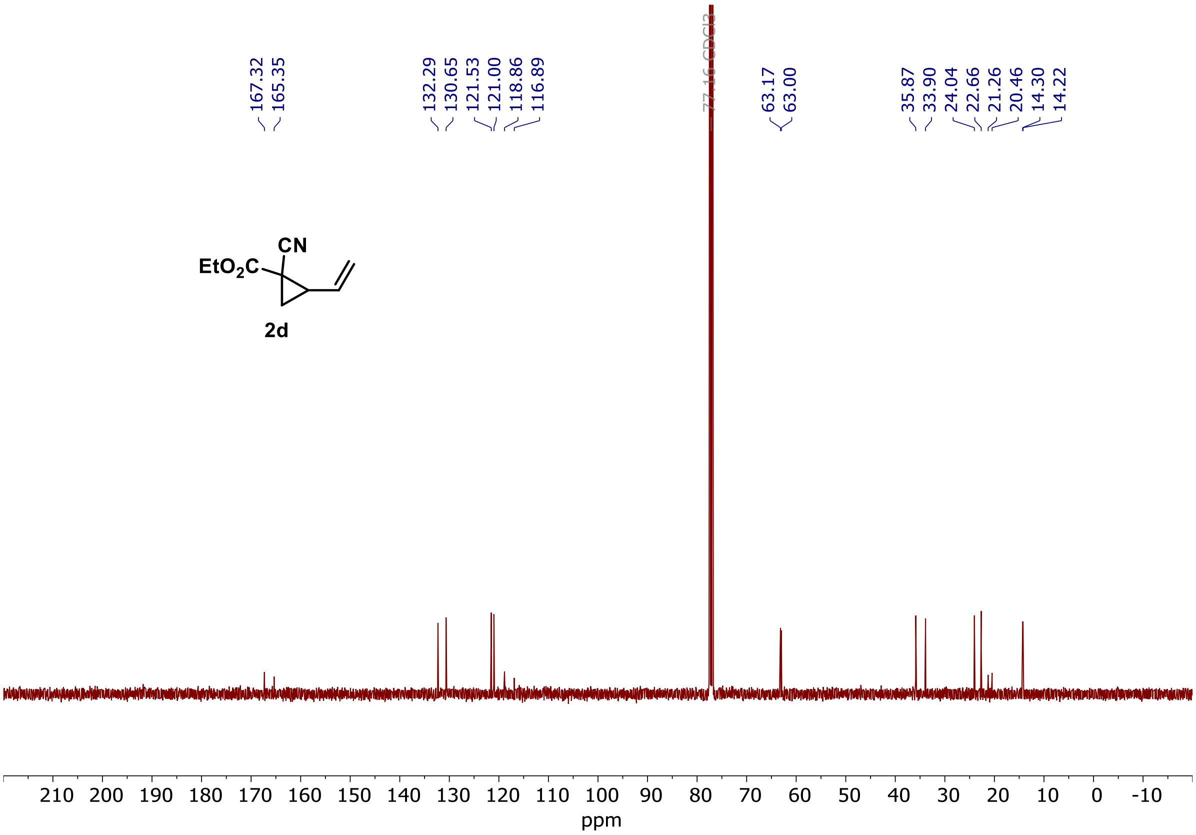

^{1}H NMR (400 MHz, $CDCl_3$) of **2h**

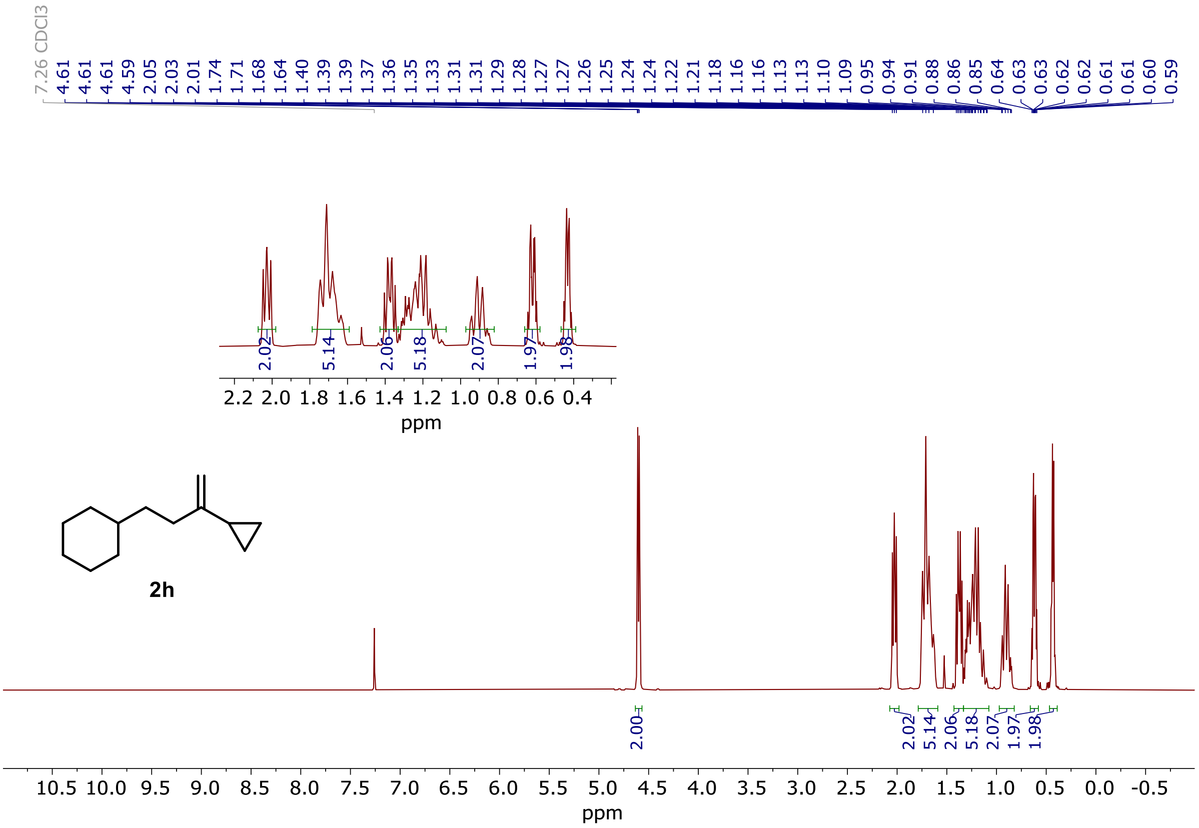

^{13}C NMR (101 MHz, $CDCl_3$) of **2h**

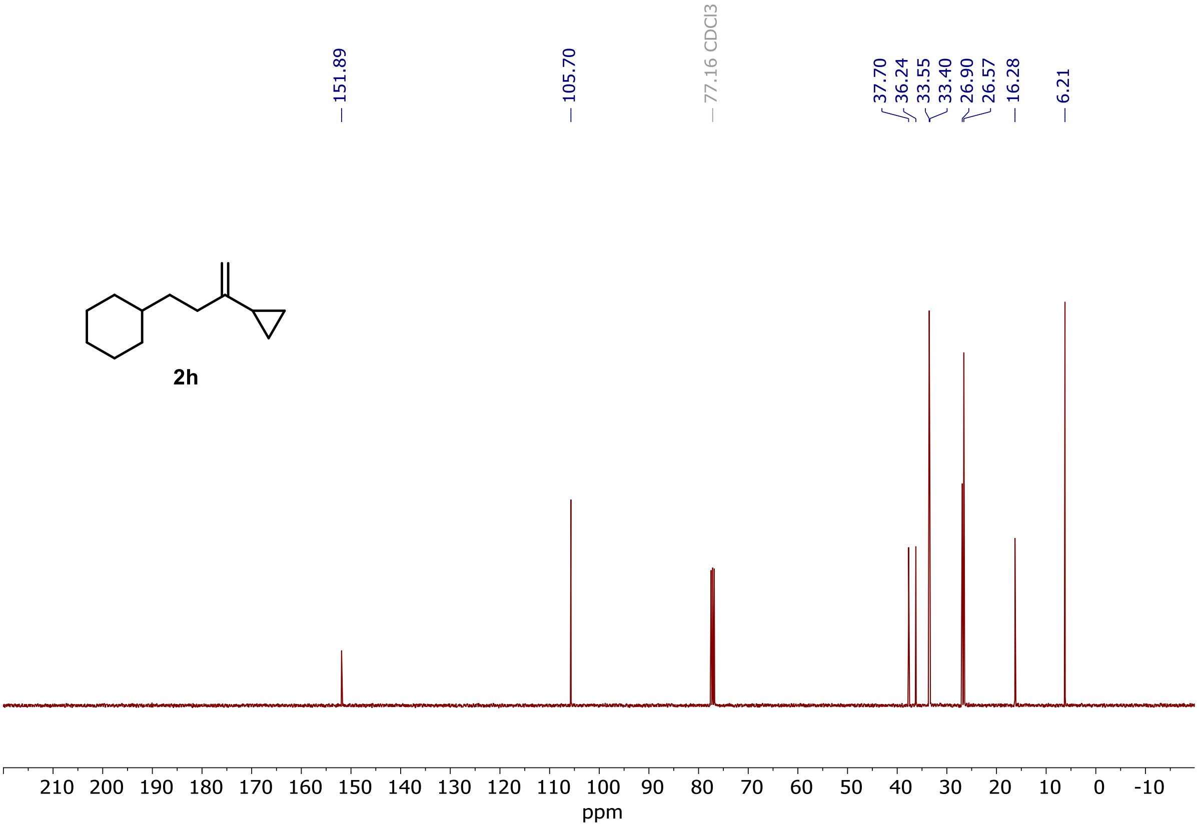

^{1}H NMR (400 MHz, $CDCl_3$) of **2i**

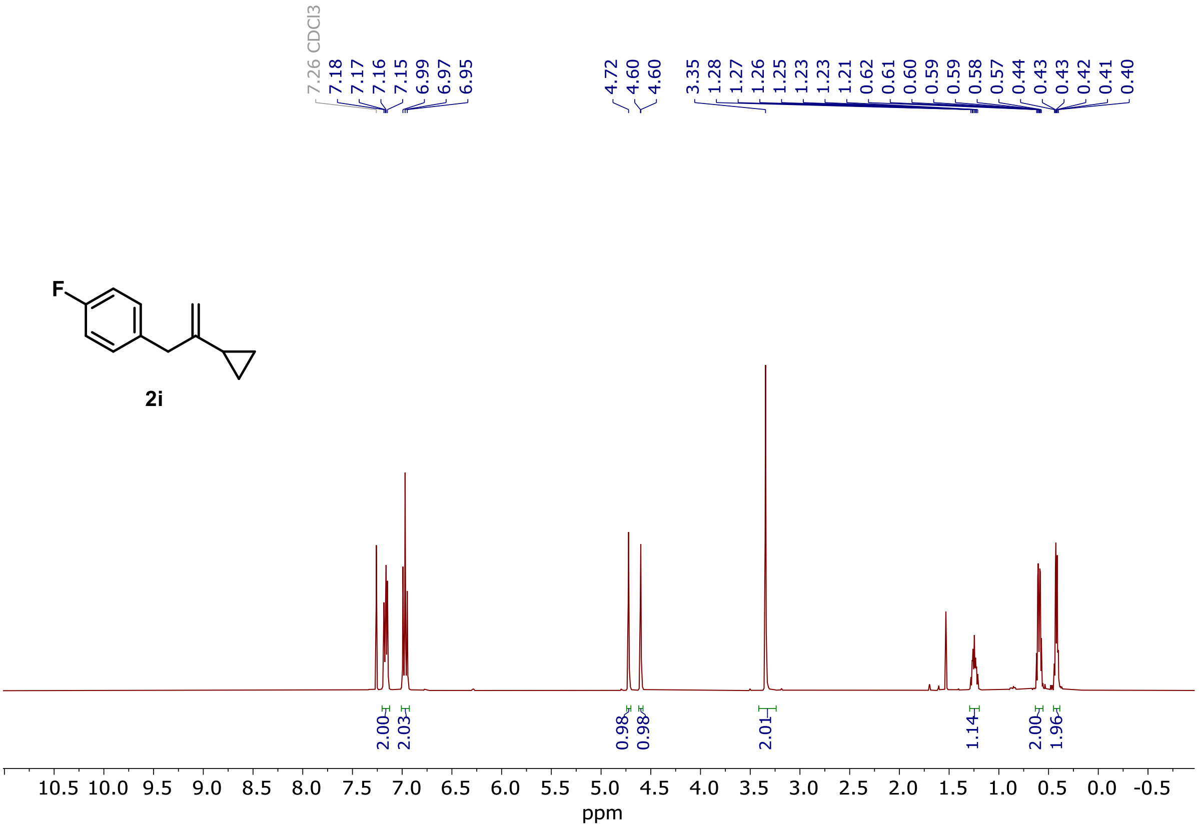

$^{13}C\{^{19}F\}$ NMR (126 MHz, $CDCl_3$) of **2i**

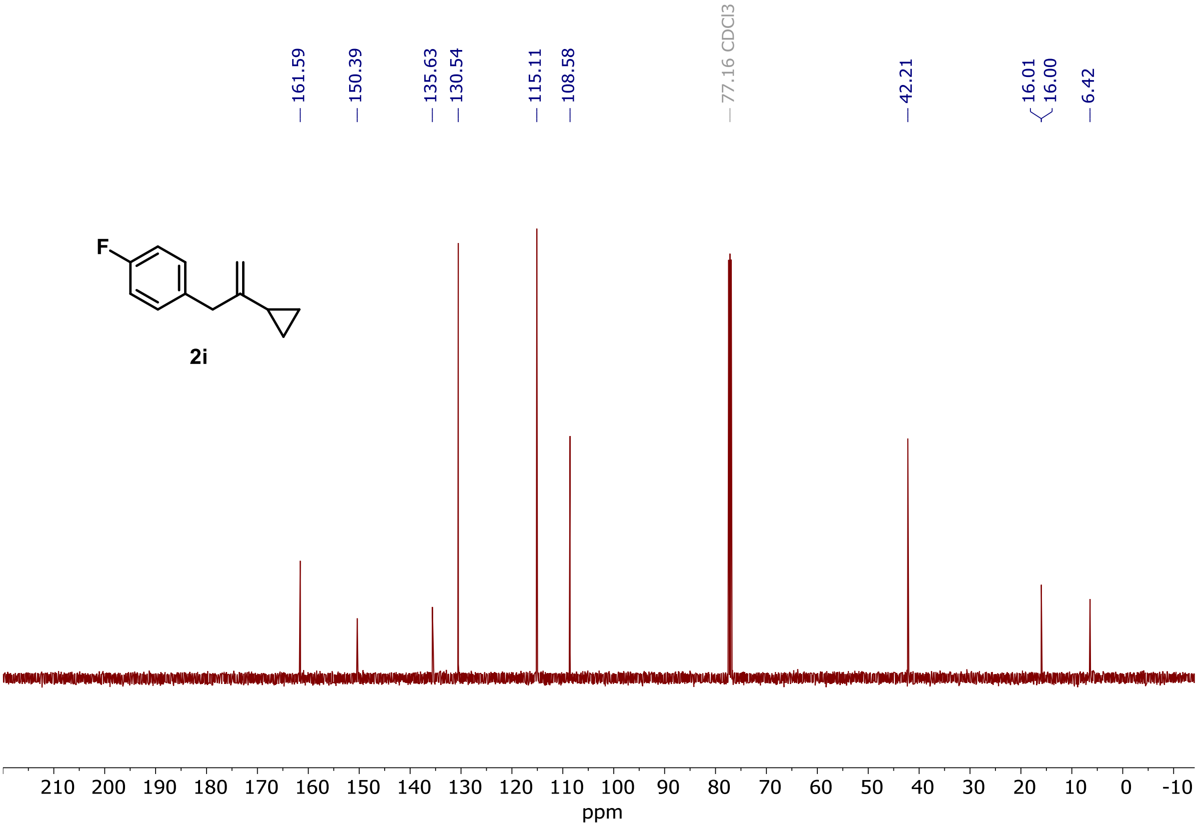

^{13}C NMR (126 MHz, $CDCl_3$) of **2i**

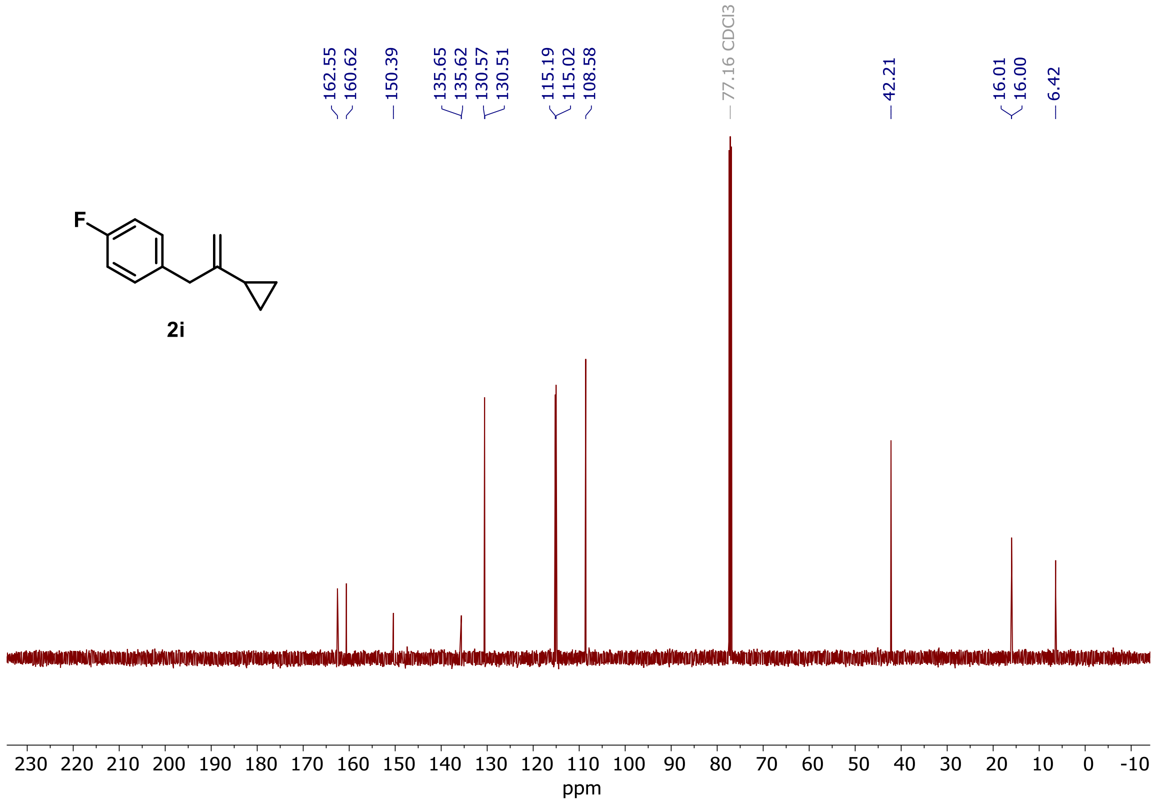

^{19}F NMR (376 MHz, $CDCl_3$) of **2i**

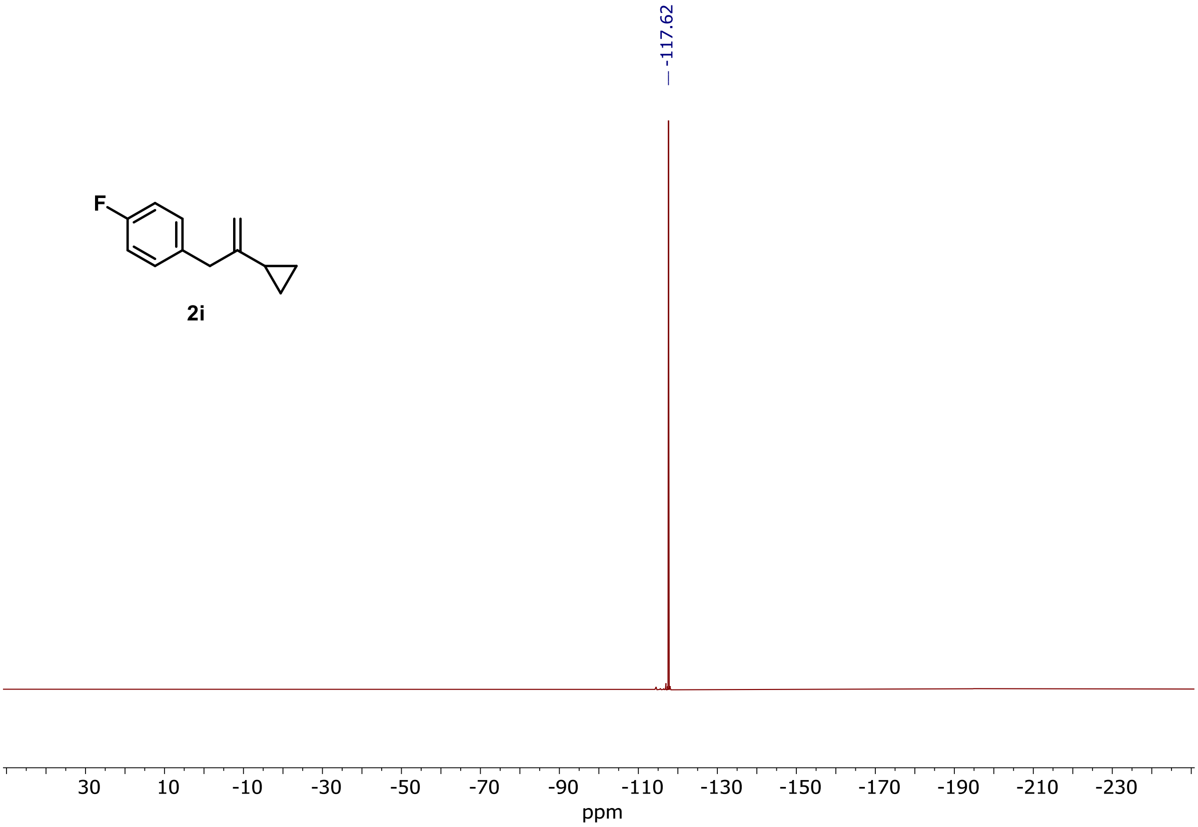

^{1}H NMR (400 MHz, $CDCl_3$) of **2j**

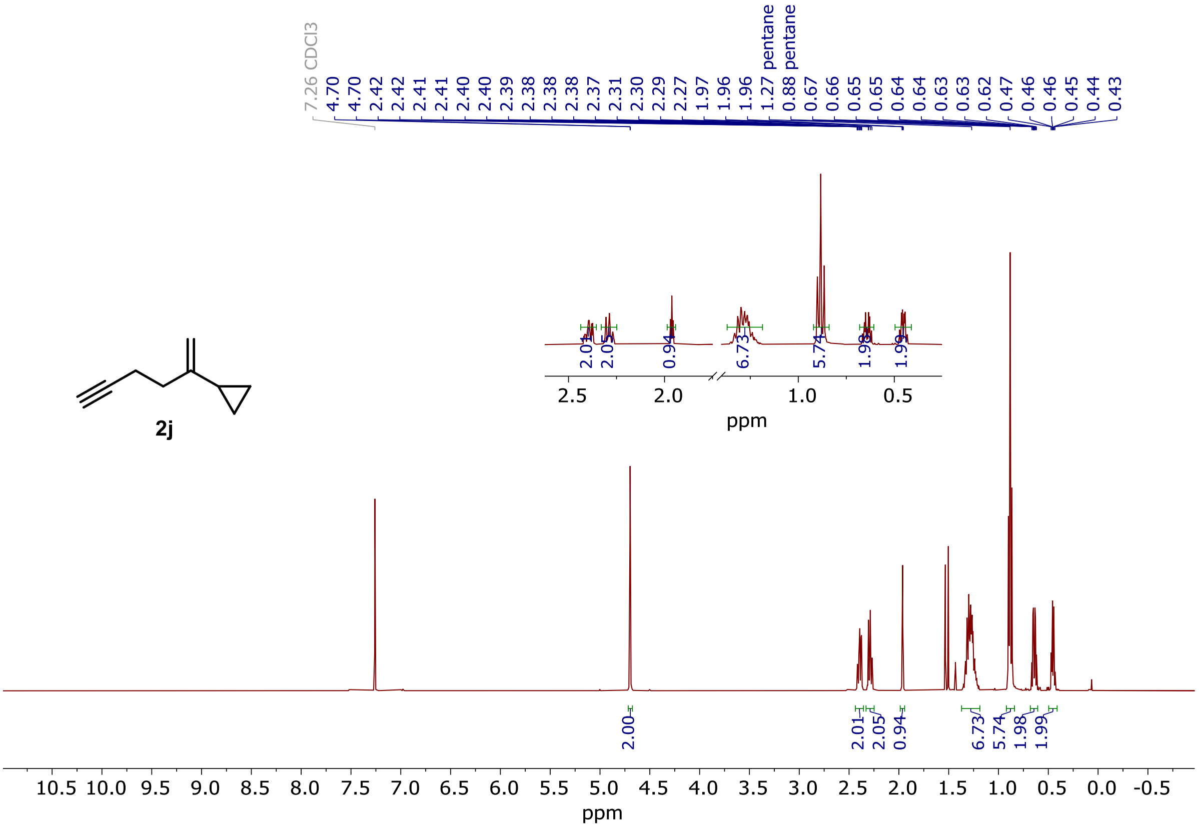

^{13}C NMR (101 MHz, $CDCl_3$) of **2j**

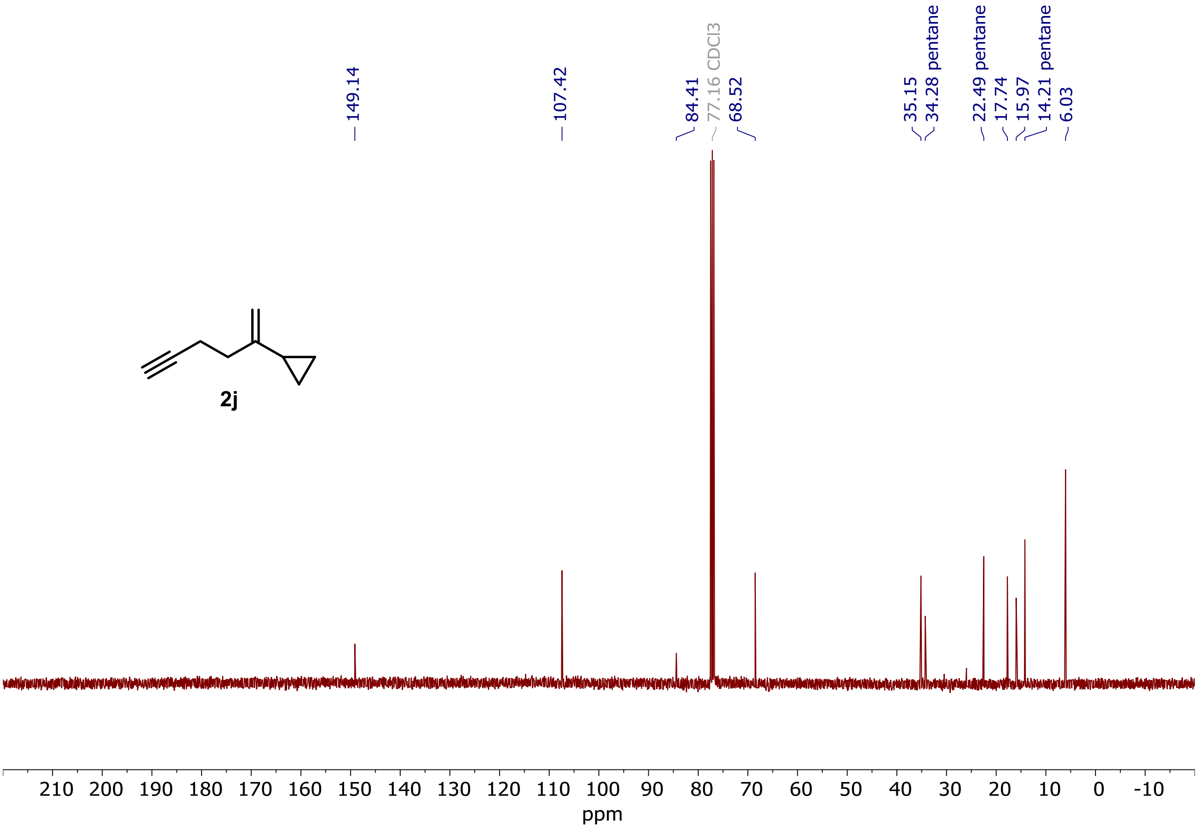

^{1}H NMR (599 MHz, $CDCl_3$) of **3a**

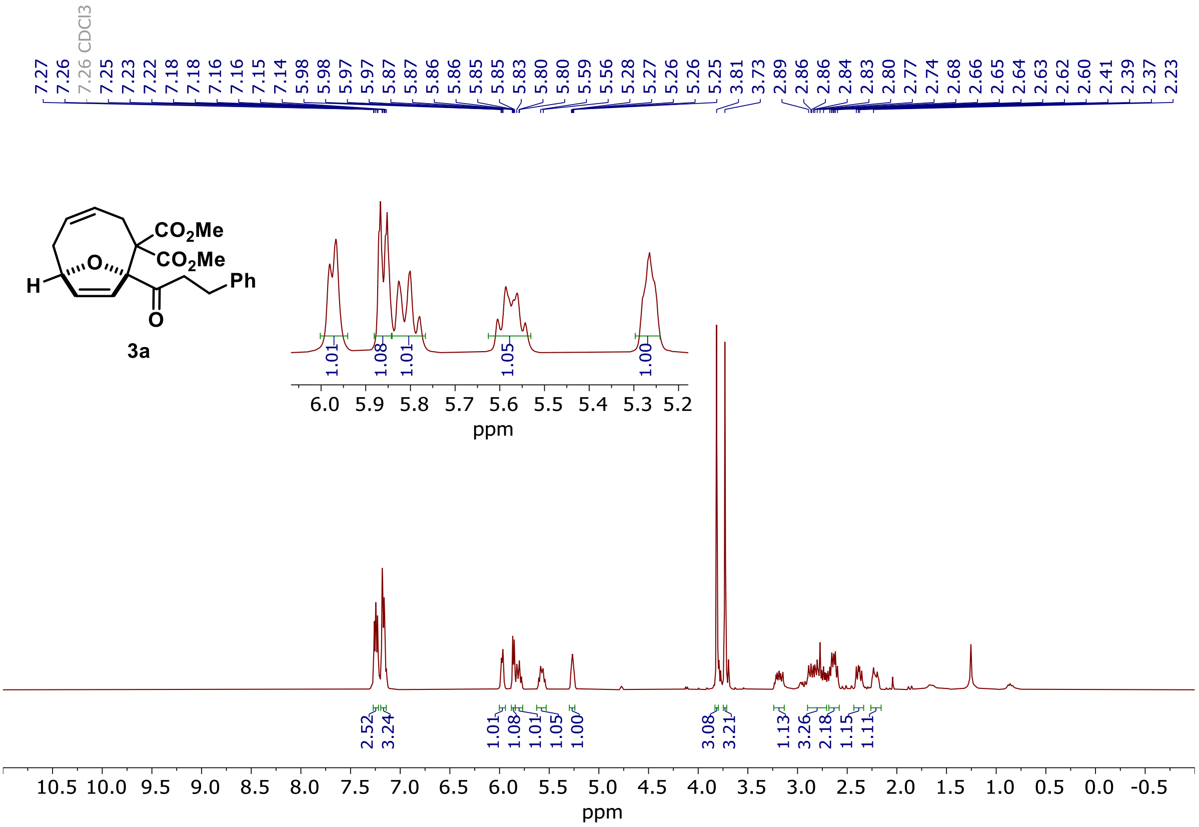

^{13}C NMR (101 MHz, $CDCl_3$) of **3a**

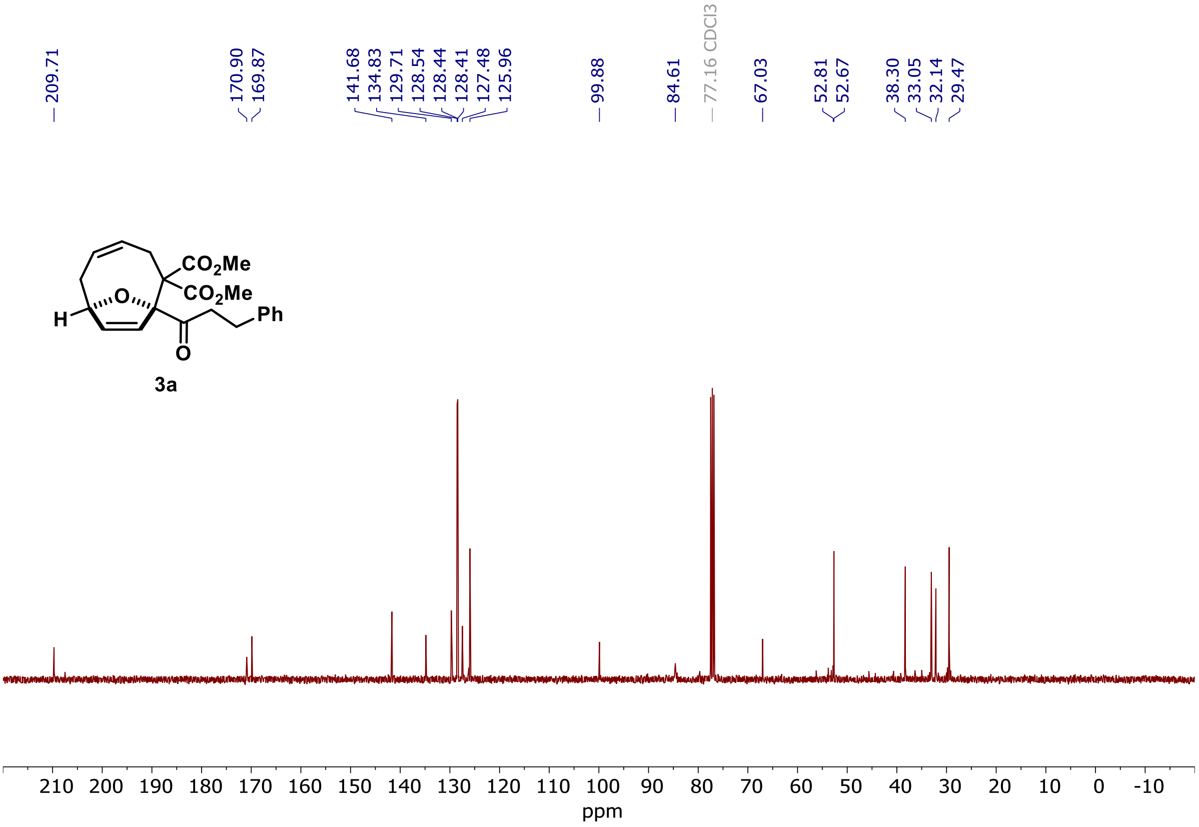

^{1}H NMR (599 MHz, $CDCl_3$) of **3a’**

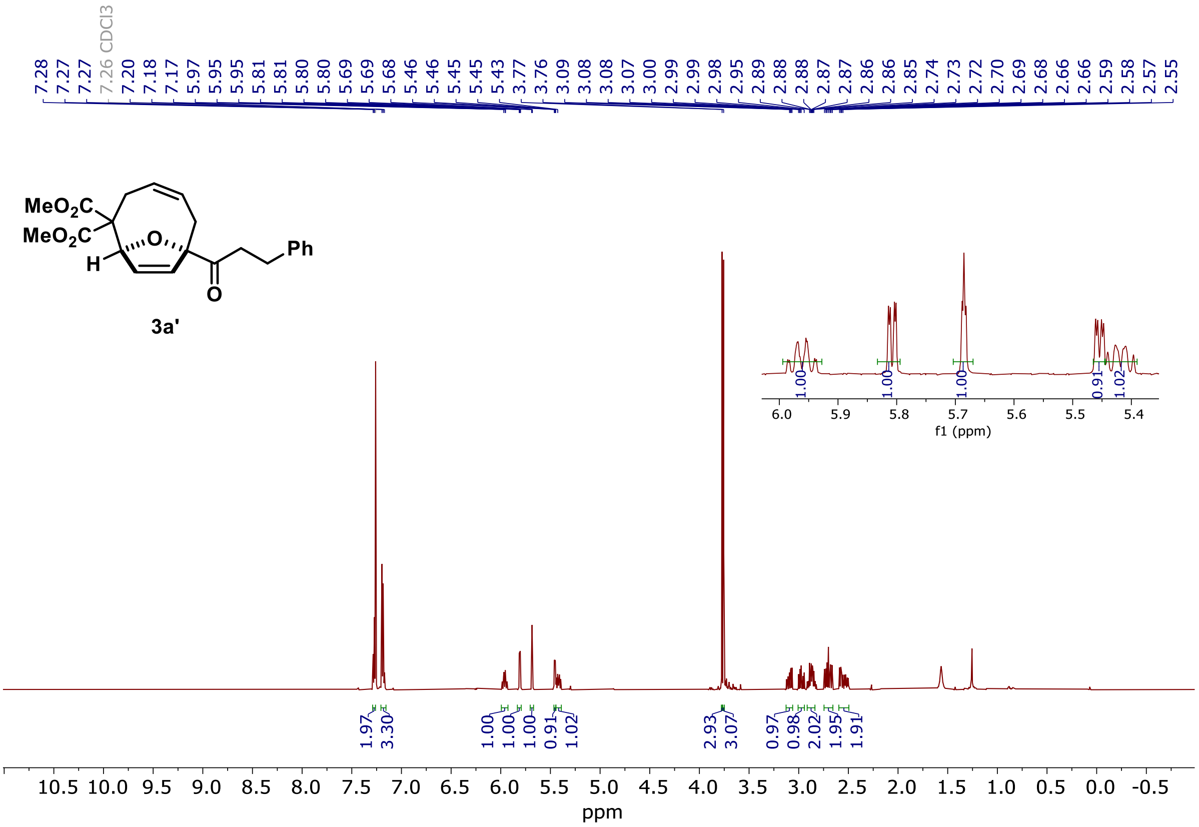

^{13}C NMR (151 MHz, $CDCl_3$) of **3a’**

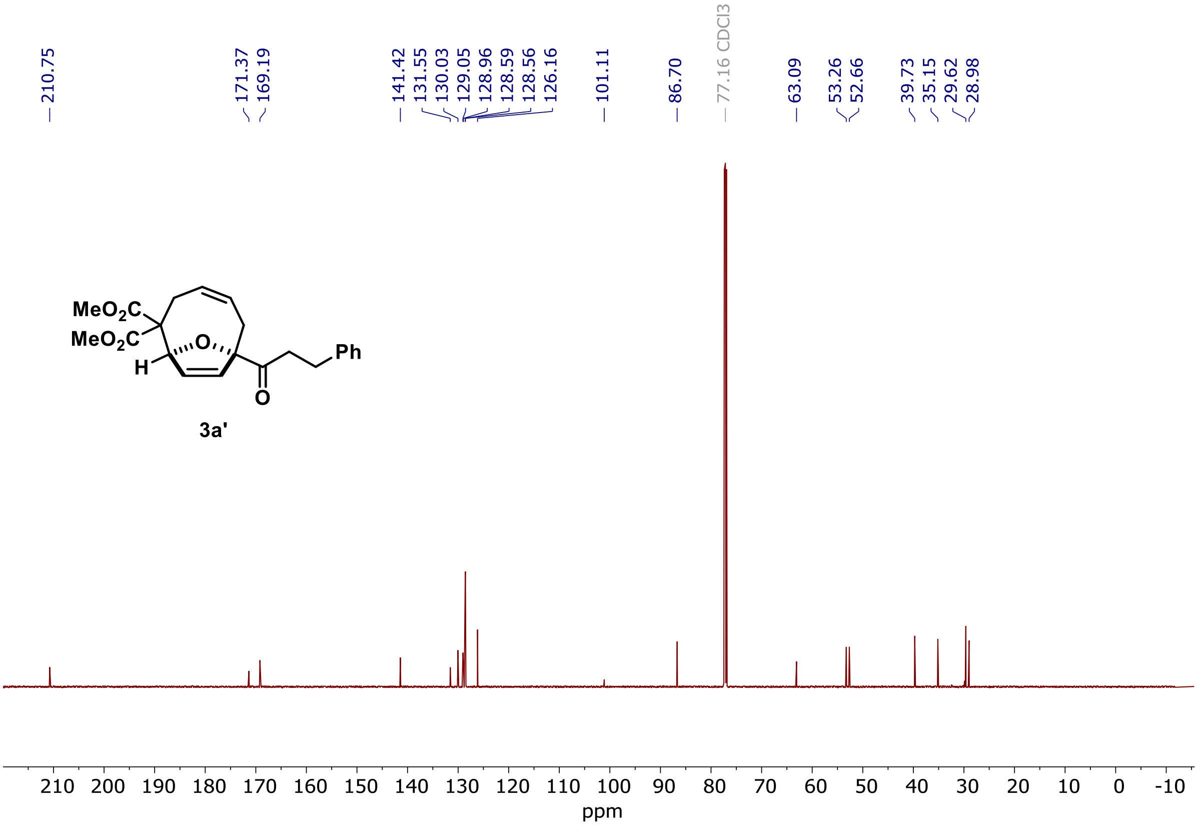

^{1}H NMR (400 MHz, $CDCl_3$) of **3b**

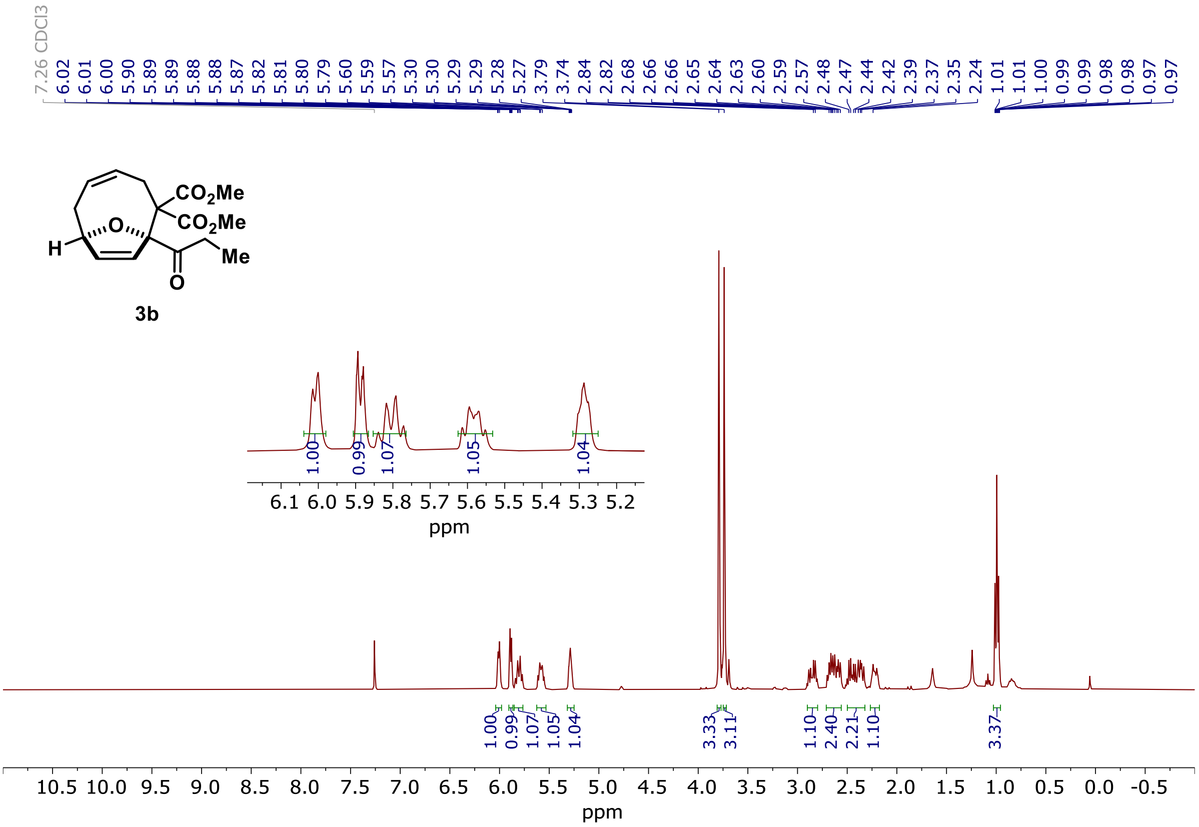

^{13}C NMR (101 MHz, $CDCl_3$) of **3b**

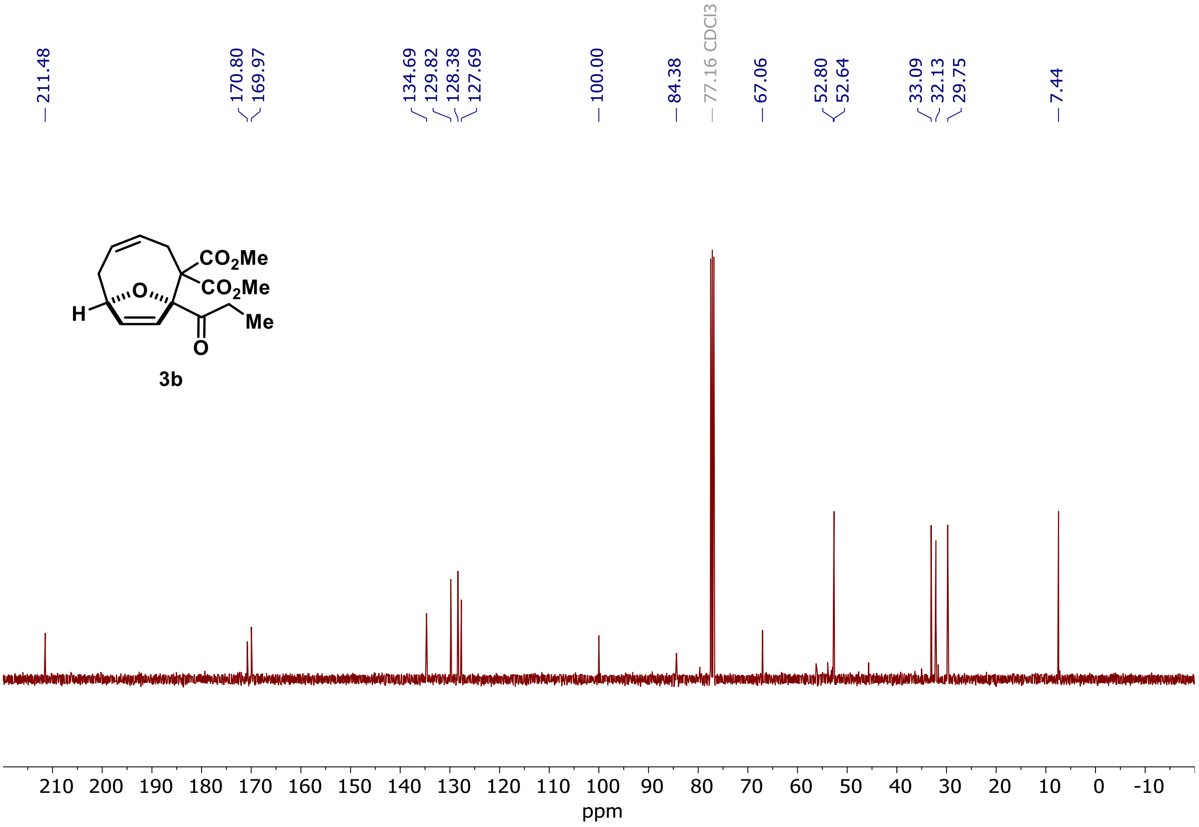

^{1}H NMR (400 MHz, CDCl3) of **3b'**

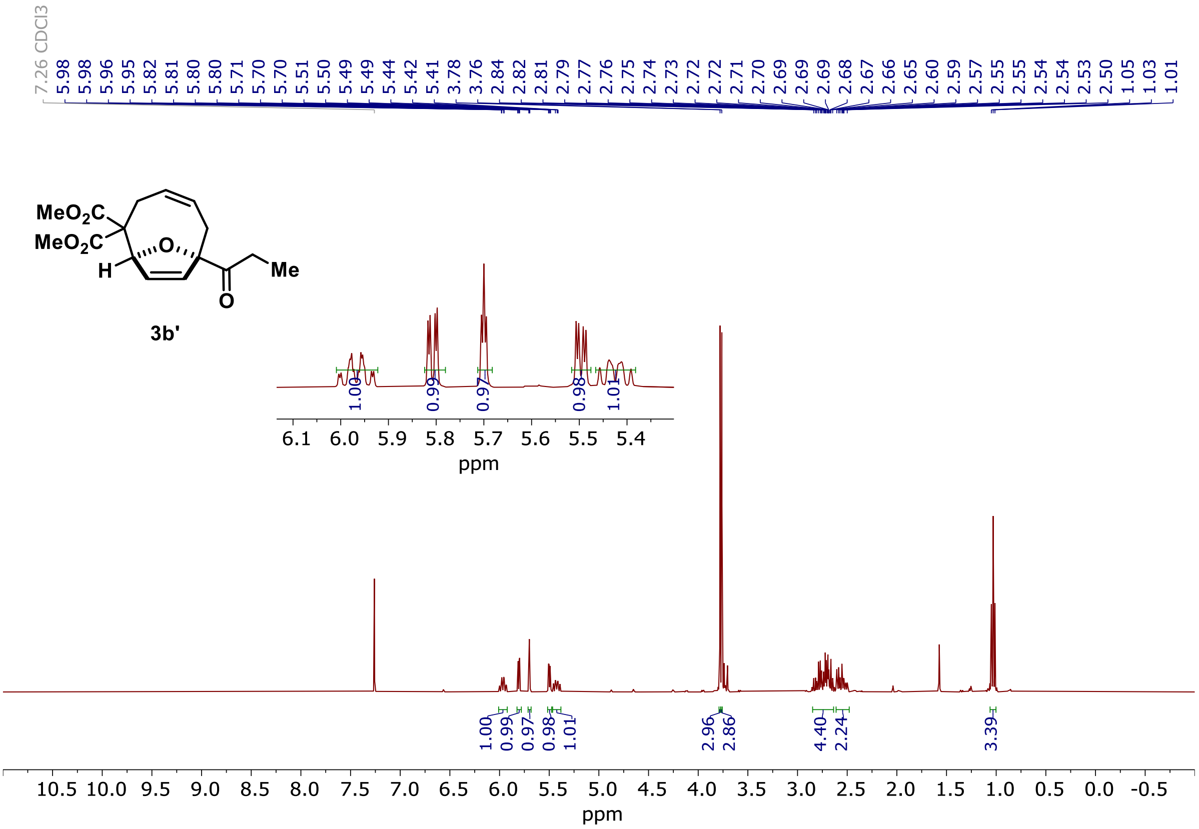

^{13}C NMR (101 MHz, CDCl3) of **3b'**

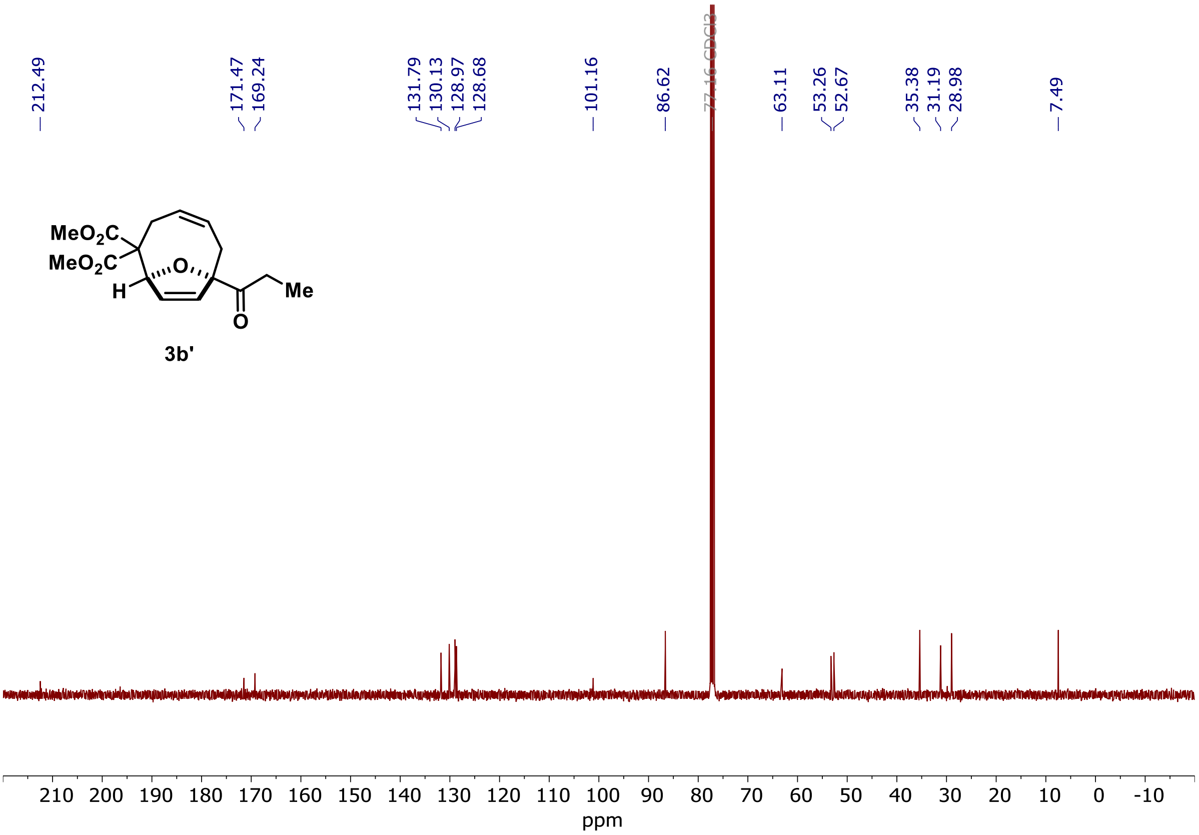

^{1}H NMR (400 MHz, $CDCl_3$) of **3c**

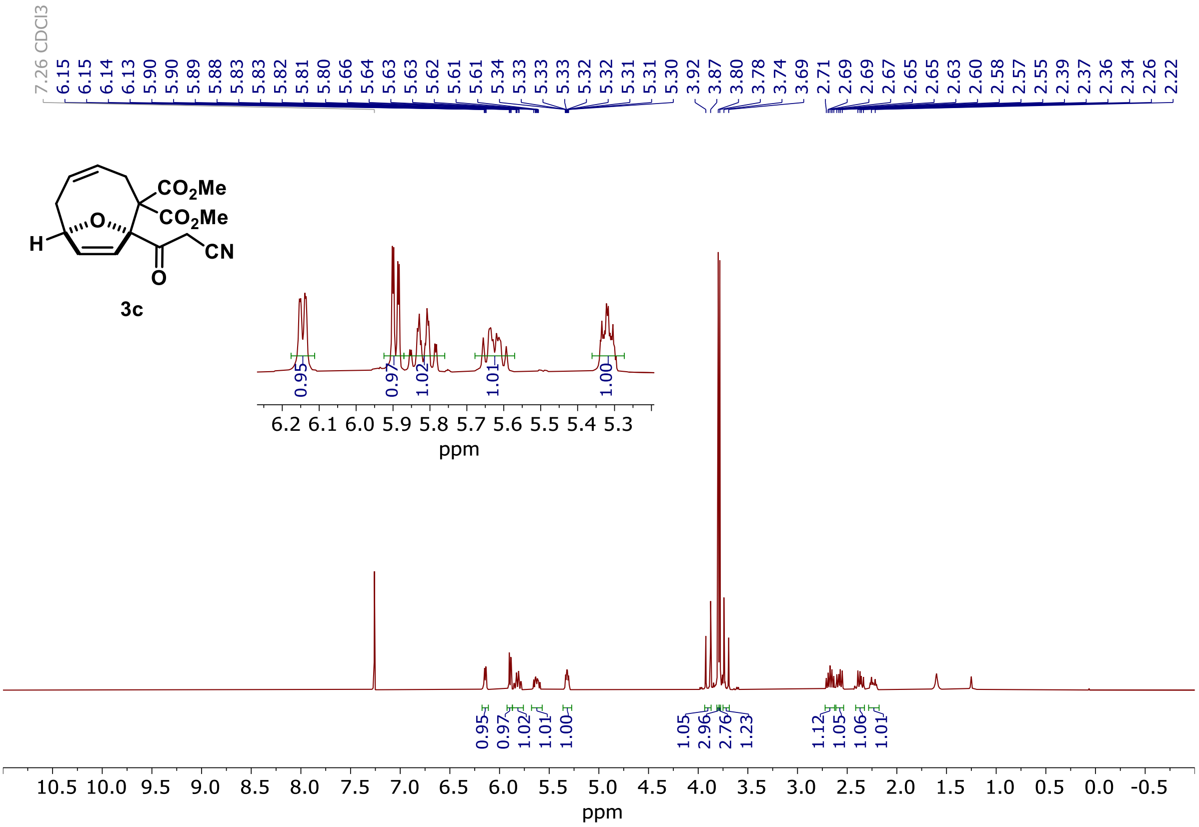

^{13}C NMR (101 MHz, $CDCl_3$) of **3c**

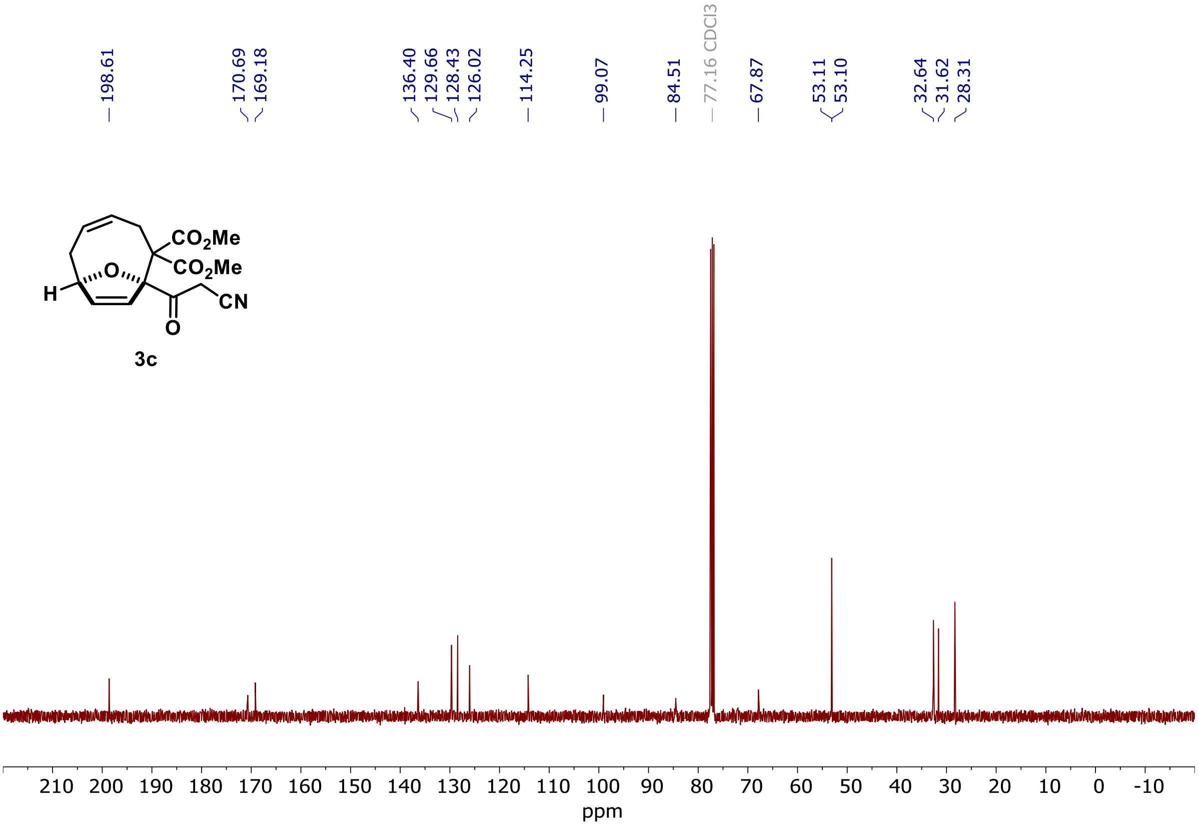

^{1}H NMR (400 MHz, $CDCl_3$) of **3d**

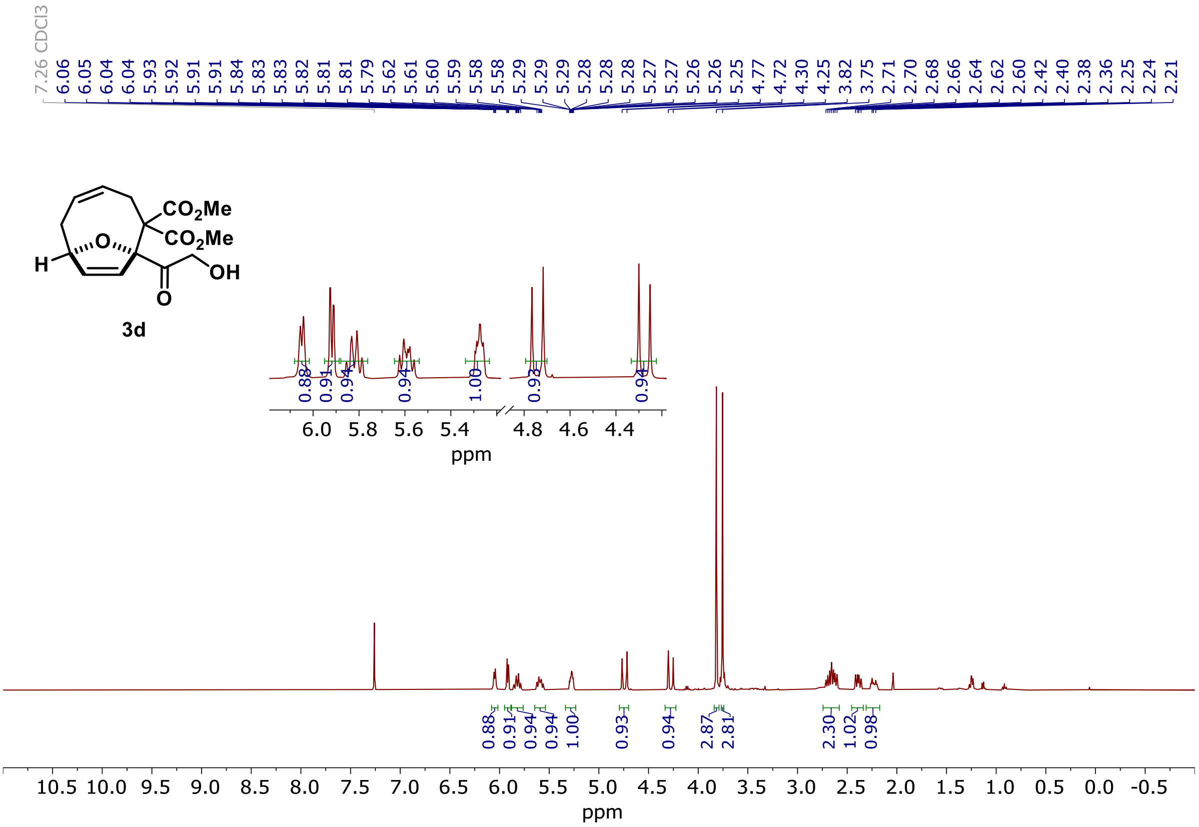

^{13}C NMR (101 MHz, $CDCl_3$) of **3d**

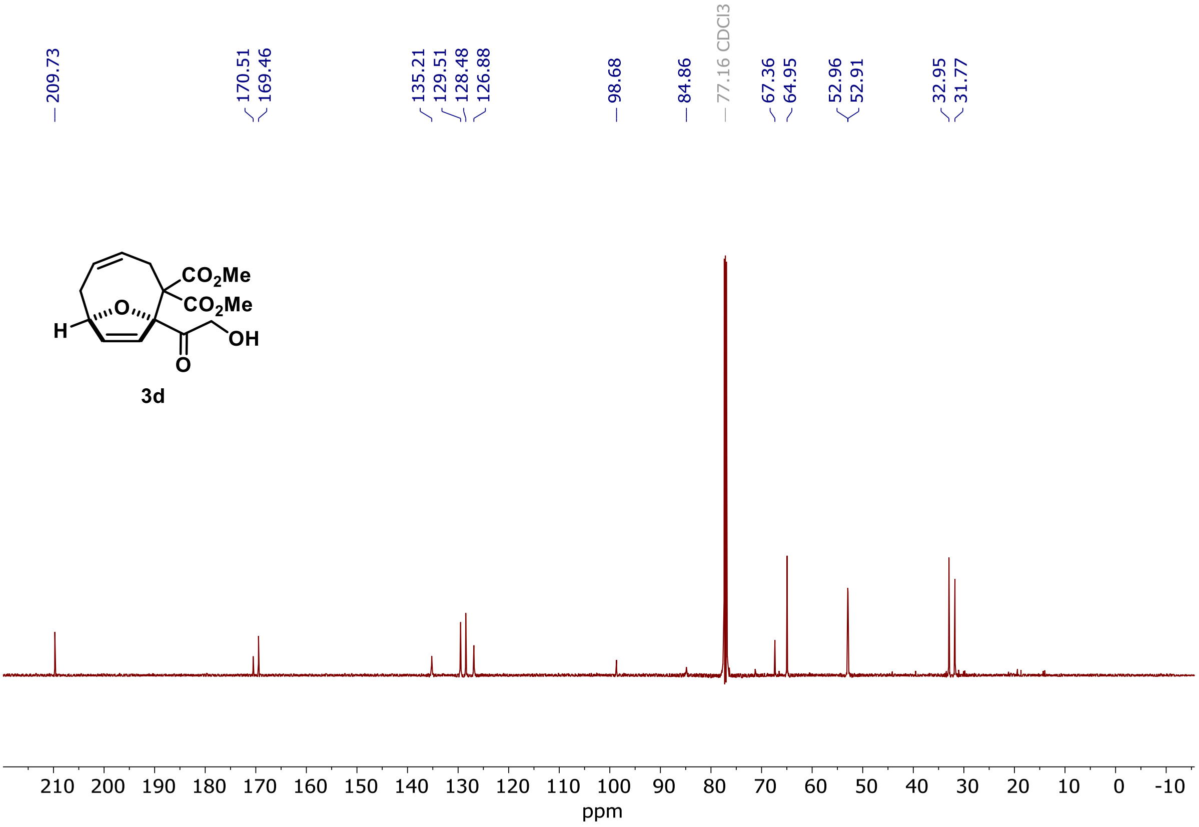

^{1}H NMR (599 MHz, $CDCl_3$) of **3d’**

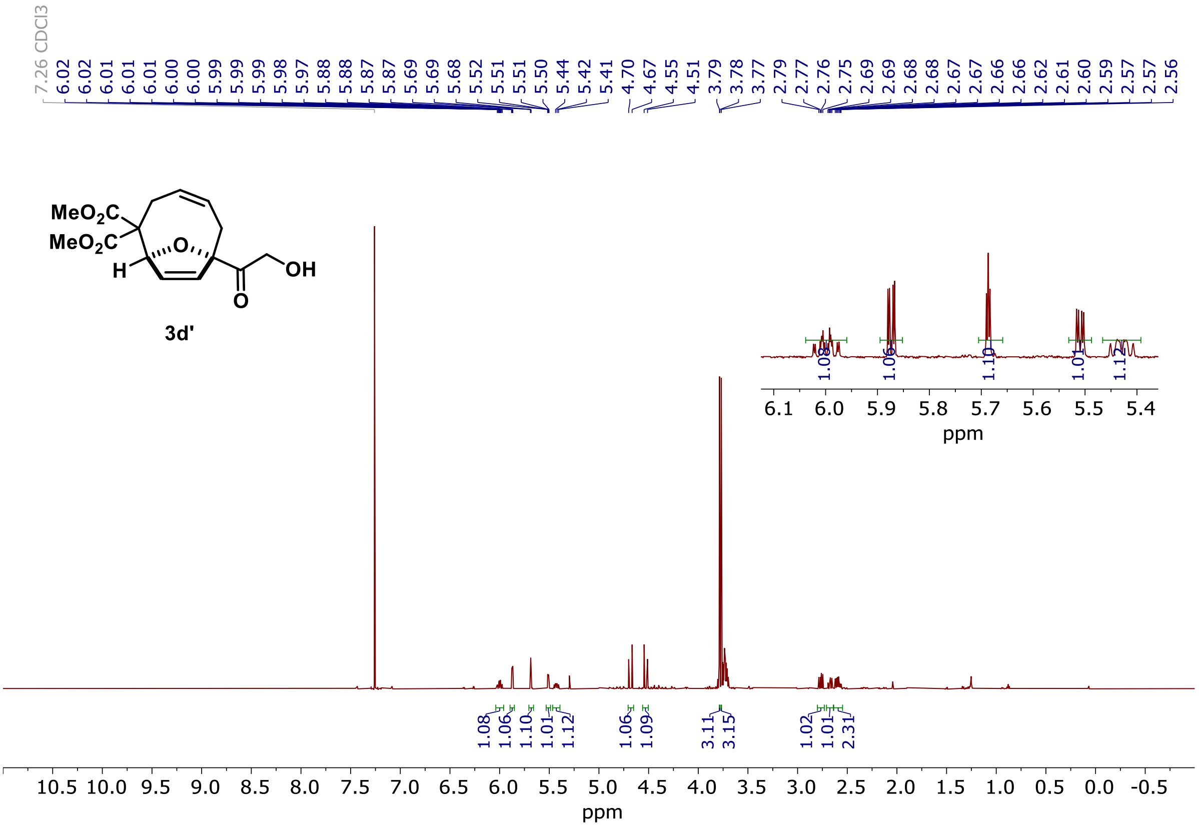

^{13}C NMR (151 MHz, $CDCl_3$) of **3d’**

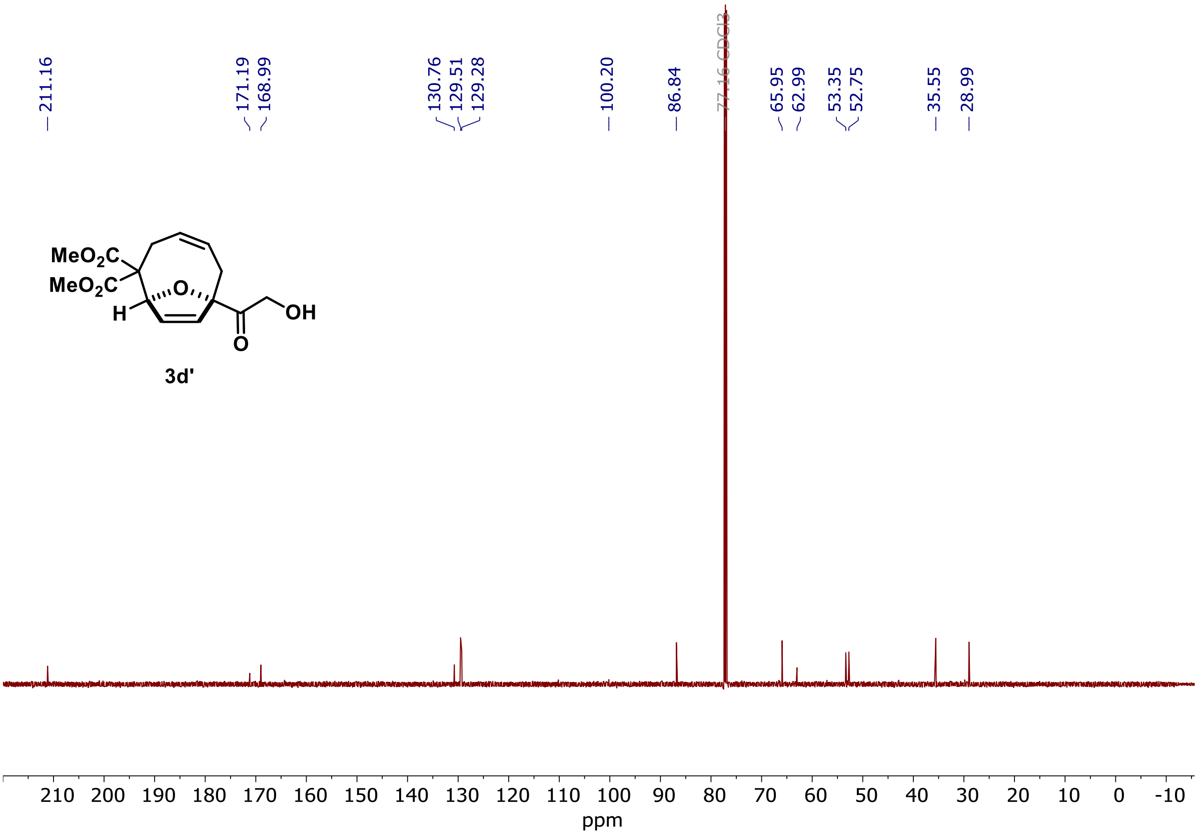

^{1}H NMR (599 MHz, $CDCl_3$) of **3e**

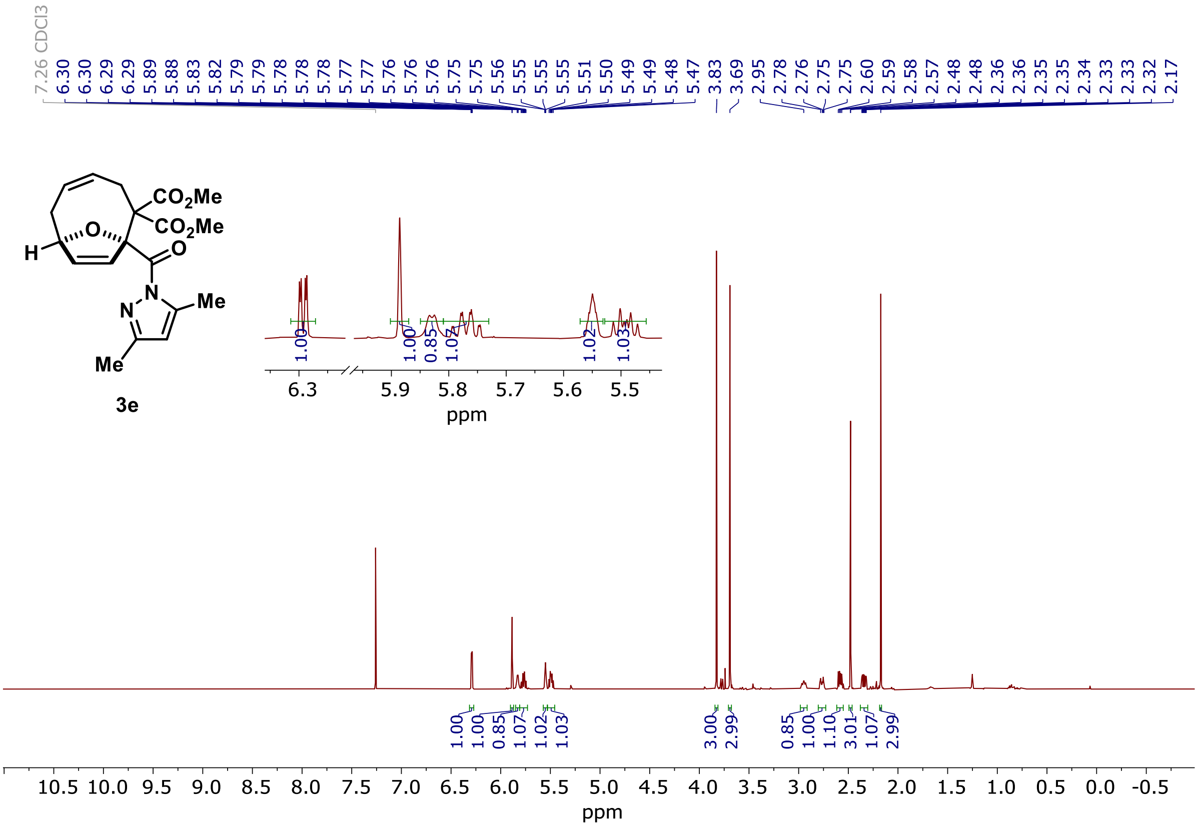

^{13}C NMR (151 MHz, $CDCl_3$) of **3e**

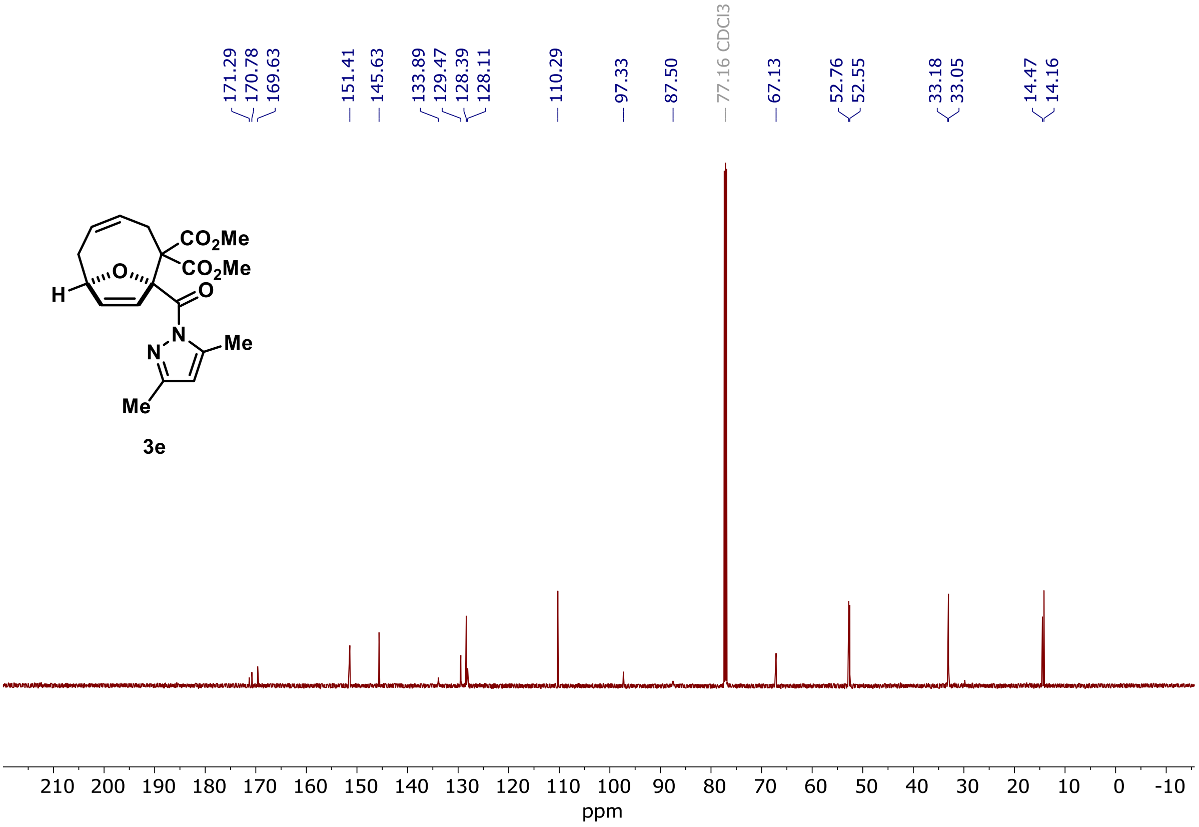

^{1}H NMR (599 MHz, $CDCl_3$) of **3f**

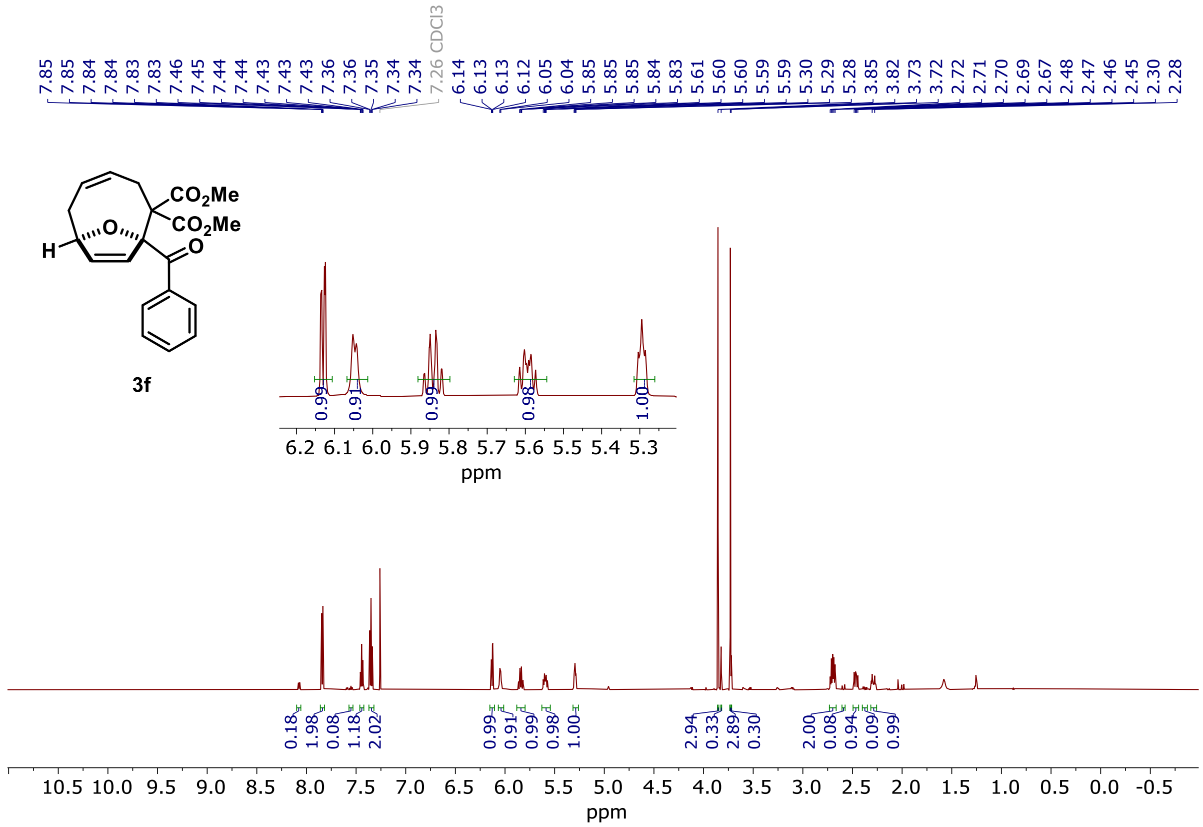

^{13}C NMR (101 MHz, $CDCl_3$) of **3f**

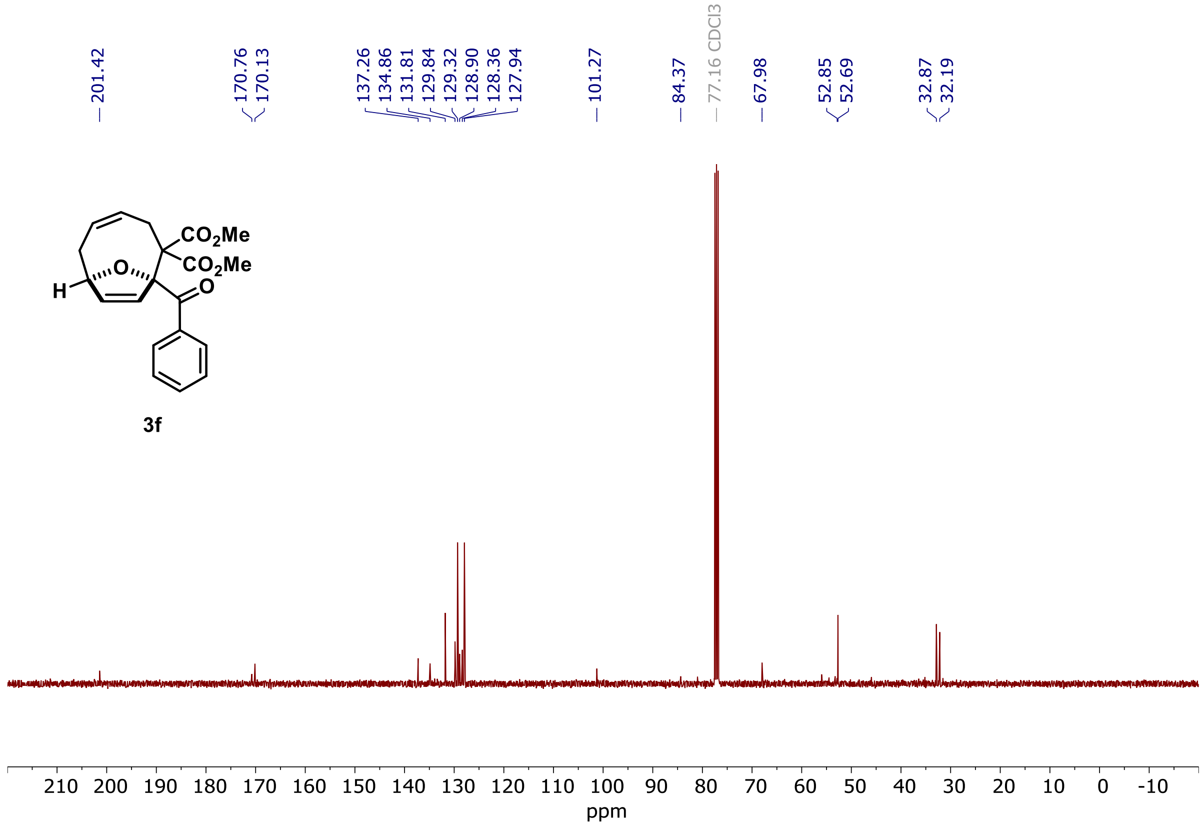

^{1}H NMR (400 MHz, $CDCl_3$) of **3g**

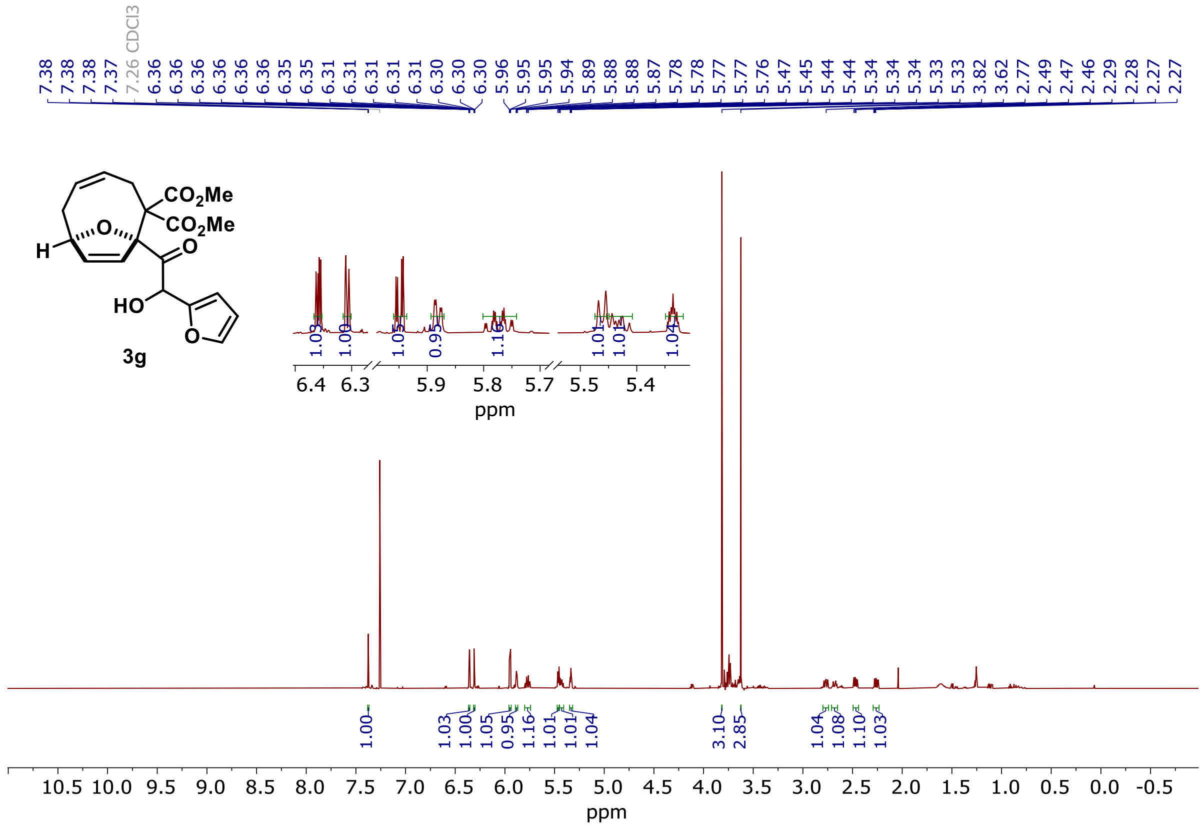

^{13}C NMR (101 MHz, $CDCl_3$) of **3g**

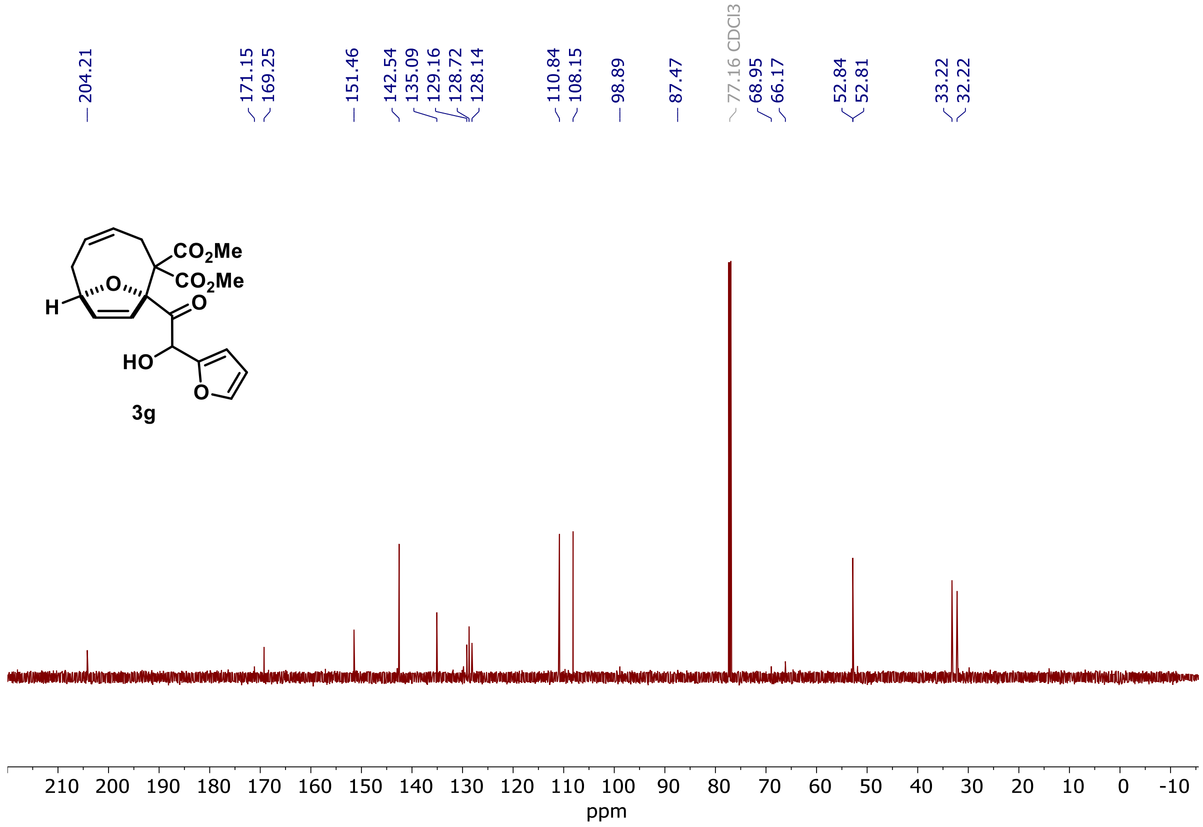

^{1}H NMR (599 MHz, $CDCl_3$) of **3g’**

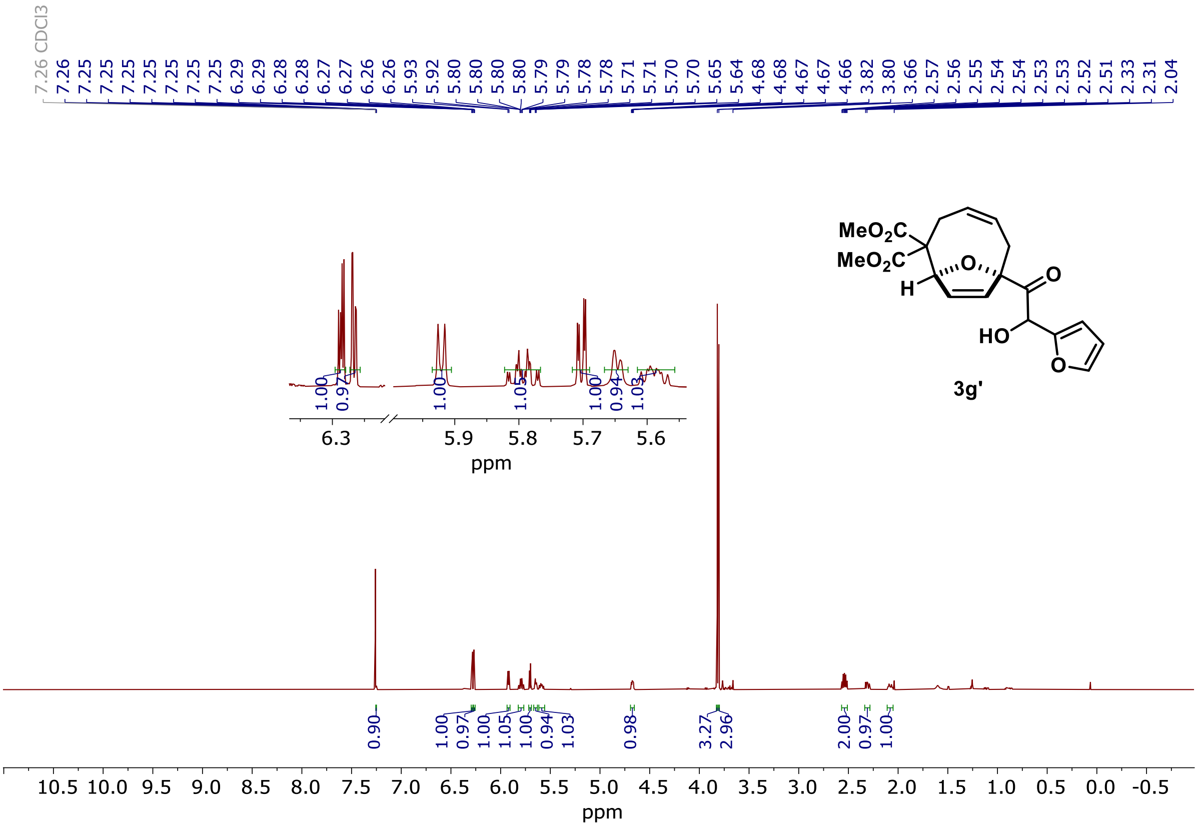

^{13}C NMR (151 MHz, CDCl3) of **3g’**

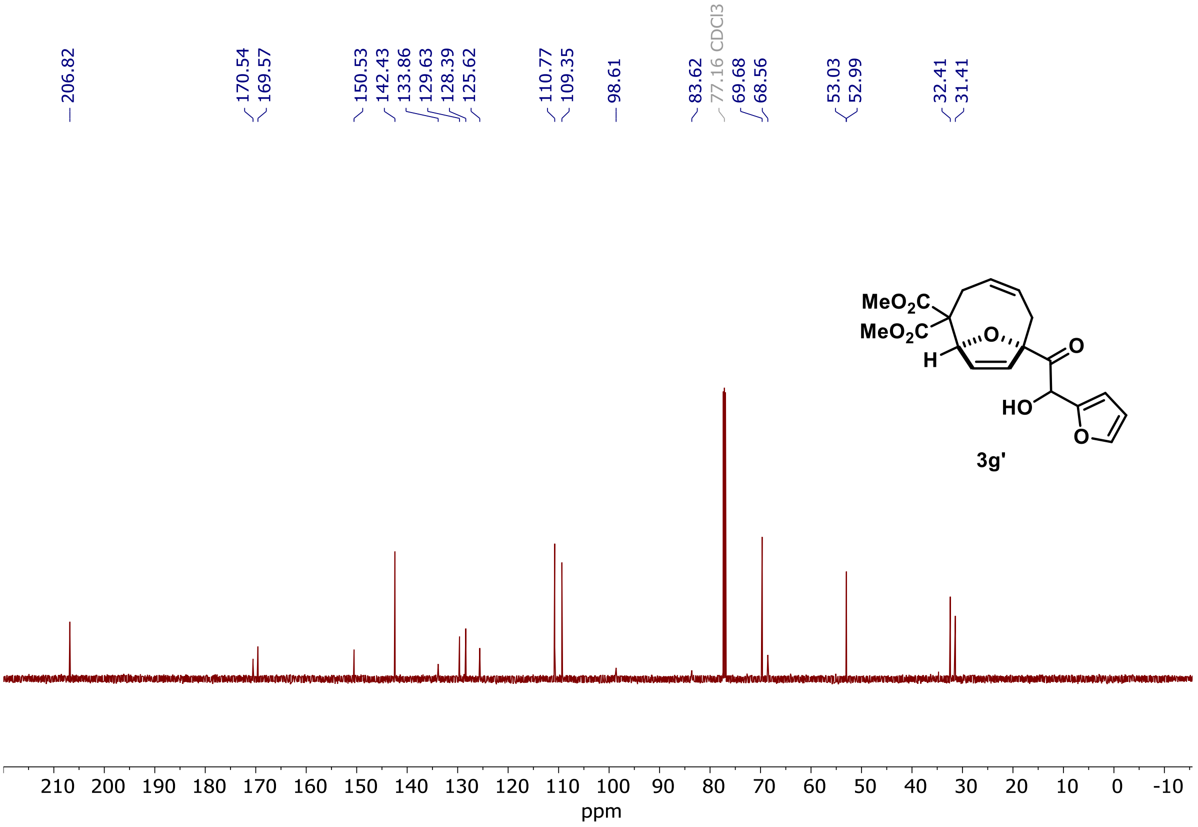

^{1}H NMR (400 MHz, $CDCl_3$) of **3h**

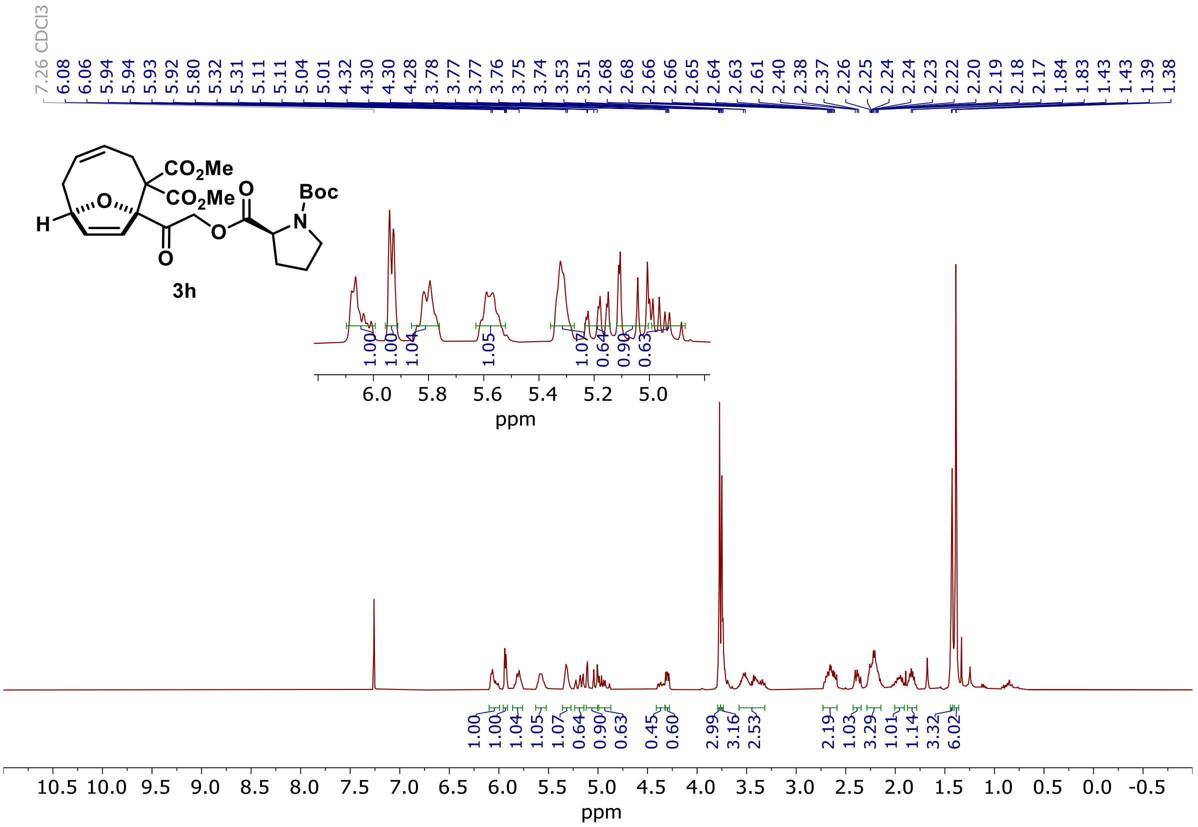

^{13}C NMR (101 MHz, $CDCl_3$) of **3h**

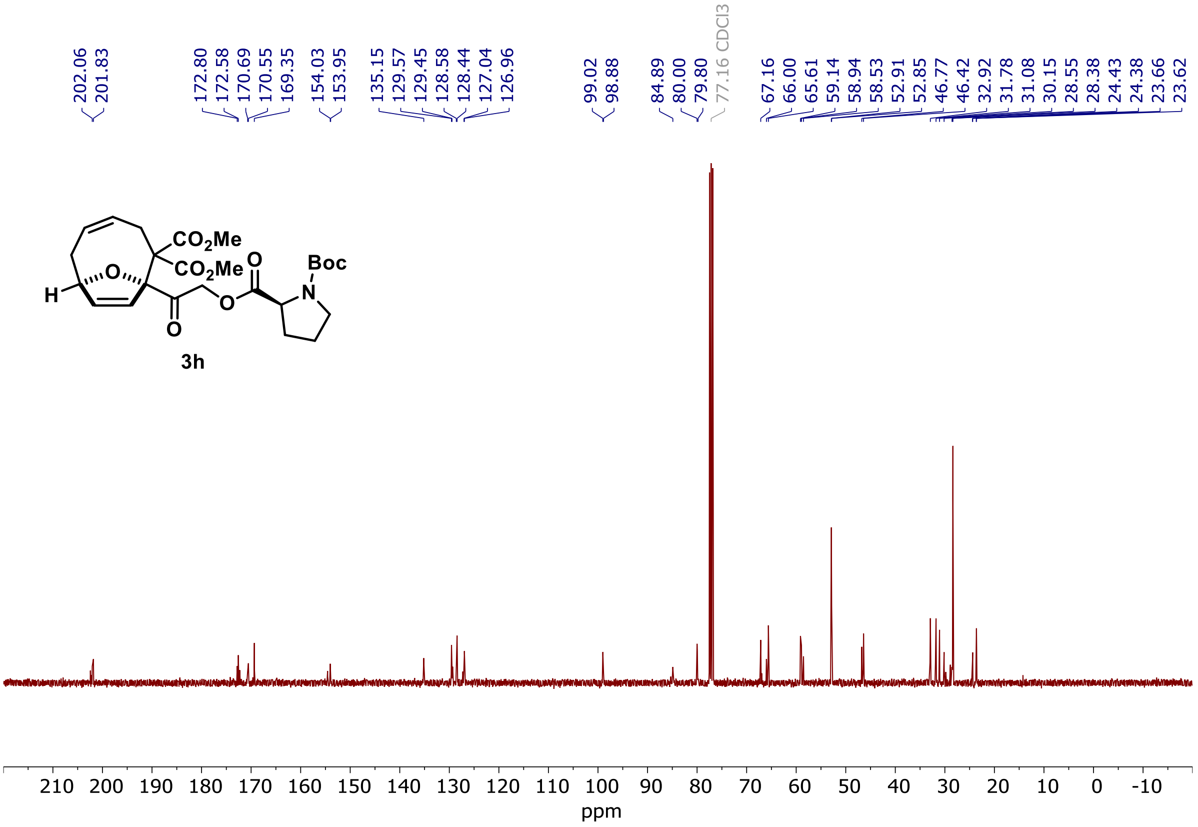

^{1}H NMR (400 MHz, $CDCl_3$) of **3h’**

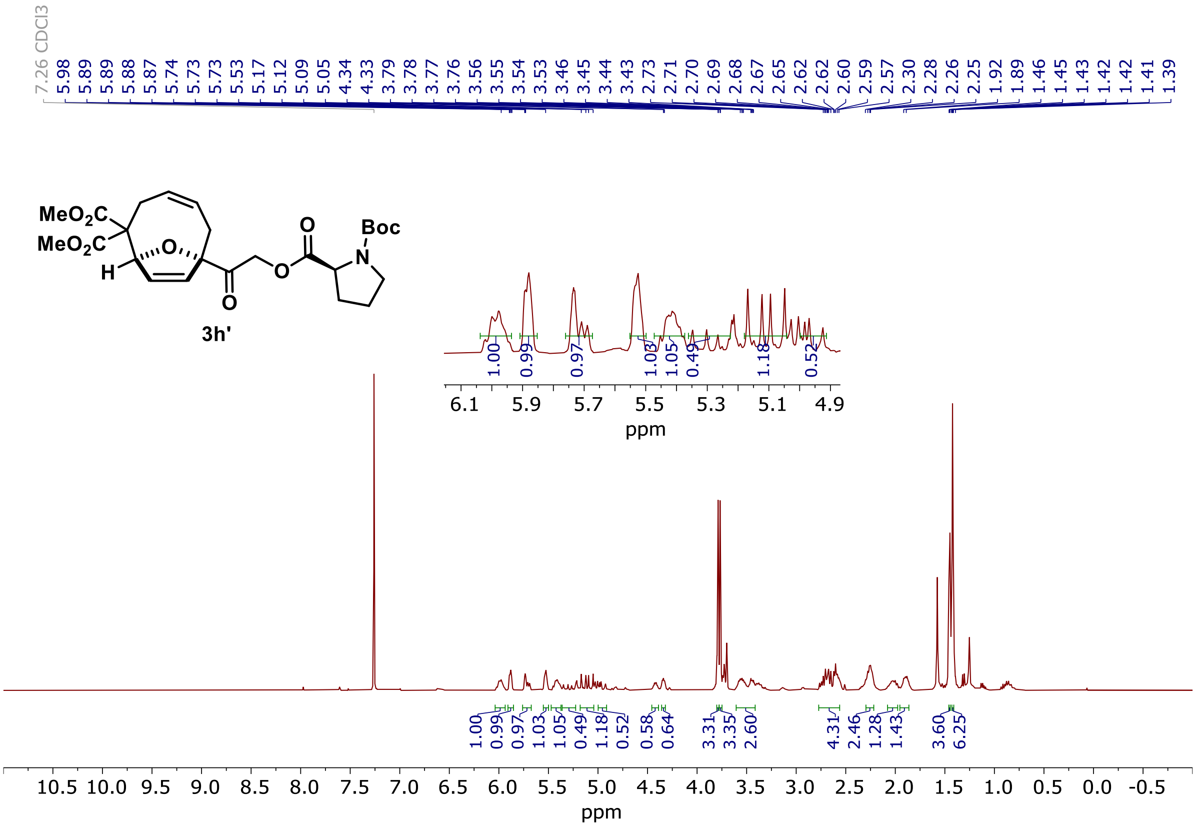

^{13}C NMR (151 MHz, $CDCl_3$) of **3h’**

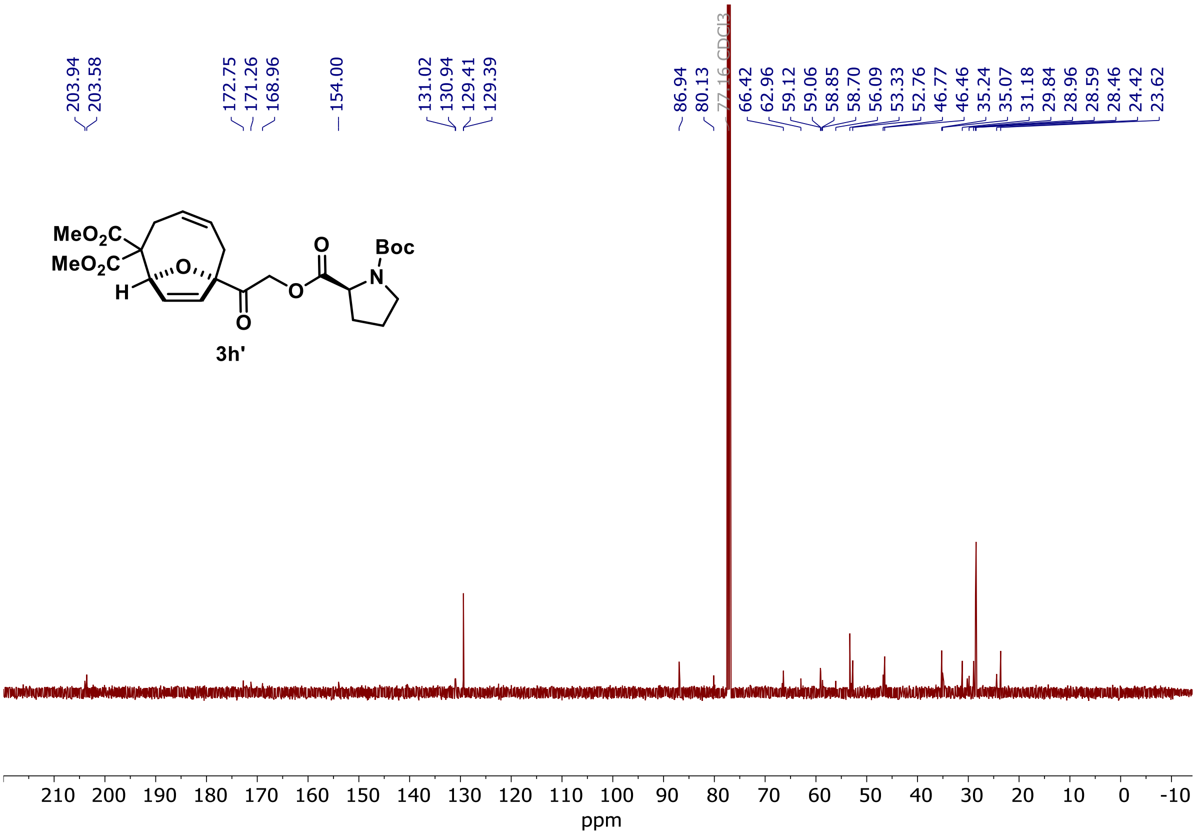

^{1}H NMR (400 MHz, $CDCl_3$) of **3i**

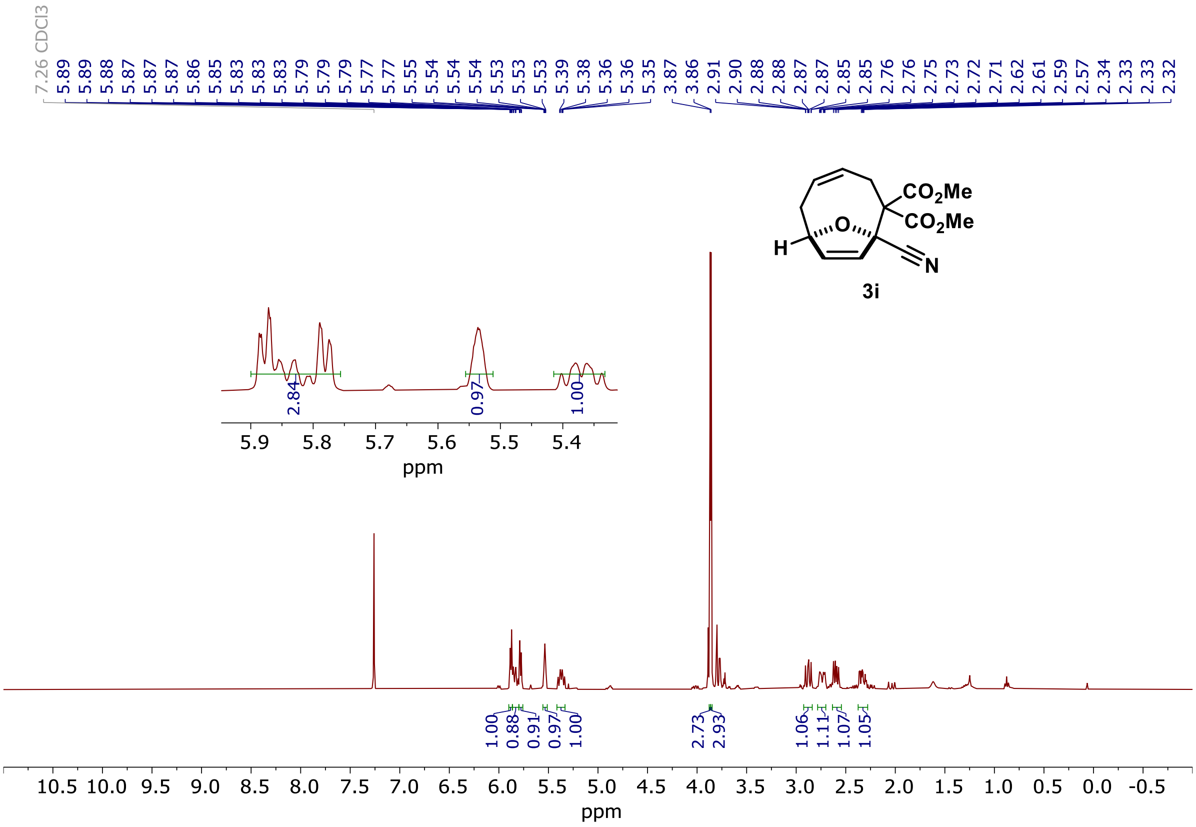

^{13}C NMR (101 MHz, $CDCl_3$) of **3i**

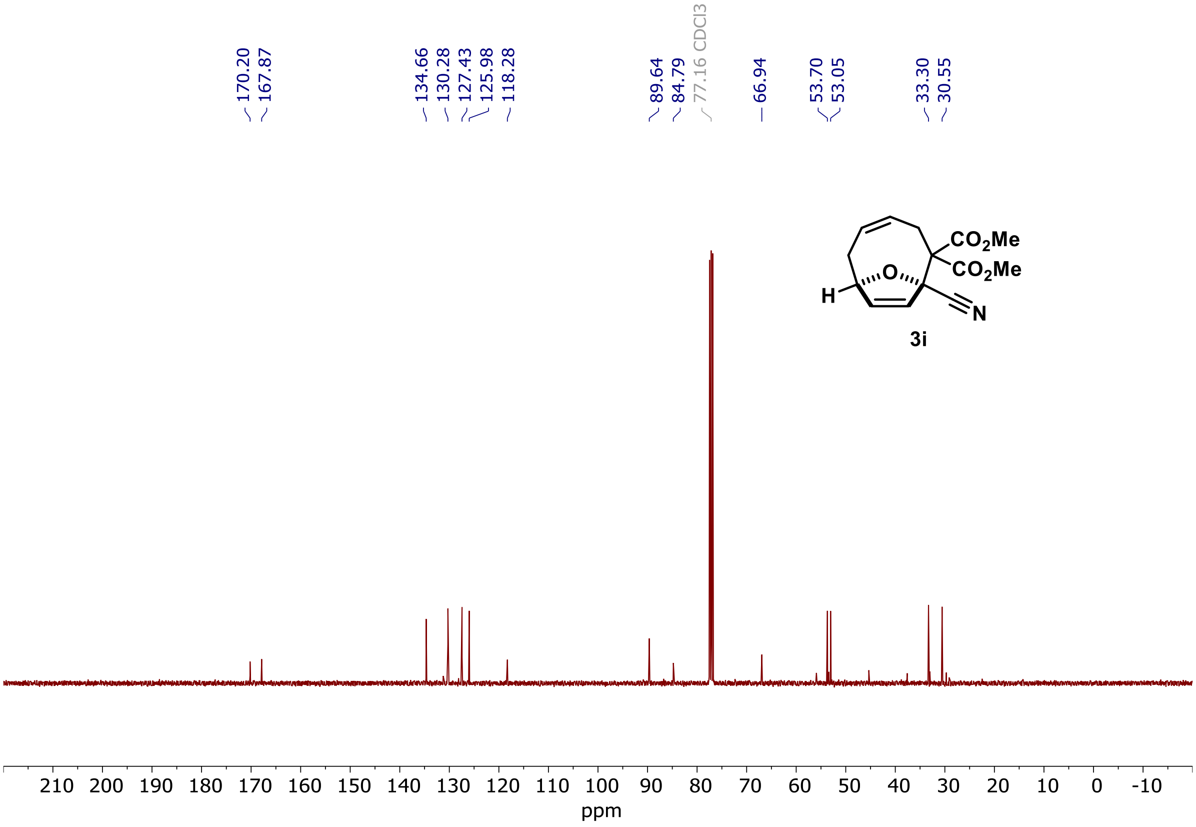

^{1}H NMR (400 MHz, $CDCl_3$) of **3i’**

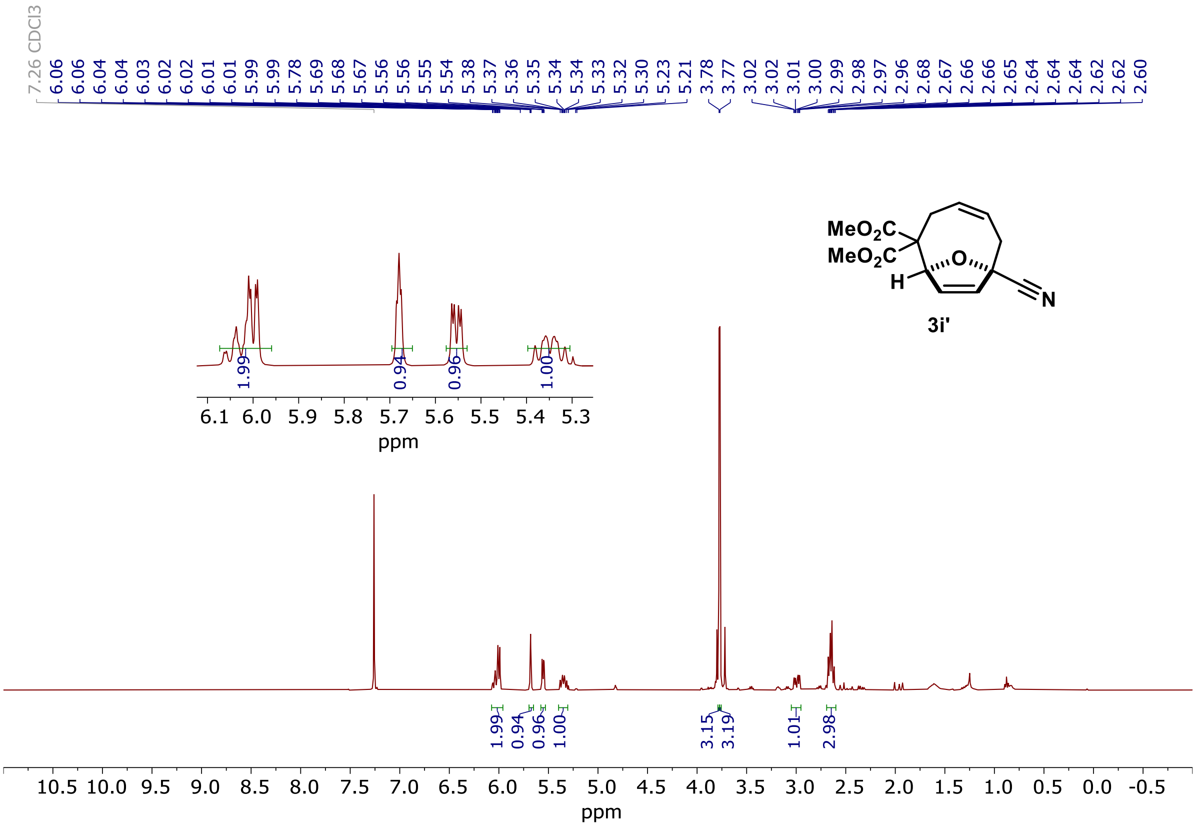

^{13}C NMR (101 MHz, $CDCl_3$) of **3i’**

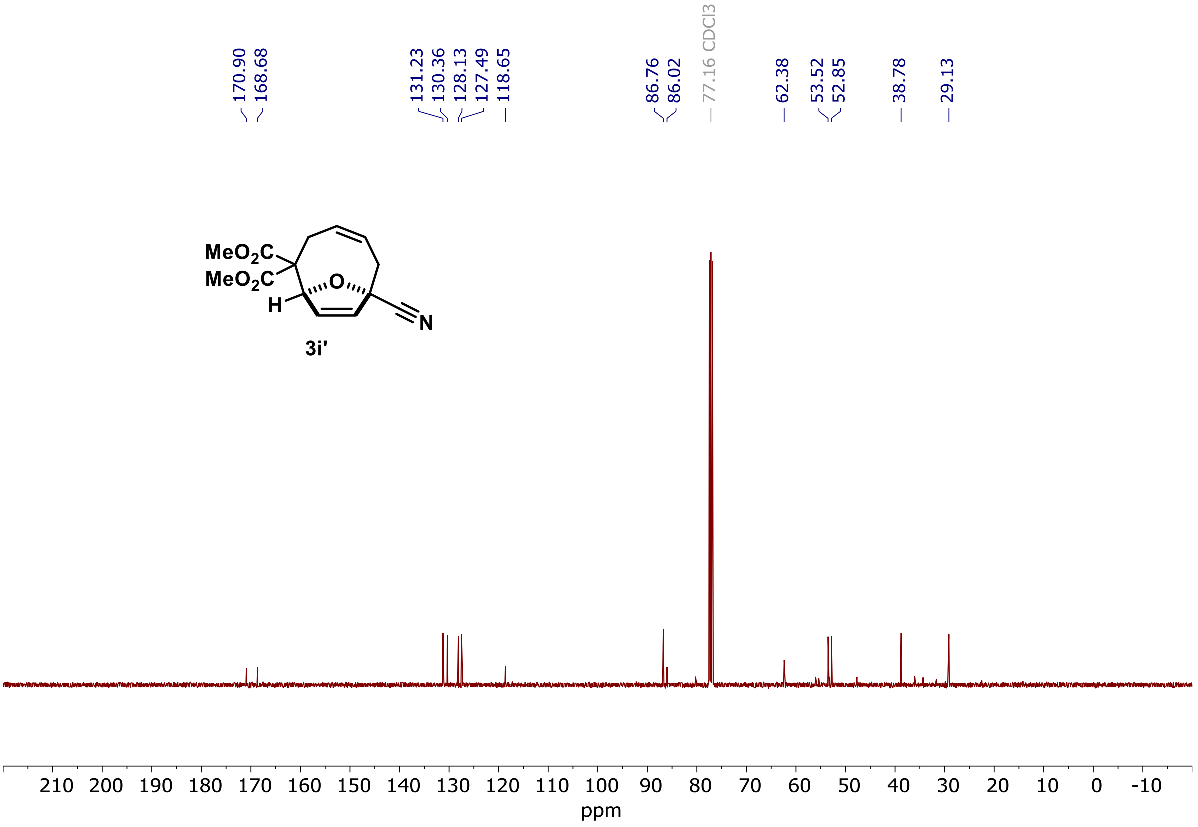

^{1}H NMR (400 MHz, $CDCl_3$) of **3j**

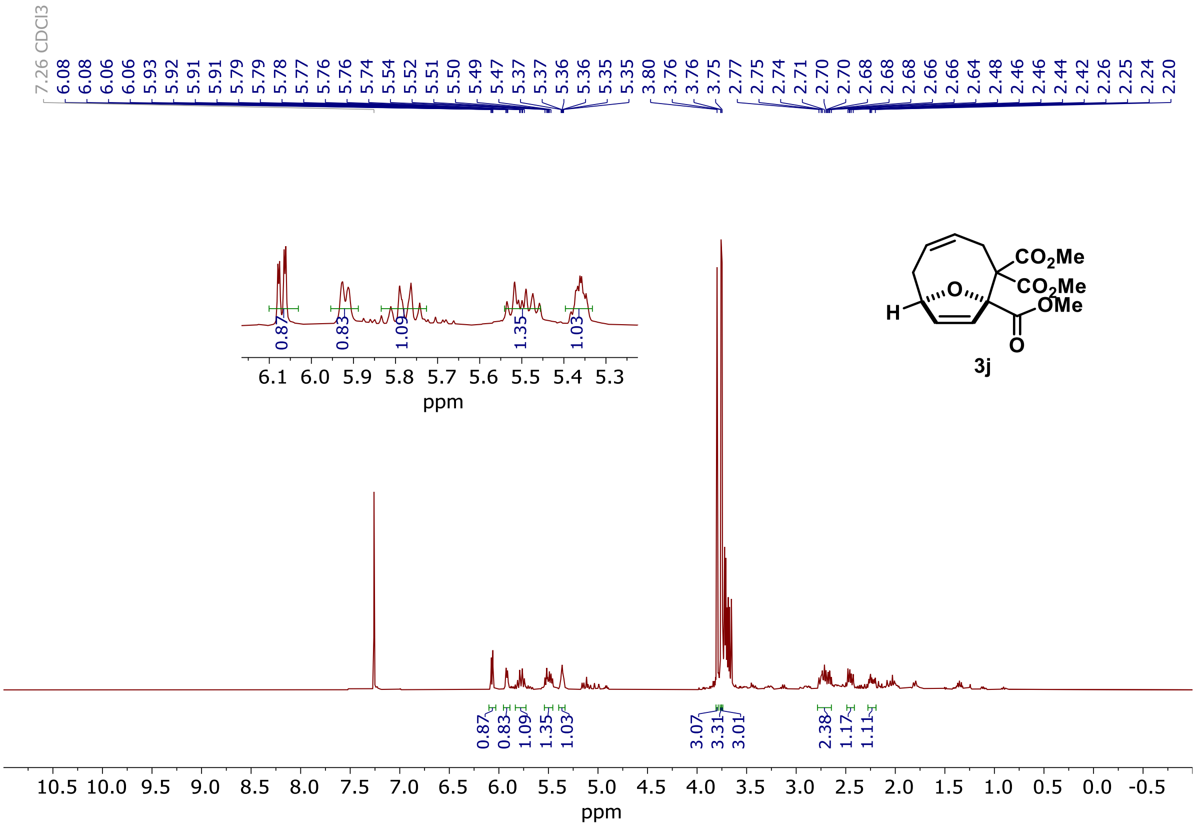

^{13}C NMR (101 MHz, $CDCl_3$) of **3j**

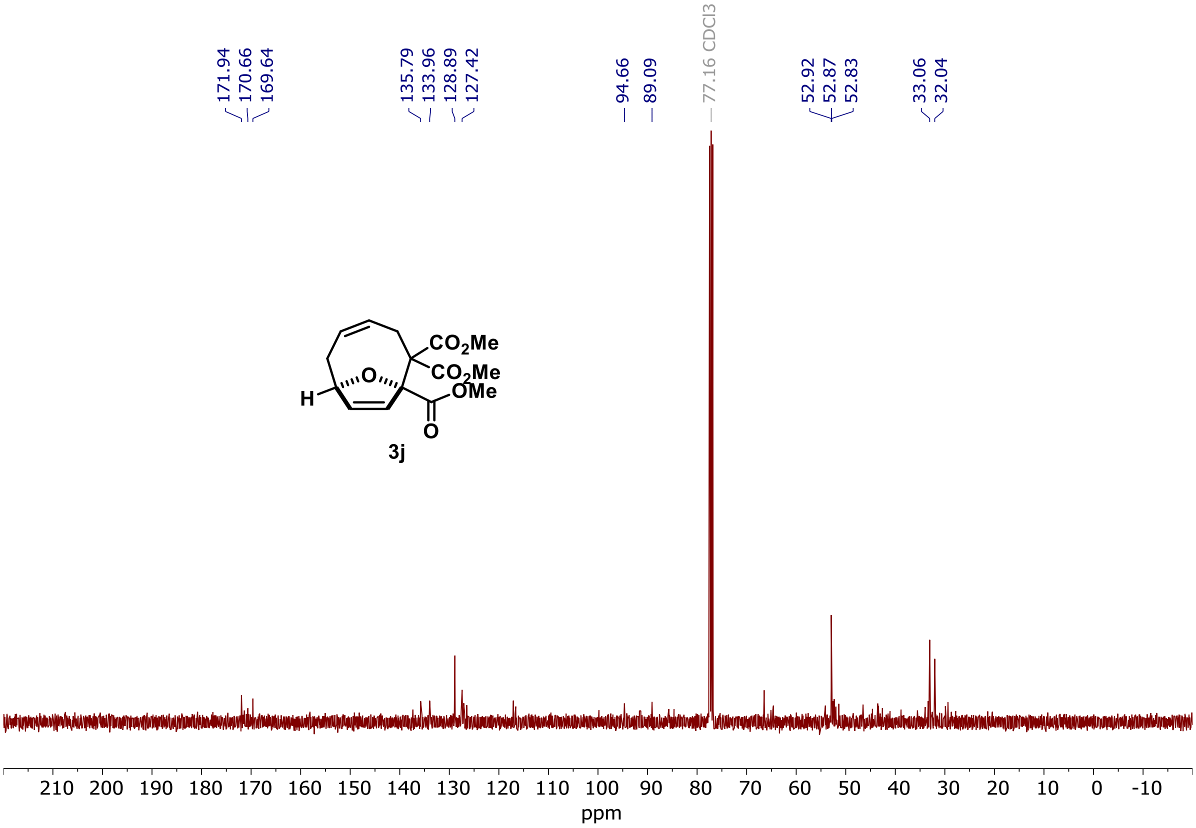

^{1}H NMR (400 MHz, $CDCl_3$) of **3j'**

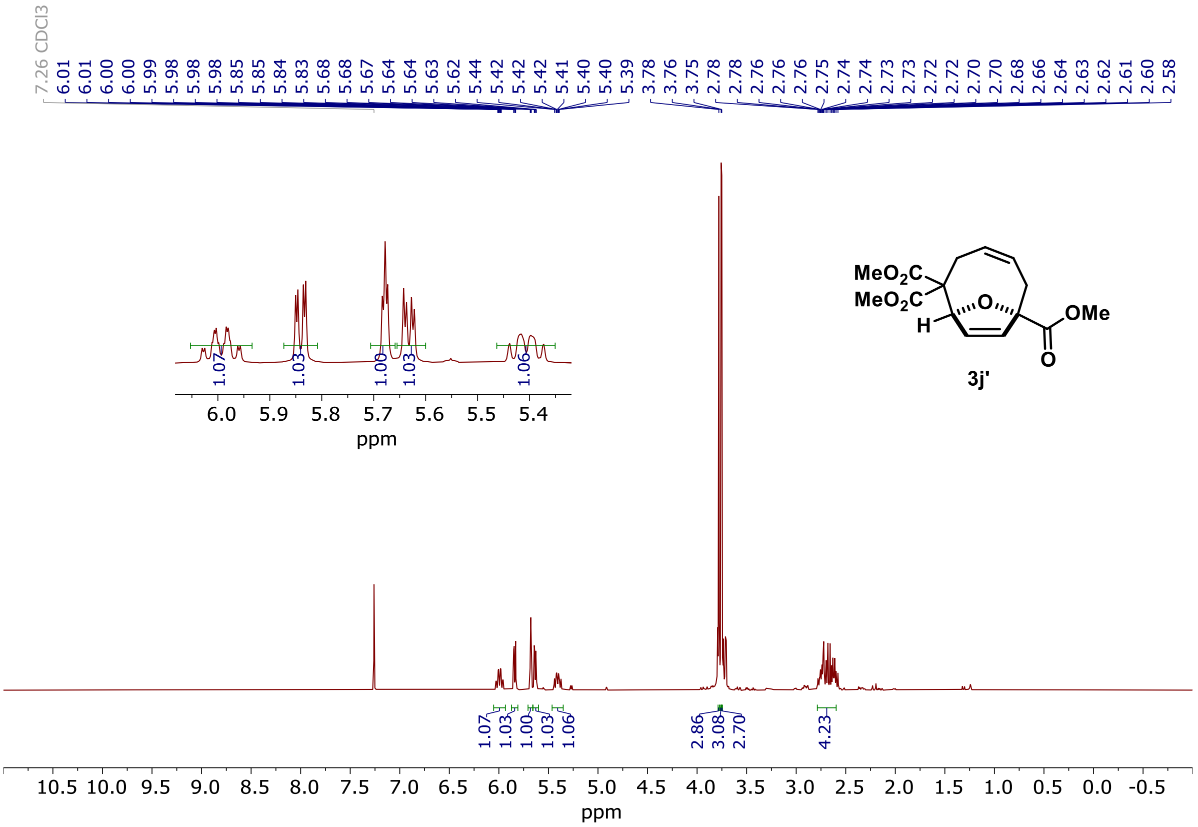

^{13}C NMR (101 MHz, $CDCl_3$) of **3j'**

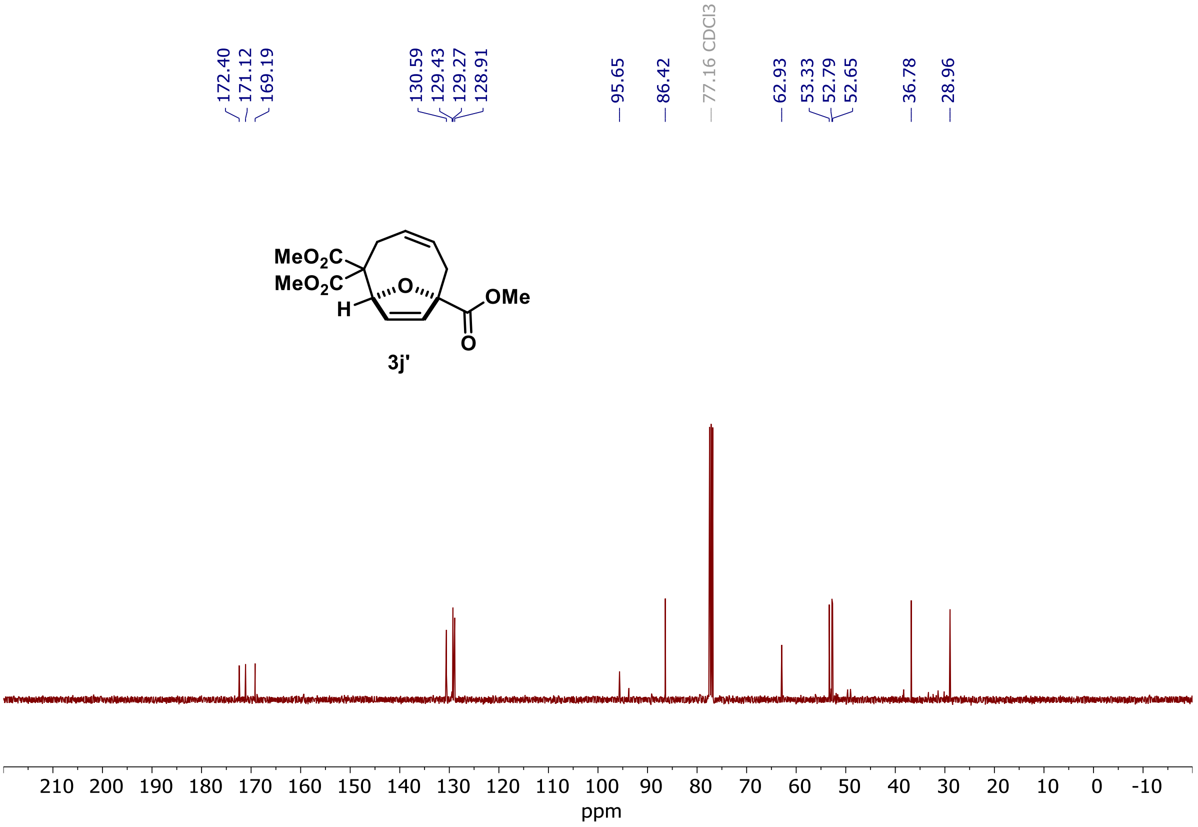

^{1}H NMR (599 MHz, $CDCl_3$) of **3k** and **3k'**

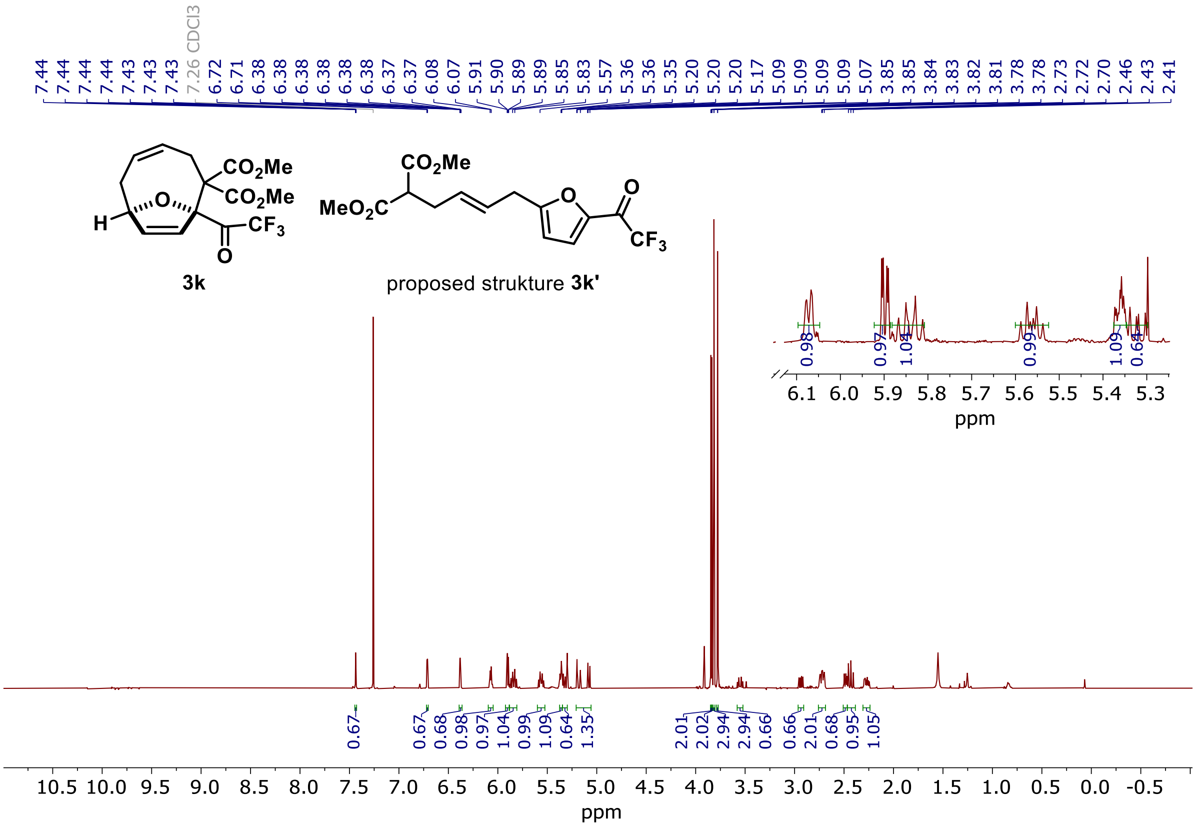

$^{13}C\{^{19}F\}$ NMR (101 MHz, $CDCl_3$) of **3k** and **3k'**

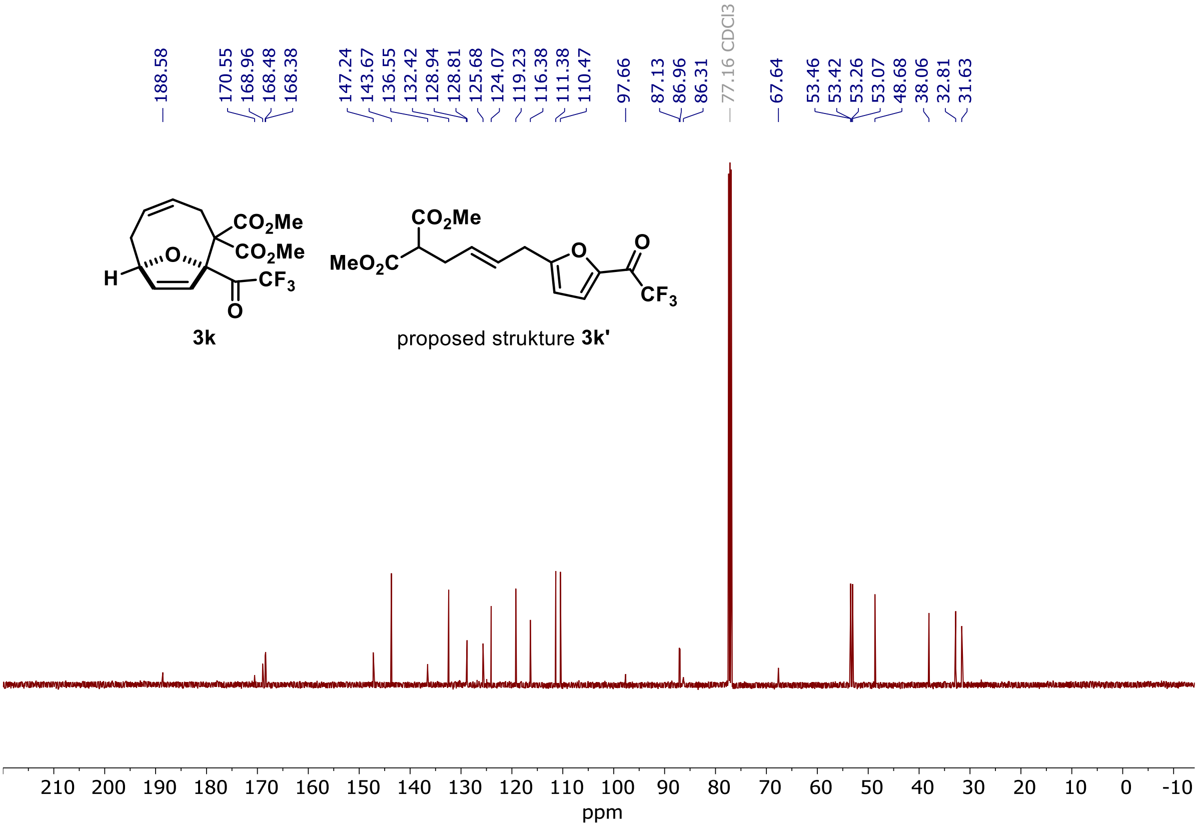

^{13}C NMR (101 MHz, $CDCl_3$) of **3k** and **3k'**

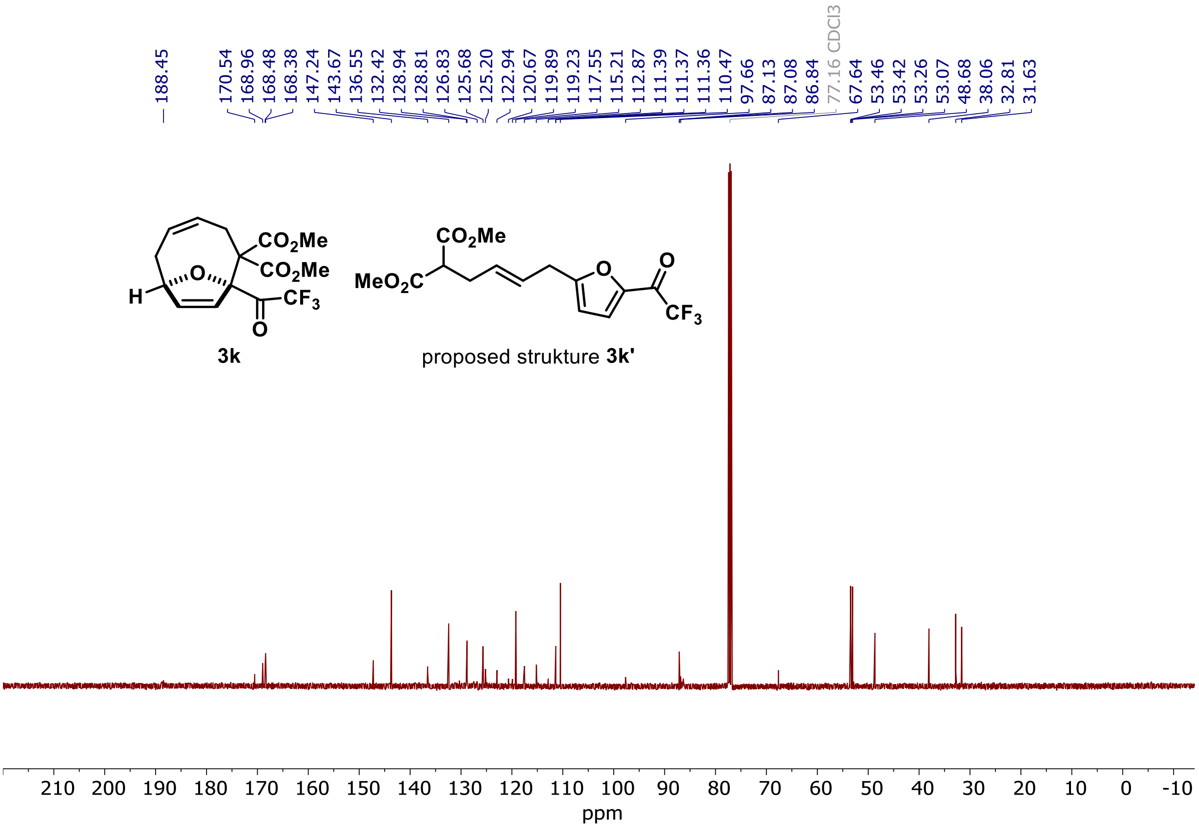

^{19}F NMR (376 MHz, $CDCl_3$) of **3k** and **3k**'

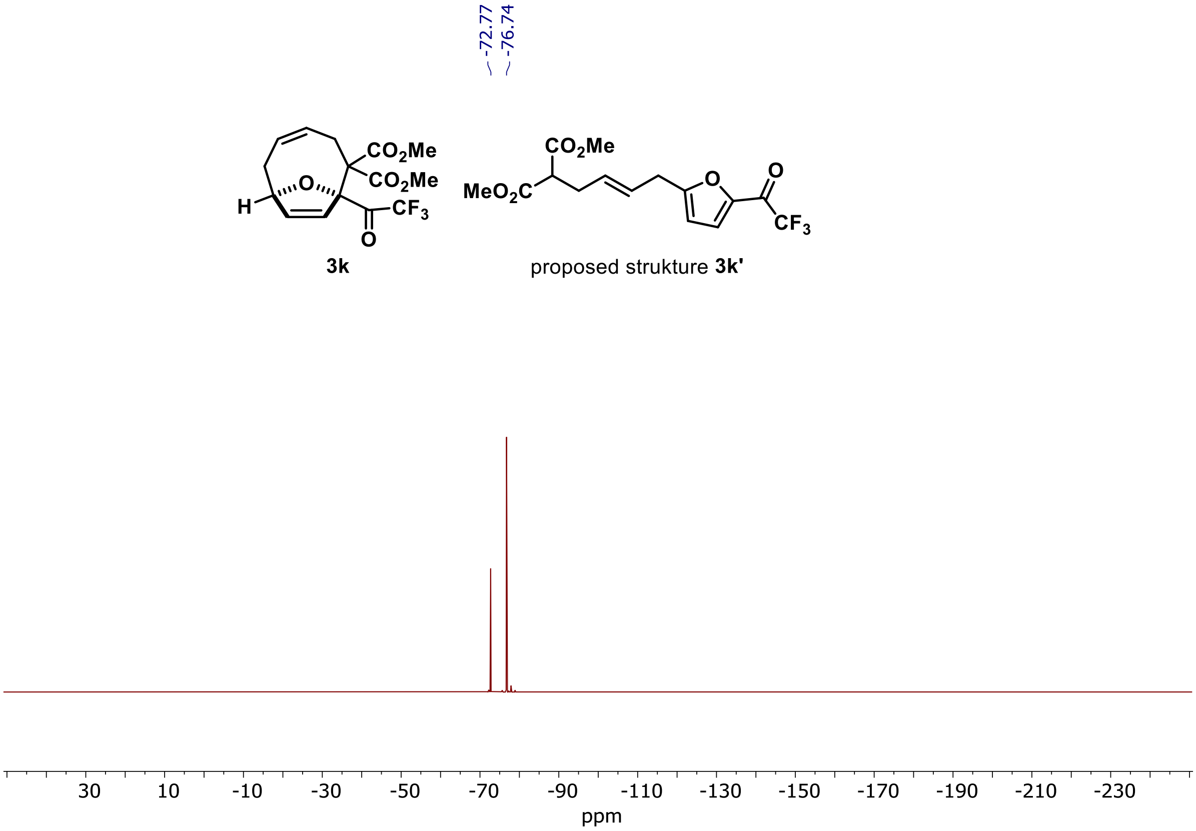

^{1}H NMR (400 MHz, $CDCl_3$) of **3l**

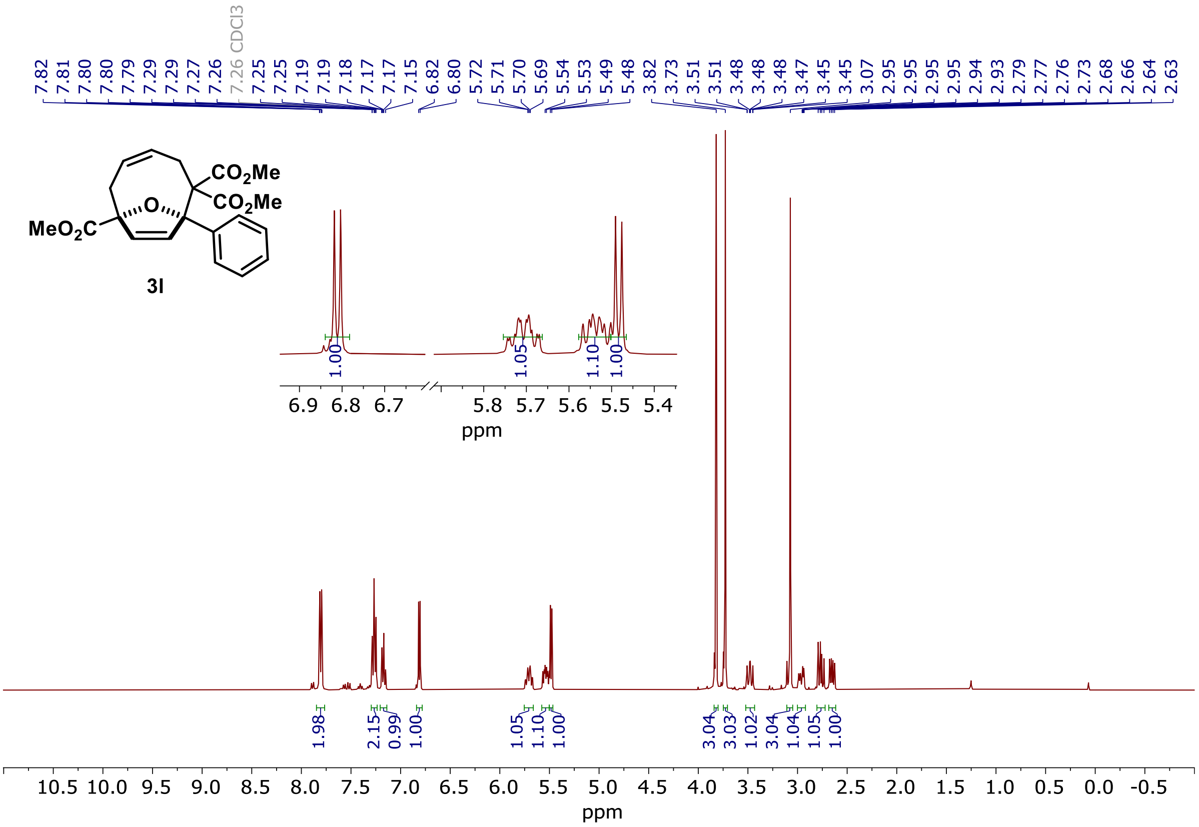

^{13}C NMR (101 MHz, $CDCl_3$) of **3l**

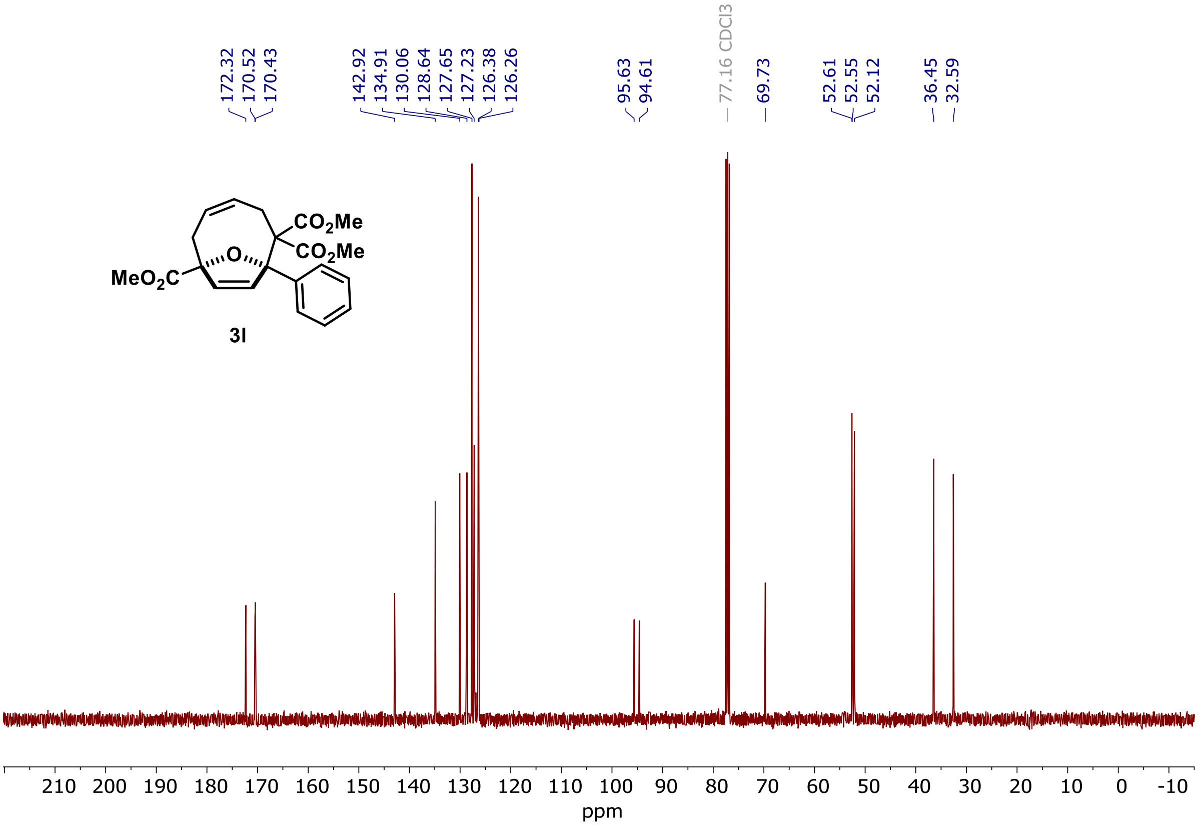

^{1}H NMR (400 MHz, $CDCl_3$) of **3m**

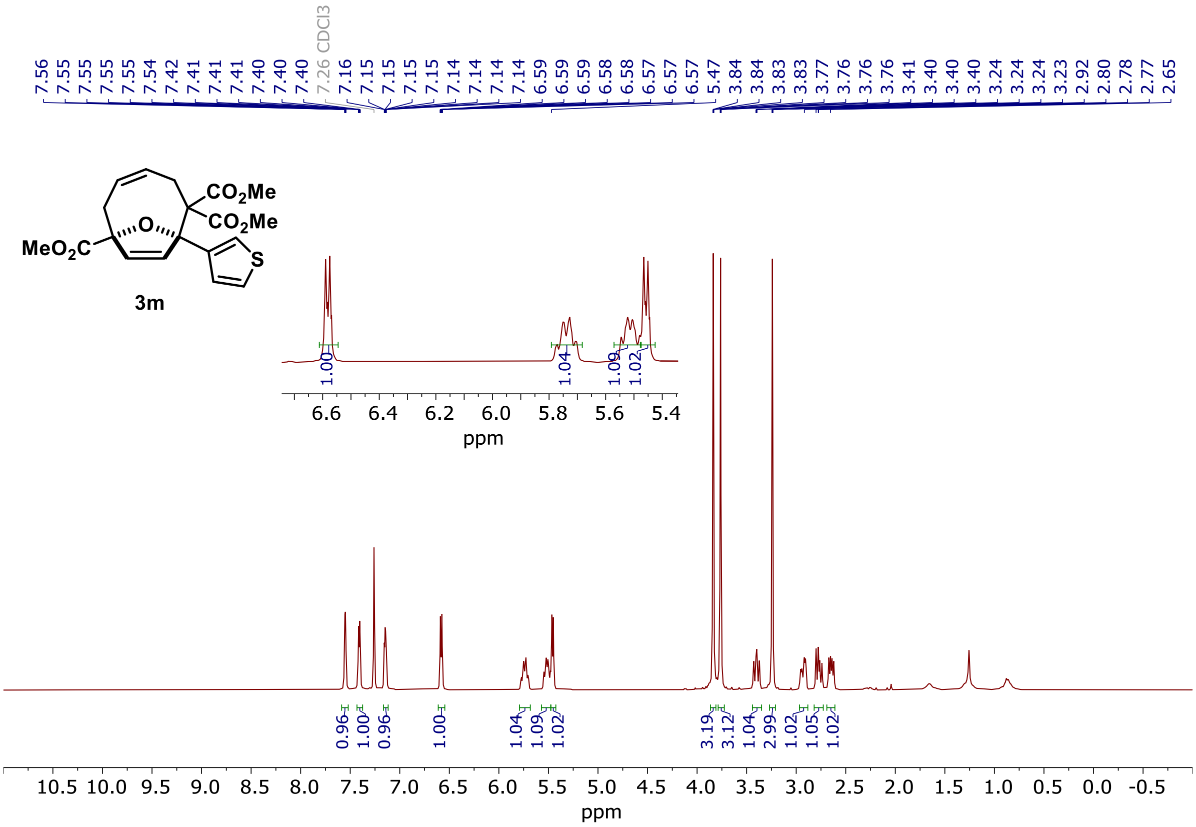

^{13}C NMR (126 MHz, $CDCl_3$) of **3m**

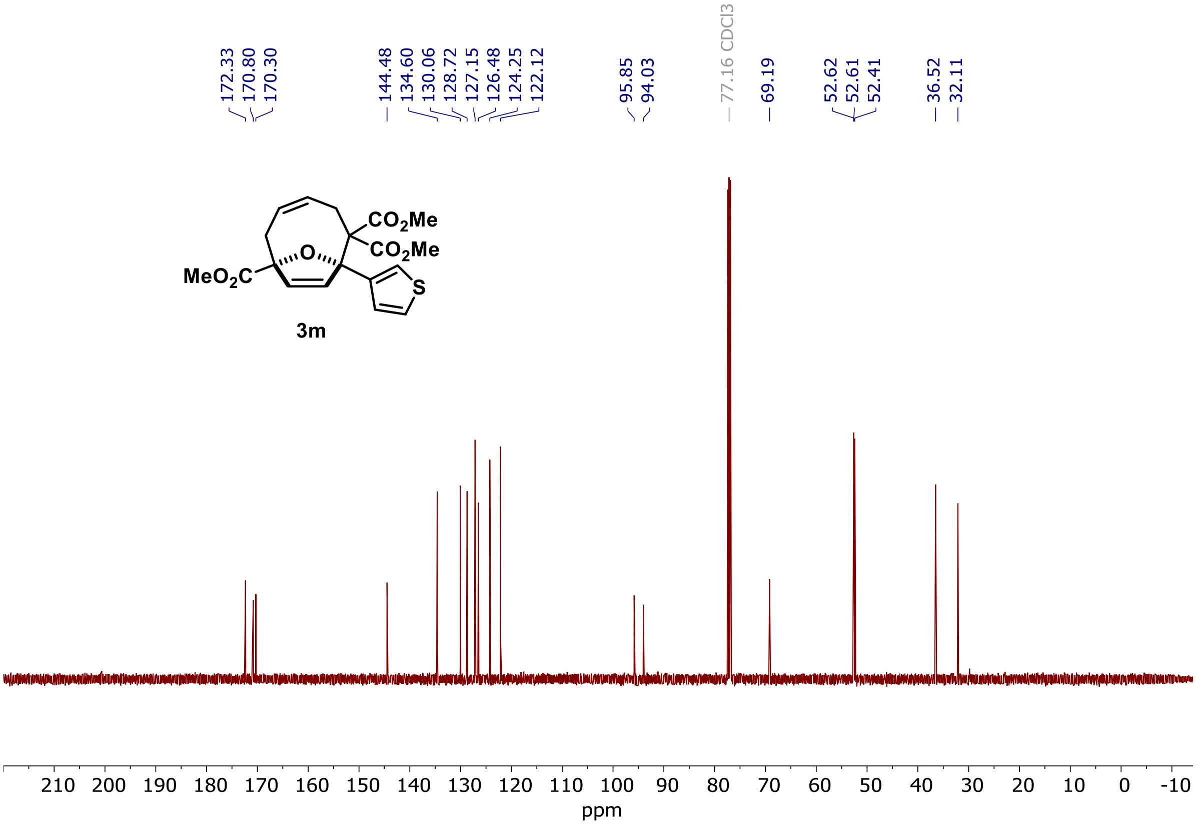

^{1}H NMR (599 MHz, $CDCl_3$) of **3n**

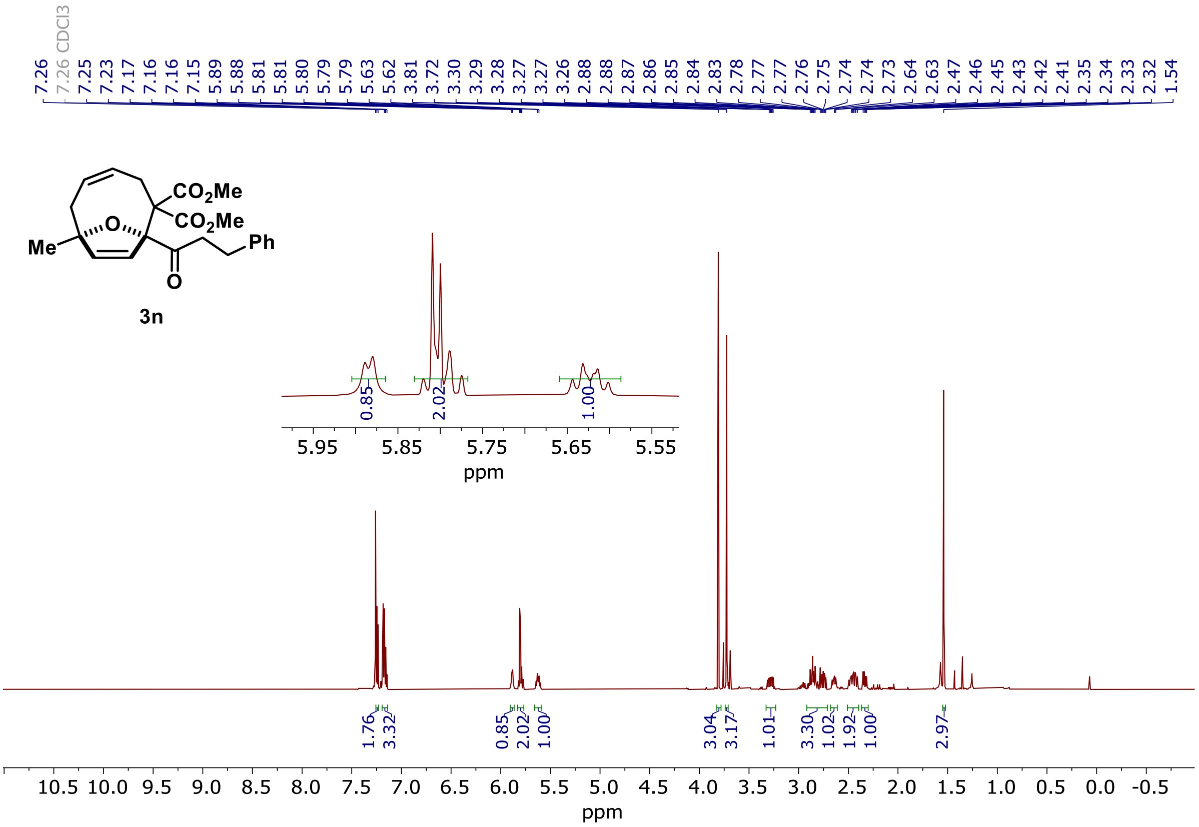

^{13}C NMR (151 MHz, $CDCl_3$) of **3n**

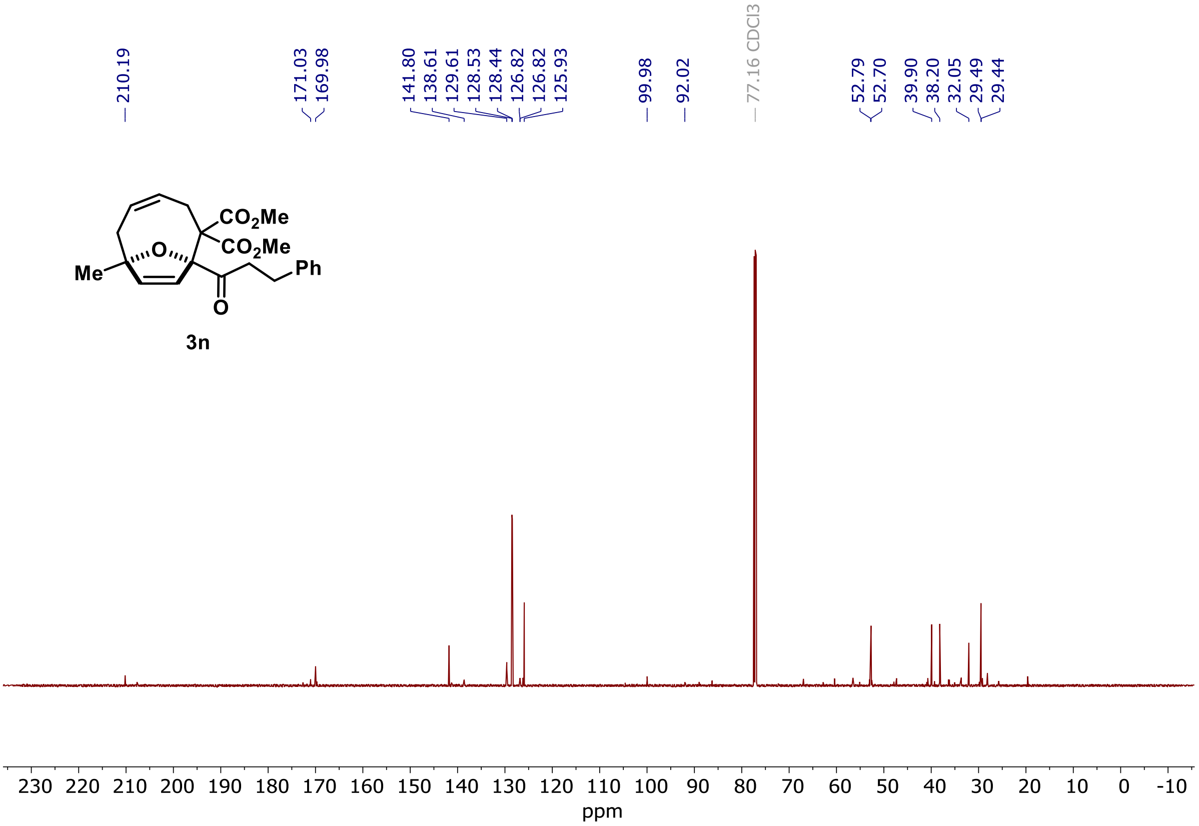

^{1}H NMR (400 MHz, $CDCl_3$) of **3o**

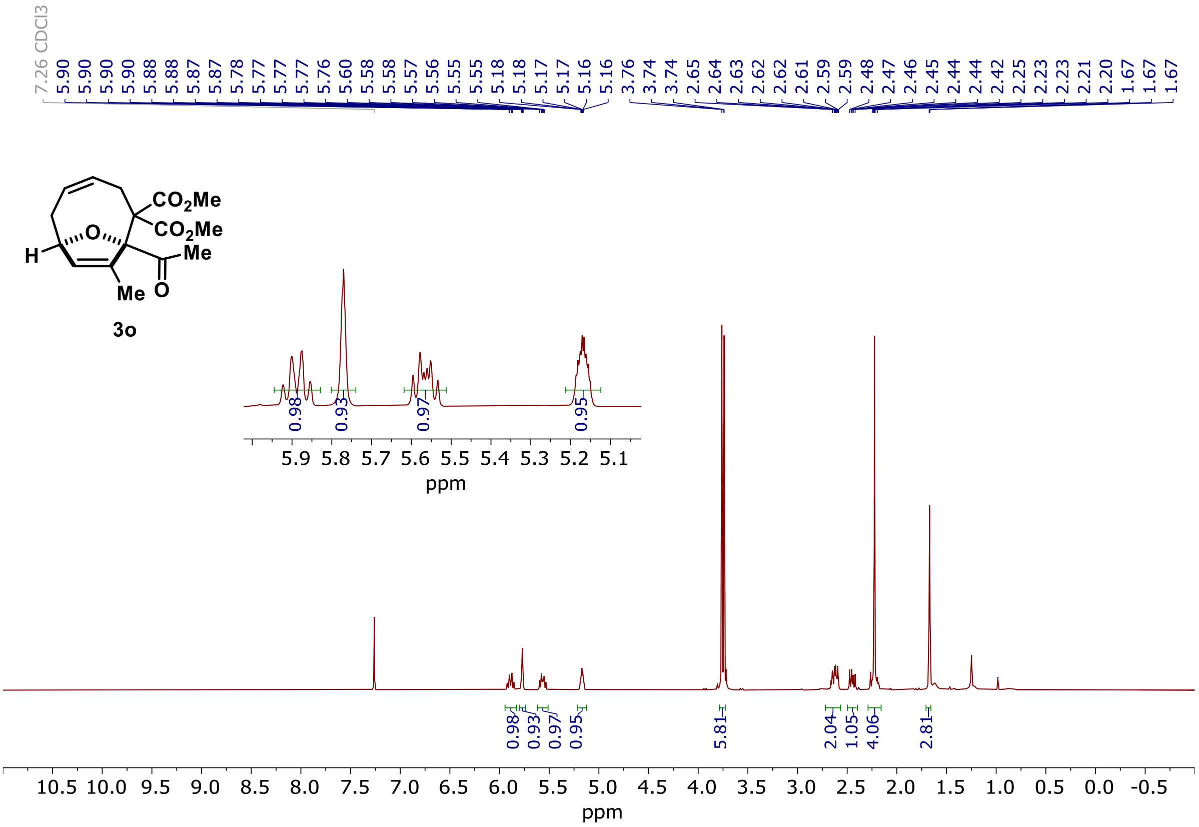

^{13}C NMR (151 MHz, $CDCl_3$) of **3o**

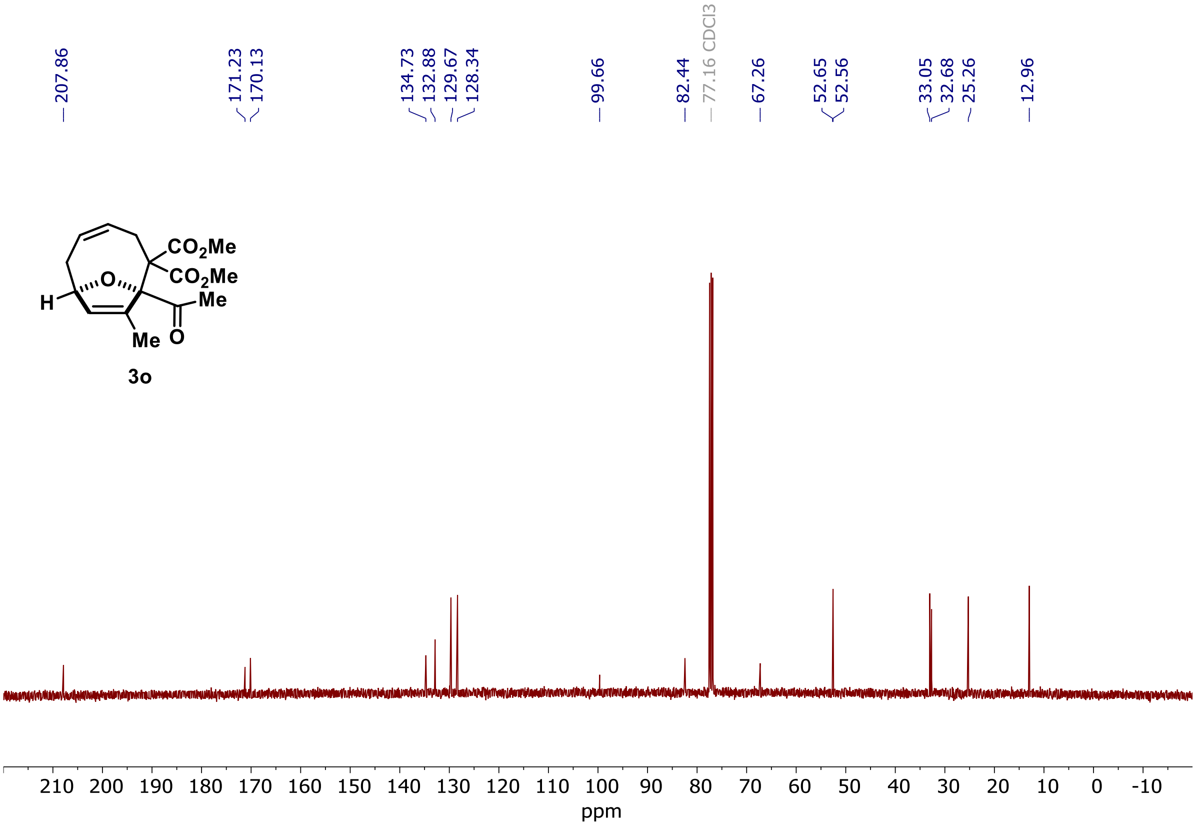

^{1}H NMR (400 MHz, $CDCl_3$) of **3o'**

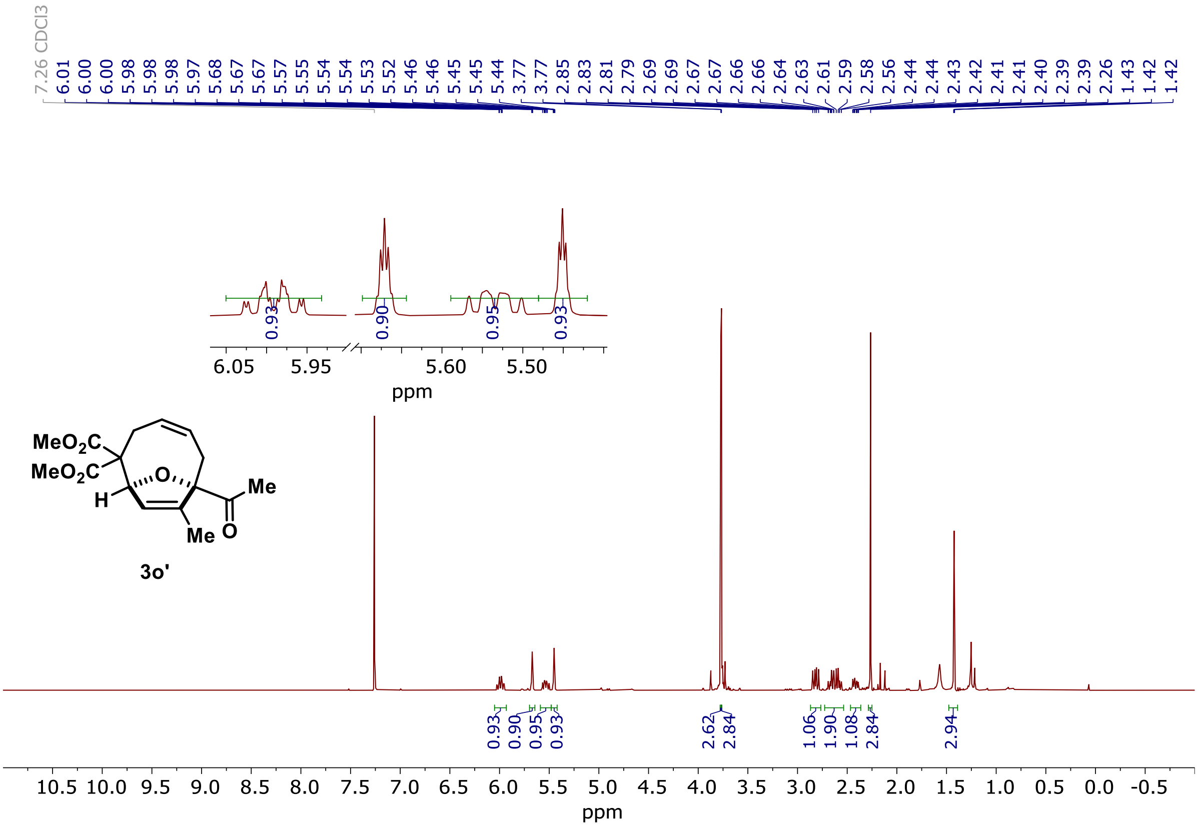

^{13}C NMR (151 MHz, $CDCl_3$) of **3o'**

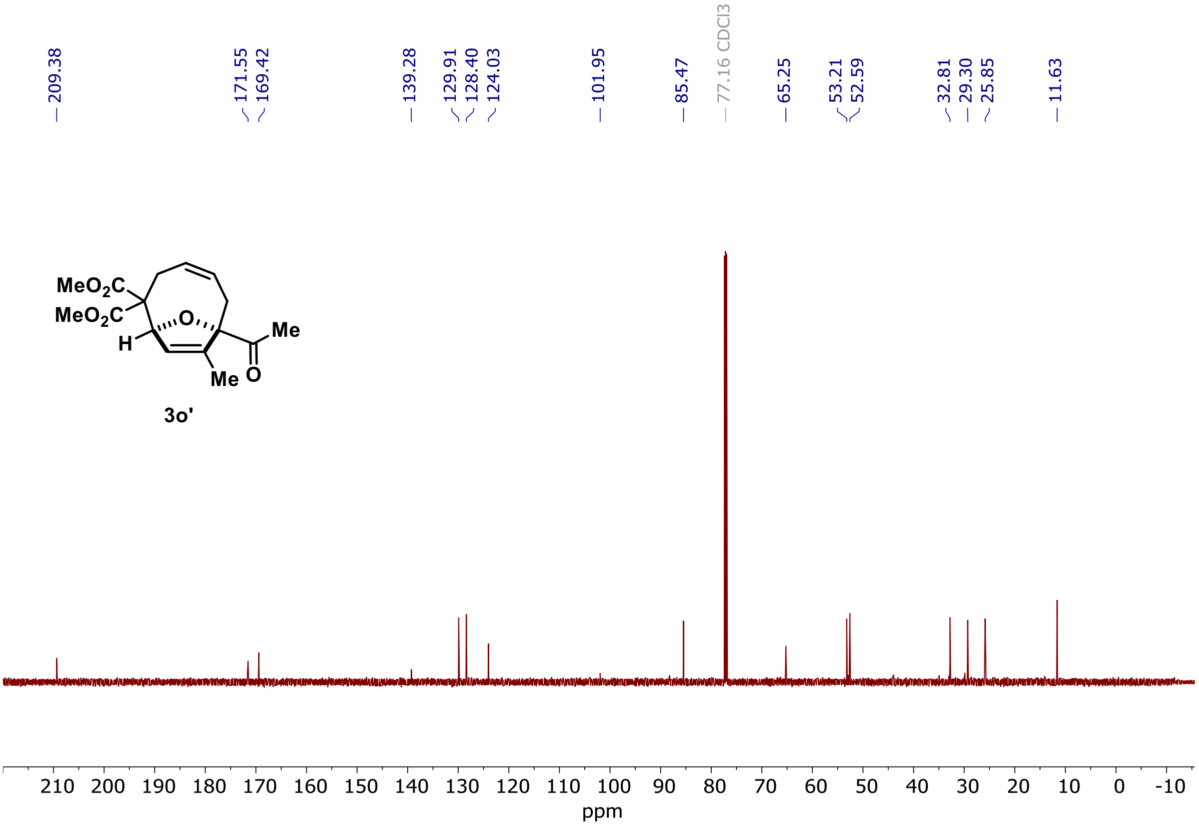

^{1}H NMR (400 MHz, $CDCl_3$) of **3p**

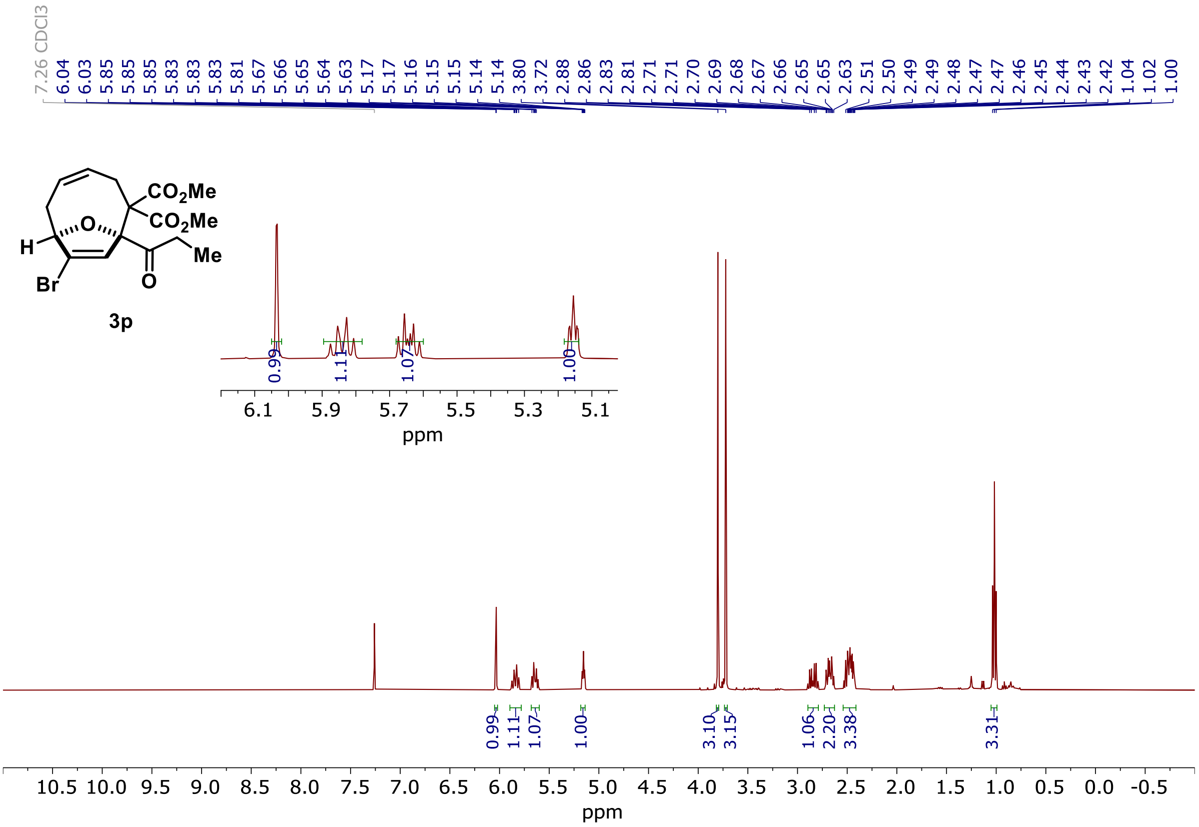

^{13}C NMR (101 MHz, $CDCl_3$) of **3p**

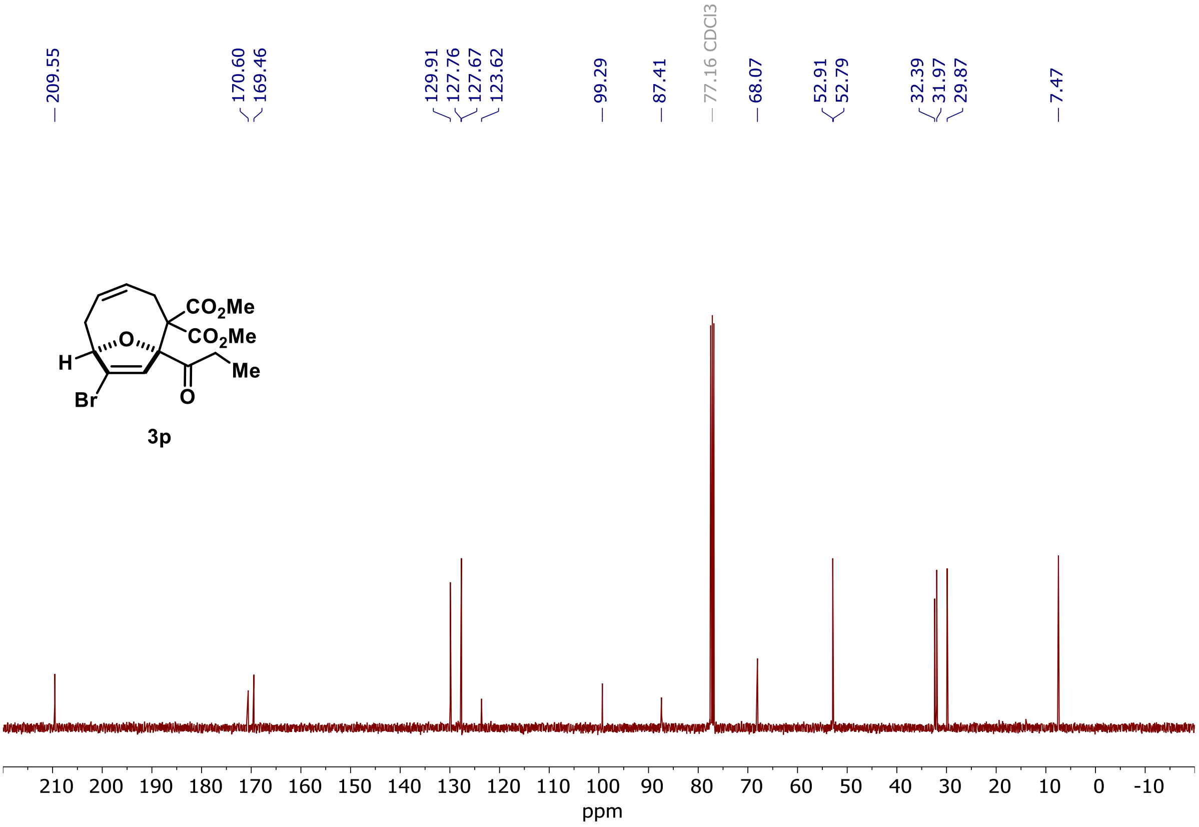

^{1}H NMR (400 MHz, $CDCl_3$) of **3q**

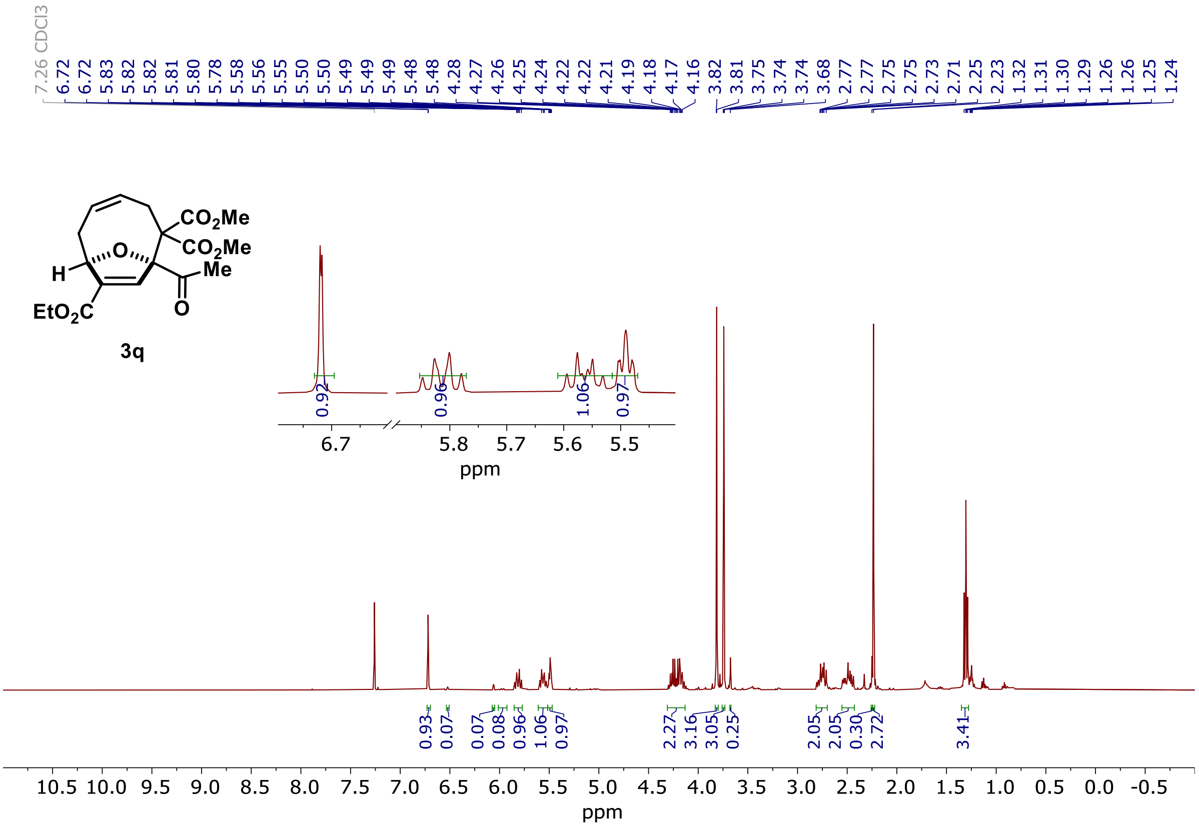

^{13}C NMR (101 MHz, $CDCl_3$) of **3q**

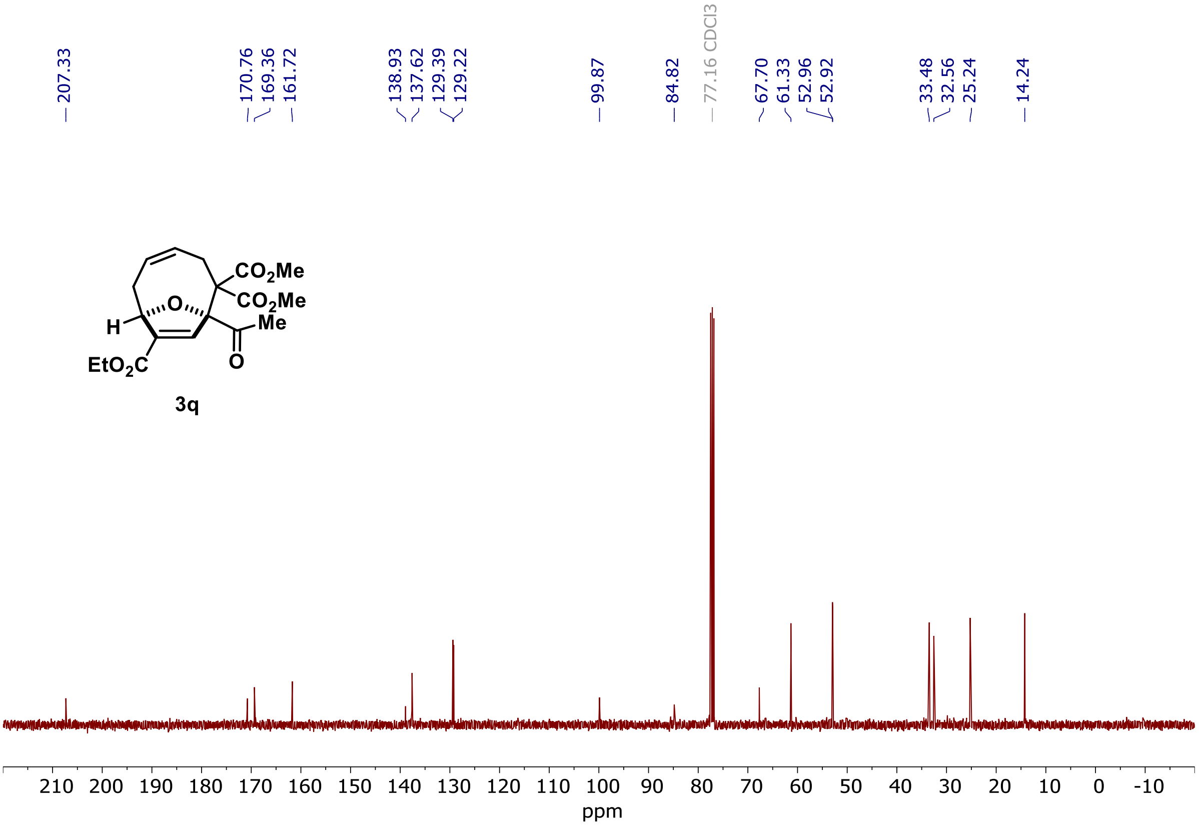

^{1}H NMR (400 MHz, $CDCl_3$) of **3r**

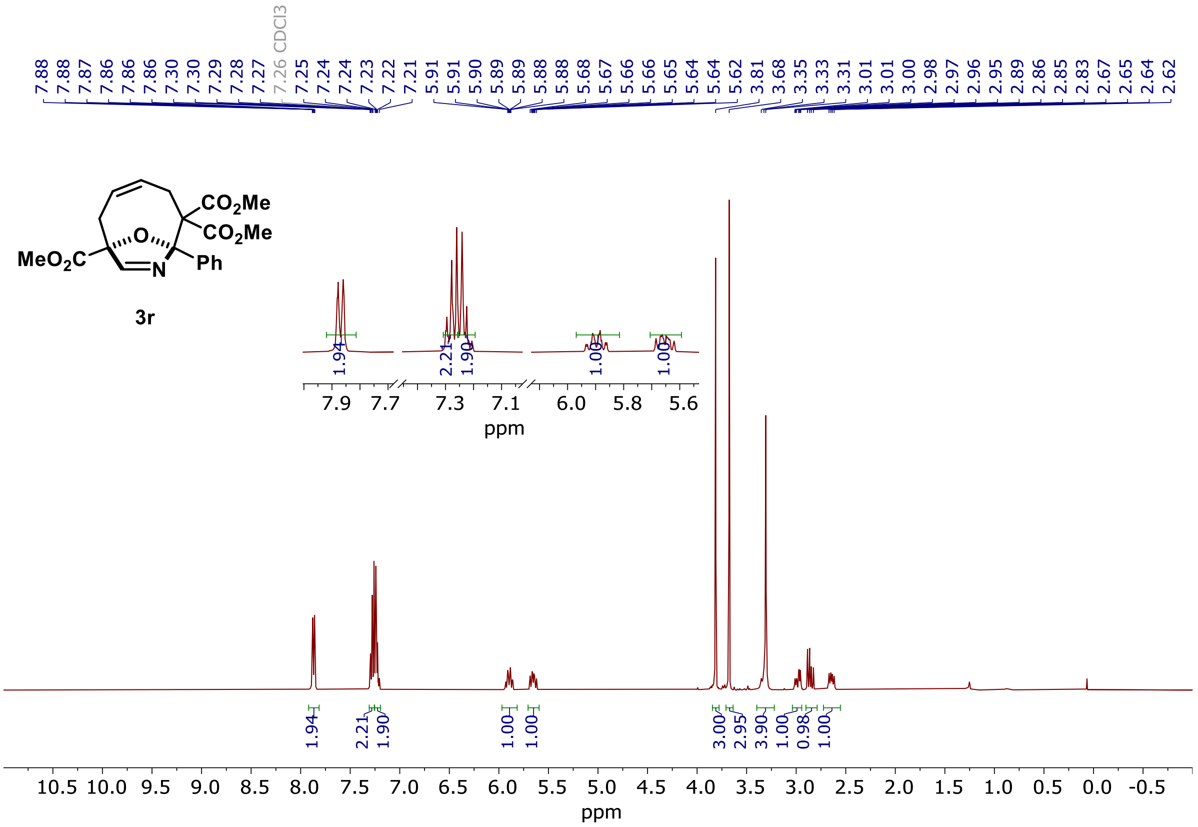

^{13}C NMR (101 MHz, $CDCl_3$) of **3r**

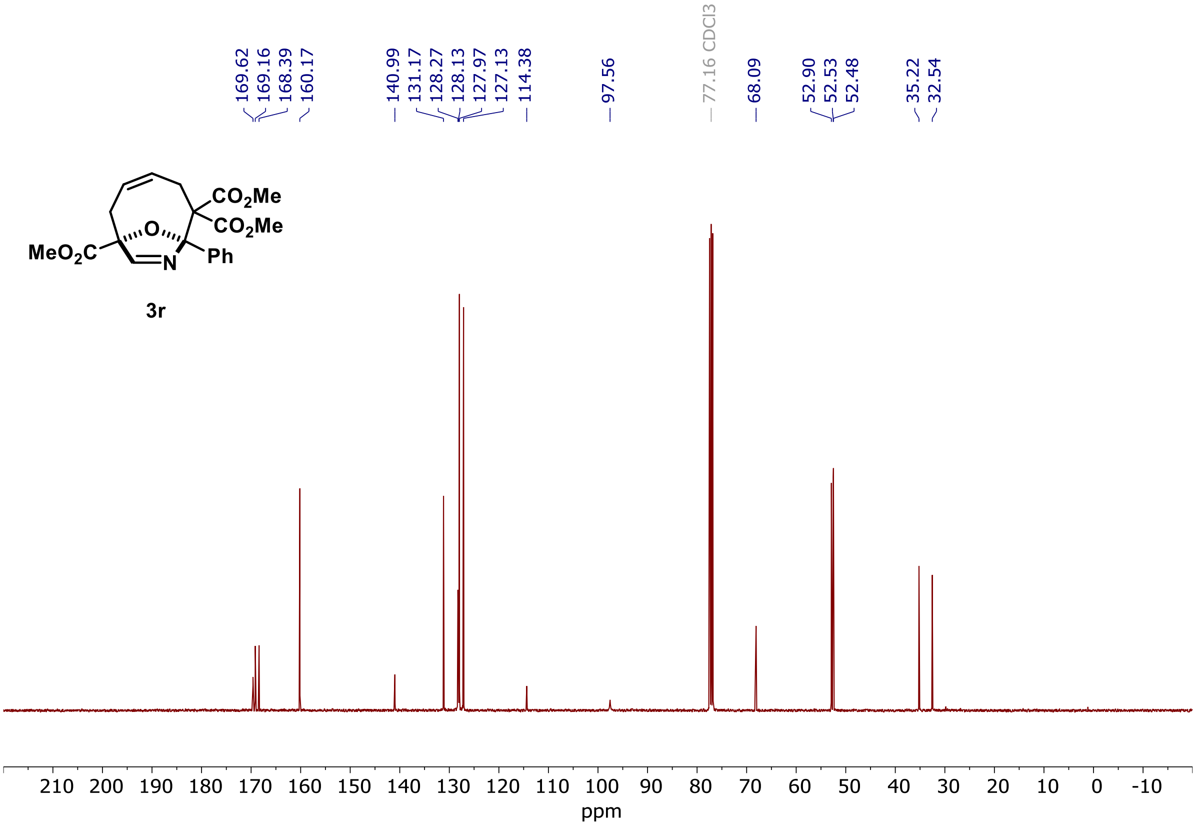

^1H NMR (400 MHz, $CDCl_3$) of **3r'**

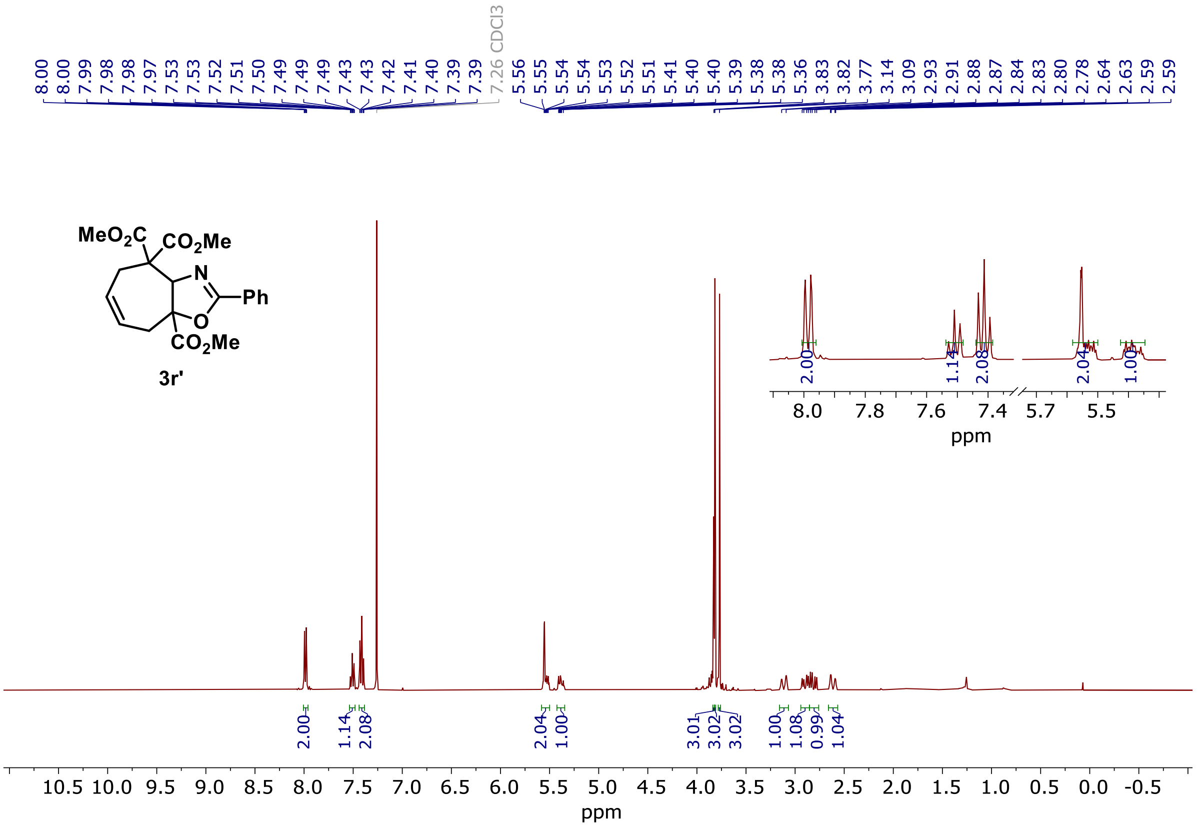

^{13}C NMR (101 MHz, $CDCl_3$) of **3r'**

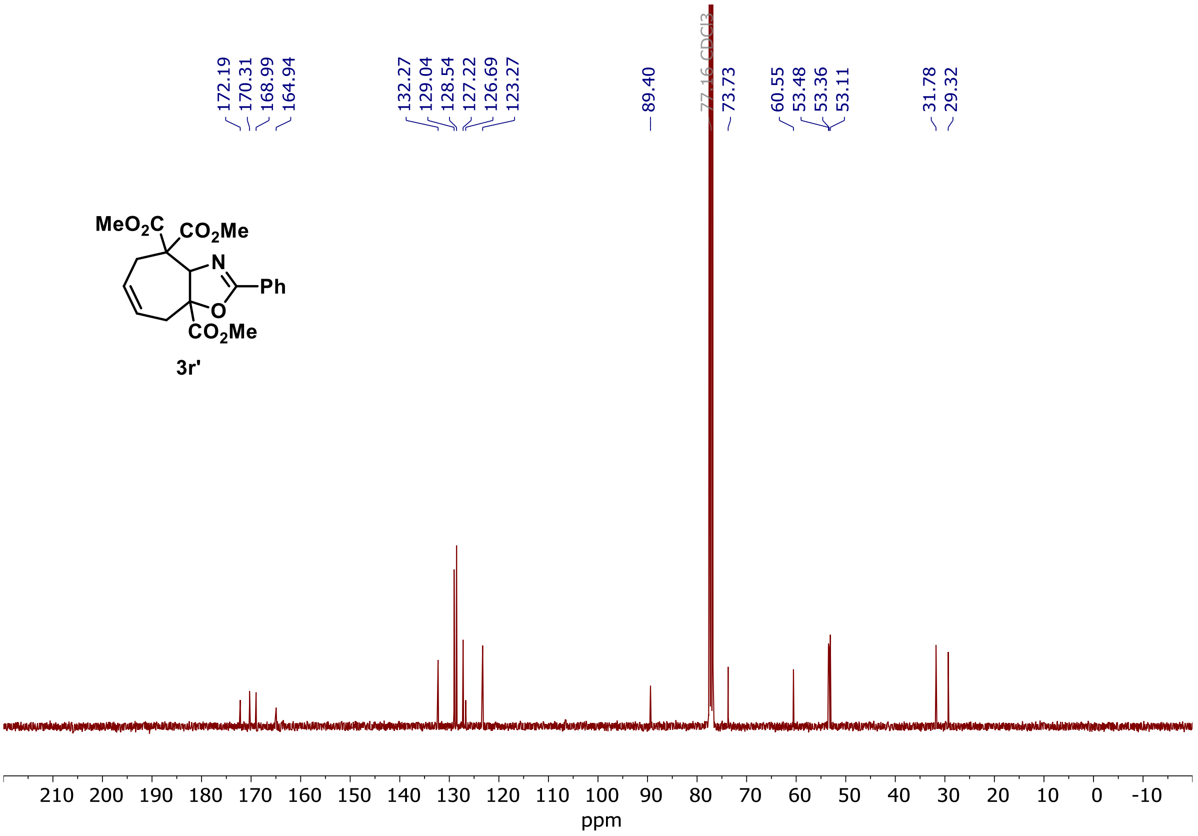

^{1}H NMR (400 MHz, $CDCl_3$) of **3s**

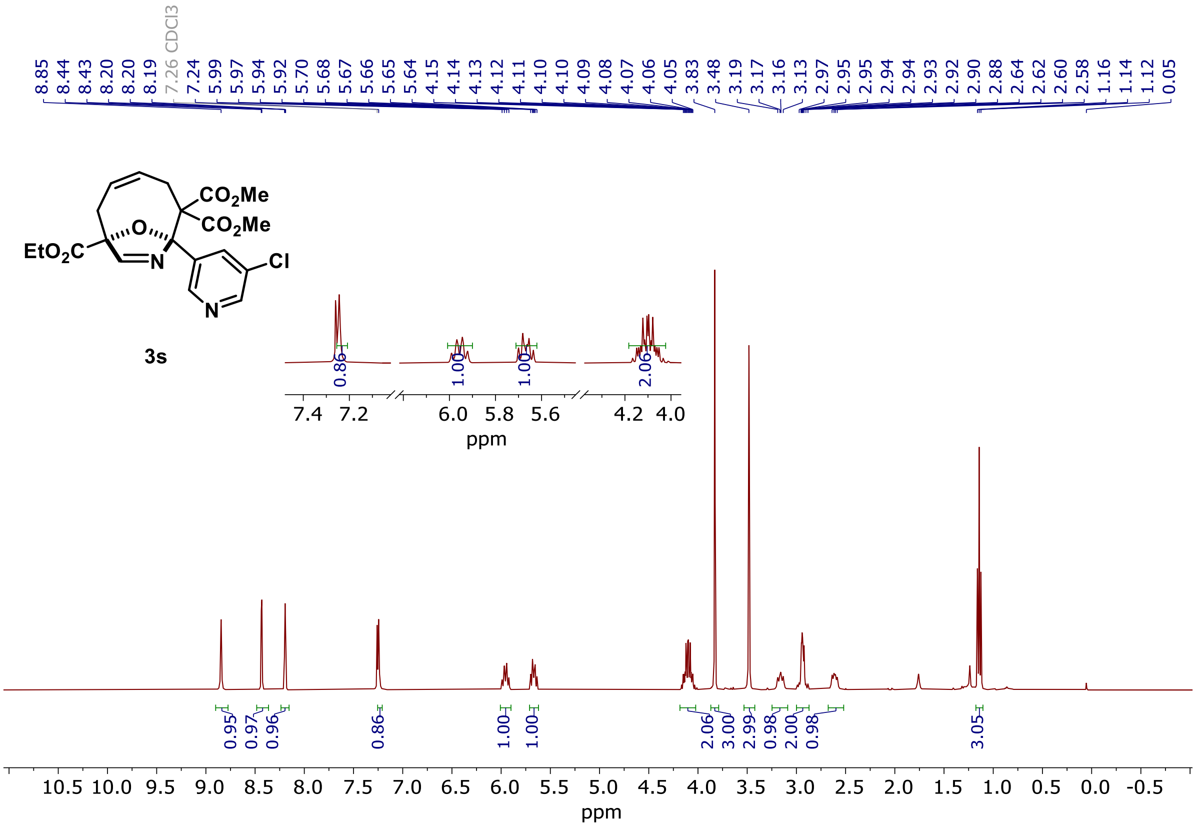

^{13}C NMR (101 MHz, $CDCl_3$) of **3s**

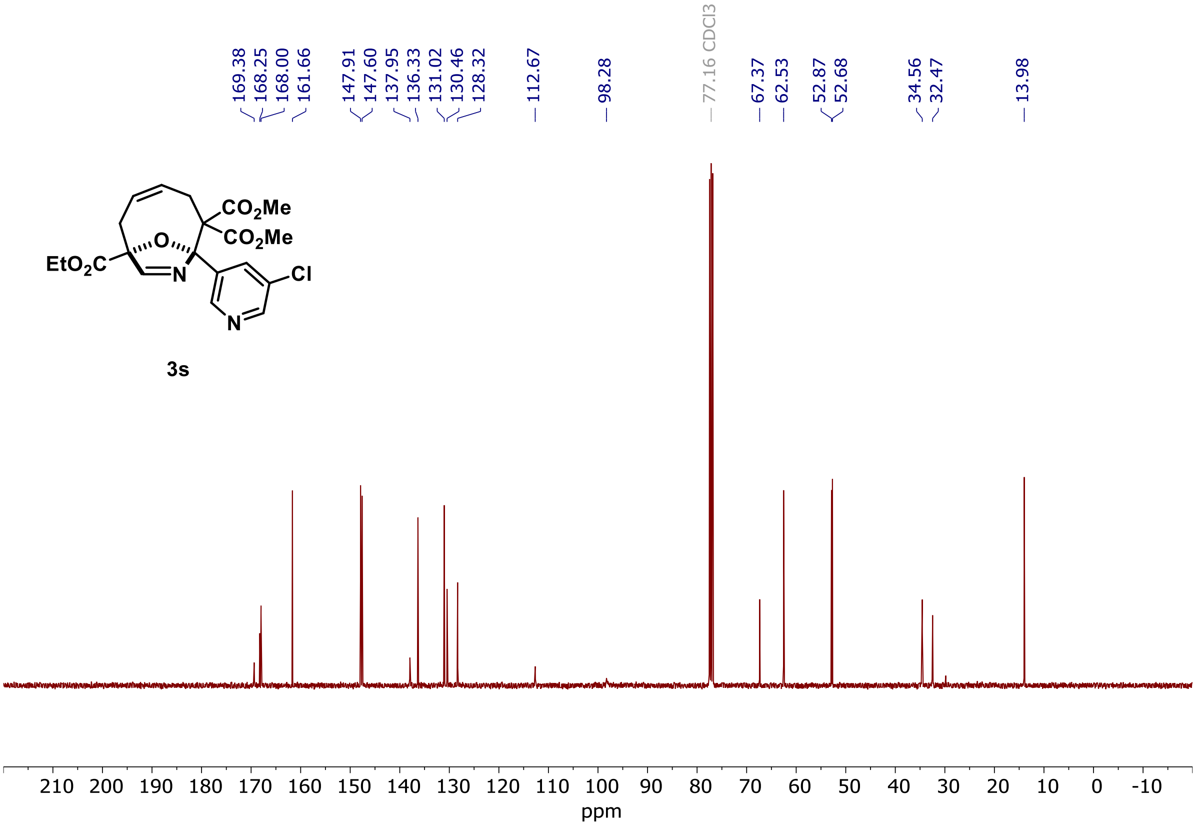

^{1}H NMR (400 MHz, $CDCl_3$) of **3t**

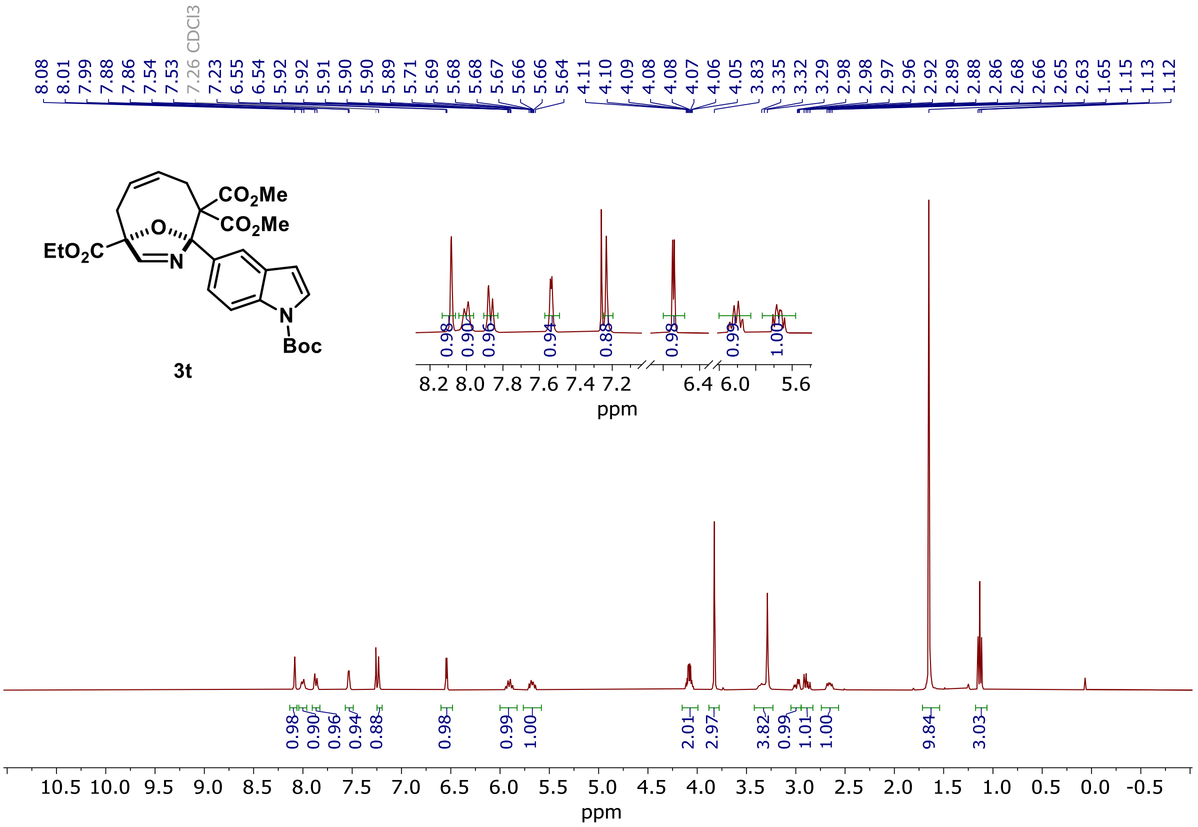

^{13}C NMR (101 MHz, $CDCl_3$) of **3t**

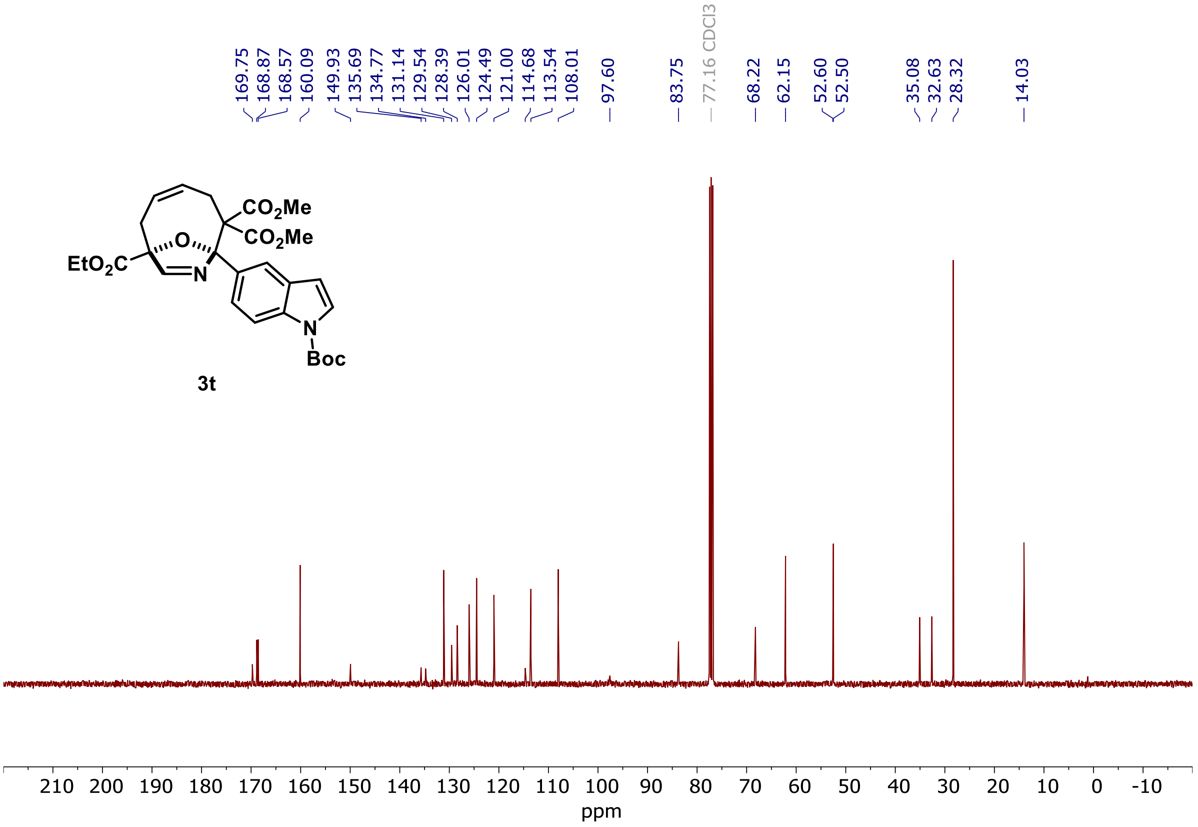

^{1}H NMR (400 MHz, $CDCl_3$) of **3u**

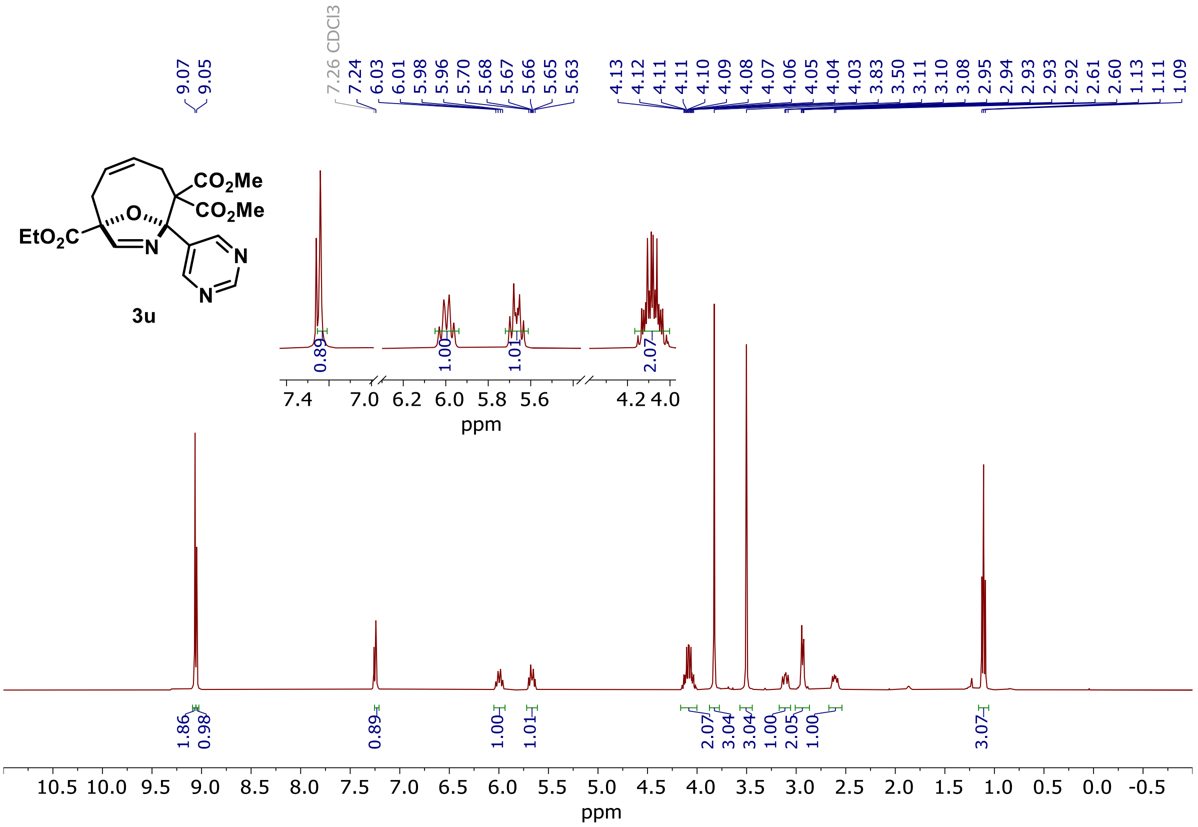

^{13}C NMR (101 MHz, $CDCl_3$) of **3u**

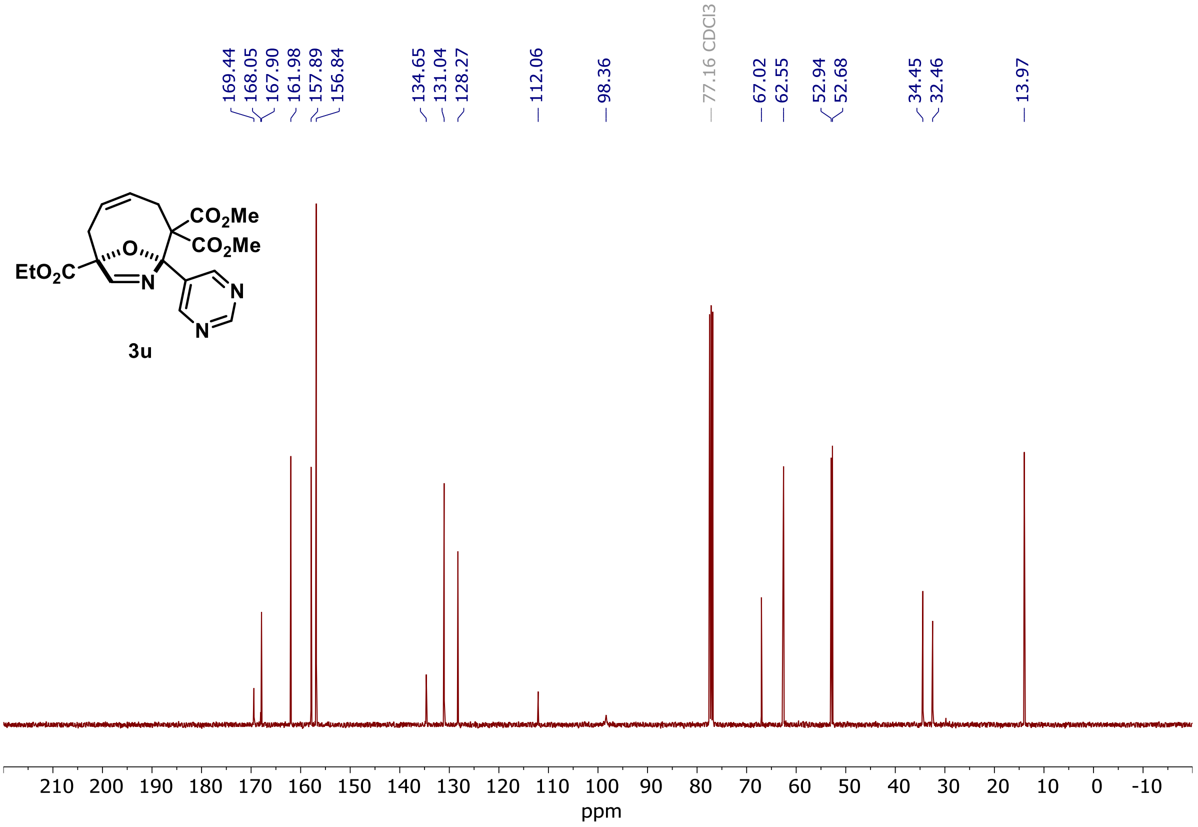

^{1}H NMR (400 MHz, $CDCl_3$) of **3v**

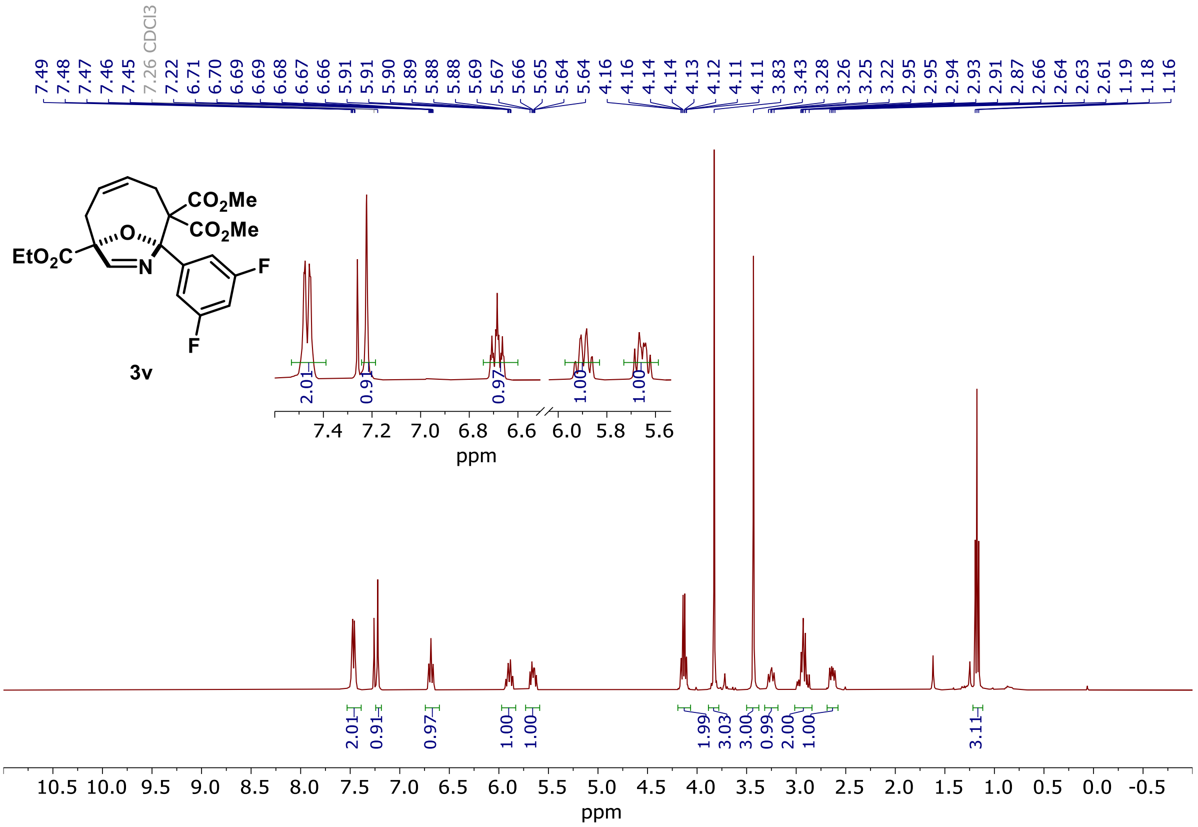

$^{13}C\{^{19}F\}$ NMR (151 MHz, $CDCl_3$) of **3v**

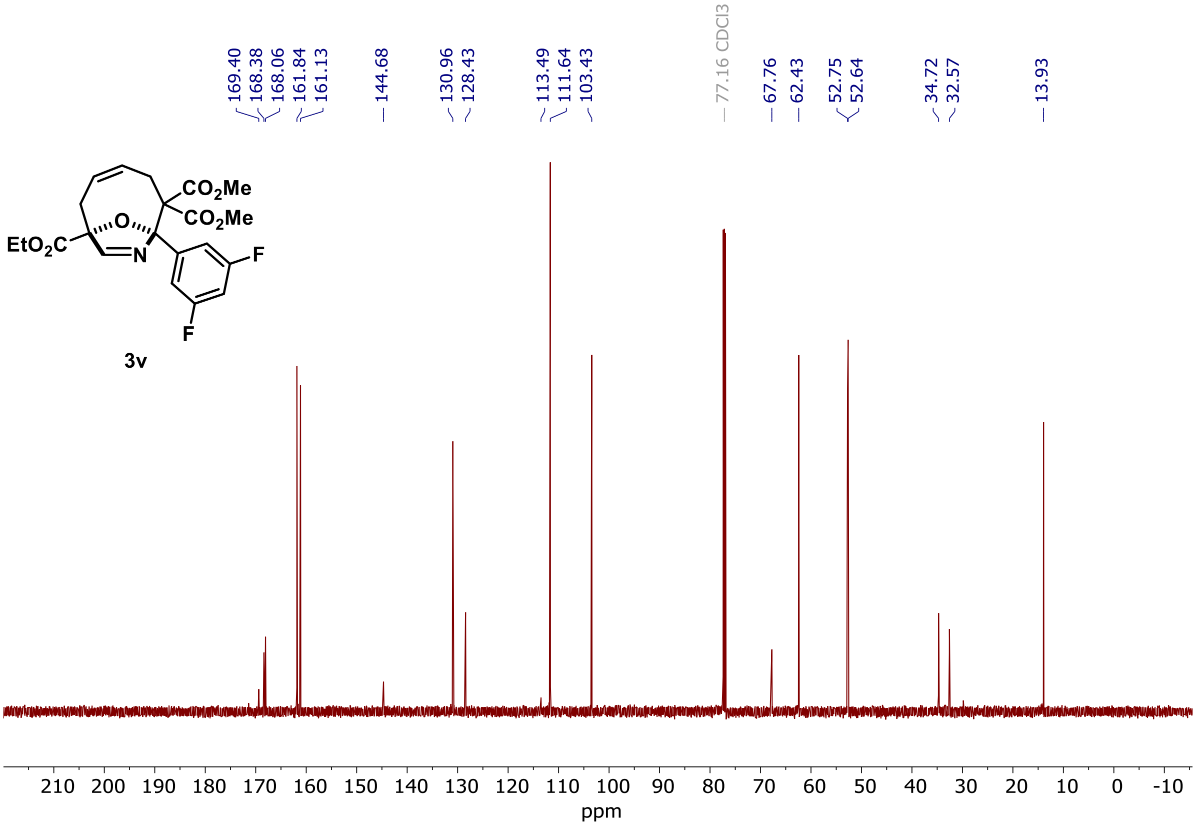

^{13}C NMR (151 MHz, $CDCl_3$) of **3v**

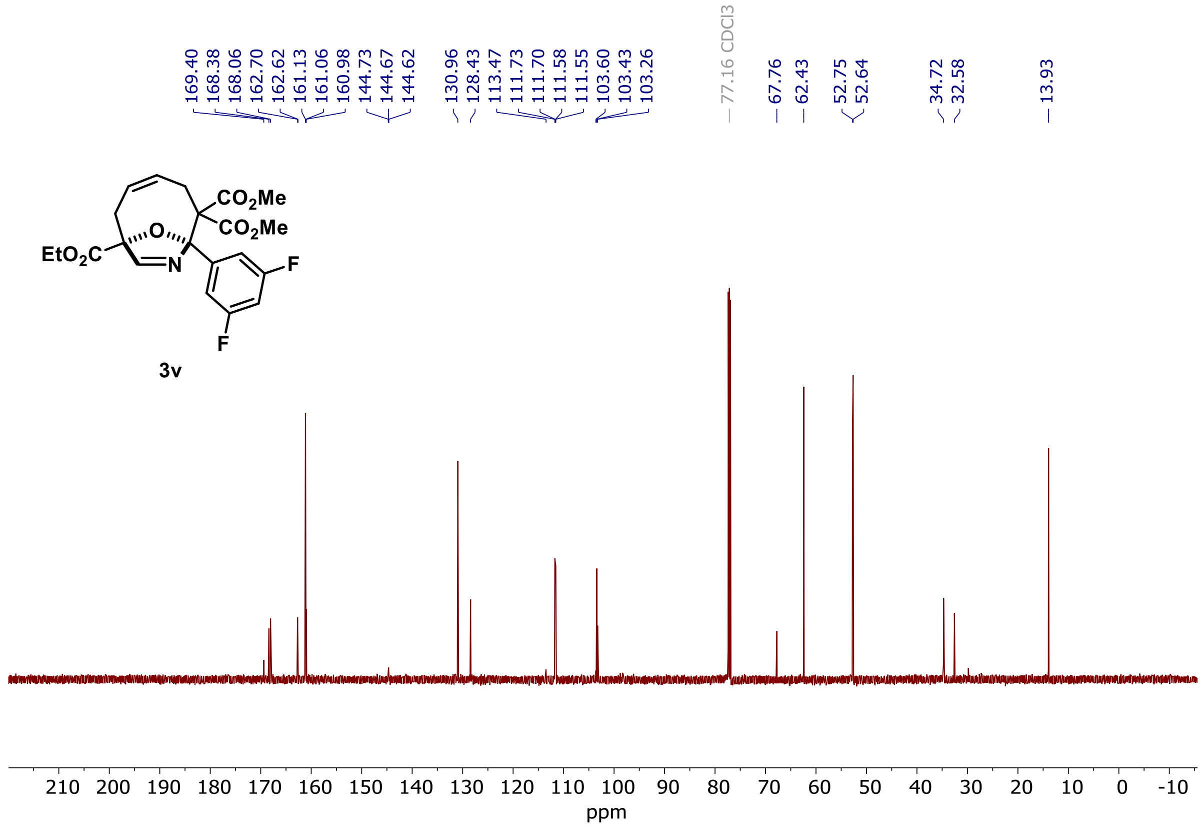

^{19}F NMR (563 MHz, $CDCl_3$) of **3v**

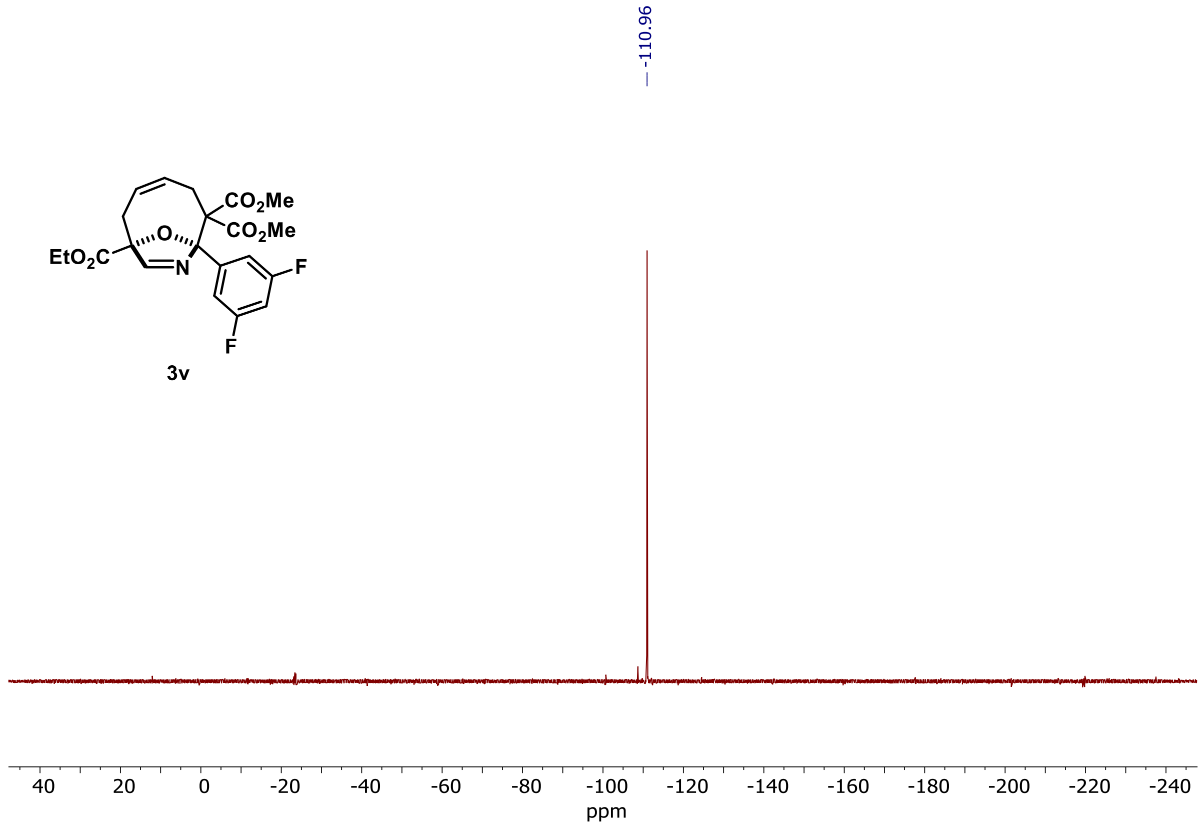

^{1}H NMR (400 MHz, $CDCl_3$) of **3w** & **3w’**

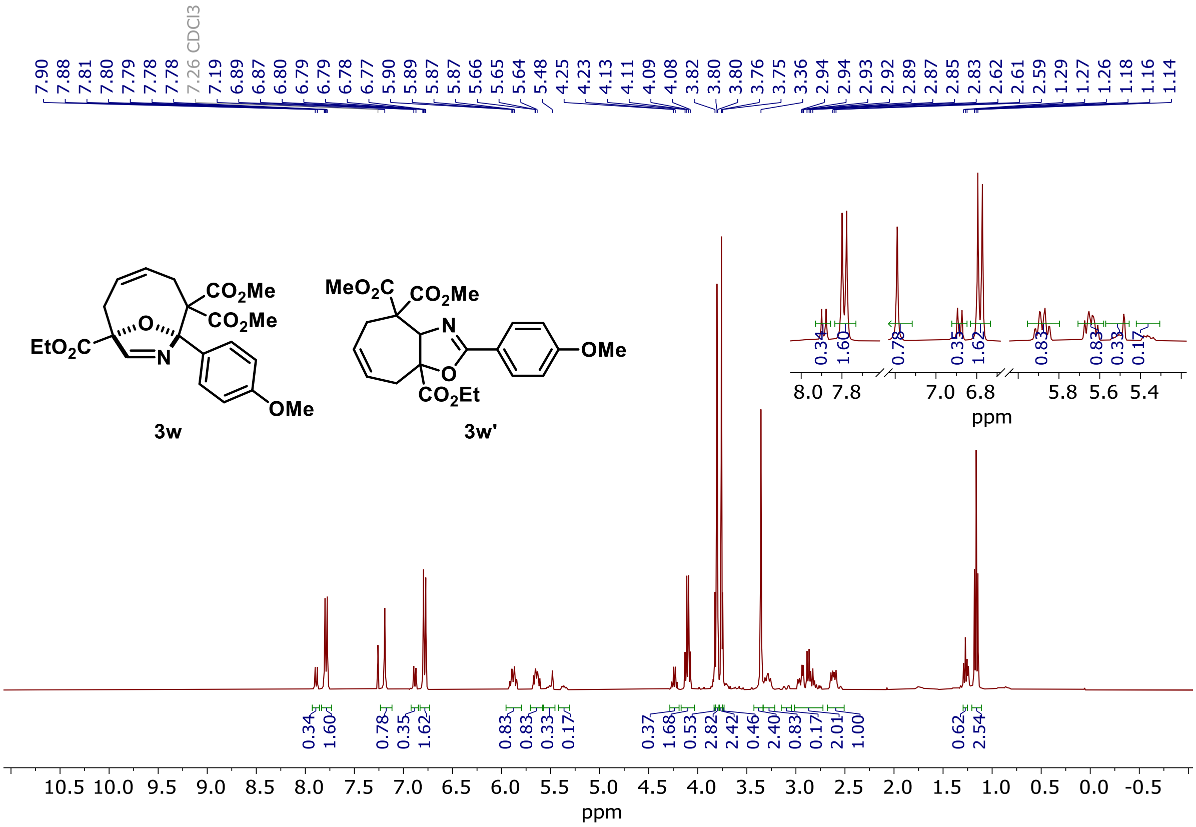

^{13}C NMR (101 MHz, $CDCl_3$) of **3w** & **3w‘**

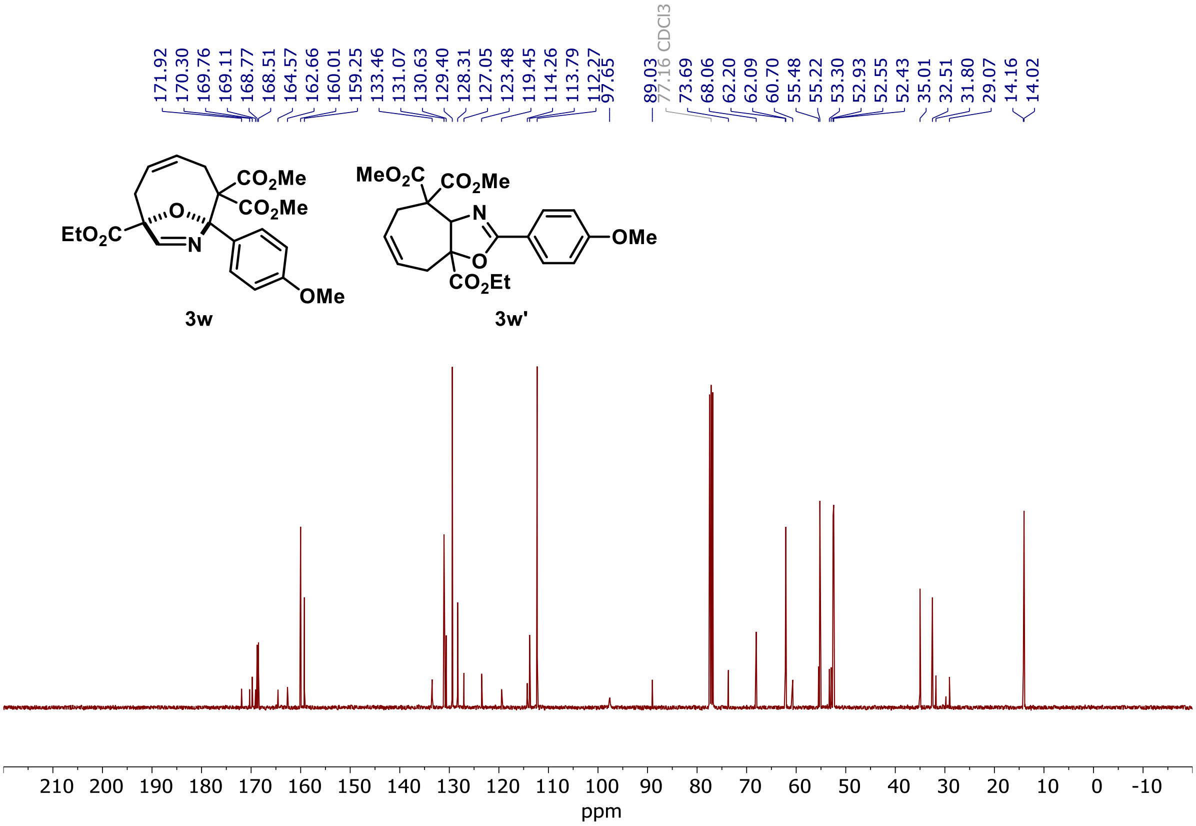

^{1}H NMR (400 MHz, $CDCl_3$) of **3x**

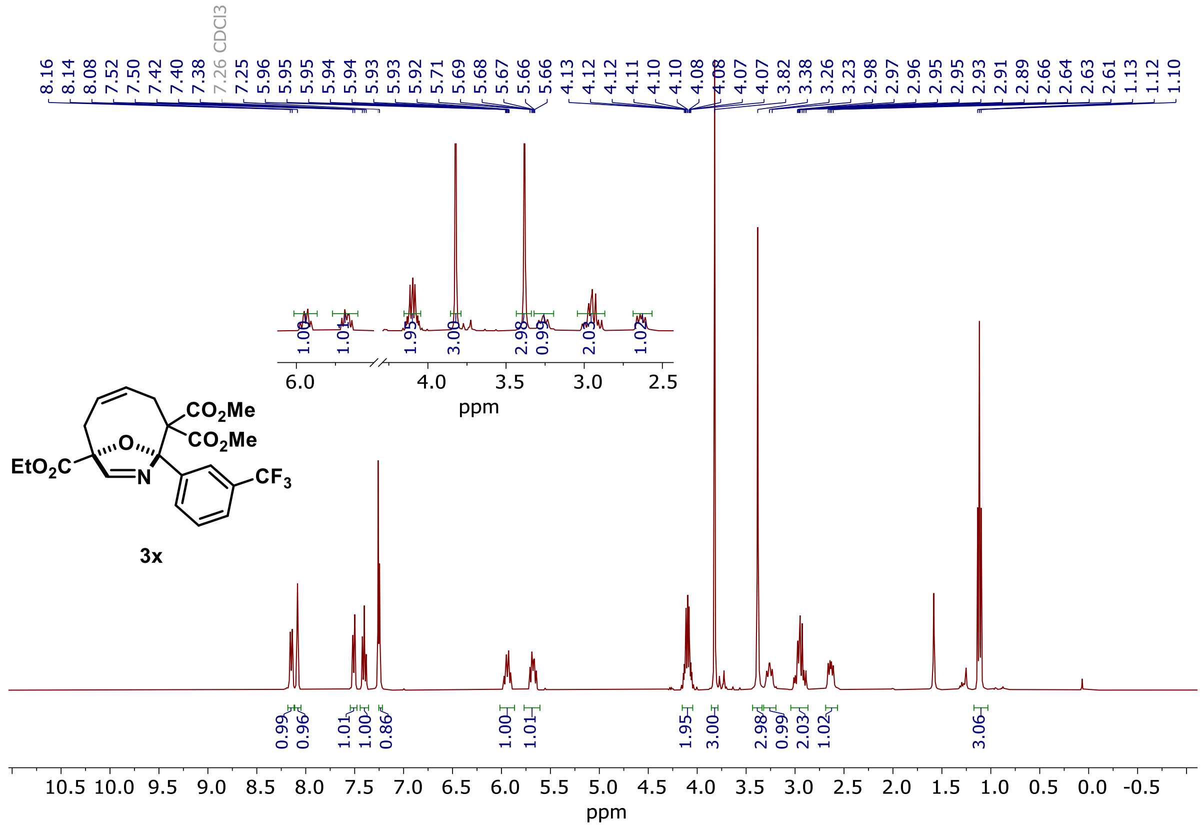

$^{13}C\{^{19}F\}$ NMR (126 MHz, $CDCl_3$) of **3x**

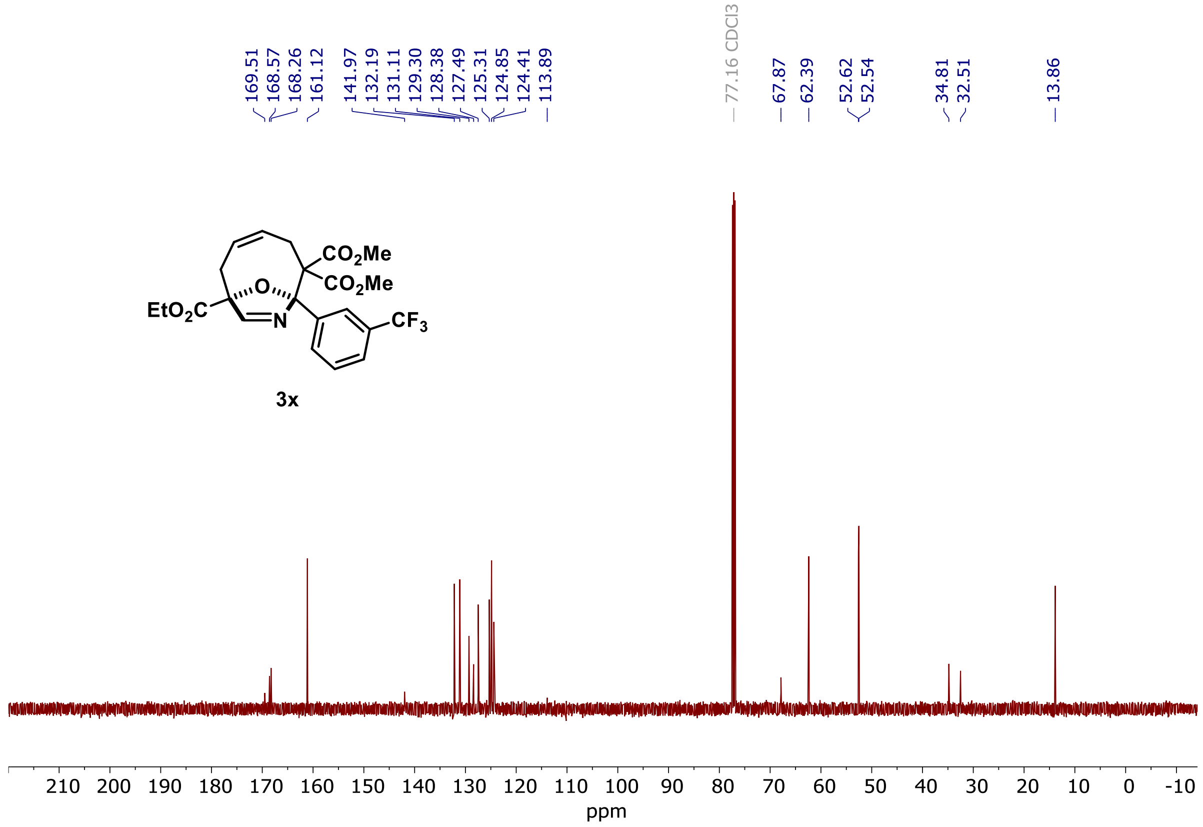

^{13}C NMR (126 MHz, $CDCl_3$) of **3x**

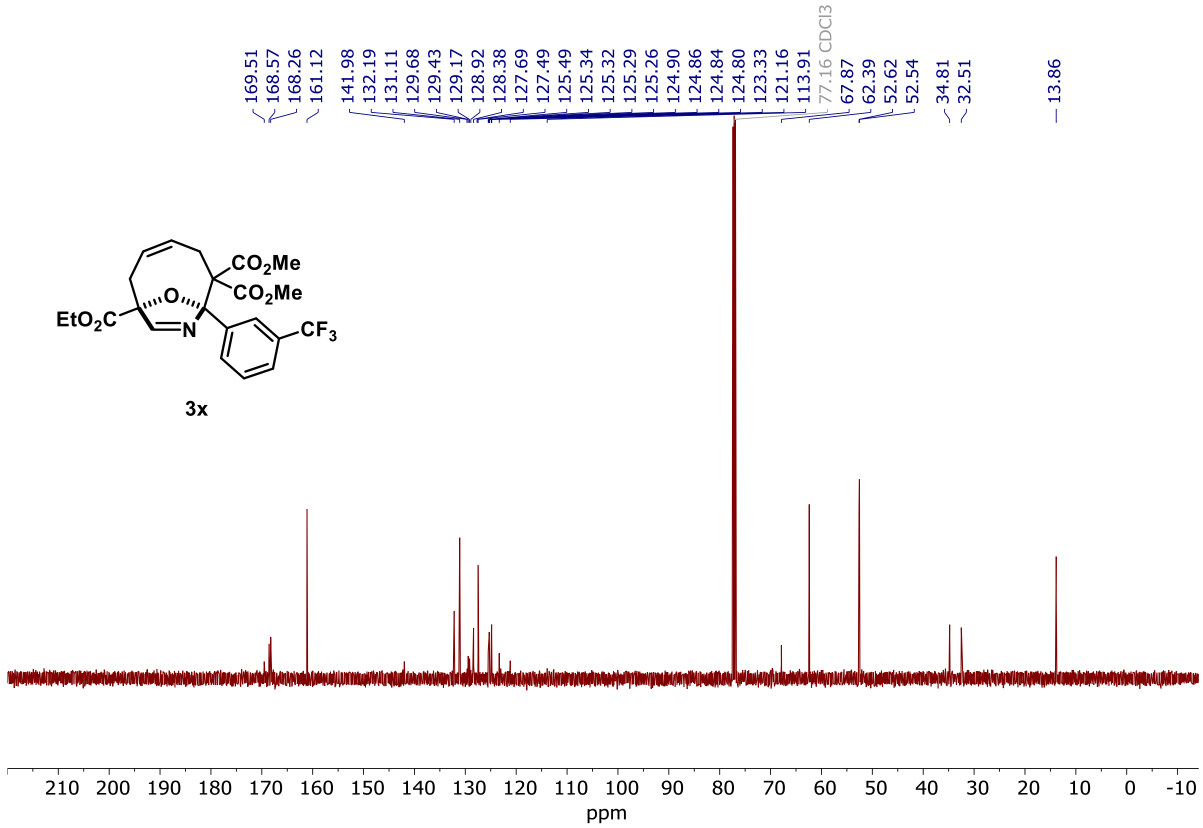

^{19}F NMR (376 MHz, $CDCl_3$) of **3x**

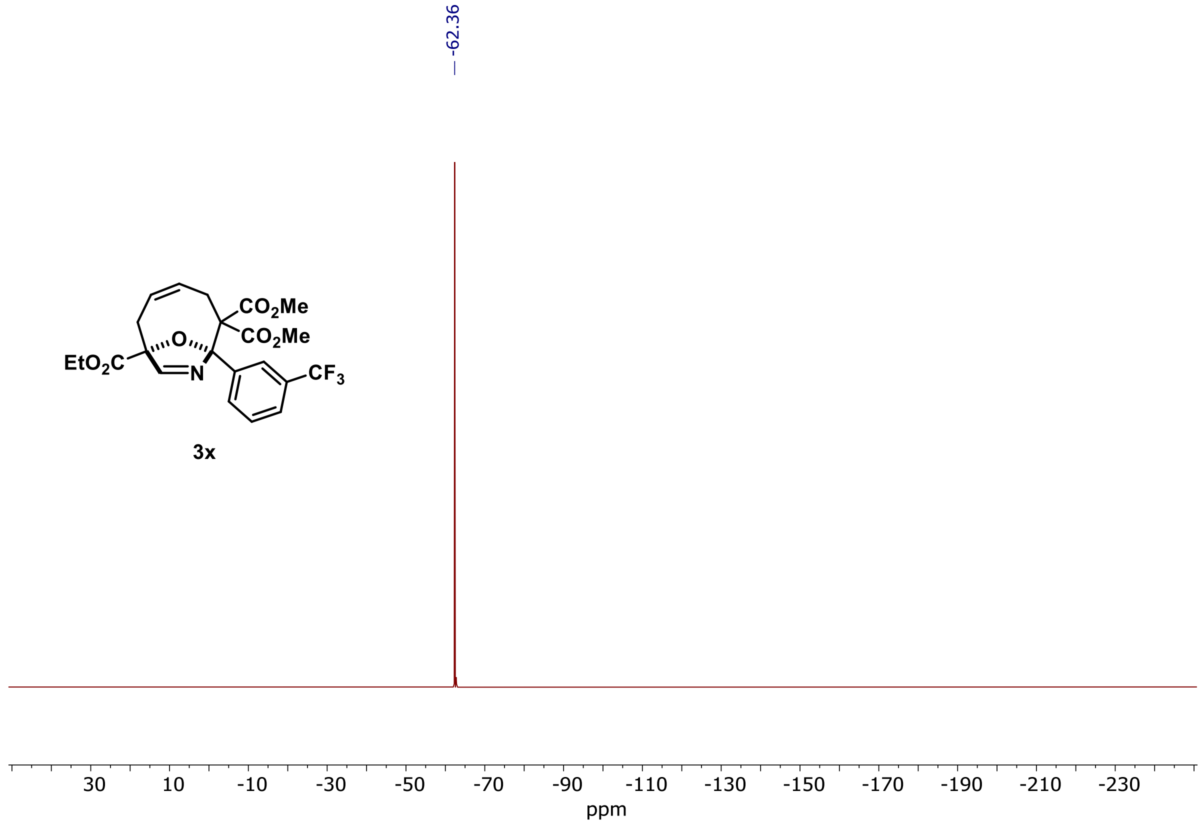

^{1}H NMR (400 MHz, $CDCl_3$) of **3y**

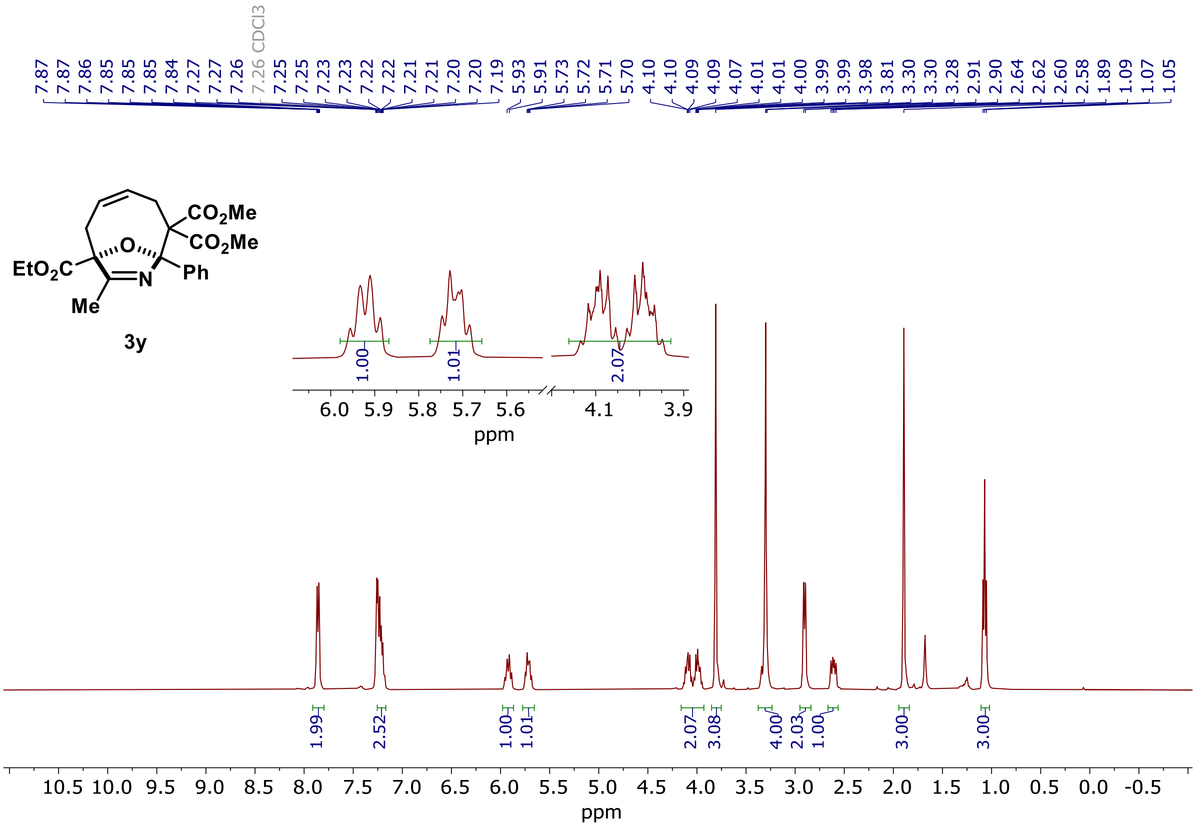

^{13}C NMR (101 MHz, $CDCl_3$) of **3y**

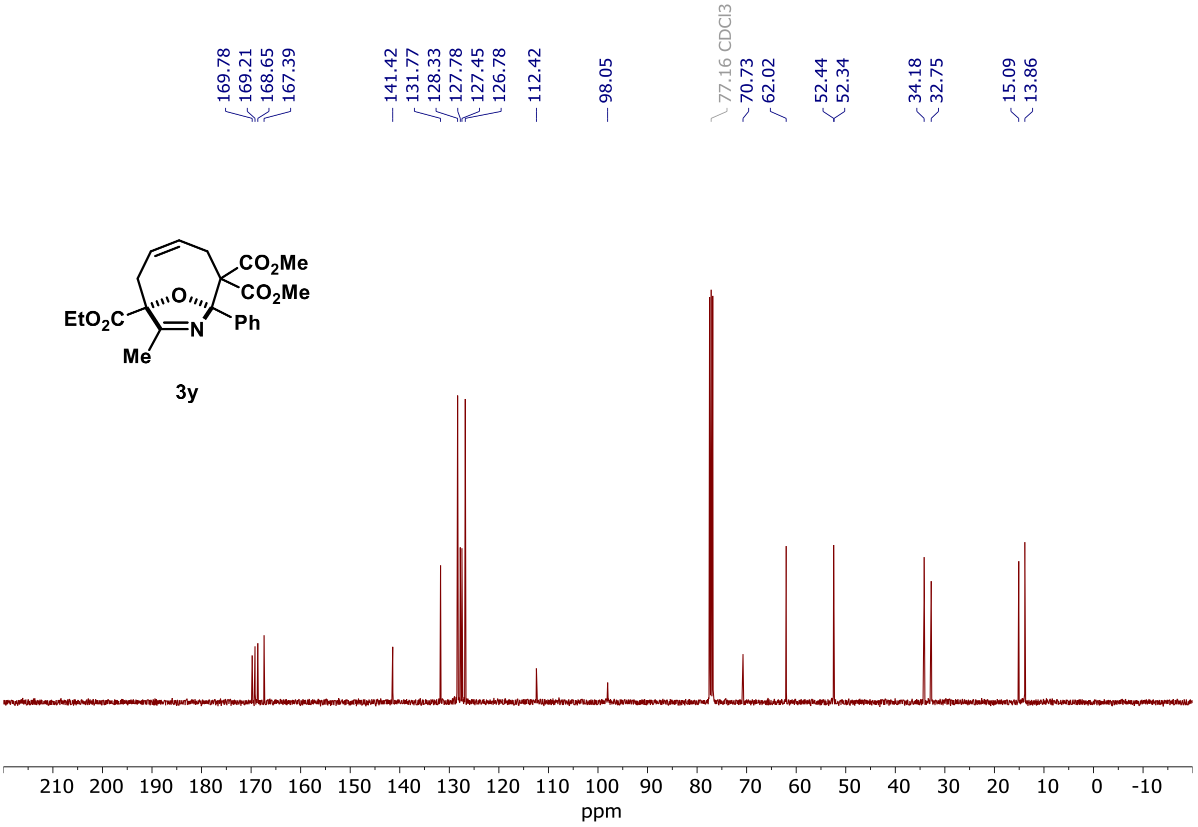

^{1}H NMR (400 MHz, $CDCl_3$) of **3z**

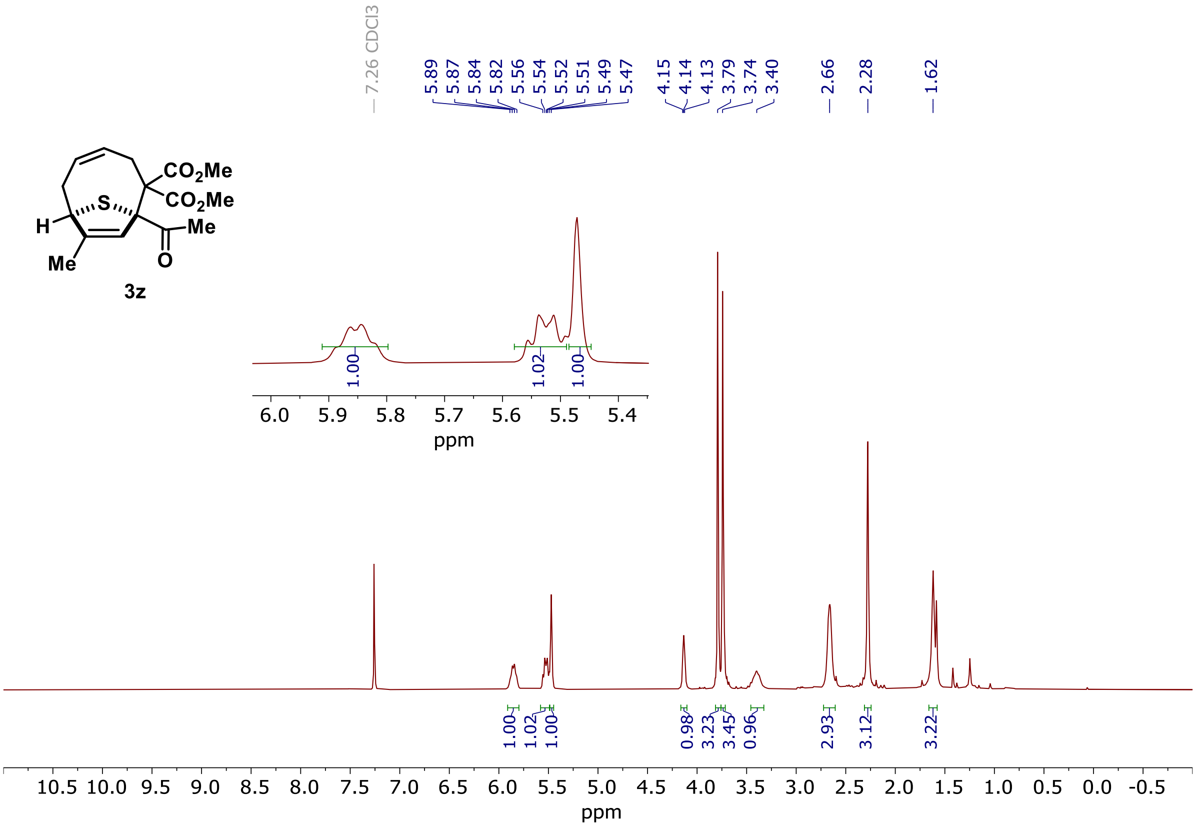

^{13}C NMR (101 MHz, $CDCl_3$) of **3z**

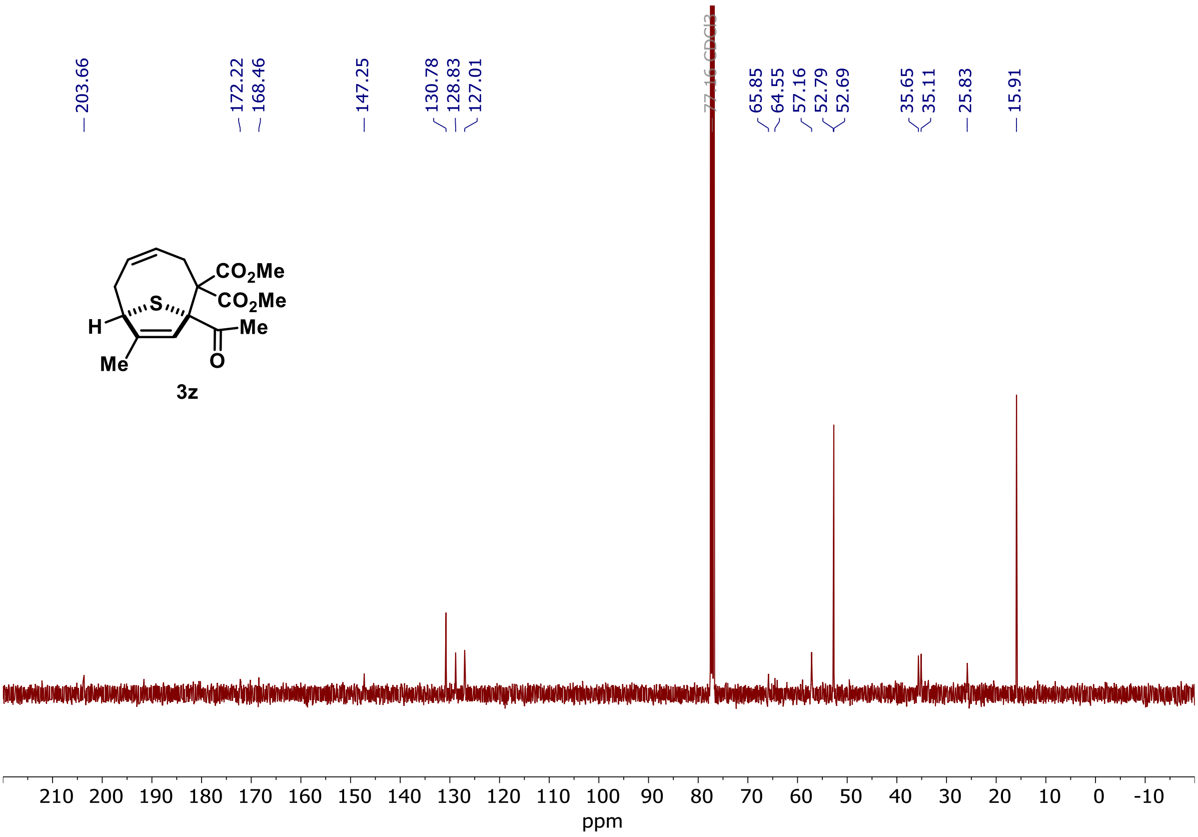

^{1}H NMR (400 MHz, $CDCl_3$) of **3aa**

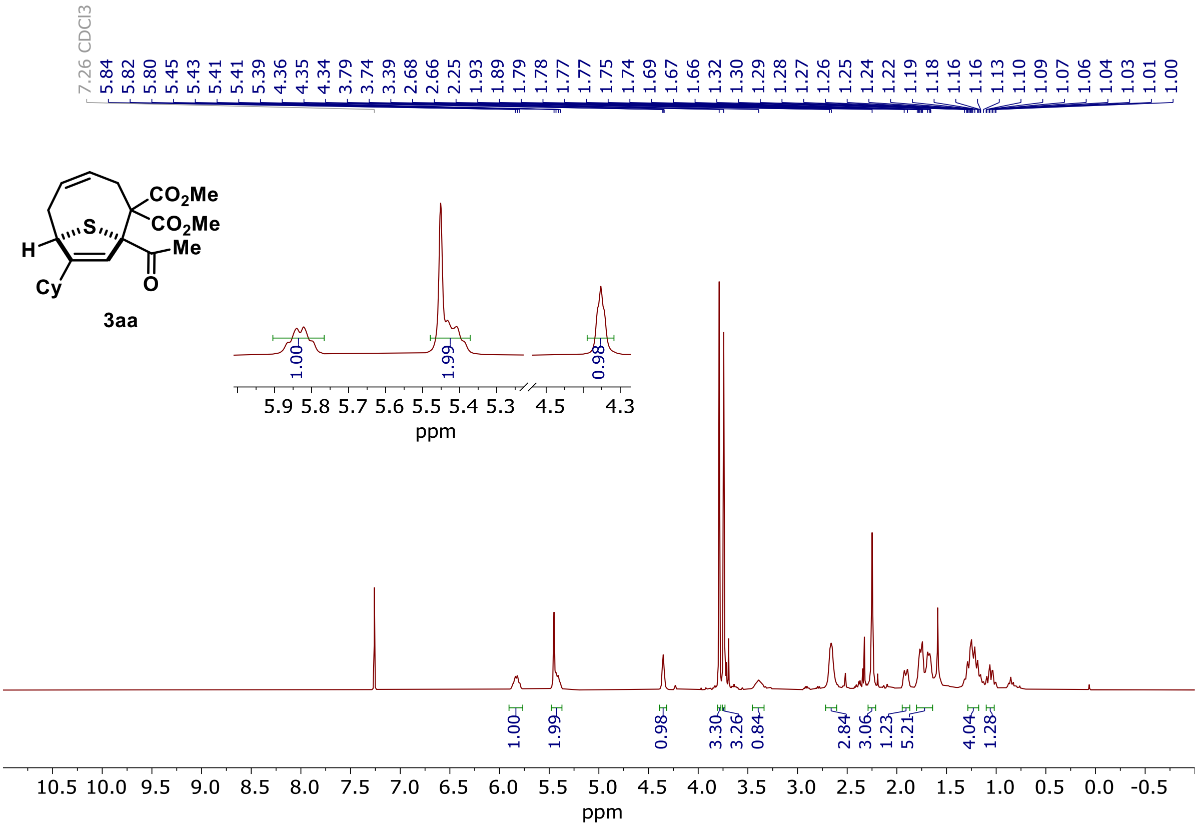

^{13}C NMR (101 MHz, $CDCl_3$) of **3aa**

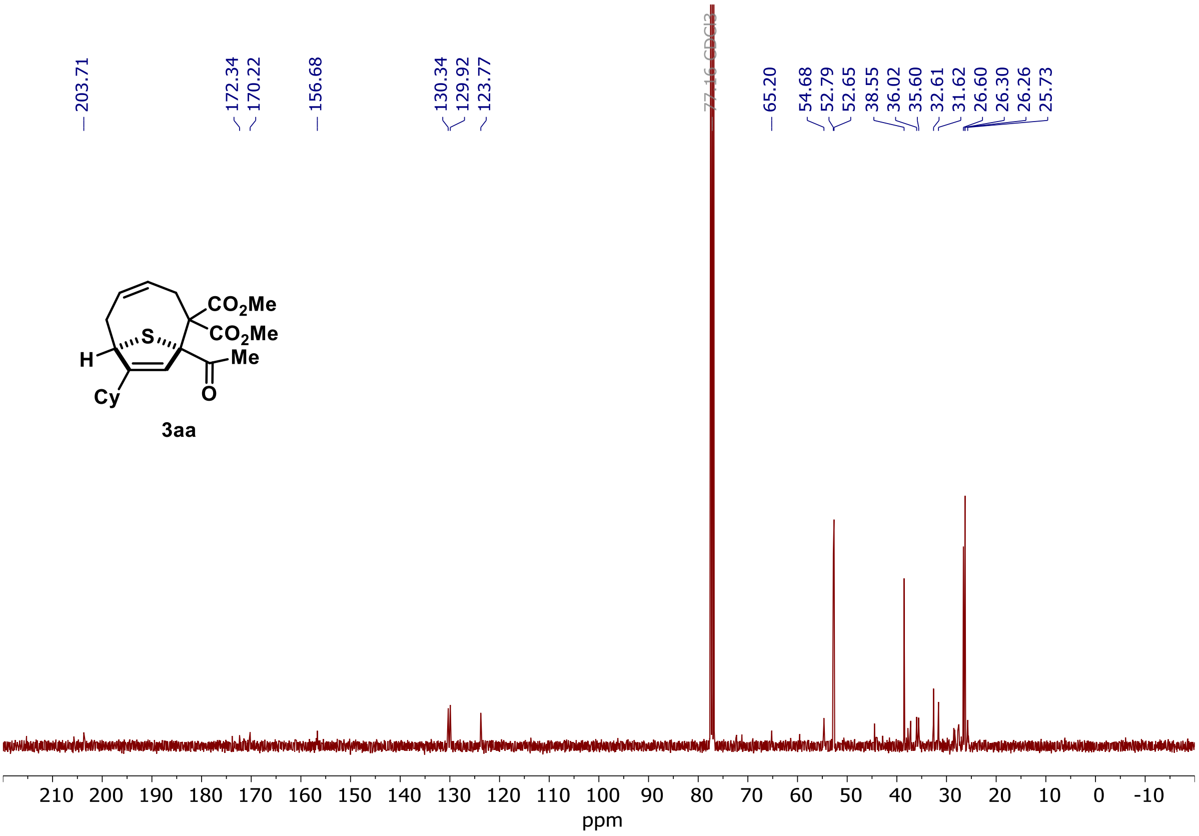

^{1}H NMR (400 MHz, $CDCl_3$) of **3ab**

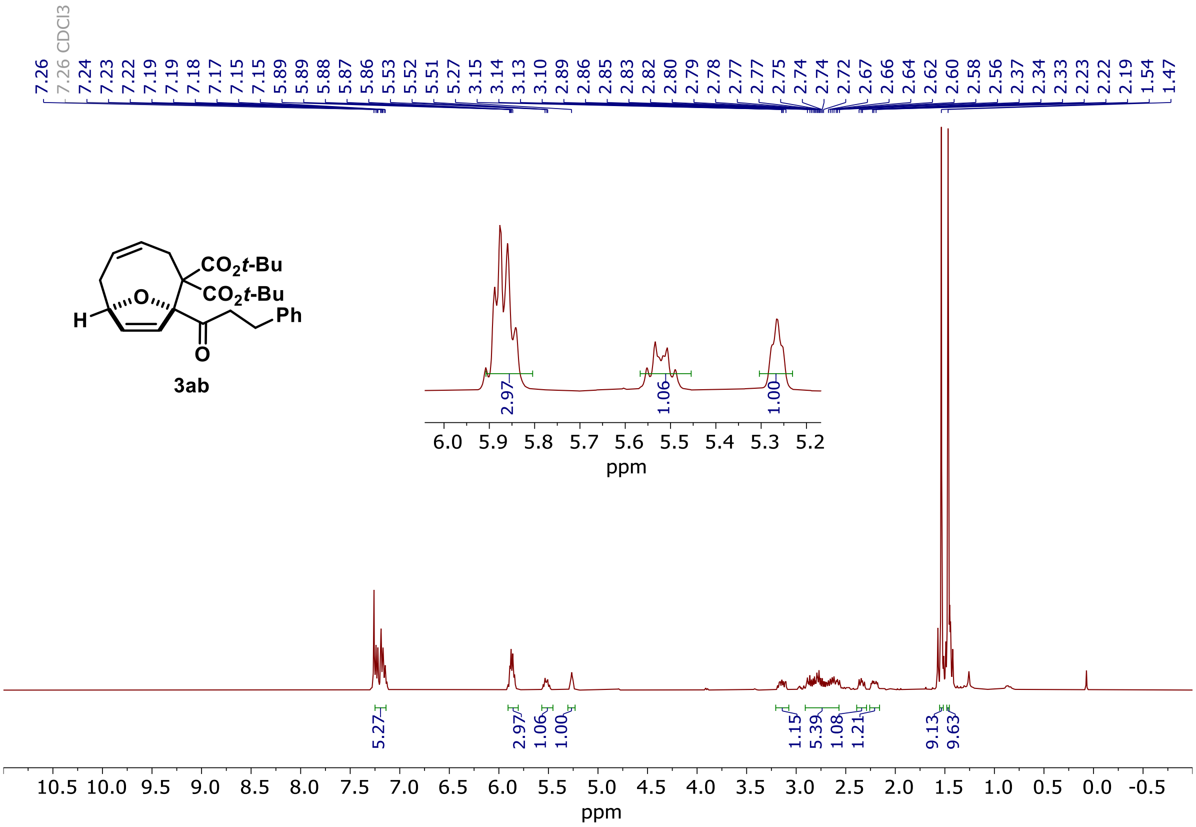

^{13}C NMR (101 MHz, $CDCl_3$) of **3ab**

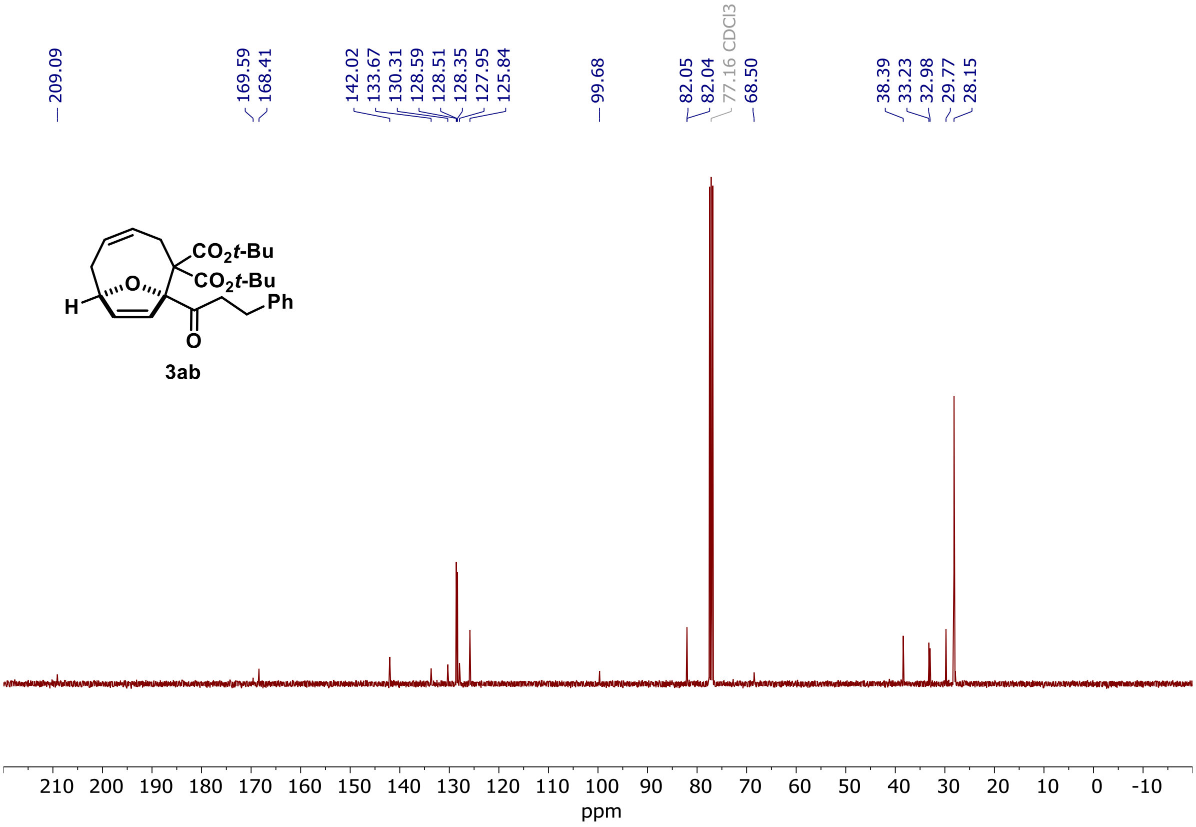

^{1}H NMR (400 MHz, $CDCl_3$) of **3ab'**

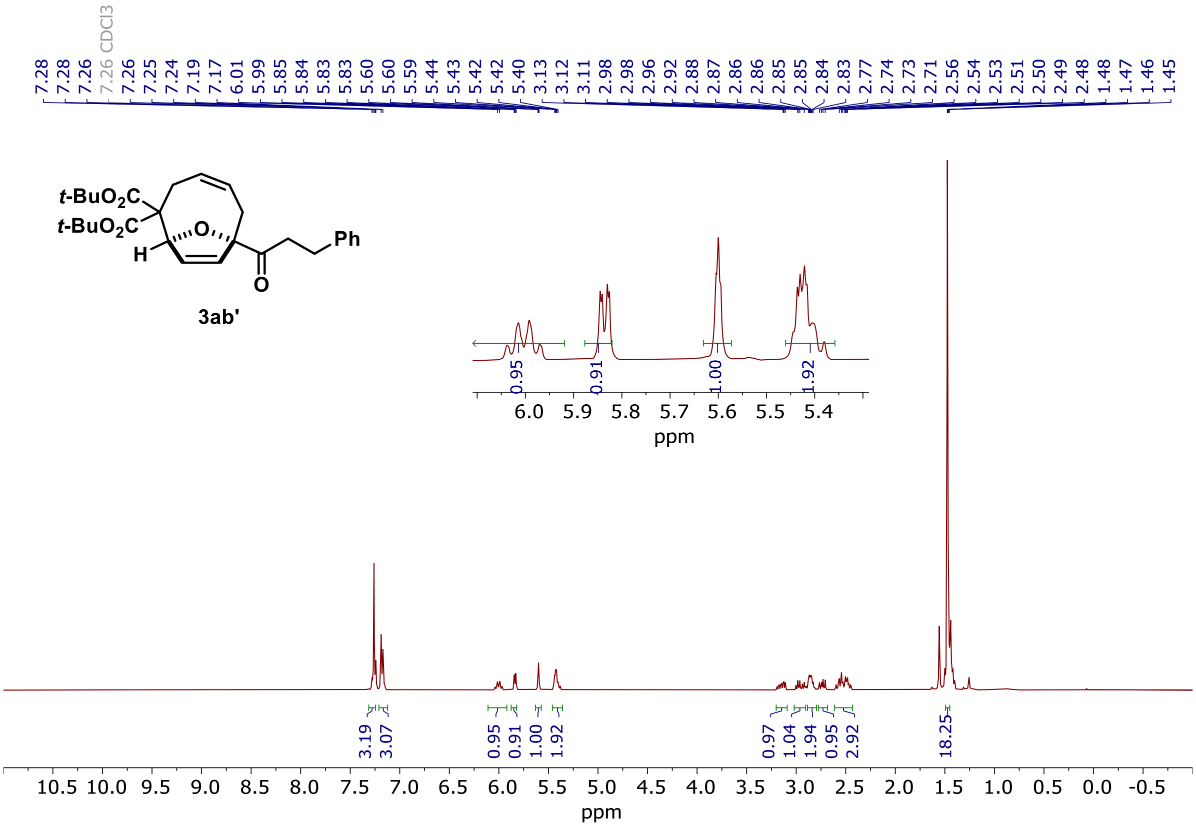

^{13}C NMR (151 MHz, $CDCl_3$) of **3ab'**

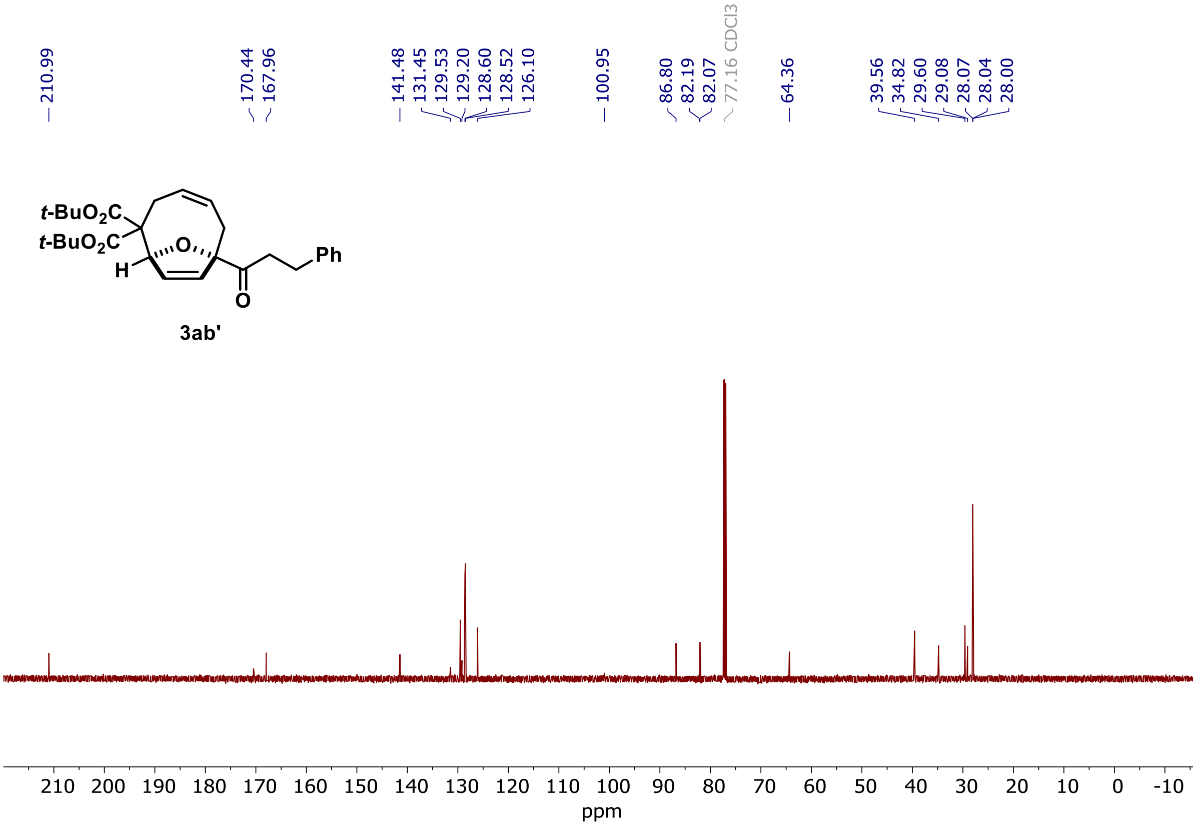

^{1}H NMR (400 MHz, $CDCl_3$) of **3ac**

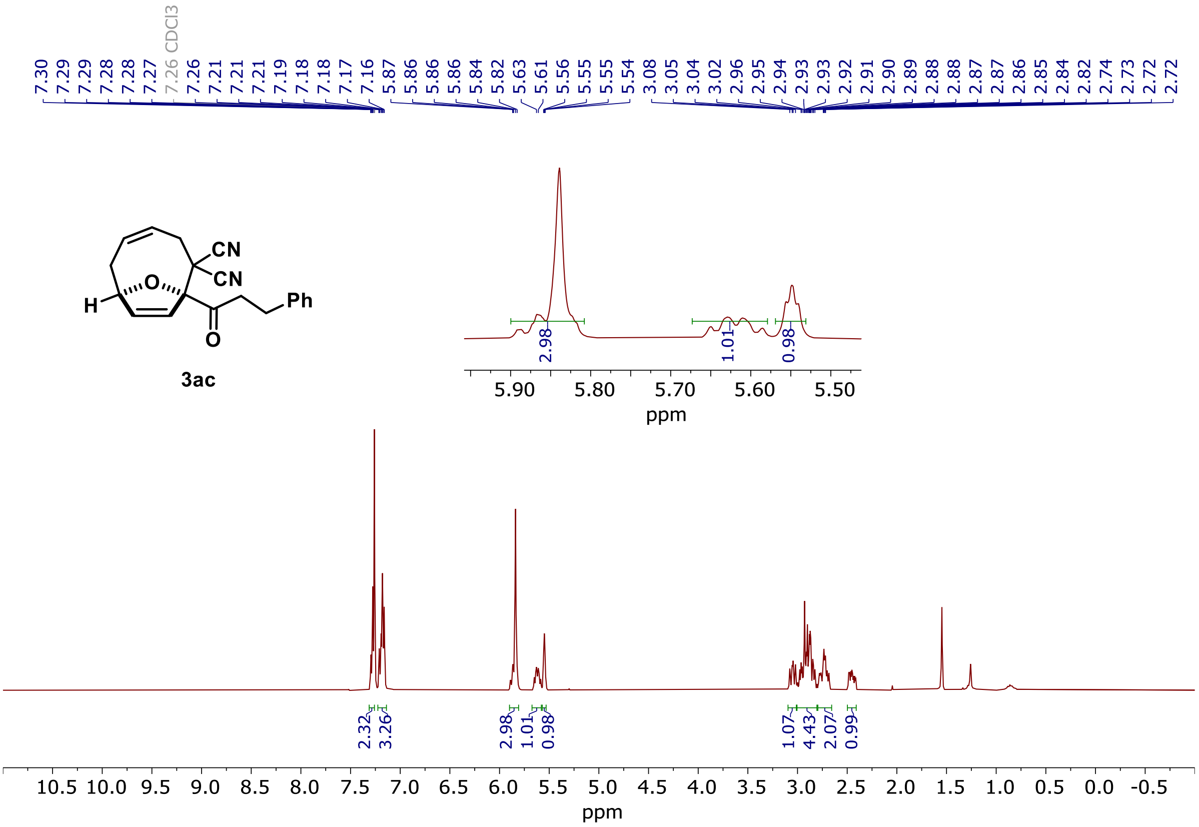

^{13}C NMR (101 MHz, $CDCl_3$) of **3ac**

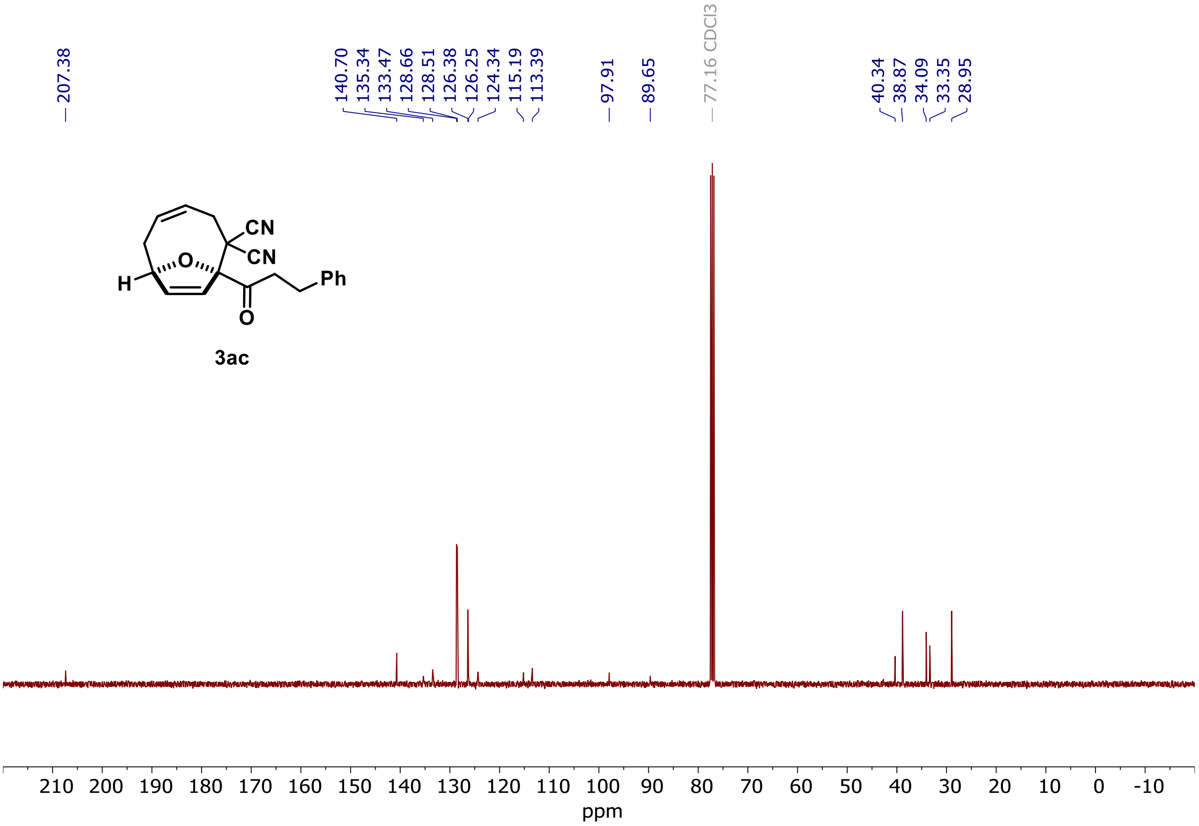

^{1}H NMR (599 MHz, CDCl$_3$) of **3ad d$_1$**

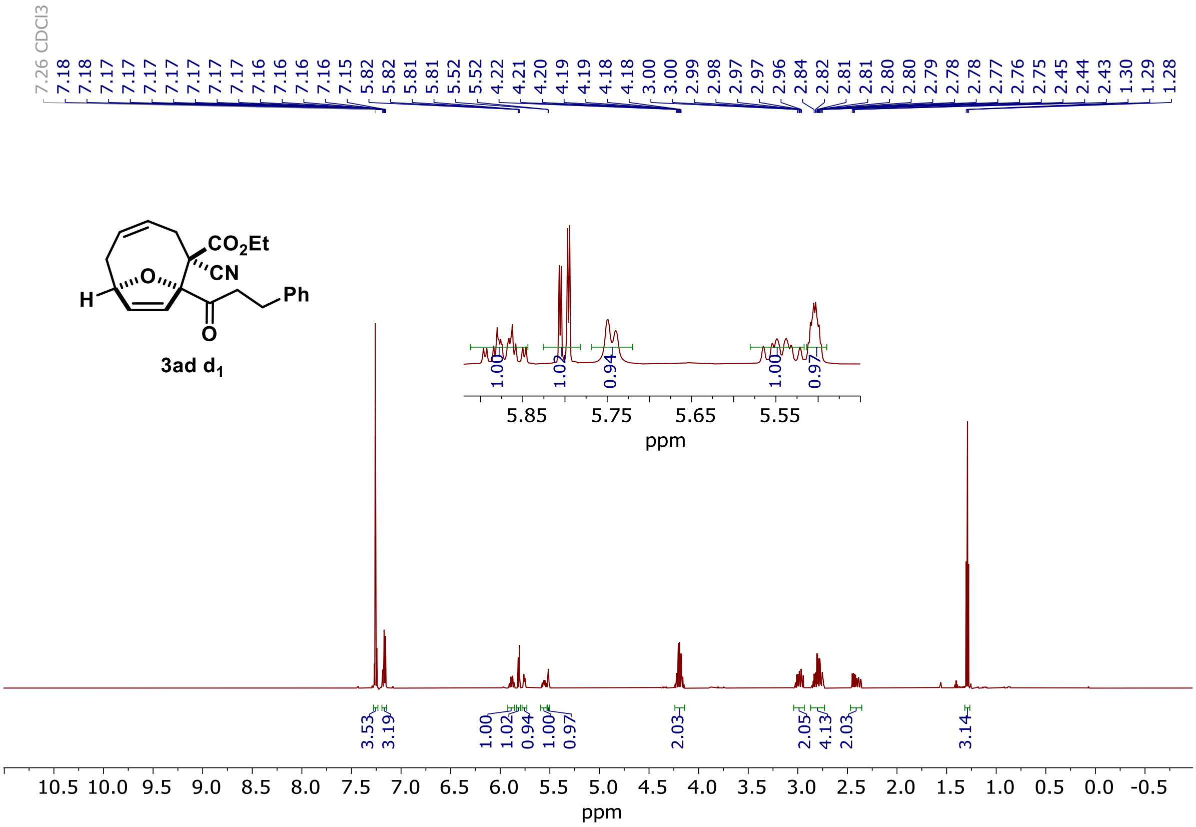

^{13}C NMR (151 MHz, CDCl$_3$) of **3ad d$_1$**

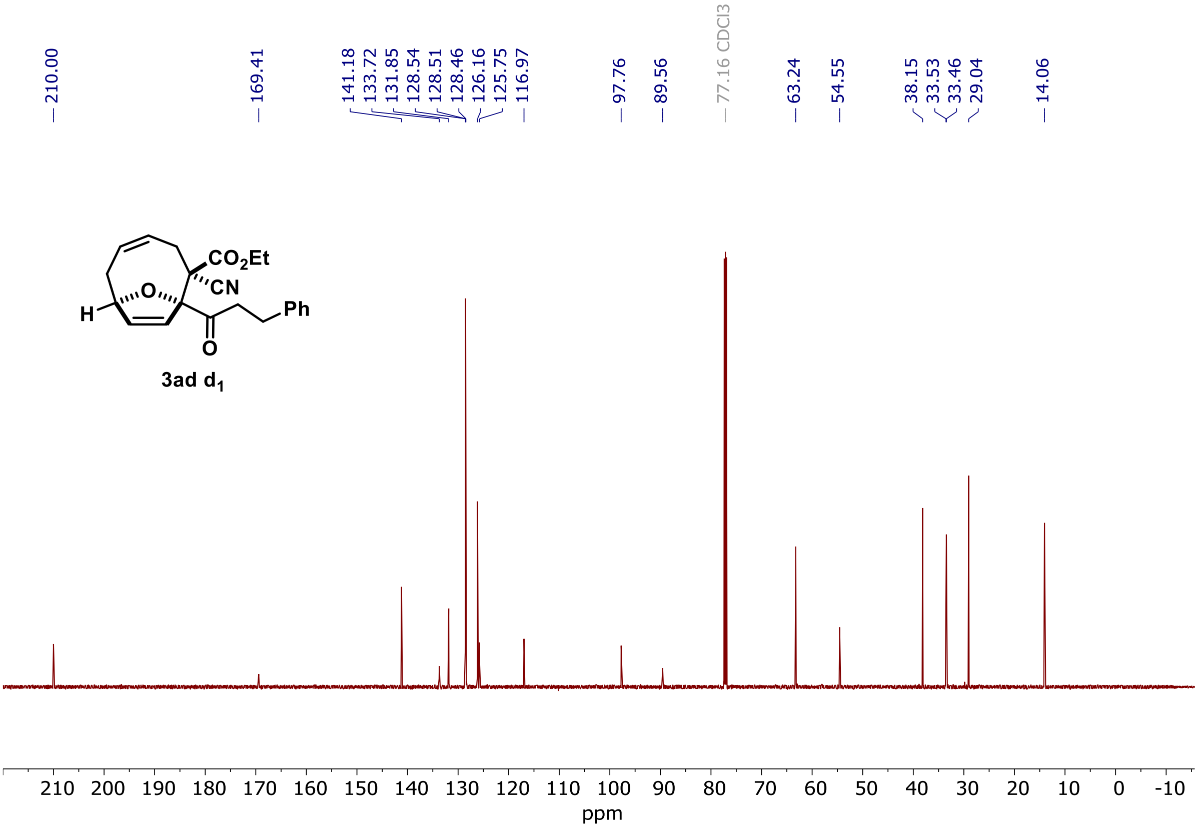

^{1}H NMR (599 MHz, $CDCl_3$) of **3ad d$_2$**

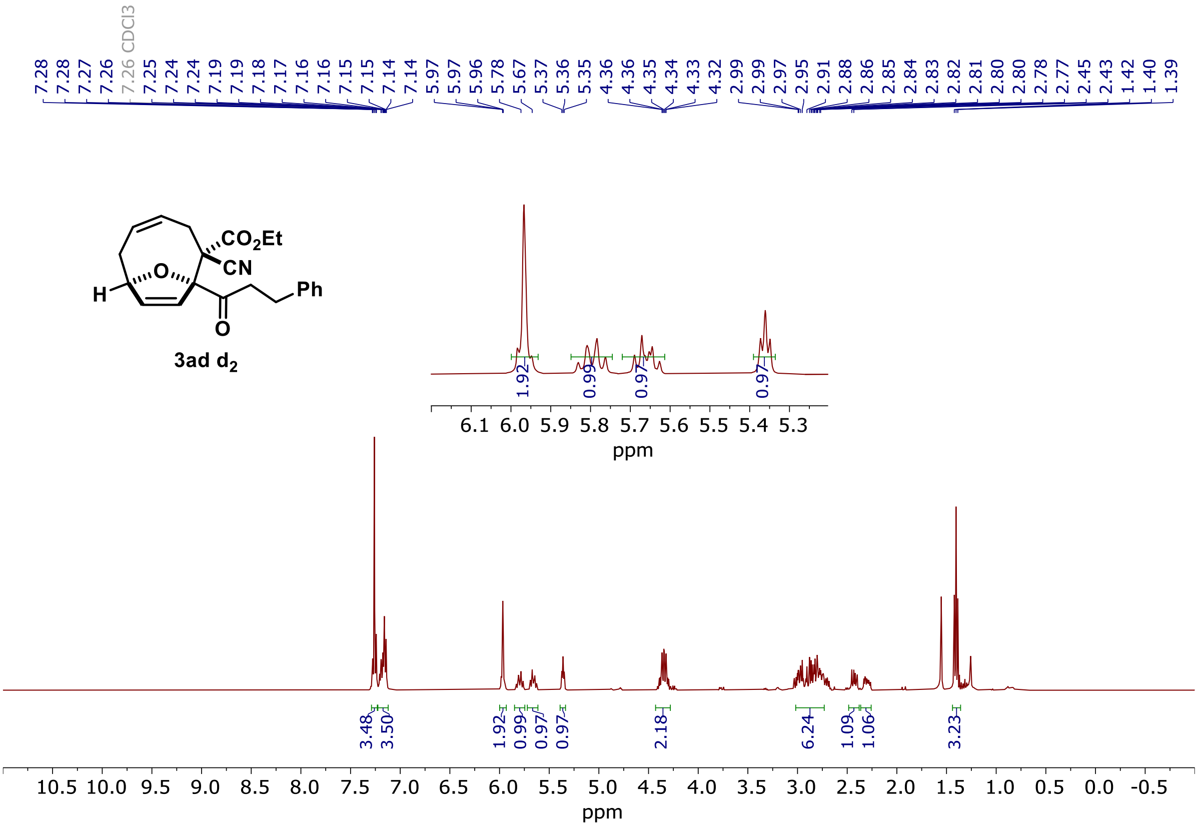

3ad d$_2$

^{13}C NMR (151 MHz, $CDCl_3$) of **3ad d$_2$**

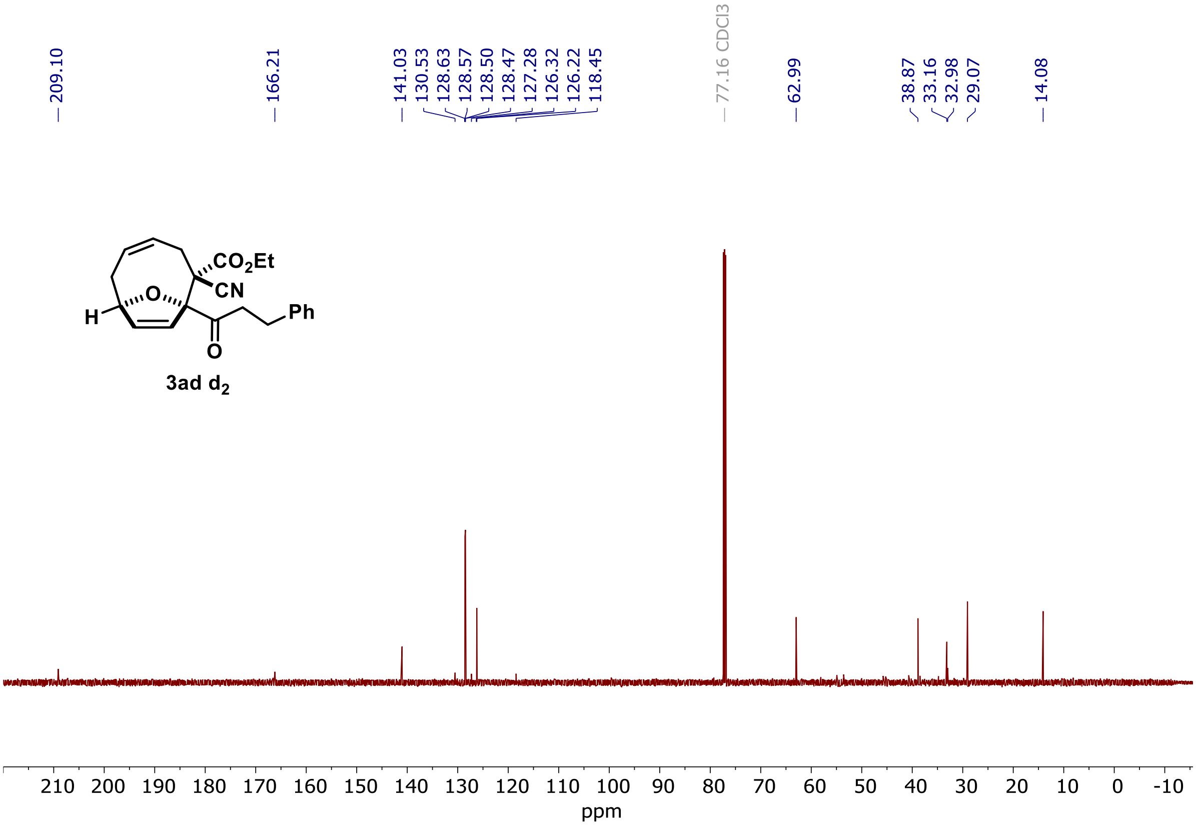

3ad d$_2$

^{1}H NMR (599 MHz, $CDCl_3$) of **3ad'**

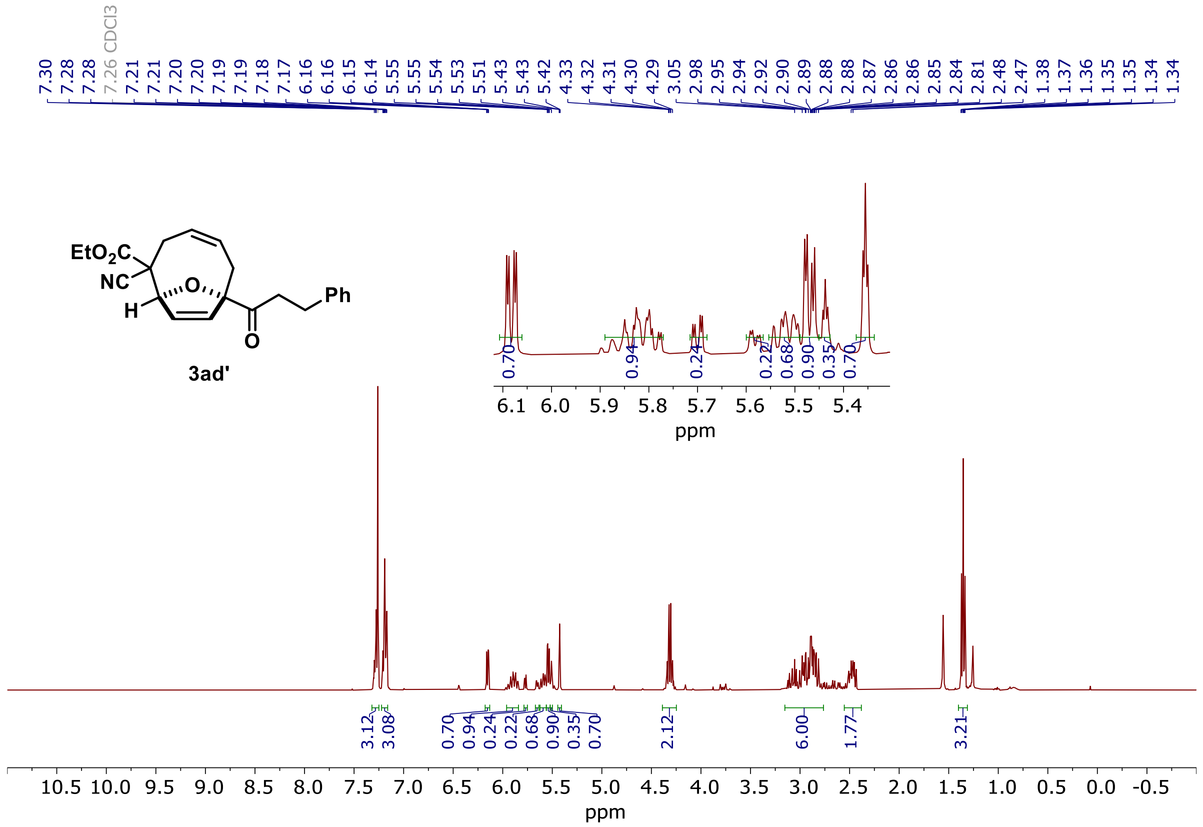

^{13}C NMR (151 MHz, $CDCl_3$) of **3ad'**

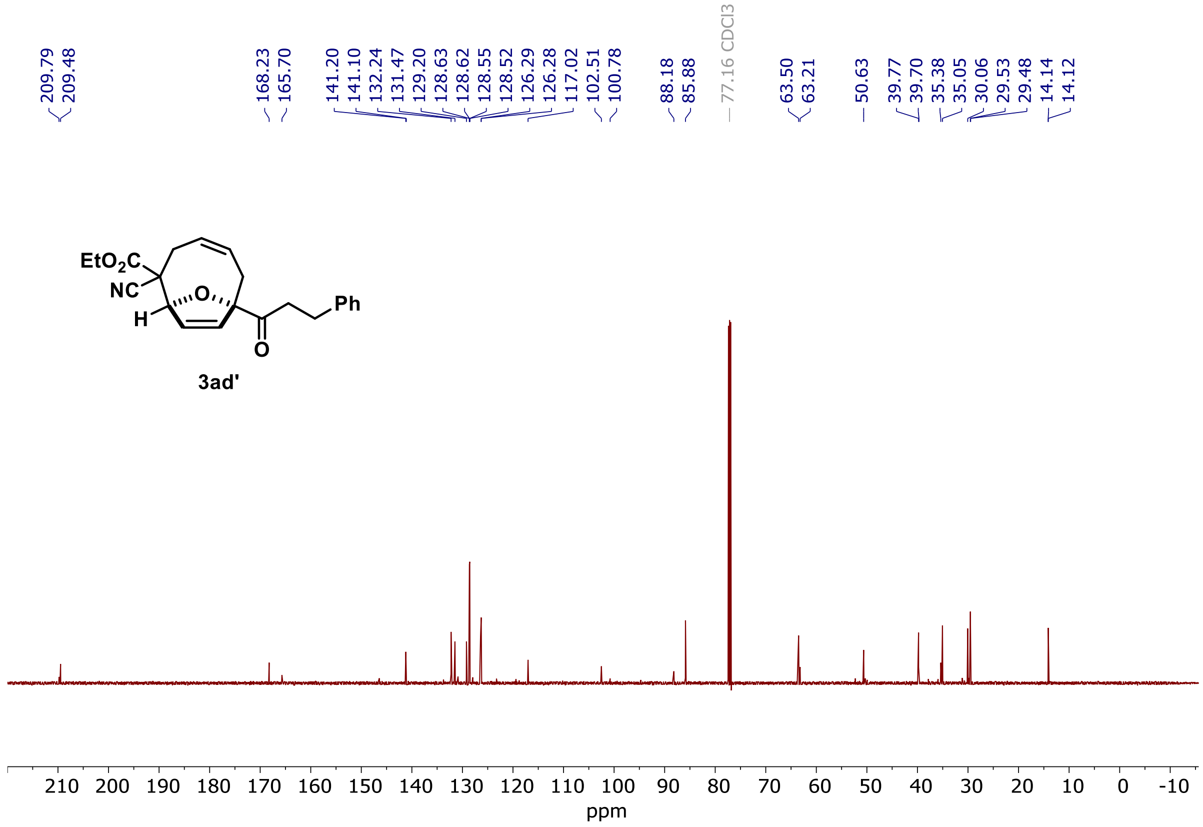

^{1}H NMR (400 MHz, $CDCl_3$) of **3ae**

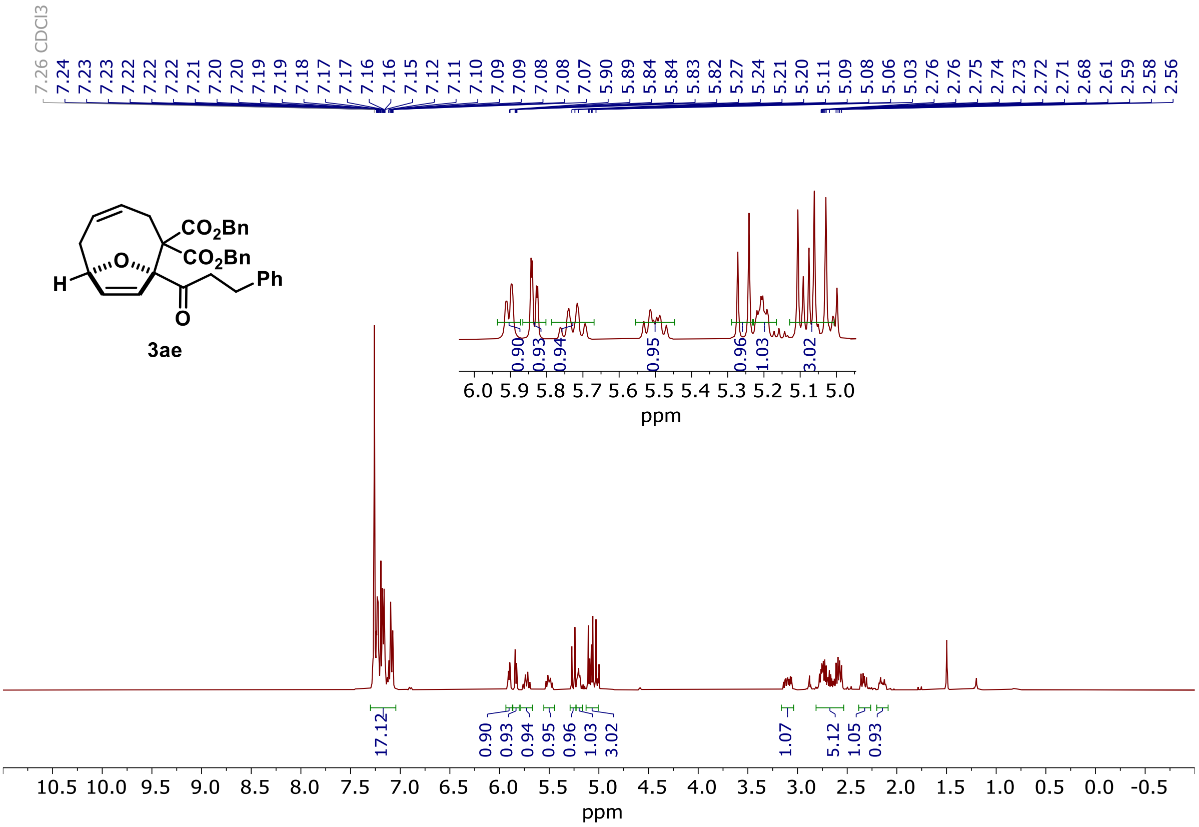

^{13}C NMR (101 MHz, $CDCl_3$) of **3ae**

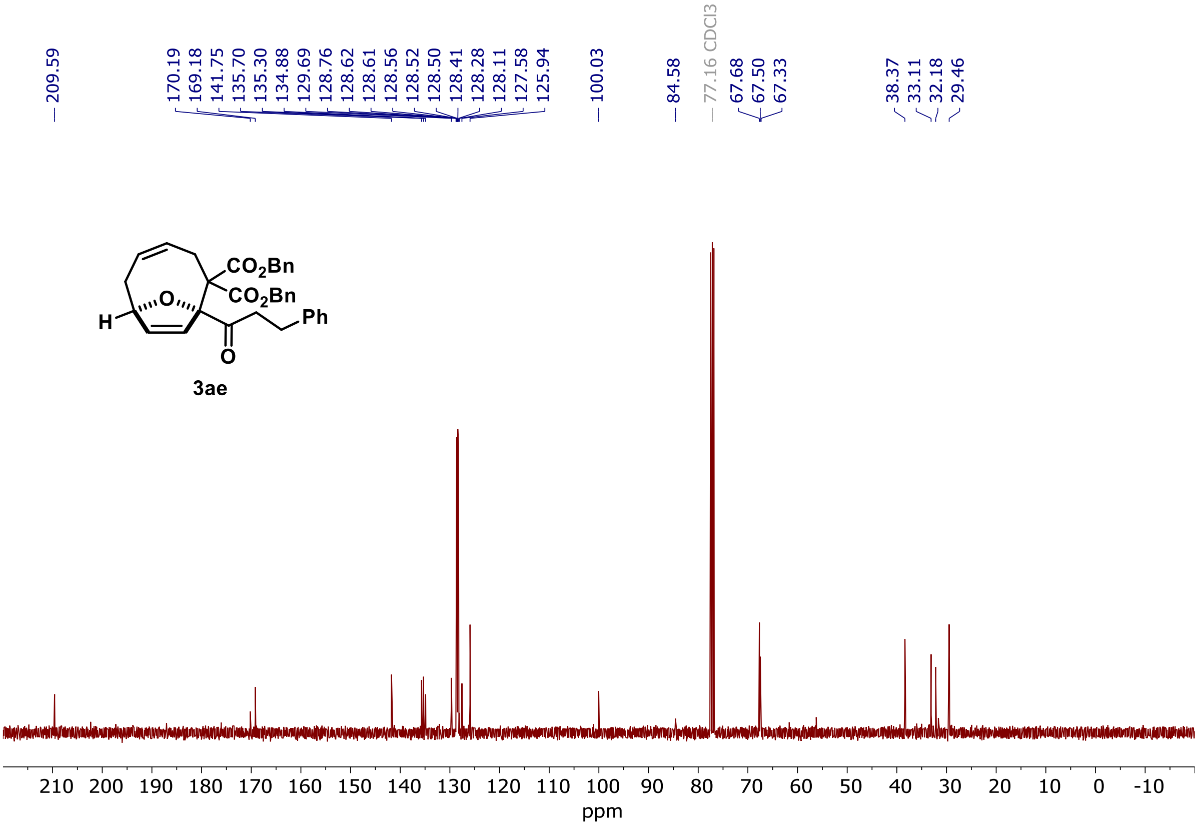

^{1}H NMR (400 MHz, $CDCl_3$) of **3ae’**

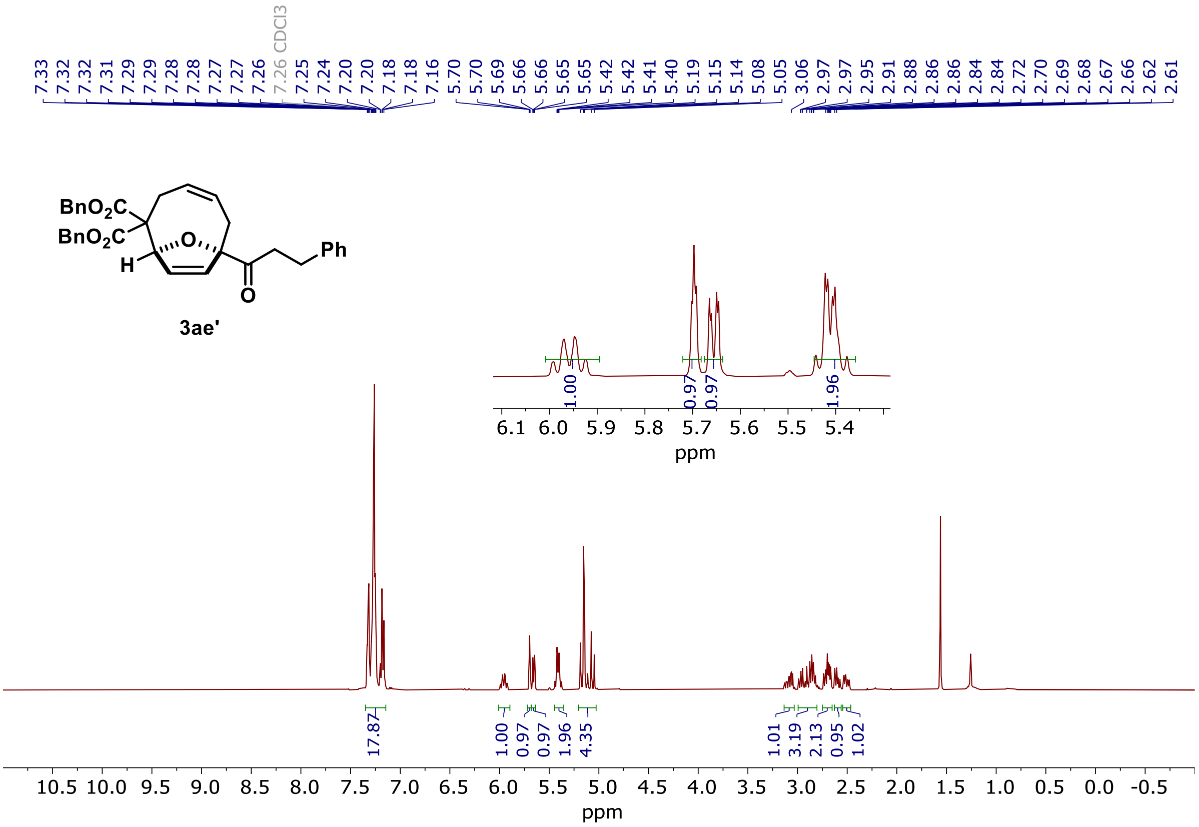

^{13}C NMR (101 MHz, $CDCl_3$) of **3ae’**

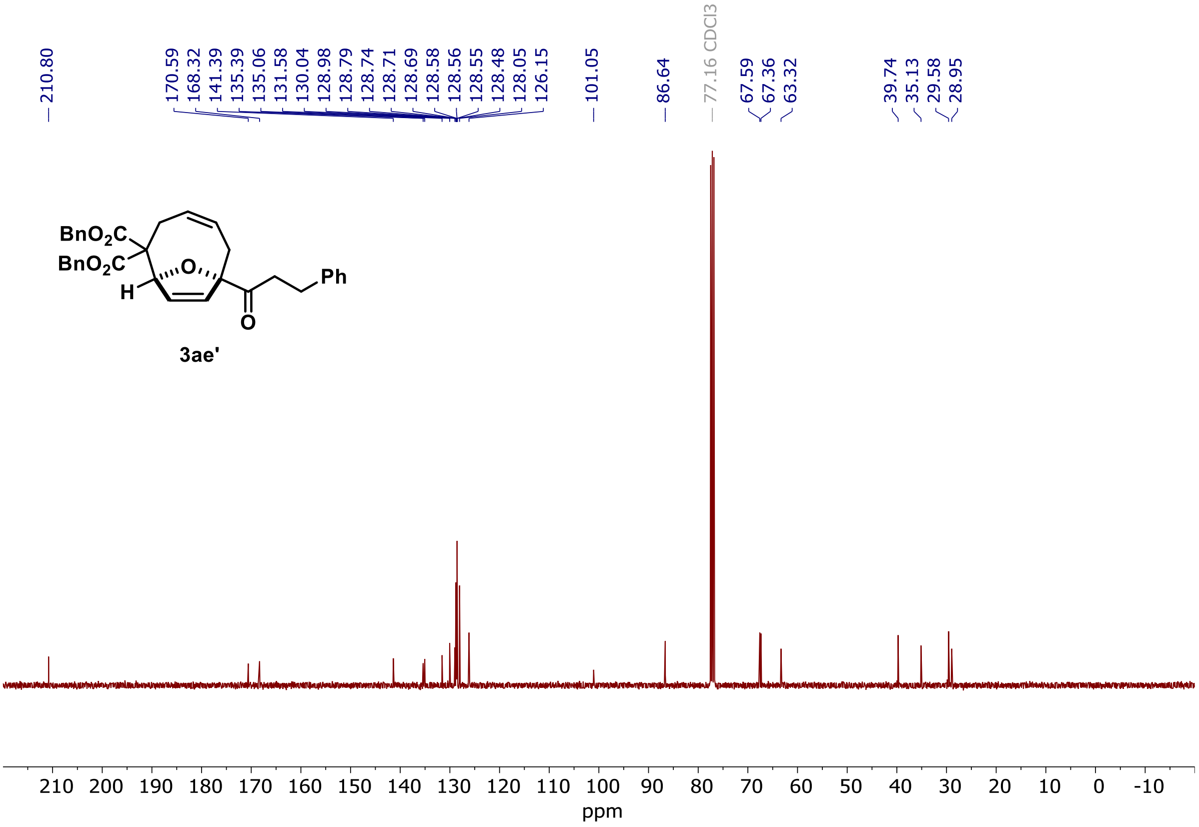

1H NMR (400 MHz, $CDCl_3$) of **3af** & **3af'**

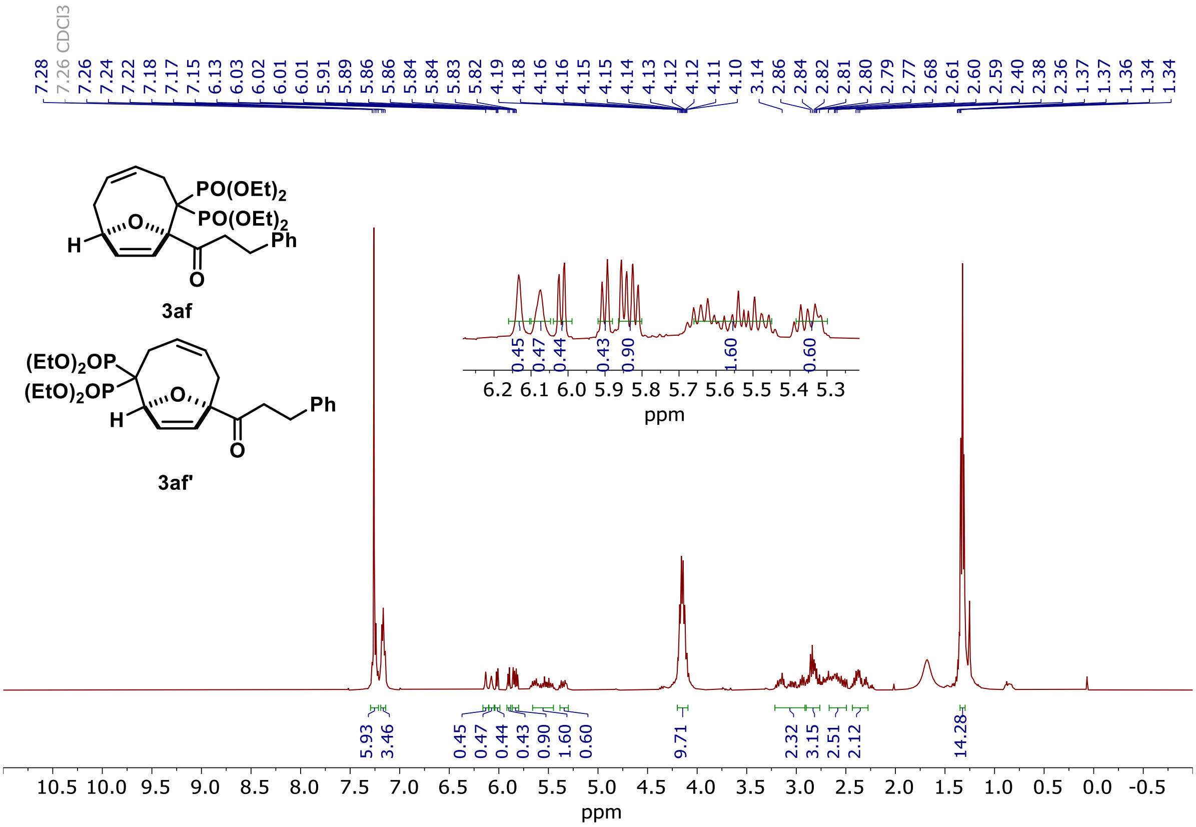

^{13}C NMR (151 MHz, $CDCl_3$) of **3af** & **3af'**

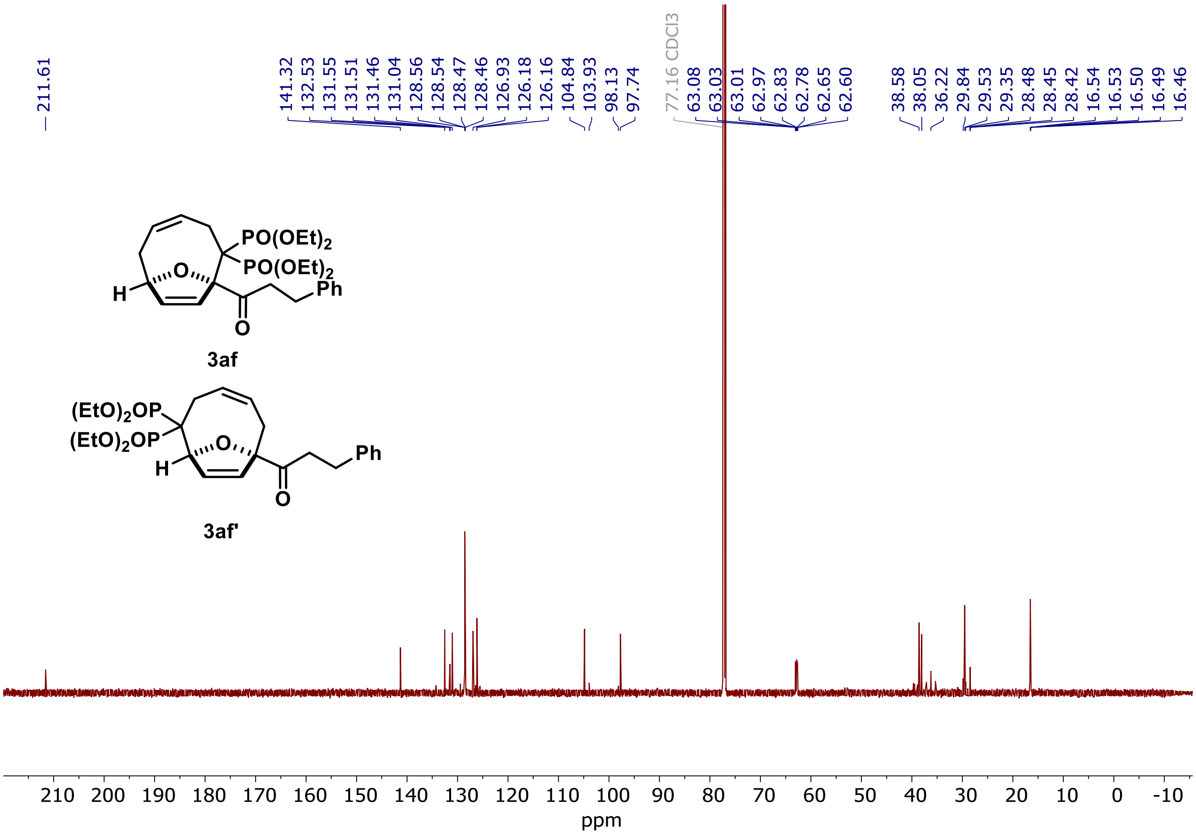

^{1}H NMR (400 MHz, $CDCl_3$) of **3ag**

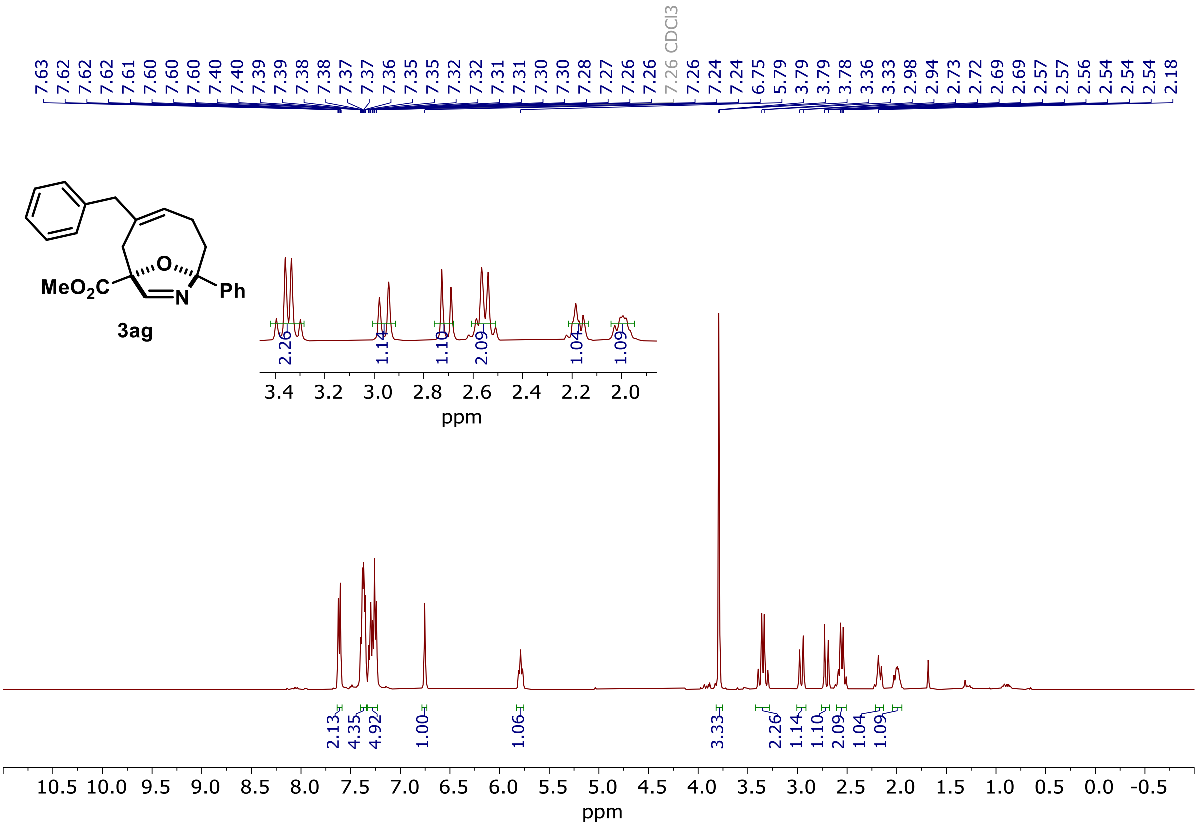

^{13}C NMR (101 MHz, $CDCl_3$) of **3ag**

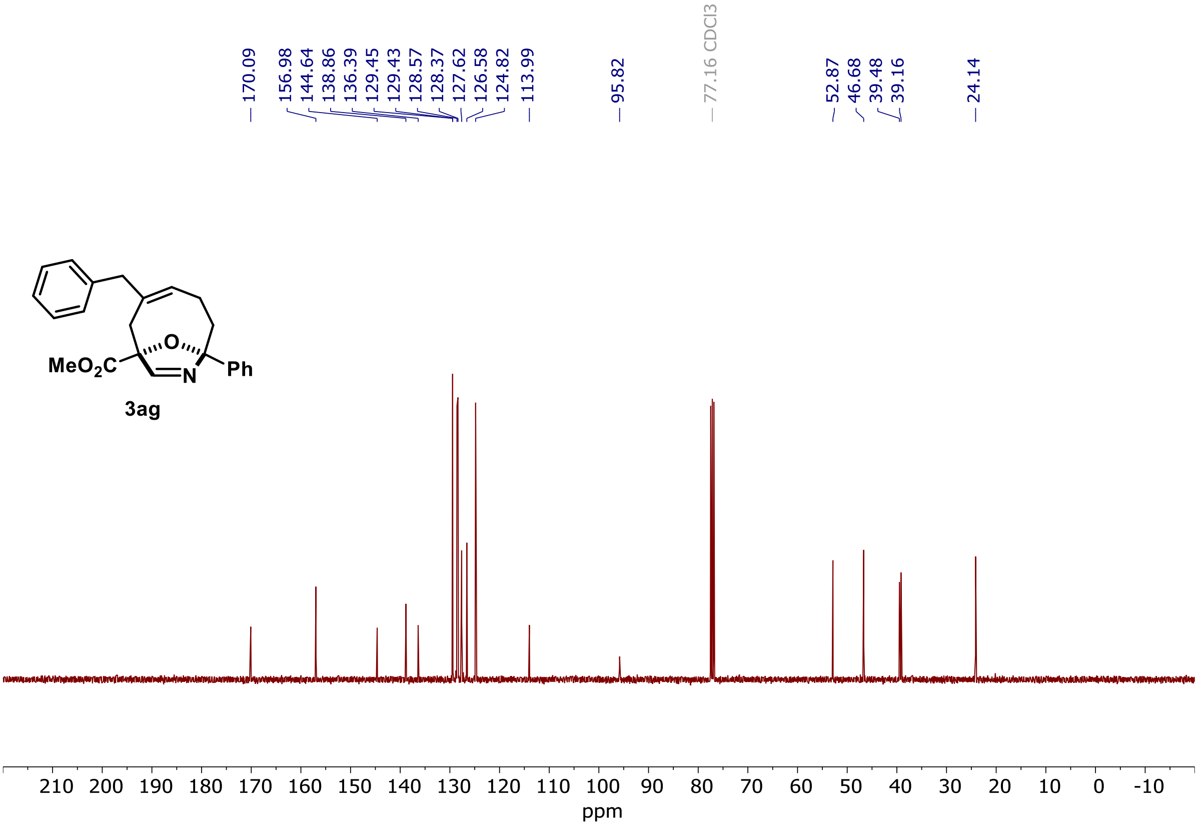

^{1}H NMR (400 MHz, $CDCl_3$) of **3ag'**

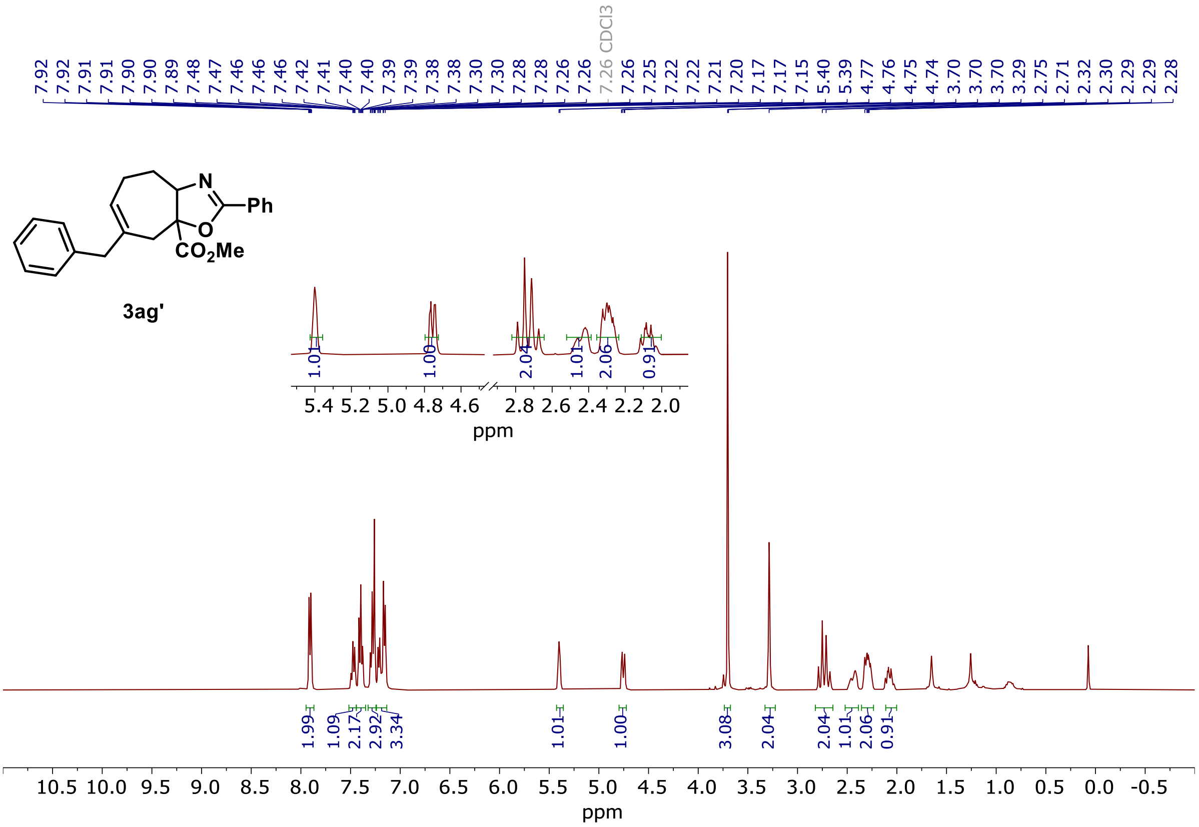

^{13}C NMR (101 MHz, $CDCl_3$) of **3ag'**

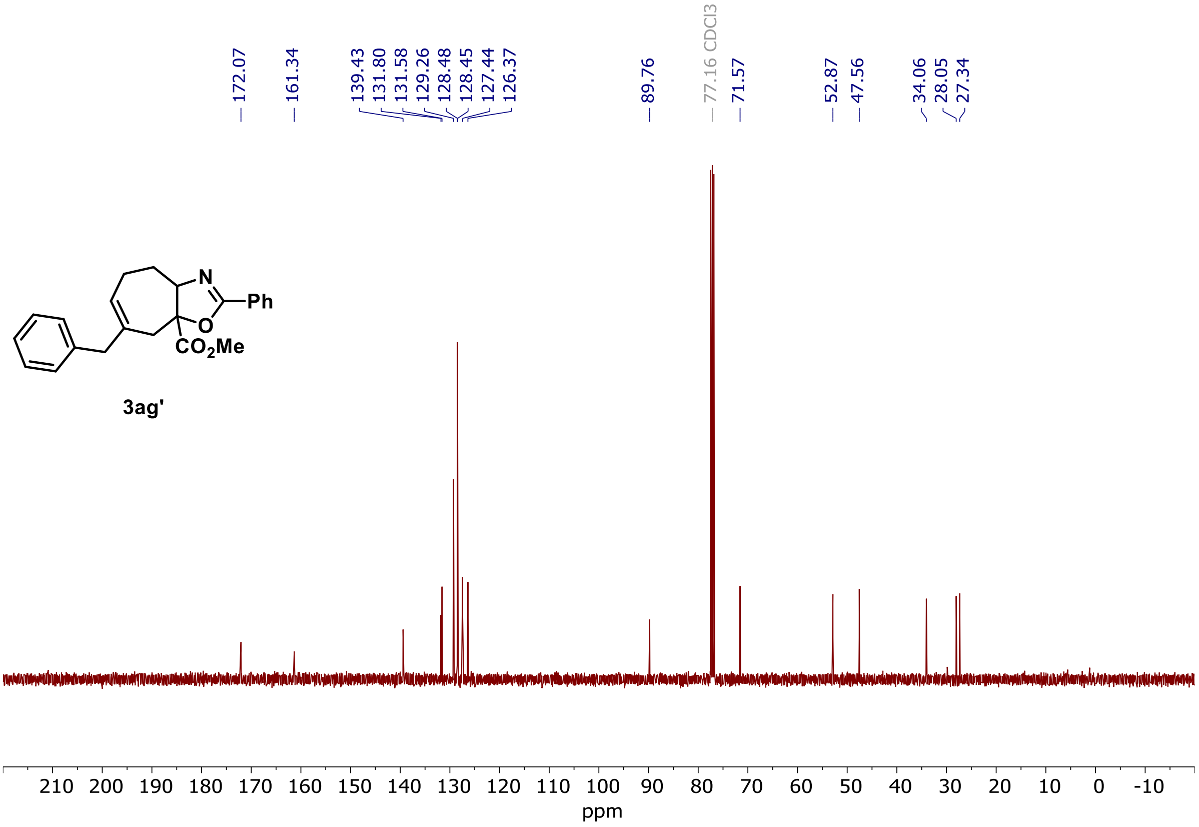

^{1}H NMR (599 MHz, $CDCl_3$) of **3ah**

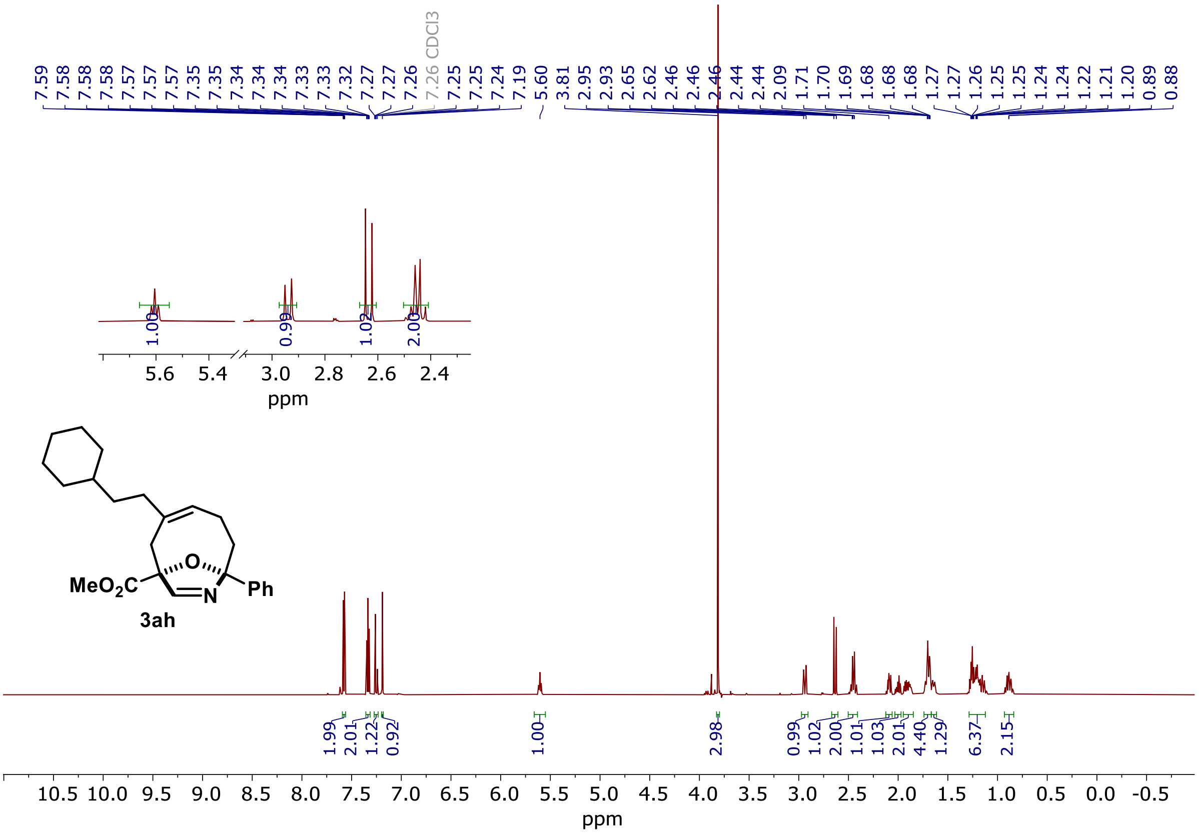

^{13}C NMR (151 MHz, $CDCl_3$) of **3ah**

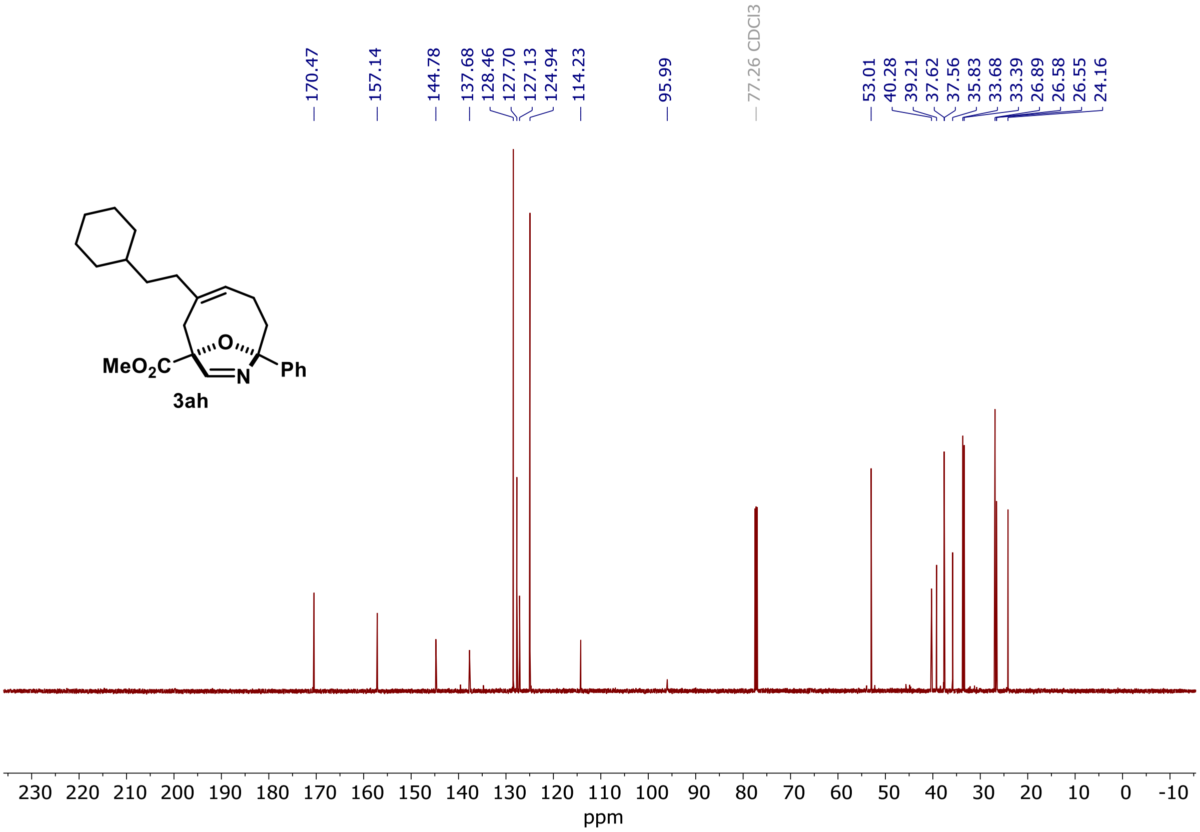

^{1}H NMR (599 MHz, $CDCl_3$) of **3ah’**

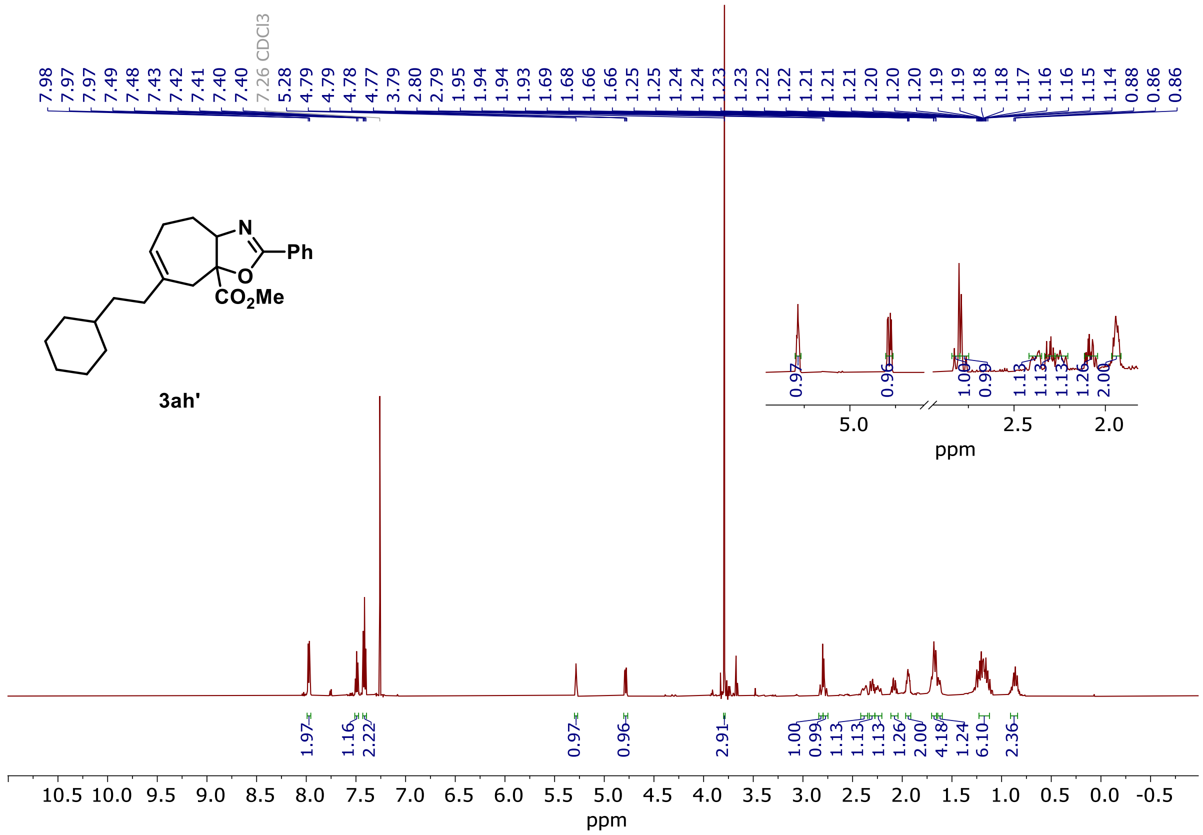

^{13}C NMR (151 MHz, $CDCl_3$) of **3ah’**

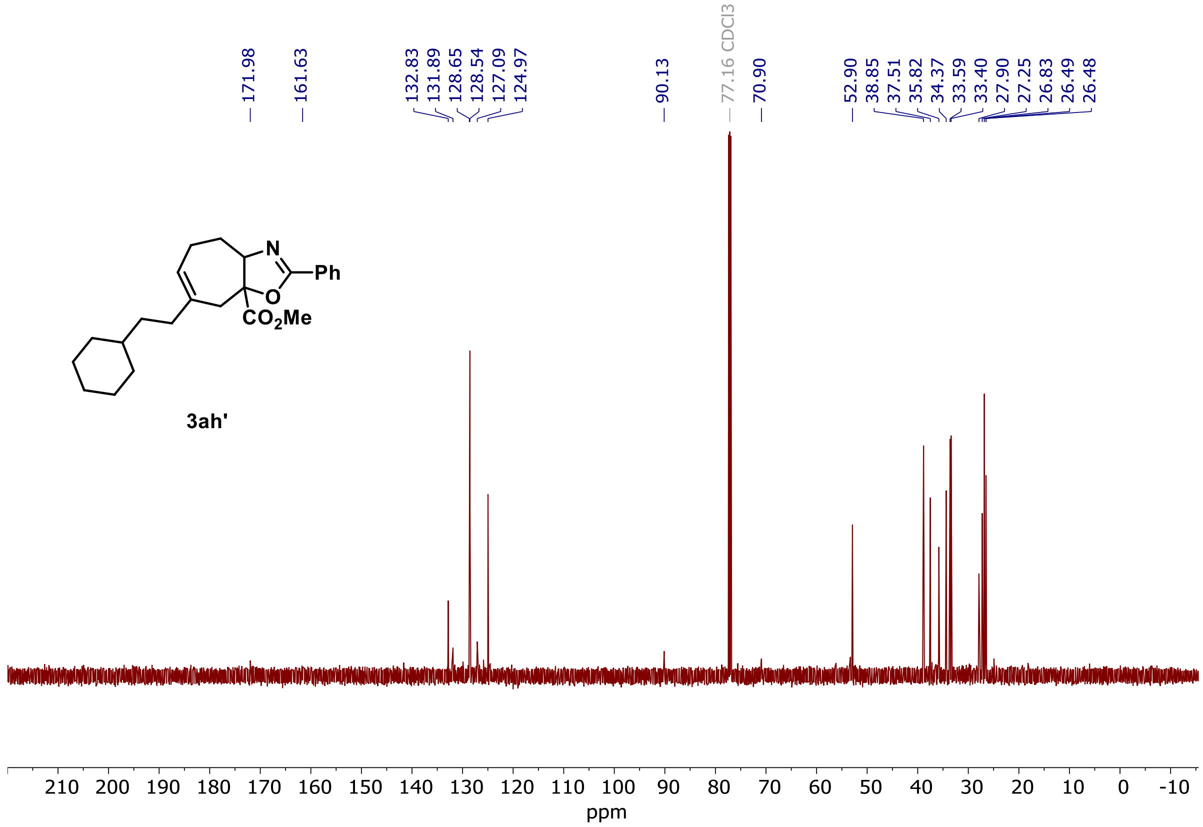

^{1}H NMR (599 MHz, $CDCl_3$) of **3ai**

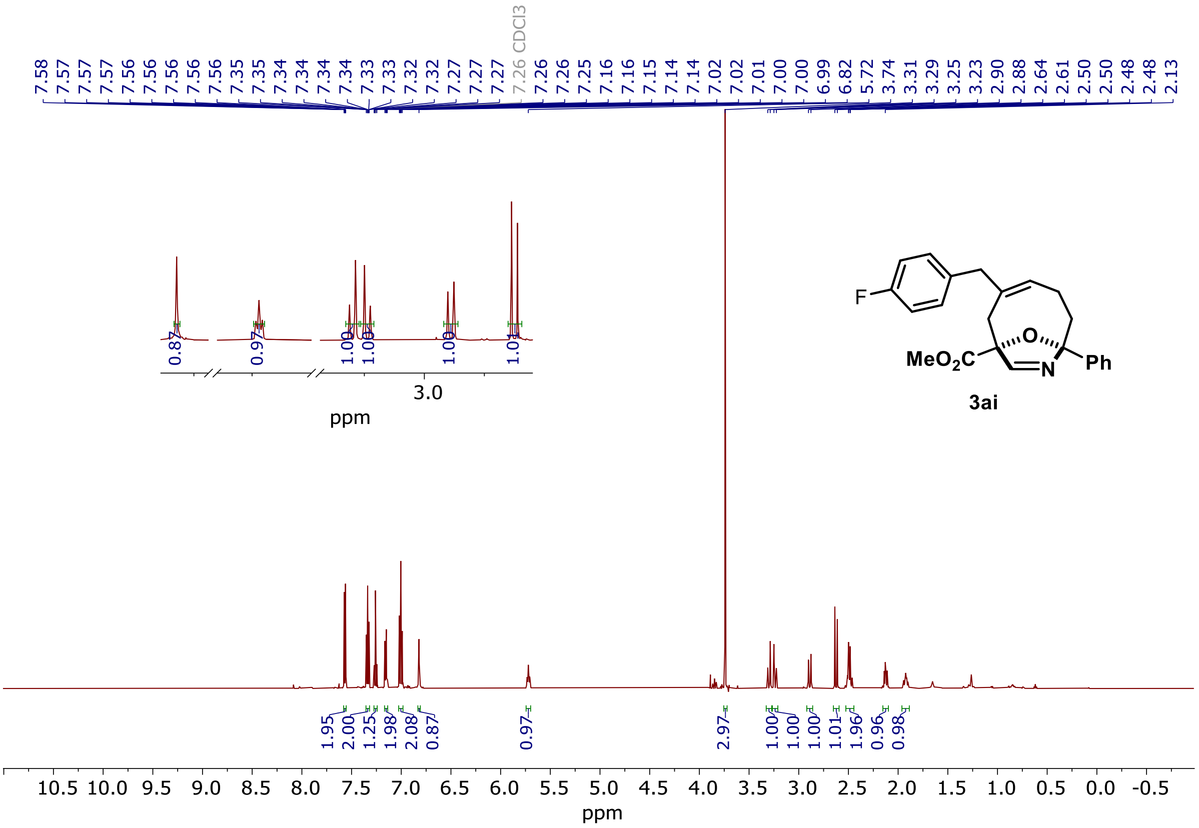

^{13}C{^{19}F} NMR (126 MHz, $CDCl_3$) of **3ai**

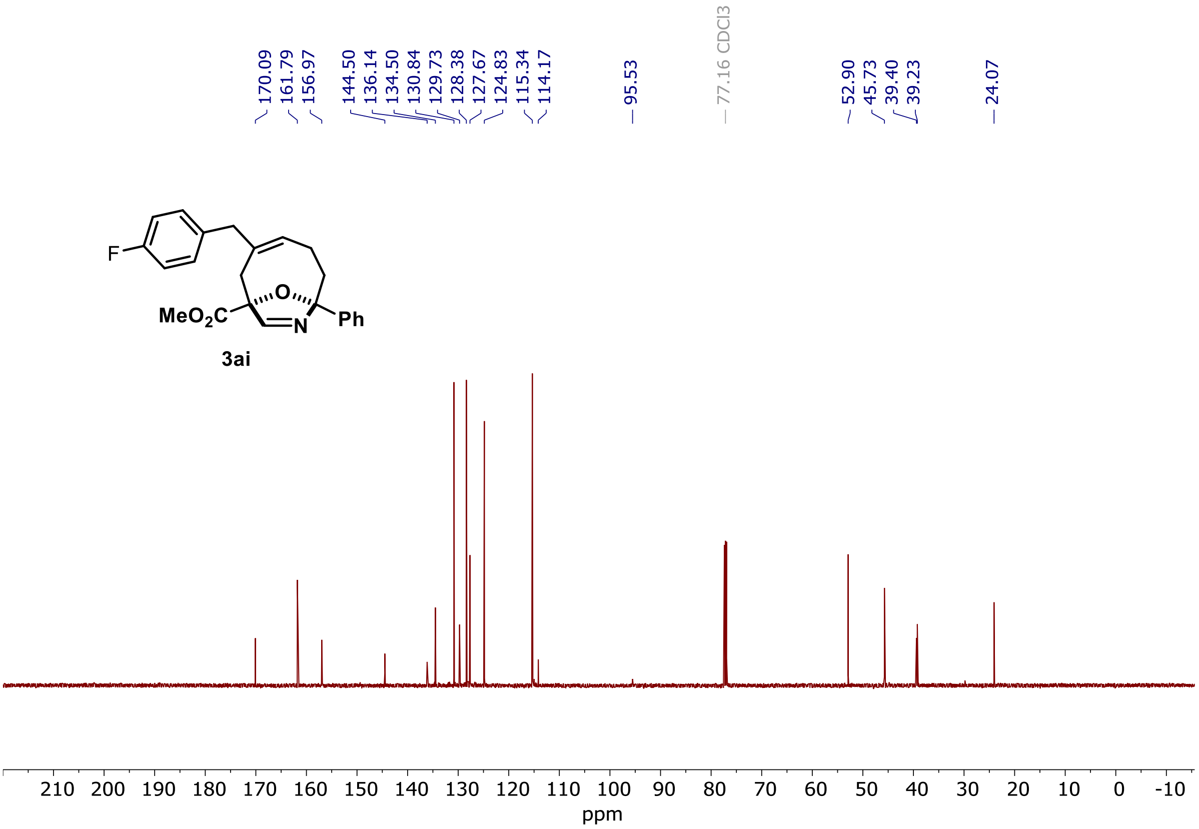

^{13}C NMR (126 MHz, $CDCl_3$) of **3ai**

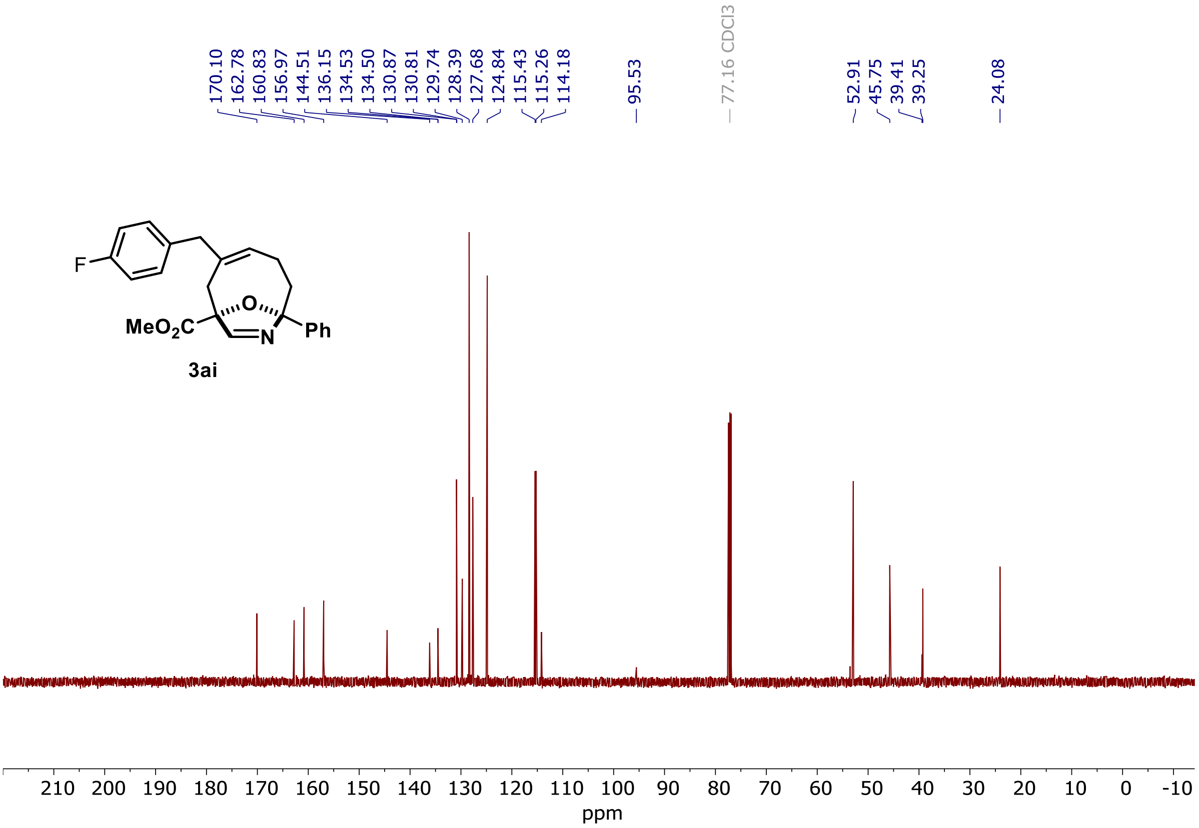

^{19}F NMR (563 MHz, $CDCl_3$) of **3ai**

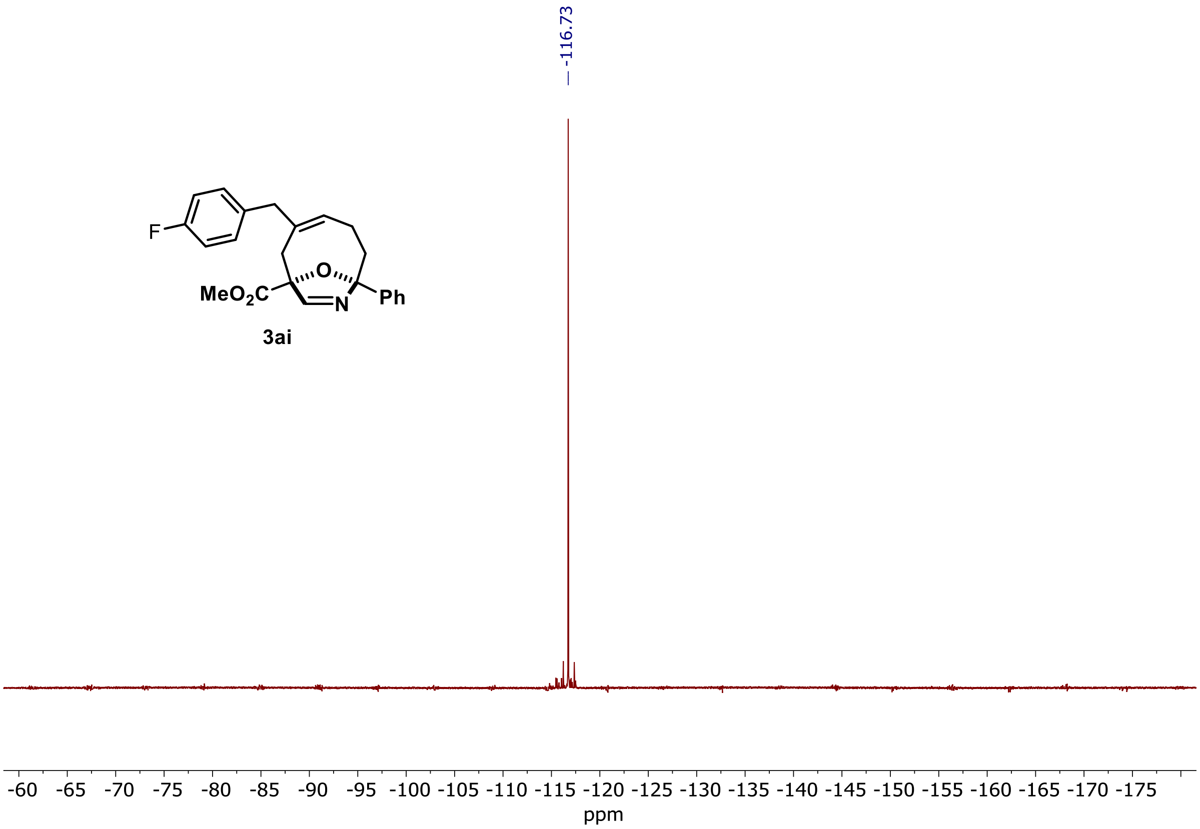

^{1}H NMR (500 MHz, $CDCl_3$) of **3ai'**

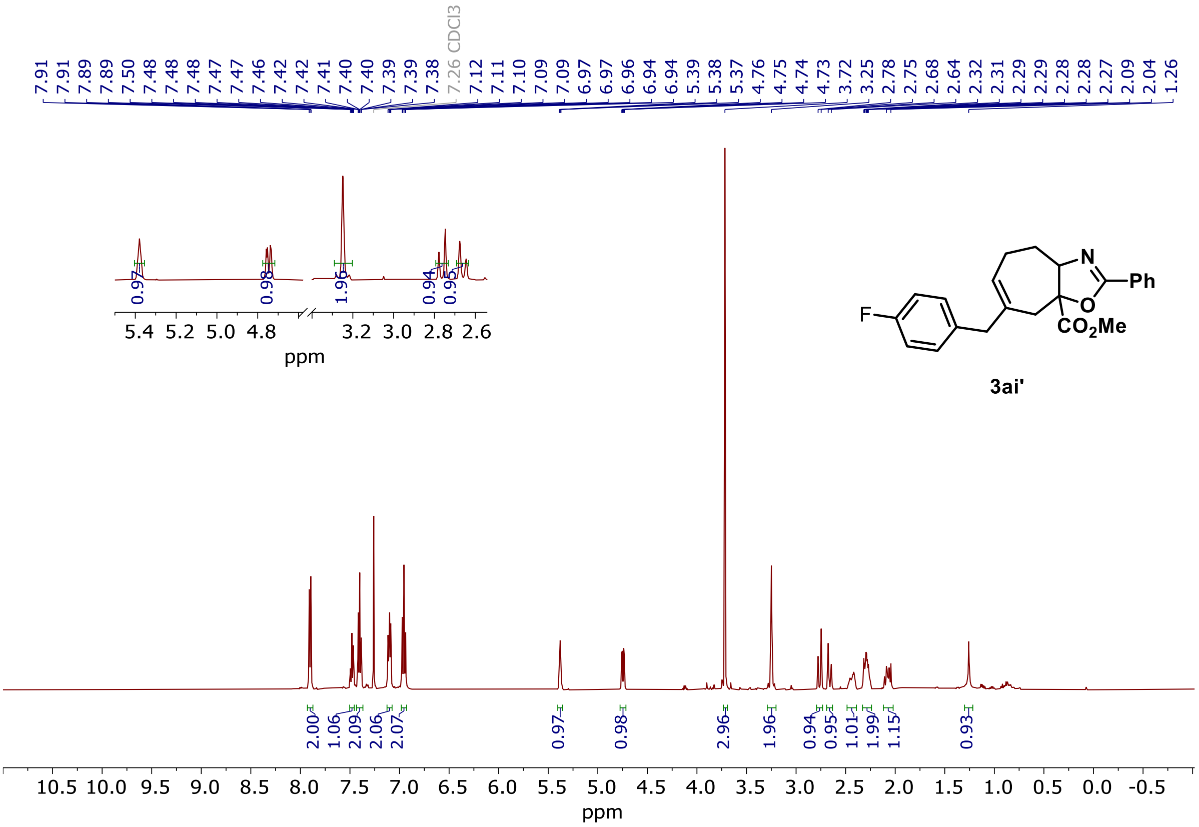

^{13}C{^{19}F} NMR (126 MHz, $CDCl_3$) of **3ai'**

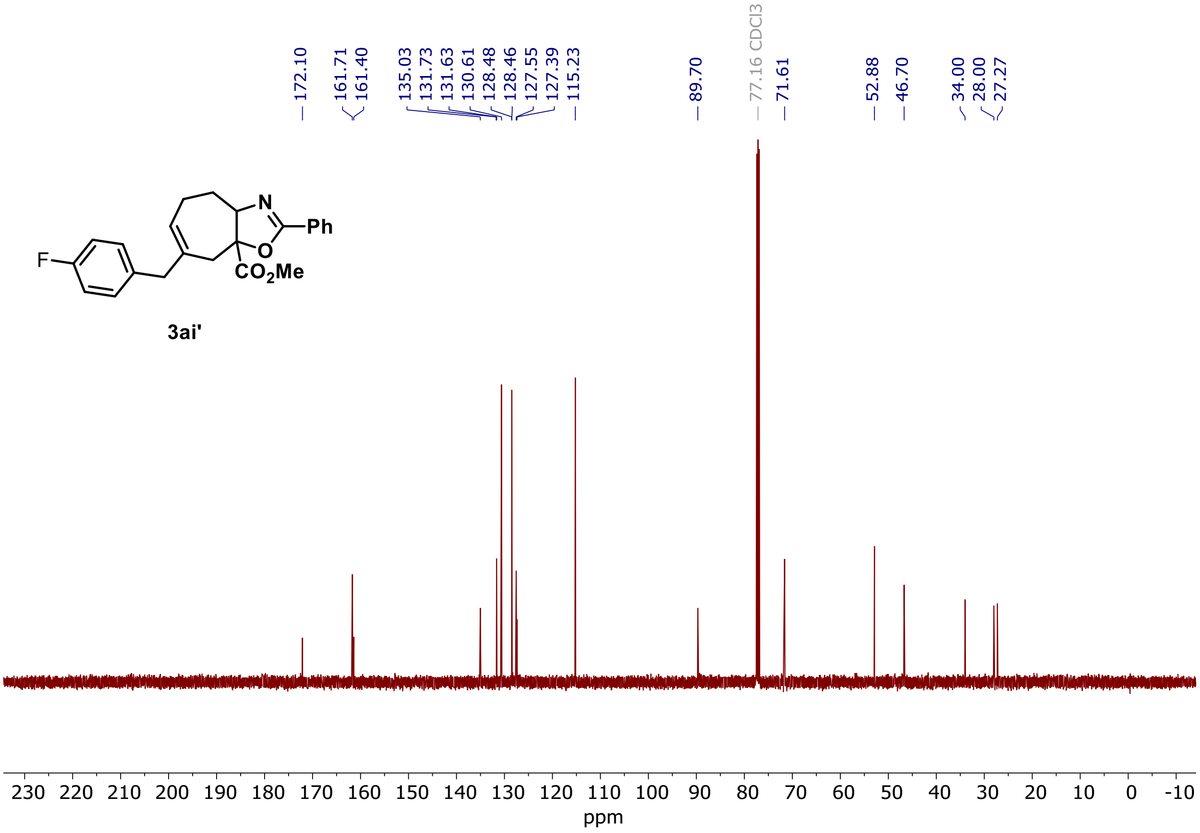

^{13}C NMR (126 MHz, $CDCl_3$) of **3ai’**

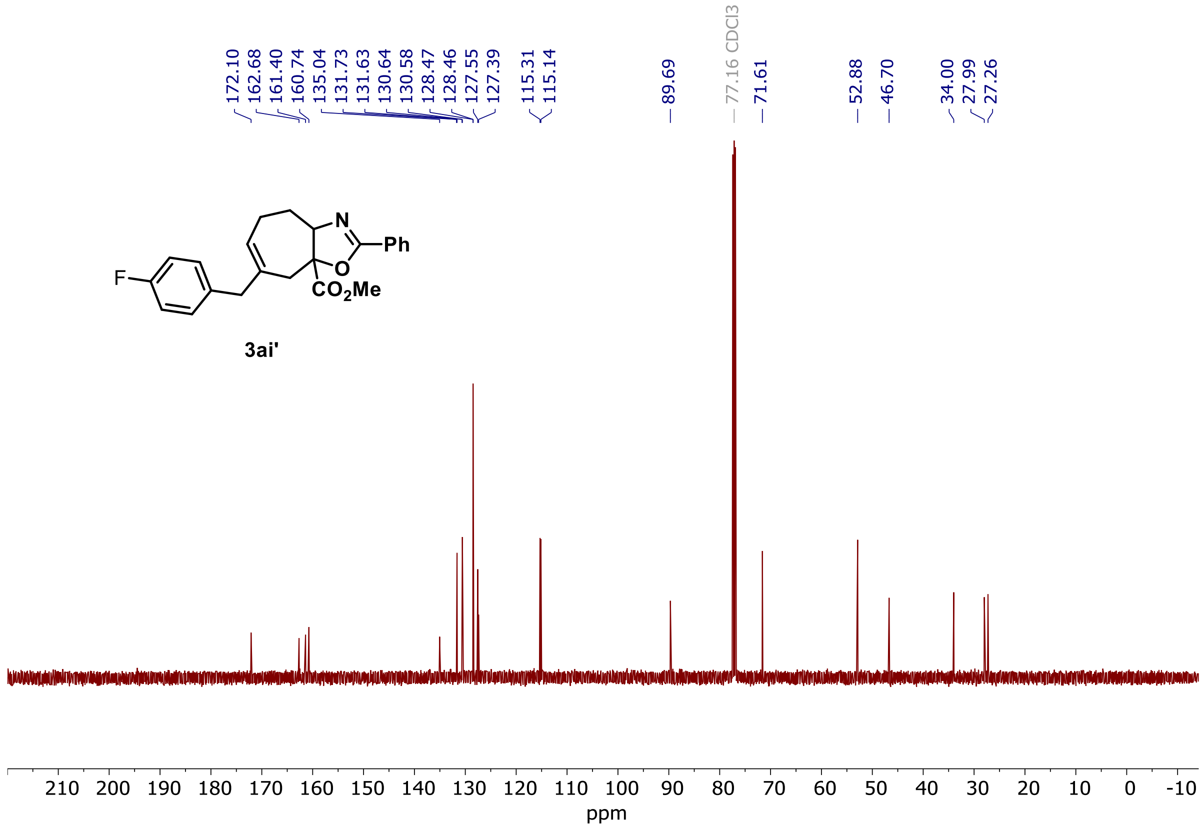

^{19}F NMR (470 MHz, $CDCl_3$) of **3ai’**

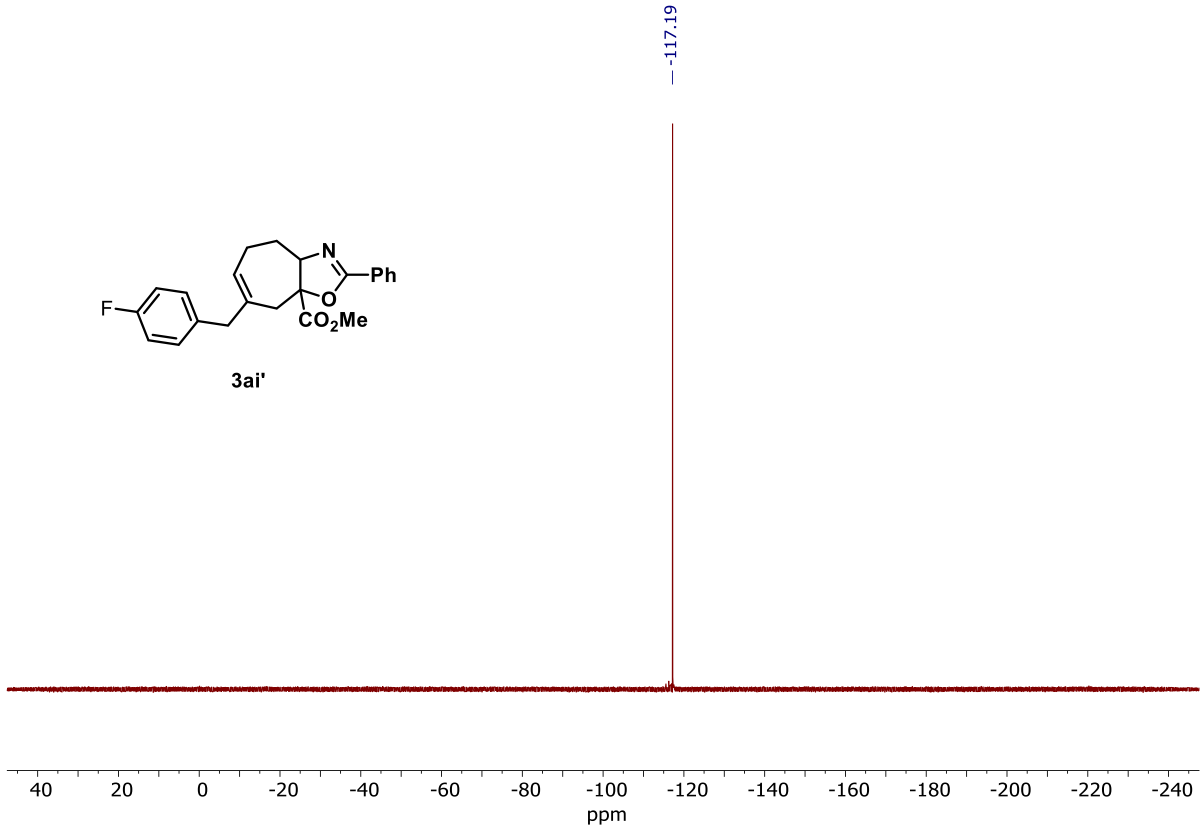

^{1}H NMR (400 MHz, $CDCl_3$) **3aj**

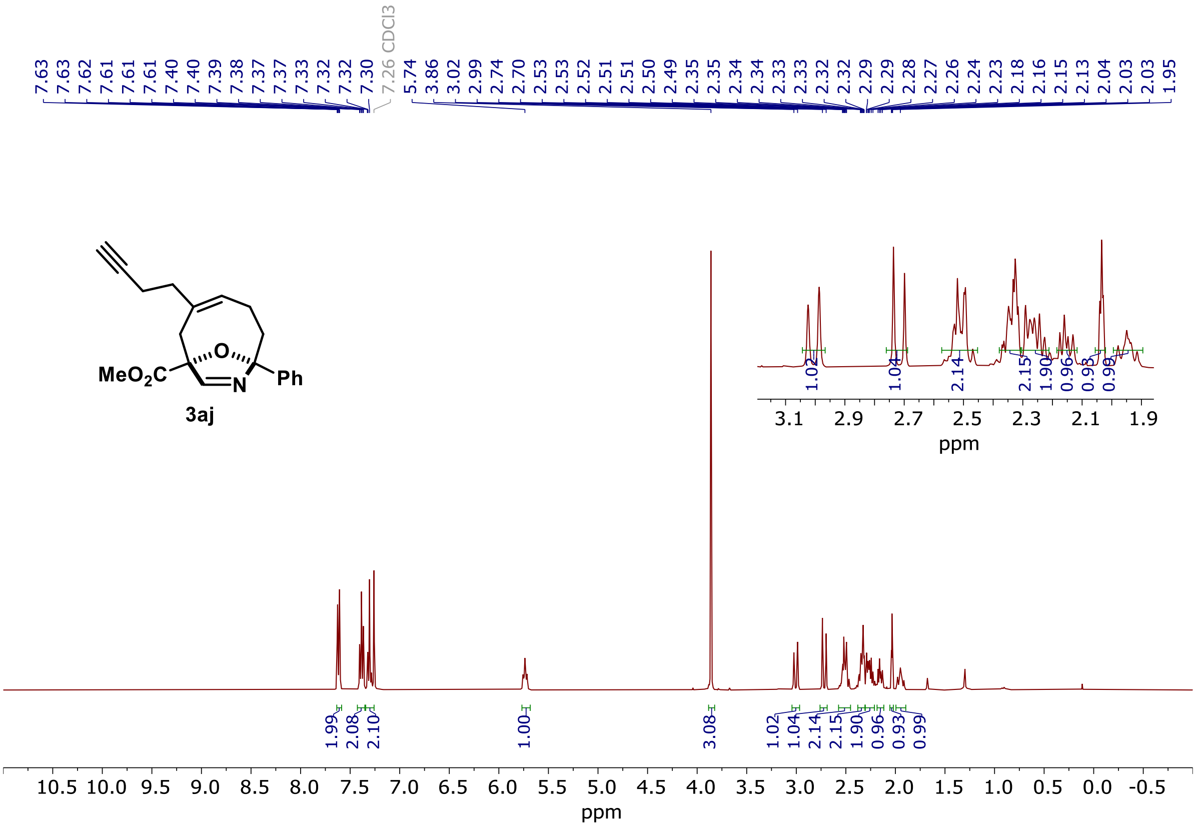

^{13}C NMR (101 MHz, $CDCl_3$) of **3aj**

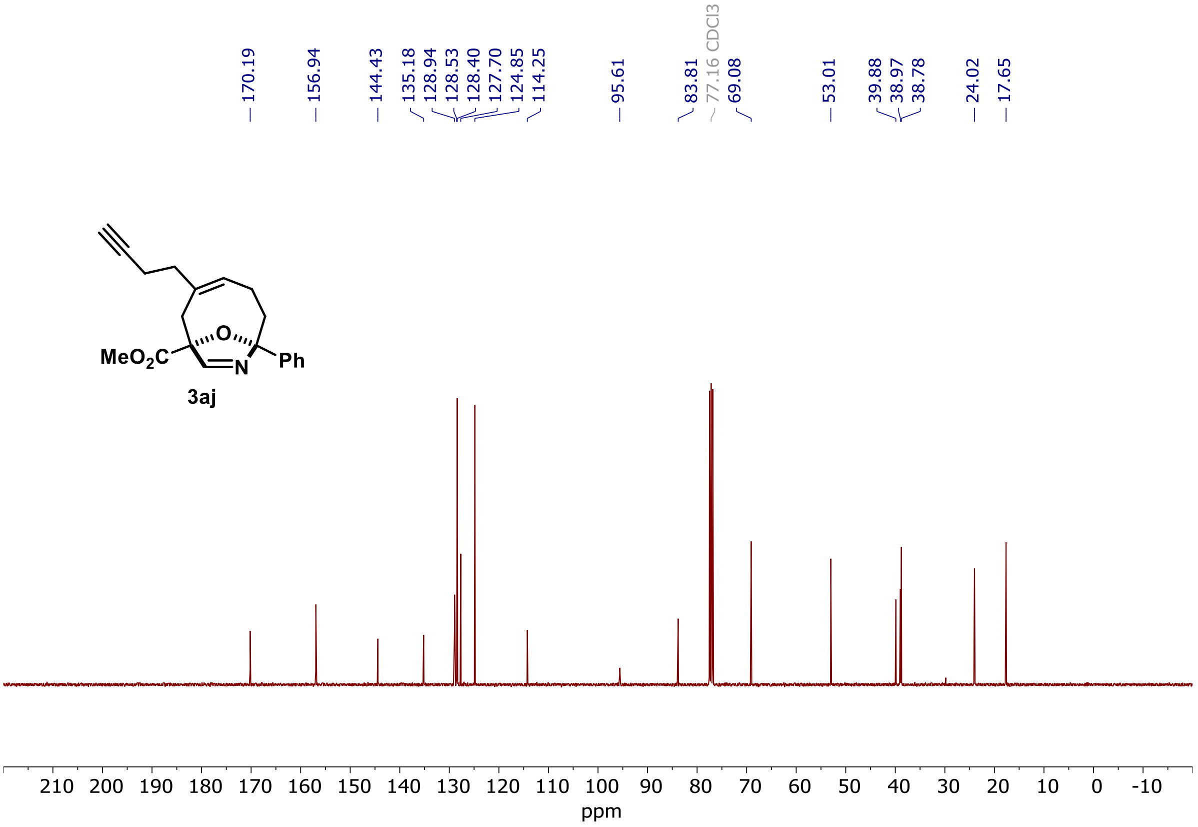

^{1}H NMR (400 MHz, $CDCl_3$) of **3aj'**

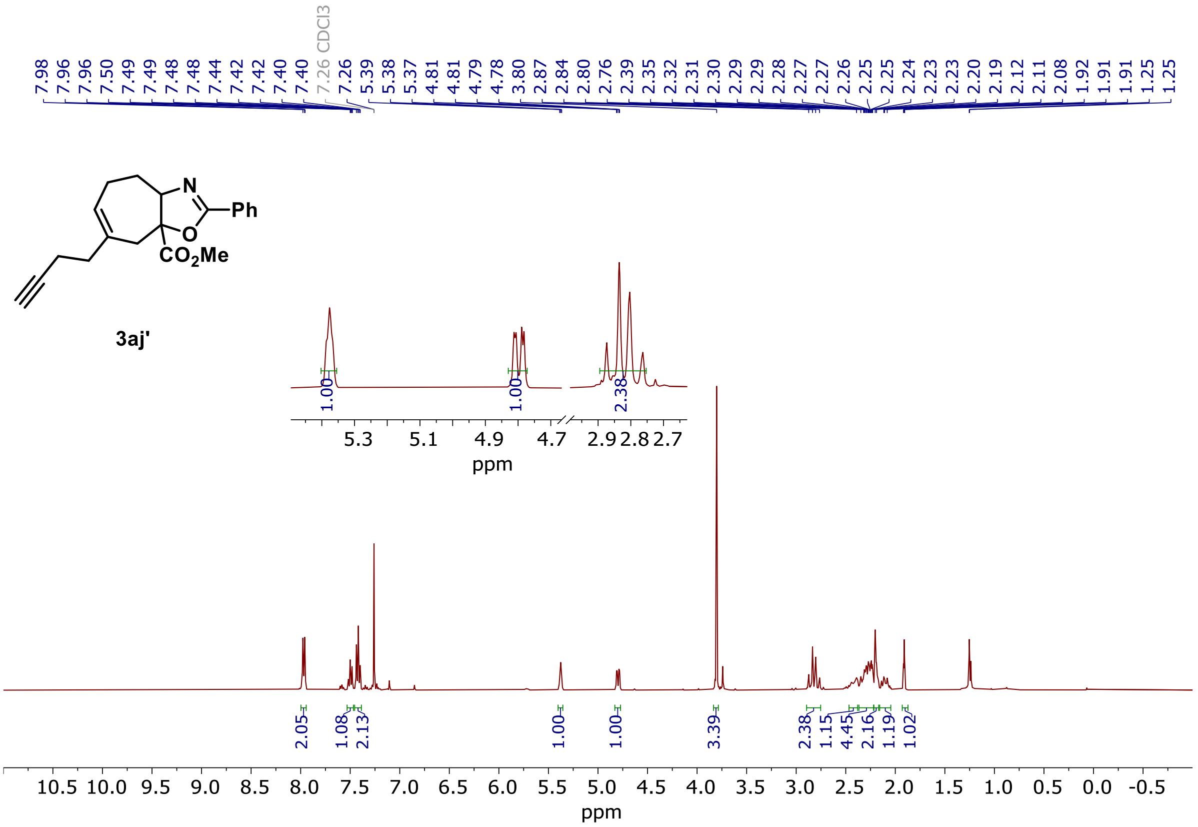

^{13}C NMR (101 MHz, $CDCl_3$) of **3aj'**

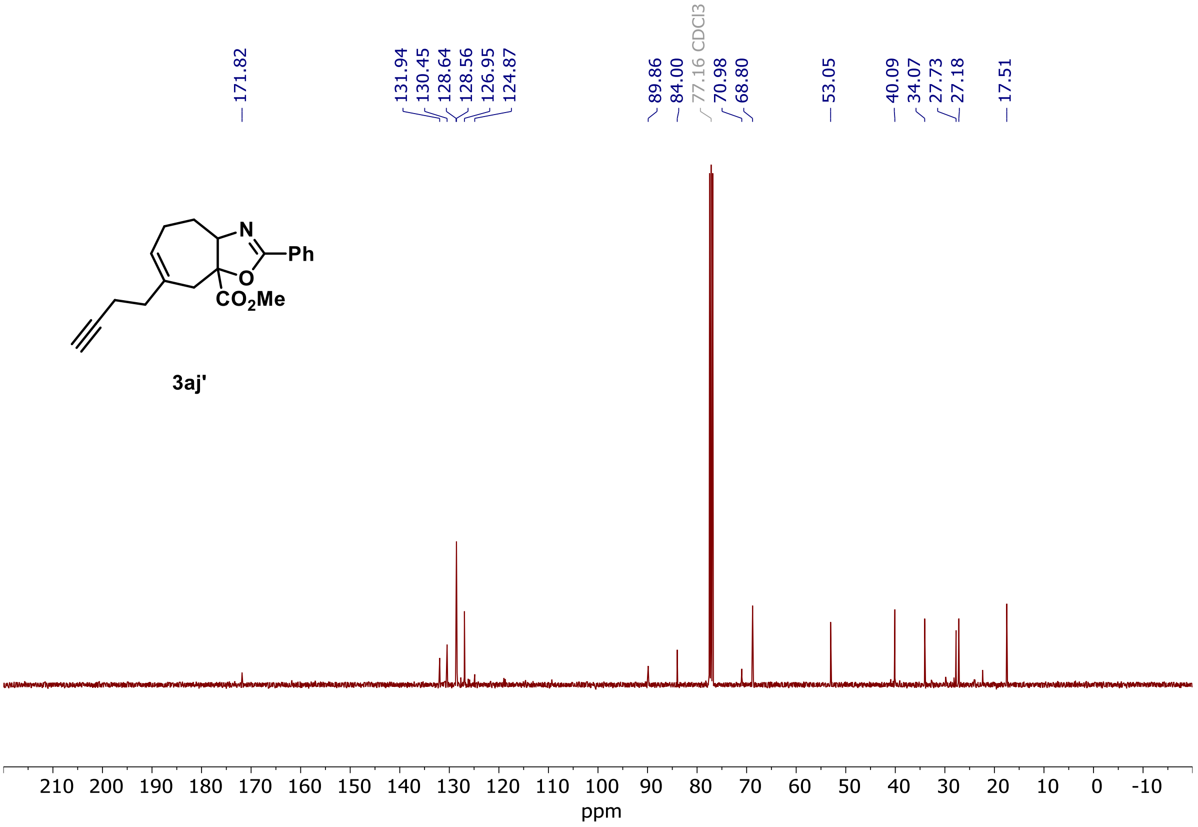

^{1}H NMR (400 MHz, $CDCl_3$) of **3ak**

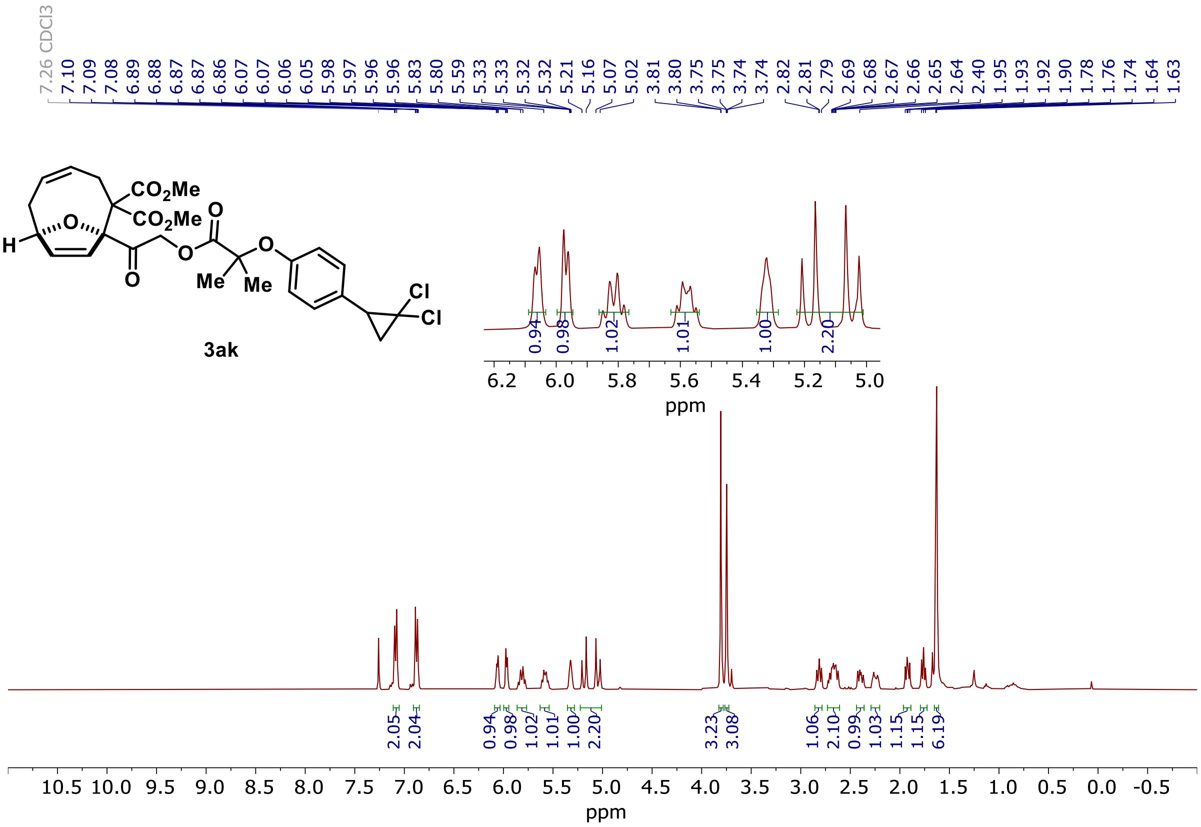

^{13}C NMR (101 MHz, $CDCl_3$) of **3ak**

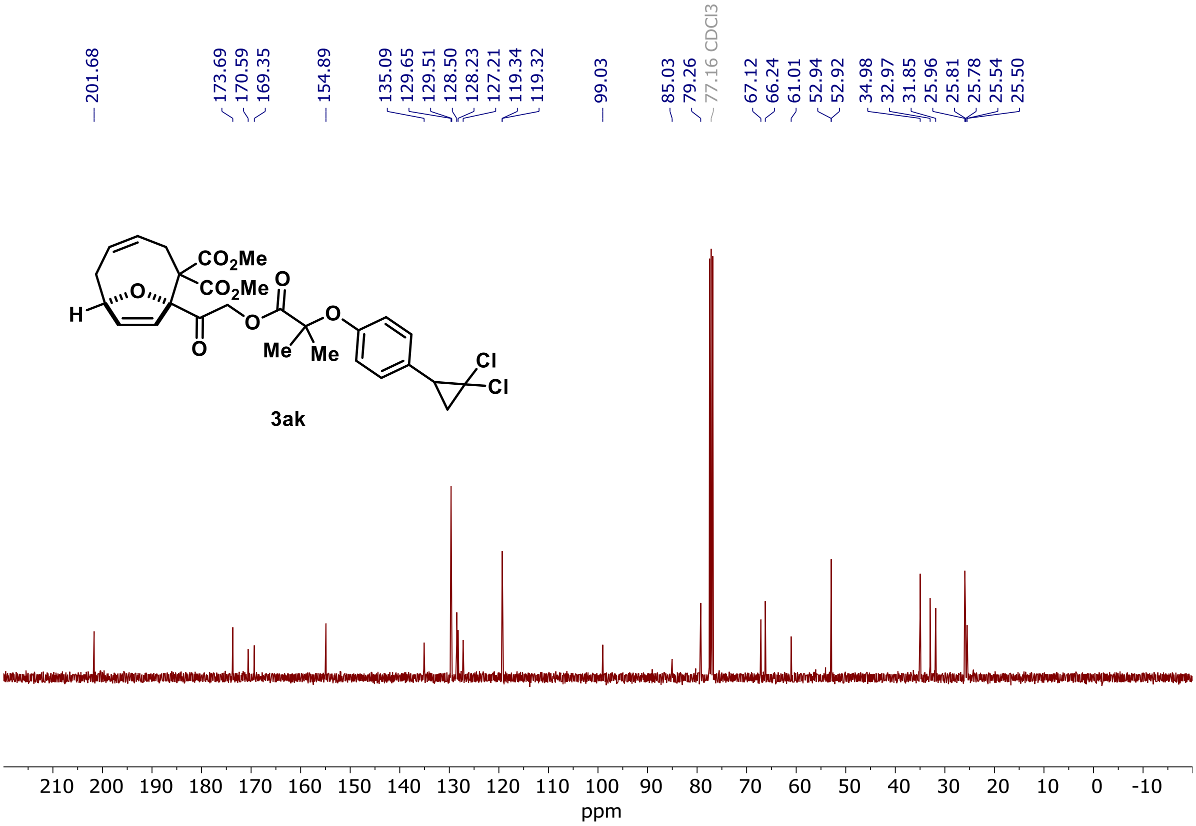

^{1}H NMR (400 MHz, CDCl$_3$) of **3ak’**

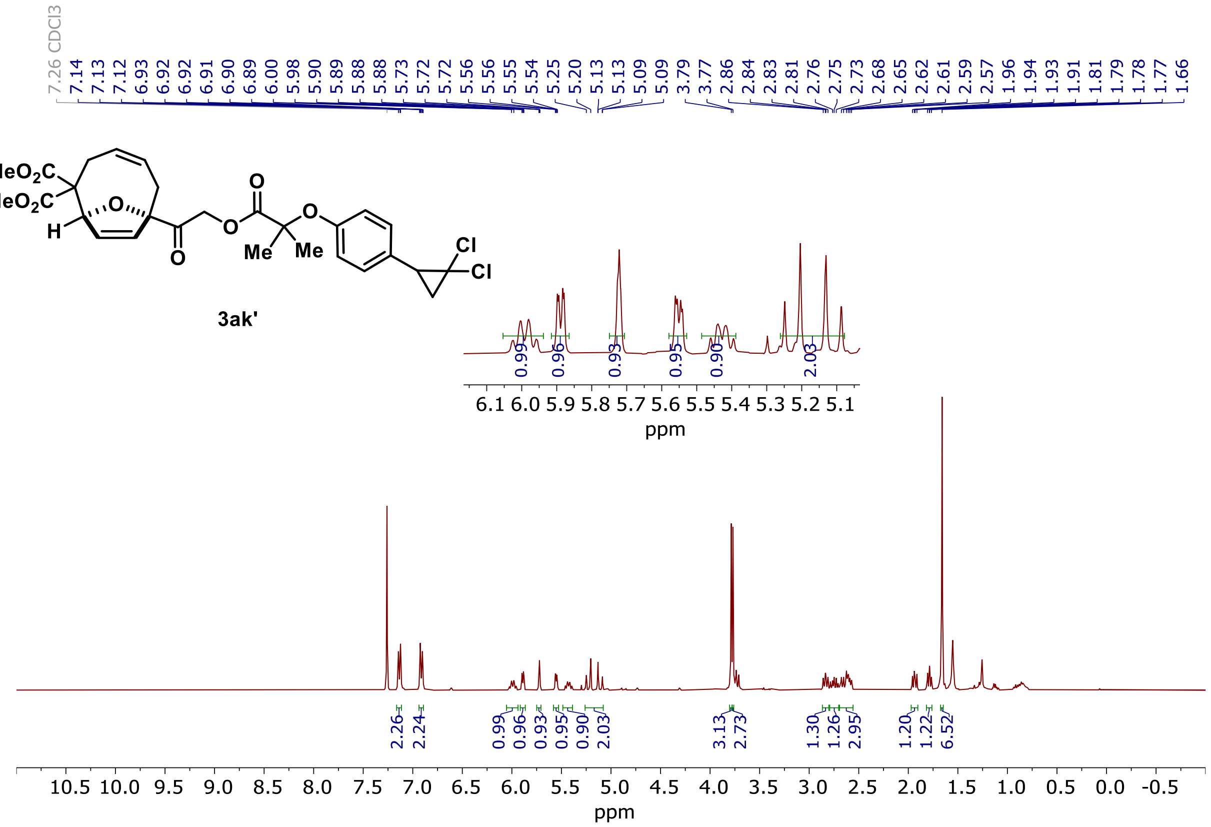

^{13}C NMR (151 MHz, CDCl$_3$) of **3ak’**

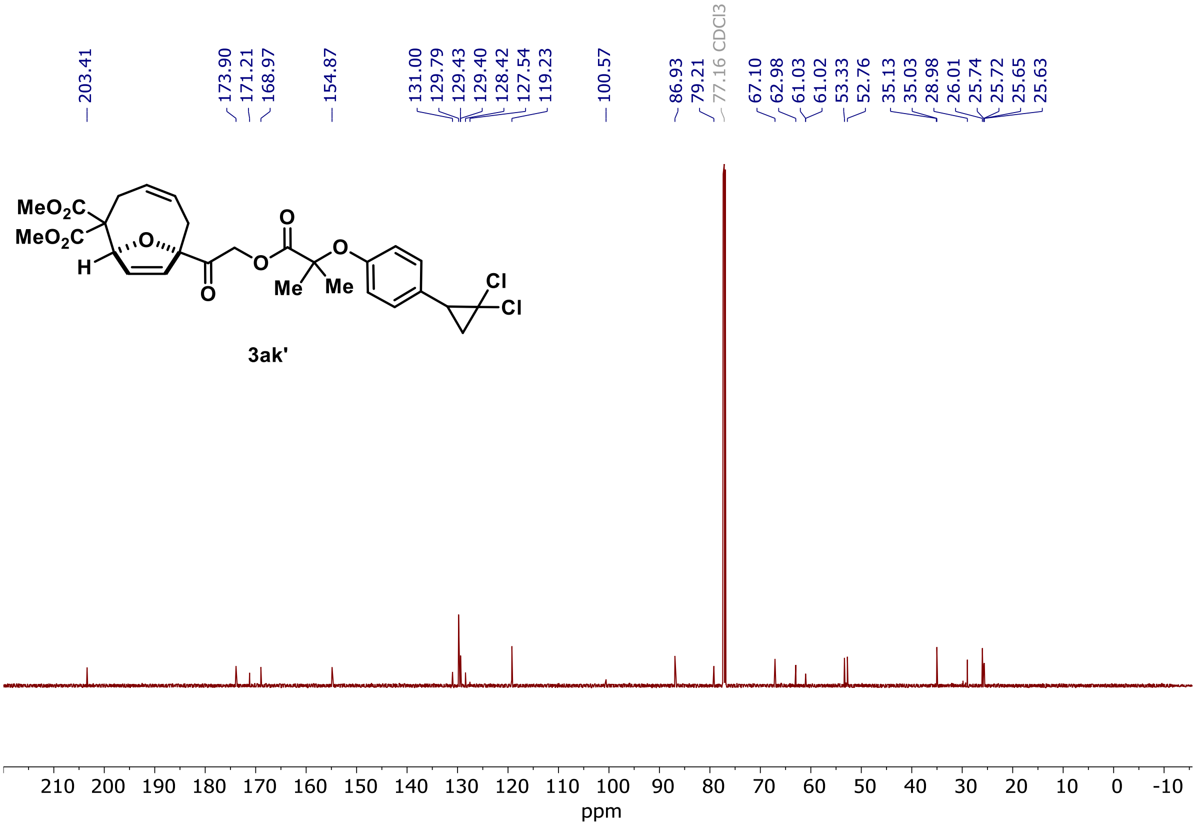

^{1}H NMR (400 MHz, $CDCl_3$) of **3al**

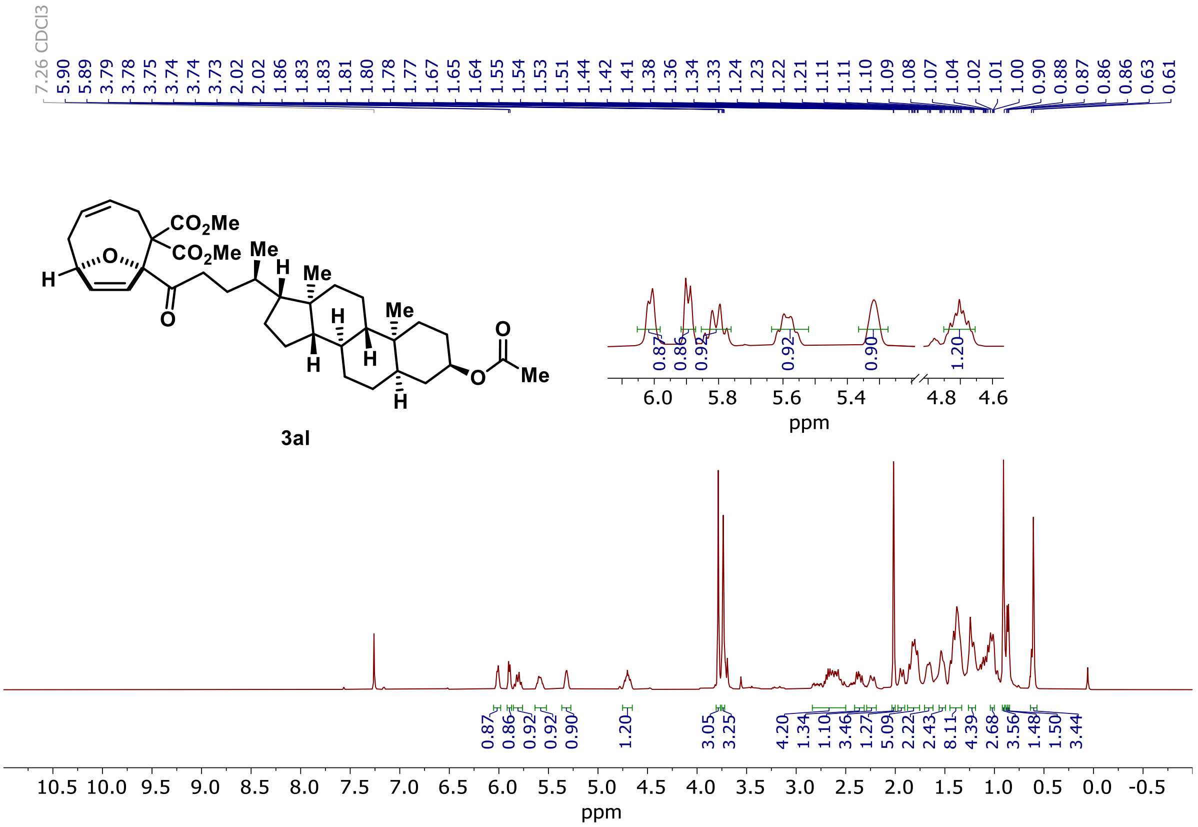

^{13}C NMR (101 MHz, $CDCl_3$) of **3al**

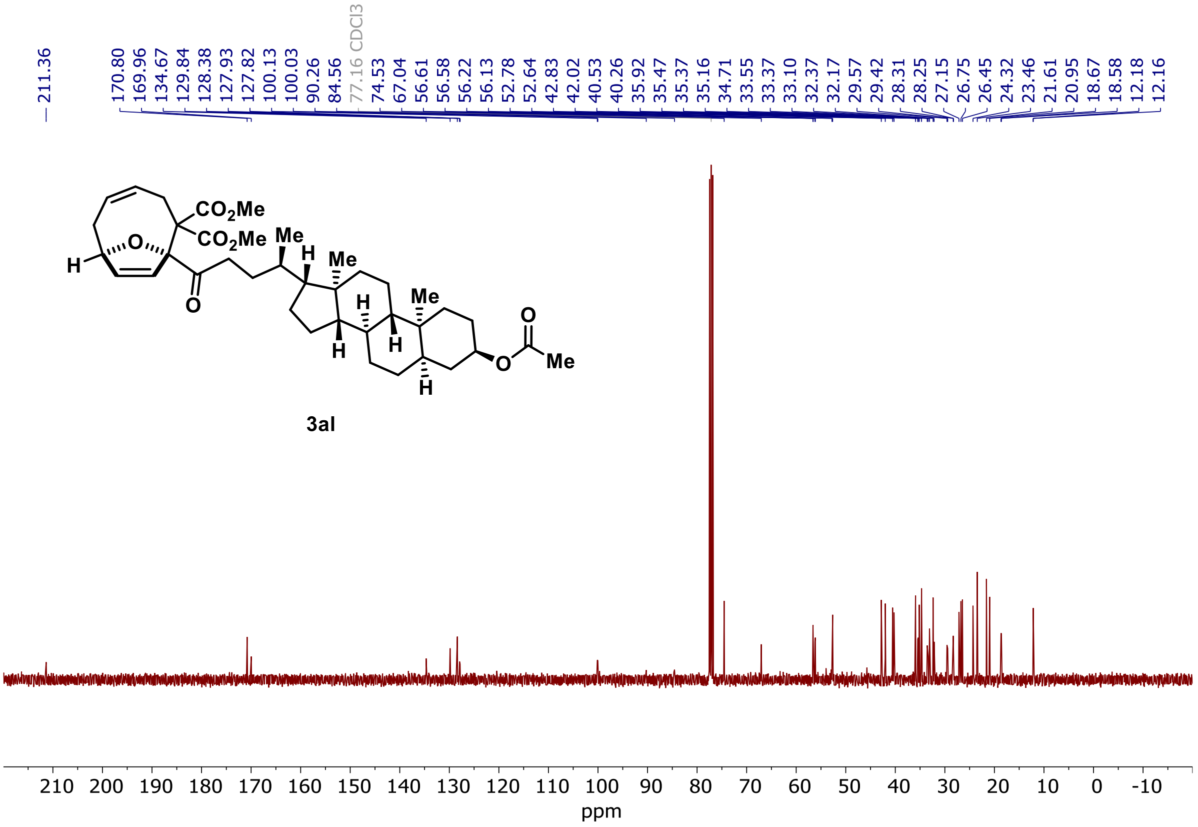

^{1}H NMR (400 MHz, CDCl$_3$) of **3al’**

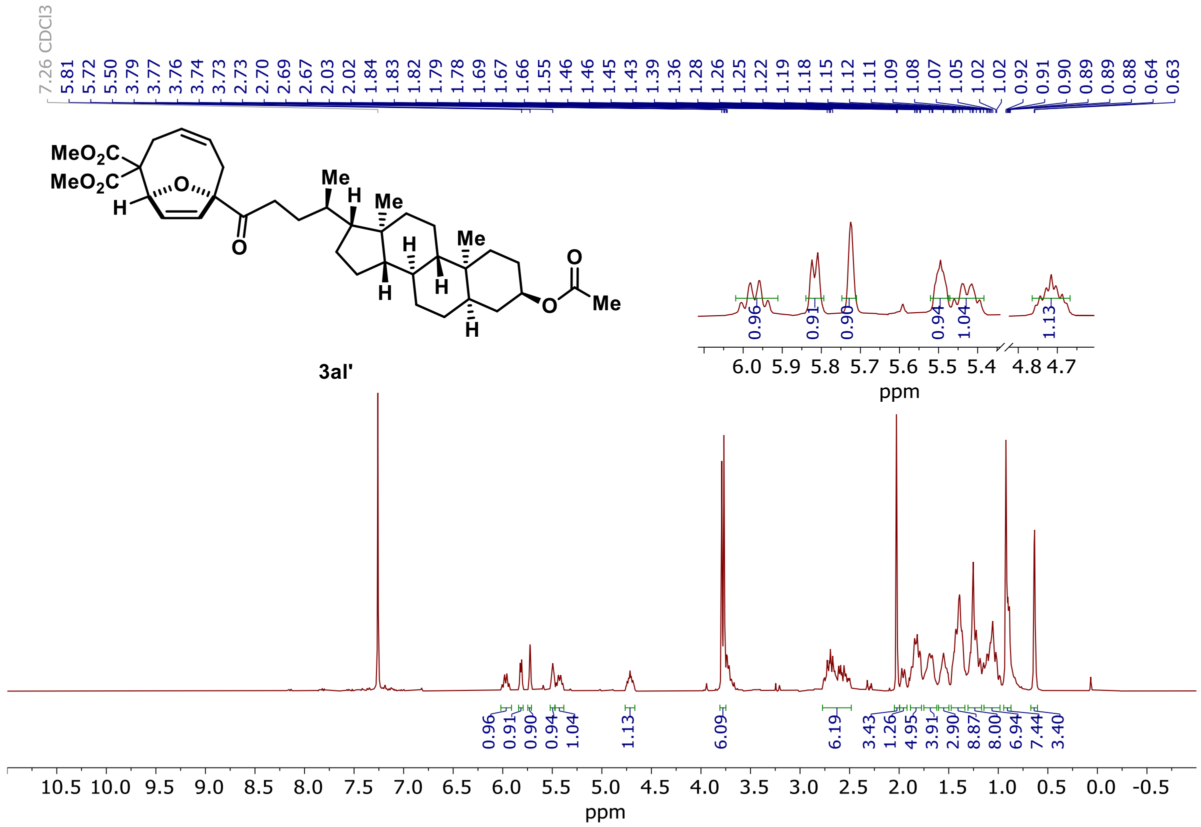

^{13}C NMR (151 MHz, CDCl$_3$) of **3al’**

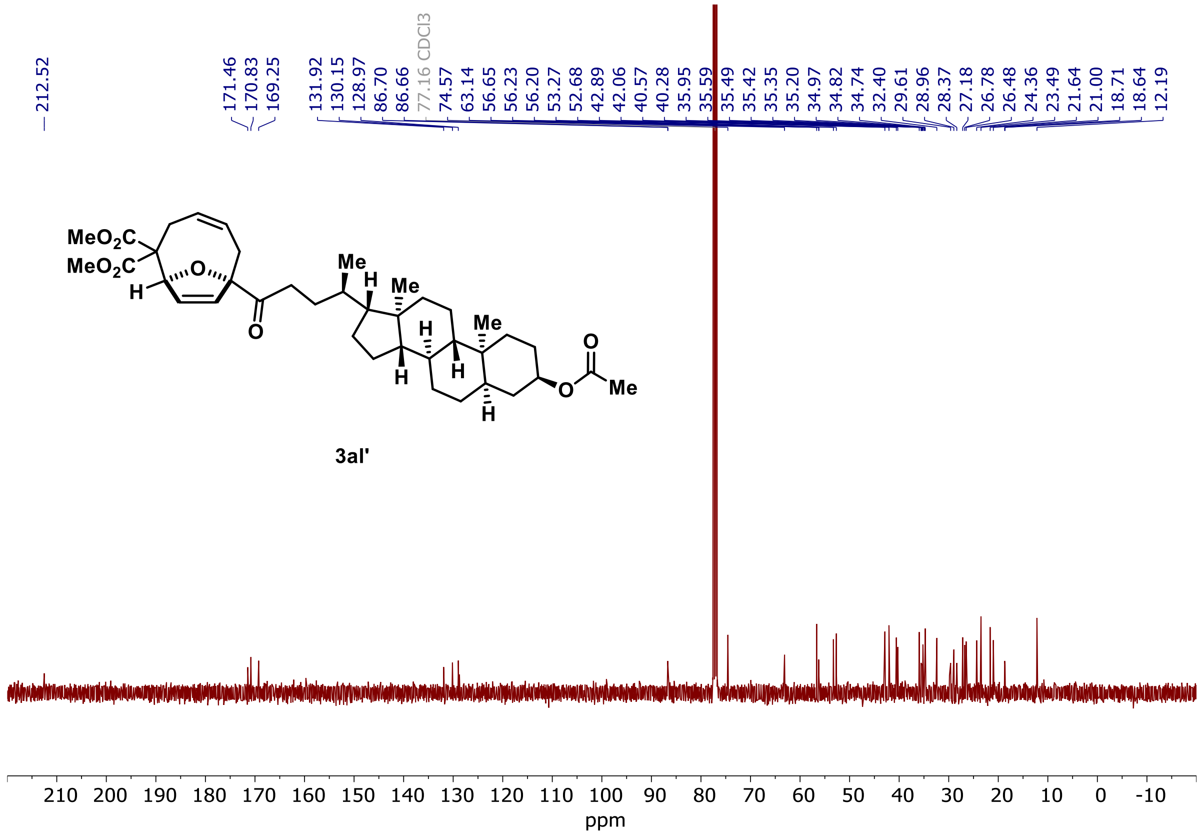

^{1}H NMR (400 MHz, CDCl$_3$) of **3am**

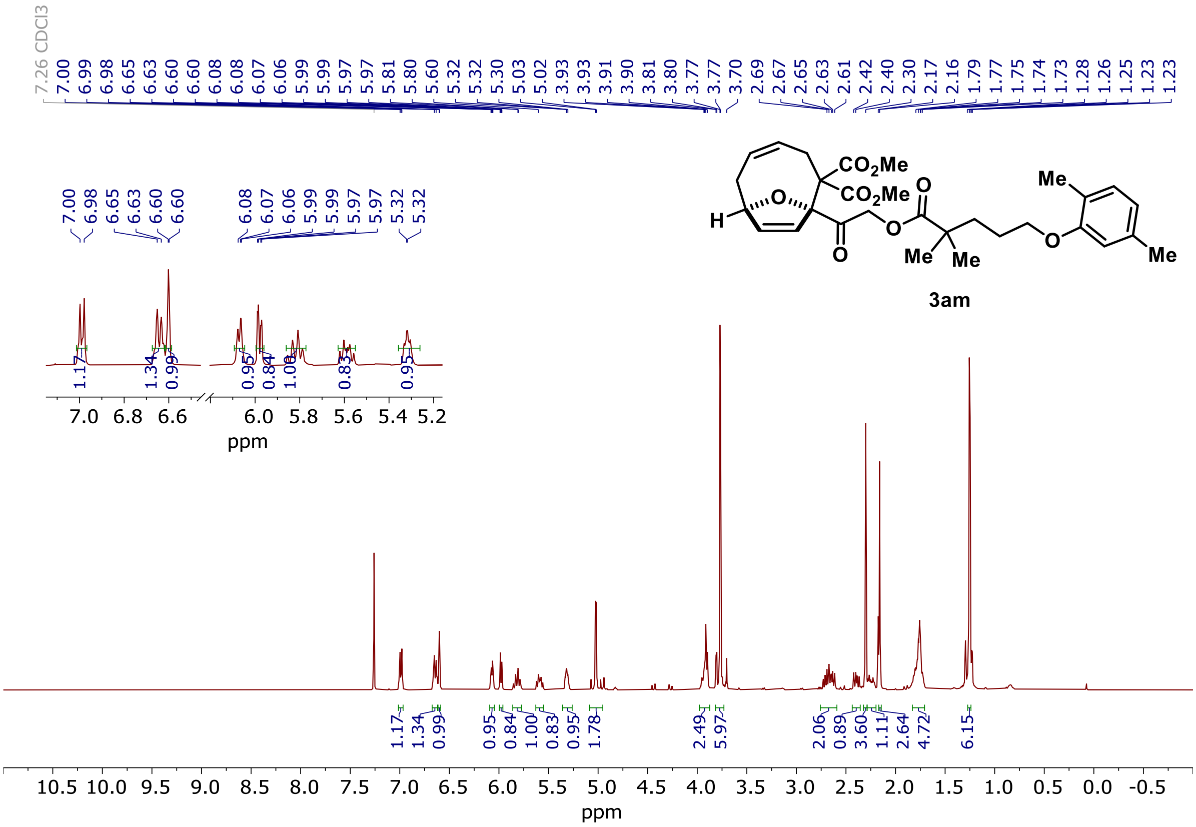

^{13}C NMR (151 MHz, CDCl$_3$) of **3am**

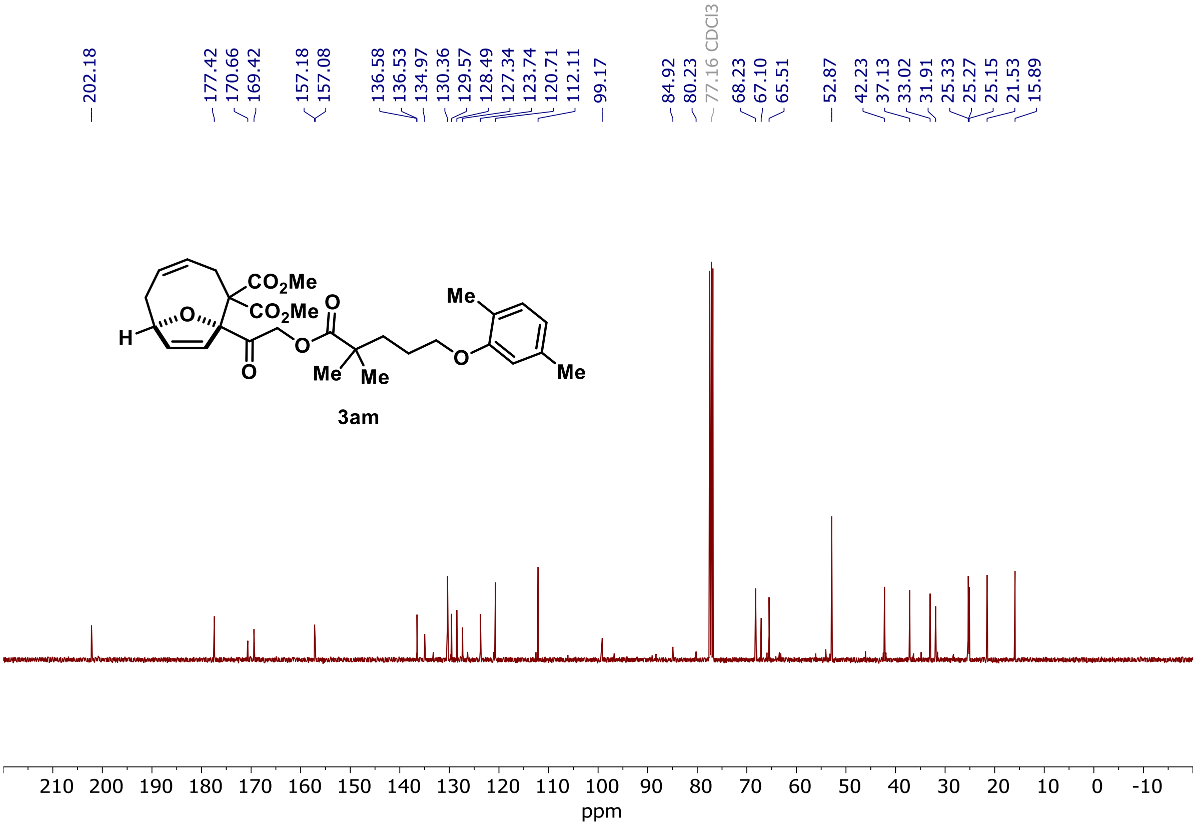

^{1}H NMR (400 MHz, $CDCl_3$) of **3am’**

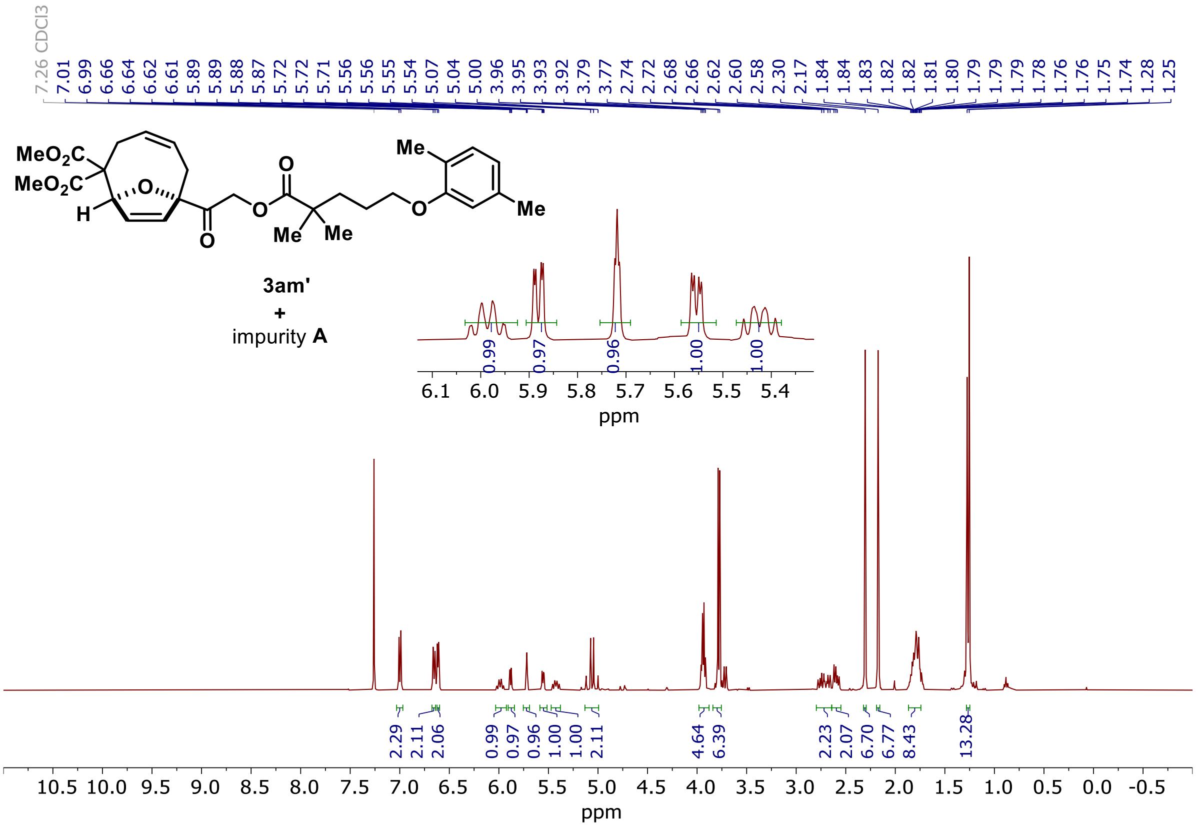

^{13}C NMR (151 MHz, $CDCl_3$) of **3am’**

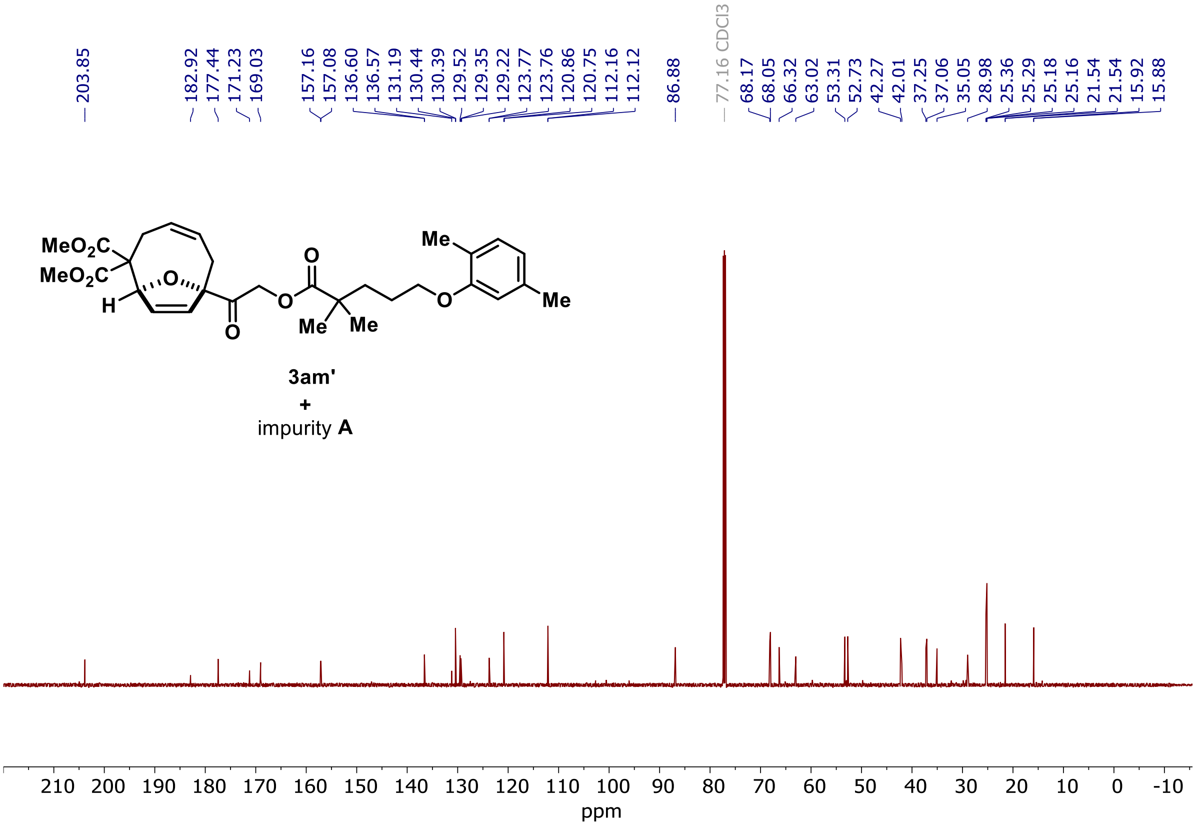

^{1}H NMR (400 MHz, $CDCl_3$) of **3an**

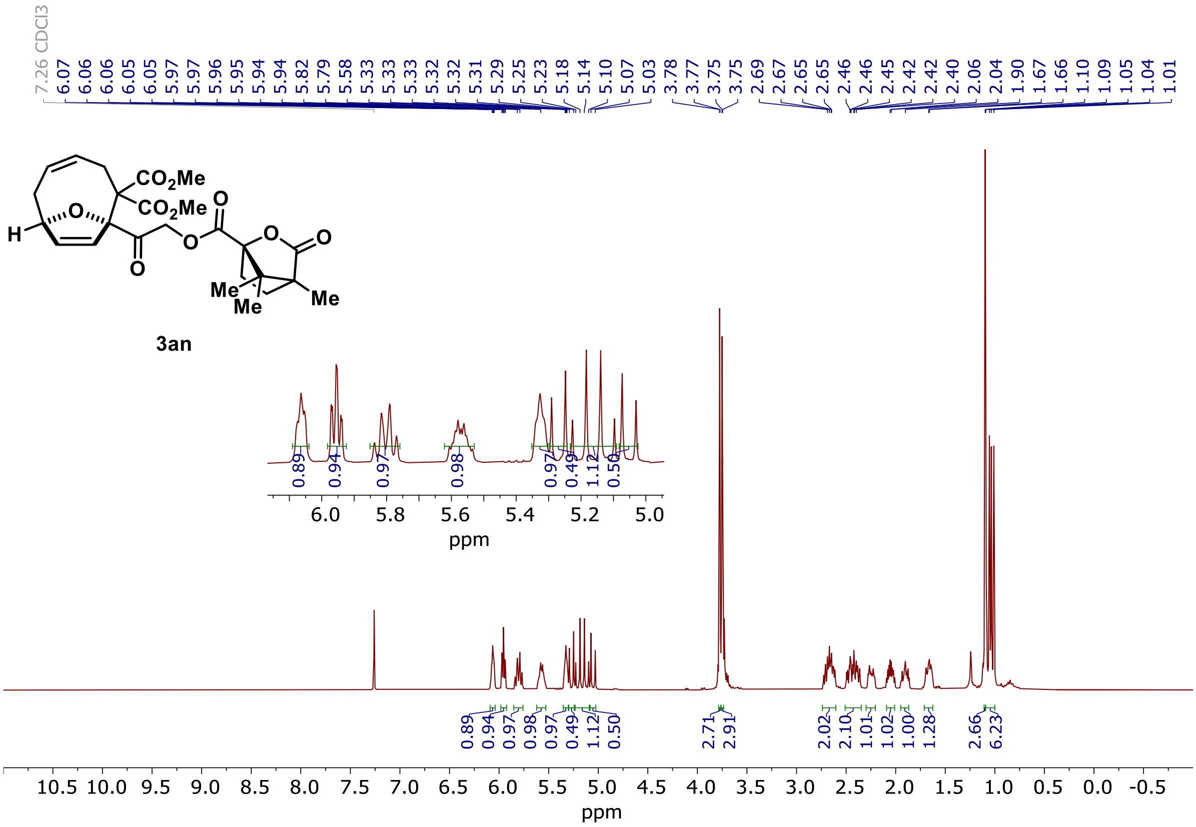

^{13}C NMR (101 MHz, $CDCl_3$) of **3an**

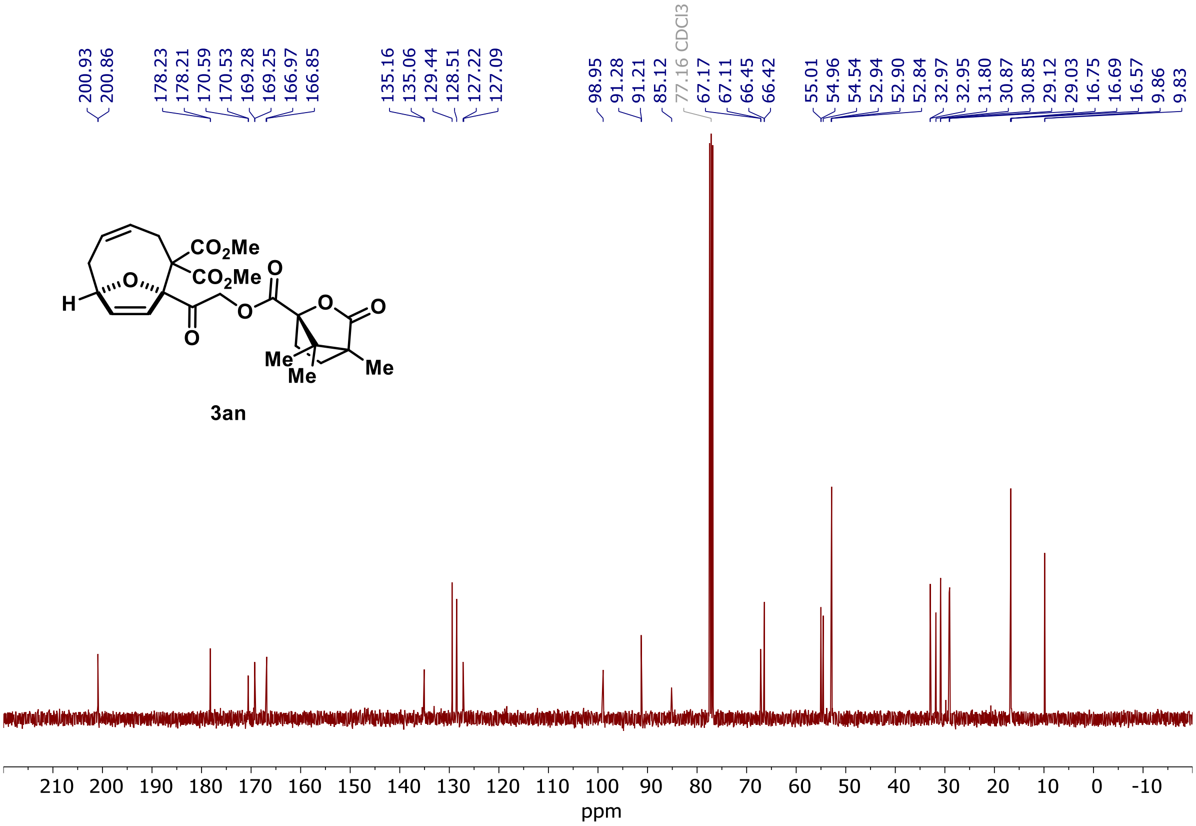

^{1}H NMR (400 MHz, $CDCl_3$) of **3an’**

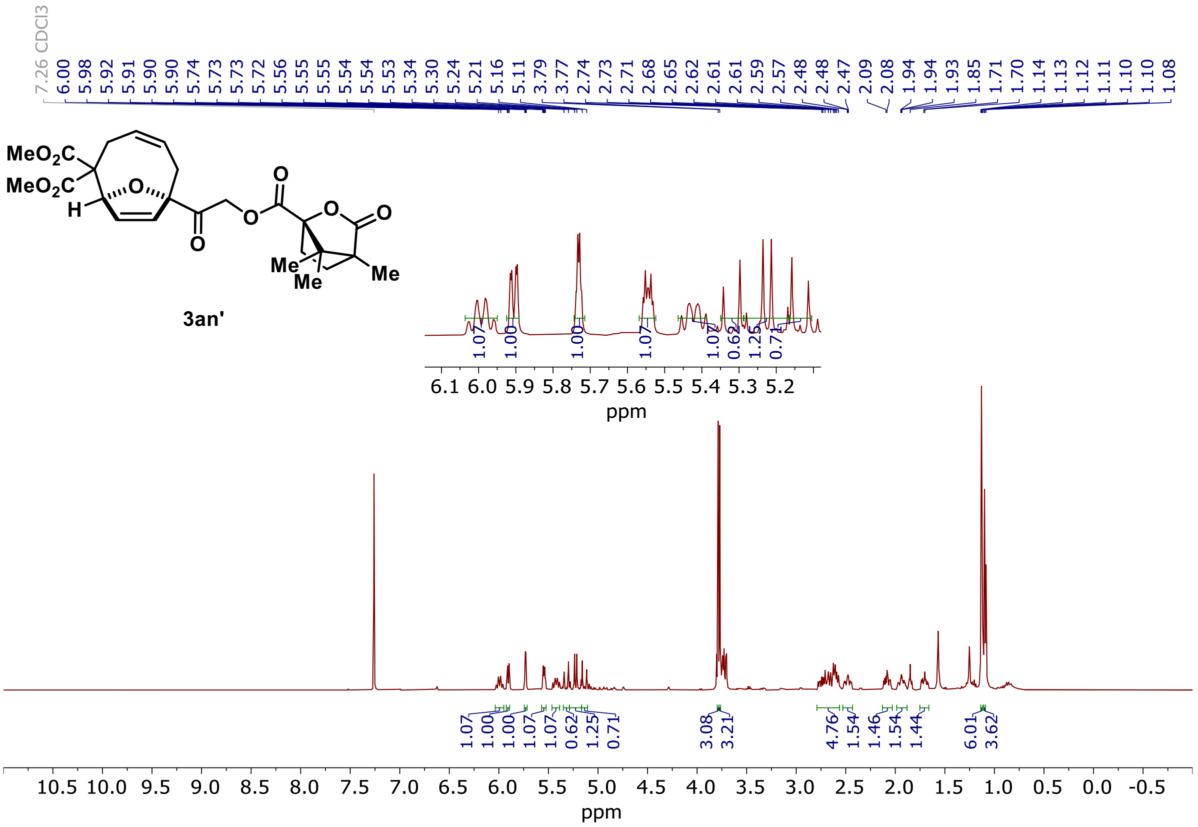

^{13}C NMR (101 MHz, $CDCl_3$) of **3an’**

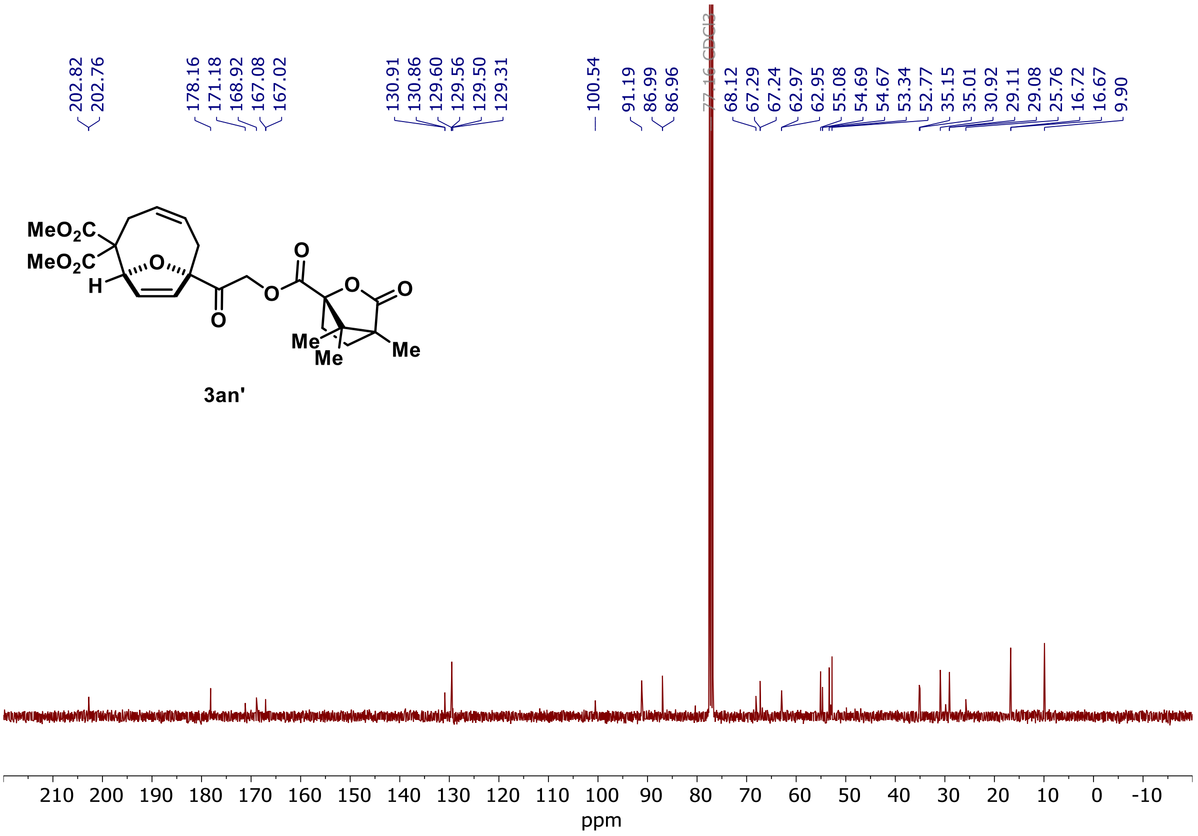

^{1}H NMR (400 MHz, $CDCl_3$) of **3ao**

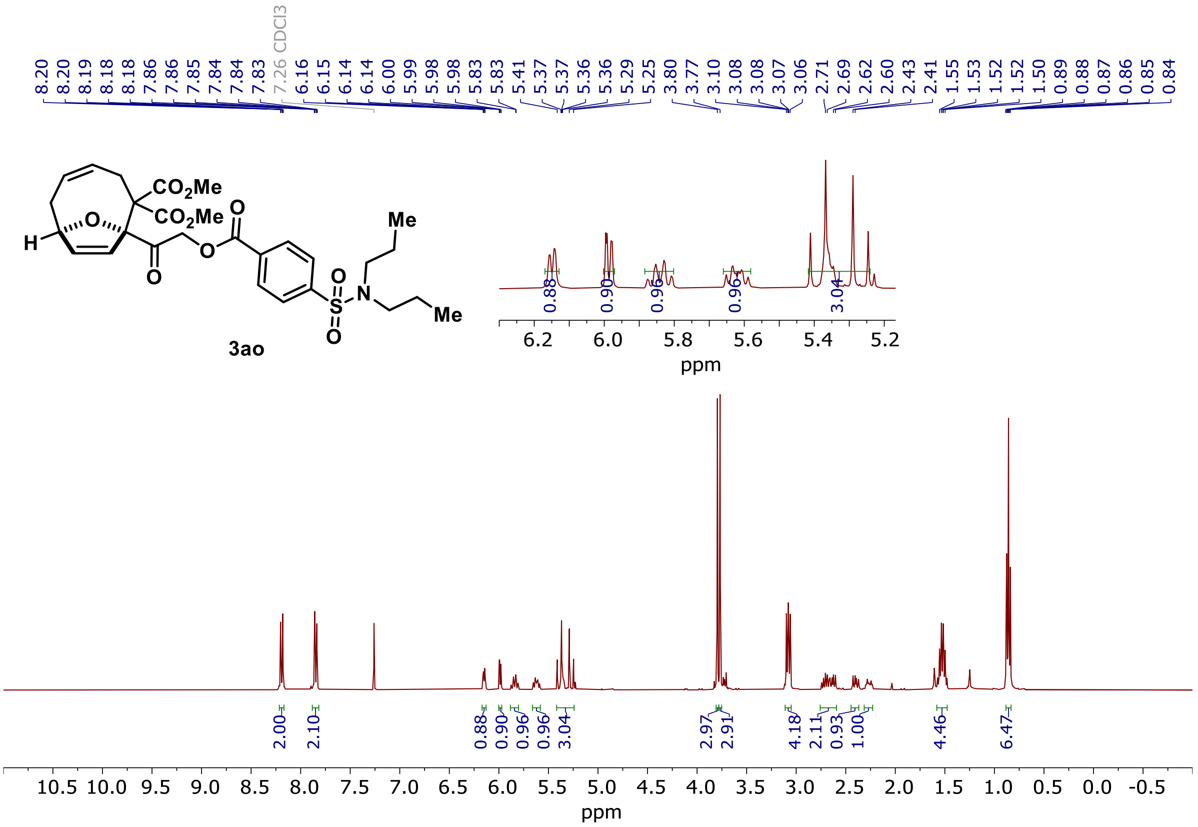

^{13}C NMR (101 MHz, $CDCl_3$) of **3ao**

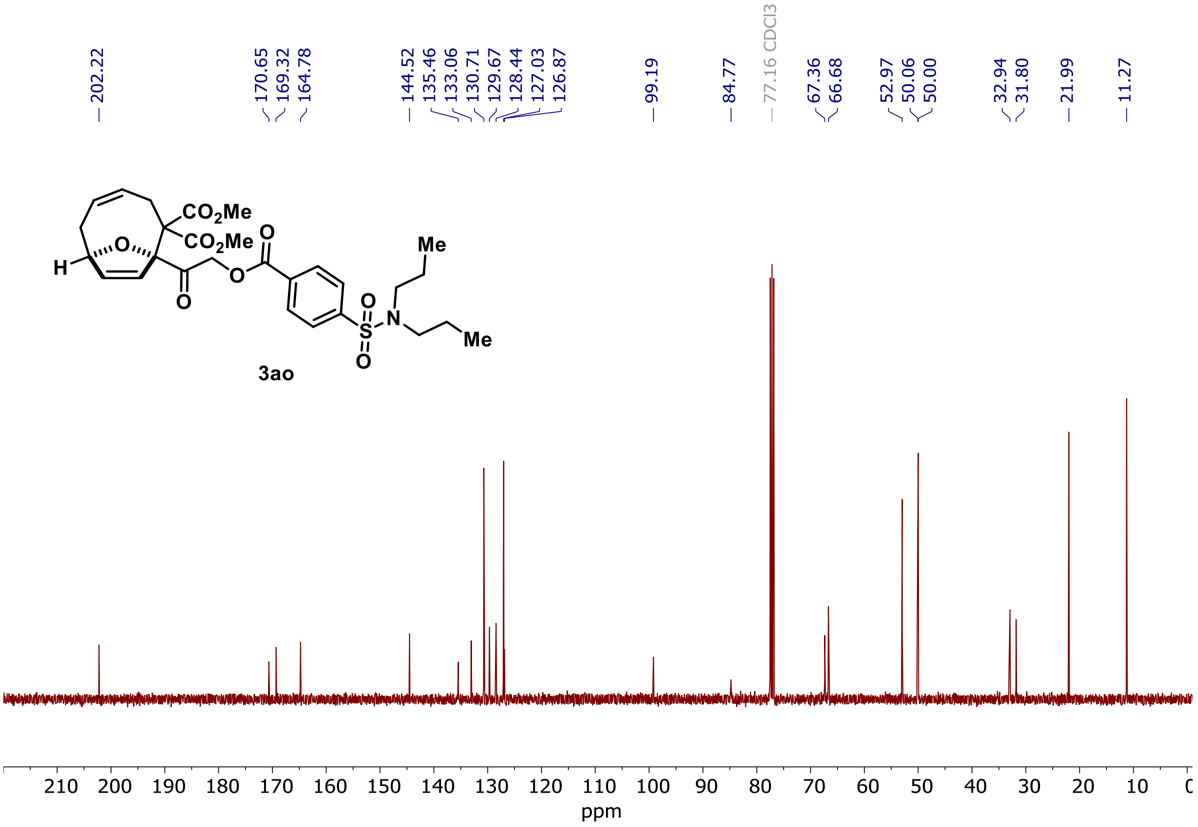

^{1}H NMR (400 MHz, CDCl$_3$) of **3ao’**

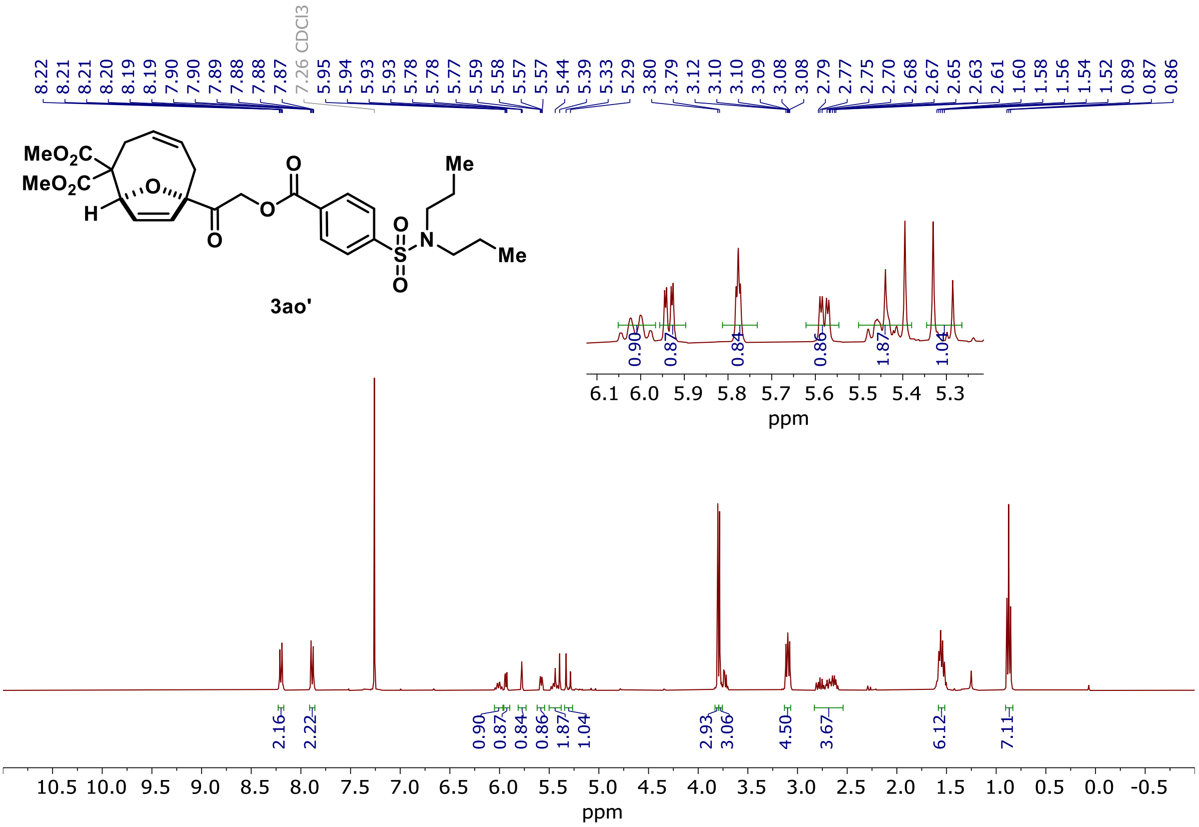

^{13}C NMR (151 MHz, CDCl$_3$) of **3ao’**

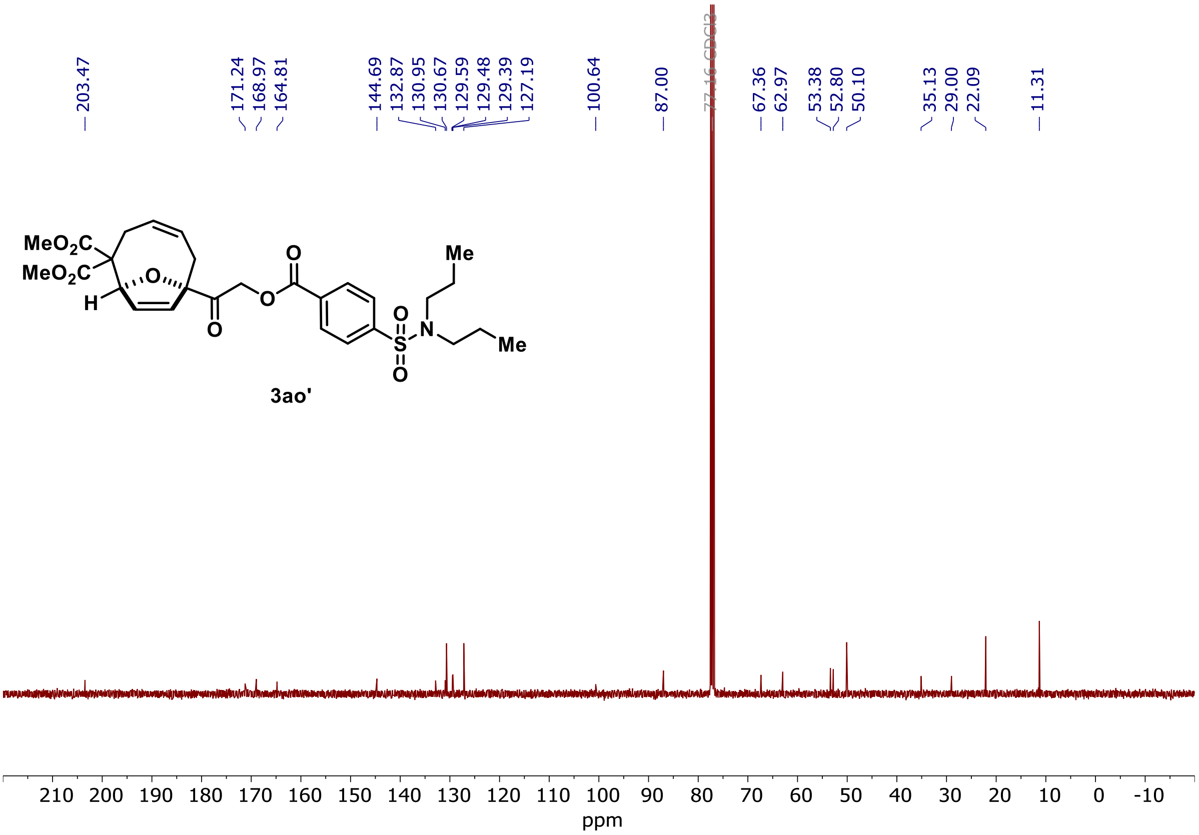

^{1}H NMR (400 MHz, $CDCl_3$) of **3ap**

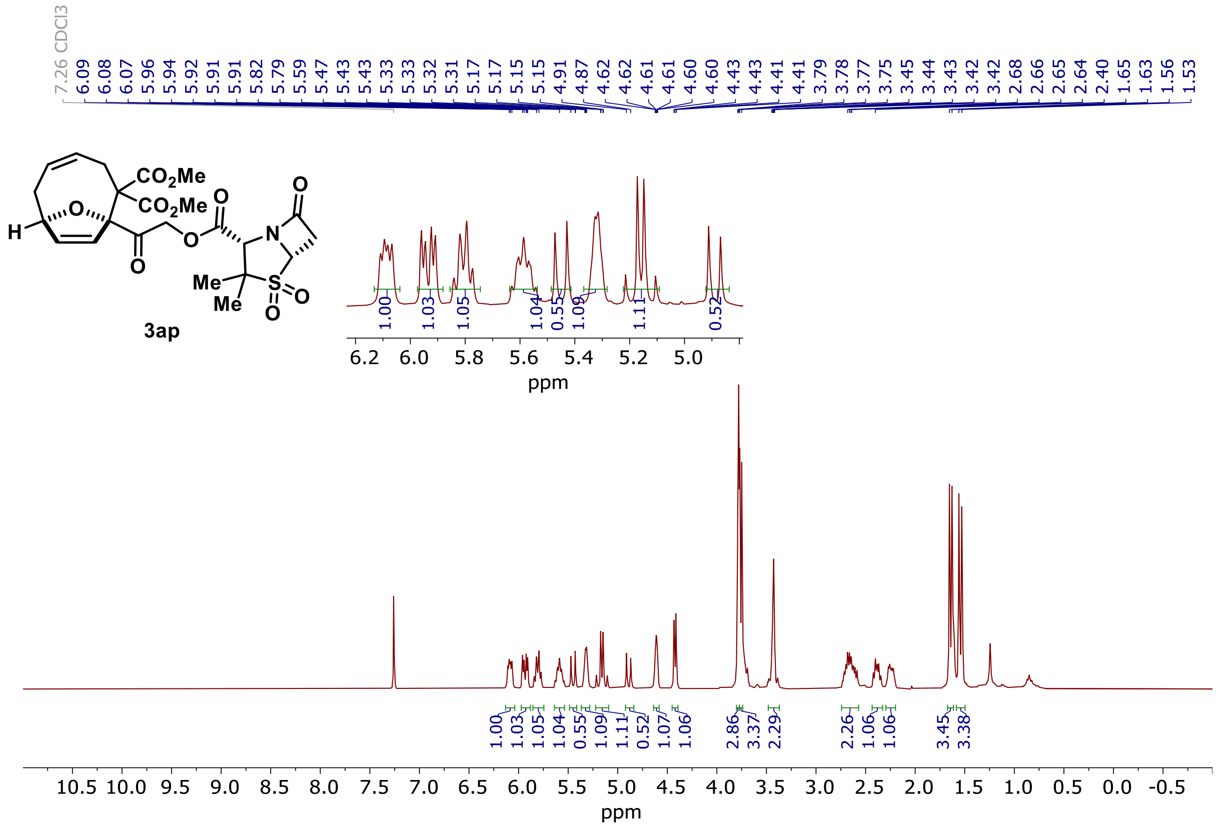

^{13}C NMR (101 MHz, $CDCl_3$) of **3ap**

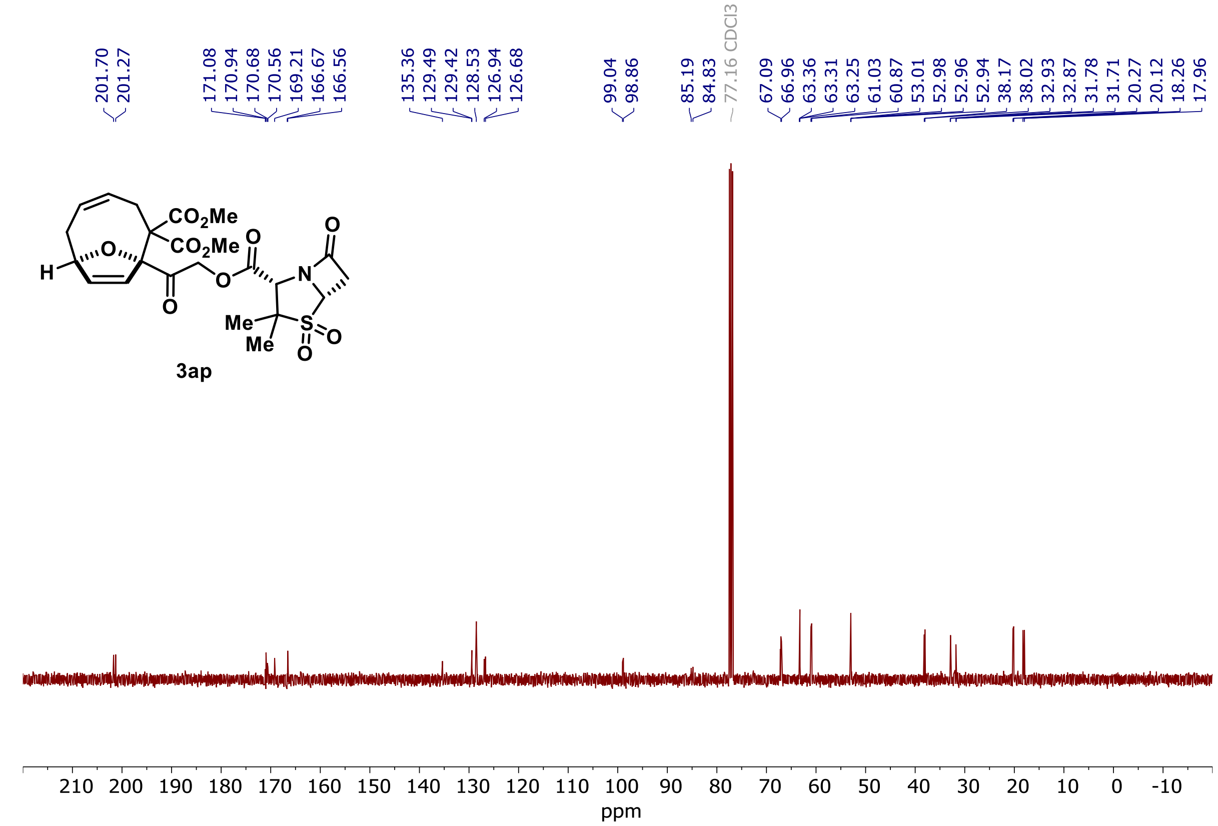

^{1}H NMR (400 MHz, $CDCl_3$) of **3ap'**

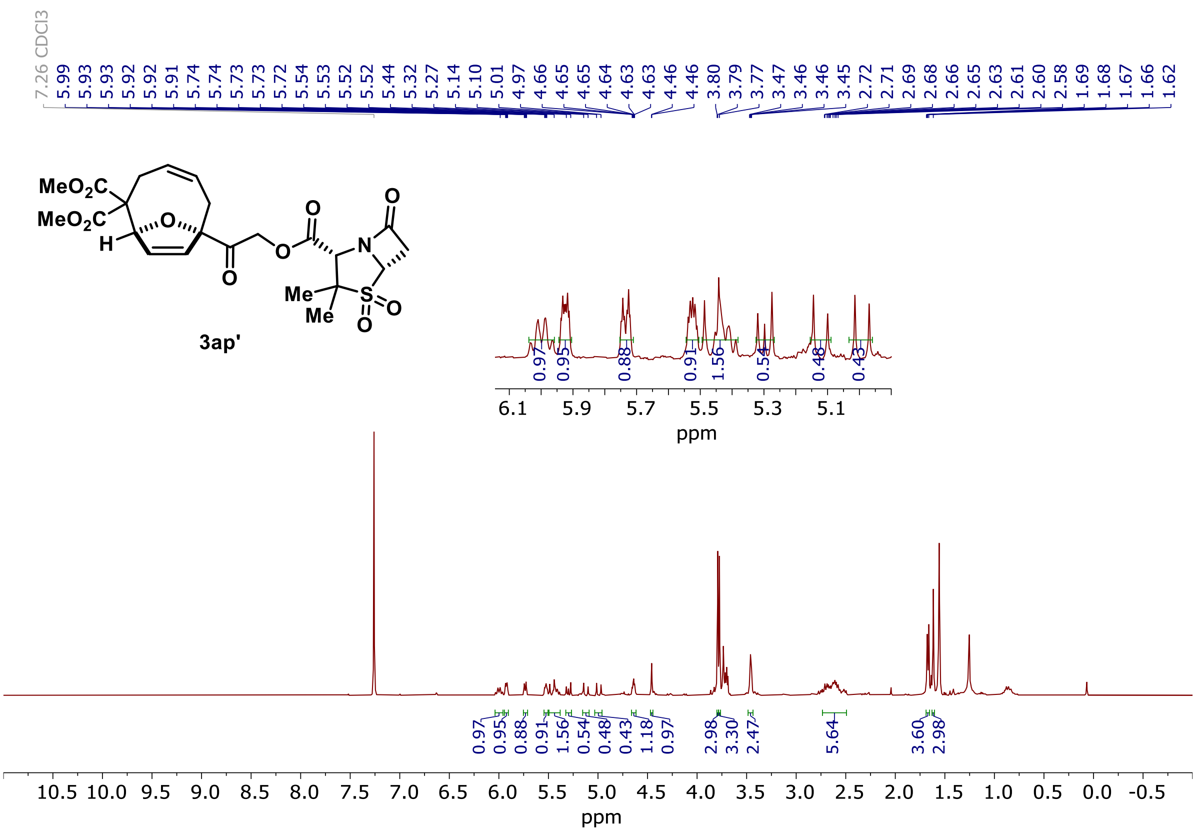

^{13}C NMR (151 MHz, $CDCl_3$) of **3ap'**

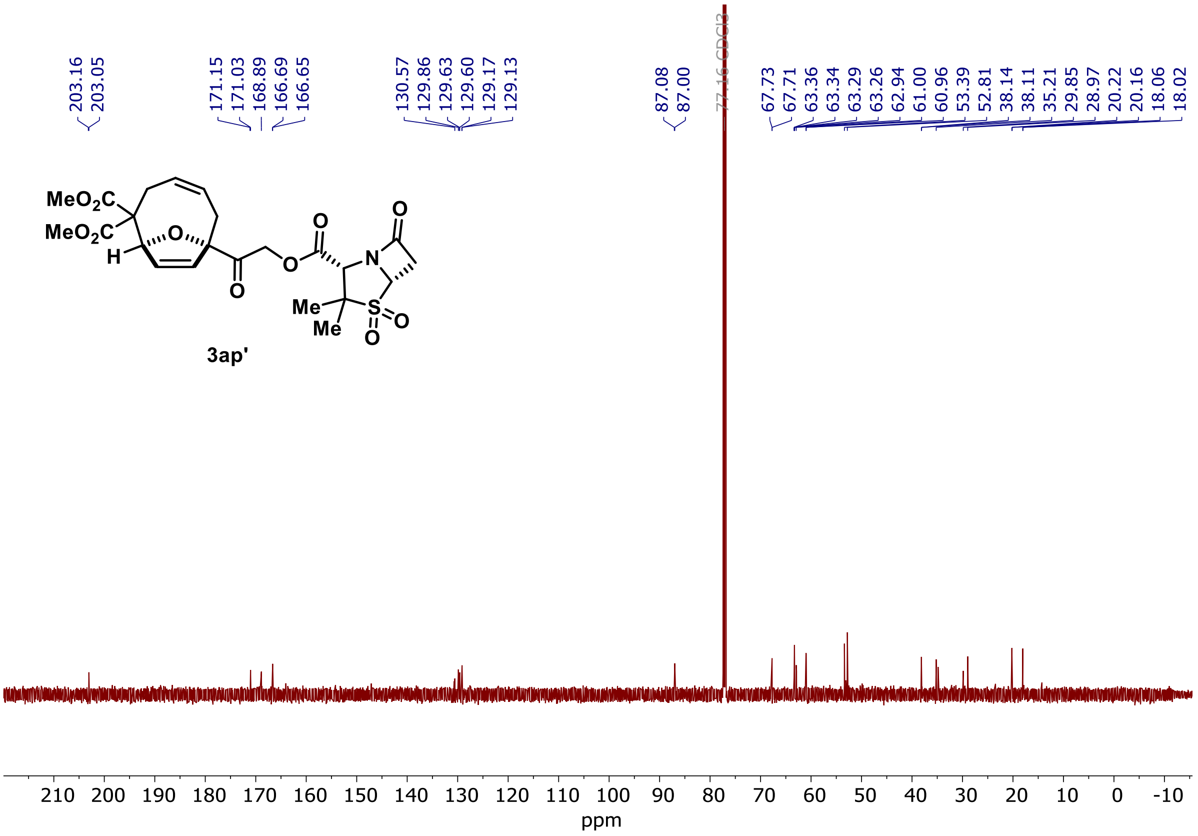

^{1}H NMR (400 MHz, $CDCl_3$) of **3aq** & **3aq'**

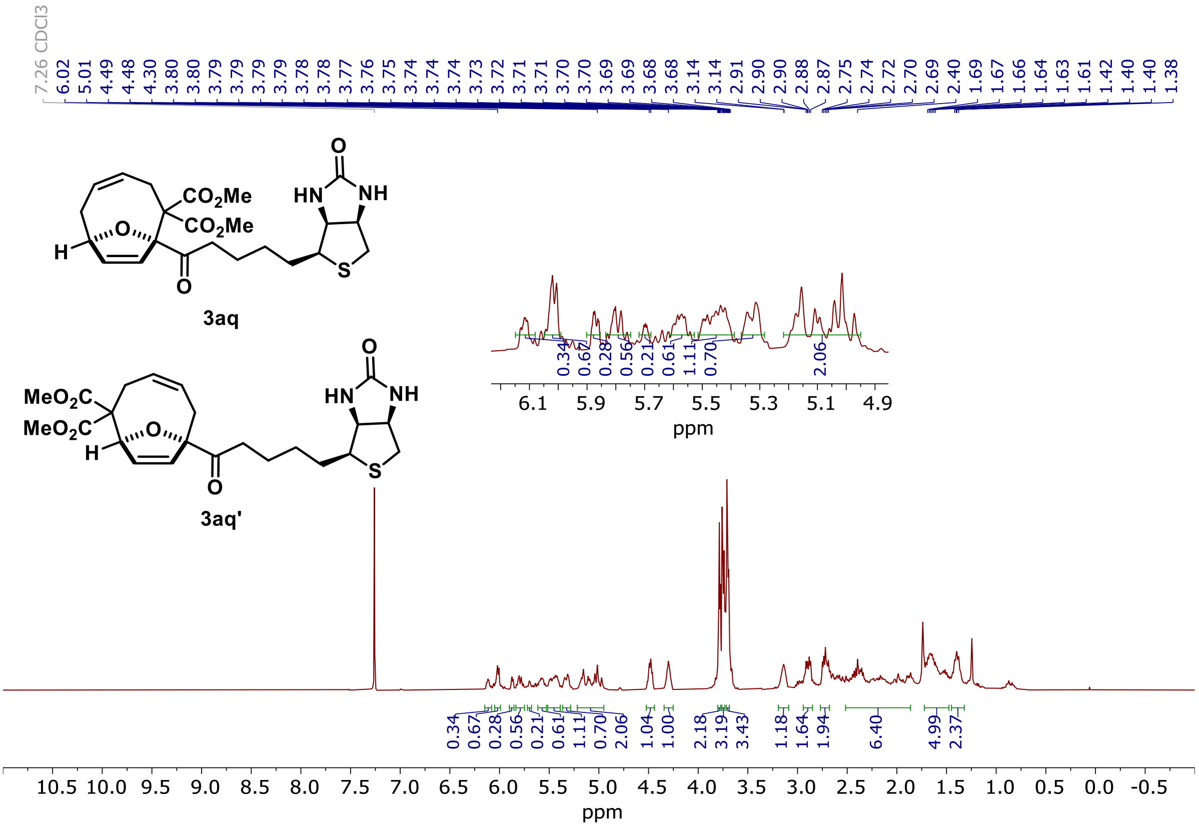

^{13}C NMR (151 MHz, $CDCl_3$) of **3aq** & **3aq'**

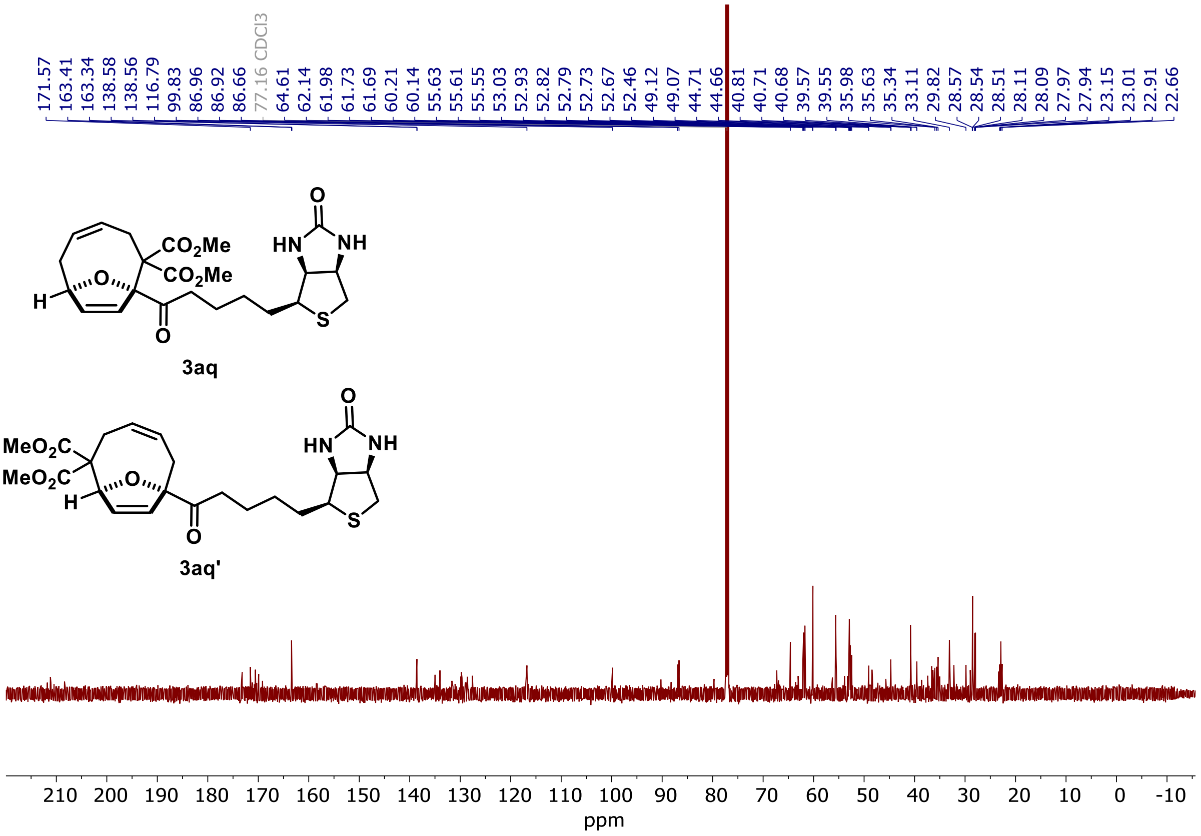

^{1}H NMR (400 MHz, $CDCl_3$) of **4a**

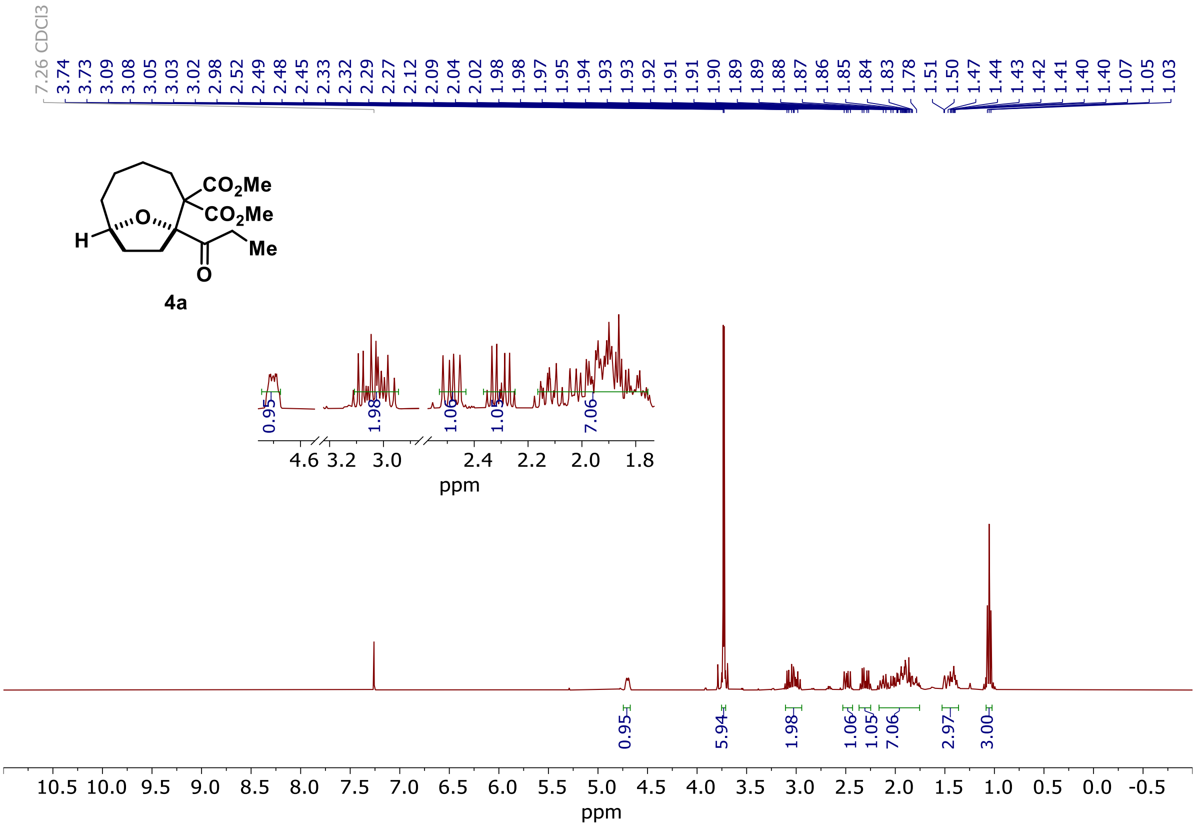

^{13}C NMR (101 MHz, $CDCl_3$) of **4a**

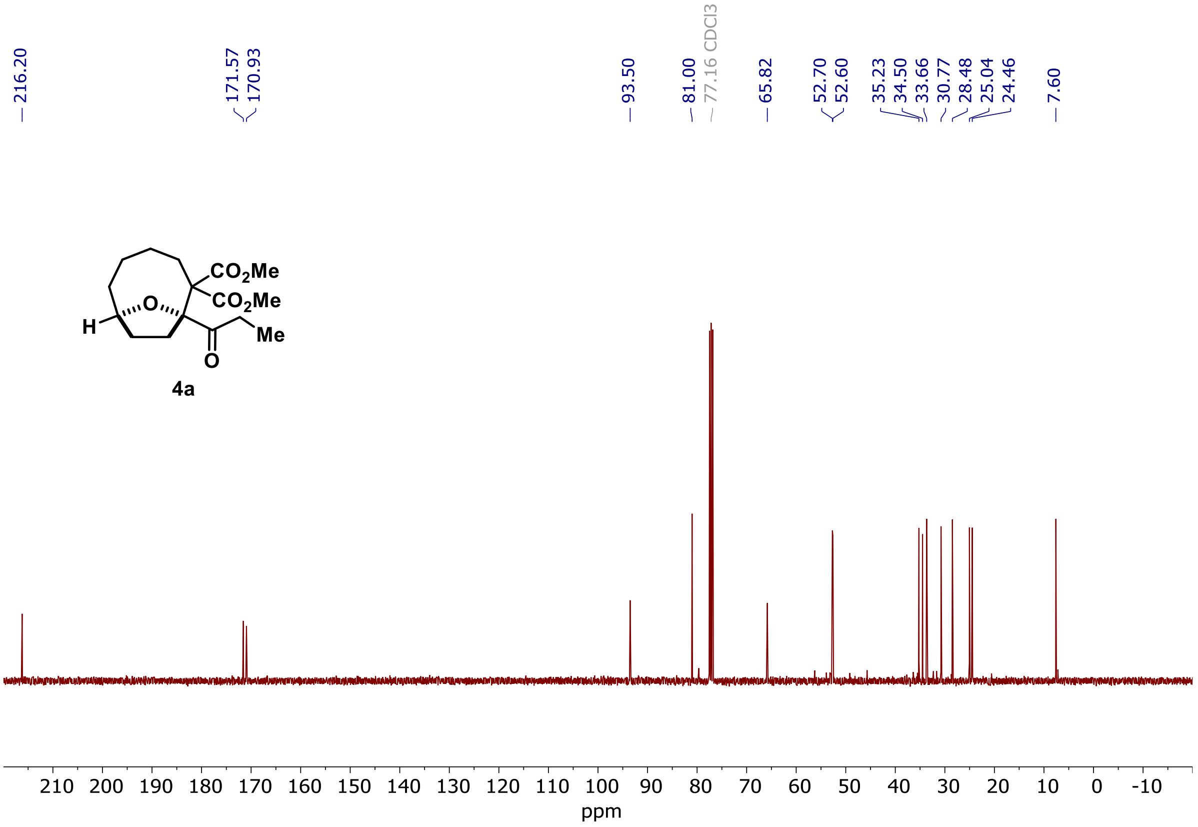

^{1}H NMR (400 MHz, $CDCl_3$) of **4k**

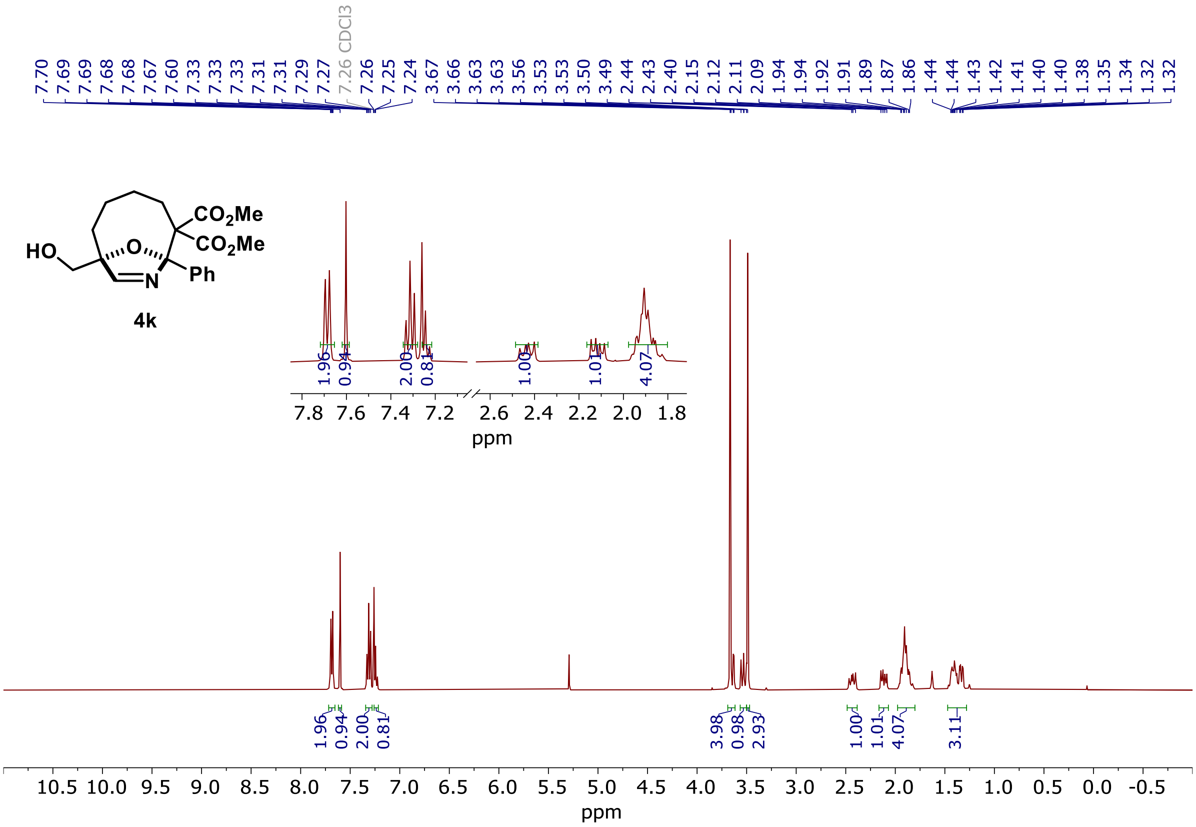

^{13}C NMR (101 MHz, $CDCl_3$) of **4k**

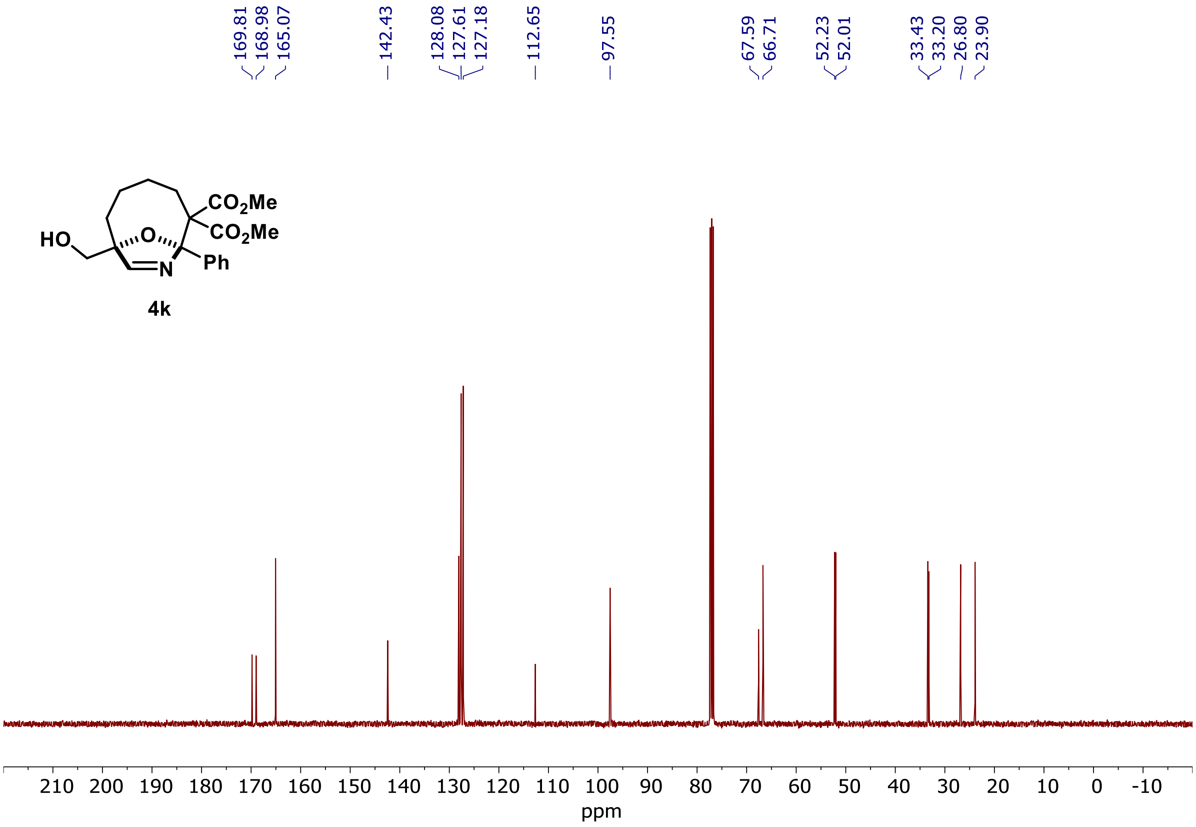

^{1}H NMR (599 MHz, CDCl$_3$) of **4k’**

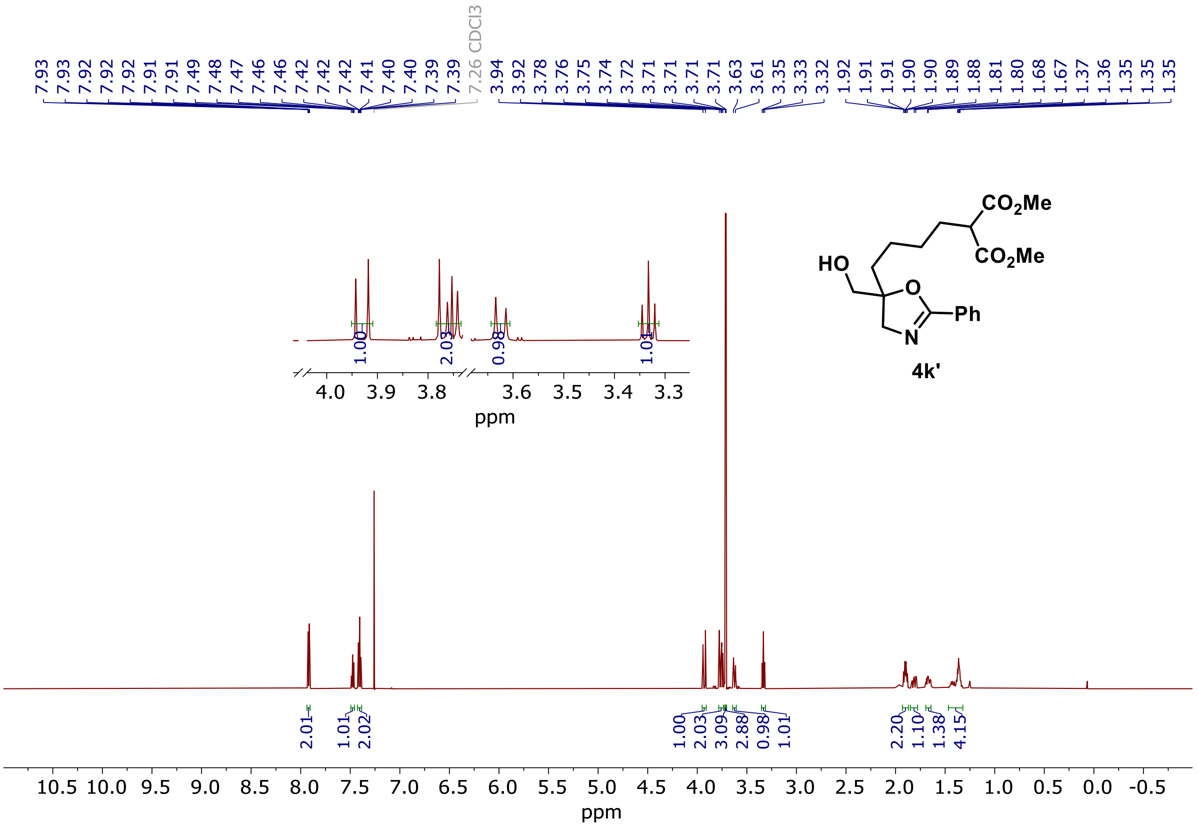

^{13}C NMR (151 MHz, CDCl$_3$) of **4k’**

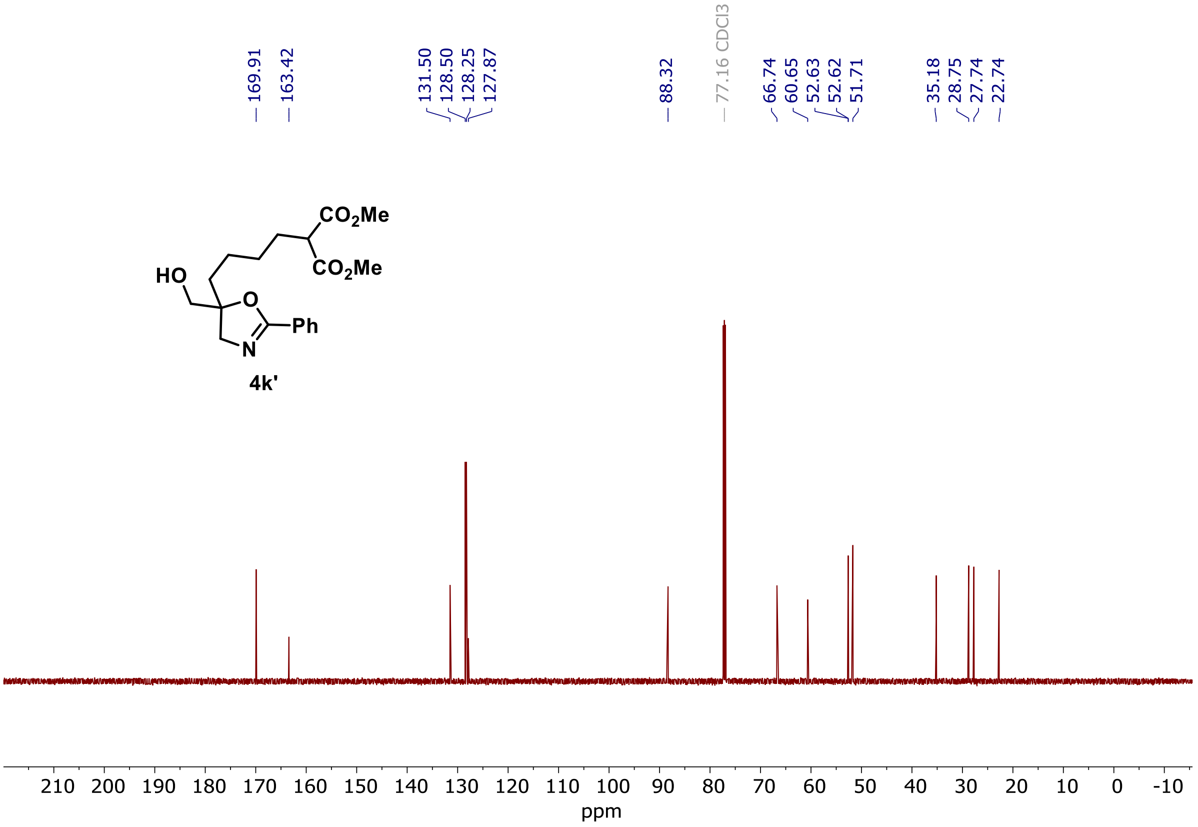

^{1}H NMR (400 MHz, $CDCl_3$) of **4c**

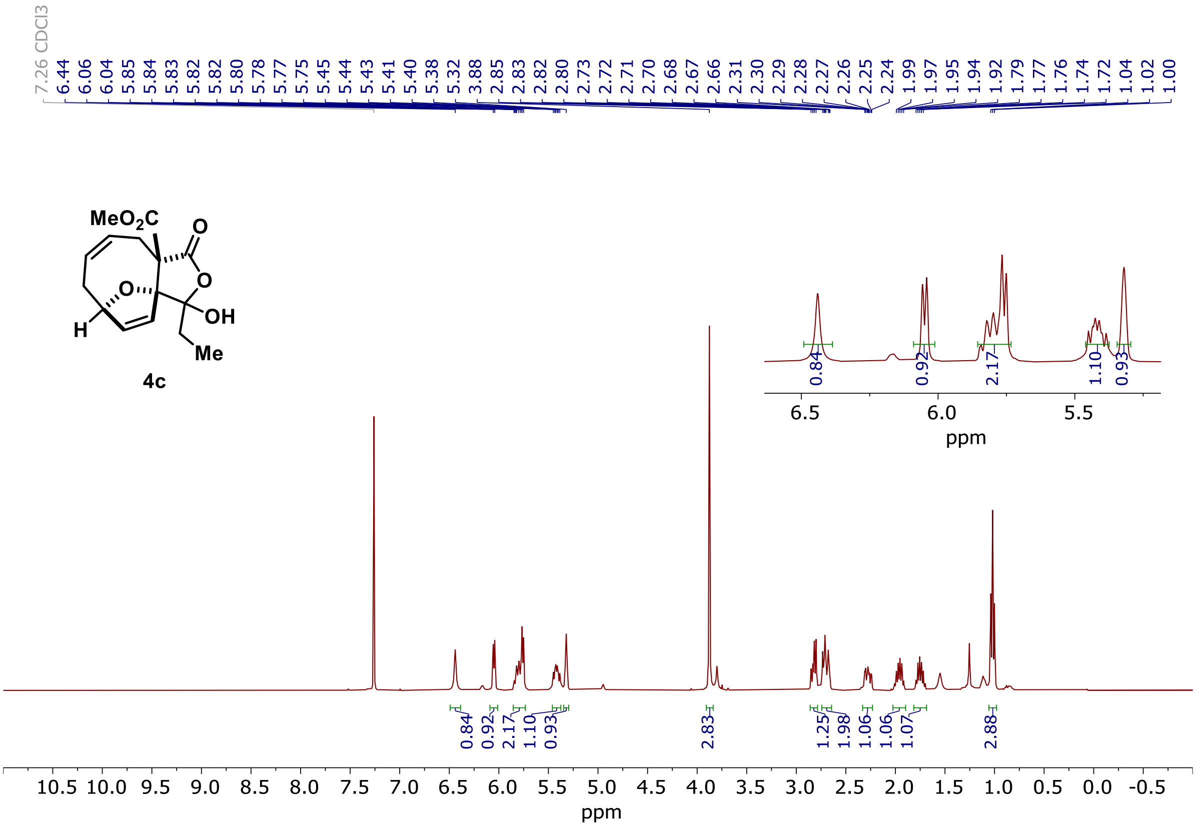

^{13}C NMR (101 MHz, $CDCl_3$) of **4c**

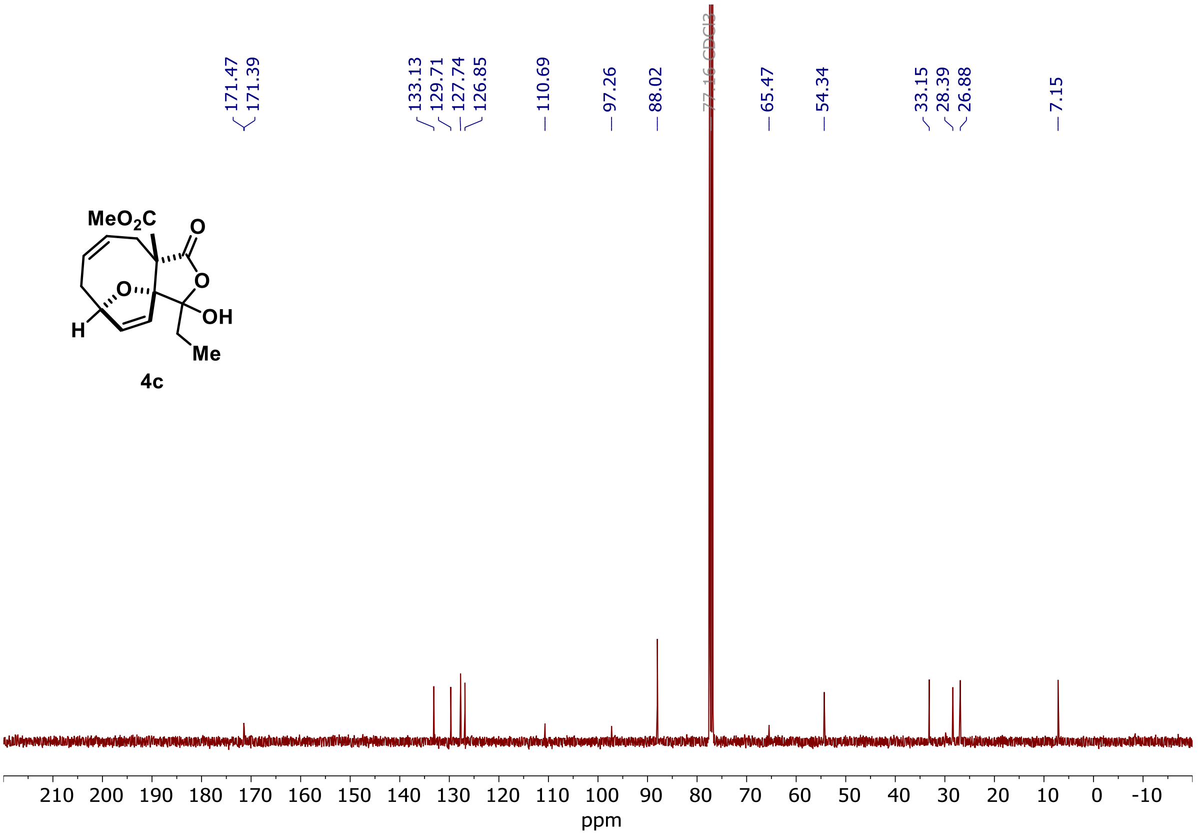

^{1}H NMR (599 MHz, $CDCl_3$) of **4d**

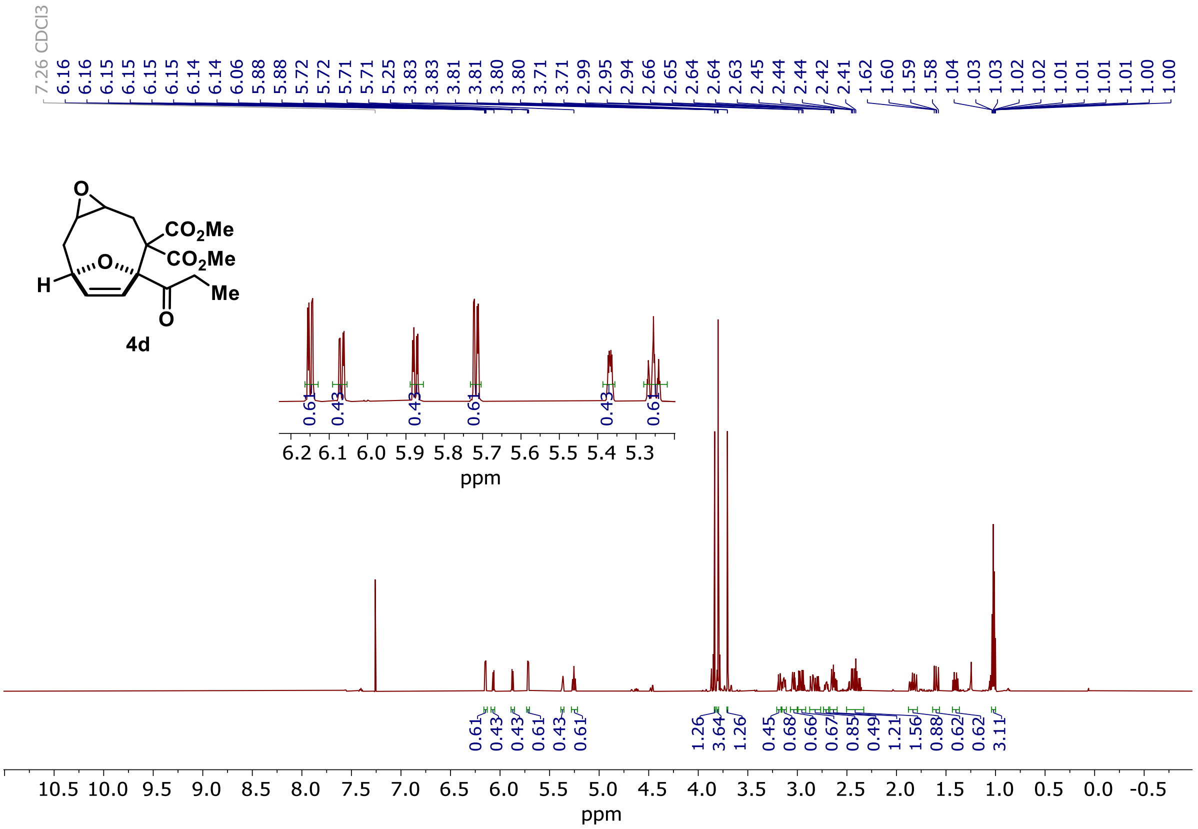

^{13}C NMR (151 MHz, $CDCl_3$) of **4d**

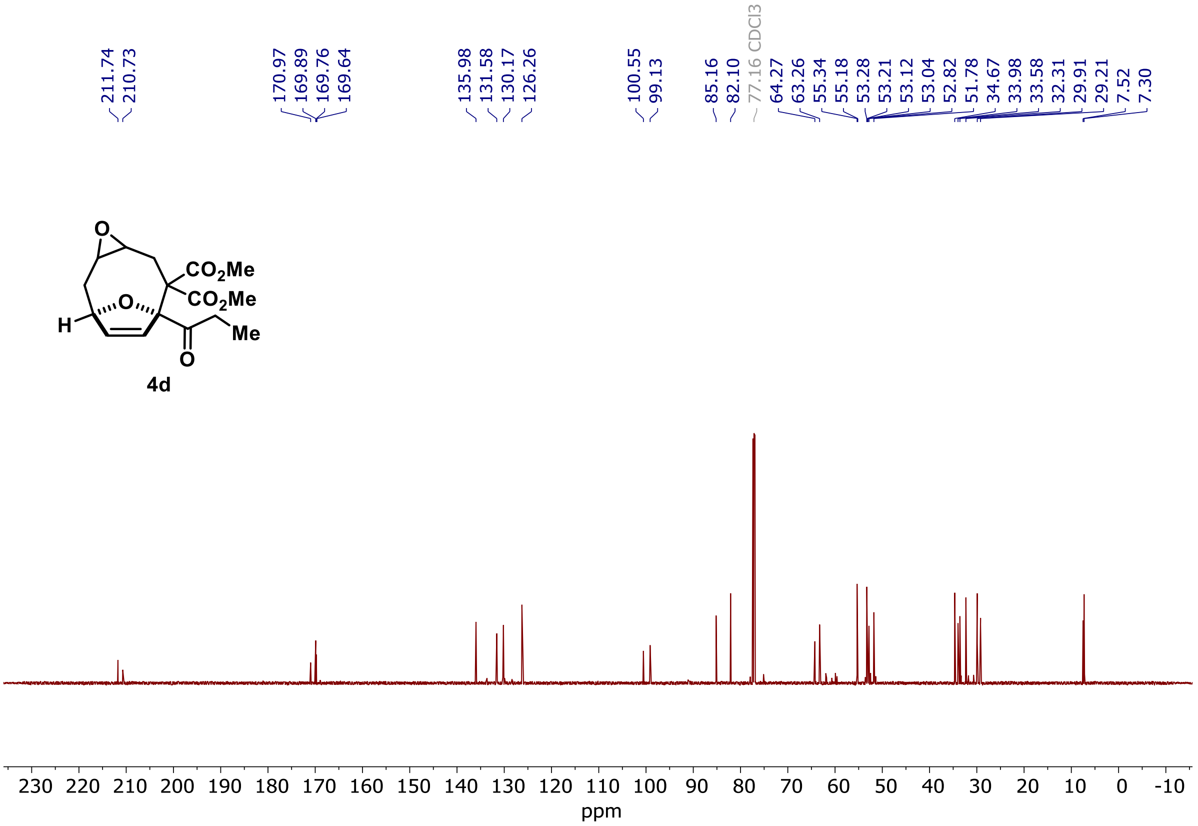

^{1}H NMR (400 MHz, $CDCl_3$) of **4e**

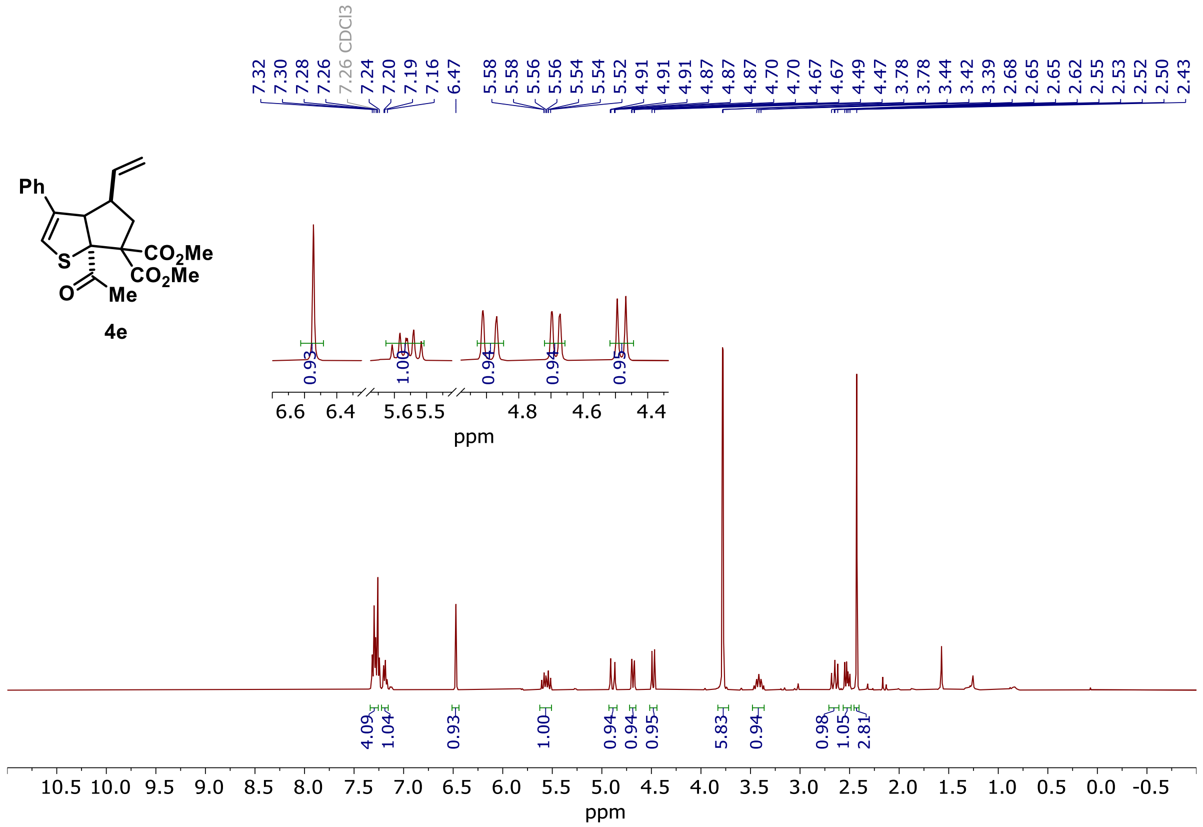

^{13}C NMR (101 MHz, $CDCl_3$) of **4e**

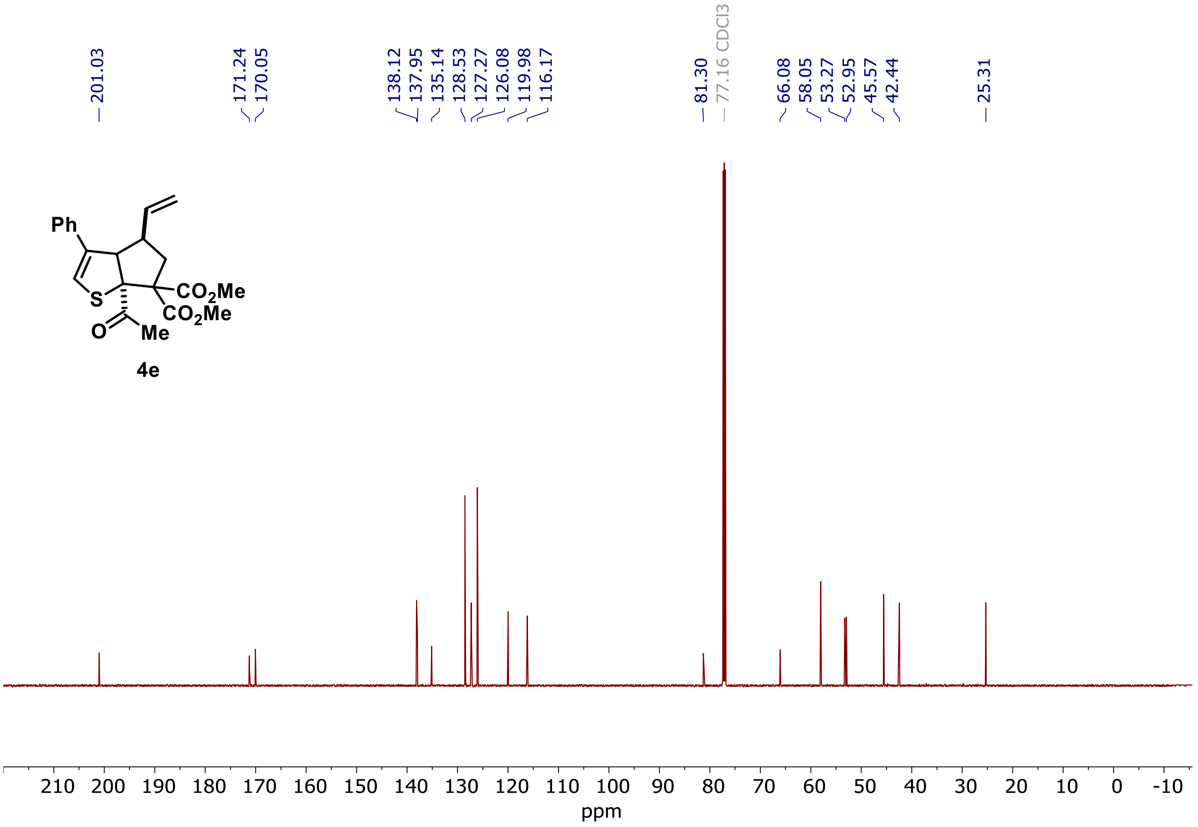

^{1}H NMR (400 MHz, $CDCl_3$) of **3as**

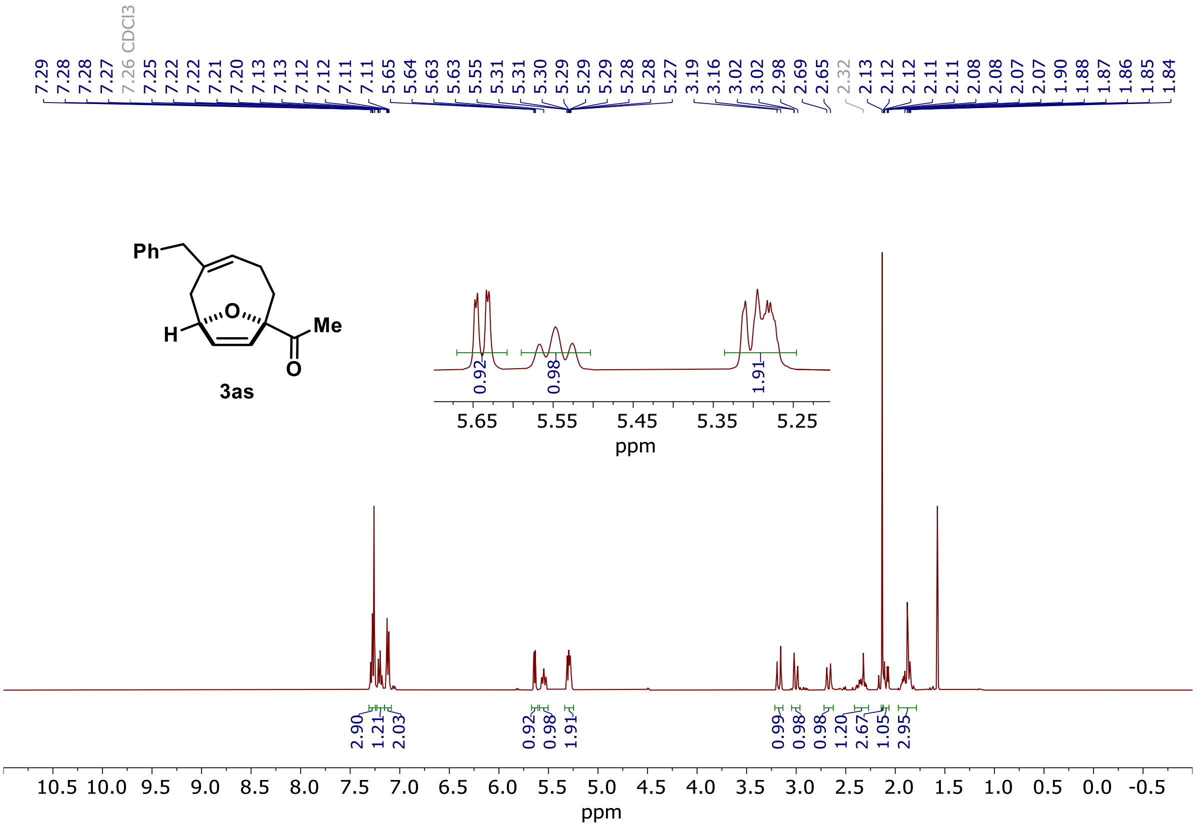

^{13}C NMR (101 MHz, $CDCl_3$) of **3as**

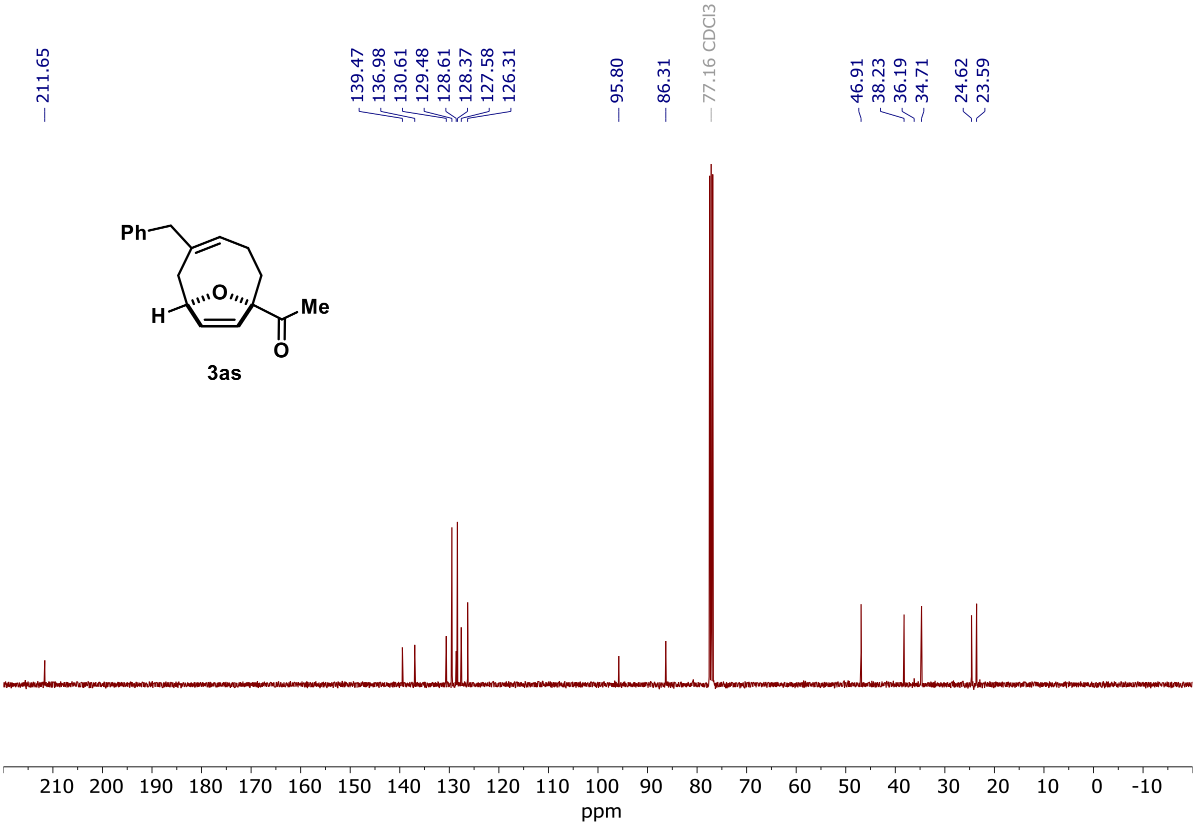

^{1}H NMR (400 MHz, $CDCl_3$) of **3as’**

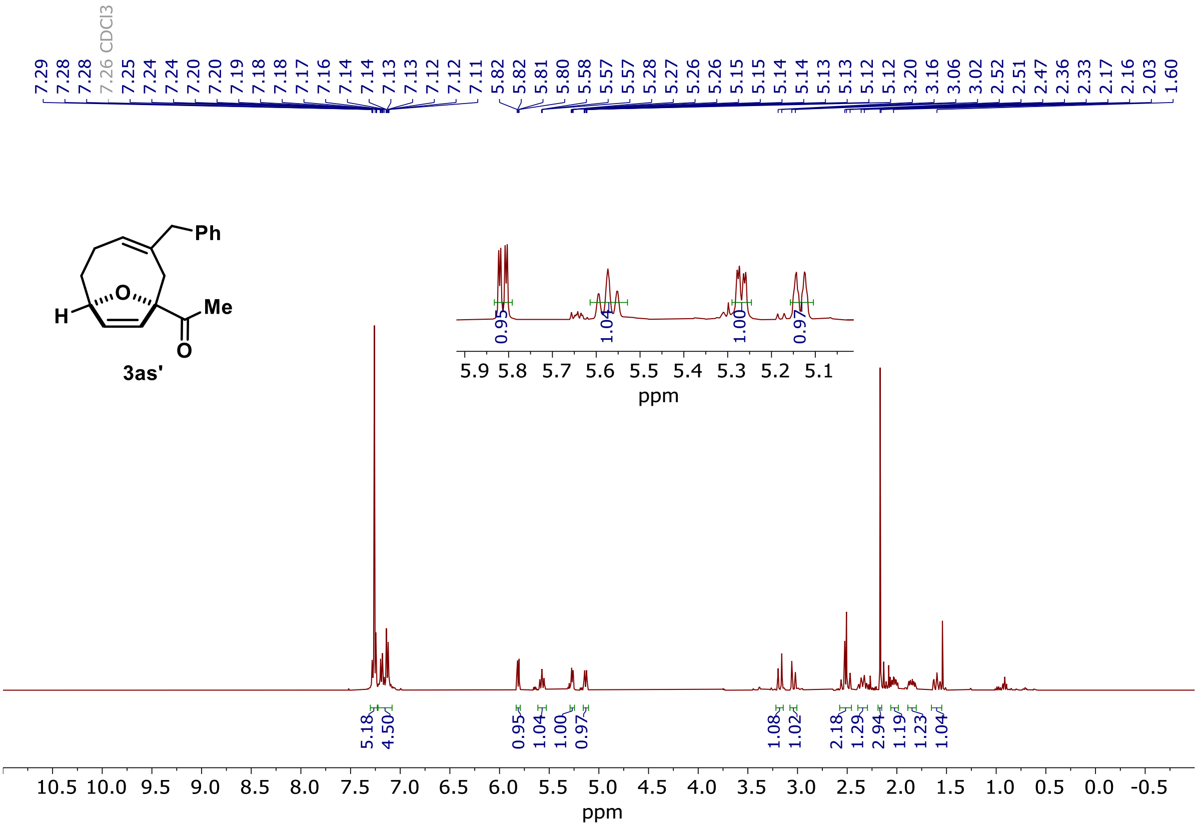

^{13}C NMR (101 MHz, $CDCl_3$) of **3as’**

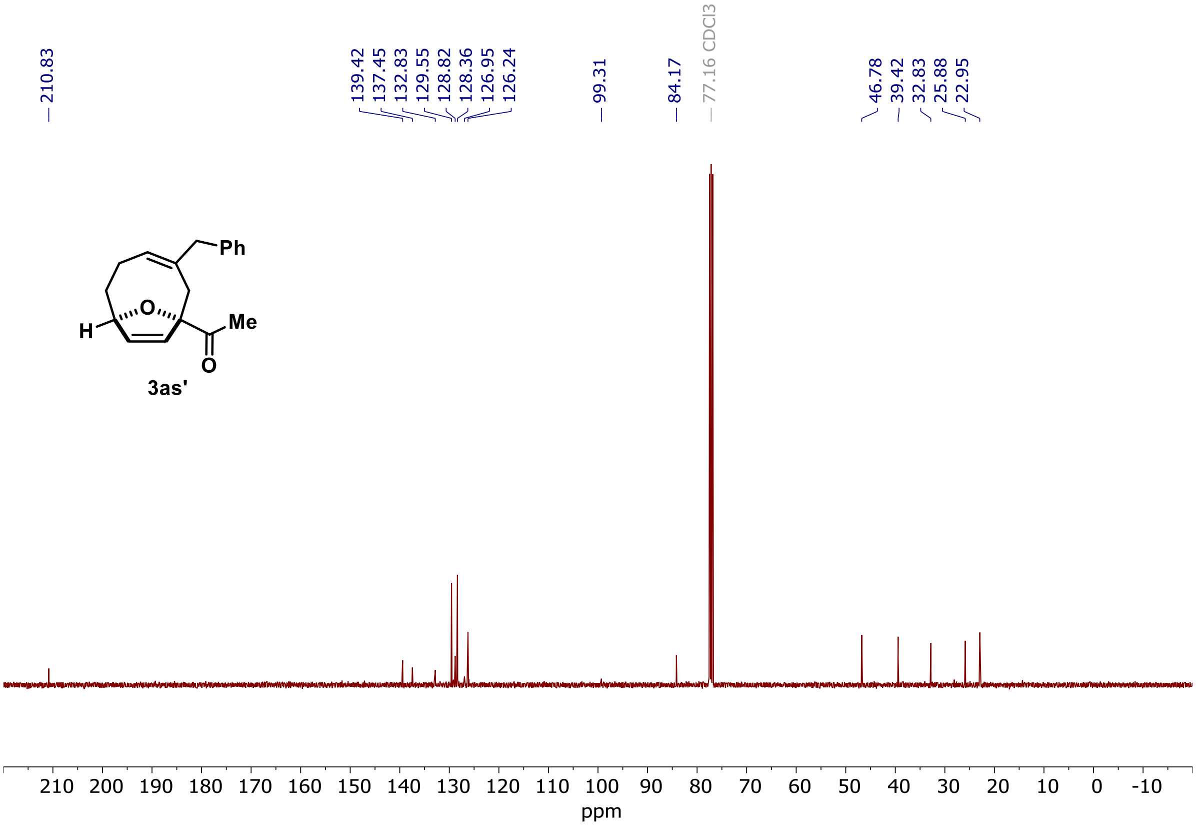

^{1}H NMR (400 MHz, $CDCl_3$) of **4j**

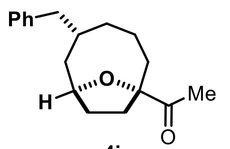

4j

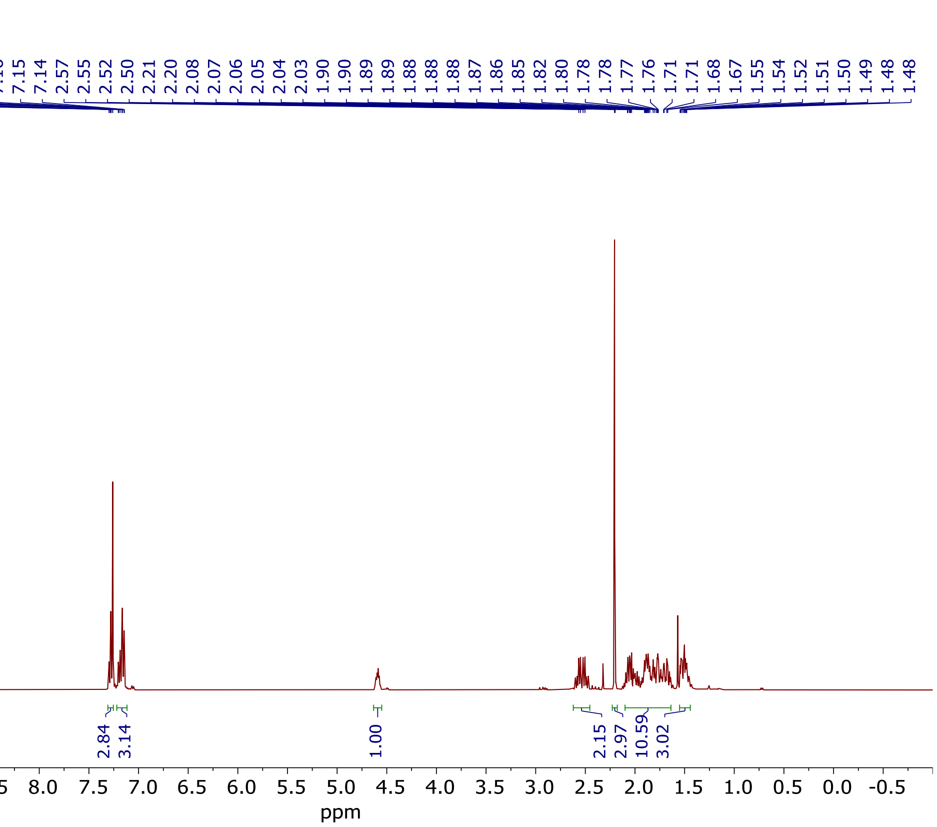

^{13}C NMR (101 MHz, $CDCl_3$) of **4j**

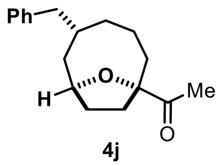

4j

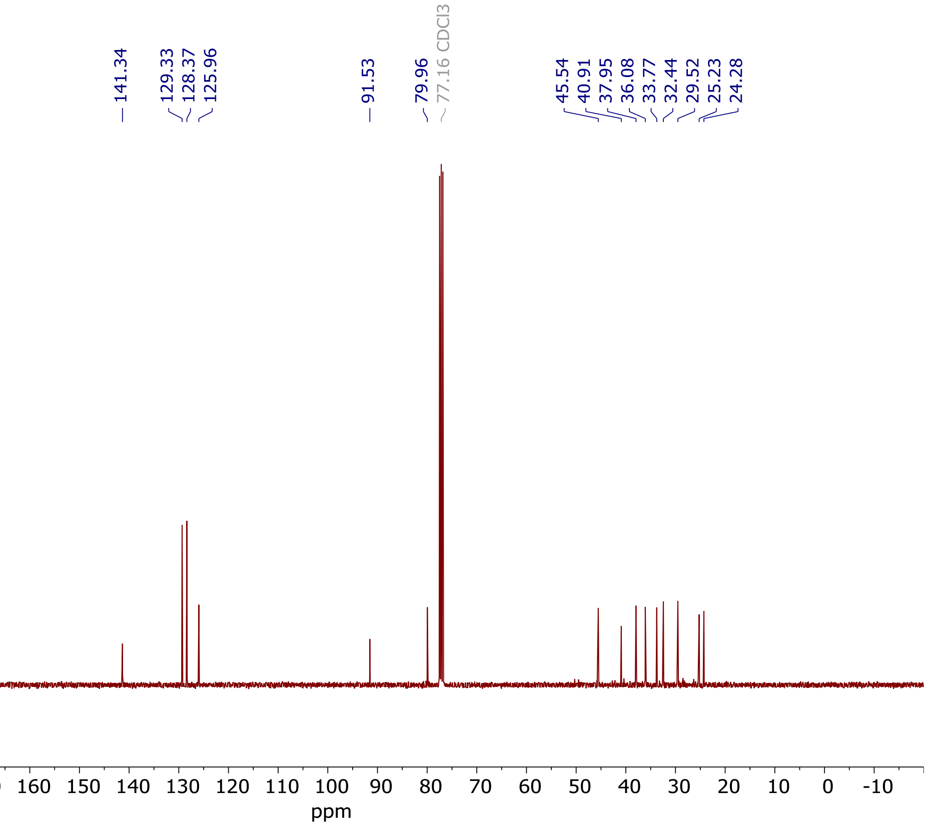

^{1}H NMR (400 MHz, $CDCl_3$) of **4j'**

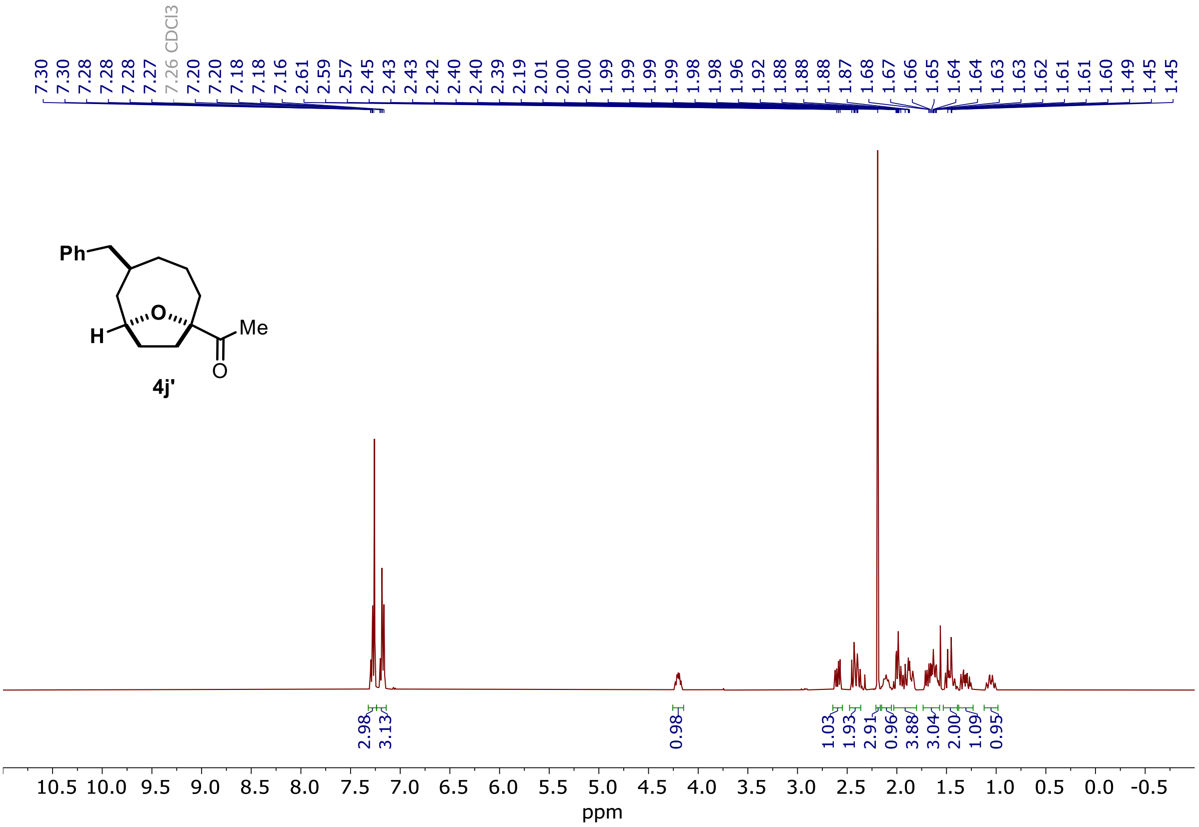

^{13}C NMR (101 MHz, $CDCl_3$) of **4j'**

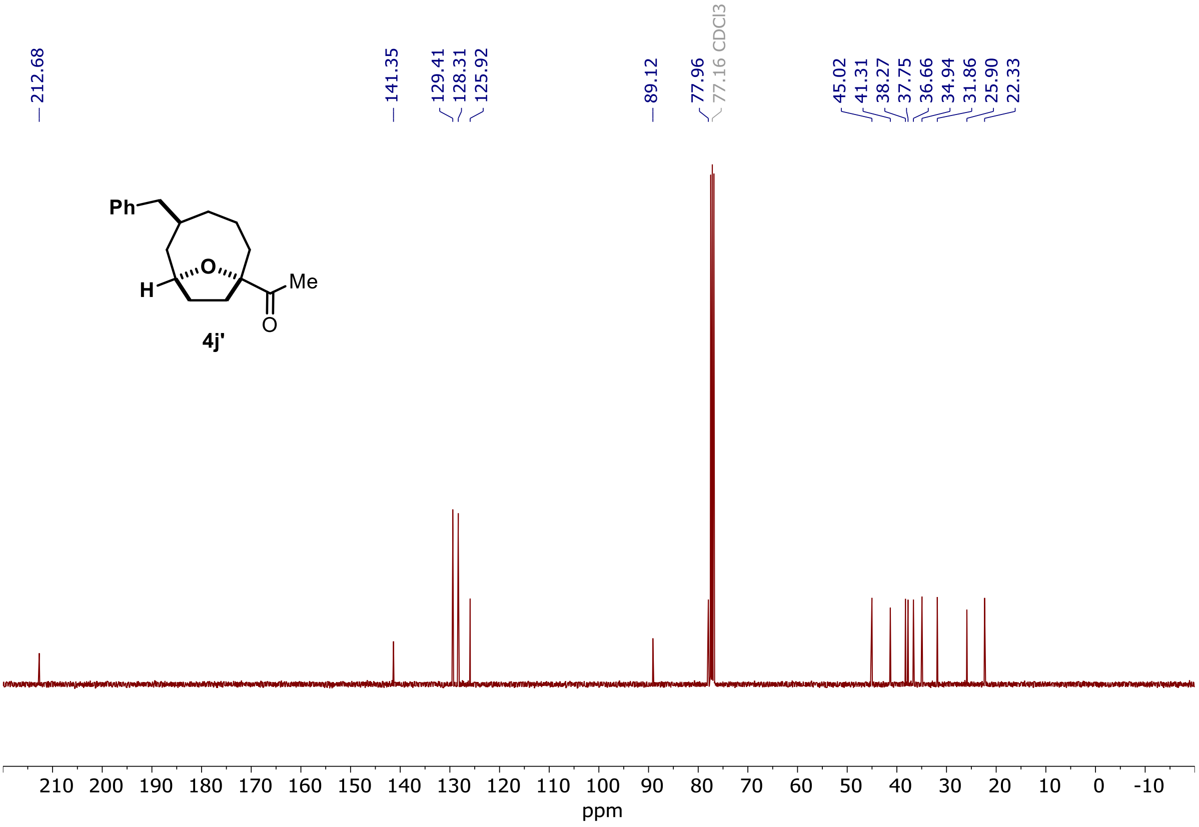

^{1}H NMR (400 MHz, $CDCl_3$) of **4f**

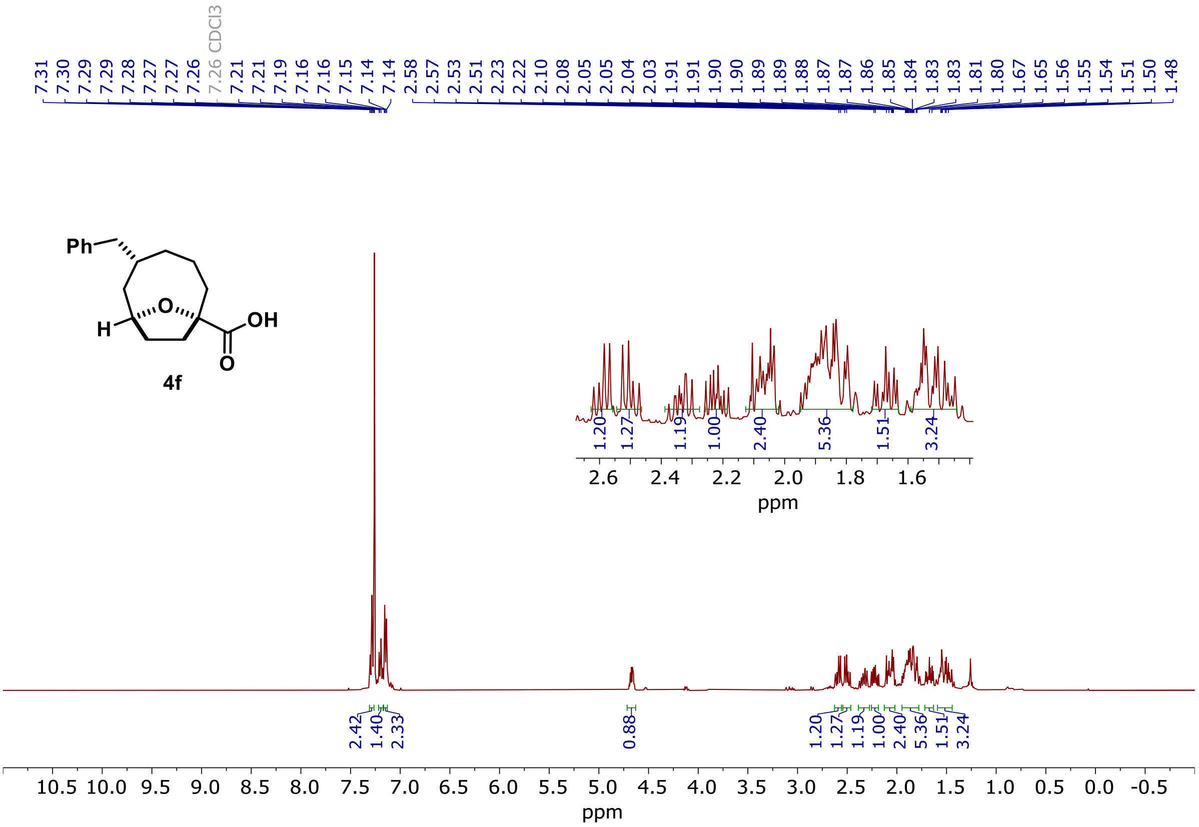

^{13}C NMR (101 MHz, $CDCl_3$) of **4f**

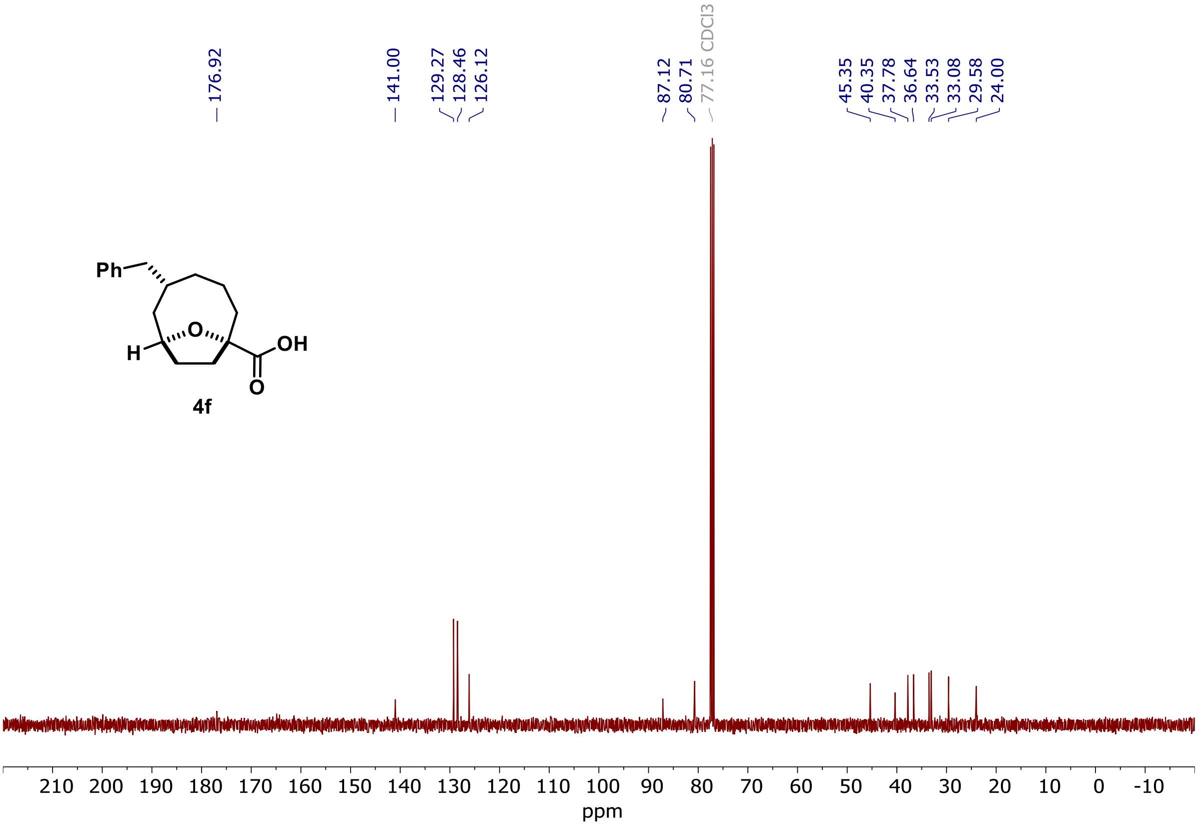

^{1}H NMR (599 MHz, $CDCl_3$) of **4g**

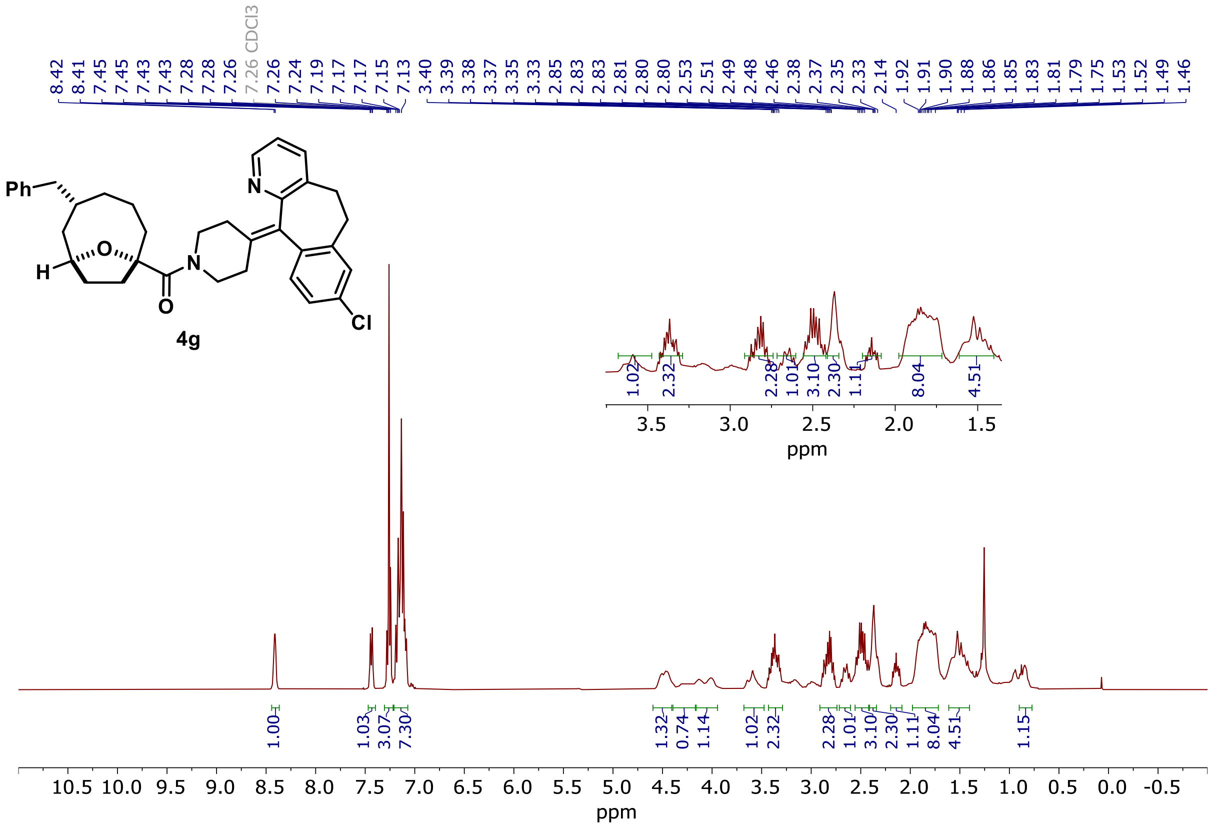

^{13}C NMR (151 MHz, $CDCl_3$) of **4g**

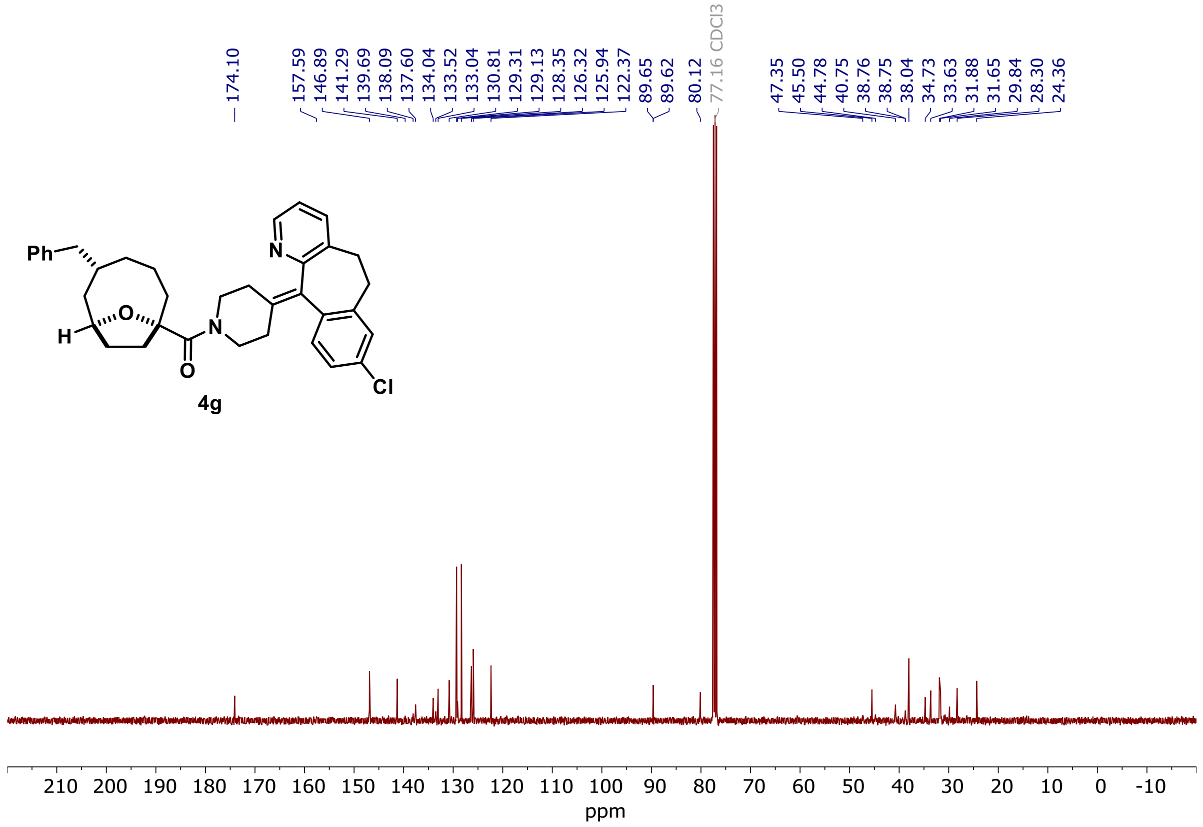

^{1}H NMR (599 MHz, $CDCl_3$) of **4h**

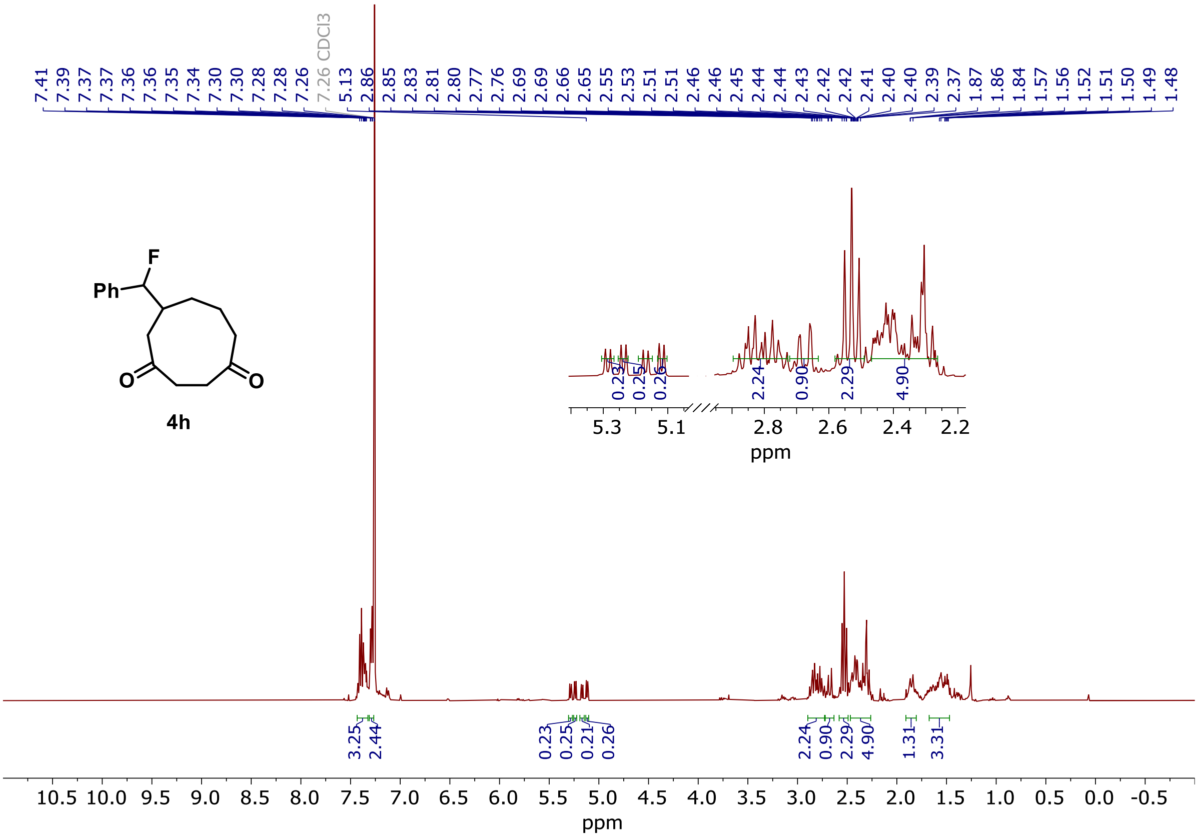

^{13}C NMR (151 MHz, $CDCl_3$) of **4h**

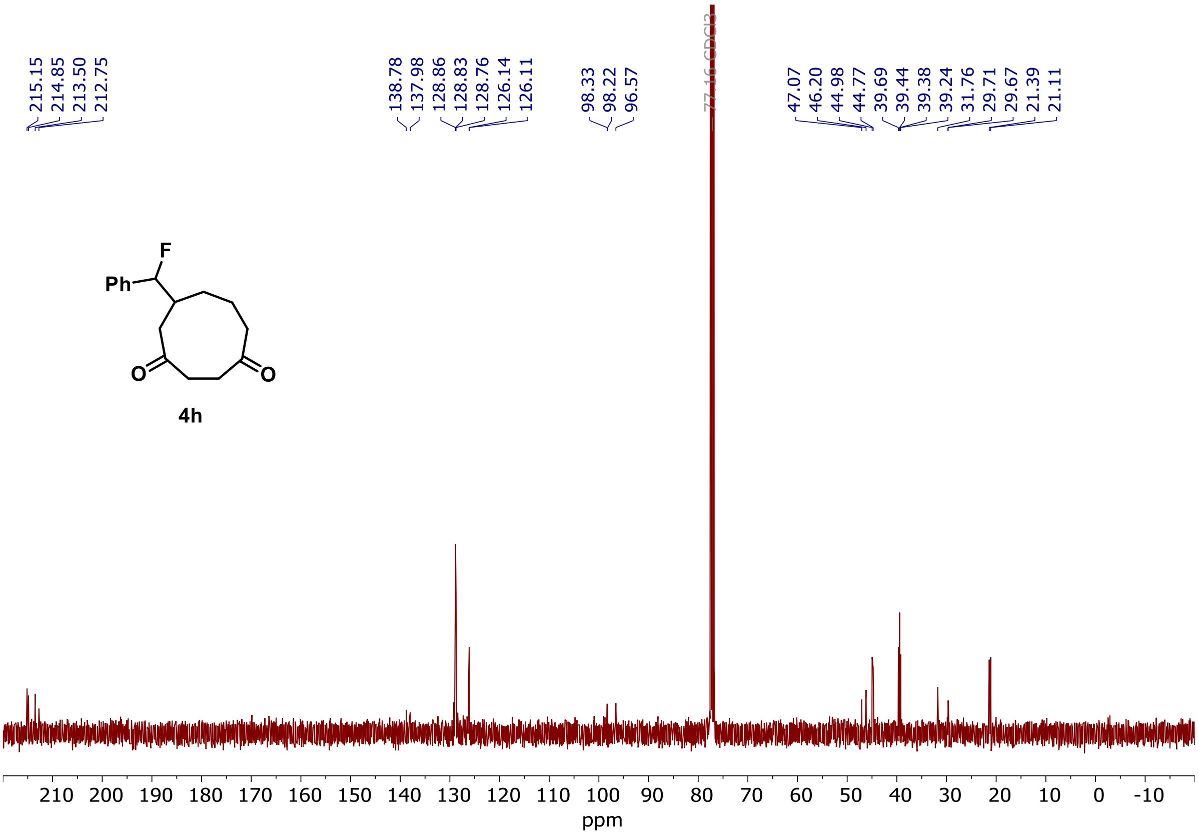

^{19}F NMR (376 MHz, CDCl3) of **4h**

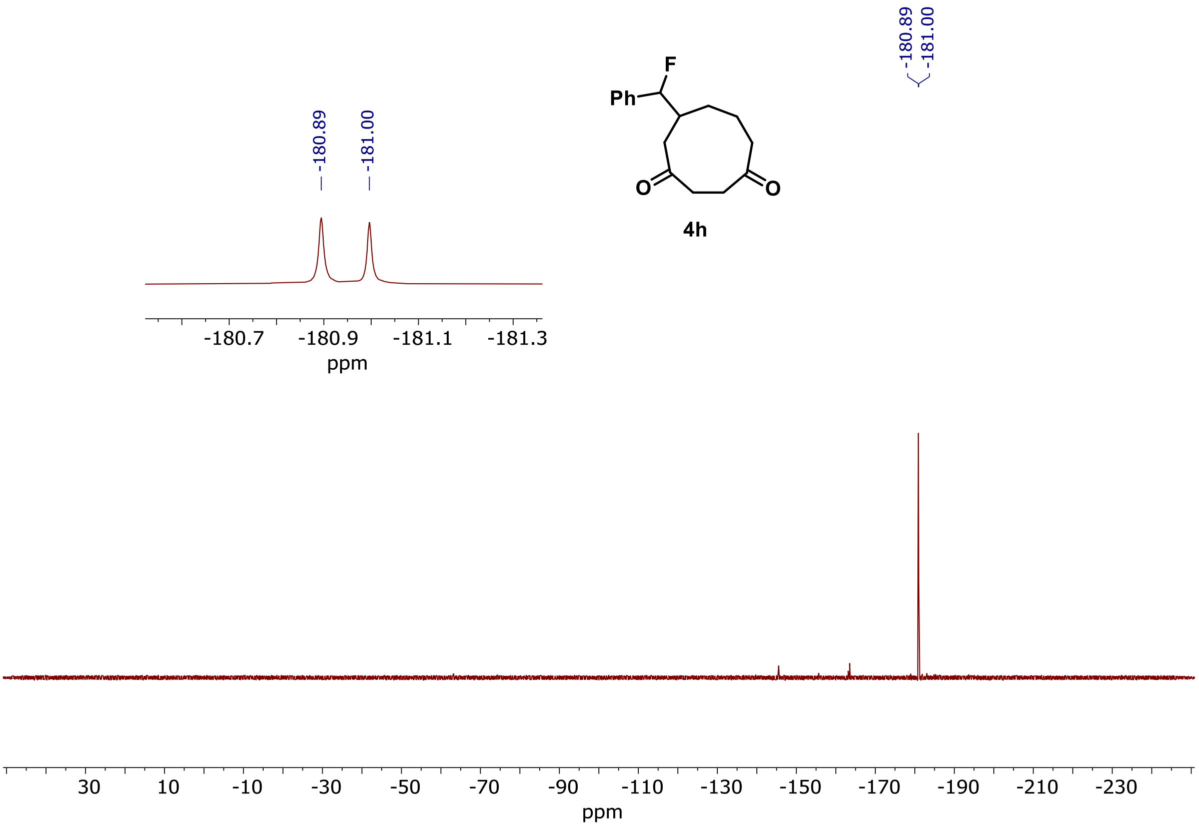

^{1}H NMR (599 MHz, $CDCl_3$) of **4h’**

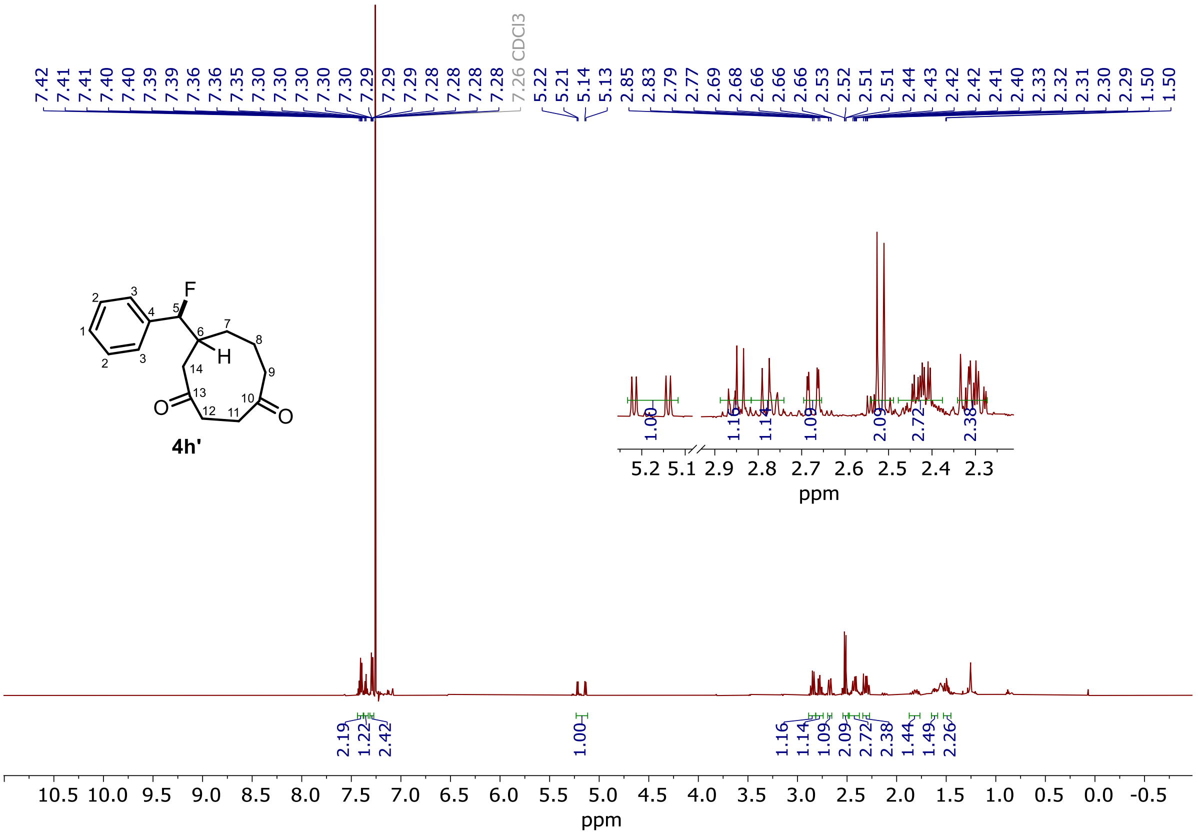

$^{13}C\{^{19}F\}$ NMR (151 MHz, $CDCl_3$) of **4h’**

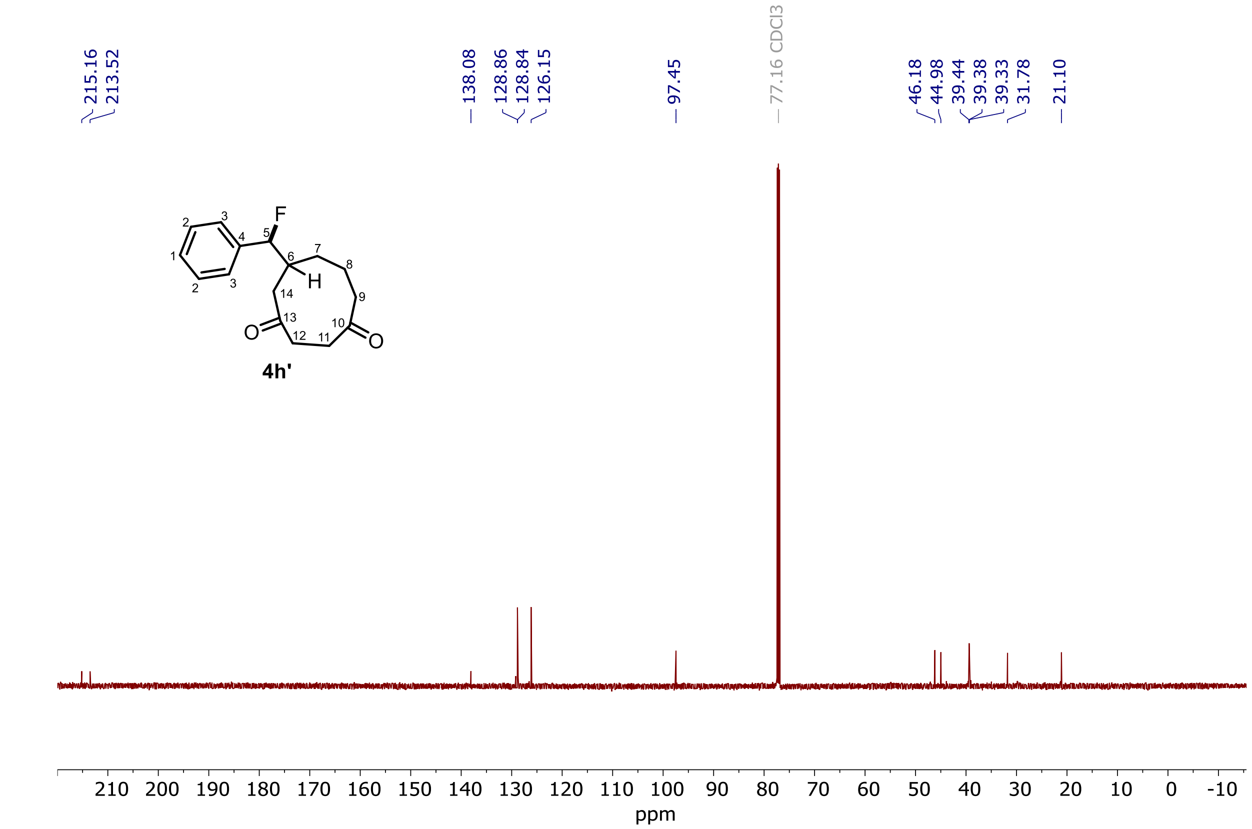

^{13}C NMR (151 MHz, $CDCl_3$) of **4h’**

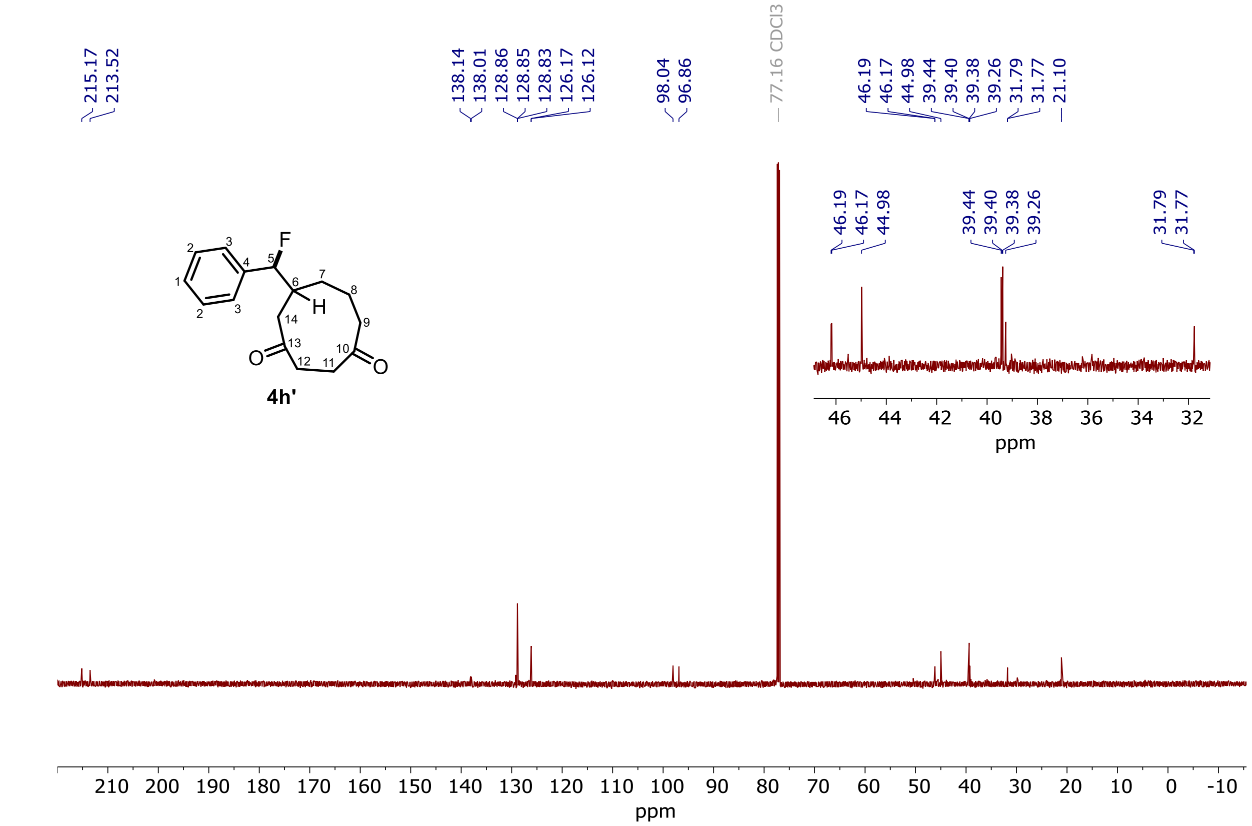

^{19}F NMR (376 MHz, CDCl3) of **4h’**

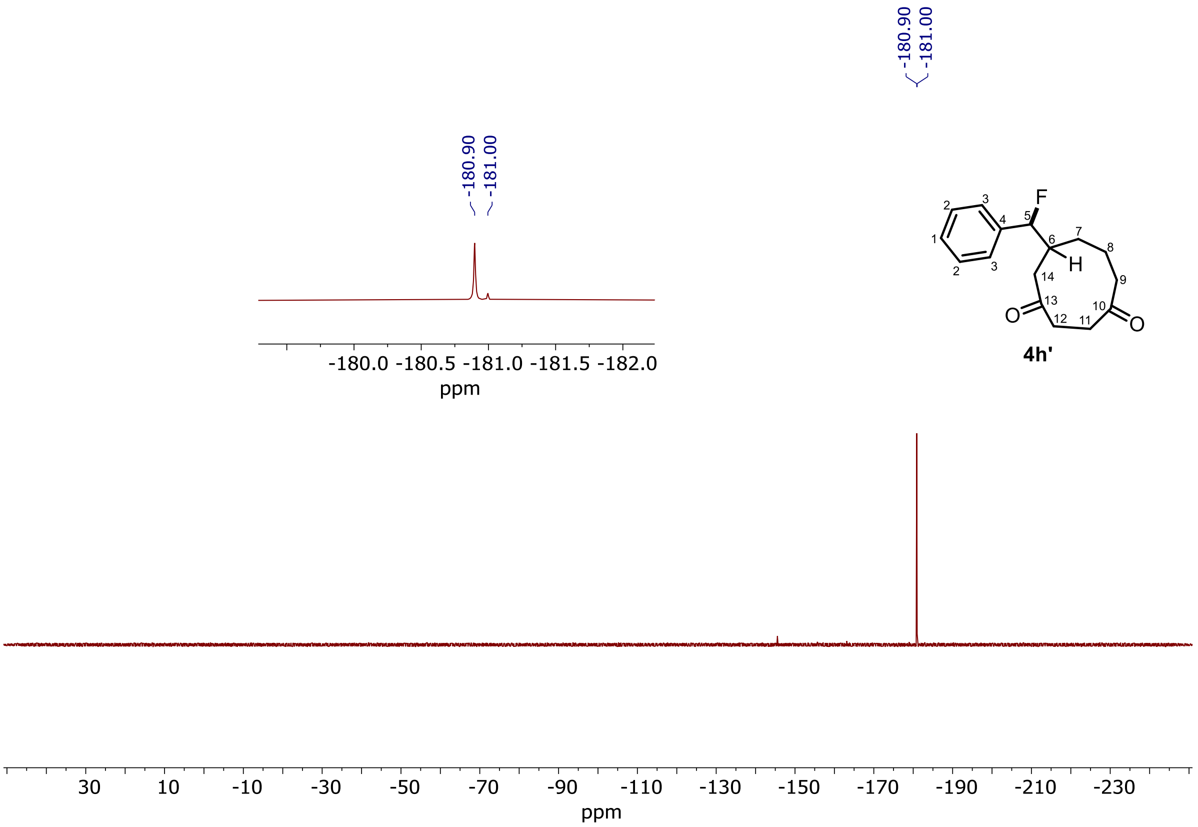

gHSQC of **4h’**

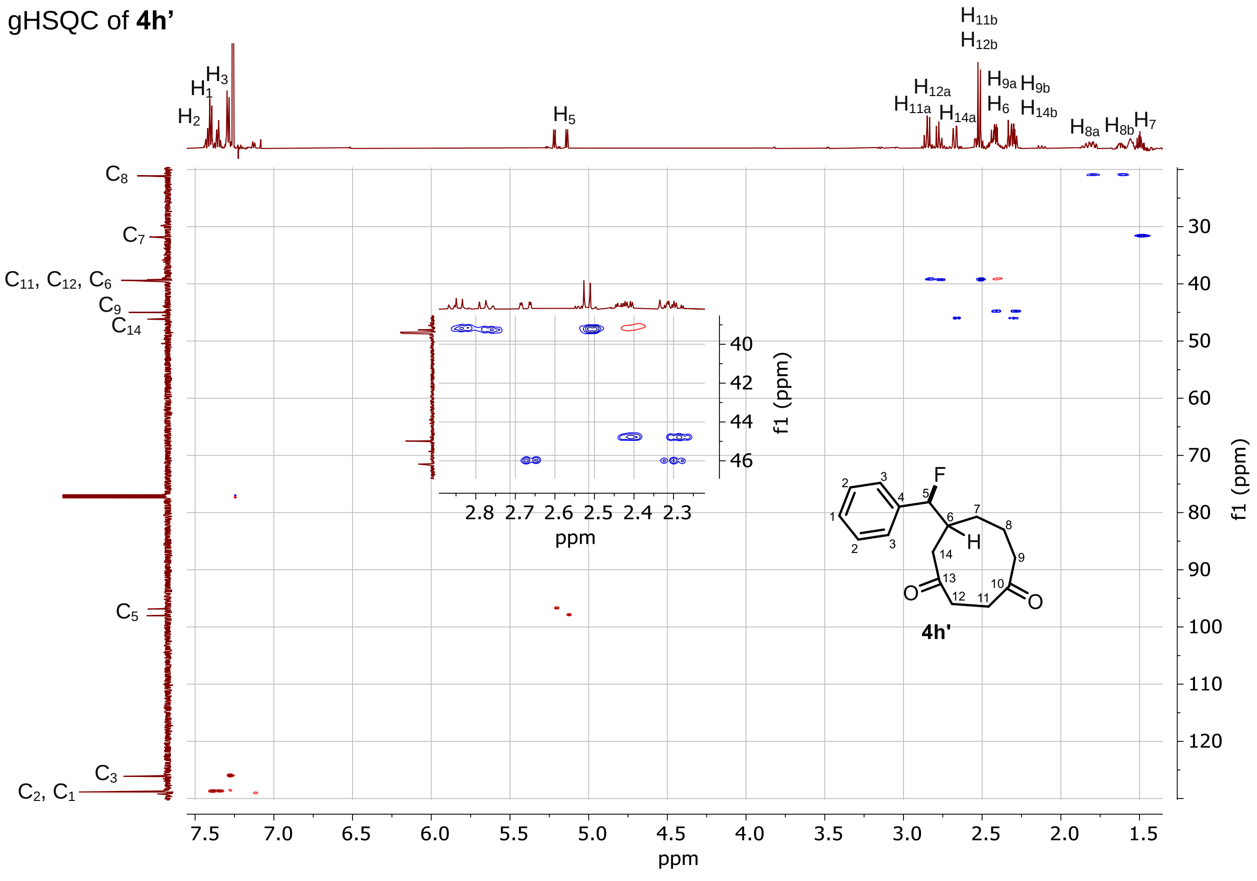

gHMBC of **4h'**

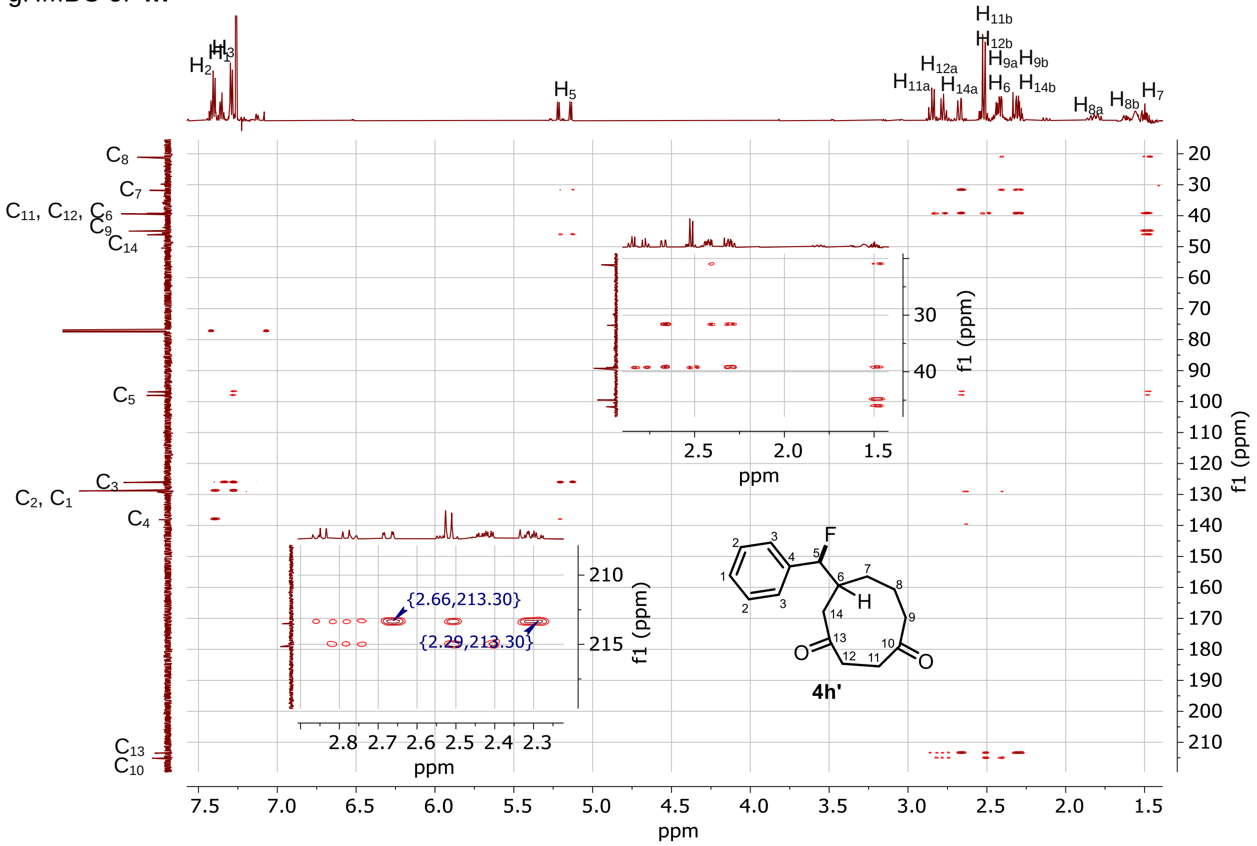

gCOSY of **4h'**

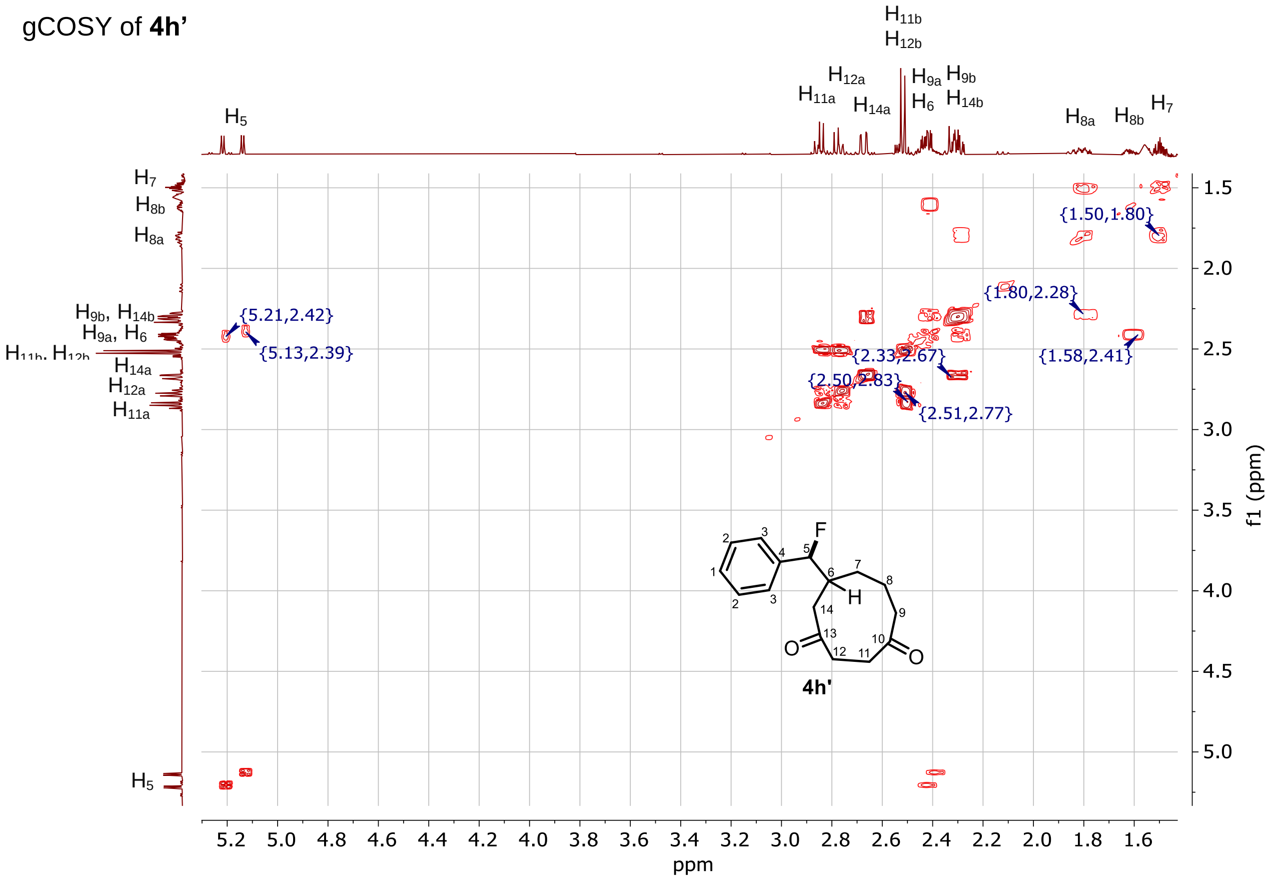

1D-TOCSY

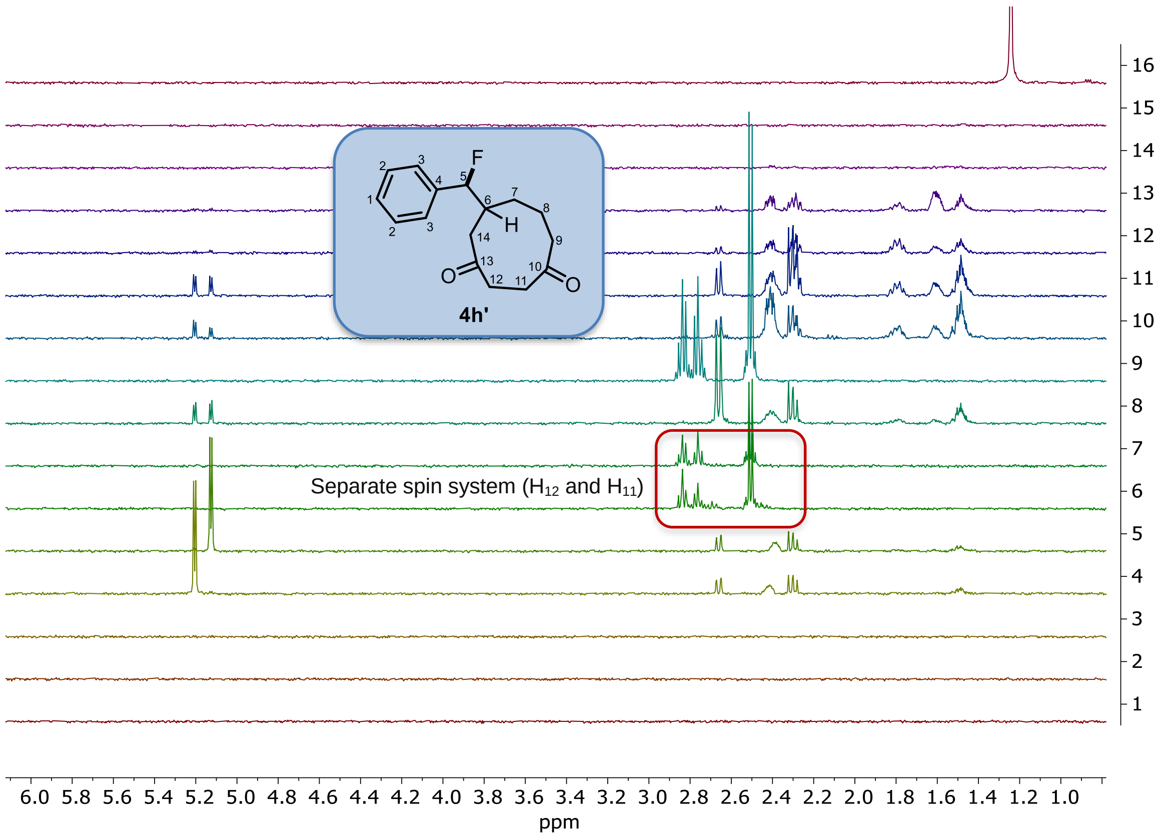

^{1}H NMR (400 MHz, $CDCl_3$) of **4i**

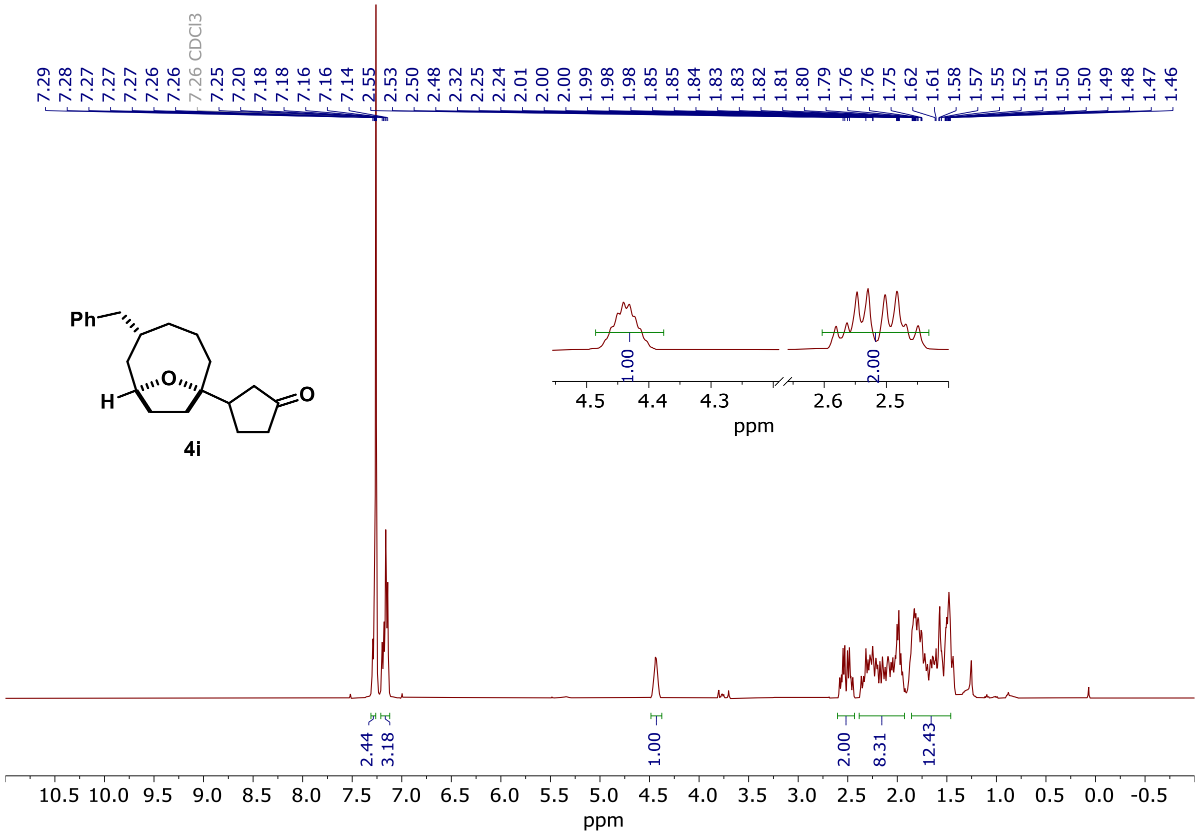

^{13}C NMR (101 MHz, $CDCl_3$) of **4i**

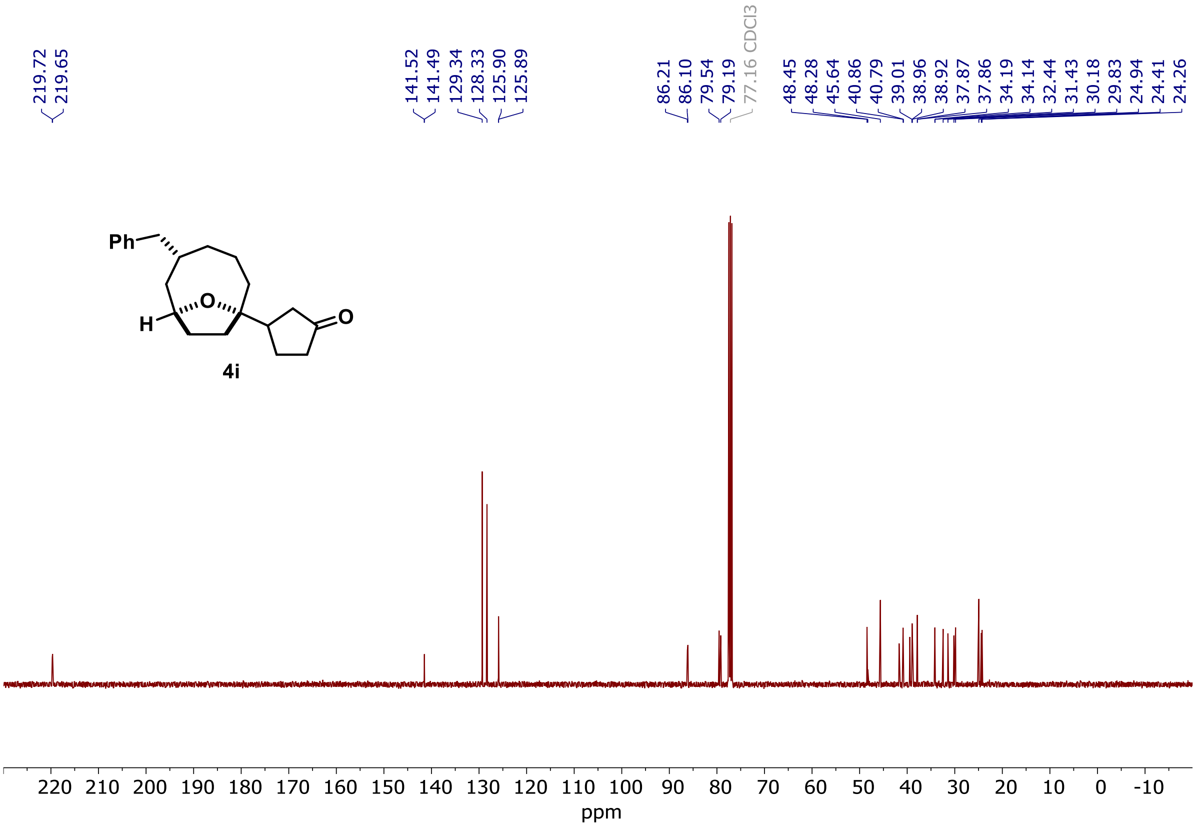

7. REFERENCES

(1) Lowry, M. S.; Goldsmith, J. I.; Slinker, J. D.; Rohl, R.; Pascal, R. A.; Malliaras, G. G.; Bernhard, S. Single-Layer Electroluminescent Devices and Photoinduced Hydrogen Production from an Ionic Iridium(III) Complex. *Chem. Mater.* **2005**, *17*, 5712–5719.

(2) Tamayo, A. B.; Alleyne, B. D.; Djurovich, P. I.; Lamansky, S.; Tsyba, I.; Ho, N. N.; Bau, R.; Thompson, M. E. Synthesis and characterization of facial and meridional tris-cyclometalated iridium(III) complexes. *J. Am. Chem. Soc.* **2003**, *125*, 7377–7387.

(3) Luo, J.; Zhang, J. Donor–Acceptor Fluorophores for Visible-Light-Promoted Organic Synthesis: Photoredox/Ni Dual Catalytic C(sp 3)–C(sp 2) Cross-Coupling. *ACS Catal.* **2016**, *6*, 873–877.

(4) Li, J.; Lu, L.; Pan, Q.; Ren, Y.; Liu, B.; Yin, B. Palladium-Catalyzed Dearomatizing Alkoxydiarylation of Furan Rings by Coupling with Arylboronic Acids: Access to Polysubstituted Oxabicyclic Compounds. *Adv. Synth. Catal.* **2017**, *359*, 2001–2007.

(5) Bream, R. N.; Clark, H.; Edney, D.; Harsanyi, A.; Hayler, J.; Ironmonger, A.; Mc Cleary, N.; Phillips, N.; Priestley, C.; Roberts, A.; Rushworth, P.; Szeto, P.; Webb, M. R.; Wheelhouse, K. Application of C–H Functionalization in the Development of a Concise and Convergent Route to the Phosphatidylinositol-3-kinase Delta Inhibitor Nemiralisib. *Org. Process Res. Dev.* **2021**, *25*, 529–540.

(6) Piou, T.; Slutskyy, Y.; Kevin, N. J.; Sun, Z.; Xiao, D.; Kong, J. Direct Arylation of Azoles Enabled by Pd/Cu Dual Catalysis. *Org. Lett.* **2021**, *23*, 1996–2001.

(7) a) Hashimoto, T.; Kawamata, Y.; Maruoka, K. An organic thiyl radical catalyst for enantioselective cyclization. *Nat. Chem.* **2014**, *6*, 702–705; b) Liu, Y.; Luo, W.; Wang, Z.; Zhao, Y.; Zhao, J.; Xu, X.; Wang, C.; Li, P. Visible-Light Photoredox-Catalyzed Formal 5 + 1 Cycloaddition of N-Tosyl Vinylaziridines with Difluoroalkyl Halides. *Org. Lett.* **2020**, *22*, 9658–9664; c) Moreau, P.; Maffei, M. A stereoselective palladium-catalyzed synthesis of amino alkenyl geminal bisphosphonates. *Tetrahedron Lett.* **2004**, *45*, 743–746; d) Parsons, A. T.; Campbell, M. J.; Johnson, J. S. Diastereoselective synthesis of tetrahydrofurans via Palladium(0)-catalyzed 3 + 2 cycloaddition of vinylcyclopropanes and aldehydes. *Org. Lett.* **2008**, *10*, 2541–2544; e) Rana, D.; Hümpel, C.; Laskar, R.; Schlosser, L.; Korgitzsch, S.; Dutta, S.; Daniliuc, C. G.; Glorius, F. Accelerated Discovery of Energy Transfer-Catalyzed Dearomative Cycloadditions through a Data-Driven Three-Layer Screening Strategy. *J. Am. Chem. Soc.* **2025**, *147*, 28359–28369; f) Wang, S.; Miao, C.; Wang, W.; Lei, Z.; Sun, W. A Salen–Co 3+ Catalyst for the Hydration of Terminal Alkynes and in Tandem Catalysis with Ru–TsDPEN for the One-Pot Transformation of Alkynes into Chiral Alcohols. *ChemCatChem* **2014**, *6*, 1612–1616;

(8) Li, H.-C.; An, C.; Wu, G.; Li, G.-X.; Huang, X.-B.; Gao, W.-X.; Ding, J.-C.; Zhou, Y.-B.; Liu, M.-C.; Wu, H.-Y. Transition-Metal-Free Highly Chemoselective and Stereoselective Reduction with Se/DMF/H2O System. *Org. Lett.* **2018**, *20*, 5573–5577.

(9) Huang, J.; Li, G.-X.; Yang, G.-F.; Fu, D.-Q.; Nie, X.-K.; Cui, X.; Zhao, J.-Z.; Tang, Z. Catalytic asymmetric synthesis of N-substituted tetrahydroquinoxalines via regioselective Heyns rearrangement and stereoselective transfer hydrogenation in one pot. *Chem. Sci.* **2021**, *12*, 4789–4793.

(10) Mascal, M.; Nikitin, E. B. Direct, high-yield conversion of cellulose into biofuel. *Angew. Chem. Int. Ed.* **2008**, *47*, 7924–7926.

(11) Ma, H.; Sun, Z.; Liu, J.; Zhang, X.; Cui, H.; Zhang, Y.; Wang, J. CBr 4 -Mediated Intermolecular Cyclization Reaction: Efficient Synthesis of Substituted N -Acylpyrazoles. *Chin. J. Org. Chem.* **2021**, *41*, 4353.

(12) Zhao, H.; Yin, L.; Cai, M. A Phosphane-Free, Atom-Efficient Cross-Coupling Reaction of Triarylbismuths with Acyl Chlorides Catalyzed by MCM-41-Immobilized Palladium Complex. *Eur. J. Org. Chem.* **2013**, *2013*, 1337–1345.

(13) Peeters, M.; Decaens, J.; Fürstner, A. Taming of Furfurylidenes by Chiral Bismuth-Rhodium Paddlewheel Catalysts. Preparation and Functionalization of Optically Active 1,1-Disubstituted (Trifluoromethyl)cyclopropanes. *Angew. Chem. Int. Ed.* **2023**, *62*, e202311598.

(14) Bai, L.; Wang, J.-X. Reusable, Polymer-Supported, Palladium-Catalyzed, Atom- Efficient Coupling Reaction of Aryl Halides with Sodium Tetraphenylborate in Water by Focused Microwave Irradiation. *Adv. Synth. Catal.* **2008**, *350*, 315–320.

(15) Song, H.-J.; Jiang, W.-T.; Zhou, Q.-L.; Xu, M.-Y.; Xiao, B. Structure-Modified Germatranes for Pd-Catalyzed Biaryl Synthesis. *ACS Catal.* **2018**, *8*, 9287–9291.

(16) Dang, T. T.; Shan, S. P.; Ramalingam, B.; Seayad, A. M. An efficient heterogenized palladium catalyst for N-alkylation of amines and α-alkylation of ketones using alcohols. *RSC Adv.* **2015**, *5*, 42399–42406.

(17) Sumiya, T.; Ishigami, K.; Watanabe, H. Stereoselective synthesis of (±)-urechitol A employing [4+3] cycloaddition. *Tetrahedron* **2016**, *72*, 6982–6987.

(18) Taveras, A. G.; Aki, C. J.; Bond, R. W.; Chao, J.; Dwyer, M.; Ferreira, J. A.; Chao, J.; Yu, Y.; Baldwin, J. J.; Kaiser, B.; Li, G.; Merritt, J. R.; Biju, P. J.; Nelson, K. H.; Rokosz, L. L.; Jakway, J. P.; Lai, G.; Wu, M.; Hecker, E. A.; Lundell, D.; Fine, J. S.; Nelson, K. H., JR. 3,4-Di-substituted cyclobutene-1,2-diones as CXC-chemokine receptor ligands. US2004147559 (A1), 2004.

(19) Deepak, N. M. S.; Kutateladze, A. G.; Elyashberg, M.; Williams, C. M.; Bates, R. W. Arneroma B: Structure reassignment and total synthesis. *Tetrahedron* **2023**, *147*, 133670.

(20) Ohta, Y.; Doe, M.; Morimoto, Y.; Kinoshita, T. Regiospecific synthesis of 2-substituted furanonaphthoquinones. *J. Heterocycl. Chem.* **2000**, *37*, 731–734.

(21) Yuan, D.; Liu, S.; Li, S.; Liu, R.; Zhu, X. Design, Synthesis and Biological Evaluation of 7-Substituted-1,3-diaminopyrrol3,2-fquinazolines as Potential Antibacterial Agents. *ChemMedChem* **2023**, *18*, e202300078.

(22) Zhou, J.; Fu, G. C. Suzuki cross-couplings of unactivated secondary alkyl bromides and iodides. *J. Am. Chem. Soc.* **2004**, *126*, 1340–1341.

(23) Miralinaghi, P.; Salimi, M.; Amirhamzeh, A.; Norouzi, M.; Kandelousi, H. M.; Shafiee, A.; Amini, M. Synthesis, molecular docking study, and anticancer activity of triaryl-1,2,4-oxadiazole. *Med. Chem. Res.* **2013**, *22*, 4253–4262.

(24) Brossard, D.; Lechevrel, M.; El Kihel, L.; Quesnelle, C.; Khalid, M.; Moslemi, S.; Reimund, J.-M. Synthesis and biological evaluation of bile carboxamide derivatives with pro-apoptotic effect on human colon adenocarcinoma cell lines. *Eur. J. Med. Chem.* **2014**, *86*, 279–290.

(25) Mayorquín-Torres, M. C.; Iglesias-Arteaga, M. A. Application of palladium-catalyzed cross-coupling between bile acids and 2-furanylboronic acid to the synthesis of 24-(2'-furanyl)-24-oxo steroids. *Steroids* **2020**, *162*, 108689.

(26) Lv, X.-Y.; Abrams, R.; Martin, R. Dihydroquinazolinones as adaptative C(sp3) handles in arylations and alkylations via dual catalytic C-C bond-functionalization. *Nat. Commun.* **2022**, *13*, 2394.

(27) Huang, H.; Yuan, G.; Li, X.; Jiang, H. Electrochemical synthesis of amides: direct transformation of methyl ketones with formamides. *Tetrahedron Lett.* **2013**, *54*, 7156–7159.

(28) Marzo, L.; Wang, S.; König, B. Visible-Light-Mediated Radical Arylation of Anilines with Acceptor-Substituted (Hetero)aryl Halides. *Org. Lett.* **2017**, *19*, 5976–5979.

(29) Juwaini, N. A. B.; Ng, J. K. P.; Seayad, J. Catalytic Regioselective Oxidative Coupling of Furan-2-Carbonyls with Simple Arenes. *ACS Catal.* **2012**, *2*, 1787–1791.

(30) Gryparis, C.; Lykakis, I. N.; Efe, C.; Zaravinos, I.-P.; Vidali, T.; Kladou, E.; Stratakis, M. Functionalized 3(2H)-furanones via photooxygenation of (β-keto)-2-substituted furans: application to the biomimetic synthesis of merrekentrone C. *Org. Biomol. Chem.* **2011**, *9*, 5655–5658.

(31) Bitai, J.; Nimmo, A. J.; Slawin, A. M. Z.; Smith, A. D. Cooperative Palladium/Isothiourea Catalyzed Enantioselective Formal (3+2) Cycloaddition of Vinylcyclopropanes and α,β-Unsaturated Esters. *Angew. Chem. Int. Ed.* **2022**, *61*, e202202621.

(32) He, T.; Wang, G.; Long, P.-W.; Kemper, S.; Irran, E.; Klare, H. F. T.; Oestreich, M. Intramolecular Friedel-Crafts alkylation with a silylium-ion-activated cyclopropyl group: formation of tricyclic ring systems from benzyl-substituted vinylcyclopropanes and hydrosilanes. *Chem. Sci.* **2020**, *12*, 569–575.

(33) Donlin, M. J.; Zunica, A.; Lipnicky, A.; Garimallaprabhakaran, A. K.; Berkowitz, A. J.; Grigoryan, A.; Meyers, M. J.; Tavis, J. E.; Murelli, R. P. Troponoids Can Inhibit Growth of the Human Fungal Pathogen Cryptococcus neoformans. *Antimicrob. Agents Chemother.* **2017**, *61*.

(34) Zhu, M.; Gao, Y.-J.; Huang, X.-L.; Li, M.; Zheng, C.; You, S.-L. Photo-induced intramolecular dearomative 5 + 4 cycloaddition of arenes for the construction of highly strained medium-sized-rings. *Nat. Commun.* **2024**, *15*, 2462.

(35) Baumann, A. N.; Schüppel, F.; Eisold, M.; Kreppel, A.; Vivie-Riedle, R. de; Didier, D. Oxidative Ring Contraction of Cyclobutenes: General Approach to Cyclopropylketones including Mechanistic Insights. *J. Org. Chem.* **2018**, *83*, 4905–4921.

(36) Ventre, S.; Petronijevic, F. R.; MacMillan, D. W. C. Decarboxylative Fluorination of Aliphatic Carboxylic Acids via Photoredox Catalysis. *J. Am. Chem. Soc.* **2015**, *137*, 5654–5657.

(37) Chu, L.; Ohta, C.; Zuo, Z.; MacMillan, D. W. C. Carboxylic acids as a traceless activation group for conjugate additions: a three-step synthesis of (±)-pregabalin. *J. Am. Chem. Soc.* **2014**, *136*, 10886–10889.

(38) Kleinmans, R.; Pinkert, T.; Dutta, S.; Paulisch, T. O.; Keum, H.; Daniliuc, C. G.; Glorius, F. Intermolecular 2π+2σ-photocycloaddition enabled by triplet energy transfer. *Nature* **2022**, *605*, 477–482.

(39) Schlosser, L.; Rana, D.; Pflüger, P.; Katzenburg, F.; Glorius, F. EnTdecker - A Machine Learning-Based Platform for Guiding Substrate Discovery in Energy Transfer Catalysis. *J. Am. Chem. Soc.* **2024**, *146*, 13266–13275.

(40) Elliott, L. D.; Kayal, S.; George, M. W.; Booker-Milburn, K. Rational Design of Triplet Sensitizers for the Transfer of Excited State Photochemistry from UV to Visible. *J. Am. Chem. Soc.* **2020**, *142*, 14947–14956.

(41) Pitzer, L.; Schäfers, F.; Glorius, F. Rapid Assessment of the Reaction-Condition-Based Sensitivity of Chemical Transformations. *Angew. Chem. Int. Ed.* **2019**, *58*, 8572–8576.

(42) Collins, K. D.; Glorius, F. A robustness screen for the rapid assessment of chemical reactions. *Nat. Chem.* **2013**, *5*, 597–601.

(43) Cismesia, M. A.; Yoon, T. P. Characterizing chain processes in visible light photoredox catalysis. *Chem. Sci.* **2015**, *6*, 5426–5434.

(44) C. G. Hatchard and C. A. Parker. A new sensitive chemical actinometer - II. Potassium ferrioxalate as a

standard chemical actinometer. *Proc. R. Soc. Lond. A Math. Phys. Sci.* **1956**, *235*, 518–536.

(45) Pozdnyakov, I. P.; Kel, O. V.; Plyusnin, V. F.; Grivin, V. P.; Bazhin, N. M. Reply to “Comment on ‘New insight into photochemistry of ferrioxalate’”. *J. Phys. Chem. A* **2009**, *113*, 8820–8822.

(46) Wegner, E. E.; Adamson, A. W. Photochemistry of complex ions. III. Absolute quantum yields for the photolysis of some aqueous Chromium(III) complexes. Chemical actinometry in the long wavelength visible region. *J. Am. Chem. Soc.* **1966**, *88*, 394–404.

(47) a) Neese, F. The ORCA program system. *WIREs Comput. Mol. Sci.* **2012**, *2*, 73–78; b) Neese, F. Software update: The ORCA program system—Version 5.0. *WIREs Comput. Mol. Sci.* **2022**, *12*;

(48) Becke, A. D. Density-functional thermochemistry. III. The role of exact exchange. *J. Chem. Phys.* **1993**, *98*, 5648–5652.

(49) Weigend, F.; Ahlrichs, R. Balanced basis sets of split valence, triple zeta valence and quadruple zeta valence quality for H to Rn: Design and assessment of accuracy. *Phys. Chem. Chem. Phys.* **2005**, *7*, 3297–3305.

(50) Barone, V.; Cossi, M. Quantum Calculation of Molecular Energies and Energy Gradients in Solution by a Conductor Solvent Model. *J. Phys. Chem. A* **1998**, *102*, 1995–2001.

(51) Cossi, M.; Rega, N.; Scalmani, G.; Barone, V. Energies, structures, and electronic properties of molecules in solution with the C-PCM solvation model. *J. Comput. Chem.* **2003**, *24*, 669–681.

(52) a) Grimme, S.; Antony, J.; Ehrlich, S.; Krieg, H. A consistent and accurate ab initio parametrization of density functional dispersion correction (DFT-D) for the 94 elements H-Pu. *J. Chem. Phys.* **2010**, *132*, 154104; b) Grimme, S.; Ehrlich, S.; Goerigk, L. Effect of the damping function in dispersion corrected density functional theory. *J. Comput. Chem.* **2011**, *32*, 1456–1465;

(53) Bannwarth, C.; Ehlert, S.; Grimme, S. GFN2-xTB-An Accurate and Broadly Parametrized Self-Consistent Tight-Binding Quantum Chemical Method with Multipole Electrostatics and Density-Dependent Dispersion Contributions. *J. Chem. Theory Comput.* **2019**, *15*, 1652–1671.

(54) Ásgeirsson, V.; Birgisson, B. O.; Bjornsson, R.; Becker, U.; Neese, F.; Riplinger, C.; Jónsson, H. Nudged Elastic Band Method for Molecular Reactions Using Energy-Weighted Springs Combined with Eigenvector Following. *J. Chem. Theory Comput.* **2021**, *17*, 4929–4945.

(55) Chai, J.-D.; Head-Gordon, M. Long-range corrected hybrid density functionals with damped atom-atom dispersion corrections. *Phys. Chem. Chem. Phys.* **2008**, *10*, 6615–6620.

(56) Bruker. *SAINT, V8.41;* Bruker AXS Inc., Madison, Winsconsin, USA.

(57) Krause, L.; Herbst-Irmer, R.; Sheldrick, G. M.; Stalke, D. Comparison of silver and molybdenum microfocus X-ray sources for single-crystal structure determination. *J. Appl. Crystallogr.* **2015**, *48*, 3–10.

(58) Sheldrick, G. M. Crystal structure refinement with SHELXL. *Acta Cryst.* **2015**, *71*, 3–8.

(59) Sheldrick, G. M. SHELXT - integrated space-group and crystal-structure determination. *Acta Cryst.* **2015**, *71*, 3–8.

(60) Groom, C. R.; Bruno, I. J.; Lightfoot, M. P.; Ward, S. C. The Cambridge Structural Database. *Acta Cryst.* **2016**, *72*, 171–179.

(61) Kratzert D. *FinalCif, (Bruker Edition)*. https://dkratzert.de/finalcif.html. https://dkratzert.de/finalcif.html.
